# Growth study under combined effects of temperature, pH and salinity and transcriptome analysis revealed adaptations of *Aspergillus terreus* NTOU4989 to the extreme conditions at Kueishan Island Hydrothermal Vent Field, Taiwan

**DOI:** 10.1371/journal.pone.0233621

**Published:** 2020-05-26

**Authors:** Ka-Lai Pang, Michael Wai-Lun Chiang, Sheng-Yu Guo, Chi-Yu Shih, Hans U. Dahms, Jiang-Shiou Hwang, Hyo-Jung Cha

**Affiliations:** 1 Institute of Marine Biology and Centre of Excellence for the Oceans, National Taiwan Ocean University, Keelung, Taiwan; 2 Department of Chemistry, City University of Hong Kong, Kowloon Tong, Hong Kong; 3 Department of Biomedical Science and Environment Biology, Kaohsiung Medical University, Kaohsiung, Taiwan; Sidra Medicine, QATAR

## Abstract

A high diversity of fungi was discovered on various substrates collected at the marine shallow-water Kueishan Island Hydrothermal Vent Field, Taiwan, using culture and metabarcoding methods but whether these fungi can grow and play an active role in such an extreme environment is unknown. We investigated the combined effects of different salinity, temperature and pH on growth of ten fungi (in the genera *Aspergillus*, *Penicillium*, *Fodinomyces*, *Microascus*, *Trichoderma*, *Verticillium*) isolated from the sediment and the vent crab *Xenograpsus testudinatus*. The growth responses of the tested fungi could be referred to three groups: (1) wide pH, salinity and temperature ranges, (2) salinity-dependent and temperature-sensitive, and (3) temperature-tolerant. *Aspergillus terreus* NTOU4989 was the only fungus which showed growth at 45 °C, pH 3 and 30 ‰ salinity, and might be active near the vents. We also carried out a transcriptome analysis to understand the molecular adaptations of *A*. *terreus* NTOU4989 under these extreme conditions. Data revealed that stress-related genes were differentially expressed at high temperature (45 °C); for instance, mannitol biosynthetic genes were up-regulated while glutathione S-transferase and amino acid oxidase genes down-regulated in response to high temperature. On the other hand, hydrogen ion transmembrane transport genes and phenylalanine ammonia lyase were up-regulated while pH-response transcription factor was down-regulated at pH 3, a relative acidic environment. However, genes related to salt tolerance, such as glycerol lipid metabolism and mitogen-activated protein kinase, were up-regulated in both conditions, possibly related to maintaining water homeostasis. The results of this study revealed the genetic evidence of adaptation in *A*. *terreus* NTOU4989 to changes of environmental conditions.

## Introduction

Our understanding on the diversity of microorganisms of marine shallow-water and deep-sea hydrothermal vent ecosystems is improving. While prokaryotes (bacteria and archaea) dominate at hydrothermal vent ecosystems [1], footprint of fungal presence was discovered from various substrates of this extreme habitat through the use of culture-dependent and -independent techniques. At various deep-sea hydrothermal vent sites in the Pacific and Atlantic Oceans, ninety-seven fungal isolates were cultured from 210 samples of animal, sediment, rock and seawater samples; most isolates belonged to the Ascomycota while only one basidiomycete was isolated (*Tilletiopsis* sp.) [2]. The Ascomycota was also dominant in sulfide and black smoker samples at a deep-sea hydrothermal vent site located near the Mid-Atlantic Ridge of the South Atlantic Ocean: 129 isolates belonging to the Ascomycota and 32 isolates to the Basidiomycota [3]. In contrast, mainly basidiomycetous yeasts were cultured from Fe-oxide mats and basalt rock surfaces at the active volcano, Vailulu’u seamount, Samoa [4]. Yeasts have been found dominant on substrates collected at hydrothermal vent sites, suggesting this growth form may be ecologically advantageous in these extreme habitats [5,6]. In addition to the Ascomycota and the Basidiomycota, sequence signatures of the Chytridiomycota were recovered from animal and rock samples of deep-sea hydrothermal vents at the East Pacific Rise and the Mid-Atlantic Ridge using PCR-cloning-sequencing analysis [7]. However, information on whether these fungal groups are active in these hydrothermal vent ecosystems is deficient. Burgaud and co-workers investigated growth of yeast isolates cultured from animals collected at a hydrothermal vent site at the Mid-Atlantic Ridge in response to different salinities and temperatures, and found that majority of the isolates grew at low salinity conditions (0–15 g/L salt) and 25 °C while two isolates were able to grow at high salinities (30 and 45 g/L salt) and 35 °C [6].

Environmental conditions of marine shallow-water hydrothermal vents are different from those of the deep-sea vents, in terms of temperature (up to 119 °C in shallow-water vents, exceed 400 °C in deep-sea vents), chemistry of vent fluids, primary production (chemoautotrophy at deep-sea vents, chemoautotrophy and photoautotrophy at shallow-water vents), hydrostatic pressure, among others [8]. Kueishan Island is a volcanic island lying just outside Yilan County, Taiwan with roughly 50 shallow-water hydrothermal vent discharges of depths ranging from 10 m to 80 m, constantly emitting hydrothermal fluids (fluid temperature between 48 °C and 116 °C) and volcanic gases (carbon dioxide and hydrogen sulfide) [9,10]. Our earlier study reported 25 fungal species isolated from yellow sediment, 23 from black sediment, 13 from the vent crab *Xenograpsus testudinatus* and 2 species each from seawater and animal egg samples collected at/near the hydrothermal vents of Kueishan Island [11]. The ecological origin of these fungi (terrestrial or marine) and whether these fungi can grow at/near the vents are unknown.

In this study, we hypothesize that the fungi isolated at/near the vent area are able to grow at a high temperature and a low pH, possible conditions of hydrothermal vent environments. Selected fungi isolated from substrates collected at/near the hydrothermal vents of Kueishan Island [11] were tested on their growth under the combined effects of pH (1, 3, 5, 7, 9) and temperature (15, 25, 37, 45 °C) in both freshwater (0 ‰) and seawater (30 ‰) media: six fungal species from the yellow sediment (with sulfur granules, characteristics of the sediment near vents) and two each from the black sediment and the vent crab *X*. *testudinatus*. The bottom water temperature and pH at the hydrothermal vents of Kueishan Island were found to be around 27–30 °C and pH 7.2, respectively [12]. However, in order to test if the fungi could grow at a low pH, the experimental pH values were selected from acidic (sediment of hydrothermal vent environment) to slightly alkaline (normal seawater) pH. The four tested temperatures with a ~10 °C interval were chosen based on the lowest water temperature recorded at Kueishan Island (~17 °C within 01/02/2014–31/12/2017, http://opendata.cwb.gov.tw) and the highest growth temperature by any of our selected fungi from a preliminary experiment (45 °C, results not shown). Growth of these fungi was tested in a freshwater medium (as well as a seawater medium) as some of the selected fungi are well known terrestrial fungi but can be found in the marine environment.

*Aspergillus terreus* NTOU4989 was found to be able to grow at 45 °C, pH 3 and 30 ‰ salinity, and its transcriptome expressed under these conditions was analyzed by RNA-seq to determine its physiological adaptations at the molecular level over the control conditions (25 °C, pH 7, 30 ‰) based on a median water temperature at Kueishan Island (17 C-31.3 °C between 01/02/2014-31/12/2017, http://opendata.cwb.gov.tw) and a pH close to seawater. The objectives of this study were: (1) to investigate growth of selected fungi isolated from substrates collected at/near the hydrothermal vents of Kueishan Island under the combined effects of pH (1, 3, 5, 7, 9), temperature (15, 25, 37, 45 °C) and salinity (0 ‰, 30 ‰), and (2) to reveal the molecular adaptations of *A*. *terreus* NTOU4989 at 45 °C, pH 3 and 30 ‰ over at 25 °C, pH 7 and 30 ‰ by RNA-seq.

## Materials and methods

### Fungal isolates

Ten fungi isolated from the sediment and vent crab (*Xenograpsus testudinatus*) samples collected at/near the hydrothermal vent system at Kueishan Island (121°57'6.01" E, 24°50'30.98" N) between 2015 and 2017 on GYPS (0.4% glucose (BioShop, Ontario, Canada), 0.4% yeast extract (Oxoid, Hampshire, England), 0.4% peptone (Oxoid, Hampshire, England), 30 g sea salt, 1 L distilled water) or one-fifth strength of CDS (Czapek-Dox agar prepared with natural seawater, Difco^™^, BD, Sparks, MD, USA) were selected for the growth study [11]. No permit was required to collect the samples as the collection site is not a national park. Identification of these fungi was based on sequencing of the internal transcribed spacers of rDNA (ITS) and a comparison of these sequences with the reference sequences in the GenBank. These fungi included *Aspergillus terreus* NTOU4989, *A*. *aculeatus* NTOU4990, *Penicillium matriti* NTOU4992, *P*. *sumatrense* NTOU5001, *P*. *oxalicum* NTOU5115, *Fodinomyces uranophilus* NTOU5428 from the yellow sediment samples (with sulfur granules); *Verticillium dahlia* NTOU4998 and *Trichoderma harzianum* NTOU5005 from the black sediment samples; *Aspergillus sydowii* NTOU4991 and *Microascus brevicaulis* NTOU5292 from the vent crab samples.

### Combined effects of pH, temperature and salinity on growth

All isolates were subcultured on GYPS agar plates for 2 weeks, where aerial spores were formed abundantly on the medium. Three milliliters of Tween 20 (0.1%, Honeywell Fluka, Seelze, Germany) were added on top of the fungal colony. The plates were gently shaken to dislodge the spores. Concentrations of the spore suspension were counted using a haemocytometer and adjusted to 2×10^4^ spores/ml for all isolates.

**Fig 1:**
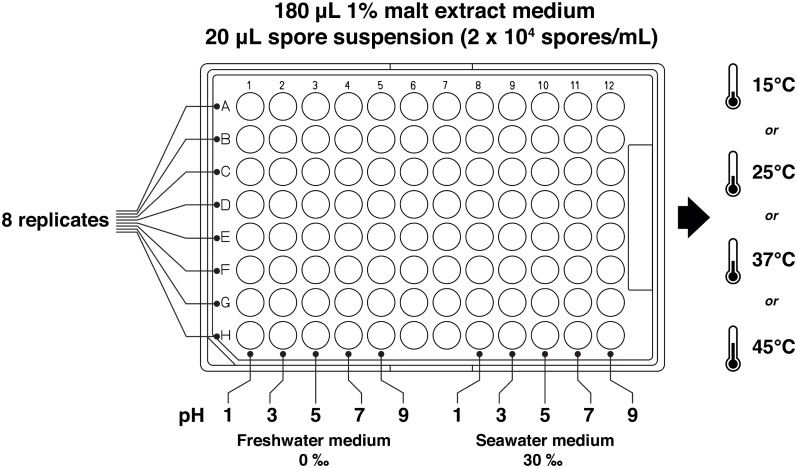
Experimental set-up. Combined effects of temperature (15 °C, 25 °C, 37 °C, 45 °C), salinity (0 ‰, 30 ‰) and pH (1, 3, 5, 7, 9) on mycelial growth of fungi were studied using a microtitre plate method.

A modified protocol for mycelial growth measurement of fungi using a microtiter plate was used [13]. The microtiter plate with the brand and the product number Costar 3595 (Corning, Maine, USA) was used. Malt extract (1%, Bacto^™^, Sparks, USA) was used as the growth medium, and 180 μl of this medium adjusted to various pH (1, 3, 5, 7, 9) and salinities (0 ‰, 30 ‰, made by sea salt) were dispensed into wells of the microtiter plate. Acidity/alkalinity of the medium was adjusted with sulfuric acid (Taiwan Green Version Technology Ltd., New Taipei City, Taiwan) and sodium hydroxide (Panrea, Barcelona, Spain). No precipitation was formed in the 1% malt extract the medium for the five different pHs. The spore suspensions (20 μl) were added to the wells and mixed briefly. The inoculated plates were incubated at 15 °C, 25 °C, 37 °C and 45 °C. For each treatment, 8 replicates (wells) were prepared. For each fungus, a total of 320 wells (2 salinities, 5 pHs, 4 temperatures, 8 replicates) were inoculated. A set of control wells for each treatment, i.e. without addition of spores, was also prepared. The pictorial set-up is shown in Fig 1. The absorbance of the wells of the microtiter plates was measured daily at 630 nm by the multi-detection microplate readers (Synergy HT, BioTek) for 1 month. Growth of the fungi was represented by subtraction of the absorbance of the inoculated wells to that of the control wells.

For growth curve fitting, the sigmoidal function, Richards II, was used to describe growth curves of the fungi. The modelling was performed using program Origin 8.1 (OriginLab Corporation, USA). This function is a generalized logistic model and has the formula:

$$y={a[1+(d-1)e^{-k(x-xc)}]}^{1/(1-d)},d\neq 1$$ (1)

where, *y* represents the observed optical density at time *x*; a is the upper asymptote; *xc* is the point of inflection on the x axis; *k*, growth rate constant; *d*, shape parameter; *e*, is the base of the natural logarithm;

The average normalized growth rate is:

$$\frac{k}{2(d+1)}$$ (2)

Statistical analyses were conducted separately on the growth rate data of isolates cultured in freshwater and seawater media. Normality of the data was tested with Shapiro-Wilks test and homogeneity of variances using Brown-Forsythe. Nearly all data were found to be non-normally distributed with heteroscedastic residuals. Aligned rank transformations (ART) were performed using the ARTool program (http://depts.washington.edu/madlab/proj/art/index.html) [14]. This procedure analyses data from a factorial treatment structure using ranks rather than raw data, alleviating non-normality problem and allowing tests of interactive effects.

A two-way analysis of variance (ANOVA) was applied to the aligned rank transformed growth rate data to determine differences among pH and temperature treatments, as well as their interactions. When there was an interaction among the two factors, the effects of pH and temperature were analysed separately at each level of the other factors by non-parametric one-way ANOVA, followed by multiple comparison Dunn’s test. For this test, SigmaStat 4.0 (Systat Software, Inc., Germany) was used.

### Transcriptomic response of *Aspergillus terreus*

β-tubulin gene of *Aspergillus terreus* NTOU4989 was amplified by PCR and sequenced by the primers bt2a and bt2b [15] and a nucleotide BLAST search was run to confirm the identity of the isolate as *A*. *terreus* (GenBank accession number: MT241263). *Aspergillus terreus* NTOU4989 was subcultured on a CMAS plate (17 g/L cornmeal agar, HiMedia, Mumbai, India; 1 L natural seawater) for 2 weeks where aerial spores were formed abundantly on the medium. Three milliliters of Tween 20 (0.1%) were added on top of the fungal colony, gently shaken to dislodge the spores and transferred to a universal bottle. Spore concentration of this spore suspension was counted using a haemocytometer. Two growth conditions were tested in 30 ‰ salinity: (1) 25 °C and pH 7, and (2) 45 °C and pH 3. The spore suspension was inoculated into 100 ml MESB (1% malt extract; 30 g sea salt; 1 L distilled water) adjusted to pH 3 and pH 7 (with sulfuric acid) in duplicate conical flasks where the final spore concentration was 2×10^3^ spores/ml. The two flasks with the medium adjusted to pH 3 were incubated at 45 °C while those adjusted to pH 7 were incubated at 25 °C, for 10 days (end of log phase in both conditions, S1 Fig). Total RNA was extracted using EasyPure Total RNA Mini Kit (Bioman Scientific Co., Ltd.) according to manufacturer’s instructions. The extracted RNA was sent to Genomics BioSci & Tech (New Taipei City, Taiwan) for RNA quality control and sequencing.

The extracted total RNA was digested with DNaseI; RNA concentration was estimated by Agilent 2100 Bioanalyzer and RNA purity was analyzed using NanoDrop (OD 260/280). For library construction, eukaryotic mRNA was enriched with Oligo(dT) beads, fragmented by fragmentation buffer and reverse transcribed with 6-base random hexamers. Quality control of the library was analyzed by Agilent 2100 Bioanalyzer and ABI StepOnePlus Real-Time PCR System before Illumina HiSeq sequencing.

Base calling of the signal intensity from the Illumina sequencing was analyzed by CASAVA v1.8. Quality of the identification of the nucleobases was reflected by the Phred score. Raw reads with adaptor sequences, reads with the unknown base N > 5% and other low quality reads (nucleotides with Phred < 10 constituting 20% or more of the total length of reads) were removed and the cleaned reads were outputted as a FASTQ file. HISAT v2.0.4 (hierarchical indexing for spliced alignment of transcripts) was used for aligning reads with the reference genome *Aspergillus terreus* NIH2624 (GenBank accession numbers AAJN01000001-AAJN01000268) [16]. StringTie v1.2.1 assembler (parameters: -f = 0.3, -j = 3, -c = 5, -g = 100, -s = 10000, -p = 8) was used to transform RNA-Seq alignments into potential transcripts [17]. Using Cufflinks v2.2.1 [18], Cuffmerge was used to merge together assemblies; Cuffcompare was used to filter a number of artifactual transfrags, merge novel isoforms and known isoforms and maximize overall assembly quality. Bowtie 2 v2.2.6 (input option: -q,–phred33, end-to-end mode:–sensitive, alignment option:–dpad 0,–gbar 99999999, scoring option:–mp 1,1,–np 1,–score-min L,0,-0.1, paired-end option: -I 1, -X 1000,–no-mixed,–no-discordant, performance option: -p 1 reporting option: -k 200) was used to align sequencing reads to the reference genome [19] and RSEM v1.2.12 was used to quantify gene and isoform expression (minAS = 0.66) [20]. FPKM (fragments per kilobase of exon model per million reads mapped) was the algorithm adopted to normalize estimation of gene expression [21]. A cluster analysis was performed to compare gene expression profiles of the four RNA samples [22,23]. For differential expressed genes (DEG), DEseq2 (fold change ≥ 2.00 and adjusted P value ≤ 0.05), EBseq (fold change ≥ 2.00 and posterior probability of being equivalent expression (PPEE) ≤ 0.05), NOIseq (Fold Change ≥ 2.00 and probability ≥ 0.8) and PossionDis (fold change ≥ 2.00 and FDR ≤ 0.001) methods were used to determine significantly differentially expressed genes. A hierarchical clustering was performed for the four RNA samples based on FPKM values of gene expression of DEG using the pheatmap function in R [24]. Phyper function in R was used to run gene enrichment analysis (false discovery rate (FDR) ≤ 0.01) for functional classification of the DEG (gene ontology, GO) and for biological pathway classification of the DEG (Kyoto Encyclopedia of Genes and Genomes, KEGG) [24]. Raw RNA sequences are available at Sequence Read Archive (SRA) under the number PRJNA561236.

## Results

### Combined effects of pH, temperature and salinity on growth

**Fig 2:**
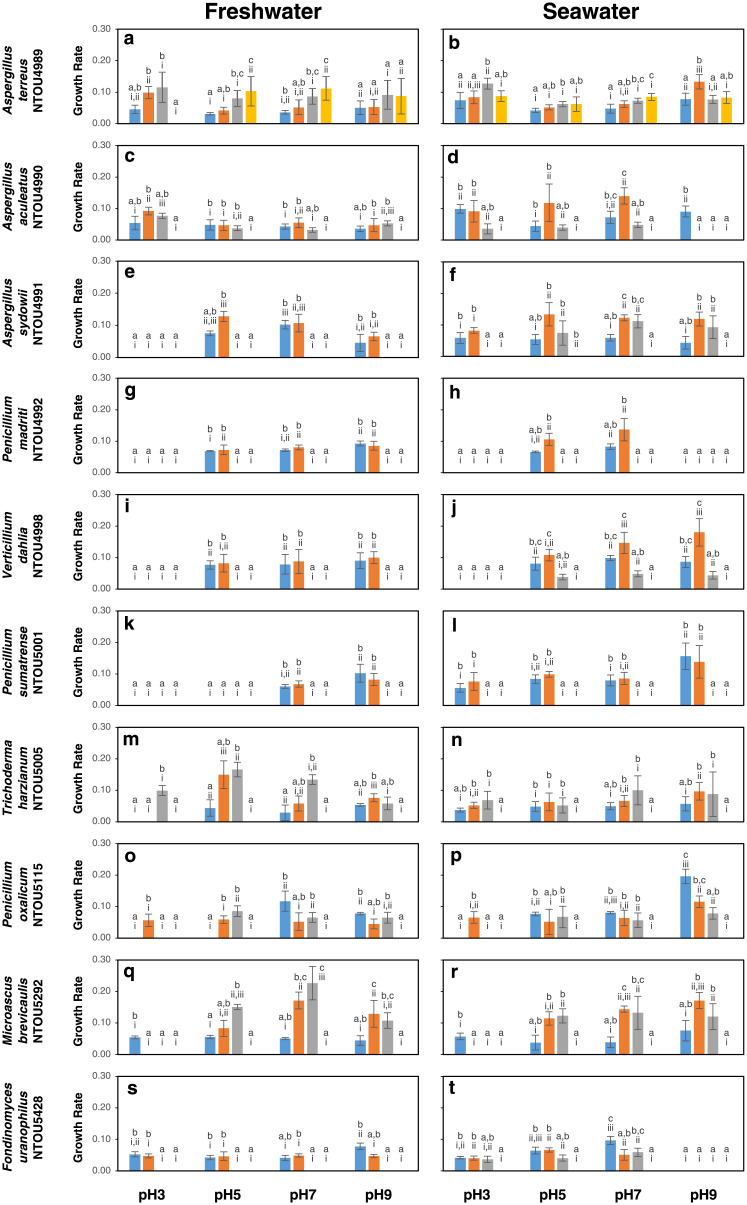
Normalized averaged growth rates of the tested fungal species cultured in/at 2 salinities (0‰ and 30‰), 4 pHs (pH 3, pH 5, pH 7 and pH 9) and 4 temperatures (15 °C, blue bar; 25 °C, orange bar; 37 °C, grey bar and 45 °C, yellow bar) for 30 days. Data are presented as means ± S.D. Different letters indicate significant differences between temperatures (*p* < 0.05) within the same pH treatments and different Roman numerals indicate significant differences between pH within the same temperature treatments (*p* < 0.05).

In this study, the primary model given by Eq 1 was fitted to the experimental growth data obtained from the ten fungal species cultured in/at 2 different salinities (0 ‰ and 30 ‰), 4 different pHs (3, 5, 7 and 9) and 4 temperatures (15 °C, 25 °C, 37 °C and 45 °C). The growth curves of the fungi are shown in S1 Fig. From these growth curves, the normalized average growth rate of the fungi under the different culture conditions was estimated and is presented in Fig 2. In general, growth rate of all fungi, either cultured in the freshwater (0 ‰) or seawater (30 ‰) media, was dependent on pH and temperature. There was a statistically significant interaction between the effects of pH and temperature on growth rate. No growth was observed in the medium adjusted to pH 1 for all fungi. In general, growth of most fungi reached a plateau after 14 days of incubation and an increase in temperature delayed the time reaching the stationary phase.

Growth response of the ten fungi can be grouped into three categories based on their growth ranges under the tested temperatures, pHs and salinities: (1) wide pH, salinity and temperature ranges (*Aspergillus aculeatus* NTOU4990, *Trichoderma harzianum* NTOU5005, *Penicillium oxalicum* NTOU5115, *Microascus brevicaulis* NTOU5292) with the ability to grow at 3 temperatures (15 °C, 25 °C, 37 °C), 4 pHs (3, 5, 7, 9) and 2 salinities (0 ‰, 30 ‰), (2) salinity-dependent and temperature-sensitive (*Aspergillus sydowii* NTOU4991, *Penicillium madriti* NTOU4992, *Verticillium dahlia* NTOU4998, *Penicillium sumatrense* NTOU5001, *Fodinomyces uranophilus* NTOU5428) with growth generally occurred at only 2 temperatures (15 °C, 25 °C) and 2–4 pHs (generally >pH 5) and having better growth at 30 ‰ over 0 ‰, and (3) temperature-tolerant (*Aspergillus terreus* NTOU4989) with growth at 45 °C.

*Aspergillus terreus* NTOU4989 was temperature-tolerant; growth was recorded from pH 3 to pH 9 and from 15 °C to 45 °C in the freshwater and seawater media (S1 Fig, Fig 2a and 2b). It was the only fungus that showed growth at 45 °C in this study. No growth was observed when this fungus was incubated at 50 °C (results not shown). In the freshwater medium, better growth (*p*<0.05) was observed at higher incubation temperatures, i.e. 37 °C and 45 °C, except that no growth was observed at pH 3 at 45 °C. Low growth rate was observed at pH 9 for all salinities and temperatures. In the seawater medium, *A*. *terreus* grew better at temperatures above 15 °C, independent of the pH. The optimal growth conditions were pH 3, 37 °C and 30 ‰ salinity.

Growth of five fungi was salinity-dependent and temperature-sensitive. *Aspergillus sydowii* NTOU4991 grew better in 30 ‰ than 0 ‰ salinity in general (S1 Fig, Fig 2e and 2f). In 0 ‰ salinity, no growth was observed in pH 3 and incubation temperatures above 25 °C. There was no significant difference in growth rate at 15 °C and 25 °C, but better growth (*p*<0.05) was found in pH 5 and pH 7. The fungus showed better growth (*p*<0.05) in pH above 5 at 25°C and 37 °C in 30 ‰ salinity. For *Penicillium madriti* NTOU4992, no growth was observed in pH 3 in 0 ‰ salinity, as well as temperatures above 25 °C (S1 Fig, Fig 2g and 2h). No significant difference was found in growth in pH 5–pH 9 at 15 °C and 25 °C. In 30 ‰ salinity, *P*. *madriti* NTOU4992 only grew in pH 5 and pH 7 at 15 °C and 25 °C, with a better growth (*p*<0.05) at 25 °C. The growth response of *Verticillium dahlia* NTOU4998 was similar to *P*. *madriti* NTOU4992, except that growth was observed at 37 °C in pH 5–pH 9 and at 15 °C and 25 °C in pH 9 in 30 ‰ salinity (S1 Fig, Fig 2i and 2j). At 25 °C, growth increased with pH, with the optimal growth in pH 9. For *Penicillium sumatrense* NTOU5001, growth occurred only at 15 °C and 25 °C in both salinities and in pH 7 and pH 9 in 30 ‰ salinity, with no significant differences in their growth rates (S1 Fig, Fig 2k and 2l). Better growth (*p*<0.05) was observed in pH 9.

The last group of fungi showed wide pH, salinity and temperature ranges. For *Aspergillus aculeatus* NTOU4990, either cultured in 0 ‰ or 30 ‰ salinity, no growth was observed at 45 °C (S1 Fig, Fig 2c and 2d). In 0 ‰ salinity, growth was comparable in general in all tested temperatures and pHs although the optimal growth conditions were pH 3, 25°C and 37 °C. In 30 ‰ salinity, no growth was observed in pH 9 at temperatures above 15 °C. In pH 3 and pH 5, the optimal growth temperatures were 15 °C and 25 °C. The maximum growth rate was found in pH 7 at 37 °C. For *Trichoderma harzianum* NTOU5005, no growth was observed at 45 °C (S1 Fig, Fig 2m and 2n). In pH 3 and 0 ‰ salinity, growth occurred only at 37 °C. An increase in growth rate with increasing temperature was observed in pH 5 and pH 7, but not in pH 9. This trend was also noticed in 30 ‰ salinity, but the growth rates were similar among all tested pHs. In 0‰ salinity, the growth rates of *Penicillium oxalicum* NTOU5115 were similar, and the responses to temperature changes were slightly different in different pHs (S1 Fig, Fig 2o and 2p), i.e., growth only found at 25 °C in pH 3, and 25 °C and 37 °C in pH 5. For pH 7 and pH 9, growth was observed from 15 °C to 37 °C. In 30‰ salinity, growth rates were generally higher in pH 9 with a decreasing trend with increasing temperatures. Growth was only found in pH 3 at 25 °C. Growth was not observed at 45 °C. A similar growth pattern was resulted in both 0 ‰ and 30 ‰ salinities for *Microascus brevicaulis* NTOU5292 (S1 Fig, Fig 2q and 2r). In pH 3, no growth was observed at temperatures higher than 15 °C. Within each tested pH, there was a slightly increasing trend in growth rate when the incubation temperature increased from 15 °C to 37 °C. No significant differences were discovered in different pHs at the same incubation temperature. No growth was observed at 45 °C. *Fodinomyces uranophilus* NTOU5428 could grow in a wider range of pH in 0 ‰ salinity than 30 ‰ salinity, but restricted to 15 °C and 25 °C (S1 Fig, Fig 2s and 2t). In contrast, *F*. *uranophilus* NTOU5428 could only grow from pH 3 up to pH 7, but growth was observed at 37 °C in 30 ‰ salinity.

### Transcriptomic response of *Aspergillus terreus*

For the transcriptome analysis of *Aspergillus terreus* NTOU4989 at 25 °C-pH 7 and at 45 °C-pH 3, > 6 giga clean bases and > 40 million reads were obtained from the samples (S1 Table); a Phred score of Q20 (base call accuracy 99%) for > 97% of the bases sequenced. The total mapped reads of the samples were >88% including 18.93–23.83% spice mapped and 65.38–70.66% non-splice mapped reads (S2 Table). A total of 4471 novel transcripts (new spliced types of a known gene or new transcripts) were obtained, with 3656 coding transcripts after assembly (S3 Table). After alignment with the reference genome, total gene number for the samples ranged from 8570 to 8830 with 55–57 novel genes (predicted coding transcripts that were previously unknown after comparison with the reference genome; S4 Table). The Venn diagram shown in S2 Fig shows the number of unique and common genes in/between the samples; 7945 genes were expressed in all samples and more unique genes were expressed in the 25 °C-pH 7 samples (118–122) than in the 45 °C-pH 3 samples (81–83). The correlation between samples based on differences in gene expression is shown in S3 Fig. The coefficients (S3a Fig), the cluster analysis (S3b Fig) and the hierarchical clustering of genes based on FPKM values (S3c Fig) on the four samples showed the gene expression between the replicate samples of each growth condition was highly similar but between the two growth conditions was very different.

A comparison of differential gene expression between the samples is shown in S4 Fig. The differential expressed genes between the replicates of each growth condition were low (46–91 up-regulated and 113–121 down-regulated genes), and those between growth conditions were high (1015 and 943 genes up- and down-regulated, respectively).

**Fig 3:**
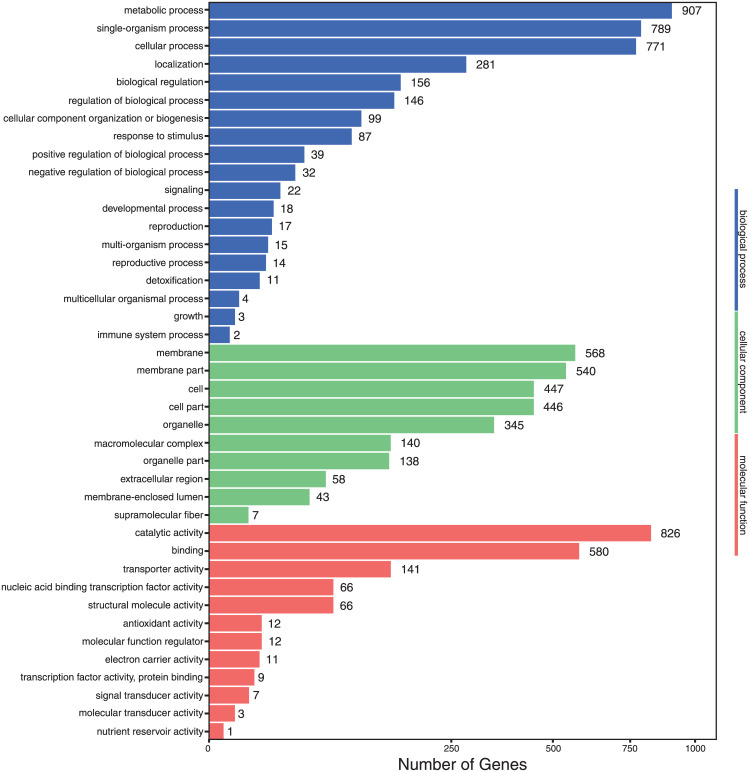
Gene function analysis of the differential expressed genes of RNA-seq analysis of *Aspergillus terreus* incubated at 25 °C-pH 7 and at 45 °C-pH 3 based on Gene Ontology (GO). The differential expressed genes could be referred to 19 categories in biological process, 10 categories in cellular component and 12 categories in molecular function.

**Fig 4:**
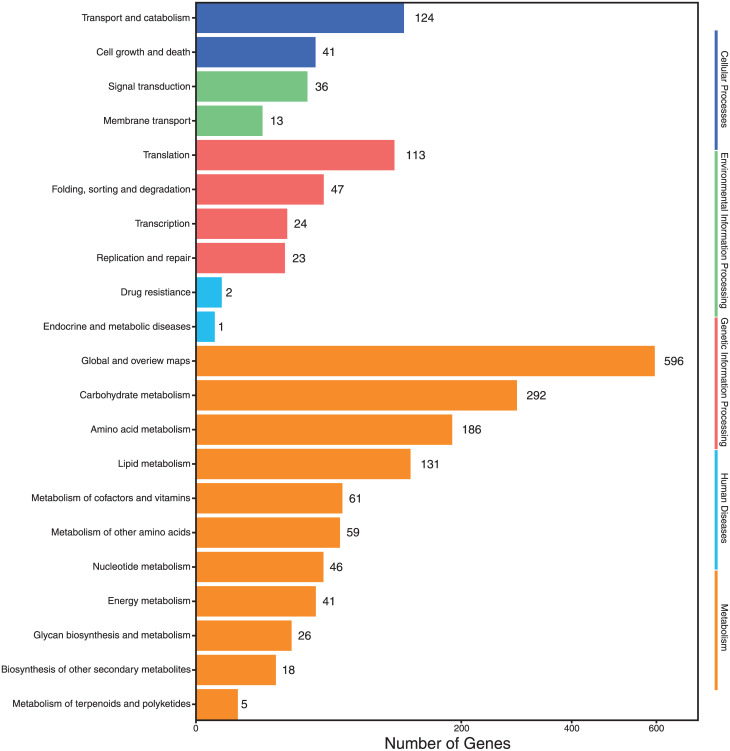
KEGG (Kyoto Encyclopedia of Genes and Genomes) pathway classification of the differential expressed genes of RNA-seq analysis of *Aspergillus terreus* incubated at 25 °C-pH 7 and at 45 °C-pH 3. The differential expressed genes could be referred to 21 groups (2 in cellular processes, 2 in environmental information, 4 in genetic information processing, 2 in human diseases, 11 in metabolism).

Abundance of GO (Gene Ontology) and KEGG (Kyoto Encyclopedia of Genes and Genomes) function categories of the differential expressed genes (DEG) between the two growth conditions are shown in Figs 3 and 4, respectively. In GO functional classification (Fig 3), the DEG of the two growth conditions could be referred to 19 categories in biological process, 10 categories in cellular component, 12 categories in molecular function. The dominant DEG annotations in biological process were metabolic process, single-organism process and cellular process, those in cellular component were membrane, membrane parts, cell, cell parts and organelle, and those in molecular function were catalytic activity and binding.

In KEGG pathway classification (Fig 4), the DEG of the two growth conditions could be referred to 21 groups (2 in cellular processes, 2 in environmental information, 4 in genetic information processing, 2 in human diseases, 11 in metabolism). The dominant enriched pathways (>100 DEG), in decreasing order, were global and overview maps, carbohydrate metabolism, amino acid metabolism, lipid metabolism, transport and catabolism and translation.

**Fig 5:**
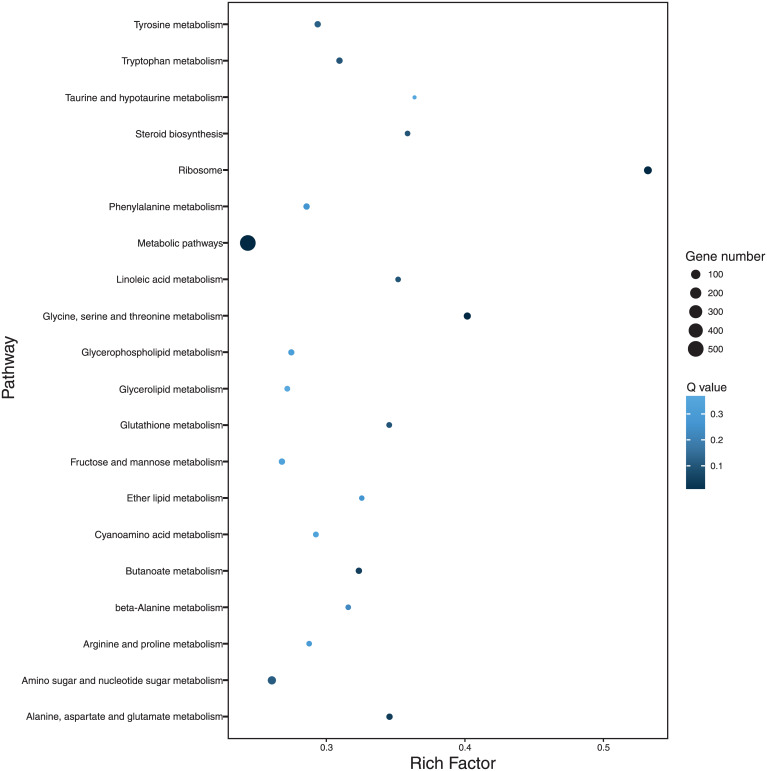
Kyoto Encyclopedia of Genes and Genomes (KEGG) enrichment scatter plot. Twenty most enriched differential expressed genes (DEGs) of RNA-seq analysis of *Aspergillus terreus* incubated at 25 °C and pH 7 and at 45 °C and pH 3; y-axis = name of pathway, x-axis = rich factor, dot size = number of different genes, color = q-value.

The 20 most enriched KEGG pathways of DEG between the two growth conditions are shown in Fig 5. These pathways of DEG are related to amino acid metabolism, steroid biosynthesis, ribosome, metabolic pathways, fatty acid metabolism, antioxidant metabolism and carbohydrate metabolism.

S5 Table lists the up- and down-regulated DEGs (45 °C-pH 3/25 °C-pH 7). A total of 1435 DEGs (out of 1958, ~73%) was found to be hypothetical proteins, while the rest could be referred to a GO function. There was a core group of genes expressed by *A*. *terreus* in both growth conditions (45 °C-pH 3 and 25 °C-pH 7) related to normal cell metabolism, including RNA polymerase II transcription factor activity, DNA/RNA binding, zinc ion binding, catalytic activity, ATP binding, amino acid transmembrane transporter activity, oxidoreductase activity, transferase activity, hydrolase activity, iron ion binding, mitochondrial metabolism, carbohydrate metabolism among others. A number of DEGs related to various stress responses of fungi were observed in the transcriptome of *A*. *terreus* NTOU4989 and are summarized in Table 1.

**Table 1:** Differential expressed genes in transcriptome of *Aspergillus terreus* NTOU4989 grown at 45 °C-pH 3 and 25 °C-pH 7 related to potential stress response of fungi. Proteins related to pH, oxidative, hypoxic, temperature, osmotic, carbon-depletion, cell wall and nitrosative stress responses in fungi.

Protein	Possible stress response (predicted response)	Up-regulated Gene ID (log2FoldChange (45 °C-pH 3/ 25 °C-pH 7))	Down-regulated Gene ID (log2FoldChange (45 °C-pH 3/ 25 °C-pH 7))	Reference
Acetyl xylan esterase	acid, oxidative (down-regulation)	4354490 (4.00)	—	[41, 42]
Aflatoxin	oxidative (up-regulation)	4319148 (3.76), 4316834 (2.06), 4320858 (1.60), 4322721 (1.40)	4354671 (-3.82), 4322332 (-3.64), 4323497 (-2.71), 4354697 (-2.01), 4319475 (-1.64), 4355706 (-1.39), 4319293 (-1.19)	[54]
Alcohol dehydrogenase	hypoxic, temperature (up-regulation)	4321194 (5.25), 4353690 (3.79), 4317125 (1.93), 4320311 (1.49), 4321883 (1.36), 4317432 (1.28)	4319354 (-2.50), 4322652 (-2.01), 4322136 (-1.13)	[53]
Amino acid oxidase	temperature (up-regulation)	—	4316479 (-1.79)	[59]
Arginine metabolism	osmotic (up-regulation)	4319103 (3.88), 4354418 (3.78), 4322366 (3.55), 4321618 (3.52), 4353077 (3.41), 4321586 (3.34), 4321384 (2.56), 4316050 (2.39), 4320734 (2.14), 4321676 (2.11), 4354830 (1.80), 4321391 (1.77), 4355438 (1.76), 4355125 (1.71), 4315800 (1.63), 4354417 (1.62), 4319112 (1.61), 4353388 (1.52), 4322328 (1.49), 4320098 (1.36), 4315839 (1.20), 4353079 (1.19), 4318840 (1.12), 4315748 (1.05), 4315591 (1.04), 4321722 (1.03)	4321852 (-3.05), 4321275 (-1.91), 4316479 (-1.79), 4354282 (-1.56)	[43]
Aspartic-type endopeptidase	osmotic (up-regulation)	4321672 (3.53), 4323187 (1.20)	4321997 (-1.50)	[45]
Aspergillopepsin	oxidative (down-regulation)	4321672 (3.53)	—	[41]
ATP-binding cassette transporters (ABC)	acid (up-regulation)	4316513 (7.27), 4353093 (5.03), 4318937 (4.19), 4318279 (2.38), 4354610 (1.48), 4320477 (1.18)	4322542 (-5.35), 4323000 (-2.54), 4323154 (-1.93), 4319221 (-1.89), 4322078 (-1.86), 4354819 (-1.48), 4315724 (-1.15)	[38]
Beta-glucosidase	carbon-depletion (up-regulation)	4317260 (5.06), 4318123 (2.99), 4320812 (1.26), 4353962 (1.23), 4318025 (1.18)	4354491 (-2.84)	[52]
Catalase	oxidative (up-regulation)	4315896 (3.14)	4355555 (-4.40), 4322573 (-3.39)	[37, 60]
Chitinase	carbon-depletion (up-regulation), oxidative (down-regulation)	—	4317444 (3.64), 4320222 (-1.33), 4323544 (-1.30), 4316602 (-1.21)	[52, 54]
Chitosanase	oxidative (down-regulation)	—	4315966 (-6.32)	[54]
Choline sulfatase	osmotic (up-regulation)	4355225 (1.35)	—	[45]
Copper amine oxidase	oxidative (up-regulation)	4353565 (1.48)	4317980 (-1.16)	[39]
Ergosterol	osmotic, oxidative (up-regulation)	—	4315670 (-4.61)	[57]
Gamma-aminobutyric acid	acid (up-regulation)	4317602 (1.43), 4321181 (1.14), 4315919 (1.03)	—	[39]
Gliotoxin biosynthesis	oxidative (hydrogen peroxide-induced) (up-regulation)	4353298 (3.08)	4320660 (-1.94)	[61]
Glutamate decarboxylase	osmotic, oxidative (up-regulation)	—	4322664 (-4.66)	[39, 50]
Glutamine	cell wall stress (up-regulation)	4320749 (8.15), 4319103 (3.88), 4321586 (3.34), 4321384 (2.56), 4355438 (1.76), 4354352 (1.55), 4353399 (1.55), 4353081 (1.30), 4322780 (1.18), 4353370 (1.17), 4315591 (1.04), 4319291 (1.02)	4320031 (-6.50), 4321275 (-1.91)	[62]
Glutathione	oxidative (up-regulation)	4322268 (5.93), 4321194 (5.25), 4321195 (4.87), 4322350 (3.57), 4353112 (3.20), 4353104 (2.44), 4319118 (2.33), 4320301 (2.15), 4354830 (1.80), 4322827 (1.50), 4318180 (1.16), 4319614 (1.15), 4355704 (1.12)	4317199 (-3.78), 4323475 (-3.63), 4355663 (-3.07), 4317813 (-2.93), 4322391 (-2.55), 4318595 (-1.92), 4315603 (-1.89), 4319310 (-1.26), 4320897 (-1.21), 4320597 (-1.09), 4322689 (-1.04)	[54]
Glutathione S-transferase	oxidative, temperature (up-regulation)	—	4317813 (-2.93), 4319310 (-1.26), 4320897 (-1.21)	[48, 63]
Glycerolipid metabolism	osmotic (up-regulation)	4321046 (5.46), 4322616 (3.96), 4354944 (3.30), 4323185 (3.16), 4353360 (2.20), 4354635 (2.10), 4317867 (1.74), 4320290 (1.60), 4316651 (1.53), 4353388 (1.52), 4316296 (1.51), 4318243 (1.51), 4320098 (1.36), 4319925 (1.22), 4316619 (1.12), 4355674 (1.03)	4354928 (-4.42), 4354297 (-3.85), 4319597 (3.62), 4355310 (-2.66), 4323438 (-2.52), 4321483 (-1.96), 4316410 (-1.65), 4320789 (-1.53), 4321468 (-1.27), 4321158 (-1.26)	[50]
Heat shock proteins	pH, temperature (up-regulation)	4354296 (1.64)	4355072 (-1.66), 4319660 (-1.27), 4355586 (-1.22), 4353464 (-1.01)	[35, 57]
Hexosaminidase	oxidative (down-regulation)	4354880 (1.07)	—	[41]
Histidine kinase	oxidative, temperature (up-regulation)	4354131 (1.37)	—	[57]
Hydrogen ion transmembrane transport	acid (up-regulation)	4316676 (7.04), 4316796 (2.76), 4316399 (2.26), 4319629 (2.21), 4320653 (1.59)	4353371 (-1.08)	This study
Linoleic acid	salinity (up-regulation)	4321046 (5.46), 4353690 (3.79), 4323320 (3.31), 4316197 (2.22), 4355287 (1.79), 4322309 (1.62), 4321171 (1.36), 4316757 (1.25)	4322333 (-3.73), 4319122 (-3.43), 4320159 (-3.40), 4317455 (-2.53), 4354505 (-2.26), 4319076 (-1.94), 4317482 (-1.93), 4321264 (-1.70), 4353959 (-1.45), 4317752 (-1.41), 4353494 (-1.04)	[43]
Mannitol biosynthesis	oxidative, temperature (up-regulation)	4315529 (1.13), 4354927 (1.44)	—	[64]
Mitogen-activated protein kinase (MAPK)	cell wall stress, oxidative (up-regulation)	4320728 (8.20), 4315896 (3.14), 4317554 (2.39), 4323135 (2.35), 4319134 (1.98), 4318064 (1.97), 4355602 (1.97), 4321870 (1.52), 4315701 (1.25), 4322910 (1.00)	4321050 (-4.79), 4317063 (-4.76), 4355555 (-4.40), 4322342 (-3.64), 4322804 (-3.59), 4322573 (-3.39), 4322564 (-3.38), 4316130 (-3.31), 4322243 (-3.31), 4316279 (-3.14), 4321013 (-2.88), 4354085 (-2.69), 4322424 (-2.47), 4320166 (-2.37), 4316375 (-2.34), 4354865 (-1.87), 4355651 (-1.80), 4319314 (-1.41), 4354530 (-1.32), 4318402 (-1.25), 4315579 (-1.16)	[49, 55, 57, 65]
Monooxygenase	oxidative (up-regulation)	—	4322890 (-3.05), 4315604 (-2.67), 4355310 (-2.66), 4320632 (-2.55), 4321773 (-2.43), 4318673 (-2.10), 4320375 (-2.09), 4318481 (-2.04), 4318365 (-1.62), 4322598 (-1.53), 4322596 (-1.44), 4321797 (-1.39), 4318709 (-1.33), 4321412 (-1.10), 4323015 (-6.47), 4319006 (-4.14), 4319545 (-4.08), 4317485 (-3.66), 4318940 (-3.66)	[59]
NAD(P)-binding domain-containing proteins	temperature (down-regulation)	4321032 (10.51), 4315574 (7.91), 4322619 (7.15), 4355513 (6.58), 4321194 (5.25), 4321780 (4.95), 4354776 (3.61), 4318976 (3.27), 4354421 (2.68), 4319414 (2.60), 4317090 (2.44), 4321063 (2.36), 4317050 (2.27), 4316197 (2.22), 4316270 (2.19), 4315523 (2.15), 4317599 (2.10), 4317125 (1.93), 4316273 (1.88), 4354830 (1.80), 4355287 (1.79), 4354271 (1.70), 4322822 (1.66), 4354417 (1.62), 4315740 (1.59), 4353399 (1.55), 4354903 (1.54), 4318165 (1.52), 4353388 (1.52), 4355113 (1.52), 4318243 (1.51), 4320311 (1.49), 4322328 (1.49), 4320098 (1.36), 4319639 (1.34), 4317103 (1.32), 4323459 (1.29), 4319993 (1.23), 4320546 (1.22), 4321181 (1.14), 4318618 (1.14), 4353403 (1.10), 4320195 (1.05), 4353673 (1.05), 4355335 (1.05), 4355414 (1.04), 4318193 (1.00)	4317257 (-5.55), 4320192 (-3.68), 4354068 (-3.30), 4354821 (-3.00), 4322586 (-2.99), 4316169 (-2.97), 4319658 (-2.79), 4322652 (-2.01), 4321656 (-1.67), 4317534 (-1.56), 4317823 (-1.49), 4319020 (-1.36), 4319611 (-1.29), 4316247 (-1.17)	[59]
Peroxidase	temperature (down-regulation)	—	4316488 (-1.69), 4355239 (-1.18), 4355094 (-2.31)	[66]
Peroxiredoxin	temperature (up-regulation)	4354964 (2.18)	4318739 (-1.36)	[67]
Phenylalanine ammonia lyase (PAL)	acid (up-regulation)	4353823 (1.77)	—	[38]
pH-response transcription factor (PacC)	acid (down-regulation)	—	4320166 (-2.37)	[39, 40]
Proteins of nitrosative stress	nitrosative stress (up-regulation)	4317090 (2.44)	—	[55]
Pyrroline-5-carboxylate dehydrogenase	osmotic (up-regulation)	4354417 (1.62)	—	[50]
Pyruvate decarboxylase	hypoxic (up-regulation)	4318879 (2.05)	—	[53]
Structural constituent of ribosome	unknown	4317446 (8.50), 4320221 (2.03), 4323138 (1.83), 4318679 (1.71), 4318230 (1.66), 4320928 (1.65), 4321617 (1.62), 4317434 (1.61), 4354992 (1.61), 4317815 (1.60), 4320943 (1.60), 4315560 (1.56), 4318617 (1.55), 4317384 (1.54), 4316233 (1.53), 4317038 (1.53), 4316312 (1.52), 4323484 (1.51), 4318410 (1.48), 4353751 (1.46), 4354157 (1.44), 4319586 (1.39), 4355396 (1.38), 4319384 (1.32), 4317339 (1.28), 4320447 (1.28), 4323260 (1.27), 4323576 (1.25), 4355066 (1.25), 4317627 (1.22), 4316669 (1.21), 4317284 (1.19), 4318457 (1.19), 4320422 (1.18), 4318265 (1.15), 4353661 (1.15), 4321075 (1.13), 4318065 (1.03), 4354007 (1.02), 4355235 (1.67), 4354329 (1.05), 4318045 (1.35), 4323302 (1.76), 4323457 (1.40), 4323259 (1.38), 4354109 (1.22), 4354397 (1.50), 4316141 (2.05), 4316736 (1.10), 4322138 (1.45), 4323287 (1.18)	—	This study
Superoxide dismutase	oxidative (up-regulation)	4322186 (1.17)	—	[37]
Thioredoxin	oxidative (up-regulation)	—	4318595 (-1.92), 4323550 (-1.09), 4320597 (-1.09)	[54, 60]
Ubiquitin	oxidative (up-regulation)	4321110 (2.61), 4320221 (2.03), 4316267 (1.59), 4318004 (1.52), 4315747 (1.11)	4320192 (-3.68), 4320450 (-3.56), 4315954 (-2.78), 4320609 (-2.25), 4319633 (-2.23), 4319351 (-1.57), 4321997 (-1.50), 4354867 (-1.41), 4321444 (-1.31), 4355664 (-1.22), 4318281 (-1.08)	[68]

For genes related to temperature response, mannitol biosynthetic genes were up-regulated at 45 °C-pH 3. Heat-shock protein genes were mostly down-regulated (log2 Fold Change (45 °C-pH 3/ 25 °C-pH 7) [Log2FC] = -1.01 to -1.66) while only one heat shock transcription factor gene was up-regulated (Log2FC = 1.64). Other down-regulated genes included glutathione S-transferase (Log2FC = -1.21 to -2.93) and amino acid oxidase (Log2FC = -1.79). Other possible temperature-related stress genes reported to be differentially expressed were both up-regulated and down-regulated, including gliotoxin biosynthesis (Log2FC: up-regulation = 3.08, down-regulation = -1.94), peroxiredoxin (Log2FC: up-regulation = 2.18, down-regulation = -1.36), alcohol dehydrogenase (Log2FC: up-regulation = 1.28 to 5.25, down-regulation = -1.13 to -2.50) and NAD(P)-binding domain-containing proteins (Log2FC: up-regulation = 1.00 to 10.51, down-regulation = -1.17 to -5.55).

Several DEGs could be referred to pH response. ATP-binding cassette transporters (ABC) (Log2FC: up-regulation = 1.18 to 7.27, down-regulation = -1.56 to -3.05) and hydrogen ion transmembrane transport (Log2FC: up-regulation = 1.59 to 7.04, down-regulation = -1.08) were both up-regulated and down-regulated although proteins of the hydrogen ion transmembrane transport were mostly up-regulated. Phenylalanine ammonia lyase (PAL) (Log2FC = 1.77) was up-regulated while pH-response transcription factor (PacC) was down-regulated (Log2FC = -2.37).

Up-regulated genes related to various oxidative stress responses include superoxide dismutase (Log2FC = 1.17), histidine kinase (Log2FC = 1.37), acetyl xylan esterase (Log2FC = 4.00), aspergillopepsin (Log2FC = 3.53) and hexosaminidase (Log2FC = 1.07) while thioredoxin (Log2FC = -1.09 to -1.92), ergosterol (Log2FC = -4.61), chitosanase (Log2FC = -6.32) and monooxygenase (Log2FC = -1.10 to -6.47) were down-regulated. Other genes related to oxidative stress were both up- and down-regulated, i.e. catalase (Log2FC: up-regulation = 3.14, down-regulation = -3.39 to -3.40), ubiquitin (Log2FC: up-regulation = 1.11 to 2.61, down-regulation = -1.08 to -3.68), mitogen-activated protein kinase (MAPK) (Log2FC: up-regulation = 1.00 to 8.20, down-regulation = -1.16 to -4.79), glutathione (Log2FC: up-regulation = 1.12 to 5.93, down-regulation = -1.04 to -3.78) and aflatoxin biosynthesis (Log2FC: up-regulation = 1.40 to 3.76, down-regulation = -1.19 to -3.82). One unknown gene related to nitrosative stress was up-regulated (Log2FC = 2.44).

For osmotic response, genes of glycerolipid metabolism (Log2FC: up-regulation = 1.03 to 5.46, down-regulation = -1.26 to -4.42), linoleic acid (Log2FC: up-regulation = 1.25 to 5.46, down-regulation = -1.04 to -3.73) and MAPK were both up-regulated and down-regulated. One pyrroline-5-carboxylate dehydrogenase gene was up-regulated (Log2FC = 1.62) while one glutamate decarboxylase gene was down-regulated (Log2FC = -4.66).

Other genes related to various stress types were also noticed. Hypoxic stress response genes were differentially expressed; pyruvate decarboxylase was up-regulated (Log2FC = 2.05) while alcohol dehydrogenase was both up- (Log2FC = 1.28 to 5.25) and down-regulated (Log2FC = -1.13 to -2.50). For stress genes related to carbon depletion, four chitinase genes were down-regulated (Log2FC = -1.21 to -3.64). Beta-glucosidase genes were both up- (Log2FC = 1.18 to 5.06) and down-regulated (Log2FC = -2.84). Glutamine genes related to cell wall stress were mainly up-regulated (12 genes, Log2FC = 1.02 to 8.15) but also down-regulated (2 genes, Log2FC = -1.91 to -6.50). Many genes related to structural constituent of ribosome were only up-regulated (Log2FC = 1.02 to 8.50).

## Discussion

### Physiological adaptability of fungi

With the increased interests in the study of fungi in marine hydrothermal vent ecosystems in recent years, our understanding on the fungal diversity in these habitats, predominantly in deep-sea vents, has advanced dramatically. However, insufficient evidence is available to suggest if these fungi play a definite role in this environment. Some yeasts, isolated from deep-sea hydrothermal vents, were able to grow under high hydrostatic pressure conditions [25]. Deep-sea *Aspergillus* isolates were also shown to be able to grow and germinate under a high pressure [26]. The effect of hydrostatic pressure on fungal growth at the shallow-water hydrothermal vent field of Kueishan Island (with depths up to 80 m) is not as severe as that in deep-sea hydrothermal vent fields [25]. However, the physical and chemical conditions at both marine shallow-water and deep-sea hydrothermal vent fields can be extreme with the possibility of a low pH and a high temperature. In this study, different growth responses of the selected fungi isolated from the sediment and crab (*Xenograpsus testudinatus*) samples at/near the hydrothermal vents at Kueishan Island, Taiwan [11] under the combined influence of salinity, pH and temperature were observed. Fungi are eukaryotic organisms with a low temperature tolerance limit, when compared to prokaryotes [27]. Temperature is a key factor determining geographical distribution of marine fungi [28] and was found in this study to be an important growth-limiting factor with only half of the taxa tested able to grow at 37 °C or above.

*Aspergillus terreus* NTOU4989, cultured from the yellow sediment (near the vents), was the only fungus that showed growth at 45 °C, pH 3 and 30 ‰ salinity, suggesting a possible adaptation of this fungus near the vents. It is unknown why no growth was observed at 45 °C, pH 3 and 0 ‰ salinity. *Aspergillus* species exhibit wide temperature, salinity and pH growth ranges and can tolerate comparative high temperatures and salinities [29]. In both freshwater and seawater media, the optimal growth conditions for *A*. *terreus* were in acidic environment (pH 3) at 37 °C.

*Aspergillus aculeatus*, *Microascus brevicaulis*, *Penicillium oxalicum* and *Trichoderma harzianum* were regarded as marine fungal species [30] and they displayed wide temperature, salinity and pH growth ranges, implicating that they could adapt to changes in environmental conditions. *Aspergillus sydowii* occurs in both terrestrial and marine environments, and *A*. *sydowii* NTOU4991 is likely a marine isolate with its better growth in the 30 ‰ salinity condition. *Verticillium dahlia* and *Penicillium sumatrense* were able to grow in 0 ‰ and 30 ‰ salinities, showing their salinity tolerance and a possible marine nature [31], but these two species were not regarded as marine species [30]. These results show that seabed (including substrates growing therein) is a sink for both marine and terrestrial (air deposition or freshwater runoff) fungi and cautions must be paid to refer ecological niches of fungi recovered from this habitat. These also confirm the difficulty in defining a fungus marine [31].

*Penicillium madriti* and *Fodinomyces uranophilus* showed a wider pH tolerance range (up to pH 9) in 0 ‰ over in 30 ‰ salinity. Both fungi were not regarded as marine [30] and were probably of a terrestrial origin. Fröhlich-Nowoisky and co-workers [32] found that Dothideomycetes (*F*. *uranophilus*) and Eurotiomycetes (*P*. *madriti*) were two of the dominant classes of fungi in coastal and marine air samples and propagules of these species may eventually settle into the seabed.

### Stress response of *Aspergillus terreus* NTOU4989

*Aspergillus terreus* has a wide geographical distribution in the terrestrial environment and can cause diseases of human [33] but it has also been reported from the marine environment [34]. This is the first study to look into the molecular adaptations of an isolate of *A*. *terreus* (NTOU4989) cultured from a marine shallow-water hydrothermal vent ecosystem to a high temperature and a low pH conditions, possible environmental conditions at hydrothermal vent ecosystems.

The results obtained from the samples in the transcriptome analysis were reliable with over 6 Giga clean bases, over 40 million clean reads, a Phred score of Q20 for > 97% bases and with total mapped percentages of reads of approximately 90%. Among the >8500 genes expressed, 7945 common genes (>90%) were found in all samples, suggesting these genes were essential for mycelial growth of *Aspergillus terreus* NTOU4989, regardless of the pH (3 or 7) of the growth medium and the incubation temperature (25 °C or 37 °C). The high correlation of gene expression within the same growth conditions over that between growth conditions suggests that the differences in gene expression were due to differences in the growth conditions.

A number of stress response genes were identified in the transcriptome of *A*. *terreus*; most of these were up-regulated at 45 °C-pH 3 over 25 °C-pH 7. Heat stress causes denaturation of proteins while heat shock proteins (Hsps) belong to a family of proteins related to unfold and refold mismatched or aggregated proteins [35]. Heat stress was reported to up-regulate expression of various Hsps [35] but genes related to heat shock proteins were mostly down-regulated in *A*. *terreus*, with heat shock transcriptional factor 2 gene up-regulated. The effect of Hsps might be a short-term adaptation (e.g. Hsp90 in *Candida albicans* [36]). In this study, *A*. *terreus* was incubated for 10 days for RNA-seq analysis based on the growth curve of the combined effects of salinity, temperature and pH experiment. The molecular adaptations expressed by *A*. *terreus* may represent a long-term adaptation strategy to temperature. Ubiquitin was regarded as a type of low-molecular-weight Hsps and this molecule was both up- and down-regulated. High temperature induces reactive oxygen species (ROS), and genes of catalases and superoxide dismutases were up-regulated to possibly diminish the effect of ROS [37].

A number of proteins were reported to be related to pH responses of fungi: up-regulation of ATP-binding cassette transporters (ABC), phenylalanine ammonia lyase (PAL) [38] and gamma-aminobutyric acid [39], and down-regulation of pH-response transcription factor (PacC) and acetyl xylan esterase [39–42] in acidic pH. Gamma-aminobutyric acid and PAL were up-regulated while PacC was down-regulated in *A*. *terreus* and these results may suggest a stress response to pH. ABC was both up- and down-regulated, suggesting these genes, together with acetyl xylan esterase (up-regulated in this study), might not involve in pH homeostasis in *A*. *terreus*. Hydrogen ion transmembrane transport proteins involve in transporting hydrogen ions across a membrane and genes of these proteins were mostly up-regulated in *A*. *terreus*.

Genes related to arginine metabolism, glycerolipid metabolism (high osmolarity glycerol pathway, HOG), mitogen-activated protein kinase (MAPK) and linoleic acid were up-regulated in both growth conditions and might be involved in intracellular osmotic balance of *A*. *terreus*. Linoleic acid was both up- and down-regulated, suggesting a salinity stress in both growth conditions [43]. Arginine was found to enhance conidia production of *A*. *oryzae* under salinity stress [43]. Genes related to arginine metabolism were predominantly up-regulated in *A*. *terreus* at 45 °C-pH 3. Arginine was suggested to have a positive effect on acidic pH during propionic acid fermentation in *Propionibacterium acidipropionici* [44]. In a comparative transcriptome analysis of the marine fungus *Corollospora maritima* when grown in a freshwater and a seawater media, transcripts of aspartic-type peptidase activities and a choline sulfatase were up-regulated and concluded to be related to osmotic regulation [45]; these genes were also found to be up-regulated in this study. However, no expression of the endoplasmic reticulum transmembrane protein (MPS), predicted to be involved in osmotic homeostasis, was found in *A*. *terreus*, in contrast to 4- to 25-fold up-regulation in the seawater condition in *C*. *maritima* [45]. The similarities and differences in the molecular responses to salt adaptations in marine Ascomycota may be related to their phylogenetic affiliation (*Aspergillus terreus* (Eurotiales), *Corollospora maritima* (Microascales)) and/or their environmental origin. For the latter aspect, *C*. *maritima* is a cosmopolitan marine fungus growing on wood/within sand grains while *A*. *terreus* can be found in both terrestrial and marine environments including the deep-sea [26]. Based on phylogenetic analyses of the calmodulin gene, isolates of *A*. *terreus* cultured from marine/sea-related environments intermixed with those cultured from terrestrial habitats, suggesting that the marine isolates are not phylogenetically distinct [46,47]. The *Aspergillus terreus* isolate in this study may be of a terrestrial origin as the molecular mechanisms expressed towards salt tolerance (arginine biosynthesis, HOG, MAPK) are similar to those expressed by other terrestrial *Aspergillus* species, such as *A*. *oryzae* [43], *A*. *nidulans* [48], *A*. *fumigatus* [49] and *A*. *montevidensis* [50]. *Aspergillus* species have been shown to be able to tolerate a range of environmental stressors by their repertoire of stress-related genes [37, 41, 49–57] and were found ubiquitous in deep-sea environments.

Down-regulation of chitinase genes was related to oxidative stress in *Aspergillus flavus* [54]. Producing secondary metabolites and antioxidant enzymes, maintaining cell wall integrity, regulating primary metabolism to meet energetic requirements and recycling damaged cellular components were reported to be integral components of the stress responses of *A*. *flavus* [54]. Down-regulation of chitinase may be related to maintenance of cell wall integrity of *A*. *terreus* at 45 °C-pH 3 [54]. However, chitinase genes were found to be up-regulated under carbon starvation in *A*. *nidulans* [52] and it was speculated to be a result of reallocation of cellular resources to primary metabolism [54].

Pyruvate decarboxylase, a gene related to anaerobic ethanol fermentation in yeasts, was up-regulated at 45 °C-pH 3 by *A*. *terreus*, an observation reported to be related to hypoxic conditions in *A*. *fumigatus* [53]. For *A*. *terreus* in this study, anaerobic respiration may have occurred in the flasks incubated at 45 °C due to a lower oxygen concentration over those incubated at 25 °C, in conjunction with aerobic respiration [58].

Many genes related to one GO (Gene Ontology) term ‘structural constituent of ribosome’ (a molecule that contributes to the structural integrity of the ribosome) were only up-regulated at 45 °C-pH 3 by *A*. *terreus*. These unknown proteins may ensure proper protein synthesis under temperature, pH and/or salinity stresses.

### Ecology of sediment fungi at hydrothermal vents

Both marine and terrestrial fungi are present in the seabed and whether these fungi are active depends on their physiological and genetic adaptabilities to the environmental conditions. In this study, different physiological groups in relation to temperature, salinity and pH were found for fungi isolated from various substrates at/near Kueishan Island Hydrothermal Vent Field. Majority of the tested fungi were temperature-sensitive while some had comparatively wide pH, salinity and temperature growth ranges. These fungi may be able to adapt to changes in physical and chemical conditions of the habitat, but their growth limit of ≤37 °C implies that they might not be active at/near the hydrothermal vents. *Aspergillus* species have a high genetic adaptability to physical/chemical stresses [55]. *Aspergillus terreus* NTOU4989 was the only temperature-tolerant fungus showing growth at 45 °C, and it may be active near the vents.

## Conclusions

The growth responses of the ten tested fungi could be referred to three different types: (1) wide pH, salinity and temperature ranges (4 isolates), (2) salinity-dependent and temperature-sensitive (5 isolates), and (3) temperature-tolerant (*Aspergillus terreus* NTOU4989) with growth at 45 °C. Majority of these isolates may play no role at the hydrothermal vents of Kueishan Island. *Aspergillus terreus* NTOU4989, isolated from the yellow sediment (with sulfur granules), may be active at/near the vents. The transcriptome analysis revealed a number of independent or coordinated stress responses expressed by *A*. *terreus* NTOU4989 grown in/at a low pH and a high temperature in seawater, possible conditions near the vents at Kueishan Island. A degradative enzyme study of *A*. *terreus* NTOU4989 under a high temperature and a low pH will provide evidence if this fungus plays a degrader role at/near the hydrothermal vent ecosystem of Kueishan Island.

## Supporting information

S1 Table Number and quality of filtered reads obtained from transcriptome analysis of *Aspergillus terreus*. (PDF)

S2 Table Mapping results of clean reads obtained from transcriptome analysis of *Aspergillus terreus*. (PDF)

S3 Table Types of transcript obtained from transcriptome analysis of *Aspergillus terreus*. (PDF)

S4 Table Characteristics of mapped genes obtained from transcriptome analysis of *Aspergillus terreus*. (PDF)

S5 Table Up- and down-regulation of differential expressed genes by *Aspergillus terreus* between 45 °C and pH 3 (A45-1, A45-2) and 25 °C and pH 7 (A25-1, A25-2). (PDF)

S1 Fig Growth curves showing changes in optical density (OD) over time for (a) *Aspergillus terreus* NTOU4989, (b) *Aspergillus aculeatus* NTOU4990, (c) *Aspergillus sydowii* NTOU4991, (d) *Penicillium madriti* NTOU4992, (e) *Verticillium dahlia* NTOU4998, (f) *Penicillium sumatrense* NTOU5001, (g) *Trichoderma harzianum* NTOU5005, (h) *Penicillium oxalicum* NTOU5115, (i) *Microascus brevicaulis* NTOU5292 and (j) *Fodinomyces uranophilus* NTOU5428, cultured in/at 2 salinities (freshwater (0‰) and seawater (30‰)), 4 pHs (pH 3, pH 5, pH 7 and pH 9) and 4 temperatures (15°C, 25°C, 37°C and 45°C). The x-axes represent time in days, while the y-axes show growth as measured using optical density at 630 nm. The color lines (magenta, pH 3; orange, pH 5; green, pH 7; dark blue, pH 9) represent empirical growth curve of 8 replicates fitted with Richards II model. Error bars indicate standard deviations.
(EPS)

S2 Fig Venn diagram showing the number of expressed genes in RNA-seq analysis of *Aspergillus terreus* incubated at 25°C and pH 7 (A25-1, A25-2) and at 45°C and pH 3 (A45-1, A45-2). (EPS)

S3 Fig Correlation between samples and replicates of RNA-seq analysis of *Aspergillus terreus* incubated at 25°C and pH 7 (A25-1, A25-2) and at 45°C and pH 3 (A45-1, A45-2): (a) Correlation coefficients based on Pearson method, (b) Cluster dendrogram based on Euclidean distance, (c) Heatmap showing a hierarchical clustering of differential expressed genes. (EPS)

S4 Fig Number of up- and down-regulated differential expressed genes between samples and replicates of RNA-seq analysis of *Aspergillus terreus* incubated at 25°C and pH 7 (A25-1, A25-2) and at 45°C and pH 3 (A45-1, A45-2). (EPS)
