# Supplementary material for: Growth study under combined effects of temperature, pH and salinity and transcriptome analysis revealed adaptations of Aspergillus terreus NTOU4989 to the extreme conditions at Kueishan Island Hydrothermal Vent Field, Taiwan
Source: PLoS One. 2020 May 26;15(5):e0233621. doi: 10.1371/journal.pone.0233621 (PMC7250430; doi:10.1371/journal.pone.0233621)
Supplement: S1 Table — (PDF) [file pone.0233621.s001.pdf]

S1 Table. Number and quality of filtered reads obtained from transcriptome analysis of *Aspergillus terreus* .

Sample	Clean Reads (M)	Clean Bases (G)	Q20 (%)	Q30 (%)	GC (%)	Read length (bp)
25°C-pH7-1	40.2001M	6.0300G	97.48	93.3	57.44	150
25°C-pH7-2	40.0837M	6.0126G	97.51	93.33	57.46	150
45°C-pH3-1	41.5902M	6.2385G	97.48	93.3	57.71	150
45°C-pH3-2	40.3249M	6.0487G	97.47	93.25	57.75	150
