# Supplementary material for: Growth study under combined effects of temperature, pH and salinity and transcriptome analysis revealed adaptations of Aspergillus terreus NTOU4989 to the extreme conditions at Kueishan Island Hydrothermal Vent Field, Taiwan
Source: PLoS One. 2020 May 26;15(5):e0233621. doi: 10.1371/journal.pone.0233621 (PMC7250430; doi:10.1371/journal.pone.0233621)
Supplement: S2 Table — (PDF) [file pone.0233621.s002.pdf]

S2 Table. Mapping results of clean reads obtained from transcriptome analysis of *Aspergillus terreus* .

Sample	Total Reads	Total Mapped	Reads Map To '+'	Reads Map To '-'	Splice Mapped	Non-splice Mapped	Unique Mapped	Multiple Mapped
25°C-pH7-1	40200104	35918218(89.35%)	17968136(44.70%)	17950082(44.65%)	7629765(18.98%)	28288453(70.37%)	29943312(74.49%)	5974906(14.86%)
25°C-pH7-2	40083702	35912224(89.59%)	17969694(44.83%)	17942530(44.76%)	7588871(18.93%)	28323353(70.66%)	30106048(75.11%)	5806176(14.49%)
45°C-pH3-1	41590228	36690800(88.22%)	18342168(44.10%)	18348632(44.12%)	9497802(22.84%)	27192998(65.38%)	30244368(72.72%)	6446432(15.50%)
45°C-pH3-2	40324892	36162156(89.68%)	18077978(44.83%)	18084178(44.85%)	9611136(23.83%)	26551020(65.84%)	29672086(73.58%)	6490070(16.09%)
