# Supplementary material for: Growth study under combined effects of temperature, pH and salinity and transcriptome analysis revealed adaptations of Aspergillus terreus NTOU4989 to the extreme conditions at Kueishan Island Hydrothermal Vent Field, Taiwan
Source: PLoS One. 2020 May 26;15(5):e0233621. doi: 10.1371/journal.pone.0233621 (PMC7250430; doi:10.1371/journal.pone.0233621)
Supplement: S3 Table — (PDF) [file pone.0233621.s003.pdf]

S3 Table. Types of transcript obtained from transcriptome analysis of *Aspergillus terreus* .

Total novel transcript	Coding transcript	Non-coding transcript	Novel isoform	Novel gene
4471	3656	815	3598	58
