# Supplementary material for: Growth study under combined effects of temperature, pH and salinity and transcriptome analysis revealed adaptations of Aspergillus terreus NTOU4989 to the extreme conditions at Kueishan Island Hydrothermal Vent Field, Taiwan
Source: PLoS One. 2020 May 26;15(5):e0233621. doi: 10.1371/journal.pone.0233621 (PMC7250430; doi:10.1371/journal.pone.0233621)
Supplement: S4 Table — (PDF) [file pone.0233621.s004.pdf]

S4 Table. Characterisitics of mapped genes obtained from transcriptome analysis of *Aspergillus terreus* .

**Sample**	**Total clean reads**	**Total mapping ratio**	**Total gene number**	**Known gene number**	**Novel gene number**
25°C-pH7-1	40200104	66.77%	8830	8774	56
25°C-pH7-2	40083702	66.39%	8816	8759	57
45°C-pH3-1	41590228	67.05%	8659	8603	56
45°C-pH3-2	40324892	68.73%	8570	8515	55
