# Supplementary material for: Growth study under combined effects of temperature, pH and salinity and transcriptome analysis revealed adaptations of Aspergillus terreus NTOU4989 to the extreme conditions at Kueishan Island Hydrothermal Vent Field, Taiwan
Source: PLoS One. 2020 May 26;15(5):e0233621. doi: 10.1371/journal.pone.0233621 (PMC7250430; doi:10.1371/journal.pone.0233621)
Supplement: S5 Table — (PDF) [file pone.0233621.s005.pdf]

S5 Table. Up- and down-regulation of differential expressed genes by Aspergillus terreus between 45 °C and pH 3 (A45-1, A45-2) and 25 °C and pH 7 (A25-1, A25-2).

GeneID	Length	A25-1_25-2-Expression	A45-1_45-2-Expression	log2FoldChange(A45-1_45-2/A25-1_25-2)	Pvalue	Padj	Up/Down-Regulation	Symbol	Description	Pathway	GO Component	GO Function	GO Process	Blast nr
4321032	1650	0	2094.23	10.50957977	2.44E-56	2.01E-54	Up	ATEG_05338	retinal dehydrogenase 2	ko01100//Metabolic pathways;ko01110//Biosynthesis of secondary metabolites;ko00350//Tyrosine metabolism;ko00010//Glycolysis / Gluconeogenesis;ko00360//Phenylalanine metabolism;ko00410//beta-Alanine metabolism;ko00340//Histidine [illegible]	-	GO:0004029//aldehyde dehydrogenase (NAD) activity Up-regulation of ALDHs is a stress response in bacteria (environmental and chemical stress), plants (dehydration, salinity and oxidative stress), yeast (ethanol exposure and [illegible]	GO:0055114//oxidation-reduction process	gi|115397849|ref|XP_001214516.1|/0/retinal dehydrogenase 2 [Aspergillus terreus NIH2624]
4353091	1512	2.81	2164.94	9.366166183	3.63E-78	5.00E-76	Up	ATEG_08169	hypothetical protein	ko01100//Metabolic pathways;ko00780//Biotin metabolism	-	GO:0016740//transferase activity;GO:0030170//pyridoxal phosphate binding It is also known to function as a singlet oxygen [illegible]	GO:0009058//biosynthetic process	gi|115433306|ref|XP_001216790.1|/0/predicted protein [Aspergillus terreus NIH2624]
4318345	1434	0.56	616.25	8.606372666	6.07E-40	3.00E-38	Up	ATEG_03935	similar to potential aspartate aminotransferase	ko01100//Metabolic pathways;ko00380//Tryptophan metabolism	-	GO:0008483//transaminase activity;GO:0030170//pyridoxal phosphate binding	GO:0009058//biosynthetic process	gi|115391217|ref|XP_001213113.1|/0/hypothetical protein ATEG_03935 [Aspergillus terreus NIH2624]
4316577	1839	0	470.22	8.59973915	4.71E-33	1.77E-31	Up	ATEG_02031	acid phosphatase precursor	ko04111//Cell cycle - yeast	-	GO:0003993//acid phosphatase activity;GO:0046872//metal ion binding	GO:0016311//dephosphorylation	[illegible]|XP_001211209.1|/0/acid phosphatase precursor [Aspergillus [illegible]
4317446	327	0	439.35	8.498441605	7.20E-32	2.61E-30	Up	ATEG_02935	60S acidic ribosomal protein P1	ko03010//Ribosome	GO:0005840//ribosome	GO:0003735//structural constituent of ribosome	GO:0006414//translational elongation	[illegible]|XP_001212113.1|/9.688e-70/60S acidic ribosomal protein P1 [Aspergillus [illegible]

4320728	1878	0	346.9	8.199819692	4.27E-29	1.37E-27	Up	ATEG_05187	dual specificity mitogen-activated protein kinase kinase dSOR1	ko04011//MAPK signaling pathway - yeast	GO:0005634//nucleus;GO:0005829//cytosol;GO:0005934//cellular bud tip;GO:0005935//cellular bud neck;GO:0031416//NatB complex;GO:1990315//Mcs4 RR-MAPKKK complex	GO:0004596//peptide alpha-N-acetyltransferase activity;GO:0004708//MAP kinase kinase activity;GO:0004715//non-membrane spanning protein tyrosine kinase activity;GO:0005078//MAP-kinase scaffold activity;GO:0005524//ATP binding (Drug resistence)	GO:0000169//activation of MAPK activity involved in osmosensory signaling pathway;GO:0000208//MAPK import into nucleus involved in osmosensory signaling pathway;GO:0006972//hyperosmotic response;GO:0007015//actin filament organization;GO:0010971//positive	gi|115397547|ref|XP_001214365.1|/0/dual specificity mitogen-activated protein kinase kinase dSOR1 [Aspergillus terreus NIH2624]
4320749	1650	0	377.46	8.153389104	7.64E-28	2.36E-26	Up	ATEG_05616	imidazole glycerol phosphate synthase hisHF	ko01100//Metabolic pathways;ko01110//Biosynthesis of secondary metabolites;ko01230//Biosynthesis of amino acids;ko00340//Histidine metabolism	GO:0005634//nucleus;GO:0005829//cytosol	GO:0000107//imidazoleglycerol-phosphate synthase activity;GO:0004359//glutaminase activity;GO:0016833//oxo-acid-lyase activity (Phosphate-activated mitochondrial glutaminase (GLS1) is suggested to be linked with elevated metabolism, decreased intracellular reactive oxygen species (ROS) levels, and overall decreased DNA oxidation in both normal and stressed cells)	GO:0000105//histidine biosynthetic process;GO:0006541//glutamine metabolic process	gi|115398405|ref|XP_001214794.1|/0/imidazole glycerol phosphate synthase hisHF [Aspergillus terreus NIH2624]

4353094	7820	15.7	3552	7.92975957	2.19E-64	2.23E-62	Up	ATEG_08172	similar to polyketide synthase	ko00350//Tyrosine metabolism	-	GO:0016491//oxidoreductase activity;GO:0016740//transferase activity;GO:0031177//phosphopantetheine binding (fatty acid synthesis? PKS? NRPS?)	GO:0055114//oxidation-reduction process	gi|115433312|ref|XP_001216793.1|/0/hypothetical protein ATEG_08172 [Aspergillus terreus NIH2624]
4315574	2820	0	286.97	7.90837261	4.43E-26	1.27E-24	Up	ATEG_01604	C-1-tetrahydrofolate synthase, mitochondrial precursor	ko01100//Metabolic pathways;ko00670//One carbon pool by folate	-	GO:0004329//formate-tetrahydrofolate ligase activity;GO:0004488//methylenetetrahydrofolate dehydrogenase (NADP+) activity;GO:0005524//ATP binding (pigment synthesis?)	GO:0009396//folic acid-containing compound biosynthetic process;GO:0055114//oxidation-reduction process	gi|115384844|ref|XP_001208969.1|/0/C-1-tetrahydrofolate synthase, mitochondrial precursor [Aspergillus terreus NIH2624]
4353117	1867	5.61	1546.86	7.725735437	7.59E-37	3.41E-35	Up	ATEG_08168	hypothetical protein	ko00380//Tryptophan metabolism;ko00071//Fatty acid degradation	-	GO:0004497//monooxygenase activity;GO:0005506//iron ion binding;GO:0016705//oxidoreductase activity, acting on paired donors, with incorporation or reduction of molecular oxygen;GO:0020037//heme binding (iron binding)	GO:0055114//oxidation-reduction process	gi|115433304|ref|XP_001216789.1|/0/predicted protein [Aspergillus terreus NIH2624]
4322785	1284	3.36	638.66	7.557065225	3.65E-50	2.43E-48	Up	ATEG_07693	hypothetical protein	-	-	-	-	gi|115401452|ref|XP_001216314.1|/0/predicted protein [Aspergillus terreus NIH2624]

4353092	1128	8.41	1169.74	7.410720021	9.35E-80	1.37E-77	Up	ATEG_08170	similar to catalytic	ko01100//Metabolic pathways;ko00600//Sphingolipid metabolism	GO:0016021//integral component of membrane	GO:0005506//iron ion binding;GO:0016491//oxidoreductase activity (iron binding)	GO:0006633//fatty acid biosynthetic process;GO:0055114//oxidation-reduction process	gi|115433308|ref|XP_001216791.1|/0/hypothetical protein ATEG_08170 [Aspergillus terreus NIH2624]
4319599	1626	0	182.09	7.330280436	1.46E-21	3.26E-20	Up	ATEG_10049	hypothetical protein	-	GO:0005783//endoplasmic reticulum	GO:0003824//catalytic activity	-	gi|115385609|ref|XP_001209351.1|/0/conserved hypothetical protein [Aspergillus terreus NIH2624]
4322544	1116	0.56	246.31	7.301625389	2.27E-25	6.25E-24	Up	ATEG_07920	hypothetical protein	ko01100//Metabolic pathways;ko00500//Starch and sucrose metabolism	GO:0005576//extracellular region	GO:0004553//hydrolase activity, hydrolyzing O-glycosyl compounds;GO:0030248//cellulose binding (maltose metabolism?)	GO:0005975//carbohydrate metabolic process	gi|115401906|ref|XP_001216541.1|/0/conserved hypothetical protein [Aspergillus terreus NIH2624]
4315658	1578	0	176.03	7.27625281	4.81E-21	1.05E-19	Up	ATEG_01307	hypothetical protein	-	-	GO:0016810//hydrolase activity, acting on carbon-nitrogen (but not peptide) bonds	-	gi|115384250|ref|XP_001208672.1|/0/conserved hypothetical protein [Aspergillus terreus NIH2624]

4316513	2490	0	172.89	7.265508978	4.79E-21	1.05E-19	Up	ATEG_02621	similar to peroxisomal half ABC transporter	ko04146//Peroxisome;ko02010//ABC transporters	GO:0005779//integral component of peroxisomal membrane	GO:0005325//peroxisomal fatty-acyl-CoA transporter activity;GO:0005524//ATP binding;GO:0042626//ATPase activity, coupled to transmembrane movement of substances (degradation of fatty acid?)	GO:0006635//fatty acid beta-oxidation;GO:0015910//peroxisomal long-chain fatty acid import;GO:0015916//fatty-acyl-CoA transport;GO:0042758//long-chain fatty acid catabolic process;GO:0042760//very long-chain fatty acid catabolic process;GO:0055085//transmembrane transport	gi|115388587|ref|XP_001211799.1|/0/hypothetical protein ATEG_02621 [Aspergillus terreus NIH2624]
4322619	996	53.81	5169.37	7.150144146	9.99E-267	3.12E-263	Up	ATEG_07680	hypothetical protein	-	GO:0005737//cytoplasm	GO:0008677//2-dehydropantoate 2-reductase activity;GO:0050661//NADP binding (against oxidative stress?)	GO:0015940//pantothenate biosynthetic process;GO:0055114//oxidation-reduction process	gi|115401426|ref|XP_001216301.1|/0/predicted protein [Aspergillus terreus NIH2624]
4320850	2343	0	155.9	7.112004555	7.97E-20	1.61E-18	Up	ATEG_05172	similar to peptide transporter MTD1	-	GO:0016021//integral component of membrane	-	GO:0055085//transmembrane transport	gi|115397517|ref|XP_001214350.1|/0/hypothetical protein ATEG_05172 [Aspergillus terreus NIH2624]
4316676	2115	0	147.35	7.041797162	2.13E-19	4.19E-18	Up	ATEG_02421	similar to molybdenum cofactor biosynthesis protein	-	GO:0016021//integral component of membrane;GO:0033179//proton-transporting V-type ATPase, V0 domain	GO:0015078//hydrogen ion transmembrane transporter activity (pH control)	GO:0006777//Mo-molybdopterin cofactor biosynthetic process;GO:0015991//ATP hydrolysis coupled proton transport;GO:0032324//molybdopterin cofactor biosynthetic process	gi|115388187|ref|XP_001211599.1|/0/hypothetical protein ATEG_02421 [Aspergillus terreus NIH2624]

4318837	975	63.44	52435.7	7.010580886	1.03E-15	1.56E-14	Up	ATEG_07252	hypothetical protein	ko00460//Cyanoamino acid metabolism	-	GO:0016810//hydrolase activity, acting on carbon-nitrogen (but not peptide) bonds	GO:0006807//nitrogen compound metabolic process	gi|115386794|ref|XP_001209938.1|/0/conserved hypothetical protein [Aspergillus terreus NIH2624]
4315784	2493	0	118.48	6.706318492	5.22E-17	8.63E-16	Up	ATEG_01722	hypothetical protein	-	GO:0016021//integral component of membrane	-	-	gi|115385080|ref|XP_001209087.1|/0/conserved hypothetical protein [Aspergillus terreus NIH2624]
4355513	1392	10.1	741.13	6.578876689	1.03E-68	1.22E-66	Up	ATEG_00758	hypothetical protein	ko01100//Metabolic pathways;ko00380//Tryptophan metabolism	GO:0005737//cytoplasm	GO:0030170//pyridoxal phosphate binding;GO:0030429//kynureninase activity (tryptophan metabolism)	GO:0006569//tryptophan catabolic process;GO:0019805//quinolinate biosynthetic process;GO:0034354//'de novo' NAD biosynthetic process from tryptophan;GO:0043420//anthranilate metabolic process;GO:0097053//L-kynurenine catabolic process	gi|115492433|ref|XP_001210844.1|/0/conserved hypothetical protein [Aspergillus terreus NIH2624]
4353095	1281	0	108.63	6.572091829	4.06E-16	6.33E-15	Up	ATEG_08173	hypothetical protein	-	-	-	-	gi|115433314|ref|XP_001216794.1|/1.80026e-167/predicted protein [Aspergillus terreus NIH2624]

4322313	2919	0	104.35	6.566192648	3.60E-16	5.66E-15	Up	ATEG_06724	hypothetical protein	ko04144//Endocytosis	GO:0005634//nucleus	GO:0000981//RNA polymerase II transcription factor activity, sequence-specific DNA binding;GO:0003677//DNA binding;GO:0008270//zinc ion binding (regulation of genes involved in the stress response as well as pleiotropic drug resistance)	GO:0006357//regulation of transcription from RNA polymerase II promoter	gi|115400627|ref|XP_001215902.1|/0/conserved hypothetical protein [Aspergillus terreus NIH2624]
4322533	1314	0	97.42	6.467879678	1.54E-15	2.29E-14	Up	ATEG_07816	hypothetical protein	-	GO:0016021//integral component of membrane	-	-	gi|115401698|ref|XP_001216437.1|/0/conserved hypothetical protein [Aspergillus terreus NIH2624]
4322772	1593	27.46	1638.94	6.426788202	2.36E-140	9.22E-138	Up	ATEG_07721	hypothetical protein	-	GO:0016021//integral component of membrane	GO:0022891//substrate-specific transmembrane transporter activity	GO:0008643//carbohydrate transport;GO:0055085//transmembrane transport	gi|115401508|ref|XP_001216342.1|/0/conserved hypothetical protein [Aspergillus terreus NIH2624]
4321959	1419	0	92.37	6.387559449	4.95E-15	7.09E-14	Up	ATEG_06571	similar to triglyceride lipase-cholesterol esterase	ko00100//Steroid biosynthesis	GO:0016021//integral component of membrane	-	GO:0006629//lipid metabolic process	gi|115400321|ref|XP_001215749.1|/0/hypothetical protein ATEG_06571 [Aspergillus terreus NIH2624]
4319413	888	3.93	299.44	6.336344715	3.07E-34	1.21E-32	Up	ATEG_10008	hypothetical protein	ko00051//Fructose and mannose metabolism	-	GO:0016491//oxidoreductase activity	GO:0055114//oxidation-reduction process	gi|115385527|ref|XP_001209310.1|/1.74659e-118/predicted protein [Aspergillus terreus NIH2624]

4322835	951	264.57	13717.45	6.287205363	6.99E-237	8.20E-234	Up	ATEG_07681	hypothetical protein	ko01100//Metabolic pathways;ko01110//Biosynthesis of secondary metabolites;ko01130//Biosynthesis of antibiotics;ko00010//Glycolysis / Gluconeogenesis	-	-	-	gi|115401428|ref|XP_001216302.1|/0/conserved hypothetical protein [Aspergillus terreus NIH2624]
4318602	3369	0	81.55	6.151038841	1.32E-13	1.74E-12	Up	ATEG_03817	hypothetical protein	-	-	-	-	gi|115390981|ref|XP_001212995.1|/0/conserved hypothetical protein [Aspergillus terreus NIH2624]
4322617	1771	215.78	9554.13	6.066292801	5.87E-257	9.18E-254	Up	ATEG_07678	hypothetical protein	-	GO:0016021//integral component of membrane	-	-	gi|115401422|ref|XP_001216299.1|/0/conserved hypothetical protein [Aspergillus terreus NIH2624]
4353914	3348	0	75.16	6.043184111	4.93E-13	6.34E-12	Up	ATEG_09172	hypothetical protein	ko04111//Cell cycle - yeast	GO:0000796//condensin complex	-	GO:0007076//mitotic chromosome condensation	gi|115437374|ref|XP_001217794.1|/0/conserved hypothetical protein [Aspergillus terreus NIH2624]
4354493	4089	0	70.7	6.003438347	7.00E-13	8.93E-12	Up	ATEG_09719	hypothetical protein	ko04144//Endocytosis	-	GO:0005086//ARF guanyl-nucleotide exchange factor activity (vesicle budding, Golgi apparatus)	GO:0032012//regulation of ARF protein signal transduction;GO:0043547//positive regulation of GTPase activity	gi|115443068|ref|XP_001218341.1|/0/conserved hypothetical protein [Aspergillus terreus NIH2624]
4315935	908	5.61	306.93	5.990139088	1.92E-35	8.06E-34	Up	ATEG_01037	hypothetical protein	-	-	GO:0052689//carboxylic ester hydrolase activity (ester into alcohol and carboxylic acid)	GO:0008152//metabolic process	gi|115383710|ref|XP_001208402.1|/0/predicted protein [Aspergillus terreus NIH2624]

4317241	901	14.59	1186.17	5.933054677	3.44E-17	5.77E-16	Up	ATEG_02678	hypothetical protein	ko01100//Metabolic pathways;ko00040//Pentose and glucuronate interconversions	GO:0005634//nucleus;GO:0005829//cytosol	GO:0000166//nucleotide binding;GO:0016740//transferase activity;GO:0016887//ATPase activity	GO:0008152//metabolic process	gi|115388701|ref|XP_001211856.1|/0/conserved hypothetical protein [Aspergillus terreus NIH2624]
4322268	619	1039.73	41788.64	5.932859114	2.04E-262	4.80E-259	Up	ATEG_06521	similar to CipC2 protein	ko01100//Metabolic pathways;ko00270//Cysteine and methionine metabolism;ko00480//Glutathione metabolism	GO:0005737//cytoplasm	-	-	gi|115400221|ref|XP_001215699.1|/4.80225e-92/hypothetical protein ATEG_06521 [Aspergillus terreus NIH2624]
4322098	2235	644.76	24675.66	5.85202729	6.33E-230	6.60E-227	Up	ATEG_06274	hypothetical protein	ko01100//Metabolic pathways;ko00260//Glycine, serine and threonine metabolism	-	GO:0016614//oxidoreductase activity, acting on CH-OH group of donors;GO:0050660//flavin adenine dinucleotide binding oxidation of first hydroxyl group of glucose)	GO:0055114//oxidation-reduction process	gi|115399708|ref|XP_001215452.1|/0/predicted protein [Aspergillus terreus NIH2624]
4317425	1542	0	198.53	5.847504943	1.42E-10	1.49E-09	Up	ATEG_02949	hypothetical protein	-	-	GO:0008080//N-acetyltransferase activity (protection from oxidative stress)	-	gi|115389244|ref|XP_001212127.1|/0/conserved hypothetical protein [Aspergillus terreus NIH2624]
4316709	1836	0	62.85	5.833372202	5.48E-12	6.54E-11	Up	ATEG_02281	hypothetical protein	-	-	GO:0016491//oxidoreductase activity;GO:0071949//FAD binding (a vital role in energy transfer and utilization during fungal growth and mycelia aggregation)	GO:0055114//oxidation-reduction process	gi|115387907|ref|XP_001211459.1|/0/conserved hypothetical protein [Aspergillus terreus NIH2624]

4354462	762	0	60.53	5.753779494	1.39E-11	1.60E-10	Up	ATEG_09511	hypothetical protein	-	-	-	-	gi|115442652|ref|XP_001218133.1|/0/conserved hypothetical protein [Aspergillus terreus NIH2624]
4315987	675	0	59.31	5.698611896	3.12E-11	3.49E-10	Up	ATEG_01493	phosphoribosylglycinamide formyltransferase	ko01100//Metabolic pathways;ko01110//Biosynthesis of secondary metabolites;ko01130//Biosynthesis of antibiotics;ko00230//Purine metabolism;ko00670//One carbon pool by folate	GO:0005634//nucleus;GO:0005737//cytoplasm	GO:0004644//phosphoribosylglycinamide formyltransferase activity (purine biosynthesis)	GO:0006189//'de novo' IMP biosynthetic process;GO:0046084//adenine biosynthetic process	gi|115384622|ref|XP_001208858.1|/5.79454e-159/phosphoribosylglycinamide formyltransferase [Aspergillus terreus NIH2624]
4354316	1281	26.31	1316.8	5.69386988	2.70E-23	6.60E-22	Up	ATEG_09542	hypothetical protein	-	-	-	-	gi|115442714|ref|XP_001218164.1|/0/predicted protein [Aspergillus terreus NIH2624]
4322101	1675	208.53	7083.82	5.686212815	2.26E-288	1.06E-284	Up	ATEG_06277	hypothetical protein	ko01100//Metabolic pathways;ko01110//Biosynthesis of secondary metabolites	-	GO:0005506//iron ion binding;GO:0009055//electron carrier activity;GO:0016705//oxidoreductase activity, acting on paired donors, with incorporation or reduction of molecular oxygen;GO:0020037//heme binding (iron binding)	GO:0055114//oxidation-reduction process	gi|115399720|ref|XP_001215455.1|/0/predicted protein [Aspergillus terreus NIH2624]

4316903	1377	0	57.9	5.672605498	3.56E-11	3.94E-10	Up	ATEG_01911	hypothetical protein	-	GO:0016021//integral component of membrane	GO:0016740//transferase activity	-	gi|115387167|ref|XP_001211089.1|/0/predicted protein [Aspergillus terreus NIH2624]
4318838	2252	22.39	761.97	5.567264863	6.08E-63	5.95E-61	Up	ATEG_07253	hypothetical protein	ko01100//Metabolic pathways;ko01220//Degradation of aromatic compounds	GO:0005634//nucleus	GO:0003677//DNA binding;GO:0008270//zinc ion binding (zinc binding)	GO:0006351//transcription, DNA-templated	gi|115386796|ref|XP_001209939.1|/0/predicted protein [Aspergillus terreus NIH2624]
4315953	2127	0	50.54	5.492759348	2.30E-10	2.35E-09	Up	ATEG_01477	similar to dihydroorotate dehydrogenase	ko01100//Metabolic pathways;ko00240//Pyrimidine metabolism	GO:0005737//cytoplasm	GO:0004152//dihydroorotate dehydrogenase activity (pyrimidine biosynthesis)	GO:0006207//'de novo' pyrimidine nucleobase biosynthetic process;GO:0055114//oxidation-reduction process	gi|115384590|ref|XP_001208842.1|/0/hypothetical protein ATEG_01477 [Aspergillus terreus NIH2624]
4321046	2244	0	52.43	5.461752736	4.36E-10	4.32E-09	Up	ATEG_05353	hypothetical protein	ko01100//Metabolic pathways;ko01110//Biosynthesis of secondary metabolites;ko00564//Glycerophospholipid metabolism;ko00561//Glycerolipid metabolism;ko00591//Linoleic acid metabolism;ko00100//Steroid biosynthesis;ko00565//Ether lipid metabolism;ko00590//Arachidonic acid metabolism;ko00592//alpha-Linolenic acid metabolism	GO:0005737//cytoplasm;GO:0016020//membrane	GO:0004806//triglyceride lipase activity (lipid degradation)	GO:0016042//lipid catabolic process	gi|115397879|ref|XP_001214531.1|/0/conserved hypothetical protein [Aspergillus terreus NIH2624]

4322757	810	19.6	614.75	5.441118652	5.57E-56	4.36E-54	Up	ATEG_07915	hypothetical protein	ko01100//Metabolic pathways;ko01130//Biosynthesis of antibiotics;ko00270//Cysteine and methionine metabolism	-	-	-	gi|115401896|ref|XP_001216536.1|/0/predicted protein [Aspergillus terreus NIH2624]
4353150	1692	4.48	173.08	5.404698593	9.87E-24	2.45E-22	Up	ATEG_08389	similar to major facilitator superfamily	-	GO:0016021//integral component of membrane	-	GO:0055085//transmembrane transport	gi|115433747|ref|XP_001217010.1|/0/hypothetical protein ATEG_08389 [Aspergillus terreus NIH2624]
4315938	1083	0	48.54	5.383623931	8.48E-10	8.22E-09	Up	ATEG_01040	hypothetical protein	ko01100//Metabolic pathways;ko00564//Glycerophospholipid metabolism;ko00565//Ether lipid metabolism	GO:0016021//integral component of membrane	-	-	gi|115383716|ref|XP_001208405.1|/0/conserved hypothetical protein [Aspergillus terreus NIH2624]
4316876	2159	2.24	110.13	5.373371604	2.33E-16	3.72E-15	Up	ATEG_01896	hypothetical protein	ko01100//Metabolic pathways;ko00260//Glycine, serine and threonine metabolism	-	GO:0016614//oxidoreductase activity, acting on CH-OH group of donors;GO:0050660//flavin adenine dinucleotide binding (a vital role in energy transfer and utilization during fungal growth and mycelia aggregation)	GO:0055114//oxidation-reduction process	gi|115387137|ref|XP_001211074.1|/0/predicted protein [Aspergillus terreus NIH2624]

4317884	1617	35.31	1004.63	5.373111769	1.23E-88	2.21E-86	Up	ATEG_03190	hypothetical protein	ko01100//Metabolic pathways;ko01110//Biosynthesis of secondary metabolites;ko01130//Biosynthesis of antibiotics;ko01200//Carbon metabolism;ko00260//Glycine, serine and threonine metabolism;ko00010//Glycolysis / Gluconeogenesis;ko00620//Pyruvate metabolism;ko00280//Valine, leucine and isoleucine degradation;ko00640//Propanoate metabolism;ko00630//Glyoxylate and dicarboxylate metabolism;ko00020//Citrate cycle (TCA cycle)	GO:0016021//integral component of membrane	GO:0022891//substrate-specific transmembrane transporter activity	GO:0008643//carbohydrate transport;GO:0055085//transmembrane transport	gi|115389726|ref|XP_001212368.1|/0/conserved hypothetical protein [Aspergillus terreus NIH2624]
4319161	360	35.3	1001.1	5.364900056	4.55E-84	7.24E-82	Up	ATEG_07166	hypothetical protein	-	-	-	-	gi|115386622|ref|XP_001209852.1|/9.95484e-84/conserved hypothetical protein [Aspergillus terreus NIH2624]
4323479	1035	81.82	2214.86	5.332527733	2.63E-112	7.04E-110	Up	ATEG_08617	similar to oxidoreductase	-	-	GO:0010181//FMN binding;GO:0016491//oxidoreductase activity	GO:0055114//oxidation-reduction process	gi|115402253|ref|XP_001217203.1|/0/hypothetical protein ATEG_08617 [Aspergillus terreus NIH2624]

4322618	1363	304.86	7643.59	5.255910047	1.38E-220	1.30E-217	Up	ATEG_07679	hypothetical protein	-	-	-	-	gi|115401424|ref|XP_001216300.1|/0/predicted protein [Aspergillus terreus NIH2624]
4321194	3092	764.87	19075.4	5.251992688	3.82E-242	5.12E-239	Up	ATEG_04947	S-(hydroxymethyl)glutathione dehydrogenase	ko01100//Metabolic pathways;ko01110//Biosynthesis of secondary metabolites;ko01130//Biosynthesis of antibiotics;ko01200//Carbon metabolism;ko01220//Degradation of aromatic compounds;ko00350//Tyrosine metabolism;ko00010//Glycolysis / Gluconeogenesis; ko00071//Fatty acid degradation;ko00680//Methane metabolism	GO:0005634//nucleus;GO:0005829//cytosol	GO:0004022//alcohol dehydrogenase (NAD) activity;GO:0008270//zinc ion binding;GO:0033833//hydroxymethylfurfural reductase (NADH) activity;GO:0051903//S-(hydroxymethyl)glutathione dehydrogenase activity Up-regulation of ALDHs is a stress response in bacteria (environmental and chemical stress), plants (dehydration, salinity and oxidative stress), yeast (ethanol exposure and oxidative stress), Caenorhabditis elegans (lipid peroxidation) and mammals (oxidative stress and lipid peroxidation).	GO:0000947//amino acid catabolic process to alcohol via Ehrlich pathway;GO:0006069//ethanol oxidation;GO:0033859//furaldehyde metabolic process;GO:0046294//formaldehyde catabolic process	gi|115397067|ref|XP_001214125.1|/0/S-(hydroxymethyl)glutathione dehydrogenase [Aspergillus terreus NIH2624]

4319083	519	31.4	835.84	5.230063937	2.61E-67	2.95E-65	Up	ATEG_07360	hypothetical protein	-	GO:0009277//fungal-type cell wall	-	-	gi|115387010|ref|XP_001210046.1|/1.05232e-124/conserved hypothetical protein [Aspergillus terreus NIH2624]
4322129	1340	292.12	7149.05	5.205142501	1.08E-166	5.98E-164	Up	ATEG_06272	hypothetical protein	ko01100//Metabolic pathways;ko01220//Degradation of aromatic compounds	GO:0016021//integral component of membrane	GO:0016491//oxidoreductase activity;GO:0071949//FAD binding	GO:0055114//oxidation-reduction process	gi|115399700|ref|XP_001215450.1|/0/conserved hypothetical protein [Aspergillus terreus NIH2624]
4317624	1617	0	41.21	5.191467758	4.48E-09	4.07E-08	Up	ATEG_03232	isocitrate lyase	ko01100//Metabolic pathways;ko01110//Biosynthesis of secondary metabolites;ko01200//Carbon metabolism;ko00630//Glyoxylate and dicarboxylate metabolism	GO:0005782//peroxisomal matrix	GO:0004451//isocitrate lyase activity	GO:0009062//fatty acid catabolic process;GO:0015976//carbon utilization;GO:0045733//acetate catabolic process	gi|115389810|ref|XP_001212410.1|/0/isocitrate lyase [Aspergillus terreus NIH2624]
4322756	606	70.05	1673.84	5.156992928	2.08E-117	6.30E-115	Up	ATEG_07914	hypothetical protein	ko01100//Metabolic pathways;ko00360//Phenylalanine metabolism	-	GO:0016491//oxidoreductase activity	GO:0055114//oxidation-reduction process	gi|115401894|ref|XP_001216535.1|/1.68773e-142/conserved hypothetical protein [Aspergillus terreus NIH2624]
4319167	1651	3.92	136.49	5.14864681	1.04E-17	1.79E-16	Up	ATEG_07334	hypothetical protein	ko04113//Meiosis - yeast	GO:0016021//integral component of membrane	GO:0022891//substrate-specific transmembrane transporter activity	GO:0055085//transmembrane transport	gi|115386958|ref|XP_001210020.1|/0/conserved hypothetical protein [Aspergillus terreus NIH2624]

4317260	1392	22.4	531.22	5.060087968	1.67E-52	1.21E-50	Up	ATEG_02661	hypothetical protein	ko01100//Metabolic pathways;ko00600//Sphingolipid metabolism;ko00511//Other glycan degradation	GO:0005576//extracellular region	GO:0004348//glucosylceramidase activity;GO:0030248//cellulose binding;GO:0046557//glucan endo-1,6-beta-glucosidase activity	GO:0005975//carbohydrate metabolic process;GO:0006665//sphingolipid metabolic process	gi|115388667|ref|XP_001211839.1|/0/conserved hypothetical protein [Aspergillus terreus NIH2624]
4353093	4113	9.51	499.99	5.034284598	1.71E-10	1.77E-09	Up	ATEG_08171	multidrug resistance protein 4	ko02010//ABC transporters	GO:0016021//integral component of membrane;GO:0044464	GO:0005524//ATP binding;GO:0042626//ATPase activity, coupled to transmembrane movement of substances	GO:0055085//transmembrane transport	gi|115433310|ref|XP_001216792.1|/0/multidrug resistance protein 4 [Aspergillus terreus NIH2624]
4353116	2372	60.55	1369.32	5.009476953	4.74E-70	6.01E-68	Up	ATEG_08167	hypothetical protein	ko00380//Tryptophan metabolism;ko00071//Fatty acid degradation	-	GO:0005506//iron ion binding;GO:0016705//oxidoreductase activity, acting on paired donors, with incorporation or reduction of molecular oxygen;GO:0020037//heme binding	GO:0055114//oxidation-reduction process	gi|115433302|ref|XP_001216788.1|/0/predicted protein [Aspergillus terreus NIH2624]
4321780	1266	4676.74	93849.57	4.945081077	1.83E-258	3.44E-255	Up	ATEG_05685	formate dehydrogenase	ko01100//Metabolic pathways;ko01200//Carbon metabolism;ko00630//Glyoxylate and dicarboxylate metabolism;ko00680//Methane metabolism	GO:0005576//extracellular region;GO:0005829//cytosol	GO:0008863//formate dehydrogenase (NAD+) activity;GO:0016616//oxidoreductase activity, acting on the CH-OH group of donors, NAD or NADP as acceptor;GO:0051287//NAD binding	GO:0042183//formate catabolic process;GO:0055114//oxidation-reduction process	gi|115398548|ref|XP_001214863.1|/0/formate dehydrogenase [Aspergillus terreus NIH2624]

4354032	1887	0	44.88	4.918958419	8.81E-08	7.00E-07	Up	ATEG_09318	hypothetical protein	-	GO:0016021//integral component of membrane	-	-	gi|115437946|ref|XP_001217940.1|/0/conserved hypothetical protein [Aspergillus terreus NIH2624]
4315679	1488	42.62	1098.76	4.892237532	1.55E-18	2.81E-17	Up	ATEG_01829	hypothetical protein	ko01100//Metabolic pathways;ko00380//Tryptophan metabolism	-	GO:0020037//heme binding;GO:0051213//dioxygenase activity	GO:0055114//oxidation-reduction process	gi|115385294|ref|XP_001209194.1|/0/conserved hypothetical protein [Aspergillus terreus NIH2624]
4322099	5412	1593.32	31026.36	4.885517088	8.27E-194	6.47E-191	Up	ATEG_06275	6-methylsalicylic acid synthase	-	-	GO:0016740//transferase activity;GO:0031177//phosphopantetheine binding	GO:0008152//metabolic process	gi|115399712|ref|XP_001215453.1|/0/6-methylsalicylic acid synthase [Aspergillus terreus NIH2624]
4321195	576	139.56	2699.32	4.873801435	1.52E-154	7.93E-152	Up	ATEG_04948	glutathione-dependent formaldehyde-activating enzyme	ko01200//Carbon metabolism;ko00680//Methane metabolism	-	GO:0008270//zinc ion binding;GO:0051907//S-(hydroxymethyl)glutathione synthase activity	GO:0046294//formaldehyde catabolic process	gi|115397069|ref|XP_001214126.1|/1.11154e-142/glutathione-dependent formaldehyde-activating enzyme [Aspergillus terreus NIH2624]
4322625	1718	425.49	7642	4.771813198	2.08E-147	1.03E-144	Up	ATEG_07913	hypothetical protein	ko01100//Metabolic pathways;ko00360//Phenylalanine metabolism	GO:0016021//integral component of membrane	GO:0004497//monooxygenase activity;GO:0005506//iron ion binding;GO:0016705//oxidoreductase activity, acting on paired donors, with incorporation or reduction of molecular oxygen;GO:0020037//heme binding	GO:0055114//oxidation-reduction process	gi|115401892|ref|XP_001216534.1|/0/conserved hypothetical protein [Aspergillus terreus NIH2624]

4353212	645	2.8	82.21	4.721040321	3.02E-12	3.68E-11	Up	ATEG_08413	hypothetical protein	-	-	-	-	gi|115433795|ref|XP_001217034.1|/8.30465e-154/conserved hypothetical protein [Aspergillus terreus NIH2624]
4319518	1586	2.8	81.53	4.693889981	2.75E-12	3.36E-11	Up	ATEG_09972	hypothetical protein	-	GO:0016021//integral component of membrane	-	GO:0055085//transmembrane transport	gi|115385455|ref|XP_001209274.1|/0/predicted protein [Aspergillus terreus NIH2624]
4353827	1134	108.71	1827.95	4.658707025	2.20E-134	7.64E-132	Up	ATEG_09131	allergen Asp f 4	ko04144//Endocytosis	-	-	-	gi|115437208|ref|XP_001217753.1|/0/allergen Asp f 4 [Aspergillus terreus NIH2624]
4322442	1512	27.45	487.72	4.652761216	8.59E-41	4.36E-39	Up	ATEG_06379	similar to carboxylic acid permease	-	GO:0016021//integral component of membrane	GO:0022857//transmembrane transporter activity	GO:0008643//carbohydrate transport;GO:0055085//transmembrane transport	gi|115399936|ref|XP_001215557.1|/0/hypothetical protein ATEG_06379 [Aspergillus terreus NIH2624]
4322714	2367	416.09	8002.59	4.647716	1.27E-24	3.32E-23	Up	ATEG_07878	hypothetical protein	ko01100//Metabolic pathways;ko00500//Starch and sucrose metabolism	GO:0005783//endoplasmic reticulum;GO:0005794//Golgi apparatus;GO:0005886//plasma membrane;GO:0016021//integral component of membrane	GO:1901584//tetrapeptide transmembrane transporter activity	GO:1901583//tetrapeptide transmembrane transport	gi|115401822|ref|XP_001216499.1|/0/conserved hypothetical protein [Aspergillus terreus NIH2624]
4322097	1431	529.77	8488.76	4.608578486	9.52E-181	6.38E-178	Up	ATEG_06273	hypothetical protein	-	GO:0016021//integral component of membrane	GO:0005215//transporter activity	GO:0055085//transmembrane transport	gi|115399704|ref|XP_001215451.1|/0/conserved hypothetical protein [Aspergillus terreus NIH2624]

4322592	4817	258.37	4012.68	4.567382153	8.26E-168	4.85E-165	Up	ATEG_07502	hypothetical protein	ko01100//Metabolic pathways;ko00350//Tyrosine metabolism	-	GO:0016491//oxidoreductase activity;GO:0071949//FAD binding	GO:0055114//oxidation-reduction process	gi|115401070|ref|XP_001216123.1|/0/predicted protein [Aspergillus terreus NIH2624]
4321822	801	117.73	1722.06	4.461909879	1.48E-97	3.39E-95	Up	ATEG_05815	hypothetical protein	ko04144//Endocytosis	-	-	-	gi|115398808|ref|XP_001214993.1|/0/conserved hypothetical protein [Aspergillus terreus NIH2624]
4354133	1976	1965.86	27029.41	4.401853643	6.58E-209	5.61E-206	Up	ATEG_09537	serine-type carboxypeptidase F precursor	-	-	GO:0004185//serine-type carboxypeptidase activity	GO:0006508//proteolysis	gi|115442704|ref|XP_001218159.1|/0/serine-type carboxypeptidase F precursor [Aspergillus terreus NIH2624]
4320100	357	0.56	36.43	4.39977505	4.80E-07	3.49E-06	Up	ATEG_04855	hypothetical protein	-	-	-	-	gi|115396788|ref|XP_001214033.1|/5.15664e-81/conserved hypothetical protein [Aspergillus terreus NIH2624]
4322593	2610	739.92	9859.12	4.349667161	5.28E-147	2.48E-144	Up	ATEG_07503	similar to N,N-dimethylglycine oxidase	ko01100//Metabolic pathways;ko00260//Glycine, serine and threonine metabolism	-	GO:0016491//oxidoreductase activity	GO:0055114//oxidation-reduction process	gi|115401072|ref|XP_001216124.1|/0/hypothetical protein ATEG_07503 [Aspergillus terreus NIH2624]

4320622	2289	0	28.41	4.29947739	5.44E-06	3.38E-05	Up	ATEG_05581	hypothetical protein	ko01100//Metabolic pathways;ko01110//Biosynthesis of secondary metabolites;ko01130//Biosynthesis of antibiotics;ko01230//Biosynthesis of amino acids;ko00400//Phenylalanine, tyrosine and tryptophan biosynthesis	-	GO:0003855//3-dehydroquinate dehydratase activity	-	gi|115398335|ref|XP_001214759.1|/0/conserved hypothetical protein [Aspergillus terreus NIH2624]
4320385	3264	168.1	2165.22	4.291545545	4.11E-142	1.75E-139	Up	ATEG_04343	similar to na/K ATPase alpha 1 isoform	ko00190//Oxidative phosphorylation	GO:0016021//integral component of membrane	GO:0005524//ATP binding;GO:0016787//hydrolase activity;GO:0046872//metal ion binding	-	gi|115395640|ref|XP_001213521.1|/0/hypothetical protein ATEG_04343 [Aspergillus terreus NIH2624]
4354777	1629	1472.3	17965.94	4.213016691	8.27E-172	5.17E-169	Up	ATEG_00021	similar to oxidoreductase	ko01100//Metabolic pathways;ko00260//Glycine, serine and threonine metabolism	GO:0005622//intracellular	GO:0008812//choline dehydrogenase activity;GO:0033713;GO:0050660//flavin adenine dinucleotide binding	GO:0006066//alcohol metabolic process;GO:0042426//choline catabolic process;GO:0055114//oxidation-reduction process	gi|115490959|ref|XP_001210107.1|/0/hypothetical protein ATEG_00021 [Aspergillus terreus NIH2624]
4355398	864	28.54	608.45	4.211890605	1.24E-08	1.07E-07	Up	ATEG_00643	similar to possible secreted cellulose-binding protein	ko04144//Endocytosis	-	-	-	gi|115492203|ref|XP_001210729.1|/0/hypothetical protein ATEG_00643 [Aspergillus terreus NIH2624]
4319958	1131	6.71	110.32	4.195513205	1.51E-12	1.87E-11	Up	ATEG_04345	hypothetical protein	ko01100//Metabolic pathways;ko00520//Amino sugar and nucleotide sugar metabolism	-	-	-	gi|115395644|ref|XP_001213523.1|/0/predicted protein [Aspergillus terreus NIH2624]

4318937	4068	102.62	1575.87	4.191979873	1.01E-14	1.42E-13	Up	ATEG_07321	similar to ATP-binding cassette transporter	ko02010//ABC transporters	GO:0016021//integral component of membrane	GO:0005524//ATP binding;GO:0042626//ATPase activity, coupled to transmembrane movement of substances	GO:0055085//transmembrane transport	gi|115386932|ref|XP_001210007.1|/0/hypothetical protein ATEG_07321 [Aspergillus terreus NIH2624]
4315622	948	9.53	133.85	4.144444536	5.83E-17	9.60E-16	Up	ATEG_01013	hypothetical protein	-	GO:0016021//integral component of membrane	-	-	gi|115383662|ref|XP_001208378.1|/0/conserved hypothetical protein [Aspergillus terreus NIH2624]
4353678	2130	24.1	287.2	4.078380108	3.16E-31	1.12E-29	Up	ATEG_09081	dihydroxyacetone synthase	ko01200//Carbon metabolism;ko00680//Methane metabolism	-	GO:0003824//catalytic activity	GO:0008152//metabolic process	gi|115437012|ref|XP_001217703.1|/0/dihydroxyacetone synthase [Aspergillus terreus NIH2624]
4355551	876	0	20.28	4.062642766	1.95E-05	0.00011148	Up	ATEG_00790	hypothetical protein	-	-	GO:0016491//oxidoreductase activity	GO:0055114//oxidation-reduction process	gi|115492497|ref|XP_001210876.1|/0/conserved hypothetical protein [Aspergillus terreus NIH2624]
4315991	1940	100.86	1108.58	4.051258611	7.87E-86	1.30E-83	Up	ATEG_01687	similar to GABA permease	-	GO:0016021//integral component of membrane	GO:0015171//amino acid transmembrane transporter activity	GO:0003333//amino acid transmembrane transport	gi|115385010|ref|XP_001209052.1|/0/hypothetical protein ATEG_01687 [Aspergillus terreus NIH2624]
4354591	2868	10.65	138.11	4.047826929	9.95E-16	1.52E-14	Up	ATEG_10311	hypothetical protein	ko01100//Metabolic pathways;ko01220//Degradation of aromatic compounds	GO:0016021//integral component of membrane	GO:0004497//monooxygenase activity;GO:0071949//FAD binding	GO:0044550//secondary metabolite biosynthetic process;GO:0055085//transmembrane transport;GO:0055114//oxidation-reduction process	gi|115449643|ref|XP_001218659.1|/0/conserved hypothetical protein [Aspergillus terreus NIH2624]

4322667	2605	120.46	1317.27	4.047746936	2.14E-94	4.56E-92	Up	ATEG_07780	hypothetical protein	-	-	-	-	gi|115401626|ref|XP_001216401.1|/0/predicted protein [Aspergillus terreus NIH2624]
4323301	1431	423.78	4572.96	4.04431955	2.05E-125	6.64E-123	Up	ATEG_08735	hypothetical protein	ko01100//Metabolic pathways;ko00500//Starch and sucrose metabolism	GO:0005622//intracellular	GO:0005085//guanyl-nucleotide exchange factor activity	GO:0007264//small GTPase mediated signal transduction;GO:0043547//positive regulation of GTPase activity	gi|115402489|ref|XP_001217321.1|/0/conserved hypothetical protein [Aspergillus terreus NIH2624]
4316790	750	13.46	166.42	4.036514394	6.31E-19	1.18E-17	Up	ATEG_02406	hypothetical protein	-	GO:0016021//integral component of membrane	GO:0022891//substrate-specific transmembrane transporter activity	GO:0055085//transmembrane transport	gi|115388157|ref|XP_001211584.1|/0/conserved hypothetical protein [Aspergillus terreus NIH2624]
4323186	2114	119.36	1273.98	4.008245158	5.98E-65	6.31E-63	Up	ATEG_08671	hypothetical protein	-	GO:0016021//integral component of membrane	-	GO:0055085//transmembrane transport	gi|115402361|ref|XP_001217257.1|/0/conserved hypothetical protein [Aspergillus terreus NIH2624]
4354490	1162	12.33	149.71	3.997689175	3.62E-18	6.41E-17	Up	ATEG_09843	similar to acetyl xylan esterase	-	GO:0005576//extracellular region	GO:0046555//acetylxylan esterase activity	GO:0030245//cellulose catabolic process;GO:0045493//xylan catabolic process	gi|115443316|ref|XP_001218465.1|/0/hypothetical protein ATEG_09843 [Aspergillus terreus NIH2624]
4355505	1032	18.49	216.3	3.996146709	2.29E-23	5.62E-22	Up	ATEG_00750	similar to rAsp f 9	-	GO:0005618//cell wall	GO:0004553//hydrolase activity, hydrolyzing O-glycosyl compounds	GO:0005975//carbohydrate metabolic process;GO:0071555//cell wall organization	gi|115492417|ref|XP_001210836.1|/0/hypothetical protein ATEG_00750 [Aspergillus terreus NIH2624]

4322616	1428	15.14	176.08	3.958008748	2.48E-18	4.46E-17	Up	ATEG_07677	hypothetical protein	ko00561//Glycerolipid metabolism;ko00052//Galactose metabolism;ko00600//Sphingolipid metabolism;ko00603//Glycosphingolipid biosynthesis - globo series	GO:0042597//periplasmic space	GO:0016829//lyase activity	-	gi|115401420|ref|XP_001216298.1|/0/conserved hypothetical protein [Aspergillus terreus NIH2624]
4317653	2409	213.98	2160.63	3.933110557	3.35E-94	6.99E-92	Up	ATEG_02869	hypothetical protein	-	-	GO:0000166//nucleotide binding;GO:0003723//RNA binding	-	gi|115389084|ref|XP_001212047.1|/0/predicted protein [Aspergillus terreus NIH2624]
4319149	2229	0	18.45	3.912884271	4.54E-05	0.00024372	Up	ATEG_07255	hypothetical protein	-	GO:0005634//nucleus	GO:0000981//RNA polymerase II transcription factor activity, sequence-specific DNA binding;GO:0003677//DNA binding;GO:0008270//zinc ion binding	GO:0006357//regulation of transcription from RNA polymerase II promoter	gi|115386800|ref|XP_001209941.1|/0/conserved hypothetical protein [Aspergillus terreus NIH2624]
4319523	1068	43.11	456.58	3.894779274	8.70E-28	2.67E-26	Up	ATEG_09977	hypothetical protein	ko03010//Ribosome	GO:0016021//integral component of membrane	-	-	gi|115385465|ref|XP_001209279.1|/0/conserved hypothetical protein [Aspergillus terreus NIH2624]
4319103	1620	75.11	746.99	3.884420815	1.17E-41	6.11E-40	Up	ATEG_07338	similar to general amidase-C	ko00380//Tryptophan metabolism;ko00360//Phenylalanine metabolism;ko00330//Arginine and proline metabolism	-	GO:0016884//carbon-nitrogen ligase activity, with glutamine as amido-N-donor	-	gi|115386966|ref|XP_001210024.1|/0/hypothetical protein ATEG_07338 [Aspergillus terreus NIH2624]

4354062	1440	17.35	399.49	3.872942072	6.39E-06	3.92E-05	Up	ATEG_09270	choline transport protein	-	GO:0016021//integral component of membrane	GO:0015171//amino acid transmembrane transporter activity;GO:0015220//choline transmembrane transporter activity;GO:0034228//ethanolamine transmembrane transporter activity;GO:1901235//(R)-carnitine transmembrane transporter activity	GO:0003333//amino acid transmembrane transport;GO:0015871//choline transport;GO:0031460//glycine betaine transport;GO:0034229//ethanolamine transport;GO:1902270//(R)-carnitine transmembrane transport	gi|115437758|ref|XP_001217892.1|/0/choline transport protein [Aspergillus terreus NIH2624]
4321589	474	24.64	254.2	3.850733332	5.82E-23	1.40E-21	Up	ATEG_06073	hypothetical protein	ko01100//Metabolic pathways;ko00520//Amino sugar and nucleotide sugar metabolism	-	GO:0008168//methyltransferase activity	GO:0032259//methylation	gi|115399324|ref|XP_001215251.1|/2.27399e-114/predicted protein [Aspergillus terreus NIH2624]
4354849	1626	338.56	3167.31	3.836643579	1.80E-87	3.19E-85	Up	ATEG_00092	hypothetical protein	-	GO:0016021//integral component of membrane	-	GO:0055085//transmembrane transport	gi|115491101|ref|XP_001210178.1|/0/conserved hypothetical protein [Aspergillus terreus NIH2624]
4321371	444	12.89	139.45	3.835995981	1.27E-16	2.06E-15	Up	ATEG_05969	hypothetical protein	ko04144//Endocytosis	-	-	-	gi|115399116|ref|XP_001215147.1|/6.92883e-102/conserved hypothetical protein [Aspergillus terreus NIH2624]
4319415	1653	45.95	440.91	3.815575459	6.64E-36	2.86E-34	Up	ATEG_10010	hypothetical protein	ko01100//Metabolic pathways;ko00380//Tryptophan metabolism	-	GO:0001760//aminocarboxymuconate-semialdehyde decarboxylase activity;GO:0016787//hydrolase activity	GO:0006568//tryptophan metabolic process;GO:1904984//regulation of quinolinate biosynthetic process	gi|115385531|ref|XP_001209312.1|/0/predicted protein [Aspergillus terreus NIH2624]

4322686	942	22.41	234.7	3.803162171	8.67E-19	1.60E-17	Up	ATEG_07531	mitochondrial phosphate carrier protein	-	GO:0016021//integral component of membrane	-	GO:0055085//transmembrane transport	gi|115401128|ref|XP_001216152.1|/0/mitochondrial phosphate carrier protein [Aspergillus terreus NIH2624]
4353109	657	36.98	355.09	3.790358342	3.97E-33	1.49E-31	Up	ATEG_08136	hypothetical protein	-	-	-	-	gi|115433240|ref|XP_001216757.1|/7.7151e-170/predicted protein [Aspergillus terreus NIH2624]
4353690	1152	1.12	28.69	3.78923198	1.13E-05	6.65E-05	Up	ATEG_09069	aryl-alcohol dehydrogenase	ko01100//Metabolic pathways;ko00051//Fructose and mannose metabolism;ko00650//Butanoate metabolism;ko00591//Linoleic acid metabolism	-	-	-	gi|115436968|ref|XP_001217691.1|/0/aryl-alcohol dehydrogenase [Aspergillus terreus NIH2624]

4354418	1461	15.68	160.08	3.775298944	8.76E-17	1.43E-15	Up	ATEG_09885	hypothetical protein	ko01100//Metabolic pathways;ko01110//Biosynthesis of secondary metabolites;ko01130//Biosynthesis of antibiotics;ko01230//Biosynthesis of amino acids;ko01200//Carbon metabolism;ko00350//Tyrosine metabolism;ko00360//Phenylalanine metabolism;ko00250//Alanine, aspartate and glutamate metabolism;ko00330//Arginine and proline metabolism;ko00270//Cysteine and methionine metabolism;ko01210//2-Oxocarboxylic acid metabolism;ko00400//Phenylalanine, tyrosine and tryptophan biosynthesis;ko00220//Arginine biosynthesis	-	GO:0004657//proline dehydrogenase activity	GO:0006562//proline catabolic process;GO:0055114//oxidation-reduction process	gi|115443400|ref|XP_001218507.1|/0/conserved hypothetical protein [Aspergillus terreus NIH2624]
4322958	1044	0	18.56	3.769346763	0.00010377	0.00051845	Up	ATEG_07453	hypothetical protein	ko00640//Propanoate metabolism	-	GO:0003824//catalytic activity	-	gi|115400972|ref|XP_001216074.1|/0/conserved hypothetical protein [Aspergillus terreus NIH2624]

4319148	2140	57.72	531.69	3.760054572	7.73E-33	2.87E-31	Up	ATEG_07254	hypothetical protein	ko00254//Aflatoxin biosynthesis	-	GO:0004497//monooxygenase activity;GO:0005506//iron ion binding;GO:0016705//oxidoreductase activity, acting on paired donors, with incorporation or reduction of molecular oxygen;GO:0020037//heme binding	GO:0055114//oxidation-reduction process	gi|115386798|ref|XP_001209940.1|/0/predicted protein [Aspergillus terreus NIH2624]
4323478	4439	218.07	1939.15	3.751349716	3.50E-56	2.83E-54	Up	ATEG_08616	xanthine dehydrogenase	ko01100//Metabolic pathways;ko01110//Biosynthesis of secondary metabolites;ko00230//Purine metabolism;ko04146//Peroxisome;ko00232//Caffeine metabolism	-	GO:0005506//iron ion binding;GO:0008762//UDP-N-acetylmuramate dehydrogenase activity;GO:0009055//electron carrier activity;GO:0050660//flavin adenine dinucleotide binding;GO:0051537//2 iron, 2 sulfur cluster binding	GO:0055114//oxidation-reduction process	gi|115402251|ref|XP_001217202.1|/0/xanthine dehydrogenase [Aspergillus terreus NIH2624]
4316795	3415	20.16	192.28	3.712115126	1.77E-19	3.51E-18	Up	ATEG_02400	similar to na/K ATPase alpha 1 isoform	ko00190//Oxidative phosphorylation	GO:0016021//integral component of membrane	GO:0005524//ATP binding;GO:0016787//hydrolase activity;GO:0046872//metal ion binding	-	gi|115388145|ref|XP_001211578.1|/0/hypothetical protein ATEG_02400 [Aspergillus terreus NIH2624]
4355494	1779	932	7898.73	3.705359078	5.74E-145	2.56E-142	Up	ATEG_00739	hypothetical protein	-	GO:0016021//integral component of membrane	GO:0015171//amino acid transmembrane transporter activity	GO:0003333//amino acid transmembrane transport	gi|115492395|ref|XP_001210825.1|/0/conserved hypothetical protein [Aspergillus terreus NIH2624]

4317542	696	9.52	104.18	3.694469899	1.01E-10	1.08E-09	Up	ATEG_02897	allergen Asp f 4	ko04144//Endocytosis	-	-	-	gi|115389140|ref|XP_001212075.1|/1.32486e-172/allergen Asp f 4 [Aspergillus terreus NIH2624]
4317423	1611	59.94	523.58	3.6775984	1.15E-39	5.58E-38	Up	ATEG_02947	hypothetical protein	ko01100//Metabolic pathways;ko01110//Biosynthesis of secondary metabolites;ko01130//Biosynthesis of antibiotics;ko00230//Purine metabolism	GO:0016021//integral component of membrane	-	GO:0055085//transmembrane transport	gi|115389240|ref|XP_001212125.1|/0/conserved hypothetical protein [Aspergillus terreus NIH2624]
4355493	1576	1281.39	10579.36	3.665670114	4.96E-129	1.66E-126	Up	ATEG_00738	hypothetical protein	ko01100//Metabolic pathways;ko01110//Biosynthesis of secondary metabolites	-	GO:0004497//monooxygenase activity;GO:0005506//iron ion binding;GO:0016705//oxidoreductase activity, acting on paired donors, with incorporation or reduction of molecular oxygen;GO:0020037//heme binding	GO:0055114//oxidation-reduction process	gi|115492393|ref|XP_001210824.1|/0/conserved hypothetical protein [Aspergillus terreus NIH2624]
4315665	885	118.25	991.71	3.664231479	1.22E-56	1.02E-54	Up	ATEG_01109	hypothetical protein	-	-	GO:0051213//dioxygenase activity	GO:0055114//oxidation-reduction process	gi|115383854|ref|XP_001208474.1|/0/conserved hypothetical protein [Aspergillus terreus NIH2624]
4353852	1497	216.88	1776.61	3.643996843	2.71E-79	3.91E-77	Up	ATEG_09335	hypothetical protein	-	GO:0016021//integral component of membrane	-	GO:0055085//transmembrane transport	gi|115438014|ref|XP_001217957.1|/0/conserved hypothetical protein [Aspergillus terreus NIH2624]

4355514	1296	95.36	978.25	3.638001425	2.28E-10	2.33E-09	Up	ATEG_00759	hypothetical protein	ko01100//Metabolic pathways;ko00380//Tryptophan metabolism	-	GO:0020037//heme binding;GO:0033754//indoleamine 2,3-dioxygenase activity	GO:0019441//tryptophan catabolic process to kynurenine;GO:0055114//oxidation-reduction process	gi|115492435|ref|XP_001210845.1|/0/conserved hypothetical protein [Aspergillus terreus NIH2624]
4316800	3092	17.35	162.28	3.636796833	5.67E-15	8.10E-14	Up	ATEG_02244	hypothetical protein	-	-	GO:0005524//ATP binding;GO:0008270//zinc ion binding	-	gi|115387833|ref|XP_001211422.1|/0/predicted protein [Aspergillus terreus NIH2624]
4354776	1494	639.62	5146.88	3.605672337	4.28E-72	5.66E-70	Up	ATEG_00020	betaine aldehyde dehydrogenase	ko01100//Metabolic pathways;ko00260//Glycine, serine and threonine metabolism	-	GO:0016620//oxidoreductase activity, acting on the aldehyde or oxo group of donors, NAD or NADP as acceptor	GO:0055114//oxidation-reduction process	gi|115490957|ref|XP_001210106.1|/0/betaine aldehyde dehydrogenase [Aspergillus terreus NIH2624]
4318122	2520	0.56	20.16	3.588169518	0.00010421	0.0005204	Up	ATEG_02805	hypothetical protein	ko03410//Base excision repair	GO:0005634//nucleus	GO:0000981//RNA polymerase II transcription factor activity, sequence-specific DNA binding;GO:0008270//zinc ion binding	GO:0006357//regulation of transcription from RNA polymerase II promoter	gi|115388956|ref|XP_001211983.1|/0/conserved hypothetical protein [Aspergillus terreus NIH2624]
4322350	861	959.71	7448.99	3.572643802	5.78E-87	9.68E-85	Up	ATEG_06438	esterase D	ko01200//Carbon metabolism;ko00680//Methane metabolism	GO:0005829//cytosol	GO:0018738//S-formylglutathione hydrolase activity;GO:0052689//carboxylic ester hydrolase activity	GO:0046294//formaldehyde catabolic process	gi|115400055|ref|XP_001215616.1|/0/esterase D [Aspergillus terreus NIH2624]
4321943	1251	0	16.91	3.571412659	0.00026415	0.00123188	Up	ATEG_06578	hypothetical protein	ko04111//Cell cycle - yeast	-	-	-	gi|115400335|ref|XP_001215756.1|/0/predicted protein [Aspergillus terreus NIH2624]

4354153	705	2.24	35.64	3.568186244	1.13E-05	6.65E-05	Up	ATEG_09894	endoglucanase I precursor	-	-	GO:0008810//cellulase activity;GO:0033946//xyloglucan-specific endo-beta-1,4-glucanase activity	GO:0009251//glucan catabolic process;GO:0010411//xyloglucan metabolic process	gi|115443418|ref|XP_001218516.1|/2.5728e-176/endoglucanase I precursor [Aspergillus terreus NIH2624]
4322366	1458	158.59	1218.48	3.547099281	1.05E-71	1.37E-69	Up	ATEG_06764	similar to arginase family protein	ko01100//Metabolic pathways;ko00330//Arginine and proline metabolism	-	GO:0016813//hydrolase activity, acting on carbon-nitrogen (but not peptide) bonds, in linear amidines;GO:0046872//metal ion binding	-	gi|115400707|ref|XP_001215942.1|/0/hypothetical protein ATEG_06764 [Aspergillus terreus NIH2624]
4323030	1350	101.99	787.79	3.543152992	8.96E-58	7.71E-56	Up	ATEG_07526	hypothetical protein	ko01100//Metabolic pathways;ko04146//Peroxisome;ko00260//Glycine, serine and threonine metabolism;ko00310//Lysine degradation	GO:0005576//extracellular region	GO:0016491//oxidoreductase activity	GO:0055114//oxidation-reduction process	gi|115401118|ref|XP_001216147.1|/0/conserved hypothetical protein [Aspergillus terreus NIH2624]
4321672	1351	926.43	7440.8	3.526932111	7.94E-21	1.70E-19	Up	ATEG_06182	aspergillopepsin F precursor	-	GO:0005576//extracellular region	GO:0004190//aspartic-type endopeptidase activity	GO:0006508//proteolysis;GO:0009405//pathogenesis	gi|115399542|ref|XP_001215360.1|/0/aspergillopepsin F precursor [Aspergillus terreus NIH2624]
4321618	1403	260.63	1963.13	3.522497318	5.89E-101	1.38E-98	Up	ATEG_06192	similar to arginase family protein	ko01100//Metabolic pathways;ko00330//Arginine and proline metabolism	-	GO:0008783//agmatinase activity;GO:0046872//metal ion binding	-	gi|115399562|ref|XP_001215370.1|/0/hypothetical protein ATEG_06192 [Aspergillus terreus NIH2624]
4316250	1629	162.56	1224.7	3.515694842	2.62E-57	2.23E-55	Up	ATEG_01874	sugar transporter STL1	-	GO:0016021//integral component of membrane	GO:0022891//substrate-specific transmembrane transporter activity	GO:0008643//carbohydrate transport;GO:0055085//transmembrane transport	gi|115385384|ref|XP_001209239.1|/0/sugar transporter STL1 [Aspergillus terreus NIH2624]

4317873	1827	1.12	24.54	3.514005421	7.81E-05	0.00039997	Up	ATEG_03069	hypothetical protein	-	GO:0016021//integral component of membrane	-	-	gi|115389484|ref|XP_001212247.1|/0/conserved hypothetical protein [Aspergillus terreus NIH2624]
4321801	1002	3288.2	24302.4	3.510679147	6.19E-141	2.53E-138	Up	ATEG_05818	similar to STI35 protein	ko01100//Metabolic pathways;ko00730//Thiamine metabolism	GO:0005634//nucleus;GO:0005829//cytosol	GO:0008198//ferrous iron binding;GO:0016491//oxidoreductase activity;GO:0042802//identical protein binding	GO:0000002//mitochondrial genome maintenance;GO:0006950//response to stress;GO:0009228//thiamine biosynthetic process;GO:0052837//thiazole biosynthetic process;GO:0055114//oxidation-reduction process	gi|115398814|ref|XP_001214996.1|/0/hypothetical protein ATEG_05818 [Aspergillus terreus NIH2624]
4320271	1759	999.97	7381.12	3.500029803	1.42E-138	5.13E-136	Up	ATEG_04823	protein EPD1 precursor	ko01100//Metabolic pathways;ko00500//Starch and sucrose metabolism	GO:0005886//plasma membrane;GO:0016021//integral component of membrane;GO:0031225//anchored component of membrane	GO:0016740//transferase activity	GO:0005975//carbohydrate metabolic process	gi|115396724|ref|XP_001214001.1|/0/protein EPD1 precursor [Aspergillus terreus NIH2624]
4317422	1071	27.47	221.02	3.491124818	1.27E-19	2.55E-18	Up	ATEG_02946	hypothetical protein	ko01100//Metabolic pathways;ko01212//Fatty acid metabolism;ko00062//Fatty acid elongation	-	GO:0008270//zinc ion binding;GO:0016491//oxidoreductase activity	GO:0055114//oxidation-reduction process	gi|115389238|ref|XP_001212124.1|/0/predicted protein [Aspergillus terreus NIH2624]
4315565	1149	133.97	979.75	3.459698596	6.58E-59	5.72E-57	Up	ATEG_01215	hypothetical protein	-	-	GO:0016491//oxidoreductase activity	GO:0055114//oxidation-reduction process	gi|115384066|ref|XP_001208580.1|/0/predicted protein [Aspergillus terreus NIH2624]

4321518	7794	57.15	422.97	3.452369166	2.85E-35	1.18E-33	Up	ATEG_06056	similar to polyketide synthase	-	-	GO:0016491//oxidoreductase activity;GO:0016740//transferase activity;GO:0031177//phosphopantetheine binding	GO:0055114//oxidation-reduction process	gi|115399290|ref|XP_001215234.1|/0/hypothetical protein ATEG_06056 [Aspergillus terreus NIH2624]
4319957	975	31.93	242.99	3.441529204	1.50E-21	3.34E-20	Up	ATEG_04344	hypothetical protein	-	-	GO:0004672//protein kinase activity;GO:0005524//ATP binding	GO:0006468//protein phosphorylation	gi|115395642|ref|XP_001213522.1|/0/predicted protein [Aspergillus terreus NIH2624]
4354069	4548	77.84	572.61	3.440133923	3.79E-30	1.30E-28	Up	ATEG_09271	hypothetical protein	-	-	-	-	gi|115437762|ref|XP_001217893.1|/2.37444e-163/predicted protein [Aspergillus terreus NIH2624]
4353077	1196	41.48	303	3.4145791	1.24E-24	3.26E-23	Up	ATEG_08179	hypothetical protein	ko01100//Metabolic pathways;ko00330//Arginine and proline metabolism	-	GO:0016813//hydrolase activity, acting on carbon-nitrogen (but not peptide) bonds, in linear amidines;GO:0046872//metal ion binding	-	gi|115433326|ref|XP_001216800.1|/0/conserved hypothetical protein [Aspergillus terreus NIH2624]
4321754	4332	1628.8	10920.24	3.368479963	1.19E-101	2.95E-99	Up	ATEG_05950	hypothetical protein	-	GO:0016021//integral component of membrane	GO:0035673//oligopeptide transmembrane transporter activity	GO:0055085//transmembrane transport	gi|115399078|ref|XP_001215128.1|/0/predicted protein [Aspergillus terreus NIH2624]

4319110	3810	594.08	3955.71	3.354917395	7.22E-79	1.03E-76	Up	ATEG_07313	hypothetical protein	-	GO:0016021//integral component of membrane	GO:0004497//monooxygenase activity;GO:0005506//iron ion binding;GO:0015171//amino acid transmembrane transporter activity;GO:0016705//oxidoreductase activity, acting on paired donors, with incorporation or reduction of molecular oxygen;GO:0020037//heme binding	GO:0003333//amino acid transmembrane transport;GO:0055114//oxidation-reduction process	gi|115386916|ref|XP_001209999.1|/0/conserved hypothetical protein [Aspergillus terreus NIH2624]
4323477	2163	397.9	2649.62	3.353520421	6.96E-84	1.09E-81	Up	ATEG_08615	hypothetical protein	-	-	GO:0004497//monooxygenase activity;GO:0071949//FAD binding	GO:0055114//oxidation-reduction process	gi|115402249|ref|XP_001217201.1|/0/conserved hypothetical protein [Aspergillus terreus NIH2624]
4316162	1620	1.68	26.62	3.350721372	0.00010478	0.0005224	Up	ATEG_01645	hypothetical protein	-	-	-	-	gi|115384926|ref|XP_001209010.1|/0/conserved hypothetical protein [Aspergillus terreus NIH2624]
4353365	1131	68.93	470.28	3.348720849	2.13E-36	9.36E-35	Up	ATEG_08246	hypothetical protein	ko00240//Pyrimidine metabolism;ko00760//Nicotinate and nicotinamide metabolism	-	-	-	gi|115433460|ref|XP_001216867.1|/0/predicted protein [Aspergillus terreus NIH2624]
4317540	315	100.87	678.22	3.341362901	2.25E-49	1.48E-47	Up	ATEG_02895	hypothetical protein	ko03013//RNA transport;ko03015//mRNA surveillance pathway	-	-	-	gi|115389136|ref|XP_001212073.1|/3.17351e-73/conserved hypothetical protein [Aspergillus terreus NIH2624]

4321586	1641	20.18	147.83	3.340047693	3.38E-14	4.64E-13	Up	ATEG_05674	hypothetical protein	ko00380//Tryptophan metabolism;ko00360//Phenylalanine metabolism;ko00330//Arginine and proline metabolism	-	GO:0004040//amidase activity;GO:0016884//carbon-nitrogen ligase activity, with glutamine as amido-N-donor	-	gi|115398526|ref|XP_001214852.1|/0/predicted protein [Aspergillus terreus NIH2624]
4354933	873	3.93	40.04	3.31415515	6.63E-06	4.05E-05	Up	ATEG_00176	hypothetical protein	-	-	-	-	gi|115491269|ref|XP_001210262.1|/0/predicted protein [Aspergillus terreus NIH2624]
4321790	1314	922.58	5954.06	3.312148323	9.23E-110	2.40E-107	Up	ATEG_05951	similar to fructosyl-amino acid oxidase	ko01100//Metabolic pathways;ko04146//Peroxisome;ko00260//Glycine, serine and threonine metabolism;ko00310//Lysine degradation	-	GO:0051700//fructosyl-amino acid oxidase activity	GO:0055114//oxidation-reduction process	gi|115399080|ref|XP_001215129.1|/0/hypothetical protein ATEG_05951 [Aspergillus terreus NIH2624]
4323320	963	2.8	34.54	3.309735391	4.03E-05	0.00021928	Up	ATEG_08638	hypothetical protein	ko01100//Metabolic pathways;ko00051//Fructose and mannose metabolism;ko00650//Butanoate metabolism;ko00591//Linoleic acid metabolism	-	GO:0016491//oxidoreductase activity	GO:0055114//oxidation-reduction process	gi|115402295|ref|XP_001217224.1|/0/predicted protein [Aspergillus terreus NIH2624]
4321824	2456	1731.36	11123.3	3.306861052	2.91E-120	9.11E-118	Up	ATEG_05817	hypothetical protein	ko01100//Metabolic pathways;ko00600//Sphingolipid metabolism	GO:0005773//vacuole;GO:0005886//plasma membrane;GO:0016021//integral component of membrane	-	GO:0051278//fungal-type cell wall polysaccharide biosynthetic process	gi|115398812|ref|XP_001214995.1|/0/conserved hypothetical protein [Aspergillus terreus NIH2624]

4354944	1022	436.02	2799.22	3.301598264	8.01E-82	1.19E-79	Up	ATEG_00195	similar to aldehyde reductase	ko01100//Metabolic pathways;ko00561//Glycerolipid metabolism	GO:0005576//extracellular region;GO:0005622//intracellular	GO:0016491//oxidoreductase activity	GO:0055114//oxidation-reduction process	gi|115491307|ref|XP_001210281.1|/0/hypothetical protein ATEG_00195 [Aspergillus terreus NIH2624]
4318509	797	142.29	930.1	3.299911371	9.65E-51	6.56E-49	Up	ATEG_04244	hypothetical protein	-	-	-	-	gi|115391835|ref|XP_001213422.1|/1.30361e-87/predicted protein [Aspergillus terreus NIH2624]
4355683	909	0	13.69	3.292902386	0.00088615	0.00365453	Up	ATEG_00920	hypothetical protein	-	-	-	-	gi|115492757|ref|XP_001211006.1|/0/predicted protein [Aspergillus terreus NIH2624]
4323393	1041	197.82	1269.67	3.291698946	2.12E-59	1.88E-57	Up	ATEG_08619	hypothetical protein	ko01100//Metabolic pathways;ko01130//Biosynthesis of antibiotics;ko00311//Penicillin and cephalosporin biosynthesis	-	GO:0005506//iron ion binding;GO:0016491//oxidoreductase activity	GO:0055114//oxidation-reduction process	gi|115402257|ref|XP_001217205.1|/0/conserved hypothetical protein [Aspergillus terreus NIH2624]
4318205	732	5.6	53.16	3.289147397	2.44E-06	1.61E-05	Up	ATEG_03711	hypothetical protein	-	GO:0005743//mitochondrial inner membrane	GO:0048039//ubiquinone binding	GO:0006744//ubiquinone biosynthetic process;GO:0045333//cellular respiration	gi|115390769|ref|XP_001212889.1|/2.09814e-175/conserved hypothetical protein [Aspergillus terreus NIH2624]
4316052	714	103.07	694.43	3.288244827	4.17E-28	1.30E-26	Up	ATEG_01232	hypothetical protein	-	-	GO:0036361//racemase activity, acting on amino acids and derivatives	GO:0006520//cellular amino acid metabolic process	gi|115384100|ref|XP_001208597.1|/1.94272e-168/predicted protein [Aspergillus terreus NIH2624]

4353980	2424	0	13.28	3.284590353	0.00091057	0.00374567	Up	ATEG_09310	hypothetical protein	-	GO:0005634//nucleus	GO:0000981//RNA polymerase II transcription factor activity, sequence-specific DNA binding;GO:0003677//DNA binding;GO:0008270//zinc ion binding	GO:0006357//regulation of transcription from RNA polymerase II promoter	gi|115437914|ref|XP_001217932.1|/0/predicted protein [Aspergillus terreus NIH2624]
4322491	660	593.08	3751.34	3.273958232	1.94E-93	3.87E-91	Up	ATEG_07572	hypothetical protein	-	-	GO:0016491//oxidoreductase activity	GO:0055114//oxidation-reduction process	gi|115401210|ref|XP_001216193.1|/1.08801e-156/conserved hypothetical protein [Aspergillus terreus NIH2624]
4355478	2907	156.95	999.46	3.273818054	3.30E-48	2.15E-46	Up	ATEG_00723	alpha-glucosidase precursor	ko01100//Metabolic pathways;ko00500//Starch and sucrose metabolism;ko00052//Galactose metabolism	-	GO:0004553//hydrolase activity, hydrolyzing O-glycosyl compounds;GO:0030246//carbohydrate binding	GO:0005975//carbohydrate metabolic process	gi|115492363|ref|XP_001210809.1|/0/alpha-glucosidase precursor [Aspergillus terreus NIH2624]
4318976	954	288.14	1825.71	3.273717933	2.57E-68	2.98E-66	Up	ATEG_07060	hypothetical protein	ko01100//Metabolic pathways;ko01110//Biosynthesis of secondary metabolites	-	GO:0005506//iron ion binding;GO:0016708//oxidoreductase activity, acting on paired donors, with incorporation or reduction of molecular oxygen, NAD(P)H as one donor, and incorporation of two atoms of oxygen into one donor;GO:0051537//2 iron, 2 sulfur cluster binding	GO:0019439//aromatic compound catabolic process;GO:0055114//oxidation-reduction process	gi|115386410|ref|XP_001209746.1|/0/conserved hypothetical protein [Aspergillus terreus NIH2624]

4353073	735	4.49	1063.99	3.27300953	0.00109415	0.00443472	Up	ATEG_08175	hypothetical protein	-	GO:0016021//integral component of membrane	-	-	gi|115433318|ref|XP_001216796.1|/6.2378e-173/predicted protein [Aspergillus terreus NIH2624]
4315511	1482	0	12.5	3.269187483	0.00095447	0.00391293	Up	ATEG_01021	hypothetical protein	-	GO:0016021//integral component of membrane	-	GO:0055085//transmembrane transport	gi|115383678|ref|XP_001208386.1|/0/conserved hypothetical protein [Aspergillus terreus NIH2624]
4316878	984	33.62	228.45	3.261117848	3.16E-18	5.62E-17	Up	ATEG_01898	hypothetical protein	-	-	GO:0016491//oxidoreductase activity	GO:0008152//metabolic process	gi|115387141|ref|XP_001211076.1|/0/conserved hypothetical protein [Aspergillus terreus NIH2624]
4318701	1074	74.51	479.02	3.25936115	1.61E-28	5.10E-27	Up	ATEG_03722	maleylacetate reductase	-	-	GO:0018506;GO:0046872//metal ion binding	GO:0055114//oxidation-reduction process	gi|115390791|ref|XP_001212900.1|/0/maleylacetate reductase [Aspergillus terreus NIH2624]
4354027	1803	105.4	671.22	3.254813584	1.94E-31	6.96E-30	Up	ATEG_09061	similar to N-carbamyl-D-amino acid amidohydrolase	ko01100//Metabolic pathways;ko00240//Pyrimidine metabolism;ko00770//Pantothenate and CoA biosynthesis;ko00410//beta-Alanine metabolism	-	GO:0016810//hydrolase activity, acting on carbon-nitrogen (but not peptide) bonds;GO:0036361//racemase activity, acting on amino acids and derivatives	GO:0006807//nitrogen compound metabolic process	gi|115436938|ref|XP_001217683.1|/0/hypothetical protein ATEG_09061 [Aspergillus terreus NIH2624]
4319551	1608	245.46	1546.44	3.250539488	8.03E-66	8.66E-64	Up	ATEG_10068	hypothetical protein	-	GO:0016021//integral component of membrane	-	GO:0055085//transmembrane transport	gi|115385647|ref|XP_001209370.1|/0/conserved hypothetical protein [Aspergillus terreus NIH2624]

4320683	864	83.53	529.8	3.247468749	5.43E-32	1.97E-30	Up	ATEG_05329	hypothetical protein	-	-	GO:0016491//oxidoreductase activity	GO:0055114//oxidation-reduction process	gi|115397831|ref|XP_001214507.1|/0/conserved hypothetical protein [Aspergillus terreus NIH2624]
4321147	1095	21.27	150.21	3.242169793	2.86E-11	3.21E-10	Up	ATEG_05005	carboxy-cis,cis-muconate cyclase	-	-	-	-	gi|115397183|ref|XP_001214183.1|/0/carboxy-cis,cis-muconate cyclase [Aspergillus terreus NIH2624]
4353249	2548	30.26	199.83	3.241932693	3.97E-18	7.01E-17	Up	ATEG_08215	hypothetical protein	-	-	GO:0016491//oxidoreductase activity	GO:0055114//oxidation-reduction process	gi|115433398|ref|XP_001216836.1|/0/predicted protein [Aspergillus terreus NIH2624]
4323394	1996	245.96	1504.4	3.222254415	5.35E-56	4.25E-54	Up	ATEG_08620	hypothetical protein	-	GO:0016021//integral component of membrane	-	GO:0055085//transmembrane transport	gi|115402259|ref|XP_001217206.1|/0/conserved hypothetical protein [Aspergillus terreus NIH2624]

4353112	1504	10371.42	61796.16	3.200221396	1.62E-97	3.62E-95	Up	ATEG_08139	S-(hydroxymethyl)glutathione dehydrogenase	ko01100//Metabolic pathways;ko01110//Biosynthesis of secondary metabolites;ko01130//Biosynthesis of antibiotics;ko01200//Carbon metabolism;ko01220//Degradation of aromatic compounds;ko00350//Tyrosine metabolism;ko00010//Glycolysis / Gluconeogenesis;ko00071//Fatty acid degradation;ko00680//Methane metabolism	GO:0005634//nucleus;GO:0005829//cytosol	GO:0008270//zinc ion binding;GO:0051903//S-(hydroxymethyl)glutathione dehydrogenase activity	GO:0006069//ethanol oxidation	gi|115433246|ref|XP_001216760.1|/0/S-(hydroxymethyl)glutathione dehydrogenase [Aspergillus terreus NIH2624]
4316704	465	491.51	2933.33	3.197367792	4.92E-75	6.59E-73	Up	ATEG_02276	hypothetical protein	ko00910//Nitrogen metabolism	GO:0005576//extracellular region	GO:0003677//DNA binding;GO:0008824//cyanate hydratase activity	GO:0009439//cyanate metabolic process	gi|115387897|ref|XP_001211454.1|/3.79359e-107/conserved hypothetical protein [Aspergillus terreus NIH2624]

4320613	1380	833.98	4963.6	3.196260179	7.81E-89	1.44E-86	Up	ATEG_05420	hypothetical protein	ko01100//Metabolic pathways;ko01110//Biosynthesis of secondary metabolites;ko01130//Biosynthesis of antibiotics;ko01230//Biosynthesis of amino acids;ko01200//Carbon metabolism;ko00260//Glycine, serine and threonine metabolism;ko00270//Cysteine and methionine metabolism;ko00290//Valine, leucine and isoleucine biosynthesis	-	GO:0030170//pyridoxal phosphate binding	GO:0006520//cellular amino acid metabolic process	gi|115398013|ref|XP_001214598.1|/0/conserved hypothetical protein [Aspergillus terreus NIH2624]
4353580	2409	12.9	91.25	3.191268639	5.08E-09	4.59E-08	Up	ATEG_08204	hypothetical protein	-	-	GO:0000287//magnesium ion binding;GO:0010333//terpene synthase activity	GO:0009116//nucleoside metabolic process	gi|115433376|ref|XP_001216825.1|/0/conserved hypothetical protein [Aspergillus terreus NIH2624]
4354028	1506	160.86	968.48	3.184625437	5.19E-31	1.81E-29	Up	ATEG_09062	hypothetical protein	ko01100//Metabolic pathways;ko00240//Pyrimidine metabolism;ko00770//Pantothenate and CoA biosynthesis;ko00410//beta-Alanine metabolism	GO:0005737//cytoplasm	GO:0016810//hydrolase activity, acting on carbon-nitrogen (but not peptide) bonds	-	gi|115436942|ref|XP_001217684.1|/0/conserved hypothetical protein [Aspergillus terreus NIH2624]

4353691	486	154.64	933.44	3.174495263	1.41E-44	8.08E-43	Up	ATEG_09070	hypothetical protein	ko00310//Lysine degradation	-	-	-	gi|115436970|ref|XP_001217692.1|/9.18603e-116/predicted protein [Aspergillus terreus NIH2624]
4322638	2611	117.71	703.35	3.171774933	1.52E-32	5.60E-31	Up	ATEG_07553	hypothetical protein	-	GO:0016021//integral component of membrane	-	GO:0055085//transmembrane transport	gi|115401172|ref|XP_001216174.1|/0/predicted protein [Aspergillus terreus NIH2624]
4323185	1614	117.69	694.44	3.156265262	6.81E-35	2.75E-33	Up	ATEG_08670	hypothetical protein	ko01100//Metabolic pathways;ko00561//Glycerolipid metabolism	-	GO:0016787//hydrolase activity	-	gi|115402359|ref|XP_001217256.1|/0/conserved hypothetical protein [Aspergillus terreus NIH2624]
4322687	2661	139.55	810.75	3.140238093	3.10E-51	2.15E-49	Up	ATEG_07532	hypothetical protein	ko01100//Metabolic pathways;ko01110//Biosynthesis of secondary metabolites;ko00350//Tyrosine metabolism;ko00380//Tryptophan metabolism;ko00360//Phenylalanine metabolism;ko00340//Histidine metabolism	-	GO:0003854//3-beta-hydroxy-delta5-steroid dehydrogenase activity;GO:0016831//carboxy-lyase activity;GO:0030170//pyridoxal phosphate binding;GO:0050662//coenzyme binding	GO:0006520//cellular amino acid metabolic process;GO:0006694//steroid biosynthetic process;GO:0055114//oxidation-reduction process	gi|115401130|ref|XP_001216153.1|/0/conserved hypothetical protein [Aspergillus terreus NIH2624]

4315896	1503	1685.2	10466.31	3.13774901	4.09E-15	5.90E-14	Up	ATEG_01487	catalase	ko01110//Biosynthesis of secondary metabolites;ko01130//Biosynthesis of antibiotics;ko01200//Carbon metabolism;ko04011//MAPK signaling pathway - yeast;ko04146//Peroxisome;ko00380//Tryptophan metabolism;ko00630//Glyoxylate and dicarboxylate metabolism	GO:0005759//mitochondrial matrix;GO:0005782//peroxisomal matrix	GO:0004096//catalase activity;GO:0020037//heme binding;GO:0046872//metal ion binding	GO:0001315//age-dependent response to reactive oxygen species;GO:0042744//hydrogen peroxide catabolic process;GO:0055114//oxidation-reduction process;GO:0098869//cellular oxidant detoxification	gi|115384610|ref|XP_001208852.1|/0/catalase [Aspergillus terreus NIH2624]
4353985	2361	188.3	1071.78	3.117973104	2.08E-59	1.86E-57	Up	ATEG_09118	hypothetical protein	-	-	-	-	gi|115437158|ref|XP_001217740.1|/0/predicted protein [Aspergillus terreus NIH2624]
4321445	1626	462.4	2600.85	3.109777549	4.24E-56	3.40E-54	Up	ATEG_05712	hypothetical protein	-	GO:0016021//integral component of membrane	-	GO:0055085//transmembrane transport	gi|115398602|ref|XP_001214890.1|/0/conserved hypothetical protein [Aspergillus terreus NIH2624]
4323188	537	0	14.72	3.107847143	0.00184276	0.00708631	Up	ATEG_08673	hypothetical protein	ko01100//Metabolic pathways;ko01110//Biosynthesis of secondary metabolites;ko01130//Biosynthesis of antibiotics;ko00100//Steroid biosynthesis;ko00909//Sesquiterpenoid and triterpenoid biosynthesis	-	-	-	gi|115402365|ref|XP_001217259.1|/2.76952e-132/conserved hypothetical protein [Aspergillus terreus NIH2624]

4354024	1059	1.12	17.97	3.100263799	0.00067763	0.00288093	Up	ATEG_09058	hypothetical protein	ko01100//Metabolic pathways;ko04146//Peroxisome;ko00590//Arachidonic acid metabolism	-	GO:0003824//catalytic activity	-	gi|115436926|ref|XP_001217680.1|/0/predicted protein [Aspergillus terreus NIH2624]
4321334	1345	36.43	217.74	3.093368146	2.22E-17	3.74E-16	Up	ATEG_06013	hypothetical protein	ko03018//RNA degradation	-	GO:0008168//methyltransferase activity	GO:0032259//methylation	gi|115399204|ref|XP_001215191.1|/0/predicted protein [Aspergillus terreus NIH2624]
4353298	6459	16.27	103.98	3.081775836	1.30E-09	1.24E-08	Up	ATEG_08427	similar to peptide synthetase	-	-	GO:0003824//catalytic activity;GO:0031177//phosphopantetheine binding	GO:0019184//nonribosomal peptide biosynthetic process;GO:2001310//gliotoxin biosynthetic process	gi|115433823|ref|XP_001217048.1|/0/hypothetical protein ATEG_08427 [Aspergillus terreus NIH2624]
4322537	561	72.32	421.83	3.074009446	4.98E-21	1.09E-19	Up	ATEG_07484	similar to PhiA protein	ko04144//Endocytosis	GO:0005576//extracellular region;GO:0005618//cell wall	-	GO:0000905//sporocarp development involved in asexual reproduction;GO:0043935//sexual sporulation resulting in formation of a cellular spore;GO:0070790	gi|115401034|ref|XP_001216105.1|/3.20834e-135/hypothetical protein ATEG_07484 [Aspergillus terreus NIH2624]
4323273	1932	154.14	854.08	3.073714294	6.19E-49	4.06E-47	Up	ATEG_08604	hypothetical protein	ko04113//Meiosis - yeast	GO:0016021//integral component of membrane	GO:0022891//substrate-specific transmembrane transporter activity	GO:0008643//carbohydrate transport;GO:0055085//transmembrane transport	gi|115402227|ref|XP_001217190.1|/0/conserved hypothetical protein [Aspergillus terreus NIH2624]
4316450	1443	8.96	62.86	3.071094626	4.51E-07	3.29E-06	Up	ATEG_01912	hypothetical protein	-	GO:0016021//integral component of membrane	-	-	gi|115387169|ref|XP_001211090.1|/0/conserved hypothetical protein [Aspergillus terreus NIH2624]

4317868	831	19.62	120.31	3.068991798	1.43E-10	1.50E-09	Up	ATEG_03379	hypothetical protein	ko00051//Fructose and mannose metabolism	-	GO:0016491//oxidoreductase activity	GO:0055114//oxidation-reduction process	gi|115390104|ref|XP_001212557.1|/0/conserved hypothetical protein [Aspergillus terreus NIH2624]
4321146	972	48.19	272.44	3.053285394	1.43E-19	2.85E-18	Up	ATEG_05004	hypothetical protein	ko01100//Metabolic pathways;ko01220//Degradation of aromatic compounds	-	GO:0008199//ferric iron binding;GO:0018576//catechol 1,2-dioxygenase activity	GO:0009712//catechol-containing compound metabolic process;GO:0055114//oxidation-reduction process	gi|115397181|ref|XP_001214182.1|/0/conserved hypothetical protein [Aspergillus terreus NIH2624]
4315666	1588	71.19	385.47	3.00677697	1.70E-20	3.58E-19	Up	ATEG_01110	hypothetical protein	-	GO:0016021//integral component of membrane	GO:0022891//substrate-specific transmembrane transporter activity	GO:0055085//transmembrane transport	gi|115383856|ref|XP_001208475.1|/0/conserved hypothetical protein [Aspergillus terreus NIH2624]
4316269	1578	349.62	1827.36	3.001045001	1.20E-50	8.07E-49	Up	ATEG_01757	hypothetical protein	ko04113//Meiosis - yeast	GO:0016021//integral component of membrane	GO:0022891//substrate-specific transmembrane transporter activity	GO:0008643//carbohydrate transport;GO:0055085//transmembrane transport	gi|115385150|ref|XP_001209122.1|/0/conserved hypothetical protein [Aspergillus terreus NIH2624]
4318095	2232	80.7	430.58	3.000727088	4.06E-29	1.30E-27	Up	ATEG_03318	similar to kinesin	ko04141//Protein processing in endoplasmic reticulum	GO:0005874//microtubule	GO:0003777//microtubule motor activity;GO:0005524//ATP binding;GO:0008017//microtubule binding	GO:0007018//microtubule-based movement	gi|115389982|ref|XP_001212496.1|/0/hypothetical protein ATEG_03318 [Aspergillus terreus NIH2624]
4320417	1779	0	10.68	2.996527875	0.0026959	0.00993544	Up	ATEG_04597	hypothetical protein	ko04144//Endocytosis	-	GO:0016491//oxidoreductase activity	GO:0055114//oxidation-reduction process	gi|115396272|ref|XP_001213775.1|/0/conserved hypothetical protein [Aspergillus terreus NIH2624]

4318123	2274	2.24	22.9	2.990767502	0.00042044	0.00188035	Up	ATEG_02806	similar to beta-glucosidase precursor	ko01100//Metabolic pathways;ko01110//Biosynthesis of secondary metabolites;ko00500//Starch and sucrose metabolism;ko00460//Cyanoamino acid metabolism	-	GO:0008422//beta-glucosidase activity	GO:0030245//cellulose catabolic process	gi|115388958|ref|XP_001211984.1|/0/hypothetical protein ATEG_02806 [Aspergillus terreus NIH2624]
4317053	489	33.61	190.11	2.984617413	1.48E-13	1.94E-12	Up	ATEG_02167	hypothetical protein	-	GO:0016021//integral component of membrane	-	-	gi|115387679|ref|XP_001211345.1|/6.70582e-114/conserved hypothetical protein [Aspergillus terreus NIH2624]
4317252	2172	11.76	73.99	2.984284472	3.10E-07	2.32E-06	Up	ATEG_02664	hypothetical protein	ko01100//Metabolic pathways;ko00500//Starch and sucrose metabolism	-	-	-	gi|115388673|ref|XP_001211842.1|/0/predicted protein [Aspergillus terreus NIH2624]
4321089	2996	20.17	115.82	2.97553434	9.13E-11	9.78E-10	Up	ATEG_05209	hypothetical protein	ko01100//Metabolic pathways;ko00052//Galactose metabolism	GO:0005634//nucleus	GO:0000981//RNA polymerase II transcription factor activity, sequence-specific DNA binding;GO:0003677//DNA binding;GO:0008270//zinc ion binding	GO:0006357//regulation of transcription from RNA polymerase II promoter	gi|115397591|ref|XP_001214387.1|/0/predicted protein [Aspergillus terreus NIH2624]
4355585	3364	75.65	396.08	2.970038951	4.17E-27	1.26E-25	Up	ATEG_00824	hypothetical protein	ko01100//Metabolic pathways;ko04141//Protein processing in endoplasmic reticulum;ko00510//N-Glycan biosynthesis;ko00513//Various types of N-glycan biosynthesis	-	-	-	gi|115492565|ref|XP_001210910.1|/0/predicted protein [Aspergillus terreus NIH2624]

4316608	1035	54.96	296.98	2.965887172	9.18E-17	1.49E-15	Up	ATEG_02470	hypothetical protein	ko01100//Metabolic pathways;ko00513//Various types of N-glycan biosynthesis	-	GO:0016630//protochlorophyllide reductase activity;GO:0016740//transferase activity	GO:0055114//oxidation-reduction process	gi|115388285|ref|XP_001211648.1|/0/conserved hypothetical protein [Aspergillus terreus NIH2624]
4320646	609	21.86	123.38	2.964521897	3.09E-10	3.11E-09	Up	ATEG_05317	hypothetical protein	-	GO:0016021//integral component of membrane	-	-	gi|115397807|ref|XP_001214495.1|/2.40566e-147/conserved hypothetical protein [Aspergillus terreus NIH2624]
4318808	1425	89.08	464.22	2.962416623	9.84E-23	2.33E-21	Up	ATEG_04134	hypothetical protein	ko01100//Metabolic pathways;ko01130//Biosynthesis of antibiotics;ko00311//Penicillin and cephalosporin biosynthesis	-	GO:0016746//transferase activity, transferring acyl groups	-	gi|115391615|ref|XP_001213312.1|/0/predicted protein [Aspergillus terreus NIH2624]
4353405	1071	20.16	115.13	2.95577201	1.70E-09	1.60E-08	Up	ATEG_08069	thymidylate kinase	ko01100//Metabolic pathways;ko00240//Pyrimidine metabolism	-	GO:0004798//thymidylate kinase activity;GO:0005524//ATP binding	GO:0006233//dTDP biosynthetic process;GO:0046939//nucleotide phosphorylation	gi|115433106|ref|XP_001216690.1|/7.98214e-126/thymidylate kinase [Aspergillus terreus NIH2624]
4354023	1074	2.8	25.97	2.952640132	0.00038883	0.00175079	Up	ATEG_09057	hypothetical protein	-	GO:0016021//integral component of membrane	-	-	gi|115436922|ref|XP_001217679.1|/0/predicted protein [Aspergillus terreus NIH2624]
4316339	2517	31.35	177.83	2.948167762	2.41E-10	2.46E-09	Up	ATEG_01158	hypothetical protein	ko03013//RNA transport;ko03040//Spliceosome	GO:0005829//cytosol;GO:0031932//TORC2 complex	GO:0016301//kinase activity	GO:0016310//phosphorylation;GO:0045931//positive regulation of mitotic cell cycle	gi|115383952|ref|XP_001208523.1|/0/conserved hypothetical protein [Aspergillus terreus NIH2624]

4354025	1380	7.29	48.23	2.940853902	6.96E-06	4.23E-05	Up	ATEG_09059	hypothetical protein	ko01100//Metabolic pathways;ko01110//Biosynthesis of secondary metabolites;ko00403//Indole diterpene alkaloid biosynthesis	GO:0016021//integral component of membrane	GO:0016491//oxidoreductase activity;GO:0071949//FAD binding	GO:0055114//oxidation-reduction process	gi|115436930|ref|XP_001217681.1|/0/predicted protein [Aspergillus terreus NIH2624]
4319416	1892	57.2	298.2	2.936661173	1.11E-17	1.91E-16	Up	ATEG_10011	hypothetical protein	-	GO:0005634//nucleus	GO:0000981//RNA polymerase II transcription factor activity, sequence-specific DNA binding;GO:0003677//DNA binding;GO:0008270//zinc ion binding	GO:0006357//regulation of transcription from RNA polymerase II promoter	gi|115385533|ref|XP_001209313.1|/0/predicted protein [Aspergillus terreus NIH2624]
4355568	491	24.09	130.88	2.913928498	2.46E-11	2.77E-10	Up	ATEG_00807	hypothetical protein	-	GO:0016021//integral component of membrane	-	-	gi|115492531|ref|XP_001210893.1|/1.05522e-72/predicted protein [Aspergillus terreus NIH2624]
4355562	1980	26.33	139.63	2.902175506	9.29E-12	1.08E-10	Up	ATEG_00801	hypothetical protein	ko01100//Metabolic pathways;ko00260//Glycine, serine and threonine metabolism	-	GO:0016614//oxidoreductase activity, acting on CH-OH group of donors;GO:0050660//flavin adenine dinucleotide binding	GO:0055114//oxidation-reduction process	gi|115492519|ref|XP_001210887.1|/0/conserved hypothetical protein [Aspergillus terreus NIH2624]
4320954	840	3.92	30.8	2.895045189	0.00020334	0.00096899	Up	ATEG_05015	similar to arsH protein	-	-	GO:0016491//oxidoreductase activity	GO:0055114//oxidation-reduction process	gi|115397203|ref|XP_001214193.1|/0/hypothetical protein ATEG_05015 [Aspergillus terreus NIH2624]

4316699	1317	1460.1	7498.29	2.866160872	9.31E-12	1.09E-10	Up	ATEG_02283	similar to sarcosine oxidase	ko01100//Metabolic pathways;ko04146//Peroxisome;ko00260//Glycine, serine and threonine metabolism;ko00310//Lysine degradation	GO:0016021//integral component of membrane	GO:0016491//oxidoreductase activity	GO:0055114//oxidation-reduction process	gi|115387911|ref|XP_001211461.1|/0/hypothetical protein ATEG_02283 [Aspergillus terreus NIH2624]
4353863	3099	0.56	13.13	2.864130464	0.00319262	0.01150397	Up	ATEG_09142	hypothetical protein	-	-	GO:0003824//catalytic activity;GO:0031177//phosphopantetheine binding	GO:0008152//metabolic process	gi|115437254|ref|XP_001217764.1|/0/conserved hypothetical protein [Aspergillus terreus NIH2624]
4321385	1413	146.87	704.71	2.863561253	4.13E-31	1.45E-29	Up	ATEG_06166	hypothetical protein	-	GO:0016021//integral component of membrane	-	GO:0055085//transmembrane transport	gi|115399510|ref|XP_001215344.1|/0/conserved hypothetical protein [Aspergillus terreus NIH2624]
4354066	2177	24.69	138.82	2.863242135	1.00E-07	7.94E-07	Up	ATEG_09115	hypothetical protein	-	-	-	-	gi|115437146|ref|XP_001217737.1|/0/predicted protein [Aspergillus terreus NIH2624]
4315994	360	3.92	29.15	2.852929908	0.00025184	0.00117915	Up	ATEG_01690	hypothetical protein	ko01100//Metabolic pathways;ko00520//Amino sugar and nucleotide sugar metabolism	-	-	-	gi|115385016|ref|XP_001209055.1|/1.72002e-84/predicted protein [Aspergillus terreus NIH2624]
4323040	1372	35.32	176.51	2.84926945	1.03E-13	1.36E-12	Up	ATEG_07712	hypothetical protein	-	GO:0016021//integral component of membrane	-	-	gi|115401490|ref|XP_001216333.1|/0/conserved hypothetical protein [Aspergillus terreus NIH2624]

4321459	1296	150.78	715.06	2.847776826	2.17E-29	7.04E-28	Up	ATEG_05924	fumarylacetoacetase	ko01100//Metabolic pathways;ko00350//Tyrosine metabolism	-	GO:0004334//fumarylacetoacetase activity	GO:0009072//aromatic amino acid family metabolic process	gi|115399026|ref|XP_001215102.1|/0/fumarylacetoacetase [Aspergillus terreus NIH2624]
4316705	1464	16.27	89.18	2.83692154	4.03E-07	2.96E-06	Up	ATEG_02277	hypothetical protein	-	GO:0016021//integral component of membrane	GO:0005215//transporter activity	GO:0055085//transmembrane transport	gi|115387899|ref|XP_001211455.1|/0/conserved hypothetical protein [Aspergillus terreus NIH2624]
4319156	1659	967.43	4461.77	2.829757695	1.35E-64	1.39E-62	Up	ATEG_07053	similar to fructose transporter 1	-	GO:0016021//integral component of membrane	GO:0022891//substrate-specific transmembrane transporter activity	GO:0008643//carbohydrate transport;GO:0055085//transmembrane transport	gi|115386396|ref|XP_001209739.1|/0/hypothetical protein ATEG_07053 [Aspergillus terreus NIH2624]
4319445	4086	398.36	1845.85	2.829588479	1.21E-52	8.90E-51	Up	ATEG_10118	chitin synthase A	ko00520//Amino sugar and nucleotide sugar metabolism	GO:0016021//integral component of membrane;GO:0030428//cell septum	GO:0004100//chitin synthase activity	GO:0006031//chitin biosynthetic process;GO:0030448//hyphal growth;GO:0031505//fungal-type cell wall organization;GO:0043936//asexual sporulation resulting in formation of a cellular spore;GO:0048315//conidium formation;GO:0090529	gi|115385747|ref|XP_001209420.1|/0/chitin synthase A [Aspergillus terreus NIH2624]
4353724	1242	637.74	2923.5	2.820327882	1.85E-62	1.77E-60	Up	ATEG_09408	hypothetical protein	-	GO:0016021//integral component of membrane	GO:0015171//amino acid transmembrane transporter activity	GO:0003333//amino acid transmembrane transport	gi|115438296|ref|XP_001218030.1|/0/conserved hypothetical protein [Aspergillus terreus NIH2624]

4316980	1158	5.59	41.51	2.811541348	0.00045984	0.00203717	Up	ATEG_02087	hypothetical protein	-	GO:0016021//integral component of membrane	-	GO:0006810//transport	gi|115387519|ref|XP_001211265.1|/0/conserved hypothetical protein [Aspergillus terreus NIH2624]
4355061	1422	7.28	44.56	2.807162517	3.60E-05	0.0001973	Up	ATEG_00309	hypothetical protein	-	-	-	-	gi|115491535|ref|XP_001210395.1|/0/predicted protein [Aspergillus terreus NIH2624]
4316245	1611	117.68	543.88	2.805864239	6.36E-27	1.90E-25	Up	ATEG_01344	hypothetical protein	ko04146//Peroxisome	-	GO:0000287//magnesium ion binding;GO:0003824//catalytic activity;GO:0030976//thiamine pyrophosphate binding	-	gi|115384324|ref|XP_001208709.1|/0/conserved hypothetical protein [Aspergillus terreus NIH2624]
4353515	1776	84.09	393.05	2.797321405	4.74E-23	1.15E-21	Up	ATEG_08295	similar to glucose oxidase	ko01100//Metabolic pathways;ko00260//Glycine, serine and threonine metabolism	-	GO:0016614//oxidoreductase activity, acting on CH-OH group of donors;GO:0050660//flavin adenine dinucleotide binding	GO:0055114//oxidation-reduction process	gi|115433558|ref|XP_001216916.1|/0/hypothetical protein ATEG_08295 [Aspergillus terreus NIH2624]
4323153	1930	12.32	66.59	2.793346922	9.38E-07	6.47E-06	Up	ATEG_08877	hypothetical protein	-	-	-	-	gi|115402773|ref|XP_001217463.1|/0/predicted protein [Aspergillus terreus NIH2624]
4322100	1389	2650.52	11786.48	2.779519991	2.08E-112	5.74E-110	Up	ATEG_06276	hypothetical protein	-	GO:0016021//integral component of membrane	-	-	gi|115399716|ref|XP_001215454.1|/0/predicted protein [Aspergillus terreus NIH2624]

4321706	660	104.28	476.3	2.774197721	4.43E-25	1.20E-23	Up	ATEG_05708	hypothetical protein	ko01100//Metabolic pathways;ko00500//Starch and sucrose metabolism	-	GO:0003676//nucleic acid binding;GO:0046872//metal ion binding	-	gi|115398594|ref|XP_001214886.1|/5.63822e-168/predicted protein [Aspergillus terreus NIH2624]
4316051	2094	41.5	196.76	2.771632907	1.45E-12	1.80E-11	Up	ATEG_01231	hypothetical protein	-	GO:0016021//integral component of membrane	-	-	gi|115384098|ref|XP_001208596.1|/2.86894e-169/predicted protein [Aspergillus terreus NIH2624]
4316796	1622	394.01	1734.47	2.755489628	3.08E-64	3.11E-62	Up	ATEG_02401	hypothetical protein	-	GO:0016021//integral component of membrane	GO:0015299//solute:proton antiporter activity	GO:1902600//hydrogen ion transmembrane transport	gi|115388147|ref|XP_001211579.1|/0/predicted protein [Aspergillus terreus NIH2624]
4320042	1287	0	9.04	2.729475246	0.00656998	0.02163779	Up	ATEG_04637	hypothetical protein	-	GO:0016021//integral component of membrane	-	GO:0055085//transmembrane transport	gi|115396352|ref|XP_001213815.1|/0/conserved hypothetical protein [Aspergillus terreus NIH2624]
4354420	1302	392.64	1701.15	2.72136545	3.58E-36	1.55E-34	Up	ATEG_09887	hypothetical protein	ko01100//Metabolic pathways;ko04146//Peroxisome;ko00260//Glycine, serine and threonine metabolism;ko00310//Lysine degradation	-	GO:0016491//oxidoreductase activity	GO:0055114//oxidation-reduction process	gi|115443404|ref|XP_001218509.1|/0/conserved hypothetical protein [Aspergillus terreus NIH2624]
4355223	1281	178.28	765.45	2.705593892	2.84E-29	9.15E-28	Up	ATEG_00469	hypothetical protein	-	-	-	-	gi|115491855|ref|XP_001210555.1|/0/predicted protein [Aspergillus terreus NIH2624]

4319030	1533	36.99	165.08	2.688772198	1.79E-12	2.21E-11	Up	ATEG_07355	hypothetical protein	ko04113//Meiosis - yeast	GO:0016021//integral component of membrane	GO:0022891//substrate-specific transmembrane transporter activity	GO:0008643//carbohydrate transport;GO:0055085//transmembrane transport	gi|115387000|ref|XP_001210041.1|/0/conserved hypothetical protein [Aspergillus terreus NIH2624]
4354534	2199	36.41	163.67	2.688760204	1.37E-11	1.58E-10	Up	ATEG_09740	hypothetical protein	-	GO:0016021//integral component of membrane	-	-	gi|115443110|ref|XP_001218362.1|/0/conserved hypothetical protein [Aspergillus terreus NIH2624]
4315952	486	408.59	1712.99	2.68640059	1.30E-59	1.18E-57	Up	ATEG_01476	similar to ribose 5-phosphate isomerase	ko01100//Metabolic pathways;ko01110//Biosynthesis of secondary metabolites;ko01130//Biosynthesis of antibiotics;ko01230//Biosynthesis of amino acids;ko01200//Carbon metabolism;ko00051//Fructose and mannose metabolism;ko00030//Pentose phosphate pathway	GO:0005622//intracellular	GO:0004751//ribose-5-phosphate isomerase activity;GO:0050044	GO:0005975//carbohydrate metabolic process	gi|115384588|ref|XP_001208841.1|/2.00234e-110/hypothetical protein ATEG_01476 [Aspergillus terreus NIH2624]

4354421	1125	5124.99	21190.25	2.677328556	2.88E-83	4.44E-81	Up	ATEG_09888	hypothetical protein	ko01100//Metabolic pathways;ko01110//Biosynthesis of secondary metabolites	-	GO:0004497//monooxygenase activity;GO:0005506//iron ion binding;GO:0016708//oxidoreductase activity, acting on paired donors, with incorporation or reduction of molecular oxygen, NAD(P)H as one donor, and incorporation of two atoms of oxygen into one donor;GO:0051537//2 iron, 2 sulfur cluster binding	GO:0019439//aromatic compound catabolic process;GO:0055114//oxidation-reduction process	gi|115443406|ref|XP_001218510.1|/0/conserved hypothetical protein [Aspergillus terreus NIH2624]
4323021	2169	39.82	180.16	2.675625909	1.91E-10	1.98E-09	Up	ATEG_07849	hypothetical protein	-	GO:0016021//integral component of membrane	GO:0015171//amino acid transmembrane transporter activity	GO:0003333//amino acid transmembrane transport	gi|115401764|ref|XP_001216470.1|/0/predicted protein [Aspergillus terreus NIH2624]
4319082	2100	22.41	103.68	2.674770933	1.04E-08	9.02E-08	Up	ATEG_07347	hypothetical protein	-	-	GO:0010181//FMN binding;GO:0016491//oxidoreductase activity	GO:0055114//oxidation-reduction process	gi|115386984|ref|XP_001210033.1|/0/conserved hypothetical protein [Aspergillus terreus NIH2624]
4319613	3790	325.56	1355.65	2.672057174	5.41E-56	4.27E-54	Up	ATEG_09978	hypothetical protein	ko01100//Metabolic pathways;ko01110//Biosynthesis of secondary metabolites;ko00770//Pantothenate and CoA biosynthesis	GO:0016021//integral component of membrane	-	-	gi|115385467|ref|XP_001209280.1|/0/conserved hypothetical protein [Aspergillus terreus NIH2624]

4316775	1662	9.53	49.24	2.6675877	3.00E-05	0.00016662	Up	ATEG_02413	hypothetical protein	-	-	-	-	gi|115388171|ref|XP_001211591.1|/0/conserved hypothetical protein [Aspergillus terreus NIH2624]
4317400	1309	31.95	141.32	2.661070677	4.88E-11	5.34E-10	Up	ATEG_02784	hypothetical protein	-	-	-	-	gi|115388914|ref|XP_001211962.1|/3.46975e-84/predicted protein [Aspergillus terreus NIH2624]
4319554	981	208.5	862.43	2.659180571	1.61E-29	5.28E-28	Up	ATEG_10071	alpha-L-arabinofuranosidase precursor	ko00520//Amino sugar and nucleotide sugar metabolism	GO:0005576//extracellular region	GO:0046556//alpha-L-arabinofuranosidase activity	GO:0045493//xylan catabolic process;GO:0046373//L-arabinose metabolic process	gi|115385653|ref|XP_001209373.1|/0/alpha-L-arabinofuranosidase precursor [Aspergillus terreus NIH2624]
4353274	1174	508.34	2075.13	2.647405087	2.39E-67	2.74E-65	Up	ATEG_08440	hypothetical protein	-	-	-	-	gi|115433849|ref|XP_001217061.1|/0/predicted protein [Aspergillus terreus NIH2624]
4321446	1338	153.59	633.63	2.646905927	4.23E-30	1.44E-28	Up	ATEG_05713	similar to fructosyl-amino acid oxidase	ko01100//Metabolic pathways;ko04146//Peroxisome;ko00260//Glycine, serine and threonine metabolism;ko00310//Lysine degradation	-	GO:0051700//fructosyl-amino acid oxidase activity	GO:0055114//oxidation-reduction process	gi|115398604|ref|XP_001214891.1|/0/hypothetical protein ATEG_05713 [Aspergillus terreus NIH2624]
4316735	4679	2048.73	8286.97	2.64448152	1.05E-69	1.32E-67	Up	ATEG_02275	hypothetical protein	-	-	GO:0046872//metal ion binding	-	gi|115387895|ref|XP_001211453.1|/0/predicted protein [Aspergillus terreus NIH2624]

4353658	1218	1.68	17.72	2.642115718	0.00455166	0.01575951	Up	ATEG_09264	hypothetical protein	-	-	-	-	gi|115437736|ref|XP_001217886.1|/0/predicted protein [Aspergillus terreus NIH2624]
4353853	2017	165.82	681.07	2.637518832	6.22E-31	2.16E-29	Up	ATEG_09148	hypothetical protein	-	GO:0005634//nucleus	GO:0003677//DNA binding;GO:0008270//zinc ion binding	GO:0006351//transcription, DNA-templated	gi|115437278|ref|XP_001217770.1|/0/conserved hypothetical protein [Aspergillus terreus NIH2624]
4317698	1611	28.6	126.7	2.630649081	3.06E-08	2.54E-07	Up	ATEG_02855	hypothetical protein	ko00310//Lysine degradation	-	-	-	gi|115389056|ref|XP_001212033.1|/0/conserved hypothetical protein [Aspergillus terreus NIH2624]
4315972	944	36.41	156.03	2.625869763	2.18E-11	2.47E-10	Up	ATEG_01870	hypothetical protein	-	-	-	-	gi|115385376|ref|XP_001209235.1|/4.02847e-125/predicted protein [Aspergillus terreus NIH2624]
4321291	3401	159.18	665.08	2.616083901	4.90E-19	9.26E-18	Up	ATEG_05995	hypothetical protein	-	GO:0016021//integral component of membrane	GO:0003824//catalytic activity	GO:0008152//metabolic process	gi|115399168|ref|XP_001215173.1|/0/predicted protein [Aspergillus terreus NIH2624]
4321110	1655	210.09	838.55	2.605400511	5.88E-30	1.98E-28	Up	ATEG_05114	similar to class III aminotransferase	ko04120//Ubiquitin mediated proteolysis	-	GO:0008483//transaminase activity;GO:0030170//pyridoxal phosphate binding	-	gi|115397401|ref|XP_001214292.1|/0/hypothetical protein ATEG_05114 [Aspergillus terreus NIH2624]

4315796	2130	12.33	60.86	2.601194495	2.47E-05	0.00013868	Up	ATEG_01178	glutamyl-tRNA synthetase	ko01100//Metabolic pathways;ko01110//Biosynthesis of secondary metabolites;ko00970//Aminoacyl-tRNA biosynthesis;ko00860//Porphyrin and chlorophyll metabolism	GO:0005634//nucleus;GO:0005739//mitochondrion;GO:0005829//cytosol;GO:0017102//methionyl glutamyl tRNA synthetase complex	GO:0004818//glutamate-tRNA ligase activity;GO:0005524//ATP binding	GO:0006424//glutamyl-tRNA aminoacylation	gi|115383992|ref|XP_001208543.1|/0/glutamyl-tRNA synthetase [Aspergillus terreus NIH2624]
4319414	1500	100.93	409.06	2.598086645	4.92E-15	7.05E-14	Up	ATEG_10009	hypothetical protein	ko01100//Metabolic pathways;ko01110//Biosynthesis of secondary metabolites;ko00350//Tyrosine metabolism;ko00010//Glycolysis / Gluconeogenesis;ko00360//Phenylalanine metabolism;ko00410//beta-Alanine metabolism;ko00340//Histidine metabolism	-	GO:0016620//oxidoreductase activity, acting on the aldehyde or oxo group of donors, NAD or NADP as acceptor	GO:0055114//oxidation-reduction process	gi|115385529|ref|XP_001209311.1|/0/predicted protein [Aspergillus terreus NIH2624]
4354065	1605	4.48	27.5	2.59279037	0.00109613	0.00444083	Up	ATEG_09114	hypothetical protein	-	-	-	-	gi|115437142|ref|XP_001217736.1|/0/predicted protein [Aspergillus terreus NIH2624]
4317546	2103	94.16	374.63	2.582258476	3.05E-21	6.73E-20	Up	ATEG_03274	hypothetical protein	-	GO:0005634//nucleus;GO:0072686//mitotic spindle	-	-	gi|115389894|ref|XP_001212452.1|/0/predicted protein [Aspergillus terreus NIH2624]
4316783	966	25.23	107.61	2.57587299	5.20E-08	4.26E-07	Up	ATEG_02248	hypothetical protein	ko01200//Carbon metabolism;ko00680//Methane metabolism	-	GO:0003824//catalytic activity	GO:0008152//metabolic process	gi|115387841|ref|XP_001211426.1|/0/predicted protein [Aspergillus terreus NIH2624]

4319031	1545	33.09	139.37	2.564579741	7.63E-09	6.76E-08	Up	ATEG_07356	hypothetical protein	ko01100//Metabolic pathways;ko00500//Starch and sucrose metabolism;ko00052//Galactose metabolism	-	GO:0004553//hydrolase activity, hydrolyzing O-glycosyl compounds	GO:0005975//carbohydrate metabolic process	gi|115387002|ref|XP_001210042.1|/0/conserved hypothetical protein [Aspergillus terreus NIH2624]
4321384	1614	1047.94	4009.97	2.563151324	1.53E-52	1.11E-50	Up	ATEG_06165	hypothetical protein	ko00380//Tryptophan metabolism;ko00360//Phenylalanine metabolism;ko00330//Arginine and proline metabolism	-	GO:0016884//carbon-nitrogen ligase activity, with glutamine as amido-N-donor	-	gi|115399508|ref|XP_001215343.1|/0/conserved hypothetical protein [Aspergillus terreus NIH2624]
4323047	2236	112.6	444.12	2.56093621	6.36E-22	1.44E-20	Up	ATEG_07579	hypothetical protein	-	-	-	-	gi|115401224|ref|XP_001216200.1|/0/predicted protein [Aspergillus terreus NIH2624]
4353618	2747	179.91	691.47	2.551270247	2.05E-25	5.69E-24	Up	ATEG_09390	similar to regulatory protein	-	GO:0005634//nucleus	GO:0003700//transcription factor activity, sequence-specific DNA binding;GO:0008270//zinc ion binding;GO:0044212//transcription regulatory region DNA binding	GO:0006366//transcription from RNA polymerase II promoter;GO:0034251	gi|115438226|ref|XP_001218012.1|/0/hypothetical protein ATEG_09390 [Aspergillus terreus NIH2624]
4353469	1538	24.66	102.96	2.548905797	2.79E-08	2.32E-07	Up	ATEG_08417	hypothetical protein	-	GO:0016021//integral component of membrane	GO:0022857//transmembrane transporter activity	GO:0055085//transmembrane transport	gi|115433803|ref|XP_001217038.1|/0/predicted protein [Aspergillus terreus NIH2624]
4322216	1605	33.07	134.73	2.545602815	3.78E-09	3.46E-08	Up	ATEG_06439	hypothetical protein	ko03440//Homologous recombination;ko03450//Non-homologous end-joining	GO:0016021//integral component of membrane	-	GO:0055085//transmembrane transport	gi|115400057|ref|XP_001215617.1|/0/predicted protein [Aspergillus terreus NIH2624]

4316101	1617	2.8	19.79	2.539337516	0.00355643	0.01265931	Up	ATEG_01003	similar to maltose porter	-	GO:0005887//integral component of plasma membrane	GO:0005351//sugar:proton symporter activity;GO:0005355//glucose transmembrane transporter activity	GO:0015992//proton transport;GO:0046323//glucose import;GO:1904659//glucose transmembrane transport	gi|115383642|ref|XP_001208368.1|/0/hypothetical protein ATEG_01003 [Aspergillus terreus NIH2624]
4319162	4273	1536.32	5784.5	2.537943278	1.96E-65	2.09E-63	Up	ATEG_07167	serine/threonine-protein kinase sid2	ko04111//Cell cycle - yeast	GO:0005829//cytosol;GO:0031097//medial cortex;GO:0034973//Sid2-Mob1 complex;GO:0044732//mitotic spindle pole body	GO:0004674//protein serine/threonine kinase activity;GO:0005524//ATP binding	GO:0018105//peptidyl-serine phosphorylation;GO:0031028//septation initiation signaling;GO:0032463//negative regulation of protein homooligomerization;GO:0044878//mitotic cytokinesis checkpoint;GO:0051220//cytoplasmic sequestering of protein;GO:0061167//maintenance of endoplasmic reticulum location involved in endoplasmic reticulum polarization at cell division site;GO:1902817//negative regulation of protein localization to microtubule;GO:1902846//positive regulation of mitotic spindle elongation;GO:1902854//positive regulation of nuclear migration [illegible]	gi|115386624|ref|XP_001209853.1|/0/serine/threonine-protein kinase sid2 [Aspergillus terreus NIH2624]

4320953	1274	118.2	454.86	2.536744379	2.77E-19	5.41E-18	Up	ATEG_05014	hypothetical protein	ko01100//Metabolic pathways;ko00240//Pyrimidine metabolism;ko00250//Alanine, aspartate and glutamate metabolism	-	-	-	gi|115397201|ref|XP_001214192.1|/0/predicted protein [Aspergillus terreus NIH2624]
4319146	1980	29.73	123.38	2.534691105	2.46E-07	1.85E-06	Up	ATEG_07147	hypothetical protein	-	GO:0016021//integral component of membrane	GO:0016491//oxidoreductase activity;GO:0071949//FAD binding	GO:0055085//transmembrane transport;GO:0055114//oxidation-reduction process	gi|115386584|ref|XP_001209833.1|/0/conserved hypothetical protein [Aspergillus terreus NIH2624]
4322771	2085	50.42	204.26	2.532942719	4.83E-11	5.28E-10	Up	ATEG_07720	tryptophan synthase	ko01100//Metabolic pathways;ko01110//Biosynthesis of secondary metabolites;ko01230//Biosynthesis of amino acids;ko00260//Glycine, serine and threonine metabolism;ko00400//Phenylalanine, tyrosine and tryptophan biosynthesis	-	GO:0004834//tryptophan synthase activity	GO:0000162//tryptophan biosynthetic process	gi|115401506|ref|XP_001216341.1|/0/tryptophan synthase [Aspergillus terreus NIH2624]
4354287	2849	47.62	188.49	2.529987946	3.62E-12	4.38E-11	Up	ATEG_09876	DNA ligase (Polydeoxyribonucleotide synthase [ATP])	ko03420//Nucleotide excision repair;ko03030//DNA replication;ko03430//Mismatch repair;ko03410//Base excision repair	-	GO:0003677//DNA binding;GO:0003910//DNA ligase (ATP) activity;GO:0005524//ATP binding	GO:0006260//DNA replication;GO:0006310//DNA recombination;GO:0051103//DNA ligation involved in DNA repair;GO:0071897//DNA biosynthetic process	gi|115443382|ref|XP_001218498.1|/0/DNA ligase (Polydeoxyribonucleotide synthase [ATP]) [Aspergillus terreus NIH2624]

4317772	606	332.3	1246.35	2.525433329	8.42E-46	4.94E-44	Up	ATEG_03399	hypothetical protein	-	-	-	-	gi|115390144|ref|XP_001212577.1|/7.45086e-146/predicted protein [Aspergillus terreus NIH2624]
4319476	1533	38.69	154.16	2.524076567	2.83E-10	2.87E-09	Up	ATEG_09995	similar to polyamine transporter	-	GO:0016021//integral component of membrane	-	GO:0055085//transmembrane transport	gi|115385501|ref|XP_001209297.1|/0/hypothetical protein ATEG_09995 [Aspergillus terreus NIH2624]
4322495	912	52.11	208.39	2.518160345	2.96E-11	3.32E-10	Up	ATEG_07576	hypothetical protein	ko01100//Metabolic pathways;ko01200//Carbon metabolism;ko00280//Valine, leucine and isoleucine degradation;ko00640//Propanoate metabolism;ko00562//Inositol phosphate metabolism;ko00410//beta-Alanine metabolism	-	-	-	gi|115401218|ref|XP_001216197.1|/0/predicted protein [Aspergillus terreus NIH2624]
4353758	372	17.95	77.26	2.51563344	6.14E-06	3.77E-05	Up	ATEG_09092	hypothetical protein	-	GO:0005739//mitochondrion	GO:0042030//ATPase inhibitor activity	GO:0032780//negative regulation of ATPase activity	gi|115437054|ref|XP_001217714.1|/3.99724e-85/predicted protein [Aspergillus terreus NIH2624]
4321170	2082	21.83	92.83	2.515135336	3.04E-06	1.98E-05	Up	ATEG_05079	hypothetical protein	-	GO:0005634//nucleus	GO:0000981//RNA polymerase II transcription factor activity, sequence-specific DNA binding;GO:0003677//DNA binding;GO:0008270//zinc ion binding	GO:0006357//regulation of transcription from RNA polymerase II promoter	gi|115397331|ref|XP_001214257.1|/0/conserved hypothetical protein [Aspergillus terreus NIH2624]

4319040	1419	4.49	25.48	2.513335402	0.00167684	0.00650426	Up	ATEG_06914	hypothetical protein	ko01100//Metabolic pathways;ko01110//Biosynthesis of secondary metabolites	GO:0016021//integral component of membrane	GO:0004497//monooxygenase activity;GO:0005506//iron ion binding;GO:0009055//electron carrier activity;GO:0016705//oxidoreductase activity, acting on paired donors, with incorporation or reduction of molecular oxygen;GO:0020037//heme binding	GO:0055114//oxidation-reduction process	gi|115386118|ref|XP_001209600.1|/0/predicted protein [Aspergillus terreus NIH2624]
4317237	816	50.99	196.29	2.502866781	4.73E-12	5.67E-11	Up	ATEG_02685	hypothetical protein	ko00350//Tyrosine metabolism	-	GO:0003824//catalytic activity	-	gi|115388715|ref|XP_001211863.1|/0/conserved hypothetical protein [Aspergillus terreus NIH2624]
4354402	852	23.52	100.55	2.48263566	4.82E-06	3.01E-05	Up	ATEG_09715	hypothetical protein	-	-	-	-	gi|115443060|ref|XP_001218337.1|/0/predicted protein [Aspergillus terreus NIH2624]
4323211	1383	745.69	2692.64	2.4743098	3.00E-37	1.39E-35	Up	ATEG_08614	hypothetical protein	ko01100//Metabolic pathways;ko01220//Degradation of aromatic compounds	GO:0016021//integral component of membrane	GO:0004497//monooxygenase activity;GO:0071949//FAD binding	GO:0055114//oxidation-reduction process	gi|115402247|ref|XP_001217200.1|/0/conserved hypothetical protein [Aspergillus terreus NIH2624]

4317090	1248	376.56	1340.2	2.444854572	1.67E-47	1.06E-45	Up	ATEG_02147	bacterial hemoglobin	-	GO:0005829//cytosol	GO:0008941//nitric oxide dioxygenase activity;GO:0019825//oxygen binding;GO:0020037//heme binding;GO:0042602//riboflavin reductase (NADPH) activity	GO:0055114//oxidation-reduction process;GO:0071500//cellular response to nitrosative stress	gi|115387639|ref|XP_001211325.1|/0/bacterial hemoglobin [Aspergillus terreus NIH2624]
4354091	1095	266.7	953.77	2.443534935	1.98E-35	8.27E-34	Up	ATEG_09498	zinc-regulated transporter 2	-	GO:0005886//plasma membrane;GO:0016021//integral component of membrane	GO:0000007//low-affinity zinc ion transmembrane transporter activity	GO:0006831//low-affinity zinc II ion transport;GO:0010043//response to zinc ion	gi|115442626|ref|XP_001218120.1|/0/zinc-regulated transporter 2 [Aspergillus terreus NIH2624]
4353104	681	58.28	214.98	2.442354191	1.89E-13	2.46E-12	Up	ATEG_08454	similar to glutathione transferase 2	ko00480//Glutathione metabolism	GO:0005622//intracellular	GO:0004364//glutathione transferase activity;GO:0004602//glutathione peroxidase activity	-	gi|115433877|ref|XP_001217593.1|/1.55077e-173/hypothetical protein ATEG_08454 [Aspergillus terreus NIH2624]
4354419	2165	106.46	383.05	2.440304981	2.89E-16	4.56E-15	Up	ATEG_09886	hypothetical protein	-	GO:0005634//nucleus	GO:0000981//RNA polymerase II transcription factor activity, sequence-specific DNA binding;GO:0003677//DNA binding;GO:0008270//zinc ion binding	GO:0006357//regulation of transcription from RNA polymerase II promoter	gi|115443402|ref|XP_001218508.1|/0/conserved hypothetical protein [Aspergillus terreus NIH2624]

4353121	441	3.36	21.92	2.440136236	0.00496198	0.01695494	Up	ATEG_08324	similar to PENR2 protein	-	GO:0000776//kinetochore;GO:0005634//nucleus;GO:0005739//mitochondrion;GO:0089713//Cbf1-Met4-Met28 complex	GO:0000978//RNA polymerase II core promoter proximal region sequence-specific DNA binding;GO:0001076//transcription factor activity, RNA polymerase II transcription factor binding;GO:0001077//transcriptional activator activity, RNA polymerase II core promoter proximal region sequence-specific binding;GO:0001078//transcriptional repressor activity, RNA polymerase II core promoter proximal region sequence-specific binding;GO:0001102//RNA polymerase II activating transcription factor binding;GO:0001103//RNA polymerase II repressing	GO:0006338//chromatin remodeling;GO:0007059//chromosome segregation;GO:0009086//methionine biosynthetic process;GO:0061427//negative regulation of ceramide biosynthetic process by negative regulation of transcription from RNA Polymerase II promoter;GO:0061432//regulation of transcription from RNA polymerase II promoter in response to methionine;GO:1900375//positive regulation of inositol biosynthetic process by positive regulation of transcription from RNA polymerase II promoter;GO:1900478//positive	gi|115433616|ref|XP_001216945.1|/9.48971e-104/hypothetical protein ATEG_08324 [Aspergillus terreus NIH2624]

4354592	1311	46.47	176.99	2.438808264	9.38E-08	7.44E-07	Up	ATEG_10312	2,2-dialkylglycine decarboxylase	ko01100//Metabolic pathways;ko00650//Butanoate metabolism;ko00250//Alanine, aspartate and glutamate metabolism;ko00640//Propanoate metabolism;ko00410//beta-Alanine metabolism	GO:0005739//mitochondrion;GO:0005829//cytosol	GO:0008483//transaminase activity;GO:0030170//pyridoxal phosphate binding	-	gi|115449647|ref|XP_001218660.1|/0/2,2-dialkylglycine decarboxylase [Aspergillus terreus NIH2624]
4320264	1437	241.61	855.87	2.436382407	1.64E-24	4.26E-23	Up	ATEG_04844	hypothetical protein	ko01100//Metabolic pathways;ko00230//Purine metabolism;ko00240//Pyrimidine metabolism;ko03020//RNA polymerase	GO:0016021//integral component of membrane	GO:0022891//substrate-specific transmembrane transporter activity	GO:0008643//carbohydrate transport;GO:0055085//transmembrane transport	gi|115396766|ref|XP_001214022.1|/0/conserved hypothetical protein [Aspergillus terreus NIH2624]
4319145	1803	154.68	566.65	2.434694754	2.51E-17	4.21E-16	Up	ATEG_07146	hypothetical protein	ko03018//RNA degradation	GO:0016021//integral component of membrane	-	GO:0055085//transmembrane transport	gi|115386582|ref|XP_001209832.1|/0/conserved hypothetical protein [Aspergillus terreus NIH2624]
4323480	4316	1073.13	3753.4	2.434124976	1.29E-47	8.22E-46	Up	ATEG_08618	hypothetical protein	-	GO:0005634//nucleus;GO:0016021//integral component of membrane	GO:0003677//DNA binding;GO:0008270//zinc ion binding	GO:0006351//transcription, DNA-templated;GO:0055085//transmembrane transport	gi|115402255|ref|XP_001217204.1|/0/conserved hypothetical protein [Aspergillus terreus NIH2624]
4355224	4221	531.25	1856.78	2.429260703	1.51E-46	9.24E-45	Up	ATEG_00470	hypothetical protein	ko01100//Metabolic pathways;ko01110//Biosynthesis of secondary metabolites;ko00770//Pantothenate and CoA biosynthesis	GO:0016021//integral component of membrane	-	-	gi|115491857|ref|XP_001210556.1|/0/conserved hypothetical protein [Aspergillus terreus NIH2624]

4353612	1026	9914.32	34454.3	2.426492642	4.00E-67	4.47E-65	Up	ATEG_09213	pyrimidine precursor biosynthesis enzyme THI11	ko01100//Metabolic pathways;ko00730//Thiamine metabolism	GO:0005622//intracellular	-	GO:0009228//thiamine biosynthetic process	gi|115437534|ref|XP_001217835.1|/0/pyrimidine precursor biosynthesis enzyme THI11 [Aspergillus terreus NIH2624]
4315765	1659	72.82	265.99	2.424186231	1.30E-13	1.71E-12	Up	ATEG_01803	hydroxymethylglutaryl-CoA lyase, mitochondrial precursor	ko01100//Metabolic pathways;ko04146//Peroxisome;ko00650//Butanoate metabolism;ko00280//Valine, leucine and isoleucine degradation;ko00072//Synthesis and degradation of ketone bodies	-	GO:0016829//lyase activity	GO:0008152//metabolic process	gi|115385242|ref|XP_001209168.1|/0/hydroxymethylglutaryl-CoA lyase, mitochondrial precursor [Aspergillus terreus NIH2624]
4316125	2078	1963.1	6867.49	2.424153889	1.57E-56	1.30E-54	Up	ATEG_01192	hypothetical protein	ko01100//Metabolic pathways;ko01110//Biosynthesis of secondary metabolites;ko00770//Pantothenate and CoA biosynthesis	GO:0016021//integral component of membrane	-	-	gi|115384020|ref|XP_001208557.1|/0/conserved hypothetical protein [Aspergillus terreus NIH2624]
4322801	3603	22.96	89.14	2.421433925	2.03E-06	1.35E-05	Up	ATEG_07749	hypothetical protein	-	GO:0005622//intracellular	GO:0000155//phosphorelay sensor kinase activity	GO:0000160//phosphorelay signal transduction system;GO:0023014//signal transduction by protein phosphorylation	gi|115401564|ref|XP_001216370.1|/0/conserved hypothetical protein [Aspergillus terreus NIH2624]
4355176	2021	4229.39	14660.08	2.420550671	2.53E-53	1.89E-51	Up	ATEG_00422	hypothetical protein	-	GO:0016021//integral component of membrane	GO:0005215//transporter activity	GO:0006857//oligopeptide transport	gi|115491761|ref|XP_001210508.1|/0/conserved hypothetical protein [Aspergillus terreus NIH2624]

4321454	459	67.25	240.41	2.414262543	1.21E-13	1.59E-12	Up	ATEG_05745	hypothetical protein	ko00380//Tryptophan metabolism	-	-	-	gi|115398668|ref|XP_001214923.1|/2.12265e-112/predicted protein [Aspergillus terreus NIH2624]
4316784	1527	92.96	333.28	2.41259712	6.07E-12	7.23E-11	Up	ATEG_02249	hypothetical protein	ko04146//Peroxisome	GO:0016021//integral component of membrane	-	GO:0055085//transmembrane transport	gi|115387843|ref|XP_001211427.1|/1.61697e-141/predicted protein [Aspergillus terreus NIH2624]
4322550	1015	7.86	6435.73	2.410778769	0.01622498	0.04724983	Up	ATEG_07694	hypothetical protein	-	GO:0016021//integral component of membrane	-	-	gi|115401454|ref|XP_001216315.1|/2.93724e-139/predicted protein [Aspergillus terreus NIH2624]
4353657	1950	28	106.87	2.406029928	4.24E-07	3.11E-06	Up	ATEG_09263	hypothetical protein	-	-	-	-	gi|115437732|ref|XP_001217885.1|/0/conserved hypothetical protein [Aspergillus terreus NIH2624]
4355584	1623	2.24	15.85	2.403521859	0.00794867	0.02546341	Up	ATEG_00823	hypothetical protein	ko00562//Inositol phosphate metabolism	-	GO:0003993//acid phosphatase activity	GO:0016311//dephosphorylation	gi|115492563|ref|XP_001210909.1|/0/conserved hypothetical protein [Aspergillus terreus NIH2624]

4355159	522	1337.74	4572.58	2.400821746	1.61E-68	1.88E-66	Up	ATEG_00405	glycine cleavage system H protein	ko01100//Metabolic pathways;ko01110//Biosynthesis of secondary metabolites;ko01130//Biosynthesis of antibiotics;ko00260//Glycine, serine and threonine metabolism;ko00630//Glyoxylate and dicarboxylate metabolism	GO:0005739//mitochondrion;GO:0005960//glycine cleavage complex	-	GO:0006730//one-carbon metabolic process;GO:0009249//protein lipoylation;GO:0019464//glycine decarboxylation via glycine cleavage system;GO:0055114//oxidation-reduction process	gi|115491727|ref|XP_001210491.1|/3.51253e-119/glycine cleavage system H protein [Aspergillus terreus NIH2624]
4316050	1806	116.6	405.39	2.393053217	2.09E-16	3.36E-15	Up	ATEG_01230	hypothetical protein	ko01100//Metabolic pathways;ko01110//Biosynthesis of secondary metabolites;ko00350//Tyrosine metabolism;ko00260//Glycine, serine and threonine metabolism;ko00380//Tryptophan metabolism;ko00360//Phenylalanine metabolism;ko00330//Arginine and proline metabolism;ko00340//Histidine metabolism	-	GO:0016491//oxidoreductase activity	GO:0055114//oxidation-reduction process	gi|115384096|ref|XP_001208595.1|/0/conserved hypothetical protein [Aspergillus terreus NIH2624]
4317254	2040	0.56	9.43	2.390132598	0.01618019	0.04713405	Up	ATEG_02666	cholinesterase	ko00564//Glycerophospholipid metabolism	-	GO:0016787//hydrolase activity	-	gi|115388677|ref|XP_001211844.1|/0/cholinesterase [Aspergillus terreus NIH2624]

4317554	1571	187.73	645.96	2.386796828	2.14E-29	6.99E-28	Up	ATEG_02890	hypothetical protein	ko04011//MAPK signaling pathway - yeast	GO:0016021//integral component of membrane	-	GO:0055085//transmembrane transport	gi|115389126|ref|XP_001212068.1|/0/conserved hypothetical protein [Aspergillus terreus NIH2624]
4321458	1353	121.71	427.28	2.384643134	1.42E-12	1.76E-11	Up	ATEG_05923	homogentisate 1,2-dioxygenase	ko01100//Metabolic pathways;ko00350//Tyrosine metabolism	-	GO:0004411//homogentisate 1,2-dioxygenase activity	GO:0006559//L-phenylalanine catabolic process;GO:0006572//tyrosine catabolic process;GO:0055114//oxidation-reduction process	gi|115399024|ref|XP_001215101.1|/0/homogentisate 1,2-dioxygenase [Aspergillus terreus NIH2624]
4317862	2058	303.72	1032.38	2.379615767	4.00E-40	1.98E-38	Up	ATEG_03018	hypothetical protein	ko03040//Spliceosome	-	GO:0016872//intramolecular lyase activity	-	gi|115389382|ref|XP_001212196.1|/0/conserved hypothetical protein [Aspergillus terreus NIH2624]
4353826	2217	1.12	12.25	2.379185248	0.01447203	0.04264794	Up	ATEG_09130	hypothetical protein	-	GO:0005634//nucleus	GO:0000981//RNA polymerase II transcription factor activity, sequence-specific DNA binding;GO:0003677//DNA binding;GO:0008270//zinc ion binding	GO:0006357//regulation of transcription from RNA polymerase II promoter	gi|115437204|ref|XP_001217752.1|/0/predicted protein [Aspergillus terreus NIH2624]
4318279	4859	26.37	109.14	2.377233552	8.43E-05	0.00042927	Up	ATEG_03928	similar to multidrug resistance protein	ko02010//ABC transporters	GO:0016021//integral component of membrane	GO:0005524//ATP binding;GO:0042626//ATPase activity, coupled to transmembrane movement of substances	GO:0055085//transmembrane transport	gi|115391203|ref|XP_001213106.1|/0/hypothetical protein ATEG_03928 [Aspergillus terreus NIH2624]

4321259	4096	105.35	360.28	2.366812874	3.45E-19	6.63E-18	Up	ATEG_05796	hypothetical protein	ko01100//Metabolic pathways;ko00052//Galactose metabolism	GO:0005634//nucleus;GO:0016021//integral component of membrane	GO:0000981//RNA polymerase II transcription factor activity, sequence-specific DNA binding;GO:0003677//DNA binding;GO:0008270//zinc ion binding	GO:0006357//regulation of transcription from RNA polymerase II promoter	gi|115398770|ref|XP_001214974.1|/0/conserved hypothetical protein [Aspergillus terreus NIH2624]
4321063	921	76.21	261.81	2.36286803	9.51E-14	1.27E-12	Up	ATEG_05062	hypothetical protein	ko01100//Metabolic pathways;ko00280//Valine, leucine and isoleucine degradation	-	GO:0004616//phosphogluconate dehydrogenase (decarboxylating) activity;GO:0051287//NAD binding	GO:0006098//pentose-phosphate shunt;GO:0055114//oxidation-reduction process	gi|115397297|ref|XP_001214240.1|/0/conserved hypothetical protein [Aspergillus terreus NIH2624]
4353115	1564	779.97	2605.6	2.355908462	5.20E-55	4.00E-53	Up	ATEG_08166	hypothetical protein	ko04144//Endocytosis	-	-	-	gi|115433300|ref|XP_001216787.1|/0/predicted protein [Aspergillus terreus NIH2624]
4323135	2658	19.59	76.02	2.352090292	4.61E-05	0.00024692	Up	ATEG_08741	hypothetical protein	ko03013//RNA transport;ko04011//MAPK signaling pathway - yeast	-	-	-	gi|115402501|ref|XP_001217327.1|/0/predicted protein [Aspergillus terreus NIH2624]
4354164	2055	31.38	111.11	2.340757038	1.64E-07	1.26E-06	Up	ATEG_09786	hypothetical protein	ko00970//Aminoacyl-tRNA biosynthesis	GO:0005634//nucleus	GO:0000981//RNA polymerase II transcription factor activity, sequence-specific DNA binding;GO:0003677//DNA binding;GO:0008270//zinc ion binding	GO:0006357//regulation of transcription from RNA polymerase II promoter	gi|115443202|ref|XP_001218408.1|/0/conserved hypothetical protein [Aspergillus terreus NIH2624]
4320228	888	18.51	71.05	2.337700702	3.55E-05	0.00019496	Up	ATEG_04856	similar to mitochondrion protein	ko01200//Carbon metabolism;ko00680//Methane metabolism	-	GO:0016787//hydrolase activity	GO:0008152//metabolic process	gi|115396790|ref|XP_001214034.1|/0/hypothetical protein ATEG_04856 [Aspergillus terreus NIH2624]

4317527	1010	58.32	200.13	2.331349159	3.43E-09	3.16E-08	Up	ATEG_03426	hypothetical protein	ko00051//Fructose and mannose metabolism	-	GO:0016491//oxidoreductase activity	GO:0055114//oxidation-reduction process	gi|115390198|ref|XP_001212604.1|/0/conserved hypothetical protein [Aspergillus terreus NIH2624]
4315690	696	10.07	43.08	2.330564915	0.00101212	0.00413618	Up	ATEG_01649	hypothetical protein	-	-	-	-	gi|115384934|ref|XP_001209014.1|/2.46521e-168/predicted protein [Aspergillus terreus NIH2624]
4321447	1236	2105.75	6835.7	2.32851062	1.54E-38	7.35E-37	Up	ATEG_05714	formamidase	ko01200//Carbon metabolism;ko00460//Cyanoamino acid metabolism;ko00630//Glyoxylate and dicarboxylate metabolism;ko00910//Nitrogen metabolism	GO:0005576//extracellular region	GO:0004328//formamidase activity	GO:0008152//metabolic process	gi|115398606|ref|XP_001214892.1|/0/formamidase [Aspergillus terreus NIH2624]
4319118	1185	161.4	533.48	2.327738996	5.55E-25	1.50E-23	Up	ATEG_07304	hypothetical protein	ko01100//Metabolic pathways;ko00460//Cyanoamino acid metabolism;ko00480//Glutathione metabolism;ko00430//Taurine and hypotaurine metabolism	GO:0005634//nucleus;GO:0016021//integral component of membrane	GO:0000981//RNA polymerase II transcription factor activity, sequence-specific DNA binding;GO:0008270//zinc ion binding	GO:0006357//regulation of transcription from RNA polymerase II promoter	gi|115386898|ref|XP_001209990.1|/0/predicted protein [Aspergillus terreus NIH2624]
4316778	939	151.94	516.67	2.322763114	7.11E-14	9.53E-13	Up	ATEG_02561	hypothetical protein	ko01100//Metabolic pathways;ko00760//Nicotinate and nicotinamide metabolism	-	-	-	gi|115388467|ref|XP_001211739.1|/0/conserved hypothetical protein [Aspergillus terreus NIH2624]

4316083	1611	23.53	85.55	2.322361909	1.86E-05	0.00010673	Up	ATEG_01293	hypothetical protein	-	GO:0005634//nucleus	GO:0000981//RNA polymerase II transcription factor activity, sequence-specific DNA binding;GO:0003677//DNA binding;GO:0008270//zinc ion binding	GO:0006357//regulation of transcription from RNA polymerase II promoter	gi|115384222|ref|XP_001208658.1|/0/predicted protein [Aspergillus terreus NIH2624]
4323028	1689	511.24	1660.95	2.319917524	7.45E-37	3.36E-35	Up	ATEG_07524	hypothetical protein	-	GO:0016021//integral component of membrane	GO:0015171//amino acid transmembrane transporter activity	GO:0003333//amino acid transmembrane transport	gi|115401114|ref|XP_001216145.1|/0/conserved hypothetical protein [Aspergillus terreus NIH2624]
4322800	714	16.81	62.84	2.315710482	9.04E-05	0.00045738	Up	ATEG_07748	polygalacturonase-4 precursor	ko01100//Metabolic pathways;ko00500//Starch and sucrose metabolism;ko00040//Pentose and glucuronate interconversions	GO:0005576//extracellular region	GO:0004650//polygalacturonase activity	GO:0005975//carbohydrate metabolic process;GO:0071555//cell wall organization	gi|115401562|ref|XP_001216369.1|/2.9887e-172/polygalacturonase-4 precursor [Aspergillus terreus NIH2624]
4316028	888	628.83	2024.3	2.312478888	2.47E-33	9.45E-32	Up	ATEG_01361	hypothetical protein	ko03018//RNA degradation	-	GO:0016740//transferase activity	-	gi|115384358|ref|XP_001208726.1|/0/predicted protein [Aspergillus terreus NIH2624]
4321714	2727	35.87	123.25	2.310527667	6.83E-08	5.50E-07	Up	ATEG_05763	similar to acyl-CoA synthase	ko01100//Metabolic pathways;ko04146//Peroxisome;ko00071//Fatty acid degradation;ko01212//Fatty acid metabolism;ko00061//Fatty acid biosynthesis	-	GO:0004467//long-chain fatty acid-CoA ligase activity	GO:0001676//long-chain fatty acid metabolic process	gi|115398704|ref|XP_001214941.1|/0/hypothetical protein ATEG_05763 [Aspergillus terreus NIH2624]

4321120	1665	72.26	251.66	2.309331025	5.34E-09	4.80E-08	Up	ATEG_05259	hypothetical protein	ko01100//Metabolic pathways;ko01220//Degradation of aromatic compounds	GO:0016020//membrane	GO:0005506//iron ion binding;GO:0016705//oxidoreductase activity, acting on paired donors, with incorporation or reduction of molecular oxygen;GO:0020037//heme binding	GO:0055114//oxidation-reduction process	gi|115397691|ref|XP_001214437.1|/0/predicted protein [Aspergillus terreus NIH2624]
4317076	3003	42.59	144.61	2.309147616	3.07E-09	2.84E-08	Up	ATEG_02599	hypothetical protein	-	-	-	GO:0007051//spindle organization	gi|115388543|ref|XP_001211777.1|/0/predicted protein [Aspergillus terreus NIH2624]
4322776	2182	452.17	1461.18	2.308856289	1.20E-43	6.66E-42	Up	ATEG_07804	similar to oxalate decarboxylase	ko04144//Endocytosis	-	GO:0045735//nutrient reservoir activity	GO:0033609//oxalate metabolic process	gi|115401674|ref|XP_001216425.1|/0/hypothetical protein ATEG_07804 [Aspergillus terreus NIH2624]
4316080	999	8.39	37.44	2.308679044	0.00206371	0.00783324	Up	ATEG_01290	hypothetical protein	ko03440//Homologous recombination	-	-	-	gi|115384216|ref|XP_001208655.1|/0/predicted protein [Aspergillus terreus NIH2624]
4354197	1461	427.5	1376.26	2.307770684	1.10E-29	3.64E-28	Up	ATEG_09778	hypothetical protein	ko01100//Metabolic pathways;ko00230//Purine metabolism;ko00250//Alanine, aspartate and glutamate metabolism	GO:0016021//integral component of membrane	-	GO:0055085//transmembrane transport	gi|115443186|ref|XP_001218400.1|/0/predicted protein [Aspergillus terreus NIH2624]

4353842	2097	4.48	22.05	2.307304385	0.00457084	0.01582007	Up	ATEG_09139	hypothetical protein	ko01100//Metabolic pathways;ko01110//Biosynthesis of secondary metabolites;ko00650//Butanoate metabolism;ko00250//Alanine, aspartate and glutamate metabolism;ko00410//beta-Alanine metabolism;ko00430//Taurine and hypotaurine metabolism	-	GO:0016831//carboxy-lyase activity;GO:0030170//pyridoxal phosphate binding	GO:0019752//carboxylic acid metabolic process	gi|115437242|ref|XP_001217761.1|/0/predicted protein [Aspergillus terreus NIH2624]
4353581	2601	96.42	316.01	2.300925835	1.59E-15	2.37E-14	Up	ATEG_08205	hypothetical protein	-	GO:0016021//integral component of membrane	-	-	gi|115433378|ref|XP_001216826.1|/0/conserved hypothetical protein [Aspergillus terreus NIH2624]
4321806	1551	23.54	83.19	2.299813156	2.92E-06	1.91E-05	Up	ATEG_05823	hypothetical protein	-	GO:0016021//integral component of membrane	-	GO:0055085//transmembrane transport	gi|115398824|ref|XP_001215001.1|/0/predicted protein [Aspergillus terreus NIH2624]
4354605	1731	70.57	235.58	2.299184348	6.25E-11	6.76E-10	Up	ATEG_10285	hypothetical protein	-	-	-	-	gi|115449539|ref|XP_001218633.1|/6.01203e-92/predicted protein [Aspergillus terreus NIH2624]
4353307	2201	3.37	18.89	2.298056239	0.00864373	0.02737229	Up	ATEG_08241	hypothetical protein	-	GO:0005634//nucleus	GO:0003677//DNA binding;GO:0008270//zinc ion binding	GO:0006351//transcription, DNA-templated	gi|115433450|ref|XP_001216862.1|/0/predicted protein [Aspergillus terreus NIH2624]

4353821	360	7.85	33.45	2.294209702	0.0014803	0.00582374	Up	ATEG_09125	hypothetical protein	ko04144//Endocytosis	-	-	-	gi|115437184|ref|XP_001217747.1|/3.08294e-87/predicted protein [Aspergillus terreus NIH2624]
4355134	1386	3.37	18.77	2.290463615	0.00923083	0.02881334	Up	ATEG_00380	hypothetical protein	-	-	GO:0016491//oxidoreductase activity	GO:0055114//oxidation-reduction process	gi|115491677|ref|XP_001210466.1|/0/conserved hypothetical protein [Aspergillus terreus NIH2624]
4315888	1431	244.32	781.29	2.288491686	1.76E-31	6.31E-30	Up	ATEG_01295	hypothetical protein	ko00620//Pyruvate metabolism	-	GO:0008762//UDP-N-acetylmuramate dehydrogenase activity;GO:0050660//flavin adenine dinucleotide binding	GO:0055114//oxidation-reduction process	gi|115384226|ref|XP_001208660.1|/0/predicted protein [Aspergillus terreus NIH2624]
4317650	1857	165.46	540.94	2.287973412	4.73E-13	6.11E-12	Up	ATEG_02866	hypothetical protein	ko04144//Endocytosis	-	-	-	gi|115389078|ref|XP_001212044.1|/0/conserved hypothetical protein [Aspergillus terreus NIH2624]
4319450	3414	4.48	23	2.278307088	0.00701507	0.02288677	Up	ATEG_10145	hypothetical protein	-	GO:0016021//integral component of membrane	GO:0046873//metal ion transmembrane transporter activity	GO:0030001//metal ion transport;GO:0055085//transmembrane transport	gi|115385801|ref|XP_001209447.1|/0/predicted protein [Aspergillus terreus NIH2624]
4353282	2976	633.2	1989.26	2.276960385	8.50E-40	4.18E-38	Up	ATEG_08013	hypothetical protein	-	GO:0005576//extracellular region	GO:0004177//aminopeptidase activity;GO:0008237//metallopeptidase activity;GO:0008270//zinc ion binding	GO:0006508//proteolysis	gi|115432994|ref|XP_001216634.1|/0/conserved hypothetical protein [Aspergillus terreus NIH2624]

4354087	1185	91.38	293.72	2.271496817	4.80E-14	6.55E-13	Up	ATEG_09495	hypothetical protein	ko01200//Carbon metabolism;ko00460//Cyanoamino acid metabolism;ko00630//Glyoxylate and dicarboxylate metabolism;ko00910//Nitrogen metabolism	-	GO:0016811//hydrolase activity, acting on carbon-nitrogen (but not peptide) bonds, in linear amides	-	gi|115442620|ref|XP_001218117.1|/0/predicted protein [Aspergillus terreus NIH2624]
4317050	1638	4.49	21.54	2.271102406	0.00569669	0.01907637	Up	ATEG_02608	hypothetical protein	-	-	GO:0004499//N,N-dimethylaniline monooxygenase activity;GO:0050660//flavin adenine dinucleotide binding;GO:0050661//NADP binding	GO:0055114//oxidation-reduction process	gi|115388561|ref|XP_001211786.1|/0/conserved hypothetical protein [Aspergillus terreus NIH2624]
4316399	2973	7872.88	24434.58	2.260315488	9.35E-83	1.42E-80	Up	ATEG_01703	plasma membrane ATPase 2	ko00190//Oxidative phosphorylation	GO:0016021//integral component of membrane	GO:0005524//ATP binding;GO:0008553//hydrogen-exporting ATPase activity, phosphorylative mechanism;GO:0046872//metal ion binding	GO:0006754//ATP biosynthetic process;GO:1902600//hydrogen ion transmembrane transport	gi|115385042|ref|XP_001209068.1|/0/plasma membrane ATPase 2 [Aspergillus terreus NIH2624]
4320913	4123	321.12	991.46	2.24599004	5.18E-22	1.18E-20	Up	ATEG_05246	similar to acetyl-CoA carboxylase	ko01100//Metabolic pathways;ko00220//Arginine biosynthesis	GO:0016021//integral component of membrane	GO:0004075//biotin carboxylase activity;GO:0005524//ATP binding;GO:0046872//metal ion binding	-	gi|115397665|ref|XP_001214424.1|/0/hypothetical protein ATEG_05246 [Aspergillus terreus NIH2624]
4317673	1605	95.25	300.42	2.245288143	3.64E-15	5.28E-14	Up	ATEG_03480	hypothetical protein	ko00970//Aminoacyl-tRNA biosynthesis	-	-	-	gi|115390306|ref|XP_001212658.1|/0/conserved hypothetical protein [Aspergillus terreus NIH2624]

4319969	1368	78.49	249.29	2.243353155	1.72E-12	2.13E-11	Up	ATEG_04390	endoglucanase 3 precursor	ko01100//Metabolic pathways;ko00500//Starch and sucrose metabolism	GO:0005576//extracellular region	GO:0004553//hydrolase activity, hydrolyzing O-glycosyl compounds;GO:0030248//cellulose binding	GO:0005975//carbohydrate metabolic process	gi|115395858|ref|XP_001213568.1|/0/endoglucanase 3 precursor [Aspergillus terreus NIH2624]
4321858	1284	2.8	15.68	2.240660973	0.01215874	0.03668343	Up	ATEG_05725	hypothetical protein	-	GO:0016021//integral component of membrane	GO:0022891//substrate-specific transmembrane transporter activity	GO:0008643//carbohydrate transport;GO:0055085//transmembrane transport	gi|115398628|ref|XP_001214903.1|/0/conserved hypothetical protein [Aspergillus terreus NIH2624]
4355313	1545	5.03	25.99	2.239504029	0.00987472	0.03054878	Up	ATEG_00559	hypothetical protein	-	-	-	-	gi|115492035|ref|XP_001210645.1|/0/conserved hypothetical protein [Aspergillus terreus NIH2624]
4354318	2259	146.9	462.1	2.236098345	1.09E-15	1.65E-14	Up	ATEG_09544	hypothetical protein	ko04144//Endocytosis	-	-	-	gi|115442718|ref|XP_001218166.1|/0/conserved hypothetical protein [Aspergillus terreus NIH2624]

4319233	1185	122.17	379.34	2.23422019	3.95E-18	6.98E-17	Up	ATEG_06864	serine/threonine-protein kinase Eg2	-	GO:0000778//condensed nuclear chromosome kinetochore;GO:0032133//chromosome passenger complex;GO:1990023//mitotic spindle midzone	GO:0004674//protein serine/threonine kinase activity;GO:0005524//ATP binding	GO:0000281//mitotic cytokinesis;GO:0007034//vacuolar transport;GO:0007094//mitotic spindle assembly checkpoint;GO:0034501//protein localization to kinetochore;GO:0034503//protein localization to nucleolar rDNA repeats;GO:0098783//correction of merotelic kinetochore attachment, mitotic;GO:2000775//histone H3-S10 phosphorylation involved in chromosome condensation	gi|115386018|ref|XP_001209550.1|/0/serine/threonine-protein kinase Eg2 [Aspergillus terreus NIH2624]
4317490	363	24.67	84.23	2.232942602	5.28E-05	0.00027879	Up	ATEG_02907	hypothetical protein	-	-	-	-	gi|115389160|ref|XP_001212085.1|/8.83015e-83/conserved hypothetical protein [Aspergillus terreus NIH2624]
4316197	933	5795.74	17632.4	2.224615391	5.17E-64	5.16E-62	Up	ATEG_01418	hypothetical protein	ko01100//Metabolic pathways;ko00051//Fructose and mannose metabolism;ko00650//Butanoate metabolism;ko00591//Linoleic acid metabolism	-	GO:0004616//phosphogluconate dehydrogenase (decarboxylating) activity;GO:0051287//NAD binding	GO:0055114//oxidation-reduction process	gi|115384472|ref|XP_001208783.1|/0/conserved hypothetical protein [Aspergillus terreus NIH2624]

4322630	1427	325.56	985.44	2.217869723	6.34E-28	1.97E-26	Up	ATEG_07593	pectin lyase A precursor	-	GO:0005576//extracellular region	GO:0047490//pectin lyase activity	GO:0045490//pectin catabolic process;GO:0071555//cell wall organization	gi|115401252|ref|XP_001216214.1|/0/pectin lyase A precursor [Aspergillus terreus NIH2624]
4317066	1623	81.79	255.31	2.217494294	1.22E-12	1.54E-11	Up	ATEG_02157	hypothetical protein	-	GO:0016021//integral component of membrane	GO:0015171//amino acid transmembrane transporter activity	GO:0003333//amino acid transmembrane transport	gi|115387659|ref|XP_001211335.1|/0/conserved hypothetical protein [Aspergillus terreus NIH2624]
4354422	2673	20.18	69.64	2.216124302	4.54E-05	0.00024372	Up	ATEG_09889	hypothetical protein	-	GO:0005618//cell wall;GO:0016020//membrane	GO:0004252//serine-type endopeptidase activity	GO:0006508//proteolysis	gi|115443408|ref|XP_001218511.1|/0/predicted protein [Aspergillus terreus NIH2624]
4319629	3504	61.1	191.17	2.213718875	5.27E-10	5.19E-09	Up	ATEG_09988	similar to na,K-ATPase alpha subunit	ko00230//Purine metabolism	GO:0005887//integral component of plasma membrane;GO:0043231//intracellular membrane-bounded organelle	GO:0005388//calcium-transporting ATPase activity;GO:0005524//ATP binding;GO:0008900//hydrogen:potassium-exchanging ATPase activity;GO:0046872//metal ion binding	GO:0006091//generation of precursor metabolites and energy;GO:0006874//cellular calcium ion homeostasis;GO:0070588//calcium ion transmembrane transport;GO:1902600//hydrogen ion transmembrane transport	gi|115385487|ref|XP_001209290.1|/0/hypothetical protein ATEG_09988 [Aspergillus terreus NIH2624]
4355507	630	501.75	1525.43	2.210278522	3.25E-28	1.02E-26	Up	ATEG_00752	hypothetical protein	-	GO:0005576//extracellular region;GO:0005622//intracellular	GO:0016491//oxidoreductase activity	GO:0055114//oxidation-reduction process	gi|115492421|ref|XP_001210838.1|/2.29749e-153/conserved hypothetical protein [Aspergillus terreus NIH2624]

4319041	1384	249.46	755.14	2.209787531	4.73E-23	1.15E-21	Up	ATEG_07305	hypothetical protein	-	GO:0016021//integral component of membrane	GO:0004659//prenyltransferase activity	-	gi|115386900|ref|XP_001209991.1|/0/predicted protein [Aspergillus terreus NIH2624]
4319615	1275	4.49	21.85	2.209048219	0.00880436	0.02779654	Up	ATEG_09980	hypothetical protein	-	-	GO:0004659//prenyltransferase activity;GO:0050364	GO:0045461;GO:1900796	gi|115385471|ref|XP_001209282.1|/0/predicted protein [Aspergillus terreus NIH2624]
4323032	2043	20.74	71.61	2.20823738	5.40E-05	0.00028389	Up	ATEG_07528	hypothetical protein	-	GO:0016021//integral component of membrane	GO:0003676//nucleic acid binding;GO:0008270//zinc ion binding;GO:0016491//oxidoreductase activity;GO:0071949//FAD binding	GO:0055114//oxidation-reduction process	gi|115401122|ref|XP_001216149.1|/0/predicted protein [Aspergillus terreus NIH2624]
4320709	1730	14.56	51.5	2.206100191	0.00035054	0.00159743	Up	ATEG_05324	hypothetical protein	-	-	GO:0004672//protein kinase activity;GO:0005524//ATP binding	GO:0006468//protein phosphorylation	gi|115397821|ref|XP_001214502.1|/0/predicted protein [Aspergillus terreus NIH2624]
4322081	1920	917.48	2758.03	2.205777337	1.69E-24	4.38E-23	Up	ATEG_06562	hypothetical protein	ko01100//Metabolic pathways;ko00510//N-Glycan biosynthesis;ko00513//Various types of N-glycan biosynthesis	GO:0016021//integral component of membrane	-	-	gi|115400303|ref|XP_001215740.1|/0/conserved hypothetical protein [Aspergillus terreus NIH2624]
4354092	2545	42.62	140.13	2.203803492	1.41E-06	9.53E-06	Up	ATEG_09499	hypothetical protein	-	GO:0005634//nucleus	GO:0003677//DNA binding;GO:0008270//zinc ion binding	GO:0006351//transcription, DNA-templated	gi|115442628|ref|XP_001218121.1|/0/predicted protein [Aspergillus terreus NIH2624]
4353360	978	31.91	105.09	2.201073036	1.89E-05	0.00010822	Up	ATEG_08033	hypothetical protein	ko01100//Metabolic pathways;ko00561//Glycerolipid metabolism	-	GO:0016787//hydrolase activity	GO:0008152//metabolic process	gi|115433034|ref|XP_001216654.1|/0/conserved hypothetical protein [Aspergillus terreus NIH2624]

4320625	984	58.86	182.95	2.199823266	7.25E-10	7.08E-09	Up	ATEG_05584	hypothetical protein	-	GO:0005739//mitochondrion;GO:0016021//integral component of membrane	-	GO:0055085//transmembrane transport	gi|115398341|ref|XP_001214762.1|/0/conserved hypothetical protein [Aspergillus terreus NIH2624]
4322939	980	120.54	364.47	2.18962384	1.03E-13	1.36E-12	Up	ATEG_07789	hypothetical protein	-	-	GO:0016491//oxidoreductase activity;GO:0071949//FAD binding	GO:0055114//oxidation-reduction process	gi|115401644|ref|XP_001216410.1|/0/conserved hypothetical protein [Aspergillus terreus NIH2624]
4321370	1353	6.16	25.77	2.188643701	0.00467627	0.01613143	Up	ATEG_05968	hypothetical protein	ko00660//C5-Branched dibasic acid metabolism	-	GO:0016829//lyase activity	-	gi|115399114|ref|XP_001215146.1|/0/predicted protein [Aspergillus terreus NIH2624]
4316270	1068	88.56	267.82	2.186023532	8.07E-12	9.50E-11	Up	ATEG_01758	similar to tartrate dehydrogenase	ko01100//Metabolic pathways;ko01130//Biosynthesis of antibiotics;ko01230//Biosynthesis of amino acids;ko01210//2-Oxocarboxylic acid metabolism;ko00300//Lysine biosynthesis	-	GO:0000287//magnesium ion binding;GO:0016616//oxidoreductase activity, acting on the CH-OH group of donors, NAD or NADP as acceptor;GO:0051287//NAD binding	GO:0055114//oxidation-reduction process	gi|115385152|ref|XP_001209123.1|/0/hypothetical protein ATEG_01758 [Aspergillus terreus NIH2624]
4354964	1065	113.76	341.78	2.183491472	1.96E-16	3.16E-15	Up	ATEG_00215	hypothetical protein	ko01100//Metabolic pathways;ko01220//Degradation of aromatic compounds	-	GO:0047575;GO:0051920//peroxiredoxin activity	GO:0055114//oxidation-reduction process;GO:0098869//cellular oxidant detoxification	gi|115491347|ref|XP_001210301.1|/0/conserved hypothetical protein [Aspergillus terreus NIH2624]

4320220	2298	184.93	549.66	2.182669463	8.64E-22	1.95E-20	Up	ATEG_04658	hypothetical protein	ko04111//Cell cycle - yeast	GO:0005634//nucleus;GO:0005737//cytoplasm	GO:0001077//transcriptional activator activity, RNA polymerase II core promoter proximal region sequence-specific binding;GO:0003676//nucleic acid binding;GO:0046872//metal ion binding	GO:0045944//positive regulation of transcription from RNA polymerase II promoter	gi|115396394|ref|XP_001213836.1|/0/conserved hypothetical protein [Aspergillus terreus NIH2624]
4318702	1584	5.04	22.7	2.179972385	0.00776207	0.02492522	Up	ATEG_03723	hypothetical protein	-	-	-	-	gi|115390793|ref|XP_001212901.1|/0/predicted protein [Aspergillus terreus NIH2624]
4317981	1335	20.16	67.05	2.174574781	7.04E-05	0.00036475	Up	ATEG_03507	hypothetical protein	ko03440//Homologous recombination	-	-	-	gi|115390360|ref|XP_001212685.1|/0/predicted protein [Aspergillus terreus NIH2624]
4353364	1686	8.97	33.51	2.168856046	0.00247303	0.00919718	Up	ATEG_08245	hypothetical protein	ko04113//Meiosis - yeast	GO:0016021//integral component of membrane	GO:0022891//substrate-specific transmembrane transporter activity	GO:0055085//transmembrane transport	gi|115433458|ref|XP_001216866.1|/0/conserved hypothetical protein [Aspergillus terreus NIH2624]
4322484	2040	6.72	27.15	2.16618574	0.00444382	0.01544881	Up	ATEG_06318	hypothetical protein	-	-	-	-	gi|115399814|ref|XP_001215496.1|/0/conserved hypothetical protein [Aspergillus terreus NIH2624]
4353699	1686	29.71	96.57	2.163085921	1.33E-05	7.80E-05	Up	ATEG_09076	similar to basic amino acid permease	-	GO:0016021//integral component of membrane	GO:0015171//amino acid transmembrane transporter activity	GO:0003333//amino acid transmembrane transport	gi|115436992|ref|XP_001217698.1|/0/hypothetical protein ATEG_09076 [Aspergillus terreus NIH2624]

4317330	1217	959.86	2774.57	2.157994964	3.47E-46	2.06E-44	Up	ATEG_02970	hypothetical protein	-	GO:0016021//integral component of membrane	-	GO:0055085//transmembrane transport	gi|115389286|ref|XP_001212148.1|/0/conserved hypothetical protein [Aspergillus terreus NIH2624]
4320301	2243	3279.81	9431.42	2.153310421	1.54E-62	1.49E-60	Up	ATEG_04362	hypothetical protein	ko01100//Metabolic pathways;ko00460//Cyanoamino acid metabolism;ko00480//Glutathione metabolism;ko00430//Taurine and hypotaurine metabolism	-	GO:0003840//gamma-glutamyltransferase activity	GO:0006749//glutathione metabolic process	gi|115395802|ref|XP_001213540.1|/0/conserved hypothetical protein [Aspergillus terreus NIH2624]
4315523	975	82.41	244.01	2.149992579	4.78E-11	5.24E-10	Up	ATEG_01034	hypothetical protein	-	GO:0005737//cytoplasm;GO:0016021//integral component of membrane	GO:0008677//2-dehydropantoate 2-reductase activity;GO:0050661//NADP binding	GO:0015940//pantothenate biosynthetic process;GO:0055114//oxidation-reduction process	gi|115383704|ref|XP_001208399.1|/0/conserved hypothetical protein [Aspergillus terreus NIH2624]
4320090	2760	146.78	430.31	2.14835002	4.54E-17	7.54E-16	Up	ATEG_04556	hypothetical protein	ko01100//Metabolic pathways;ko00500//Starch and sucrose metabolism	GO:0005826//actomyosin contractile ring;GO:0005829//cytosol	-	GO:1903475//mitotic actomyosin contractile ring assembly	gi|115396190|ref|XP_001213734.1|/0/conserved hypothetical protein [Aspergillus terreus NIH2624]
4320734	1782	462.92	1329.69	2.143717015	3.95E-38	1.84E-36	Up	ATEG_05300	ornithine aminotransferase	ko01100//Metabolic pathways;ko01110//Biosynthesis of secondary metabolites;ko01130//Biosynthesis of antibiotics;ko00330//Arginine and proline metabolism	-	GO:0004587//ornithine-oxo-acid transaminase activity;GO:0030170//pyridoxal phosphate binding	-	gi|115397773|ref|XP_001214478.1|/0/ornithine aminotransferase [Aspergillus terreus NIH2624]

4320012	1005	405.63	1164.03	2.141239892	4.43E-25	1.20E-23	Up	ATEG_04463	hypothetical protein	ko00620//Pyruvate metabolism;ko00640//Propanoate metabolism	-	GO:0003824//catalytic activity;GO:0050662//coenzyme binding	GO:0044237//cellular metabolic process	gi|115396004|ref|XP_001213641.1|/0/conserved hypothetical protein [Aspergillus terreus NIH2624]
4353278	612	24.1	75.79	2.138901611	2.51E-05	0.00014081	Up	ATEG_08444	hypothetical protein	ko01100//Metabolic pathways;ko01212//Fatty acid metabolism;ko00062//Fatty acid elongation	-	-	-	gi|115433857|ref|XP_001217065.1|/2.91011e-144/predicted protein [Aspergillus terreus NIH2624]
4318883	2100	77.88	228.74	2.137912311	5.26E-11	5.72E-10	Up	ATEG_07188	G2-specific protein kinase nimA	-	GO:0005635//nuclear envelope;GO:0005829//cytosol;GO:0005876//spindle microtubule;GO:0030428//cell septum;GO:0071958//new mitotic spindle pole body	GO:0004674//protein serine/threonine kinase activity;GO:0005524//ATP binding	GO:0010971//positive regulation of G2/M transition of mitotic cell cycle;GO:0031030//negative regulation of septation initiation signaling;GO:0043987//histone H3-S10 phosphorylation;GO:0090307//mitotic spindle assembly;GO:1903380//positive regulation of mitotic chromosome condensation	gi|115386666|ref|XP_001209874.1|/0/G2-specific protein kinase nimA [Aspergillus terreus NIH2624]
4322941	1826	1034.66	2947.89	2.130186559	6.99E-48	4.49E-46	Up	ATEG_07791	similar to fructose facilitator	-	GO:0016021//integral component of membrane	-	GO:0055085//transmembrane transport	gi|115401648|ref|XP_001216412.1|/0/hypothetical protein ATEG_07791 [Aspergillus terreus NIH2624]

4322604	411	427.54	1212.1	2.126545963	1.55E-27	4.76E-26	Up	ATEG_07881	hypothetical protein	-	-	GO:0016846//carbon-sulfur lyase activity	GO:0008152//metabolic process	gi|115401828|ref|XP_001216502.1|/3.53538e-101/conserved hypothetical protein [Aspergillus terreus NIH2624]
4354703	1605	5.6	22.76	2.124249142	0.00850486	0.02700342	Up	ATEG_10247	hypothetical protein	-	GO:0016021//integral component of membrane	GO:0005267//potassium channel activity	GO:0071805//potassium ion transmembrane transport	gi|115449393|ref|XP_001218595.1|/0/predicted protein [Aspergillus terreus NIH2624]
4354702	1538	154.73	444.23	2.122031166	4.12E-16	6.42E-15	Up	ATEG_10246	hypothetical protein	-	GO:0016021//integral component of membrane	-	-	gi|115449389|ref|XP_001218594.1|/0/predicted protein [Aspergillus terreus NIH2624]
4316578	1815	7.29	28.42	2.121655371	0.00583942	0.01949869	Up	ATEG_02032	hypothetical protein	-	-	-	-	gi|115387409|ref|XP_001211210.1|/0/predicted protein [Aspergillus terreus NIH2624]
4353697	1728	2077.64	5819.06	2.115645959	7.12E-51	4.87E-49	Up	ATEG_09074	hypothetical protein	ko01100//Metabolic pathways;ko00260//Glycine, serine and threonine metabolism	-	GO:0016614//oxidoreductase activity, acting on CH-OH group of donors;GO:0050660//flavin adenine dinucleotide binding	GO:0055114//oxidation-reduction process	gi|115436984|ref|XP_001217696.1|/0/conserved hypothetical protein [Aspergillus terreus NIH2624]

4321676	1488	327.21	921.23	2.109774262	4.99E-28	1.56E-26	Up	ATEG_05887	similar to : alanine aminotransferase	ko01100//Metabolic pathways;ko01230//Biosynthesis of amino acids;ko01200//Carbon metabolism;ko00250//Alanine, aspartate and glutamate metabolism;ko01210//2-Oxocarboxylic acid metabolism;ko00220//Arginine biosynthesis	-	GO:0008483//transaminase activity;GO:0030170//pyridoxal phosphate binding	GO:0009058//biosynthetic process	gi|115398952|ref|XP_001215065.1|/0/hypothetical protein ATEG_05887 [Aspergillus terreus NIH2624]
4319376	501	47.07	139.32	2.108546917	5.87E-08	4.77E-07	Up	ATEG_06890	hypothetical protein	-	-	-	-	gi|115386070|ref|XP_001209576.1|/1.0238e-119/predicted protein [Aspergillus terreus NIH2624]
4323161	1015	40.91	121.63	2.107666926	2.62E-07	1.97E-06	Up	ATEG_08897	hypothetical protein	-	-	-	-	gi|115402813|ref|XP_001217483.1|/4.75665e-135/predicted protein [Aspergillus terreus NIH2624]
4322402	702	530.54	1484.49	2.105075657	6.75E-28	2.09E-26	Up	ATEG_06485	hypothetical protein	ko01100//Metabolic pathways;ko00520//Amino sugar and nucleotide sugar metabolism	-	-	-	gi|115400149|ref|XP_001215663.1|/7.80893e-157/predicted protein [Aspergillus terreus NIH2624]
4317475	1935	8.4	30.7	2.104684618	0.00368939	0.01308298	Up	ATEG_02921	hypothetical protein	-	-	-	-	gi|115389188|ref|XP_001212099.1|/0/conserved hypothetical protein [Aspergillus terreus NIH2624]

4321781	3227	503.32	1404.47	2.104469312	3.47E-32	1.27E-30	Up	ATEG_05686	leucyl-tRNA synthetase, mitochondrial precursor	ko00970//Aminoacyl-tRNA biosynthesis	-	GO:0002161//aminoacyl-tRNA editing activity;GO:0004823//leucine-tRNA ligase activity;GO:0005524//ATP binding	GO:0006429//leucyl-tRNA aminoacylation;GO:0006450//regulation of translational fidelity	gi|115398550|ref|XP_001214864.1|/0/leucyl-tRNA synthetase, mitochondrial precursor [Aspergillus terreus NIH2624]
4322719	1467	49.91	146.91	2.102740776	7.65E-07	5.36E-06	Up	ATEG_07889	hypothetical protein	-	GO:0005774//vacuolar membrane;GO:0016021//integral component of membrane	-	GO:0006865//amino acid transport;GO:0006914//autophagy	gi|166989551|sp|Q0CEJ5.2|AT221_ASPTN/0/RecName: Full=Autophagy-related protein 22-1
4317599	1485	621	1752.83	2.10237449	3.92E-30	1.34E-28	Up	ATEG_03394	similar to lbr protein	ko01100//Metabolic pathways;ko01110//Biosynthesis of secondary metabolites;ko01130//Biosynthesis of antibiotics;ko00100//Steroid biosynthesis	GO:0016021//integral component of membrane	GO:0016628//oxidoreductase activity, acting on the CH-CH group of donors, NAD or NADP as acceptor	GO:0055114//oxidation-reduction process	gi|115390134|ref|XP_001212572.1|/0/hypothetical protein ATEG_03394 [Aspergillus terreus NIH2624]
4354635	1332	22.43	69.56	2.10021205	8.80E-05	0.00044607	Up	ATEG_10337	hypothetical protein	ko01100//Metabolic pathways;ko00561//Glycerolipid metabolism	-	GO:0016787//hydrolase activity	-	gi|115449747|ref|XP_001218685.1|/0/conserved hypothetical protein [Aspergillus terreus NIH2624]
4323437	1926	72.32	207.86	2.095805665	5.77E-10	5.67E-09	Up	ATEG_08632	similar to multidrug transporter	-	GO:0016021//integral component of membrane	-	GO:0055085//transmembrane transport	gi|115402283|ref|XP_001217218.1|/0/hypothetical protein ATEG_08632 [Aspergillus terreus NIH2624]

4319627	1215	6.16	24.05	2.092959944	0.00907751	0.02848639	Up	ATEG_09986	hypothetical protein	-	GO:0005634//nucleus	GO:0000981//RNA polymerase II transcription factor activity, sequence-specific DNA binding;GO:0008270//zinc ion binding	GO:0006357//regulation of transcription from RNA polymerase II promoter	gi|115385483|ref|XP_001209288.1|/0/predicted protein [Aspergillus terreus NIH2624]
4353275	1677	399.07	1110.9	2.091469777	3.17E-31	1.12E-29	Up	ATEG_08441	hypothetical protein	-	GO:0016021//integral component of membrane	GO:0004497//monooxygenase activity;GO:0005506//iron ion binding;GO:0016705//oxidoreductase activity, acting on paired donors, with incorporation or reduction of molecular oxygen;GO:0020037//heme binding	GO:0055114//oxidation-reduction process	gi|115433851|ref|XP_001217062.1|/0/predicted protein [Aspergillus terreus NIH2624]
4321375	2166	21.86	67.77	2.083775035	9.10E-05	0.00046002	Up	ATEG_05973	hypothetical protein	ko04113//Meiosis - yeast	GO:0016021//integral component of membrane	GO:0022891//substrate-specific transmembrane transporter activity	GO:0055085//transmembrane transport	gi|115399124|ref|XP_001215151.1|/0/conserved hypothetical protein [Aspergillus terreus NIH2624]
4315738	1840	115.42	323.31	2.077484773	3.96E-14	5.42E-13	Up	ATEG_01395	hypothetical protein	ko00600//Sphingolipid metabolism	GO:0016021//integral component of membrane	GO:0016811//hydrolase activity, acting on carbon-nitrogen (but not peptide) bonds, in linear amides	GO:0006672//ceramide metabolic process	gi|115384426|ref|XP_001208760.1|/0/conserved hypothetical protein [Aspergillus terreus NIH2624]
4319610	1242	17.94	56.21	2.076708013	0.00031962	0.00146936	Up	ATEG_09964	similar to transesterase	-	-	GO:0016746//transferase activity, transferring acyl groups;GO:0016787//hydrolase activity	GO:0017000//antibiotic biosynthetic process;GO:0030639//polyketide biosynthetic process;GO:0050832//defense response to fungus	gi|115385439|ref|XP_001209266.1|/0/hypothetical protein ATEG_09964 [Aspergillus terreus NIH2624]

4318743	3471	68.92	197.39	2.076269053	1.81E-09	1.70E-08	Up	ATEG_03675	hypothetical protein	ko04111//Cell cycle - yeast	GO:0005634//nucleus;GO:0005829//cytosol	-	-	gi|115390697|ref|XP_001212853.1|/0/predicted protein [Aspergillus terreus NIH2624]
4318835	3874	1689.17	4588.32	2.072705942	1.63E-38	7.74E-37	Up	ATEG_07250	hypothetical protein	ko04144//Endocytosis	-	GO:0003824//catalytic activity	-	gi|115386790|ref|XP_001209936.1|/0/predicted protein [Aspergillus terreus NIH2624]
4353955	1083	549.11	1496.83	2.071872271	4.31E-24	1.09E-22	Up	ATEG_09301	hypothetical protein	-	-	GO:0016491//oxidoreductase activity	GO:0055114//oxidation-reduction process	gi|115437878|ref|XP_001217923.1|/0/conserved hypothetical protein [Aspergillus terreus NIH2624]
4320914	762	189.42	521.58	2.0713422	1.42E-12	1.77E-11	Up	ATEG_05247	lactam utilization protein lamB	-	-	GO:0003824//catalytic activity	GO:0005975//carbohydrate metabolic process	gi|115397667|ref|XP_001214425.1|/0/lactam utilization protein lamB [Aspergillus terreus NIH2624]
4323276	543	6.72	25.84	2.068453303	0.00831651	0.02648805	Up	ATEG_08557	hypothetical protein	-	GO:0016020//membrane	-	-	gi|115402133|ref|XP_001217143.1|/3.77821e-131/conserved hypothetical protein [Aspergillus terreus NIH2624]
4317526	906	22.99	70.33	2.067469259	0.0001286	0.0006321	Up	ATEG_03425	hypothetical protein	ko01100//Metabolic pathways;ko00520//Amino sugar and nucleotide sugar metabolism	GO:0016021//integral component of membrane	-	-	gi|115390196|ref|XP_001212603.1|/0/predicted protein [Aspergillus terreus NIH2624]
4322037	973	86.3	242.06	2.067336456	1.26E-11	1.46E-10	Up	ATEG_06553	hypothetical protein	ko00051//Fructose and mannose metabolism	-	-	-	gi|115400285|ref|XP_001215731.1|/1.19394e-140/conserved hypothetical protein [Aspergillus terreus NIH2624]

4317747	1413	1459.13	3999.28	2.06622379	2.09E-34	8.33E-33	Up	ATEG_03484	N amino acid transport system protein	ko01100//Metabolic pathways;ko01110//Biosynthesis of secondary metabolites;ko00770//Pantothenate and CoA biosynthesis	GO:0016021//integral component of membrane	-	-	gi|115390314|ref|XP_001212662.1|/0/N amino acid transport system protein [Aspergillus terreus NIH2624]
4353731	2462	15262.56	41563.6	2.065935161	1.66E-60	1.52E-58	Up	ATEG_09404	hypothetical protein	ko01100//Metabolic pathways;ko00230//Purine metabolism;ko00240//Pyrimidine metabolism;ko03020//RNA polymerase	GO:0005736//DNA-directed RNA polymerase I complex	GO:0001054//RNA polymerase I activity;GO:0003677//DNA binding	GO:0006362//transcription elongation from RNA polymerase I promoter;GO:0042790//transcription of nuclear large rRNA transcript from RNA polymerase I promoter	gi|115438280|ref|XP_001218026.1|/0/conserved hypothetical protein [Aspergillus terreus NIH2624]
4316834	1029	5.61	21.93	2.062465698	0.01141817	0.03478464	Up	ATEG_02610	hypothetical protein	ko00254//Aflatoxin biosynthesis	-	GO:0016787//hydrolase activity	GO:0008152//metabolic process	gi|115388565|ref|XP_001211788.1|/0/conserved hypothetical protein [Aspergillus terreus NIH2624]
4321172	1041	40.34	115.99	2.061198896	8.89E-07	6.15E-06	Up	ATEG_05081	hypothetical protein	ko01100//Metabolic pathways;ko00500//Starch and sucrose metabolism	GO:0005576//extracellular region	GO:0004553//hydrolase activity, hydrolyzing O-glycosyl compounds;GO:0030248//cellulose binding	GO:0005975//carbohydrate metabolic process	gi|115397335|ref|XP_001214259.1|/0/conserved hypothetical protein [Aspergillus terreus NIH2624]
4322878	1464	9.52	33.02	2.059548937	0.00488179	0.01674191	Up	ATEG_07851	hypothetical protein	-	-	GO:0016491//oxidoreductase activity	GO:0055114//oxidation-reduction process	gi|115401768|ref|XP_001216472.1|/0/conserved hypothetical protein [Aspergillus terreus NIH2624]

4355292	1743	29.13	87.29	2.059140496	3.21E-05	0.00017688	Up	ATEG_00538	hypothetical protein	ko01100//Metabolic pathways;ko00230//Purine metabolism;ko00250//Alanine, aspartate and glutamate metabolism	GO:0016021//integral component of membrane	-	GO:0055085//transmembrane transport	gi|115491993|ref|XP_001210624.1|/0/conserved hypothetical protein [Aspergillus terreus NIH2624]
4317629	1773	57.21	164.12	2.058972023	2.47E-06	1.63E-05	Up	ATEG_03098	dihydroxy-acid dehydratase	ko01100//Metabolic pathways;ko01110//Biosynthesis of secondary metabolites;ko01130//Biosynthesis of antibiotics;ko01230//Biosynthesis of amino acids;ko01210//2-Oxocarboxylic acid metabolism;ko00770//Pantothenate and CoA biosynthesis;ko00290//Valine, leucine and isoleucine biosynthesis	-	GO:0004160//dihydroxy-acid dehydratase activity	GO:0009082//branched-chain amino acid biosynthetic process	gi|115389542|ref|XP_001212276.1|/0/dihydroxy-acid dehydratase [Aspergillus terreus NIH2624]
4317856	1335	8.41	30.36	2.058311602	0.00566286	0.01897666	Up	ATEG_03092	hypothetical protein	-	-	GO:0050364	GO:0009820//alkaloid metabolic process;GO:0019748//secondary metabolic process;GO:0044249//cellular biosynthetic process;GO:0044711;GO:0044763;GO:1901576	gi|115389530|ref|XP_001212270.1|/0/predicted protein [Aspergillus terreus NIH2624]

4316141	450	1889.15	5084.01	2.054137352	2.05E-62	1.94E-60	Up	ATEG_01528	40S ribosomal protein S14	ko03010//Ribosome	GO:0022627//cytosolic small ribosomal subunit	GO:0003735//structural constituent of ribosome;GO:0048027//mRNA 5'-UTR binding;GO:0070181//small ribosomal subunit rRNA binding	GO:0000028//ribosomal small subunit assembly;GO:0000462//maturation of SSU-rRNA from tricistronic rRNA transcript (SSU-rRNA, 5.8S rRNA, LSU-rRNA);GO:0006412//translation	gi|115384692|ref|XP_001208893.1|/1.39059e-102/40S ribosomal protein S14 [Aspergillus terreus NIH2624]
4317639	1155	94.12	263.53	2.051368132	1.51E-10	1.59E-09	Up	ATEG_02879	hypothetical protein	-	-	-	-	gi|115389104|ref|XP_001212057.1|/7.81531e-139/conserved hypothetical protein [Aspergillus terreus NIH2624]
4318879	1719	23.56	71.13	2.049940693	0.00017326	0.00083543	Up	ATEG_06881	pyruvate decarboxylase	ko01100//Metabolic pathways;ko01110//Biosynthesis of secondary metabolites;ko01130//Biosynthesis of antibiotics;ko00010//Glycolysis / Gluconeogenesis	-	GO:0000287//magnesium ion binding;GO:0016831//carboxy-lyase activity;GO:0030976//thiamine pyrophosphate binding	-	gi|115386052|ref|XP_001209567.1|/0/pyruvate decarboxylase [Aspergillus terreus NIH2624]
4319407	1312	81.85	226.87	2.049672504	1.83E-10	1.89E-09	Up	ATEG_10104	hypothetical protein	-	-	-	-	gi|115385719|ref|XP_001209406.1|/0/conserved hypothetical protein [Aspergillus terreus NIH2624]
4354783	1500	85.71	240.42	2.049289042	8.91E-10	8.63E-09	Up	ATEG_00027	hypothetical protein	-	GO:0016021//integral component of membrane	GO:0005215//transporter activity	GO:0055085//transmembrane transport	gi|115490971|ref|XP_001210113.1|/0/conserved hypothetical protein [Aspergillus terreus NIH2624]

4354173	3859	424.12	1137.5	2.043085513	3.10E-27	9.42E-26	Up	ATEG_09642	hypothetical protein	-	-	-	GO:0006355//regulation of transcription, DNA-templated	gi|115442914|ref|XP_001218264.1|/0/conserved hypothetical protein [Aspergillus terreus NIH2624]
4322271	918	421.4	1147.44	2.039131283	7.54E-21	1.62E-19	Up	ATEG_06523	hypothetical protein	ko01100//Metabolic pathways;ko01200//Carbon metabolism;ko00280//Valine, leucine and isoleucine degradation;ko00640//Propanoate metabolism;ko00562//Inositol phosphate metabolism;ko00410//beta-Alanine metabolism	-	-	-	gi|115400225|ref|XP_001215701.1|/0/conserved hypothetical protein [Aspergillus terreus NIH2624]
4354163	1551	114.92	312.3	2.034446646	8.60E-09	7.55E-08	Up	ATEG_09785	similar to phenylacetate hydroxylase	ko01100//Metabolic pathways;ko00360//Phenylalanine metabolism	-	GO:0004497//monooxygenase activity;GO:0005506//iron ion binding;GO:0016705//oxidoreductase activity, acting on paired donors, with incorporation or reduction of molecular oxygen;GO:0020037//heme binding	GO:0055114//oxidation-reduction process	gi|115443200|ref|XP_001218407.1|/0/hypothetical protein ATEG_09785 [Aspergillus terreus NIH2624]
4320221	465	2723.51	7263.35	2.03403089	1.01E-53	7.62E-52	Up	ATEG_04659	ubiquitin-40S ribosomal protein S31 fusion protein	ko03010//Ribosome	GO:0005840//ribosome	GO:0003735//structural constituent of ribosome	GO:0006412//translation	gi|115396396|ref|XP_001213837.1|/6.81374e-110/ubiquitin-40S ribosomal protein S31 fusion protein [Aspergillus terreus NIH2624]

4354827	1029	39.22	110.5	2.031925334	1.92E-06	1.28E-05	Up	ATEG_00071	hypothetical protein	-	-	GO:0008270//zinc ion binding;GO:0016491//oxidoreductase activity	GO:0055114//oxidation-reduction process	gi|115491059|ref|XP_001210157.1|/0/conserved hypothetical protein [Aspergillus terreus NIH2624]
4321894	2023	10.08	33.33	2.028321692	0.00338466	0.01212513	Up	ATEG_06368	hypothetical protein	ko03013//RNA transport	-	-	-	gi|115399914|ref|XP_001215546.1|/0/predicted protein [Aspergillus terreus NIH2624]
4355425	1452	82.43	226.04	2.025843256	4.27E-09	3.89E-08	Up	ATEG_00670	hypothetical protein	-	GO:0005634//nucleus	GO:0003677//DNA binding;GO:0046872//metal ion binding	GO:0006355//regulation of transcription, DNA-templated	gi|115492257|ref|XP_001210756.1|/0/predicted protein [Aspergillus terreus NIH2624]
4315621	1269	72.25	196.76	2.012864498	2.72E-08	2.27E-07	Up	ATEG_01012	hypothetical protein	-	GO:0005874//microtubule	GO:0003777//microtubule motor activity;GO:0005524//ATP binding;GO:0008017//microtubule binding	GO:0007018//microtubule-based movement	gi|115383660|ref|XP_001208377.1|/0/conserved hypothetical protein [Aspergillus terreus NIH2624]
4355619	1467	230.81	606.48	2.005196244	2.38E-17	4.01E-16	Up	ATEG_00858	hypothetical protein	-	GO:0005737//cytoplasm	GO:0004177//aminopeptidase activity;GO:0008237//metallopeptidase activity;GO:0030145//manganese ion binding	GO:0006508//proteolysis;GO:0009987//cellular process	gi|115492633|ref|XP_001210944.1|/0/conserved hypothetical protein [Aspergillus terreus NIH2624]
4321208	663	94.62	260.67	2.004974918	1.43E-07	1.11E-06	Up	ATEG_04943	endo-1,4-beta-xylanase B precursor	-	GO:0005576//extracellular region	GO:0031176//endo-1,4-beta-xylanase activity	GO:0045493//xylan catabolic process	gi|115397059|ref|XP_001214121.1|/1.96072e-164/endo-1,4-beta-xylanase B precursor [Aspergillus terreus NIH2624]

4321006	777	240.87	634.9	1.998541512	5.38E-17	8.87E-16	Up	ATEG_05407	hypothetical protein	ko01100//Metabolic pathways;ko00260//Glycine, serine and threonine metabolism;ko00240//Pyrimidine metabolism	-	GO:0016491//oxidoreductase activity	GO:0055114//oxidation-reduction process	gi|115397987|ref|XP_001214585.1|/0/conserved hypothetical protein [Aspergillus terreus NIH2624]
4322194	885	552.05	1425.32	1.994878762	7.66E-27	2.27E-25	Up	ATEG_06511	L-xylulose reductase	ko00051//Fructose and mannose metabolism	-	GO:0016491//oxidoreductase activity	GO:0055114//oxidation-reduction process	gi|115400201|ref|XP_001215689.1|/0/L-xylulose reductase [Aspergillus terreus NIH2624]
4321396	852	49.29	134.9	1.987449031	1.30E-06	8.84E-06	Up	ATEG_06115	protein rho4	-	GO:0000935//barrier septum;GO:0005938//cell cortex	GO:0003924//GTPase activity;GO:0005525//GTP binding	GO:0000226//microtubule cytoskeleton organization;GO:0007264//small GTPase mediated signal transduction;GO:0008360//regulation of cell shape;GO:0032955//regulation of barrier septum assembly;GO:0032956//regulation of actin cytoskeleton organization;GO:0034613//cellular protein localization	gi|115399408|ref|XP_001215293.1|/0/protein rho4 [Aspergillus terreus NIH2624]
4316199	5165	1059.17	2728.78	1.986625182	1.62E-46	9.85E-45	Up	ATEG_01420	similar to multiple drug resistance protein	-	GO:0016021//integral component of membrane	-	GO:0055085//transmembrane transport	gi|115384476|ref|XP_001208785.1|/0/hypothetical protein ATEG_01420 [Aspergillus terreus NIH2624]

4319304	660	752.52	1928.55	1.985082644	5.38E-30	1.82E-28	Up	ATEG_06979	hypothetical protein	ko04144//Endocytosis	-	-	-	gi|115386248|ref|XP_001209665.1|/1.04291e-154/conserved hypothetical protein [Aspergillus terreus NIH2624]
4319134	729	28.01	78.72	1.982679579	7.75E-05	0.00039803	Up	ATEG_07179	hypothetical protein	ko04011//MAPK signaling pathway - yeast	-	GO:0004672//protein kinase activity;GO:0005524//ATP binding	GO:0006468//protein phosphorylation	gi|115386648|ref|XP_001209865.1|/1.21994e-178/predicted protein [Aspergillus terreus NIH2624]
4321620	1948	98.59	258.34	1.97669709	2.82E-10	2.86E-09	Up	ATEG_06194	hypothetical protein	ko01212//Fatty acid metabolism;ko01040//Biosynthesis of unsaturated fatty acids	GO:0016021//integral component of membrane	-	GO:0055085//transmembrane transport	gi|115399566|ref|XP_001215372.1|/0/conserved hypothetical protein [Aspergillus terreus NIH2624]
4318064	6494	476.8	1215.59	1.969836187	1.56E-28	4.95E-27	Up	ATEG_03006	hypothetical protein	ko03013//RNA transport;ko04011//MAPK signaling pathway - yeast	-	-	-	gi|115389358|ref|XP_001212184.1|/0/predicted protein [Aspergillus terreus NIH2624]
4321090	1638	10.08	31.99	1.968641451	0.00487899	0.01673841	Up	ATEG_05210	hypothetical protein	ko01100//Metabolic pathways;ko00520//Amino sugar and nucleotide sugar metabolism	-	-	-	gi|115397593|ref|XP_001214388.1|/0/conserved hypothetical protein [Aspergillus terreus NIH2624]
4355602	1782	91.89	239.56	1.967354621	8.67E-11	9.30E-10	Up	ATEG_00841	hypothetical protein	ko03013//RNA transport;ko04011//MAPK signaling pathway - yeast	-	-	-	gi|115492599|ref|XP_001210927.1|/0/predicted protein [Aspergillus terreus NIH2624]
4317104	1425	3563.07	8996.58	1.959046776	3.19E-53	2.36E-51	Up	ATEG_02179	similar to short chain dehydrogenase	ko00051//Fructose and mannose metabolism	-	-	-	gi|115387703|ref|XP_001211357.1|/0/hypothetical protein ATEG_02179 [Aspergillus terreus NIH2624]

4320233	780	38.1	102.11	1.955041921	7.33E-06	4.43E-05	Up	ATEG_04861	hypothetical protein	ko04144//Endocytosis	-	-	-	gi|115396800|ref|XP_001214039.1|/0/predicted protein [Aspergillus terreus NIH2624]
4316527	816	163.7	417.07	1.953775912	7.00E-14	9.40E-13	Up	ATEG_01970	hypothetical protein	ko03440//Homologous recombination	GO:0016021//integral component of membrane	-	-	gi|115387285|ref|XP_001211148.1|/0/conserved hypothetical protein [Aspergillus terreus NIH2624]
4317223	1578	7.86	26.5	1.947959982	0.01249105	0.03755321	Up	ATEG_02693	hypothetical protein	-	GO:0016021//integral component of membrane	GO:0046873//metal ion transmembrane transporter activity	GO:0030001//metal ion transport;GO:0055085//transmembrane transport	gi|115388731|ref|XP_001211871.1|/0/predicted protein [Aspergillus terreus NIH2624]
4321777	1104	56.08	148.54	1.94746546	3.44E-06	2.22E-05	Up	ATEG_05682	hypothetical protein	-	GO:0005743//mitochondrial inner membrane	GO:0003924//GTPase activity;GO:0005525//GTP binding	GO:0070899//mitochondrial tRNA wobble uridine modification	gi|115398542|ref|XP_001214860.1|/0/conserved hypothetical protein [Aspergillus terreus NIH2624]
4321686	1587	120.41	308.96	1.946093333	1.24E-08	1.06E-07	Up	ATEG_05978	hypothetical protein	ko04113//Meiosis - yeast	GO:0016021//integral component of membrane	GO:0022891//substrate-specific transmembrane transporter activity	GO:0055085//transmembrane transport	gi|115399134|ref|XP_001215156.1|/0/conserved hypothetical protein [Aspergillus terreus NIH2624]
4354044	1275	11.21	34.25	1.942797282	0.00430237	0.01501829	Up	ATEG_09056	hypothetical protein	-	GO:0016021//integral component of membrane	-	-	gi|115436918|ref|XP_001217678.1|/0/predicted protein [Aspergillus terreus NIH2624]
4317934	1506	285.85	716.88	1.939397991	4.10E-22	9.50E-21	Up	ATEG_03554	hypothetical protein	ko00920//Sulfur metabolism	-	GO:0016491//oxidoreductase activity	GO:0055114//oxidation-reduction process	gi|115390454|ref|XP_001212732.1|/0/conserved hypothetical protein [Aspergillus terreus NIH2624]

4317100	3634	31.94	85.24	1.937320826	4.15E-05	0.00022499	Up	ATEG_02134	hypothetical protein	-	GO:0016021//integral component of membrane	-	-	gi|115387613|ref|XP_001211312.1|/0/conserved hypothetical protein [Aspergillus terreus NIH2624]
4318703	1080	6.17	21.71	1.935723058	0.01649243	0.04790976	Up	ATEG_03724	hypothetical protein	ko01100//Metabolic pathways;ko01230//Biosynthesis of amino acids;ko00250//Alanine, aspartate and glutamate metabolism;ko00630//Glyoxylate and dicarboxylate metabolism;ko00220//Arginine biosynthesis;ko00910//Nitrogen metabolism	-	GO:0004356//glutamate-ammonia ligase activity	GO:0006807//nitrogen compound metabolic process	gi|115390795|ref|XP_001212902.1|/0/predicted protein [Aspergillus terreus NIH2624]
4317125	1005	651.71	1607.05	1.93002698	3.50E-26	1.01E-24	Up	ATEG_02504	aldehyde reductase I (Alcohol dehydrogenase [NADP+])	ko01100//Metabolic pathways;ko00040//Pentose and glucuronate interconversions	-	GO:0016491//oxidoreductase activity	GO:0055114//oxidation-reduction process	gi|115388353|ref|XP_001211682.1|/0/aldehyde reductase I (Alcohol dehydrogenase [NADP+]) [Aspergillus terreus NIH2624]
4316390	2753	40.31	107.6	1.926056254	4.77E-05	0.00025381	Up	ATEG_01243	hypothetical protein	ko01100//Metabolic pathways;ko00500//Starch and sucrose metabolism	GO:0016021//integral component of membrane	-	-	gi|115384122|ref|XP_001208608.1|/0/conserved hypothetical protein [Aspergillus terreus NIH2624]
4318042	863	112.03	282.3	1.925116802	6.39E-10	6.25E-09	Up	ATEG_03214	hypothetical protein	ko01100//Metabolic pathways;ko00520//Amino sugar and nucleotide sugar metabolism	-	GO:0016787//hydrolase activity	-	gi|115389774|ref|XP_001212392.1|/2.98798e-137/conserved hypothetical protein [Aspergillus terreus NIH2624]

4322305	3746	202.29	502.18	1.921704711	5.74E-18	1.01E-16	Up	ATEG_06727	hypothetical protein	-	GO:0005634//nucleus;GO:0016020//membrane	GO:0003677//DNA binding;GO:0008270//zinc ion binding	GO:0006351//transcription, DNA-templated	gi|115400633|ref|XP_001215905.1|/0/conserved hypothetical protein [Aspergillus terreus NIH2624]
4321685	4080	15.13	43.85	1.921412636	0.00211208	0.00800065	Up	ATEG_05977	similar to mutanase	-	-	GO:0016787//hydrolase activity	-	gi|115399132|ref|XP_001215155.1|/0/hypothetical protein ATEG_05977 [Aspergillus terreus NIH2624]
4354259	1707	11.78	35.69	1.920639169	0.00525903	0.01783356	Up	ATEG_09802	hypothetical protein	ko01100//Metabolic pathways;ko00500//Starch and sucrose metabolism	-	GO:0008810//cellulase activity	GO:0005975//carbohydrate metabolic process	gi|115443234|ref|XP_001218424.1|/0/conserved hypothetical protein [Aspergillus terreus NIH2624]
4321372	1164	219.61	544.81	1.920615933	1.71E-16	2.75E-15	Up	ATEG_05970	hypothetical protein	ko01100//Metabolic pathways;ko00500//Starch and sucrose metabolism	-	GO:0016491//oxidoreductase activity	GO:0055114//oxidation-reduction process	gi|115399118|ref|XP_001215148.1|/0/conserved hypothetical protein [Aspergillus terreus NIH2624]
4319573	915	7.85	25.7	1.916651295	0.01289292	0.03853913	Up	ATEG_10139	hypothetical protein	ko01100//Metabolic pathways;ko00510//N-Glycan biosynthesis;ko00513//Various types of N-glycan biosynthesis	-	-	-	gi|115385789|ref|XP_001209441.1|/0/conserved hypothetical protein [Aspergillus terreus NIH2624]
4323491	4134	697.18	1708.22	1.916122286	9.26E-37	4.12E-35	Up	ATEG_08721	hypothetical protein	-	-	-	-	gi|115402461|ref|XP_001217307.1|/0/conserved hypothetical protein [Aspergillus terreus NIH2624]

4353606	906	49.85	128.75	1.914304164	2.77E-06	1.81E-05	Up	ATEG_09384	hypothetical protein	ko04144//Endocytosis	-	-	-	gi|115438204|ref|XP_001218006.1|/0/conserved hypothetical protein [Aspergillus terreus NIH2624]
4323540	633	38.09	99.51	1.911544622	1.90E-05	0.00010856	Up	ATEG_08517	hypothetical protein	-	-	GO:0035241//protein-arginine omega-N monomethyltransferase activity	GO:0035247//peptidyl-arginine omega-N-methylation	gi|115402053|ref|XP_001217103.1|/1.01317e-152/conserved hypothetical protein [Aspergillus terreus NIH2624]
4319572	1634	50.47	129.18	1.907412578	6.72E-06	4.10E-05	Up	ATEG_10138	hypothetical protein	-	-	-	-	gi|115385787|ref|XP_001209440.1|/0/predicted protein [Aspergillus terreus NIH2624]
4353111	7540	998.51	2420.98	1.906194334	7.47E-33	2.78E-31	Up	ATEG_08138	hypothetical protein	ko04144//Endocytosis	-	GO:0008270//zinc ion binding	-	gi|115433244|ref|XP_001216759.1|/0/predicted protein [Aspergillus terreus NIH2624]
4322979	2160	15.71	45	1.905794909	0.00296483	0.01079091	Up	ATEG_07458	hypothetical protein	ko00620//Pyruvate metabolism	GO:0005634//nucleus	GO:0000981//RNA polymerase II transcription factor activity, sequence-specific DNA binding;GO:0003677//DNA binding;GO:0008270//zinc ion binding	GO:0006357//regulation of transcription from RNA polymerase II promoter	gi|115400982|ref|XP_001216079.1|/0/predicted protein [Aspergillus terreus NIH2624]
4354182	1677	153.6	381.18	1.903820186	1.78E-12	2.20E-11	Up	ATEG_09663	hypothetical protein	ko03440//Homologous recombination;ko03450//Non-homologous end-joining	GO:0016021//integral component of membrane	-	GO:0055085//transmembrane transport	gi|115442956|ref|XP_001218285.1|/0/conserved hypothetical protein [Aspergillus terreus NIH2624]

4353470	1644	189.4	460.68	1.896243172	1.11E-13	1.47E-12	Up	ATEG_08418	hypothetical protein	-	-	GO:0016810//hydrolase activity, acting on carbon-nitrogen (but not peptide) bonds	-	gi|115433805|ref|XP_001217039.1|/0/conserved hypothetical protein [Aspergillus terreus NIH2624]
4322748	1281	31.95	82.61	1.890051974	7.78E-05	0.00039918	Up	ATEG_07463	hypothetical protein	-	-	-	-	gi|115400992|ref|XP_001216084.1|/1.35034e-94/conserved hypothetical protein [Aspergillus terreus NIH2624]
4320930	948	10.08	30.7	1.888153882	0.00885202	0.02792821	Up	ATEG_05179	hypothetical protein	-	-	GO:0016787//hydrolase activity	-	gi|115397531|ref|XP_001214357.1|/0/conserved hypothetical protein [Aspergillus terreus NIH2624]
4354412	351	1234.94	2967.43	1.884996431	8.56E-39	4.12E-37	Up	ATEG_09590	60S ribosomal protein L34	ko03010//Ribosome	GO:0022625//cytosolic large ribosomal subunit	GO:0003723//RNA binding;GO:0003735//structural constituent of ribosome	GO:0006412//translation;GO:0042254//ribosome biogenesis	gi|115442810|ref|XP_001218212.1|/6.31298e-81/60S ribosomal protein L34 [Aspergillus terreus NIH2624]
4316273	1620	337.99	811.27	1.883994401	7.86E-20	1.59E-18	Up	ATEG_01761	hypothetical protein	ko01100//Metabolic pathways;ko00650//Butanoate metabolism;ko00250//Alanine, aspartate and glutamate metabolism	-	GO:0016620//oxidoreductase activity, acting on the aldehyde or oxo group of donors, NAD or NADP as acceptor	GO:0055114//oxidation-reduction process	gi|115385158|ref|XP_001209126.1|/0/conserved hypothetical protein [Aspergillus terreus NIH2624]
4317726	1752	12.9	37.65	1.871039451	0.00603669	0.02007166	Up	ATEG_02836	hypothetical protein	-	-	GO:0010181//FMN binding;GO:0016491//oxidoreductase activity	GO:0055114//oxidation-reduction process	gi|115389018|ref|XP_001212014.1|/0/conserved hypothetical protein [Aspergillus terreus NIH2624]

4353548	1823	4722.4	11145.99	1.870128583	6.58E-41	3.35E-39	Up	ATEG_08126	hypothetical protein	ko01100//Metabolic pathways;ko00520//Amino sugar and nucleotide sugar metabolism	-	-	GO:0055085//transmembrane transport	gi|115433220|ref|XP_001216747.1|/0/conserved hypothetical protein [Aspergillus terreus NIH2624]
4318631	969	35.28	90.37	1.869746744	0.0001044	0.00052106	Up	ATEG_03822	hypothetical protein	-	GO:0005634//nucleus;GO:0005829//cytosol	GO:0016788//hydrolase activity, acting on ester bonds	-	gi|115390991|ref|XP_001213000.1|/0/conserved hypothetical protein [Aspergillus terreus NIH2624]
4355570	3337	443.18	1051.98	1.865191598	2.57E-24	6.59E-23	Up	ATEG_00809	endo-1,4-beta-xylanase A precursor	-	-	GO:0031176//endo-1,4-beta-xylanase activity	GO:0045493//xylan catabolic process	gi|115492535|ref|XP_001210895.1|/0/endo-1,4-beta-xylanase A precursor [Aspergillus terreus NIH2624]
4355177	774	1486.97	3511.28	1.864076348	1.50E-36	6.59E-35	Up	ATEG_00423	hypothetical protein	ko04144//Endocytosis	GO:0016021//integral component of membrane	-	-	gi|115491763|ref|XP_001210509.1|/0/conserved hypothetical protein [Aspergillus terreus NIH2624]
4320055	525	101.39	246.25	1.862862364	4.88E-09	4.42E-08	Up	ATEG_04805	hypothetical protein	ko00350//Tyrosine metabolism	GO:0005634//nucleus;GO:0005829//cytosol	GO:0008080//N-acetyltransferase activity	-	gi|115396688|ref|XP_001213983.1|/1.22803e-124/conserved hypothetical protein [Aspergillus terreus NIH2624]
4317631	1739	521.79	1225.97	1.859713875	9.98E-16	1.52E-14	Up	ATEG_03100	acid phosphatase precursor	ko04111//Cell cycle - yeast	GO:0005576//extracellular region;GO:0009277//fungal-type cell wall	GO:0003993//acid phosphatase activity	-	gi|115389546|ref|XP_001212278.1|/1.44677e-104/acid phosphatase precursor [Aspergillus terreus NIH2624]

4319039	1752	58.83	143.6	1.855737216	4.24E-06	2.68E-05	Up	ATEG_06913	hypothetical protein	ko01100//Metabolic pathways;ko01110//Biosynthesis of secondary metabolites;ko04146//Peroxisome;ko00071//Fatty acid degradation;ko01212//Fatty acid metabolism;ko01040//Biosynthesis of unsaturated fatty acids;ko00592//alpha-Linolenic acid metabolism	-	GO:0003995//acyl-CoA dehydrogenase activity	GO:0055114//oxidation-reduction process	gi|115386116|ref|XP_001209599.1|/0/predicted protein [Aspergillus terreus NIH2624]
4354258	3261	107.6	256.05	1.845099424	1.80E-10	1.86E-09	Up	ATEG_09801	hypothetical protein	ko03060//Protein export	GO:0016021//integral component of membrane	-	-	gi|115443232|ref|XP_001218423.1|/0/conserved hypothetical protein [Aspergillus terreus NIH2624]
4317974	1170	405.34	951.09	1.842093492	1.04E-17	1.78E-16	Up	ATEG_03524	lysophospholipase precursor	ko00564//Glycerophospholipid metabolism	-	GO:0004622//lysophospholipase activity	GO:0009395//phospholipid catabolic process	gi|115390394|ref|XP_001212702.1|/0/lysophospholipase precursor [Aspergillus terreus NIH2624]
4318989	1562	3339.54	7772.57	1.841511873	8.60E-47	5.31E-45	Up	ATEG_07154	hypothetical protein	ko04144//Endocytosis	GO:0016021//integral component of membrane	GO:0008198//ferrous iron binding;GO:0051213//dioxygenase activity	GO:0006725//cellular aromatic compound metabolic process;GO:0055114//oxidation-reduction process	gi|115386598|ref|XP_001209840.1|/0/conserved hypothetical protein [Aspergillus terreus NIH2624]

4323035	1742	213.54	498.68	1.840374887	2.71E-13	3.51E-12	Up	ATEG_07898	hypothetical protein	ko01100//Metabolic pathways;ko01110//Biosynthesis of secondary metabolites	-	GO:0004497//monooxygenase activity;GO:0005506//iron ion binding;GO:0016705//oxidoreductase activity, acting on paired donors, with incorporation or reduction of molecular oxygen;GO:0020037//heme binding	GO:0055114//oxidation-reduction process	gi|115401862|ref|XP_001216519.1|/0/conserved hypothetical protein [Aspergillus terreus NIH2624]
4320097	1863	585.8	1356.61	1.837998874	4.71E-15	6.77E-14	Up	ATEG_04852	hypothetical protein	ko01100//Metabolic pathways;ko00640//Propanoate metabolism	-	GO:0016614//oxidoreductase activity, acting on CH-OH group of donors;GO:0050660//flavin adenine dinucleotide binding	GO:0055114//oxidation-reduction process	gi|115396782|ref|XP_001214030.1|/0/predicted protein [Aspergillus terreus NIH2624]
4321099	1626	322.32	752.98	1.836876072	7.24E-19	1.34E-17	Up	ATEG_05002	exoglucanase 1 precursor	ko01100//Metabolic pathways;ko00500//Starch and sucrose metabolism	GO:0005576//extracellular region	GO:0016162;GO:0016829//lyase activity;GO:0030248//cellulose binding	GO:0030245//cellulose catabolic process	gi|115397177|ref|XP_001214180.1|/0/exoglucanase 1 precursor [Aspergillus terreus NIH2624]
4353471	1671	5558.28	12820.8	1.835532334	1.03E-31	3.72E-30	Up	ATEG_08419	hypothetical protein	-	GO:0016021//integral component of membrane	GO:0015171//amino acid transmembrane transporter activity	GO:0003333//amino acid transmembrane transport	gi|115433807|ref|XP_001217040.1|/0/conserved hypothetical protein [Aspergillus terreus NIH2624]
4317759	1542	29.72	75.12	1.833969902	0.00038579	0.00173951	Up	ATEG_02813	hypothetical protein	-	-	-	-	gi|115388972|ref|XP_001211991.1|/0/predicted protein [Aspergillus terreus NIH2624]

4354781	3102	408.6	942.67	1.830368646	5.81E-20	1.19E-18	Up	ATEG_00025	hypothetical protein	-	GO:0005634//nucleus	GO:0000981//RNA polymerase II transcription factor activity, sequence-specific DNA binding;GO:0003677//DNA binding;GO:0008270//zinc ion binding	GO:0006357//regulation of transcription from RNA polymerase II promoter	gi|115490967|ref|XP_001210111.1|/0/conserved hypothetical protein [Aspergillus terreus NIH2624]
4323138	2215	6526.73	15038.81	1.829193289	1.08E-56	9.17E-55	Up	ATEG_08744	60S ribosomal protein L37	ko03040//Spliceosome	GO:0005840//ribosome;GO:0071021//U2-type post-spliceosomal complex	GO:0003735//structural constituent of ribosome	GO:0000350//generation of catalytic spliceosome for second transesterification step;GO:0006412//translation;GO:0045292//mRNA cis splicing, via spliceosome	gi|115402507|ref|XP_001217330.1|/0/60S ribosomal protein L37 [Aspergillus terreus NIH2624]
4355428	3296	62.2	149.07	1.82813775	5.38E-07	3.88E-06	Up	ATEG_00673	hypothetical protein	-	GO:0016021//integral component of membrane	-	GO:0055085//transmembrane transport	gi|115492263|ref|XP_001210759.1|/0/conserved hypothetical protein [Aspergillus terreus NIH2624]
4316609	1813	64.45	153.66	1.822224196	2.77E-05	0.00015416	Up	ATEG_02471	hypothetical protein	ko00650//Butanoate metabolism	-	GO:0008270//zinc ion binding;GO:0008743//L-threonine 3-dehydrogenase activity	GO:0006520//cellular amino acid metabolic process;GO:0055114//oxidation-reduction process	gi|115388287|ref|XP_001211649.1|/0/conserved hypothetical protein [Aspergillus terreus NIH2624]

4321226	1520	891.11	2037.47	1.819003698	3.73E-35	1.53E-33	Up	ATEG_04990	hypothetical protein	ko01100//Metabolic pathways;ko01110//Biosynthesis of secondary metabolites	GO:0016021//integral component of membrane	GO:0004497//monooxygenase activity;GO:0005506//iron ion binding;GO:0009055//electron carrier activity;GO:0016705//oxidoreductase activity, acting on paired donors, with incorporation or reduction of molecular oxygen;GO:0020037//heme binding	GO:0008202//steroid metabolic process;GO:0055114//oxidation-reduction process	gi|115397153|ref|XP_001214168.1|/0/conserved hypothetical protein [Aspergillus terreus NIH2624]
4321776	3986	229.12	527.78	1.810642439	5.18E-14	7.04E-13	Up	ATEG_05847	similar to spindle assembly checkpoint protein SLDA	ko04111//Cell cycle - yeast;ko04113//Meiosis - yeast	GO:0000778//condensed nuclear chromosome kinetochore;GO:0071957//old mitotic spindle pole body;GO:1990298//bub1-bub3 complex	GO:0005524//ATP binding;GO:0072371//histone kinase activity (H2A-S121 specific)	GO:0034501//protein localization to kinetochore;GO:0051315//attachment of mitotic spindle microtubules to kinetochore;GO:0051754//meiotic sister chromatid cohesion, centromeric;GO:0072370//histone H2A-S121 phosphorylation;GO:0072480//signal transduction involved in mitotic spindle assembly checkpoint	gi|115398872|ref|XP_001215025.1|/0/hypothetical protein ATEG_05847 [Aspergillus terreus NIH2624]
4316355	889	12.34	33.77	1.80601478	0.00829133	0.02644186	Up	ATEG_01834	hypothetical protein	-	-	-	-	gi|115385304|ref|XP_001209199.1|/1.47744e-115/predicted protein [Aspergillus terreus NIH2624]

4354830	1556	1070.33	2414.78	1.802671558	2.30E-34	9.06E-33	Up	ATEG_00074	similar to saccharopine reductase	ko01100//Metabolic pathways;ko01110//Biosynthesis of secondary metabolites;ko01130//Biosynthesis of antibiotics;ko01230//Biosynthesis of amino acids;ko00310//Lysine degradation;ko00330//Arginine and proline metabolism;ko00270//Cysteine and methionine metabolism;ko00410//beta-Alanine metabolism;ko00480//Glutathione metabolism;ko00300//Lysine biosynthesis	GO:0005737//cytoplasm	GO:0004755//saccharopine dehydrogenase (NADP+, L-glutamate-forming) activity	GO:0019878//lysine biosynthetic process via aminoadipic acid;GO:0055114//oxidation-reduction process	gi|115491065|ref|XP_001210160.1|/0/hypothetical protein ATEG_00074 [Aspergillus terreus NIH2624]
4318347	1575	206.25	468.51	1.799362856	6.12E-14	8.24E-13	Up	ATEG_03775	hypothetical protein	ko00380//Tryptophan metabolism;ko00071//Fatty acid degradation	GO:0016021//integral component of membrane	GO:0004497//monooxygenase activity;GO:0005506//iron ion binding;GO:0016705//oxidoreductase activity, acting on paired donors, with incorporation or reduction of molecular oxygen;GO:0020037//heme binding	GO:0055114//oxidation-reduction process	gi|115390897|ref|XP_001212953.1|/0/conserved hypothetical protein [Aspergillus terreus NIH2624]
4321131	1720	200.01	457.79	1.798100682	6.53E-14	8.77E-13	Up	ATEG_04980	hypothetical protein	-	GO:0016021//integral component of membrane	GO:0016491//oxidoreductase activity	GO:0055085//transmembrane transport;GO:0055114//oxidation-reduction process	gi|115397133|ref|XP_001214158.1|/0/conserved hypothetical protein [Aspergillus terreus NIH2624]

4319906	1347	15.13	39.58	1.796135489	0.00452138	0.01566042	Up	ATEG_04250	hypothetical protein	-	-	-	-	gi|115394834|ref|XP_001213428.1|/0/predicted protein [Aspergillus terreus NIH2624]
4321248	1536	202.88	462.43	1.794246755	7.11E-15	1.01E-13	Up	ATEG_05445	eukaryotic translation initiation factor 2 gamma subunit	ko03013//RNA transport	GO:0005850//eukaryotic translation initiation factor 2 complex;GO:0016282//eukaryotic 43S preinitiation complex;GO:0033290//eukaryotic 48S preinitiation complex;GO:0043614//multi-eIF complex	GO:0000049//tRNA binding;GO:0003743//translation initiation factor activity;GO:0003924//GTPase activity;GO:0005525//GTP binding;GO:0031369//translation initiation factor binding	GO:0001731//formation of translation preinitiation complex;GO:0045903//positive regulation of translational fidelity	gi|115398063|ref|XP_001214623.1|/0/eukaryotic translation initiation factor 2 gamma subunit [Aspergillus terreus NIH2624]
4316071	2118	307.52	697.79	1.790891574	1.57E-14	2.19E-13	Up	ATEG_01240	hypothetical protein	-	GO:0016021//integral component of membrane	-	-	gi|115384116|ref|XP_001208605.1|/0/conserved hypothetical protein [Aspergillus terreus NIH2624]
4355287	1541	264.49	594.84	1.78817934	2.57E-16	4.09E-15	Up	ATEG_00533	hypothetical protein	ko01100//Metabolic pathways;ko00051//Fructose and mannose metabolism;ko00650//Butanoate metabolism;ko00591//Linoleic acid metabolism	-	GO:0004616//phosphogluconate dehydrogenase (decarboxylating) activity;GO:0051287//NAD binding	GO:0055114//oxidation-reduction process	gi|115491983|ref|XP_001210619.1|/0/conserved hypothetical protein [Aspergillus terreus NIH2624]
4322950	1656	36.99	87.43	1.77920334	0.00012202	0.00060258	Up	ATEG_07597	hypothetical protein	ko03440//Homologous recombination;ko03450//Non-homologous end-joining	GO:0016021//integral component of membrane	-	GO:0055085//transmembrane transport	gi|115401260|ref|XP_001216218.1|/0/conserved hypothetical protein [Aspergillus terreus NIH2624]

4355276	753	56.57	132.21	1.77820855	1.48E-05	8.60E-05	Up	ATEG_00522	similar to short chain dehydrogenase	ko01100//Metabolic pathways;ko01212//Fatty acid metabolism;ko01040//Biosynthesis of unsaturated fatty acids;ko00061//Fatty acid biosynthesis;ko00780//Biotin metabolism	-	-	-	gi|115491961|ref|XP_001210608.1|/1.31011e-177/hypothetical protein ATEG_00522 [Aspergillus terreus NIH2624]
4320374	2084	282.41	626.66	1.770754604	1.37E-14	1.92E-13	Up	ATEG_04728	hypothetical protein	-	GO:0016021//integral component of membrane	-	-	gi|115396534|ref|XP_001213906.1|/0/conserved hypothetical protein [Aspergillus terreus NIH2624]
4353823	2085	58.81	135.57	1.766398298	1.26E-05	7.38E-05	Up	ATEG_09127	phenylalanine ammonia-lyase	ko01100//Metabolic pathways;ko01110//Biosynthesis of secondary metabolites;ko00360//Phenylalanine metabolism	GO:0005737//cytoplasm	GO:0045548//phenylalanine ammonia-lyase activity	GO:0006559//L-phenylalanine catabolic process;GO:0009800//cinnamic acid biosynthetic process	gi|115437192|ref|XP_001217749.1|/0/phenylalanine ammonia-lyase [Aspergillus terreus NIH2624]
4321391	2151	52.12	120.89	1.765034436	1.07E-05	6.35E-05	Up	ATEG_05721	hypothetical protein	ko01100//Metabolic pathways;ko00230//Purine metabolism;ko00220//Arginine biosynthesis	GO:0005634//nucleus	GO:0000981//RNA polymerase II transcription factor activity, sequence-specific DNA binding;GO:0003677//DNA binding;GO:0008270//zinc ion binding	GO:0006357//regulation of transcription from RNA polymerase II promoter	gi|115398620|ref|XP_001214899.1|/0/predicted protein [Aspergillus terreus NIH2624]
4322975	2457	196.63	436.9	1.763266826	9.31E-11	9.97E-10	Up	ATEG_07454	xylulose-5-phosphate phosphoketolase	ko01100//Metabolic pathways;ko00030//Pentose phosphate pathway	-	GO:0016832//aldehyde-lyase activity	GO:0005975//carbohydrate metabolic process	gi|115400974|ref|XP_001216075.1|/0/xylulose-5-phosphate phosphoketolase [Aspergillus terreus NIH2624]

4323302	720	2982.56	6578.1	1.762735434	8.81E-43	4.75E-41	Up	ATEG_08736	40S ribosomal protein S4	ko03010//Ribosome	GO:0005576//extracellular region;GO:0005840//ribosome	GO:0003735//structural constituent of ribosome;GO:0019843//rRNA binding	GO:0006412//translation	gi|115402491|ref|XP_001217322.1|/8.94354e-175/40S ribosomal protein S4 [Aspergillus terreus NIH2624]
4320732	1212	11.21	30.24	1.758448392	0.01287986	0.03851235	Up	ATEG_05191	hypothetical protein	-	GO:0005622//intracellular	GO:0005085//guanyl-nucleotide exchange factor activity	GO:0007264//small GTPase mediated signal transduction;GO:0043547//positive regulation of GTPase activity	gi|115397555|ref|XP_001214369.1|/0/predicted protein [Aspergillus terreus NIH2624]
4355438	2202	135.59	300.94	1.756086585	5.32E-09	4.79E-08	Up	ATEG_00683	hypothetical protein	ko00380//Tryptophan metabolism;ko00360//Phenylalanine metabolism;ko00330//Arginine and proline metabolism	-	GO:0016884//carbon-nitrogen ligase activity, with glutamine as amido-N-donor	-	gi|115492283|ref|XP_001210769.1|/0/predicted protein [Aspergillus terreus NIH2624]
4321679	2991	1198.38	2611.05	1.750994899	9.09E-30	3.05E-28	Up	ATEG_05890	similar to potential glycerophosphoinositol permease	-	GO:0016021//integral component of membrane	GO:0022857//transmembrane transporter activity	GO:0055085//transmembrane transport	gi|115398958|ref|XP_001215068.1|/0/hypothetical protein ATEG_05890 [Aspergillus terreus NIH2624]
4354226	3577	5300.55	11539.27	1.74882051	5.13E-52	3.62E-50	Up	ATEG_09654	hypothetical protein	ko04113//Meiosis - yeast	GO:0016021//integral component of membrane	GO:0022891//substrate-specific transmembrane transporter activity	GO:0055085//transmembrane transport	gi|115442938|ref|XP_001218276.1|/0/conserved hypothetical protein [Aspergillus terreus NIH2624]
4354225	2184	185.04	412.69	1.747488176	2.79E-09	2.59E-08	Up	ATEG_09653	hypothetical protein	ko01100//Metabolic pathways;ko00500//Starch and sucrose metabolism	GO:0005634//nucleus;GO:0016021//integral component of membrane	GO:0000981//RNA polymerase II transcription factor activity, sequence-specific DNA binding;GO:0003677//DNA binding;GO:0008270//zinc ion binding	GO:0006357//regulation of transcription from RNA polymerase II promoter	gi|115442936|ref|XP_001218275.1|/0/conserved hypothetical protein [Aspergillus terreus NIH2624]

4353786	960	166.48	366.39	1.743093639	8.02E-12	9.45E-11	Up	ATEG_09098	hypothetical protein	-	-	GO:0046872//metal ion binding;GO:0051213//dioxygenase activity	GO:0019748//secondary metabolic process;GO:0055114//oxidation-reduction process	gi|115437078|ref|XP_001217720.1|/0/conserved hypothetical protein [Aspergillus terreus NIH2624]
4319032	2248	14.57	36.79	1.742577636	0.00738215	0.02389331	Up	ATEG_07357	hypothetical protein	-	GO:0005634//nucleus;GO:0016020//membrane	GO:0003677//DNA binding;GO:0008270//zinc ion binding	GO:0006351//transcription, DNA-templated	gi|115387004|ref|XP_001210043.1|/0/predicted protein [Aspergillus terreus NIH2624]
4317867	1644	12.33	31.95	1.738557136	0.01099004	0.03361132	Up	ATEG_03378	hypothetical protein	ko01100//Metabolic pathways;ko00561//Glycerolipid metabolism	-	-	-	gi|115390102|ref|XP_001212556.1|/0/predicted protein [Aspergillus terreus NIH2624]
4322837	942	63.92	145.47	1.735796438	1.52E-05	8.77E-05	Up	ATEG_07683	hypothetical protein	-	GO:0000214//tRNA-intron endonuclease complex	GO:0000213//tRNA-intron endonuclease activity;GO:0003676//nucleic acid binding;GO:0016829//lyase activity	GO:0000379//tRNA-type intron splice site recognition and cleavage;GO:0090502//RNA phosphodiester bond hydrolysis, endonucleolytic	gi|115401432|ref|XP_001216304.1|/0/conserved hypothetical protein [Aspergillus terreus NIH2624]
4355607	648	3702.19	7986.53	1.733674968	8.03E-45	4.65E-43	Up	ATEG_00846	40S ribosomal protein S5	ko03010//Ribosome	GO:0005576//extracellular region;GO:0015935//small ribosomal subunit	GO:0003723//RNA binding;GO:0003735//structural constituent of ribosome	GO:0006412//translation	gi|115492609|ref|XP_001210932.1|/4.09622e-150/40S ribosomal protein S5 [Aspergillus terreus NIH2624]
4353780	723	24.14	62.21	1.726416633	0.01230183	0.03705557	Up	ATEG_09221	hypothetical protein	ko04144//Endocytosis	-	-	-	gi|115437562|ref|XP_001217843.1|/4.10109e-176/predicted protein [Aspergillus terreus NIH2624]

4316916	1086	32.5	74.69	1.721550764	0.00037228	0.00168585	Up	ATEG_02207	similar to 2-deoxy-d-gluconate 3-dehydrogenase	ko00040//Pentose and glucuronate interconversions	-	GO:0016491//oxidoreductase activity	GO:0055114//oxidation-reduction process	gi|115387759|ref|XP_001211385.1|/0/hypothetical protein ATEG_02207 [Aspergillus terreus NIH2624]
4321469	1755	15.69	38.82	1.711855789	0.00727859	0.023607	Up	ATEG_05860	hypothetical protein	ko01100//Metabolic pathways;ko00500//Starch and sucrose metabolism;ko00052//Galactose metabolism	-	GO:0004553//hydrolase activity, hydrolyzing O-glycosyl compounds	GO:0005975//carbohydrate metabolic process	gi|115398898|ref|XP_001215038.1|/0/predicted protein [Aspergillus terreus NIH2624]
4317797	1179	31.37	71.7	1.710144744	0.00079169	0.00331036	Up	ATEG_03463	hypothetical protein	ko01100//Metabolic pathways;ko00520//Amino sugar and nucleotide sugar metabolism	-	-	-	gi|115390272|ref|XP_001212641.1|/0/predicted protein [Aspergillus terreus NIH2624]
4318679	378	1316.26	2801.67	1.709840288	1.27E-32	4.68E-31	Up	ATEG_04158	60S ribosomal protein L22	ko03010//Ribosome	GO:0005730//nucleolus;GO:0005829//cytosol;GO:0005840//ribosome	GO:0003735//structural constituent of ribosome	GO:0006412//translation	gi|115391663|ref|XP_001213336.1|/1.33194e-84/60S ribosomal protein L22 [Aspergillus terreus NIH2624]
4322217	6303	87.96	191.55	1.70881568	2.78E-07	2.09E-06	Up	ATEG_06440	separin	ko04111//Cell cycle - yeast;ko04113//Meiosis - yeast	GO:0005634//nucleus	GO:0008233//peptidase activity	GO:0006508//proteolysis	gi|115400059|ref|XP_001215618.1|/0/separin [Aspergillus terreus NIH2624]
4354041	1098	21.87	51.67	1.70537102	0.00546098	0.01841193	Up	ATEG_09053	hypothetical protein	ko00640//Propanoate metabolism	GO:0005622//intracellular	GO:0008270//zinc ion binding;GO:0016491//oxidoreductase activity	GO:0055114//oxidation-reduction process	gi|115436906|ref|XP_001217675.1|/0/conserved hypothetical protein [Aspergillus terreus NIH2624]
4316059	3112	3591.11	7626.77	1.705158143	7.13E-36	3.06E-34	Up	ATEG_01280	cytochrome c	ko01100//Metabolic pathways;ko00920//Sulfur metabolism	GO:0005739//mitochondrion;GO:0070469//respiratory chain	GO:0009055//electron carrier activity;GO:0020037//heme binding;GO:0046872//metal ion binding	GO:0055114//oxidation-reduction process	gi|115384196|ref|XP_001208645.1|/2.52774e-94/cytochrome c [Aspergillus terreus NIH2624]

4355125	3392	588.34	1249.74	1.705124845	6.69E-24	1.67E-22	Up	ATEG_00373	similar to possible bhlh transcription factor	ko01100//Metabolic pathways;ko00250//Alanine, aspartate and glutamate metabolism;ko00330//Arginine and proline metabolism	-	GO:0046983//protein dimerization activity	-	gi|115491663|ref|XP_001210459.1|/0/hypothetical protein ATEG_00373 [Aspergillus terreus NIH2624]
4317203	1690	14.02	34.8	1.704453013	0.01293942	0.03864116	Up	ATEG_02710	similar to luciferase	ko01100//Metabolic pathways;ko01110//Biosynthesis of secondary metabolites;ko00360//Phenylalanine metabolism;ko00130//Ubiquinone and other terpenoid-quinone biosynthesis	GO:0016021//integral component of membrane	GO:0003824//catalytic activity	GO:0008152//metabolic process	gi|115388765|ref|XP_001211888.1|/0/hypothetical protein ATEG_02710 [Aspergillus terreus NIH2624]
4318557	464	65	143.02	1.701819616	4.77E-06	2.99E-05	Up	ATEG_04195	hypothetical protein	-	-	-	-	gi|115391737|ref|XP_001213373.1|/4.33858e-84/conserved hypothetical protein [Aspergillus terreus NIH2624]
4355501	1899	40.36	90.65	1.700108148	0.00016897	0.00081598	Up	ATEG_00746	hypothetical protein	-	-	GO:0004672//protein kinase activity;GO:0005524//ATP binding	GO:0006468//protein phosphorylation	gi|115492409|ref|XP_001210832.1|/0/conserved hypothetical protein [Aspergillus terreus NIH2624]
4354271	1974	891.73	1874.98	1.697603977	2.00E-28	6.29E-27	Up	ATEG_09669	similar to NADP-dependent malic enzyme	ko01100//Metabolic pathways;ko01200//Carbon metabolism;ko00620//Pyruvate metabolism	-	GO:0004471//malate dehydrogenase (decarboxylating) (NAD+) activity;GO:0046872//metal ion binding;GO:0051287//NAD binding	GO:0006108//malate metabolic process;GO:0055114//oxidation-reduction process	gi|115442968|ref|XP_001218291.1|/0/hypothetical protein ATEG_09669 [Aspergillus terreus NIH2624]

4321221	684	18.49	45.43	1.691593929	0.00748948	0.02415738	Up	ATEG_05276	hypothetical protein	ko04144//Endocytosis	-	-	-	gi|115397725|ref|XP_001214454.1|/4.73821e-155/predicted protein [Aspergillus terreus NIH2624]
4319219	2384	70.6	152.48	1.688150498	2.60E-06	1.71E-05	Up	ATEG_07015	hypothetical protein	ko01100//Metabolic pathways;ko00500//Starch and sucrose metabolism	-	-	-	gi|115386320|ref|XP_001209701.1|/0/conserved hypothetical protein [Aspergillus terreus NIH2624]
4322849	999	105.32	224.36	1.686413024	1.68E-07	1.29E-06	Up	ATEG_07904	hypothetical protein	ko01100//Metabolic pathways;ko00500//Starch and sucrose metabolism	GO:0016021//integral component of membrane	-	-	gi|115401874|ref|XP_001216525.1|/0/conserved hypothetical protein [Aspergillus terreus NIH2624]
4316081	4643	72.83	156.64	1.685952966	1.28E-05	7.51E-05	Up	ATEG_01291	hypothetical protein	ko03040//Spliceosome	-	-	-	gi|115384218|ref|XP_001208656.1|/0/predicted protein [Aspergillus terreus NIH2624]
4319603	2076	139.58	295.86	1.682091843	2.69E-09	2.51E-08	Up	ATEG_10040	similar to Noc3p	-	GO:0005656//nuclear pre-replicative complex;GO:0005730//nucleolus;GO:0030691//Noc2p-Noc3p complex	GO:0003682//chromatin binding	GO:0006267//pre-replicative complex assembly involved in nuclear cell cycle DNA replication;GO:0006270//DNA replication initiation;GO:0006364//rRNA processing	gi|115385591|ref|XP_001209342.1|/0/hypothetical protein ATEG_10040 [Aspergillus terreus NIH2624]
4323487	1907	2695.52	5541.78	1.673891919	6.09E-27	1.82E-25	Up	ATEG_08717	hypothetical protein	-	-	GO:0004181//metallocarboxypeptidase activity	GO:0006508//proteolysis	gi|115402453|ref|XP_001217303.1|/0/conserved hypothetical protein [Aspergillus terreus NIH2624]

4355235	1230	5545.89	11448.82	1.672638271	4.22E-47	2.66E-45	Up	ATEG_00481	60S ribosomal protein L5	ko03010//Ribosome	GO:0022625//cytosolic large ribosomal subunit	GO:0003735//structural constituent of ribosome;GO:0008097//5S rRNA binding	GO:0000027//ribosomal large subunit assembly;GO:0006412//translation	gi|115491879|ref|XP_001210567.1|/0/60S ribosomal protein L5 [Aspergillus terreus NIH2624]
4317697	1608	271.77	565.82	1.672530315	1.20E-15	1.80E-14	Up	ATEG_02854	hypothetical protein	ko00790//Folate biosynthesis	GO:0016021//integral component of membrane	-	GO:0055085//transmembrane transport	gi|115389054|ref|XP_001212032.1|/0/conserved hypothetical protein [Aspergillus terreus NIH2624]
4353713	582	59.98	129.26	1.67247874	1.71E-05	9.81E-05	Up	ATEG_09250	hypothetical protein	ko04141//Protein processing in endoplasmic reticulum	-	-	-	gi|115437680|ref|XP_001217872.1|/4.52108e-143/predicted protein [Aspergillus terreus NIH2624]
4318975	1351	267.28	553.55	1.671329669	1.72E-09	1.62E-08	Up	ATEG_07059	hypothetical protein	-	-	GO:0016846//carbon-sulfur lyase activity	GO:0008152//metabolic process	gi|115386408|ref|XP_001209745.1|/1.11166e-106/predicted protein [Aspergillus terreus NIH2624]
4322822	1299	2987.82	6100.23	1.664943263	6.12E-22	1.39E-20	Up	ATEG_07523	hypothetical protein	ko01100//Metabolic pathways;ko01110//Biosynthesis of secondary metabolites	-	GO:0005506//iron ion binding;GO:0016708//oxidoreductase activity, acting on paired donors, with incorporation or reduction of molecular oxygen, NAD(P)H as one donor, and incorporation of two atoms of oxygen into one donor;GO:0051537//2 iron, 2 sulfur cluster binding	GO:0019439//aromatic compound catabolic process;GO:0055114//oxidation-reduction process	gi|115401112|ref|XP_001216144.1|/0/conserved hypothetical protein [Aspergillus terreus NIH2624]

4355383	2173	491.03	1010.08	1.664149243	4.03E-18	7.09E-17	Up	ATEG_00629	hypothetical protein	ko01100//Metabolic pathways;ko01220//Degradation of aromatic compounds	-	GO:0008199//ferric iron binding;GO:0018576//catechol 1,2-dioxygenase activity	GO:0009712//catechol-containing compound metabolic process;GO:0055114//oxidation-reduction process	gi|115492175|ref|XP_001210715.1|/0/conserved hypothetical protein [Aspergillus terreus NIH2624]
4353752	1019	508.2	1045.73	1.659251896	1.05E-20	2.23E-19	Up	ATEG_09461	hypothetical protein	ko04141//Protein processing in endoplasmic reticulum	GO:0005789//endoplasmic reticulum membrane;GO:0016021//integral component of membrane	-	-	gi|115438504|ref|XP_001218083.1|/0/conserved hypothetical protein [Aspergillus terreus NIH2624]
4318230	712	6005.72	12268.63	1.655248842	2.00E-42	1.07E-40	Up	ATEG_03764	60S ribosomal protein L15	ko03010//Ribosome	GO:0005840//ribosome	GO:0003735//structural constituent of ribosome	GO:0006412//translation	gi|115390875|ref|XP_001212942.1|/2.40758e-152/60S ribosomal protein L15 [Aspergillus terreus NIH2624]
4319160	3479	2477.99	5026.18	1.653448644	1.62E-24	4.22E-23	Up	ATEG_07165	hypothetical protein	-	GO:0005634//nucleus	GO:0000981//RNA polymerase II transcription factor activity, sequence-specific DNA binding;GO:0008270//zinc ion binding	GO:0006357//regulation of transcription from RNA polymerase II promoter	gi|115386620|ref|XP_001209851.1|/0/conserved hypothetical protein [Aspergillus terreus NIH2624]
4320928	834	7636.58	15536.88	1.651273773	7.46E-47	4.64E-45	Up	ATEG_05463	60S ribosomal protein L36	ko03010//Ribosome	GO:0005840//ribosome	GO:0003735//structural constituent of ribosome	GO:0006412//translation	gi|115398099|ref|XP_001214641.1|/1.67785e-64/60S ribosomal protein L36 [Aspergillus terreus NIH2624]
4355114	2415	142.88	294.7	1.647601444	1.15E-09	1.10E-08	Up	ATEG_00362	hypothetical protein	ko01100//Metabolic pathways;ko00780//Biotin metabolism	GO:0016021//integral component of membrane	-	-	gi|115491641|ref|XP_001210448.1|/0/conserved hypothetical protein [Aspergillus terreus NIH2624]

4319954	1869	17203.36	34843.85	1.645949356	1.51E-50	1.01E-48	Up	ATEG_04443	40S ribosomal protein S15	ko03010//Ribosome	GO:0015935//small ribosomal subunit	GO:0003723//RNA binding;GO:0003735//structural constituent of ribosome	GO:0006412//translation	gi|115395964|ref|XP_001213621.1|/7.1084e-107/40S ribosomal protein S15 [Aspergillus terreus NIH2624]
4322065	972	41.48	89.13	1.643888974	0.00029223	0.00135541	Up	ATEG_06622	hypothetical protein	-	-	GO:0016491//oxidoreductase activity	GO:0055114//oxidation-reduction process	gi|115400423|ref|XP_001215800.1|/0/conserved hypothetical protein [Aspergillus terreus NIH2624]
4322003	801	65.02	138.97	1.642718932	4.34E-05	0.00023459	Up	ATEG_06682	hypothetical protein	-	-	GO:0010181//FMN binding;GO:0016491//oxidoreductase activity	GO:0055114//oxidation-reduction process	gi|115400543|ref|XP_001215860.1|/0/conserved hypothetical protein [Aspergillus terreus NIH2624]
4354296	2905	687.74	1388.09	1.642085903	9.04E-19	1.67E-17	Up	ATEG_09811	similar to heat shock transcription factor 2	-	GO:0005634//nucleus	GO:0003700//transcription factor activity, sequence-specific DNA binding;GO:0043565//sequence-specific DNA binding	GO:0006355//regulation of transcription, DNA-templated	gi|115443252|ref|XP_001218433.1|/0/hypothetical protein ATEG_09811 [Aspergillus terreus NIH2624]
4316158	1494	1251.4	2504.05	1.633846075	7.27E-21	1.56E-19	Up	ATEG_01802	hypothetical protein	ko01100//Metabolic pathways;ko00230//Purine metabolism;ko00250//Alanine, aspartate and glutamate metabolism	GO:0016021//integral component of membrane	-	GO:0055085//transmembrane transport	gi|115385240|ref|XP_001209167.1|/0/conserved hypothetical protein [Aspergillus terreus NIH2624]
4353715	3127	833.86	1684.64	1.633753269	5.84E-26	1.66E-24	Up	ATEG_09252	hypothetical protein	ko03013//RNA transport	GO:0016021//integral component of membrane	GO:0015171//amino acid transmembrane transporter activity	GO:0003333//amino acid transmembrane transport	gi|115437688|ref|XP_001217874.1|/0/conserved hypothetical protein [Aspergillus terreus NIH2624]

4322877	3067	94.71	194.14	1.632682289	8.65E-07	6.00E-06	Up	ATEG_07850	hypothetical protein	-	GO:0005634//nucleus	GO:0000981//RNA polymerase II transcription factor activity, sequence-specific DNA binding;GO:0008270//zinc ion binding	GO:0006357//regulation of transcription from RNA polymerase II promoter	gi|115401766|ref|XP_001216471.1|/0/predicted protein [Aspergillus terreus NIH2624]
4354315	2099	458.95	929.26	1.632594553	3.53E-19	6.77E-18	Up	ATEG_09541	hypothetical protein	-	-	-	-	gi|115442712|ref|XP_001218163.1|/0/predicted protein [Aspergillus terreus NIH2624]
4315800	1395	848.99	1722.31	1.631160833	2.09E-20	4.35E-19	Up	ATEG_01692	argininosuccinate lyase	ko01100//Metabolic pathways;ko01110//Biosynthesis of secondary metabolites;ko01130//Biosynthesis of antibiotics;ko01230//Biosynthesis of amino acids;ko00250//Alanine, aspartate and glutamate metabolism;ko00220//Arginine biosynthesis	GO:0005829//cytosol	GO:0004056//argininosuccinate lyase activity	GO:0042450//arginine biosynthetic process via ornithine	gi|115385020|ref|XP_001209057.1|/0/argininosuccinate lyase [Aspergillus terreus NIH2624]
4317945	3912	36.41	78.43	1.630033943	0.00069233	0.0029328	Up	ATEG_03553	hypothetical protein	-	-	-	-	gi|115390452|ref|XP_001212731.1|/0/predicted protein [Aspergillus terreus NIH2624]

4354417	1851	28.57	61.9	1.624341905	0.00224171	0.00843719	Up	ATEG_09884	delta-1-pyrroline-5-carboxylate dehydrogenase, mitochondrial precursor	ko01100//Metabolic pathways;ko00250//Alanine, aspartate and glutamate metabolism;ko00330//Arginine and proline metabolism	-	GO:0003842//1-pyrroline-5-carboxylate dehydrogenase activity;GO:0016620//oxidoreductase activity, acting on the aldehyde or oxo group of donors, NAD or NADP as acceptor	GO:0006537//glutamate biosynthetic process;GO:0006561//proline biosynthetic process;GO:0055114//oxidation-reduction process	gi|115443398|ref|XP_001218506.1|/0/delta-1-pyrroline-5-carboxylate dehydrogenase, mitochondrial precursor [Aspergillus terreus NIH2624]
4354780	1647	5966.48	11824.16	1.622534432	2.10E-24	5.42E-23	Up	ATEG_00024	similar to carboxypeptidase S1	-	-	GO:0004185//serine-type carboxypeptidase activity	GO:0006508//proteolysis	gi|115490965|ref|XP_001210110.1|/0/hypothetical protein ATEG_00024 [Aspergillus terreus NIH2624]
4322309	1830	89.71	186.21	1.618075688	1.24E-05	7.27E-05	Up	ATEG_06720	similar to dehydrogenase	ko01100//Metabolic pathways;ko00051//Fructose and mannose metabolism;ko00650//Butanoate metabolism;ko00591//Linoleic acid metabolism	-	GO:0003857//3-hydroxyacyl-CoA dehydrogenase activity	GO:0006631//fatty acid metabolic process;GO:0055114//oxidation-reduction process	gi|115400619|ref|XP_001215898.1|/0/hypothetical protein ATEG_06720 [Aspergillus terreus NIH2624]
4321617	465	1600.13	3187.49	1.615471253	1.24E-26	3.63E-25	Up	ATEG_05654	hypothetical protein	ko03010//Ribosome	GO:0005829//cytosol;GO:0005840//ribosome	GO:0003735//structural constituent of ribosome	GO:0006412//translation	gi|115398486|ref|XP_001214832.1|/2.19782e-106/conserved hypothetical protein [Aspergillus terreus NIH2624]

4322649	3678	139.48	281.83	1.614100893	5.45E-08	4.44E-07	Up	ATEG_07762	similar to protein kinase	-	GO:0000922//spindle pole;GO:0005634//nucleus;GO:0005935//cellular bud neck	GO:0004672//protein kinase activity;GO:0005524//ATP binding;GO:0008047//enzyme activator activity	GO:0000712//resolution of meiotic recombination intermediates;GO:0006468//protein phosphorylation;GO:0008104//protein localization;GO:0010696//positive regulation of spindle pole body separation;GO:0043085//positive regulation of catalytic activity;GO:0070194//synaptonemal complex disassembly;GO:0090306//spindle assembly involved in meiosis	gi|115401590|ref|XP_001216383.1|/0/hypothetical protein ATEG_07762 [Aspergillus terreus NIH2624]
4316622	1827	2096.11	4134.42	1.611212227	3.19E-26	9.23E-25	Up	ATEG_02023	similar to adenylate-forming enzyme	ko01100//Metabolic pathways;ko01110//Biosynthesis of secondary metabolites;ko00360//Phenylalanine metabolism;ko00130//Ubiquinone and other terpenoid-quinone biosynthesis	-	GO:0003824//catalytic activity	GO:0008152//metabolic process	gi|115387391|ref|XP_001211201.1|/0/hypothetical protein ATEG_02023 [Aspergillus terreus NIH2624]
4317107	1299	54.9	119.31	1.610295644	0.00103276	0.00421322	Up	ATEG_02182	similar to oxidoreductase	ko01100//Metabolic pathways;ko01220//Degradation of aromatic compounds	-	GO:0016491//oxidoreductase activity;GO:0071949//FAD binding	GO:0055114//oxidation-reduction process	gi|115387709|ref|XP_001211360.1|/0/hypothetical protein ATEG_02182 [Aspergillus terreus NIH2624]

4316548	2322	193.93	388.87	1.609826644	5.48E-11	5.95E-10	Up	ATEG_02320	hypothetical protein	-	-	GO:0008017//microtubule binding	GO:0000226//microtubule cytoskeleton organization;GO:0000910//cytokinesis	gi|115387985|ref|XP_001211498.1|/0/conserved hypothetical protein [Aspergillus terreus NIH2624]
4320156	801	29.12	62.61	1.609712158	0.00353216	0.01259189	Up	ATEG_04913	hypothetical protein	ko01100//Metabolic pathways;ko00520//Amino sugar and nucleotide sugar metabolism	-	-	-	gi|115396904|ref|XP_001214091.1|/0/predicted protein [Aspergillus terreus NIH2624]
4317434	835	7424.68	14673.96	1.60916103	1.87E-43	1.03E-41	Up	ATEG_02940	40S ribosomal protein S26	ko03010//Ribosome	GO:0005840//ribosome	GO:0003735//structural constituent of ribosome	GO:0006412//translation	gi|115389226|ref|XP_001212118.1|/2.44281e-84/40S ribosomal protein S26 [Aspergillus terreus NIH2624]
4354372	4383	432.48	858.18	1.608104646	2.18E-14	3.02E-13	Up	ATEG_09685	hypothetical protein	-	GO:0005829//cytosol;GO:0005940//septin ring;GO:0031097//medial cortex	-	GO:0000281//mitotic cytokinesis;GO:0031107//septin ring disassembly	gi|115443000|ref|XP_001218307.1|/0/conserved hypothetical protein [Aspergillus terreus NIH2624]
4319112	1125	48.2	99.8	1.607767979	0.00016002	0.00077437	Up	ATEG_07315	similar to arginase family protein	ko01100//Metabolic pathways;ko00330//Arginine and proline metabolism	-	GO:0016813//hydrolase activity, acting on carbon-nitrogen (but not peptide) bonds, in linear amidines;GO:0046872//metal ion binding	-	gi|115386920|ref|XP_001210001.1|/0/hypothetical protein ATEG_07315 [Aspergillus terreus NIH2624]
4323304	5671	7258.1	14297	1.606336563	6.70E-46	3.95E-44	Up	ATEG_08738	hypothetical protein	ko00620//Pyruvate metabolism	-	GO:0016614//oxidoreductase activity, acting on CH-OH group of donors;GO:0050660//flavin adenine dinucleotide binding	GO:0055114//oxidation-reduction process	gi|115402495|ref|XP_001217324.1|/0/conserved hypothetical protein [Aspergillus terreus NIH2624]

4354992	375	1337.43	2648.22	1.605601653	2.72E-26	7.89E-25	Up	ATEG_00243	60S ribosomal protein L35	ko03010//Ribosome	GO:0005840//ribosome	GO:0003735//structural constituent of ribosome	GO:0006412//translation	gi|115491403|ref|XP_001210329.1|/4.84234e-82/60S ribosomal protein L35 [Aspergillus terreus NIH2624]
4323162	1374	60.5	123.66	1.604251114	8.55E-05	0.00043455	Up	ATEG_08898	hypothetical protein	ko01100//Metabolic pathways;ko01220//Degradation of aromatic compounds	-	GO:0016491//oxidoreductase activity;GO:0071949//FAD binding	GO:0055114//oxidation-reduction process	gi|115402815|ref|XP_001217484.1|/0/predicted protein [Aspergillus terreus NIH2624]
4318125	753	16.26	36.76	1.603568486	0.01401183	0.04142186	Up	ATEG_03050	hypothetical protein	ko01100//Metabolic pathways;ko01110//Biosynthesis of secondary metabolites;ko00860//Porphyrin and chlorophyll metabolism	GO:0016021//integral component of membrane	-	-	gi|115389446|ref|XP_001212228.1|/0/conserved hypothetical protein [Aspergillus terreus NIH2624]
4320858	1845	138.94	278.92	1.601410948	2.05E-08	1.73E-07	Up	ATEG_05518	hypothetical protein	ko00254//Aflatoxin biosynthesis	-	-	-	gi|115398209|ref|XP_001214696.1|/0/conserved hypothetical protein [Aspergillus terreus NIH2624]
4353814	1830	171.4	342.67	1.600729508	1.84E-08	1.57E-07	Up	ATEG_09368	hypothetical protein	-	GO:0016021//integral component of membrane	GO:0003676//nucleic acid binding;GO:0005524//ATP binding;GO:0008270//zinc ion binding;GO:0016818//hydrolase activity, acting on acid anhydrides, in phosphorus-containing anhydrides	GO:0055085//transmembrane transport	gi|115438142|ref|XP_001217990.1|/0/conserved hypothetical protein [Aspergillus terreus NIH2624]

4317815	504	3633.65	7148.84	1.599889033	2.72E-38	1.28E-36	Up	ATEG_03361	60S ribosomal protein L31	ko03010//Ribosome	GO:0005840//ribosome	GO:0003735//structural constituent of ribosome	GO:0006412//translation	gi|115390068|ref|XP_001212539.1|/1.70458e-43/60S ribosomal protein L31 [Aspergillus terreus NIH2624]
4316434	1398	478.49	941.19	1.598776851	1.23E-17	2.11E-16	Up	ATEG_02025	hypothetical protein	-	GO:0016021//integral component of membrane	-	-	gi|115387395|ref|XP_001211203.1|/0/conserved hypothetical protein [Aspergillus terreus NIH2624]
4320290	1467	61.66	126.27	1.598465031	3.83E-05	0.00020894	Up	ATEG_04688	hypothetical protein	ko01100//Metabolic pathways;ko00561//Glycerolipid metabolism	-	GO:0016787//hydrolase activity	GO:0008152//metabolic process	gi|115396454|ref|XP_001213866.1|/0/conserved hypothetical protein [Aspergillus terreus NIH2624]
4320943	694	4334.17	8491.9	1.597750156	2.21E-43	1.21E-41	Up	ATEG_05430	60S ribosomal protein L11	ko03010//Ribosome	GO:0005840//ribosome	GO:0003735//structural constituent of ribosome	GO:0006412//translation	gi|115398033|ref|XP_001214608.1|/4.26637e-126/60S ribosomal protein L11 [Aspergillus terreus NIH2624]
4354595	999	175.94	356.45	1.597734745	7.45E-08	5.98E-07	Up	ATEG_10354	hypothetical protein	-	-	GO:0016491//oxidoreductase activity	GO:0055114//oxidation-reduction process	gi|115449813|ref|XP_001218702.1|/0/conserved hypothetical protein [Aspergillus terreus NIH2624]
4321273	2775	289.73	578.23	1.595299553	1.58E-11	1.81E-10	Up	ATEG_05983	hypothetical protein	-	-	-	-	gi|115399144|ref|XP_001215161.1|/0/conserved hypothetical protein [Aspergillus terreus NIH2624]

4320653	465	2451.68	4806.91	1.594975368	2.32E-27	7.10E-26	Up	ATEG_05268	ATP synthase protein 9, mitochondrial precursor	ko01100//Metabolic pathways;ko00190//Oxidative phosphorylation	GO:0016021//integral component of membrane;GO:0045263//proton-transporting ATP synthase complex, coupling factor F(o)	GO:0015078//hydrogen ion transmembrane transporter activity;GO:0016887//ATPase activity	GO:0015986//ATP synthesis coupled proton transport;GO:0015991//ATP hydrolysis coupled proton transport	gi|115397709|ref|XP_001214446.1|/1.17378e-101/ATP synthase protein 9, mitochondrial precursor [Aspergillus terreus NIH2624]
4353190	1689	556.99	1088.95	1.593381627	1.49E-19	2.98E-18	Up	ATEG_08180	hypothetical protein	ko01100//Metabolic pathways;ko00500//Starch and sucrose metabolism	GO:0016021//integral component of membrane	-	GO:0038032//termination of G-protein coupled receptor signaling pathway	gi|115433328|ref|XP_001216801.1|/0/conserved hypothetical protein [Aspergillus terreus NIH2624]
4319362	1587	1185.46	2319.5	1.591671915	4.27E-22	9.84E-21	Up	ATEG_07126	malate synthase	ko01100//Metabolic pathways;ko01110//Biosynthesis of secondary metabolites;ko01200//Carbon metabolism;ko00620//Pyruvate metabolism;ko00630//Glyoxylate and dicarboxylate metabolism	-	GO:0004474//malate synthase activity	GO:0006097//glyoxylate cycle;GO:0006099//tricarboxylic acid cycle	gi|115386542|ref|XP_001209812.1|/0/malate synthase [Aspergillus terreus NIH2624]
4321188	2748	42.01	88.14	1.590815966	0.00074778	0.00314357	Up	ATEG_04952	hypothetical protein	-	-	-	-	gi|115397077|ref|XP_001214130.1|/0/predicted protein [Aspergillus terreus NIH2624]
4319236	1167	1219	2379.9	1.589663597	3.38E-19	6.52E-18	Up	ATEG_07101	biotin synthase	ko01100//Metabolic pathways;ko00780//Biotin metabolism	-	GO:0004076//biotin synthase activity;GO:0046872//metal ion binding;GO:0051537//2 iron, 2 sulfur cluster binding;GO:0051539//4 iron, 4 sulfur cluster binding	GO:0009102//biotin biosynthetic process	gi|115386492|ref|XP_001209787.1|/0/biotin synthase [Aspergillus terreus NIH2624]

4315740	1338	2095.27	4075.57	1.589555078	3.79E-35	1.55E-33	Up	ATEG_01397	similar to NADH-cytochrome b5 reductase	ko00520//Amino sugar and nucleotide sugar metabolism	GO:0005741//mitochondrial outer membrane;GO:0005789//endoplasmic reticulum membrane;GO:0016021//integral component of membrane	GO:0004128//cytochrome-b5 reductase activity, acting on NAD(P)H	GO:0055114//oxidation-reduction process	gi|115384430|ref|XP_001208762.1|/0/hypothetical protein ATEG_01397 [Aspergillus terreus NIH2624]
4318845	935	7797.1	15179.77	1.588563306	2.07E-41	1.07E-39	Up	ATEG_07208	60S ribosomal protein L24	ko03010//Ribosome	GO:0005840//ribosome	-	-	gi|115386706|ref|XP_001209894.1|/4.71382e-109/60S ribosomal protein L24 [Aspergillus terreus NIH2624]
4316267	2501	40.32	84.72	1.588337785	0.00138844	0.00549229	Up	ATEG_01778	hypothetical protein	ko04120//Ubiquitin mediated proteolysis	-	-	GO:0006355//regulation of transcription, DNA-templated	gi|115385192|ref|XP_001209143.1|/0/conserved hypothetical protein [Aspergillus terreus NIH2624]
4354650	2125	25.79	56.1	1.587968556	0.00534156	0.01808075	Up	ATEG_10245	hypothetical protein	ko01100//Metabolic pathways;ko00500//Starch and sucrose metabolism	GO:0005634//nucleus	GO:0000981//RNA polymerase II transcription factor activity, sequence-specific DNA binding;GO:0003677//DNA binding;GO:0004553//hydrolase activity, hydrolyzing O-glycosyl compounds;GO:0008270//zinc ion binding	GO:0006357//regulation of transcription from RNA polymerase II promoter;GO:2000999	gi|115449385|ref|XP_001218593.1|/0/conserved hypothetical protein [Aspergillus terreus NIH2624]
4354026	1721	710.16	1379.67	1.58750183	6.45E-18	1.12E-16	Up	ATEG_09060	hypothetical protein	-	GO:0005886//plasma membrane;GO:0016021//integral component of membrane	GO:0015205//nucleobase transmembrane transporter activity	GO:0015851//nucleobase transport;GO:0055085//transmembrane transport	gi|115436934|ref|XP_001217682.1|/0/conserved hypothetical protein [Aspergillus terreus NIH2624]

4355241	2025	20.76	45.83	1.586226297	0.01184144	0.03585292	Up	ATEG_00487	hypothetical protein	ko01100//Metabolic pathways;ko00260//Glycine, serine and threonine metabolism	-	GO:0016614//oxidoreductase activity, acting on CH-OH group of donors;GO:0050660//flavin adenine dinucleotide binding	GO:0055114//oxidation-reduction process	gi|115491891|ref|XP_001210573.1|/0/conserved hypothetical protein [Aspergillus terreus NIH2624]
4353255	2208	171.42	338.44	1.58492174	3.52E-09	3.24E-08	Up	ATEG_08005	lanosterol synthase	ko01100//Metabolic pathways;ko01110//Biosynthesis of secondary metabolites;ko01130//Biosynthesis of antibiotics;ko00100//Steroid biosynthesis	-	GO:0016866//intramolecular transferase activity	-	gi|115432978|ref|XP_001216626.1|/0/lanosterol synthase [Aspergillus terreus NIH2624]
4318939	2501	86.28	170.82	1.573318379	4.28E-06	2.70E-05	Up	ATEG_07323	hypothetical protein	-	GO:0005634//nucleus	GO:0000981//RNA polymerase II transcription factor activity, sequence-specific DNA binding;GO:0008270//zinc ion binding	GO:0006357//regulation of transcription from RNA polymerase II promoter	gi|115386936|ref|XP_001210009.1|/0/conserved hypothetical protein [Aspergillus terreus NIH2624]
4321639	1827	158.64	308.85	1.570822122	2.28E-08	1.92E-07	Up	ATEG_05961	hypothetical protein	-	GO:0016021//integral component of membrane	-	GO:0055085//transmembrane transport	gi|115399100|ref|XP_001215139.1|/0/conserved hypothetical protein [Aspergillus terreus NIH2624]

4316940	3195	7231.17	13851.22	1.566398365	3.80E-33	1.44E-31	Up	ATEG_02190	glycine dehydrogenase, mitochondrial precursor	ko01100//Metabolic pathways;ko01110//Biosynthesis of secondary metabolites;ko01130//Biosynthesis of antibiotics;ko01200//Carbon metabolism;ko00260//Glycine, serine and threonine metabolism;ko00630//Glyoxylate and dicarboxylate metabolism	GO:0016021//integral component of membrane	GO:0004375//glycine dehydrogenase (decarboxylating) activity;GO:0016787//hydrolase activity	GO:0006546//glycine catabolic process;GO:0055114//oxidation-reduction process	gi|115387725|ref|XP_001211368.1|/0/glycine dehydrogenase, mitochondrial precursor [Aspergillus terreus NIH2624]
4315560	432	2382.53	4557.76	1.561752654	2.84E-33	1.08E-31	Up	ATEG_01522	40S ribosomal protein S16	ko03010//Ribosome	GO:0005840//ribosome	GO:0003735//structural constituent of ribosome	GO:0006412//translation	gi|115384680|ref|XP_001208887.1|/1.65361e-98/40S ribosomal protein S16 [Aspergillus terreus NIH2624]
4317401	1323	40.37	83.81	1.559807895	0.00183097	0.00704387	Up	ATEG_02785	hypothetical protein	-	-	-	-	gi|115388916|ref|XP_001211963.1|/0/predicted protein [Aspergillus terreus NIH2624]
4316389	426	97	194.92	1.557941374	6.88E-05	0.00035757	Up	ATEG_01242	allergen Asp f 15 precursor	ko04144//Endocytosis	GO:0005576//extracellular region;GO:0005622//intracellular	-	-	gi|115384120|ref|XP_001208607.1|/4.23699e-100/allergen Asp f 15 precursor [Aspergillus terreus NIH2624]
4354352	1623	836.56	1603.19	1.554305448	1.56E-20	3.29E-19	Up	ATEG_09694	glutamine amidotransferase subunit pdxT	ko00750//Vitamin B6 metabolism	-	GO:0004359//glutaminase activity;GO:0016740//transferase activity	GO:0006541//glutamine metabolic process;GO:0042819//vitamin B6 biosynthetic process;GO:0042823//pyridoxal phosphate biosynthetic process	gi|115443018|ref|XP_001218316.1|/0/glutamine amidotransferase subunit pdxT [Aspergillus terreus NIH2624]

4353651	2207	165.88	319.36	1.554136812	1.42E-09	1.35E-08	Up	ATEG_09030	hypothetical protein	-	GO:0005634//nucleus	GO:0003677//DNA binding;GO:0008270//zinc ion binding	GO:0006351//transcription, DNA-templated	gi|115436820|ref|XP_001217652.1|/0/predicted protein [Aspergillus terreus NIH2624]
4318617	519	2826.21	5370.32	1.551806589	2.30E-36	1.01E-34	Up	ATEG_03745	60S ribosomal protein L20	ko03010//Ribosome	GO:0005840//ribosome	GO:0003735//structural constituent of ribosome	GO:0006412//translation	gi|115390837|ref|XP_001212923.1|/5.85592e-122/60S ribosomal protein L20 [Aspergillus terreus NIH2624]
4317648	1491	129.96	252.01	1.551214325	3.28E-07	2.45E-06	Up	ATEG_02875	hypothetical protein	-	-	-	-	gi|115389096|ref|XP_001212053.1|/0/predicted protein [Aspergillus terreus NIH2624]
4355717	708	74.52	145.15	1.549718524	3.62E-05	0.00019851	Up	ATEG_00954	hypothetical protein	-	GO:0005634//nucleus;GO:0005829//cytosol	GO:0071885//N-terminal protein N-methyltransferase activity	GO:0002181//cytoplasmic translation;GO:0018016//N-terminal peptidyl-proline dimethylation	gi|115492825|ref|XP_001211040.1|/5.80935e-169/conserved hypothetical protein [Aspergillus terreus NIH2624]
4322221	1908	21.31	45.22	1.548606545	0.01283426	0.03842496	Up	ATEG_06444	hypothetical protein	-	-	GO:0003824//catalytic activity	-	gi|115400067|ref|XP_001215622.1|/0/conserved hypothetical protein [Aspergillus terreus NIH2624]
4353399	2449	522.34	987.14	1.546458394	6.46E-16	9.96E-15	Up	ATEG_08080	glutamine-dependent NAD(+) synthetase synthase [glutamine-hydrolyzing])	ko01100//Metabolic pathways;ko00760//Nicotinate and nicotinamide metabolism	-	GO:0003952//NAD+ synthase (glutamine-hydrolyzing) activity;GO:0005524//ATP binding;GO:0016810//hydrolase activity, acting on carbon-nitrogen (but not peptide) bonds	GO:0009435//NAD biosynthetic process	gi|115433128|ref|XP_001216701.1|/0/glutamine-dependent NAD(+) synthetase synthase [glutamine-hydrolyzing]) [Aspergillus terreus NIH2624]

4353582	1851	21.29	44.69	1.5447866	0.01136603	0.03464829	Up	ATEG_08206	hypothetical protein	ko01100//Metabolic pathways;ko00600//Sphingolipid metabolism	-	GO:0004767//sphingomyelin phosphodiesterase activity;GO:0016798//hydrolase activity, acting on glycosyl bonds	GO:0006685//sphingomyelin catabolic process	gi|115433380|ref|XP_001216827.1|/0/predicted protein [Aspergillus terreus NIH2624]
4354903	1254	672.38	1279.9	1.544470543	6.16E-19	1.15E-17	Up	ATEG_00146	hypothetical protein	ko01100//Metabolic pathways;ko01110//Biosynthesis of secondary metabolites	-	GO:0005506//iron ion binding;GO:0016708//oxidoreductase activity, acting on paired donors, with incorporation or reduction of molecular oxygen, NAD(P)H as one donor, and incorporation of two atoms of oxygen into one donor;GO:0051537//2 iron, 2 sulfur cluster binding	GO:0019439//aromatic compound catabolic process;GO:0055114//oxidation-reduction process	gi|115491209|ref|XP_001210232.1|/0/conserved hypothetical protein [Aspergillus terreus NIH2624]
4318803	2289	100.86	193.95	1.543352974	5.22E-06	3.25E-05	Up	ATEG_03689	hypothetical protein	-	-	-	-	gi|115390725|ref|XP_001212867.1|/0/conserved hypothetical protein [Aspergillus terreus NIH2624]
4317384	1118	10329.74	19393.74	1.537979269	2.14E-44	1.22E-42	Up	ATEG_03080	40S ribosomal protein S12	ko03010//Ribosome	GO:0022627//cytosolic small ribosomal subunit	GO:0003735//structural constituent of ribosome	GO:0006412//translation	gi|115389506|ref|XP_001212258.1|/5.02438e-106/40S ribosomal protein S12 [Aspergillus terreus NIH2624]
4318101	881	6026.35	11323.73	1.535612381	3.37E-36	1.47E-34	Up	ATEG_03303	40S ribosomal protein S10-A	ko03010//Ribosome	GO:0005840//ribosome	-	-	gi|115389952|ref|XP_001212481.1|/5.31309e-109/40S ribosomal protein S10-A [Aspergillus terreus NIH2624]

4320099	2385	150.8	287.27	1.535190876	7.66E-08	6.13E-07	Up	ATEG_04854	hypothetical protein	-	GO:0005634//nucleus	GO:0000981//RNA polymerase II transcription factor activity, sequence-specific DNA binding;GO:0008270//zinc ion binding	GO:0006357//regulation of transcription from RNA polymerase II promoter	gi|115396786|ref|XP_001214032.1|/0/predicted protein [Aspergillus terreus NIH2624]
4316233	3346	6333.78	11865.47	1.534167792	1.30E-40	6.53E-39	Up	ATEG_01624	60S ribosomal protein L33	ko03010//Ribosome	GO:0022625//cytosolic large ribosomal subunit	GO:0003735//structural constituent of ribosome	GO:0002181//cytoplasmic translation;GO:0042273//ribosomal large subunit biogenesis	gi|115384884|ref|XP_001208989.1|/2.8683e-76/60S ribosomal protein L33 [Aspergillus terreus NIH2624]
4316651	2244	34.15	69.44	1.533401132	0.00506048	0.01724754	Up	ATEG_02312	alpha-galactosidase C precursor	ko00561//Glycerolipid metabolism;ko00052//Galactose metabolism;ko00600//Sphingolipid metabolism;ko00603//Glycosphingolipid biosynthesis - globo series	GO:0005576//extracellular region	GO:0052692//raffinose alpha-galactosidase activity	GO:0000272//polysaccharide catabolic process	gi|115387969|ref|XP_001211490.1|/0/alpha-galactosidase C precursor [Aspergillus terreus NIH2624]

4317565	3871	3710.72	6932.93	1.532940438	2.30E-34	9.06E-33	Up	ATEG_02982	serine/threonine-protein kinase sck1	-	GO:0000329//fungal-type vacuole membrane;GO:0005634//nucleus	GO:0004674//protein serine/threonine kinase activity;GO:0005524//ATP binding	GO:0001302//replicative cell aging;GO:0001324//age-dependent response to oxidative stress involved in chronological cell aging;GO:0006468//protein phosphorylation;GO:0032880//regulation of protein localization;GO:0043619//regulation of transcription from RNA polymerase II promoter in response to oxidative stress;GO:0045943//positive regulation of transcription from RNA polymerase I promoter;GO:0045945//positive regulation of transcription from RNA polymerase III promoter;GO:0047484//regulation of response to osmotic stress;GO:006096[illegible]	gi|115389310|ref|XP_001212160.1|/0/serine/threonine-protein kinase sck1 [Aspergillus terreus NIH2624]
4355119	2247	60.52	118.01	1.532414898	8.61E-05	0.00043747	Up	ATEG_00367	hypothetical protein	-	GO:0005634//nucleus	GO:0003677//DNA binding;GO:0008270//zinc ion binding	GO:0006351//transcription, DNA-templated	gi|115491651|ref|XP_001210453.1|/0/conserved hypothetical protein [Aspergillus terreus NIH2624]

4317038	1153	9786.03	18290.79	1.530049964	4.17E-37	1.91E-35	Up	ATEG_02739	60S ribosomal protein L13	ko03010//Ribosome	GO:0005829//cytosol;GO:0005840//ribosome	GO:0003735//structural constituent of ribosome	GO:0006412//translation	gi|115388823|ref|XP_001211917.1|/4.41307e-158/60S ribosomal protein L13 [Aspergillus terreus NIH2624]
4320509	690	20.72	44.36	1.529076632	0.01420632	0.04194397	Up	ATEG_04862	hypothetical protein	ko04144//Endocytosis	-	-	-	gi|115396802|ref|XP_001214040.1|/2.36377e-165/predicted protein [Aspergillus terreus NIH2624]
4353072	1689	136.69	261.51	1.527830375	3.88E-07	2.87E-06	Up	ATEG_08174	hypothetical protein	-	GO:0005634//nucleus	GO:0000981//RNA polymerase II transcription factor activity, sequence-specific DNA binding;GO:0008270//zinc ion binding	GO:0006357//regulation of transcription from RNA polymerase II promoter	gi|115433316|ref|XP_001216795.1|/0/predicted protein [Aspergillus terreus NIH2624]
4355399	3487	71.72	137.66	1.526369343	5.10E-05	0.00027016	Up	ATEG_00644	hypothetical protein	ko01100//Metabolic pathways;ko01130//Biosynthesis of antibiotics;ko00311//Penicillin and cephalosporin biosynthesis	GO:0016021//integral component of membrane	GO:0003824//catalytic activity	GO:0008152//metabolic process	gi|115492205|ref|XP_001210730.1|/0/conserved hypothetical protein [Aspergillus terreus NIH2624]
4318004	1086	113.17	215.59	1.524318323	9.53E-07	6.57E-06	Up	ATEG_03124	hypothetical protein	ko04120//Ubiquitin mediated proteolysis	GO:0016021//integral component of membrane	-	-	gi|115389594|ref|XP_001212302.1|/0/predicted protein [Aspergillus terreus NIH2624]

4318165	1425	982.15	1826.33	1.523371698	2.23E-18	4.03E-17	Up	ATEG_03847	hypothetical protein	ko01100//Metabolic pathways;ko00350//Tyrosine metabolism;ko00650//Butanoate metabolism;ko00310//Lysine degradation;ko00250//Alanine, aspartate and glutamate metabolism	-	GO:0016620//oxidoreductase activity, acting on the aldehyde or oxo group of donors, NAD or NADP as acceptor	GO:0055114//oxidation-reduction process	gi|115391041|ref|XP_001213025.1|/0/conserved hypothetical protein [Aspergillus terreus NIH2624]
4320412	1260	136.16	257.03	1.522670321	8.42E-08	6.70E-07	Up	ATEG_04413	similar to : Metal-dependent amidase/aminoacylase/carboxypeptidase	ko01100//Metabolic pathways;ko00520//Amino sugar and nucleotide sugar metabolism	-	GO:0004180//carboxypeptidase activity	GO:0006508//proteolysis	gi|115395904|ref|XP_001213591.1|/0/hypothetical protein ATEG_04413 [Aspergillus terreus NIH2624]
4316202	408	69.5	135.74	1.522162669	0.00010677	0.00053175	Up	ATEG_01377	hypothetical protein	-	-	-	-	gi|115384390|ref|XP_001208742.1|/4.9988e-28/conserved hypothetical protein [Aspergillus terreus NIH2624]
4353491	1530	234.31	439.55	1.521327651	1.29E-10	1.36E-09	Up	ATEG_08225	similar to aminotriazole resistance protein	-	GO:0016021//integral component of membrane	-	GO:0055085//transmembrane transport	gi|115433418|ref|XP_001216846.1|/0/hypothetical protein ATEG_08225 [Aspergillus terreus NIH2624]
4321870	2881	346.83	647.5	1.520688776	6.08E-14	8.21E-13	Up	ATEG_05790	similar to Pik3c3 protein	ko04011//MAPK signaling pathway - yeast	GO:0016021//integral component of membrane	GO:0003824//catalytic activity;GO:0005452//inorganic anion exchanger activity	GO:0015698//inorganic anion transport	gi|115398758|ref|XP_001214968.1|/0/hypothetical protein ATEG_05790 [Aspergillus terreus NIH2624]

4353388	1463	2087.95	3860.18	1.520614132	2.58E-18	4.62E-17	Up	ATEG_08120	aldehyde dehydrogenase 9	ko01100//Metabolic pathways;ko01110//Biosynthesis of secondary metabolites;ko01130//Biosynthesis of antibiotics;ko00380//Tryptophan metabolism;ko00010//Glycolysis / Gluconeogenesis;ko00620//Pyruvate metabolism;ko00561//Glycerolipid metabolism;ko00071//Fatty acid degradation;ko00310//Lysine degradation;ko00280//Valine, leucine and isoleucine degradation;ko00330//Arginine and proline metabolism;ko00040//Pentose and glucuronate interconversions;ko00410//beta-Alanine metabolism;ko00340//Histidine metabolism;ko00053//Ascorbate and	-	GO:0016620//oxidoreductase activity, acting on the aldehyde or oxo group of donors, NAD or NADP as acceptor	GO:0055114//oxidation-reduction process	gi|115433208|ref|XP_001216741.1|/0/aldehyde dehydrogenase 9 [Aspergillus terreus NIH2624]
4355113	1851	25.78	52.85	1.520327872	0.00637742	0.02107762	Up	ATEG_00361	hypothetical protein	-	-	GO:0004499//N,N-dimethylaniline monooxygenase activity;GO:0050660//flavin adenine dinucleotide binding;GO:0050661//NADP binding	GO:0055114//oxidation-reduction process	gi|115491639|ref|XP_001210447.1|/0/conserved hypothetical protein [Aspergillus terreus NIH2624]

4316312	1337	7895.26	14636.31	1.517827579	2.08E-41	1.07E-39	Up	ATEG_01126	60S ribosomal protein L3	ko03010//Ribosome	GO:0022625//cytosolic large ribosomal subunit	GO:0003735//structural constituent of ribosome	GO:0000027//ribosomal large subunit assembly;GO:0006412//translation	gi|115383888|ref|XP_001208491.1|/0/60S ribosomal protein L3 [Aspergillus terreus NIH2624]
4322999	1236	32.5	64.3	1.510711178	0.0027597	0.01013872	Up	ATEG_07863	hypothetical protein	ko04144//Endocytosis	-	-	-	gi|115401792|ref|XP_001216484.1|/0/predicted protein [Aspergillus terreus NIH2624]
4316296	879	125.56	236.15	1.507488573	3.90E-07	2.88E-06	Up	ATEG_01748	hypothetical protein	ko01100//Metabolic pathways;ko00561//Glycerolipid metabolism	-	GO:0016787//hydrolase activity	GO:0008152//metabolic process	gi|115385132|ref|XP_001209113.1|/0/conserved hypothetical protein [Aspergillus terreus NIH2624]
4323484	1140	34.21	73.61	1.507445128	0.01722708	0.04972062	Up	ATEG_08989	hypothetical protein	ko00920//Sulfur metabolism	GO:0005762//mitochondrial large ribosomal subunit	GO:0003735//structural constituent of ribosome	-	gi|115402997|ref|XP_001217575.1|/0/conserved hypothetical protein [Aspergillus terreus NIH2624]
4318243	978	1157.45	2123.95	1.506987146	3.37E-19	6.52E-18	Up	ATEG_03892	similar to NADP(+)-dependent glycerol dehydrogenase	ko01100//Metabolic pathways;ko00561//Glycerolipid metabolism	GO:0005576//extracellular region;GO:0005622//intracellular	GO:0016491//oxidoreductase activity	GO:0006071//glycerol metabolic process;GO:0006973//intracellular accumulation of glycerol;GO:0055114//oxidation-reduction process	gi|115391131|ref|XP_001213070.1|/0/hypothetical protein ATEG_03892 [Aspergillus terreus NIH2624]
4317859	1212	53.28	103.4	1.506024697	0.00113123	0.0045662	Up	ATEG_03015	hypothetical protein	ko01100//Metabolic pathways;ko00500//Starch and sucrose metabolism	-	GO:0051213//dioxygenase activity	GO:0055114//oxidation-reduction process	gi|115389376|ref|XP_001212193.1|/0/conserved hypothetical protein [Aspergillus terreus NIH2624]
4322827	507	92.48	177.54	1.504158974	3.93E-05	0.000214	Up	ATEG_07644	hypothetical protein	ko00480//Glutathione metabolism	-	-	-	gi|115401354|ref|XP_001216265.1|/6.90148e-119/predicted protein [Aspergillus terreus NIH2624]

4316139	1679	1386.94	2539.13	1.502945327	9.56E-26	2.70E-24	Up	ATEG_01526	hypothetical protein	-	GO:0016021//integral component of membrane	-	-	gi|115384688|ref|XP_001208891.1|/0/predicted protein [Aspergillus terreus NIH2624]
4354397	321	1749.77	3227.73	1.500560959	1.02E-21	2.30E-20	Up	ATEG_09858	60S ribosomal protein L30	ko03010//Ribosome	GO:0022625//cytosolic large ribosomal subunit	GO:0003735//structural constituent of ribosome;GO:0030627//pre-mRNA 5'-splice site binding	GO:0006364//rRNA processing;GO:0006412//translation;GO:0048025//negative regulation of mRNA splicing, via spliceosome	gi|115443346|ref|XP_001218480.1|/5.88604e-70/60S ribosomal protein L30 [Aspergillus terreus NIH2624]
4354935	2288	355.35	654.63	1.499331954	7.63E-14	1.02E-12	Up	ATEG_00178	hypothetical protein	-	-	GO:0003676//nucleic acid binding;GO:0046872//metal ion binding	-	gi|115491273|ref|XP_001210264.1|/0/conserved hypothetical protein [Aspergillus terreus NIH2624]
4315741	693	205.63	380.16	1.497865676	9.80E-10	9.44E-09	Up	ATEG_01398	hypothetical protein	-	-	-	-	gi|115384432|ref|XP_001208763.1|/7.38378e-174/conserved hypothetical protein [Aspergillus terreus NIH2624]
4353292	1953	39.77	77.5	1.496269761	0.00173623	0.00671797	Up	ATEG_08367	similar to PycA	ko01100//Metabolic pathways;ko01230//Biosynthesis of amino acids;ko01200//Carbon metabolism;ko00620//Pyruvate metabolism;ko00020//Citrate cycle (TCA cycle)	-	GO:0004075//biotin carboxylase activity;GO:0005524//ATP binding;GO:0046872//metal ion binding	-	gi|115433702|ref|XP_001216988.1|/0/hypothetical protein ATEG_08367 [Aspergillus terreus NIH2624]

4320157	1281	45.95	88.34	1.492696196	0.00072386	0.00305534	Up	ATEG_04914	hypothetical protein	ko01100//Metabolic pathways;ko00520//Amino sugar and nucleotide sugar metabolism	-	GO:0004519//endonuclease activity;GO:0004527//exonuclease activity	GO:0090305//nucleic acid phosphodiester bond hydrolysis	gi|115396906|ref|XP_001214092.1|/0/conserved hypothetical protein [Aspergillus terreus NIH2624]
4317220	1043	685.97	1250.48	1.491108599	1.02E-20	2.18E-19	Up	ATEG_02703	hypothetical protein	ko01100//Metabolic pathways;ko01110//Biosynthesis of secondary metabolites;ko01130//Biosynthesis of antibiotics;ko01230//Biosynthesis of amino acids;ko01200//Carbon metabolism;ko00260//Glycine, serine and threonine metabolism;ko00010//Glycolysis / Gluconeogenesis; ko00680//Methane metabolism	-	-	-	gi|115388751|ref|XP_001211881.1|/2.46908e-174/conserved hypothetical protein [Aspergillus terreus NIH2624]
4320311	1535	43.14	82.59	1.490342485	0.00116777	0.00469967	Up	ATEG_04319	alcohol dehydrogenase II	ko01100//Metabolic pathways;ko01110//Biosynthesis of secondary metabolites;ko01130//Biosynthesis of antibiotics;ko01220//Degradation of aromatic compounds;ko00350//Tyrosine metabolism;ko00010//Glycolysis / Gluconeogenesis; ko00071//Fatty acid degradation	-	GO:0004022//alcohol dehydrogenase (NAD) activity;GO:0008270//zinc ion binding	GO:0055114//oxidation-reduction process	gi|115395344|ref|XP_001213497.1|/0/alcohol dehydrogenase II [Aspergillus terreus NIH2624]

4323275	1170	201.84	375.65	1.490335587	2.29E-07	1.73E-06	Up	ATEG_08556	hypothetical protein	ko01100//Metabolic pathways;ko00520//Amino sugar and nucleotide sugar metabolism	-	-	-	gi|115402131|ref|XP_001217142.1|/0/predicted protein [Aspergillus terreus NIH2624]
4321619	1491	63.33	118.77	1.48851833	0.00019731	0.00094072	Up	ATEG_06193	hypothetical protein	-	GO:0016021//integral component of membrane	GO:0015238//drug transmembrane transporter activity;GO:0015297//antiporter activity	GO:0006855//drug transmembrane transport	gi|115399564|ref|XP_001215371.1|/0/conserved hypothetical protein [Aspergillus terreus NIH2624]
4322328	3485	1297.48	2358.63	1.488385718	1.02E-25	2.88E-24	Up	ATEG_06790	NAD-specific glutamate dehydrogenase	ko01100//Metabolic pathways;ko00250//Alanine, aspartate and glutamate metabolism;ko00220//Arginine biosynthesis;ko00910//Nitrogen metabolism;ko00430//Taurine and hypotaurine metabolism	GO:0005739//mitochondrion;GO:0005829//cytosol	GO:0004352//glutamate dehydrogenase (NAD+) activity	GO:0019551//glutamate catabolic process to 2-oxoglutarate;GO:0055114//oxidation-reduction process	gi|115400759|ref|XP_001215968.1|/0/NAD-specific glutamate dehydrogenase [Aspergillus terreus NIH2624]
4353539	432	85.2	158.52	1.487434377	2.45E-05	0.00013773	Up	ATEG_08216	hypothetical protein	-	-	-	-	gi|115433400|ref|XP_001216837.1|/1.3576e-106/predicted protein [Aspergillus terreus NIH2624]
4318329	1893	229.25	419.83	1.486765083	2.39E-10	2.44E-09	Up	ATEG_03977	transporter protein SMF2	-	GO:0016021//integral component of membrane	GO:0005215//transporter activity	GO:0006810//transport	gi|115391301|ref|XP_001213155.1|/0/transporter protein SMF2 [Aspergillus terreus NIH2624]
4318410	714	5201.25	9410.82	1.483860798	7.74E-37	3.46E-35	Up	ATEG_03989	40S ribosomal protein S6	ko03010//Ribosome	GO:0005840//ribosome	GO:0003735//structural constituent of ribosome	GO:0006412//translation	gi|115391325|ref|XP_001213167.1|/1.12222e-167/40S ribosomal protein S6 [Aspergillus terreus NIH2624]

4354610	4290	54.38	106.85	1.481621944	0.00237199	0.0088706	Up	ATEG_10369	similar to ABC transporter	ko02010//ABC transporters	GO:0016021//integral component of membrane	GO:0005524//ATP binding;GO:0042626//ATPase activity, coupled to transmembrane movement of substances	GO:0055085//transmembrane transport	gi|115449867|ref|XP_001218717.1|/0/hypothetical protein ATEG_10369 [Aspergillus terreus NIH2624]
4353296	1326	38.11	74.19	1.479965718	0.00260831	0.00963912	Up	ATEG_08425	hypothetical protein	-	GO:0016021//integral component of membrane	-	GO:0035690//cellular response to drug;GO:0044550//secondary metabolite biosynthetic process;GO:0055085//transmembrane transport	gi|115433819|ref|XP_001217046.1|/0/predicted protein [Aspergillus terreus NIH2624]
4353565	2064	1601	2885.5	1.476985843	2.08E-15	3.06E-14	Up	ATEG_08102	peroxisomal copper amine oxidase	ko01100//Metabolic pathways;ko01110//Biosynthesis of secondary metabolites;ko00350//Tyrosine metabolism;ko00260//Glycine, serine and threonine metabolism;ko00360//Phenylalanine metabolism;ko00410//beta-Alanine metabolism	-	GO:0005507//copper ion binding;GO:0008131//primary amine oxidase activity;GO:0048038//quinone binding	GO:0009308//amine metabolic process;GO:0055114//oxidation-reduction process	gi|115433172|ref|XP_001216723.1|/0/peroxisomal copper amine oxidase [Aspergillus terreus NIH2624]

4318597	1836	220.77	399.83	1.471420528	3.99E-10	3.99E-09	Up	ATEG_04163	similar to CDC14	ko04111//Cell cycle - yeast;ko04113//Meiosis - yeast	GO:0000778//condensed nuclear chromosome kinetochore;GO:0005826//actomyosin contractile ring;GO:0030869//RENT complex;GO:0044732//mitotic spindle pole body;GO:1990023//mitotic spindle midzone	GO:0004725//protein tyrosine phosphatase activity;GO:0008138//protein tyrosine/serine/threonine phosphatase activity	GO:0006974//cellular response to DNA damage stimulus;GO:0007034//vacuolar transport;GO:0031031//positive regulation of septation initiation signaling;GO:0031536//positive regulation of exit from mitosis;GO:0034501//protein localization to kinetochore;GO:0035335//peptidyl-tyrosine dephosphorylation;GO:0035853//chromosome passenger complex localization to spindle midzone;GO:0044878//mitotic cytokinesis checkpoint;GO:0045736//negative regulation of cyclin-dependent protein serine/threonine kinase activity;GO:0051229//meiotic	gi|115391673|ref|XP_001213341.1|/0/hypothetical protein ATEG_04163 [Aspergillus terreus NIH2624]
4319300	435	76.2	140.77	1.469413138	5.15E-05	0.0002724	Up	ATEG_06853	hypothetical protein	-	-	-	-	gi|115385996|ref|XP_001209538.1|/2.25098e-101/predicted protein [Aspergillus terreus NIH2624]

4355075	480	2248.24	3988.52	1.462878016	2.07E-17	3.50E-16	Up	ATEG_00323	hypothetical protein	ko04144//Endocytosis	-	-	-	gi|115491563|ref|XP_001210409.1|/1.15468e-99/predicted protein [Aspergillus terreus NIH2624]
4354424	1184	121.05	219.3	1.461394229	1.02E-06	6.96E-06	Up	ATEG_09834	hypothetical protein	-	-	GO:0008757//S-adenosylmethionine-dependent methyltransferase activity	GO:0032259//methylation	gi|115443298|ref|XP_001218456.1|/5.37879e-140/conserved hypothetical protein [Aspergillus terreus NIH2624]
4320519	2792	442.19	787.06	1.461374405	1.27E-10	1.34E-09	Up	ATEG_04729	hypothetical protein	ko01100//Metabolic pathways;ko00520//Amino sugar and nucleotide sugar metabolism;ko00531//Glycosaminoglycan degradation;ko01501//beta-Lactam resistance	-	GO:0004553//hydrolase activity, hydrolyzing O-glycosyl compounds;GO:0008080//N-acetyltransferase activity	GO:0005975//carbohydrate metabolic process	gi|115396536|ref|XP_001213907.1|/0/predicted protein [Aspergillus terreus NIH2624]
4353468	1777	68.96	127.15	1.461225049	0.00021223	0.00100521	Up	ATEG_08416	hypothetical protein	-	-	-	-	gi|115433801|ref|XP_001217037.1|/0/predicted protein [Aspergillus terreus NIH2624]
4353751	765	7060.86	12566	1.461125572	8.93E-35	3.58E-33	Up	ATEG_09396	60S ribosomal protein L2	ko03010//Ribosome	GO:0005840//ribosome	GO:0003735//structural constituent of ribosome	GO:0006412//translation	gi|115438248|ref|XP_001218018.1|/0/60S ribosomal protein L2 [Aspergillus terreus NIH2624]
4315968	1314	3682.36	6535.79	1.459874813	1.11E-24	2.94E-23	Up	ATEG_01866	protein MMF1, mitochondrial precursor	ko03015//mRNA surveillance pathway	-	-	-	gi|115385368|ref|XP_001209231.1|/1.61833e-85/protein MMF1, mitochondrial precursor [Aspergillus terreus NIH2624]

4322080	1529	454.89	810.12	1.456558804	5.17E-11	5.63E-10	Up	ATEG_06561	hypothetical protein	ko03040//Spliceosome	GO:0005634//nucleus;GO:0005829//cytosol	-	-	gi|115400301|ref|XP_001215739.1|/0/conserved hypothetical protein [Aspergillus terreus NIH2624]
4323358	713	5266.29	9343.61	1.454949413	1.24E-35	5.28E-34	Up	ATEG_08975	60S ribosomal protein L28	ko03010//Ribosome	GO:0005634//nucleus;GO:0015934//large ribosomal subunit	GO:0003723//RNA binding;GO:0003735//structural constituent of ribosome	GO:0006412//translation	gi|115402969|ref|XP_001217561.1|/2.70122e-109/60S ribosomal protein L28 [Aspergillus terreus NIH2624]
4320803	2147	447.93	796.56	1.451741817	1.20E-11	1.39E-10	Up	ATEG_05599	hypothetical protein	-	GO:0016021//integral component of membrane	GO:0015171//amino acid transmembrane transporter activity	GO:0003333//amino acid transmembrane transport	gi|115398371|ref|XP_001214777.1|/0/conserved hypothetical protein [Aspergillus terreus NIH2624]
4353110	3117	3279.43	5802.47	1.451480146	3.69E-27	1.12E-25	Up	ATEG_08137	inositol-3-phosphate synthase	ko01100//Metabolic pathways;ko01130//Biosynthesis of antibiotics;ko00562//Inositol phosphate metabolism	-	GO:0004512//inositol-3-phosphate synthase activity	GO:0006021//inositol biosynthetic process;GO:0008654//phospholipid biosynthetic process	gi|115433242|ref|XP_001216758.1|/0/inositol-3-phosphate synthase [Aspergillus terreus NIH2624]
4322138	456	1965.63	3495.07	1.451221072	2.52E-24	6.47E-23	Up	ATEG_06458	40S ribosomal protein S13	ko03010//Ribosome	GO:0022627//cytosolic small ribosomal subunit	GO:0003735//structural constituent of ribosome;GO:0070181//small ribosomal subunit rRNA binding	GO:0000462//maturation of SSU-rRNA from tricistronic rRNA transcript (SSU-rRNA, 5.8S rRNA, LSU-rRNA);GO:0006412//translation	gi|115400095|ref|XP_001215636.1|/3.39653e-104/40S ribosomal protein S13 [Aspergillus terreus NIH2624]

4320641	1695	772.28	1372.53	1.445943301	1.71E-17	2.91E-16	Up	ATEG_05389	threonine dehydratase, mitochondrial precursor	ko01100//Metabolic pathways;ko01110//Biosynthesis of secondary metabolites;ko01130//Biosynthesis of antibiotics;ko01230//Biosynthesis of amino acids;ko01200//Carbon metabolism;ko00260//Glycine, serine and threonine metabolism;ko00290//Valine, leucine and isoleucine biosynthesis	-	GO:0004794//L-threonine ammonia-lyase activity;GO:0030170//pyridoxal phosphate binding	GO:0009097//isoleucine biosynthetic process	gi|115397951|ref|XP_001214567.1|/0/threonine dehydratase, mitochondrial precursor [Aspergillus terreus NIH2624]
4355033	8325	31.98	61.65	1.443299827	0.01158267	0.03516021	Up	ATEG_00282	similar to polyketide synthase	-	-	GO:0016491//oxidoreductase activity;GO:0016740//transferase activity;GO:0031177//phosphopantetheine binding	GO:0055114//oxidation-reduction process	gi|115491481|ref|XP_001210368.1|/0/hypothetical protein ATEG_00282 [Aspergillus terreus NIH2624]
4320687	3245	1046.47	1842.86	1.442673551	3.42E-20	7.05E-19	Up	ATEG_05333	3-phytase A precursor	ko00562//Inositol phosphate metabolism	GO:0005576//extracellular region	GO:0003993//acid phosphatase activity;GO:0016158//3-phytase activity	GO:0016311//dephosphorylation	gi|115397839|ref|XP_001214511.1|/0/3-phytase A precursor [Aspergillus terreus NIH2624]
4321481	1717	110.41	197.16	1.441686218	1.20E-05	7.03E-05	Up	ATEG_05800	hypothetical protein	-	GO:0005618//cell wall	GO:0004553//hydrolase activity, hydrolyzing O-glycosyl compounds	GO:0005975//carbohydrate metabolic process;GO:0071555//cell wall organization	gi|115398778|ref|XP_001214978.1|/0/conserved hypothetical protein [Aspergillus terreus NIH2624]

4355092	1685	345.79	608.67	1.440680438	9.97E-12	1.16E-10	Up	ATEG_00340	hypothetical protein	-	-	GO:0019904//protein domain specific binding	-	gi|115491597|ref|XP_001210426.1|/0/conserved hypothetical protein [Aspergillus terreus NIH2624]
4354927	870	84.66	152.71	1.438820586	0.00010839	0.00053955	Up	ATEG_00170	hypothetical protein	-	-	GO:0050086//mannitol 2-dehydrogenase activity	GO:0055114//oxidation-reduction process	gi|115491257|ref|XP_001210256.1|/0/conserved hypothetical protein [Aspergillus terreus NIH2624]
4354966	1040	181.57	321.07	1.437519409	3.04E-08	2.53E-07	Up	ATEG_00217	hypothetical protein	ko01100//Metabolic pathways;ko00750//Vitamin B6 metabolism	GO:0005576//extracellular region	-	-	gi|115491351|ref|XP_001210303.1|/0/predicted protein [Aspergillus terreus NIH2624]
4354853	1773	72.31	131.06	1.4365584	0.00012932	0.00063482	Up	ATEG_00096	hypothetical protein	ko03440//Homologous recombination;ko03450//Non-homologous end-joining	GO:0016021//integral component of membrane	-	GO:0055085//transmembrane transport	gi|115491109|ref|XP_001210182.1|/0/conserved hypothetical protein [Aspergillus terreus NIH2624]
4354157	526	2040.1	3585.5	1.43538256	4.53E-24	1.14E-22	Up	ATEG_09532	40S ribosomal protein S21	ko03010//Ribosome	GO:0005634//nucleus;GO:0022627//cytosolic small ribosomal subunit	GO:0003735//structural constituent of ribosome	GO:0000447//endonucleolytic cleavage in ITS1 to separate SSU-rRNA from 5.8S rRNA and LSU-rRNA from tricistronic rRNA transcript (SSU-rRNA, 5.8S rRNA, LSU-rRNA);GO:0000461//endonucleolytic cleavage to generate mature 3'-end of SSU-rRNA from (SSU-rRNA, 5.8S rRNA, LSU-rRNA);GO:0006412//translation	gi|67526731|ref|XP_661427.1|/2.18554e-59/hypothetical protein AN3823.2 [Aspergillus nidulans FGSC A4]

4316732	908	40.36	74.57	1.43464435	0.00254044	0.009418	Up	ATEG_02272	hypothetical protein	-	-	-	-	gi|115387889|ref|XP_001211450.1|/3.91935e-156/predicted protein [Aspergillus terreus NIH2624]
4320069	2218	803.14	1401.5	1.434291055	2.79E-16	4.42E-15	Up	ATEG_04370	hypothetical protein	-	-	-	-	gi|115395818|ref|XP_001213548.1|/0/predicted protein [Aspergillus terreus NIH2624]
4319334	1212	131.68	232.93	1.432934235	4.59E-06	2.88E-05	Up	ATEG_07183	similar to oxidoreductase	ko00620//Pyruvate metabolism	-	GO:0010181//FMN binding;GO:0016491//oxidoreductase activity	GO:0055114//oxidation-reduction process	gi|115386656|ref|XP_001209869.1|/0/hypothetical protein ATEG_07183 [Aspergillus terreus NIH2624]
4317602	1539	1772.4	3085.76	1.432501441	7.40E-18	1.28E-16	Up	ATEG_03397	glutamate decarboxylase	ko01100//Metabolic pathways;ko01110//Biosynthesis of secondary metabolites;ko00650//Butanoate metabolism;ko00250//Alanine, aspartate and glutamate metabolism;ko00410//beta-Alanine metabolism;ko00430//Taurine and hypotaurine metabolism	-	GO:0004351//glutamate decarboxylase activity;GO:0030170//pyridoxal phosphate binding	GO:0006538//glutamate catabolic process;GO:0009448//gamma-aminobutyric acid metabolic process	gi|115390140|ref|XP_001212575.1|/0/glutamate decarboxylase [Aspergillus terreus NIH2624]
4323371	2945	636.31	1124.72	1.432496945	3.96E-11	4.36E-10	Up	ATEG_08653	similar to monosaccharide transporter	ko04113//Meiosis - yeast	GO:0016021//integral component of membrane	GO:0022891//substrate-specific transmembrane transporter activity	GO:0055085//transmembrane transport	gi|115402325|ref|XP_001217239.1|/0/hypothetical protein ATEG_08653 [Aspergillus terreus NIH2624]

4321432	1617	193.35	340.07	1.427494609	8.84E-09	7.76E-08	Up	ATEG_05680	hypothetical protein	-	GO:0016021//integral component of membrane	GO:0022891//substrate-specific transmembrane transporter activity	GO:0008643//carbohydrate transport;GO:0055085//transmembrane transport	gi|115398538|ref|XP_001214858.1|/0/conserved hypothetical protein [Aspergillus terreus NIH2624]
4355356	720	96.39	172.19	1.425826535	1.58E-05	9.08E-05	Up	ATEG_00602	diphthine synthase	-	GO:0005634//nucleus;GO:0005829//cytosol	GO:0004164//diphthine synthase activity	GO:0017183//peptidyl-diphthamide biosynthetic process from peptidyl-histidine;GO:0032259//methylation	gi|115492121|ref|XP_001210688.1|/5.177e-174/diphthine synthase [Aspergillus terreus NIH2624]
4316663	1153	116.06	205.92	1.42461964	1.50E-05	8.71E-05	Up	ATEG_02299	hypothetical protein	-	GO:0016021//integral component of membrane	-	GO:0055085//transmembrane transport	gi|115387943|ref|XP_001211477.1|/7.36669e-105/predicted protein [Aspergillus terreus NIH2624]
4321229	3201	387.2	675.4	1.423224709	2.03E-13	2.63E-12	Up	ATEG_05041	hypothetical protein	-	GO:0016021//integral component of membrane	GO:0005216//ion channel activity	GO:0034220//ion transmembrane transport	gi|115397255|ref|XP_001214219.1|/0/conserved hypothetical protein [Aspergillus terreus NIH2624]
4315863	402	1338.06	2319.83	1.418502095	5.91E-23	1.42E-21	Up	ATEG_01848	40S ribosomal protein S24	ko03010//Ribosome	GO:0005840//ribosome	GO:0000166//nucleotide binding;GO:0003735//structural constituent of ribosome	GO:0006412//translation	gi|115385332|ref|XP_001209213.1|/1.34619e-90/40S ribosomal protein S24 [Aspergillus terreus NIH2624]
4322753	1112	557.42	966.55	1.41818959	1.82E-12	2.24E-11	Up	ATEG_07417	hypothetical protein	ko03420//Nucleotide excision repair;ko03022//Basal transcription factors	-	GO:0016787//hydrolase activity	GO:0008152//metabolic process	gi|115400900|ref|XP_001216038.1|/0/conserved hypothetical protein [Aspergillus terreus NIH2624]
4318750	1440	37	67.64	1.414716588	0.00516886	0.01757864	Up	ATEG_03875	hypothetical protein	-	-	GO:0004722//protein serine/threonine phosphatase activity	GO:0006470//protein dephosphorylation	gi|115391097|ref|XP_001213053.1|/0/predicted protein [Aspergillus terreus NIH2624]

4322417	1611	30.83	56.92	1.412209537	0.00850463	0.02700342	Up	ATEG_06453	hypothetical protein	ko04113//Meiosis - yeast	GO:0016021//integral component of membrane	GO:0022891//substrate-specific transmembrane transporter activity	GO:0008643//carbohydrate transport;GO:0055085//transmembrane transport	gi|115400085|ref|XP_001215631.1|/0/predicted protein [Aspergillus terreus NIH2624]
4319111	1497	59.95	106.59	1.409071704	0.0008129	0.00338096	Up	ATEG_07314	hypothetical protein	-	-	-	-	gi|115386918|ref|XP_001210000.1|/0/predicted protein [Aspergillus terreus NIH2624]
4317798	1296	91.9	160.92	1.4079651	0.00017406	0.00083884	Up	ATEG_03464	hypothetical protein	ko01100//Metabolic pathways;ko00500//Starch and sucrose metabolism	GO:0016021//integral component of membrane	-	-	gi|115390274|ref|XP_001212642.1|/0/conserved hypothetical protein [Aspergillus terreus NIH2624]
4322036	2434	232.56	402.57	1.404791444	1.18E-09	1.13E-08	Up	ATEG_06552	hypothetical protein	-	GO:0005634//nucleus	GO:0003677//DNA binding;GO:0008270//zinc ion binding	GO:0006351//transcription, DNA-templated	gi|115400283|ref|XP_001215730.1|/0/predicted protein [Aspergillus terreus NIH2624]
4322721	1584	329.94	567.74	1.402926357	2.55E-08	2.13E-07	Up	ATEG_07797	hypothetical protein	ko00254//Aflatoxin biosynthesis	-	GO:0004497//monooxygenase activity;GO:0005506//iron ion binding;GO:0016705//oxidoreductase activity, acting on paired donors, with incorporation or reduction of molecular oxygen;GO:0020037//heme binding	GO:0055114//oxidation-reduction process	gi|115401660|ref|XP_001216418.1|/0/conserved hypothetical protein [Aspergillus terreus NIH2624]
4323457	579	3112.52	5322.31	1.401657058	2.21E-26	6.45E-25	Up	ATEG_08535	60S ribosomal protein L9	ko03010//Ribosome	GO:0005840//ribosome	GO:0003735//structural constituent of ribosome;GO:0019843//rRNA binding	GO:0006412//translation	gi|115402089|ref|XP_001217121.1|/1.45252e-134/60S ribosomal protein L9 [Aspergillus terreus NIH2624]

4321570	1474	128.35	222.6	1.399101767	2.17E-06	1.44E-05	Up	ATEG_05770	hypothetical protein	ko01100//Metabolic pathways;ko00563//Glycosylphosphatidylinositol(GPI)-anchor biosynthesis	GO:0016021//integral component of membrane	GO:0004584//dolichyl-phosphate-mannose-glycolipid alpha-mannosyltransferase activity	GO:0006506//GPI anchor biosynthetic process;GO:0097502//mannosylation	gi|115398718|ref|XP_001214948.1|/0/predicted protein [Aspergillus terreus NIH2624]
4355569	1620	45.99	84.69	1.397425019	0.00576078	0.01926351	Up	ATEG_00808	hypothetical protein	-	GO:0016021//integral component of membrane	GO:0004497//monooxygenase activity;GO:0005506//iron ion binding;GO:0016705//oxidoreductase activity, acting on paired donors, with incorporation or reduction of molecular oxygen;GO:0020037//heme binding	GO:0055114//oxidation-reduction process	gi|115492533|ref|XP_001210894.1|/0/conserved hypothetical protein [Aspergillus terreus NIH2624]
4320595	711	321.67	548.31	1.394396843	2.48E-10	2.53E-09	Up	ATEG_05558	hypothetical protein	ko01100//Metabolic pathways;ko00040//Pentose and glucuronate interconversions	-	GO:0005524//ATP binding;GO:0016301//kinase activity	GO:0016310//phosphorylation	gi|115398289|ref|XP_001214736.1|/2.27262e-171/conserved hypothetical protein [Aspergillus terreus NIH2624]
4319586	279	788.37	1343.37	1.393230415	6.19E-18	1.08E-16	Up	ATEG_10066	60S ribosomal protein L43	ko03010//Ribosome	GO:0005840//ribosome	GO:0003735//structural constituent of ribosome	GO:0006412//translation	gi|115385643|ref|XP_001209368.1|/7.43098e-62/60S ribosomal protein L43 [Aspergillus terreus NIH2624]
4320085	3351	210.67	360.24	1.390074968	3.45E-08	2.87E-07	Up	ATEG_04375	glucoamylase precursor	ko01100//Metabolic pathways;ko00500//Starch and sucrose metabolism	-	GO:0004339//glucan 1,4-alpha-glucosidase activity;GO:2001070//starch binding	GO:0000272//polysaccharide catabolic process	gi|115395828|ref|XP_001213553.1|/0/glucoamylase precursor [Aspergillus terreus NIH2624]

4355022	1434	3072.21	5192.02	1.390070927	1.04E-25	2.91E-24	Up	ATEG_00271	aminomethyltransferase, mitochondrial precursor	ko01100//Metabolic pathways;ko01110//Biosynthesis of secondary metabolites;ko01130//Biosynthesis of antibiotics;ko01200//Carbon metabolism;ko00260//Glycine, serine and threonine metabolism;ko00630//Glyoxylate and dicarboxylate metabolism;ko00670//One carbon pool by folate	-	GO:0004047//aminomethyltransferase activity;GO:0004375//glycine dehydrogenase (decarboxylating) activity;GO:0008483//transaminase activity	GO:0006546//glycine catabolic process;GO:0006730//one-carbon metabolic process;GO:0032259//methylation;GO:0055114//oxidation-reduction process	gi|115491459|ref|XP_001210357.1|/0/aminomethyltransferase, mitochondrial precursor [Aspergillus terreus NIH2624]
4323069	2346	227.02	391.19	1.389569601	4.30E-08	3.55E-07	Up	ATEG_08647	hypothetical protein	-	GO:0016021//integral component of membrane	GO:0005215//transporter activity	GO:0006810//transport	gi|115402313|ref|XP_001217233.1|/0/conserved hypothetical protein [Aspergillus terreus NIH2624]
4319117	4722	137.95	238.46	1.388742411	4.73E-05	0.00025243	Up	ATEG_07303	hypothetical protein	ko01100//Metabolic pathways;ko01110//Biosynthesis of secondary metabolites;ko00360//Phenylalanine metabolism;ko00130//Ubiquinone and other terpenoid-quinone biosynthesis	GO:0016021//integral component of membrane	GO:0008270//zinc ion binding;GO:0016491//oxidoreductase activity	GO:0006810//transport;GO:0055114//oxidation-reduction process	gi|115386896|ref|XP_001209989.1|/0/predicted protein [Aspergillus terreus NIH2624]
4321494	831	147.38	254.29	1.387502471	7.78E-07	5.44E-06	Up	ATEG_05667	hypothetical protein	ko03018//RNA degradation	GO:0005634//nucleus;GO:0005829//cytosol;GO:0030014//CCR4-NOT complex	GO:0005524//ATP binding;GO:0016887//ATPase activity	GO:0006357//regulation of transcription from RNA polymerase II promoter	gi|115398512|ref|XP_001214845.1|/0/conserved hypothetical protein [Aspergillus terreus NIH2624]

4354976	2069	336.86	575.53	1.387212693	6.64E-11	7.18E-10	Up	ATEG_00227	hypothetical protein	-	GO:0016021//integral component of membrane	-	GO:0055085//transmembrane transport	gi|115491371|ref|XP_001210313.1|/0/conserved hypothetical protein [Aspergillus terreus NIH2624]
4318330	2544	189.95	324.78	1.385614364	5.26E-08	4.30E-07	Up	ATEG_03978	hypothetical protein	-	GO:0005829//cytosol;GO:0032153//cell division site;GO:0051286//cell tip	GO:0003779//actin binding;GO:0017048//Rho GTPase binding	GO:0030036//actin cytoskeleton organization	gi|115391303|ref|XP_001213156.1|/0/conserved hypothetical protein [Aspergillus terreus NIH2624]
4323259	582	4245.3	7195.68	1.383936203	1.64E-23	4.06E-22	Up	ATEG_08725	40S ribosomal protein S9	ko03010//Ribosome	GO:0015935//small ribosomal subunit	GO:0003735//structural constituent of ribosome;GO:0019843//rRNA binding	GO:0006412//translation	gi|115402469|ref|XP_001217311.1|/7.78467e-135/40S ribosomal protein S9 [Aspergillus terreus NIH2624]
4321453	978	76.19	134.23	1.382344396	0.00037842	0.00171118	Up	ATEG_05744	hypothetical protein	-	-	GO:0016787//hydrolase activity	GO:0008152//metabolic process	gi|115398666|ref|XP_001214922.1|/0/predicted protein [Aspergillus terreus NIH2624]
4353698	2294	204.06	348.1	1.381831362	2.42E-07	1.83E-06	Up	ATEG_09075	hypothetical protein	-	GO:0005634//nucleus;GO:0016021//integral component of membrane	-	-	gi|115436988|ref|XP_001217697.1|/0/conserved hypothetical protein [Aspergillus terreus NIH2624]
4355396	693	5070.58	8528.04	1.37907075	9.34E-31	3.22E-29	Up	ATEG_00641	60S ribosomal protein L17	ko03010//Ribosome	GO:0005576//extracellular region;GO:0015934//large ribosomal subunit	GO:0003735//structural constituent of ribosome	GO:0006412//translation	gi|115492199|ref|XP_001210727.1|/1.34225e-104/60S ribosomal protein L17 [Aspergillus terreus NIH2624]
4322722	1149	573.81	965.66	1.378058625	5.85E-15	8.34E-14	Up	ATEG_07798	hypothetical protein	-	-	GO:0016740//transferase activity	-	gi|115401662|ref|XP_001216419.1|/0/conserved hypothetical protein [Aspergillus terreus NIH2624]

4318665	2315	264.53	447.34	1.377261603	7.56E-10	7.36E-09	Up	ATEG_04111	similar to kinesin	-	GO:0005874//microtubule	GO:0003777//microtubule motor activity;GO:0005524//ATP binding;GO:0008017//microtubule binding	GO:0007018//microtubule-based movement	gi|115391569|ref|XP_001213289.1|/0/hypothetical protein ATEG_04111 [Aspergillus terreus NIH2624]
4355120	654	3952.88	6652.89	1.377046435	5.37E-30	1.82E-28	Up	ATEG_00368	60S ribosomal protein L1	ko03010//Ribosome	GO:0015934//large ribosomal subunit	GO:0003723//RNA binding;GO:0003735//structural constituent of ribosome	GO:0006412//translation	gi|115491653|ref|XP_001210454.1|/4.25035e-153/60S ribosomal protein L1 [Aspergillus terreus NIH2624]
4353769	1638	223.57	380.27	1.37643743	9.72E-09	8.49E-08	Up	ATEG_09238	similar to multidrug resistant protein	-	GO:0016021//integral component of membrane	-	GO:0055085//transmembrane transport	gi|115437634|ref|XP_001217860.1|/0/hypothetical protein ATEG_09238 [Aspergillus terreus NIH2624]
4318610	2352	1147.29	1921.63	1.375150251	1.89E-18	3.43E-17	Up	ATEG_03836	hypothetical protein	-	GO:0016021//integral component of membrane	GO:0008233//peptidase activity	GO:0006508//proteolysis	gi|115391019|ref|XP_001213014.1|/0/conserved hypothetical protein [Aspergillus terreus NIH2624]
4354245	600	166.42	281.81	1.37422003	7.18E-07	5.06E-06	Up	ATEG_09789	hypothetical protein	-	-	-	-	gi|115443208|ref|XP_001218411.1|/3.85309e-147/predicted protein [Aspergillus terreus NIH2624]
4354333	1536	831.19	1391.91	1.373203603	1.34E-16	2.17E-15	Up	ATEG_09547	similar to potential cyclopropane-fatty-acyl-phospholipid synthase	-	GO:0016021//integral component of membrane	GO:0008825//cyclopropane-fatty-acyl-phospholipid synthase activity	GO:0032259//methylation	gi|115442724|ref|XP_001218169.1|/0/hypothetical protein ATEG_09547 [Aspergillus terreus NIH2624]

4354131	3699	317.78	533.8	1.37088907	1.51E-10	1.59E-09	Up	ATEG_09535	similar to histidine kinase J7	ko01100//Metabolic pathways;ko00520//Amino sugar and nucleotide sugar metabolism	-	-	-	gi|115442700|ref|XP_001218157.1|/0/hypothetical protein ATEG_09535 [Aspergillus terreus NIH2624]
4321936	1500	125.6	216.07	1.370124781	5.47E-05	0.00028745	Up	ATEG_06506	hypothetical protein	-	GO:0016021//integral component of membrane	GO:0015171//amino acid transmembrane transporter activity	GO:0003333//amino acid transmembrane transport	gi|115400191|ref|XP_001215684.1|/0/predicted protein [Aspergillus terreus NIH2624]
4323374	1326	212.99	357.79	1.369648151	7.26E-07	5.12E-06	Up	ATEG_08656	hypothetical protein	ko04111//Cell cycle - yeast	-	GO:0016810//hydrolase activity, acting on carbon-nitrogen (but not peptide) bonds	-	gi|115402331|ref|XP_001217242.1|/0/conserved hypothetical protein [Aspergillus terreus NIH2624]
4317775	468	3554.93	5967.66	1.368976314	2.86E-21	6.34E-20	Up	ATEG_03402	40S ribosomal protein S18	ko03010//Ribosome	GO:0005576//extracellular region;GO:0005840//ribosome	GO:0003723//RNA binding;GO:0003735//structural constituent of ribosome	GO:0006412//translation	gi|115390150|ref|XP_001212580.1|/2.99952e-109/40S ribosomal protein S18 [Aspergillus terreus NIH2624]
4321674	2091	405.16	680.18	1.36806231	5.92E-13	7.58E-12	Up	ATEG_06184	hypothetical protein	-	-	-	-	gi|115399546|ref|XP_001215362.1|/0/conserved hypothetical protein [Aspergillus terreus NIH2624]
4317972	7506	1155.54	1937.59	1.367938038	1.23E-20	2.61E-19	Up	ATEG_03522	hypothetical protein	-	GO:0016021//integral component of membrane	-	GO:0055085//transmembrane transport	gi|115390390|ref|XP_001212700.1|/0/predicted protein [Aspergillus terreus NIH2624]
4318358	870	61.05	106.92	1.366637942	0.00134631	0.00534821	Up	ATEG_03645	hypothetical protein	-	-	-	-	gi|115390637|ref|XP_001212823.1|/0/conserved hypothetical protein [Aspergillus terreus NIH2624]

4353890	1107	263.98	442.44	1.3661091	3.01E-09	2.78E-08	Up	ATEG_09440	hypothetical protein	ko03018//RNA degradation	GO:0005737//cytoplasm	GO:0008757//S-adenosylmethionine-dependent methyltransferase activity	GO:0032259//methylation	gi|115438422|ref|XP_001218062.1|/0/conserved hypothetical protein [Aspergillus terreus NIH2624]
4353175	987	141.24	238.58	1.365677806	2.22E-06	1.47E-05	Up	ATEG_08151	hypothetical protein	-	-	GO:0016614//oxidoreductase activity, acting on CH-OH group of donors;GO:0050660//flavin adenine dinucleotide binding	GO:0055114//oxidation-reduction process	gi|115433270|ref|XP_001216772.1|/0/conserved hypothetical protein [Aspergillus terreus NIH2624]
4321883	955	155.78	261.68	1.363916801	1.36E-05	7.94E-05	Up	ATEG_06672	similar to alcohol dehydrogenase	-	-	GO:0016491//oxidoreductase activity	GO:0008152//metabolic process	gi|115400523|ref|XP_001215850.1|/5.92738e-140/hypothetical protein ATEG_06672 [Aspergillus terreus NIH2624]
4321834	1719	847.29	1430.16	1.361182473	7.24E-12	8.57E-11	Up	ATEG_05900	electron transfer protein 1, mitochondrial precursor	ko01100//Metabolic pathways;ko01110//Biosynthesis of secondary metabolites;ko00190//Oxidative phosphorylation;ko00860//Porphyrin and chlorophyll metabolism	GO:0016021//integral component of membrane	GO:0016627//oxidoreductase activity, acting on the CH-CH group of donors	GO:0006784//heme a biosynthetic process;GO:0055114//oxidation-reduction process	gi|115398978|ref|XP_001215078.1|/0/electron transfer protein 1, mitochondrial precursor [Aspergillus terreus NIH2624]
4321455	1311	155.25	261.4	1.360285534	7.08E-07	5.00E-06	Up	ATEG_05746	hypothetical protein	ko01100//Metabolic pathways;ko00500//Starch and sucrose metabolism	GO:0016021//integral component of membrane	GO:0015098//molybdate ion transmembrane transporter activity	GO:0015689//molybdate ion transport	gi|115398670|ref|XP_001214924.1|/0/conserved hypothetical protein [Aspergillus terreus NIH2624]

4323228	2004	66.7	114.87	1.359743135	0.00053917	0.00235085	Up	ATEG_08927	hypothetical protein	-	GO:0005634//nucleus	GO:0000981//RNA polymerase II transcription factor activity, sequence-specific DNA binding;GO:0003677//DNA binding;GO:0008270//zinc ion binding	GO:0006357//regulation of transcription from RNA polymerase II promoter	gi|115402873|ref|XP_001217513.1|/0/predicted protein [Aspergillus terreus NIH2624]
4321171	3201	70.61	120.23	1.356105294	0.00045631	0.00202341	Up	ATEG_05080	voltage-gated potassium channel beta-1 subunit	ko01100//Metabolic pathways;ko00051//Fructose and mannose metabolism;ko00650//Butanoate metabolism;ko00591//Linoleic acid metabolism	-	-	-	gi|115397333|ref|XP_001214258.1|/0/voltage-gated potassium channel beta-1 subunit [Aspergillus terreus NIH2624]

4320098	1473	316.66	524.66	1.35587479	5.40E-08	4.40E-07	Up	ATEG_04853	hypothetical protein	ko01100//Metabolic pathways;ko01110//Biosynthesis of secondary metabolites;ko01130//Biosynthesis of antibiotics;ko00380//Tryptophan metabolism;ko00010//Glycolysis / Gluconeogenesis;ko00620//Pyruvate metabolism;ko00561//Glycerolipid metabolism;ko00071//Fatty acid degradation;ko00310//Lysine degradation;ko00280//Valine, leucine and isoleucine degradation;ko00330//Arginine and proline metabolism;ko00040//Pentose and glucuronate interconversions;ko00410//beta-Alanine metabolism;ko00340//Histidine metabolism;ko00053//Ascorbate and	-	GO:0016620//oxidoreductase activity, acting on the aldehyde or oxo group of donors, NAD or NADP as acceptor	GO:0055114//oxidation-reduction process	gi|115396784|ref|XP_001214031.1|/0/conserved hypothetical protein [Aspergillus terreus NIH2624]
4320828	1560	33.63	59.35	1.354216192	0.00834115	0.02653949	Up	ATEG_05018	hypothetical protein	-	GO:0005634//nucleus	GO:0003677//DNA binding;GO:0008270//zinc ion binding	GO:0006351//transcription, DNA-templated	gi|115397209|ref|XP_001214196.1|/0/conserved hypothetical protein [Aspergillus terreus NIH2624]

4317717	1326	8529.71	14103.41	1.352806729	3.03E-32	1.11E-30	Up	ATEG_03330	60S ribosomal protein L8	ko03010//Ribosome	GO:0005840//ribosome	-	GO:0042254//ribosome biogenesis	gi|115390006|ref|XP_001212508.1|/0/60S ribosomal protein L8 [Aspergillus terreus NIH2624]
4355225	1952	274.52	454.66	1.35174613	5.03E-06	3.14E-05	Up	ATEG_00471	similar to choline sulfatase	-	-	GO:0008484//sulfuric ester hydrolase activity	GO:0008152//metabolic process	gi|115491859|ref|XP_001210557.1|/0/hypothetical protein ATEG_00471 [Aspergillus terreus NIH2624]
4318045	3229	7859.01	12942.76	1.35016999	3.13E-31	1.11E-29	Up	ATEG_03027	serine palmitoyltransferase 2	ko01100//Metabolic pathways;ko00600//Sphingolipid metabolism	GO:0015934//large ribosomal subunit;GO:0016021//integral component of membrane	GO:0003735//structural constituent of ribosome;GO:0016740//transferase activity;GO:0030170//pyridoxal phosphate binding	GO:0006412//translation	gi|115389400|ref|XP_001212205.1|/0/serine palmitoyltransferase 2 [Aspergillus terreus NIH2624]
4316641	10351	173.15	287.87	1.350126662	1.30E-06	8.83E-06	Up	ATEG_02002	hypothetical protein	-	GO:0000781//chromosome, telomeric region;GO:0005634//nucleus	GO:0004674//protein serine/threonine kinase activity;GO:0005524//ATP binding	GO:0000077//DNA damage checkpoint;GO:0000723//telomere maintenance;GO:0006281//DNA repair;GO:0010212//response to ionizing radiation;GO:0016572//histone phosphorylation;GO:0090399//replicative senescence	gi|115387349|ref|XP_001211180.1|/0/conserved hypothetical protein [Aspergillus terreus NIH2624]
4317744	1731	203.5	341.41	1.349548101	7.61E-07	5.34E-06	Up	ATEG_03390	similar to sulfatase family protein	-	-	GO:0047753	GO:0008152//metabolic process	gi|115390126|ref|XP_001212568.1|/0/hypothetical protein ATEG_03390 [Aspergillus terreus NIH2624]

4319353	2804	850.26	1399.16	1.347377442	2.17E-16	3.48E-15	Up	ATEG_07061	hypothetical protein	ko01100//Metabolic pathways;ko01110//Biosynthesis of secondary metabolites	-	GO:0005488	-	gi|115386412|ref|XP_001209747.1|/7.75321e-143/conserved hypothetical protein [Aspergillus terreus NIH2624]
4322315	888	53.23	90.91	1.346355685	0.00240407	0.0089763	Up	ATEG_06625	hypothetical protein	ko00310//Lysine degradation	-	GO:0016491//oxidoreductase activity	GO:0055114//oxidation-reduction process	gi|115400429|ref|XP_001215803.1|/0/conserved hypothetical protein [Aspergillus terreus NIH2624]
4316036	4844	344.11	568.38	1.344179597	3.23E-11	3.60E-10	Up	ATEG_01411	hypothetical protein	-	GO:0005634//nucleus;GO:0005829//cytosol	-	-	gi|115384458|ref|XP_001208776.1|/0/predicted protein [Aspergillus terreus NIH2624]
4354256	1398	31.37	56.61	1.344078009	0.01583837	0.04633971	Up	ATEG_09799	hypothetical protein	ko01100//Metabolic pathways;ko00520//Amino sugar and nucleotide sugar metabolism	GO:0016021//integral component of membrane	-	GO:0055085//transmembrane transport	gi|115443228|ref|XP_001218421.1|/0/predicted protein [Aspergillus terreus NIH2624]
4355506	2173	536.9	880.61	1.340376152	5.38E-14	7.30E-13	Up	ATEG_00751	hypothetical protein	-	GO:0016021//integral component of membrane	GO:0008496//mannan endo-1,6-alpha-mannosidase activity	GO:0016052//carbohydrate catabolic process	gi|115492419|ref|XP_001210837.1|/0/conserved hypothetical protein [Aspergillus terreus NIH2624]
4317340	4970	3314.13	5416.32	1.340288158	4.97E-27	1.49E-25	Up	ATEG_02778	hypothetical protein	-	GO:0005634//nucleus;GO:0005794//Golgi apparatus;GO:0030123//AP-3 adaptor complex	GO:0008565//protein transporter activity	GO:0006623//protein targeting to vacuole;GO:0006896//Golgi to vacuole transport	gi|115388902|ref|XP_001211956.1|/0/conserved hypothetical protein [Aspergillus terreus NIH2624]
4317428	3720	6269.54	10233.81	1.33728696	1.99E-31	7.10E-30	Up	ATEG_03234	similar to potassium transport protein	-	GO:0016021//integral component of membrane	GO:0015079//potassium ion transmembrane transporter activity	GO:0071805//potassium ion transmembrane transport	gi|115389814|ref|XP_001212412.1|/0/hypothetical protein ATEG_03234 [Aspergillus terreus NIH2624]

4319639	6285	7730.03	12600.64	1.336413183	8.62E-31	2.98E-29	Up	ATEG_10174	glutamate synthase precursor	ko01100//Metabolic pathways;ko01110//Biosynthesis of secondary metabolites;ko01130//Biosynthesis of antibiotics;ko01230//Biosynthesis of amino acids;ko00250//Alanine, aspartate and glutamate metabolism;ko00910//Nitrogen metabolism	GO:0005739//mitochondrion;GO:0005829//cytosol	GO:0005506//iron ion binding;GO:0010181//FMN binding;GO:0016040//glutamate synthase (NADH) activity;GO:0050660//flavin adenine dinucleotide binding;GO:0051536//iron-sulfur cluster binding	GO:0006537//glutamate biosynthetic process;GO:0055114//oxidation-reduction process	gi|115385859|ref|XP_001209476.1|/0/glutamate synthase precursor [Aspergillus terreus NIH2624]
4320257	1065	242.7	400.52	1.334810816	2.03E-08	1.72E-07	Up	ATEG_04912	zinc-regulated transporter 1	-	GO:0005783//endoplasmic reticulum;GO:0005887//integral component of plasma membrane	GO:0000006//high-affinity zinc uptake transmembrane transporter activity	GO:0006830//high-affinity zinc II ion transport	gi|115396902|ref|XP_001214090.1|/0/zinc-regulated transporter 1 [Aspergillus terreus NIH2624]
4318098	738	954.77	1555.67	1.333109515	2.78E-16	4.41E-15	Up	ATEG_03311	hypothetical protein	ko01100//Metabolic pathways;ko00500//Starch and sucrose metabolism	GO:0005576//extracellular region	GO:0016787//hydrolase activity	-	gi|115389968|ref|XP_001212489.1|/0/conserved hypothetical protein [Aspergillus terreus NIH2624]
4316076	1644	248.81	406.06	1.3302093	1.34E-07	1.04E-06	Up	ATEG_01841	hypothetical protein	ko00910//Nitrogen metabolism	GO:0005829//cytosol	GO:0016491//oxidoreductase activity;GO:0046872//metal ion binding;GO:0050660//flavin adenine dinucleotide binding;GO:0051537//2 iron, 2 sulfur cluster binding	GO:0045454//cell redox homeostasis;GO:0055114//oxidation-reduction process	gi|115385318|ref|XP_001209206.1|/0/conserved hypothetical protein [Aspergillus terreus NIH2624]
4320201	906	327.89	532.19	1.326065958	4.43E-08	3.65E-07	Up	ATEG_04520	hypothetical protein	-	GO:0016021//integral component of membrane	-	-	gi|115396118|ref|XP_001213698.1|/0/conserved hypothetical protein [Aspergillus terreus NIH2624]

4317103	2118	2352.86	3828.81	1.324753423	8.12E-22	1.84E-20	Up	ATEG_02178	hypothetical protein	ko01100//Metabolic pathways;ko00230//Purine metabolism	-	GO:0016620//oxidoreductase activity, acting on the aldehyde or oxo group of donors, NAD or NADP as acceptor	GO:0055114//oxidation-reduction process	gi|115387701|ref|XP_001211356.1|/0/conserved hypothetical protein [Aspergillus terreus NIH2624]
4319384	832	5361.74	8683.11	1.324174735	9.63E-27	2.84E-25	Up	ATEG_07079	60S ribosomal protein L14	ko03010//Ribosome	GO:0005634//nucleus;GO:0005829//cytosol;GO:0005840//ribosome	GO:0003735//structural constituent of ribosome	GO:0006412//translation	gi|115386448|ref|XP_001209765.1|/4.84943e-103/60S ribosomal protein L14 [Aspergillus terreus NIH2624]
4323078	969	373.8	606.69	1.319565403	1.95E-11	2.22E-10	Up	ATEG_08962	hypothetical protein	ko01100//Metabolic pathways;ko01110//Biosynthesis of secondary metabolites;ko01130//Biosynthesis of antibiotics;ko00520//Amino sugar and nucleotide sugar metabolism;ko00010//Glycolysis / Gluconeogenesis;ko00052//Galactose metabolism	GO:0005576//extracellular region	GO:0016853//isomerase activity;GO:0030246//carbohydrate binding	GO:0005975//carbohydrate metabolic process	gi|115402943|ref|XP_001217548.1|/0/conserved hypothetical protein [Aspergillus terreus NIH2624]
4320154	1128	275.71	446.77	1.318974978	7.69E-09	6.80E-08	Up	ATEG_04535	hypothetical protein	-	GO:0016021//integral component of membrane	-	-	gi|115396148|ref|XP_001213713.1|/0/predicted protein [Aspergillus terreus NIH2624]
4353228	4502	255.47	414.82	1.318489193	1.08E-07	8.48E-07	Up	ATEG_08193	similar to multidrug resistance protein	-	GO:0016021//integral component of membrane	GO:0005524//ATP binding;GO:0042626//ATPase activity, coupled to transmembrane movement of substances	GO:0055085//transmembrane transport	gi|115433354|ref|XP_001216814.1|/0/hypothetical protein ATEG_08193 [Aspergillus terreus NIH2624]

4320868	2018	72.85	120.55	1.318093218	0.00074864	0.00314575	Up	ATEG_05528	similar to cytochrome b2, mitochondrial precursor	ko01100//Metabolic pathways;ko00620//Pyruvate metabolism	-	GO:0010181//FMN binding;GO:0016491//oxidoreductase activity;GO:0020037//heme binding;GO:0046872//metal ion binding	GO:0055114//oxidation-reduction process	gi|115398229|ref|XP_001214706.1|/0/hypothetical protein ATEG_05528 [Aspergillus terreus NIH2624]
4320915	5345	1022.13	1641.27	1.315666882	1.99E-15	2.94E-14	Up	ATEG_05248	hypothetical protein	-	GO:0005634//nucleus	GO:0003677//DNA binding;GO:0046872//metal ion binding	GO:0006355//regulation of transcription, DNA-templated	gi|115397669|ref|XP_001214426.1|/0/conserved hypothetical protein [Aspergillus terreus NIH2624]
4322624	1871	311.05	502.46	1.314324662	9.54E-10	9.20E-09	Up	ATEG_07912	hypothetical protein	-	GO:0005634//nucleus	GO:0003677//DNA binding;GO:0008270//zinc ion binding	GO:0006351//transcription, DNA-templated	gi|115401890|ref|XP_001216533.1|/0/predicted protein [Aspergillus terreus NIH2624]
4318015	1803	4403.47	7058.61	1.31385188	4.16E-19	7.95E-18	Up	ATEG_03352	similar to H+/nucleoside cotransporter	-	GO:0016021//integral component of membrane	GO:0005337//nucleoside transmembrane transporter activity	GO:1901642//nucleoside transmembrane transport	gi|115390050|ref|XP_001212530.1|/0/hypothetical protein ATEG_03352 [Aspergillus terreus NIH2624]
4321727	2973	190.6	308.68	1.311754983	3.58E-06	2.30E-05	Up	ATEG_05865	hypothetical protein	-	GO:0005634//nucleus	GO:0000981//RNA polymerase II transcription factor activity, sequence-specific DNA binding;GO:0003677//DNA binding;GO:0008270//zinc ion binding	GO:0006357//regulation of transcription from RNA polymerase II promoter	gi|115398908|ref|XP_001215043.1|/0/conserved hypothetical protein [Aspergillus terreus NIH2624]

4319980	531	594.77	966.97	1.310899624	5.93E-10	5.81E-09	Up	ATEG_04483	hypothetical protein	ko01100//Metabolic pathways;ko00520//Amino sugar and nucleotide sugar metabolism	GO:0009277//fungal-type cell wall	-	-	gi|115396044|ref|XP_001213661.1|/5.39659e-129/conserved hypothetical protein [Aspergillus terreus NIH2624]
4320593	1260	1222.79	1958.41	1.309870739	2.05E-19	4.05E-18	Up	ATEG_05556	hypothetical protein	ko01100//Metabolic pathways;ko00790//Folate biosynthesis	GO:0005829//cytosol	GO:0004326//tetrahydrofolylpolyglutamate synthase activity;GO:0005524//ATP binding;GO:0008841//dihydrofolate synthase activity	GO:0006730//one-carbon metabolic process;GO:0006761//dihydrofolate biosynthetic process;GO:0046901//tetrahydrofolylpolyglutamate biosynthetic process	gi|115398285|ref|XP_001214734.1|/0/conserved hypothetical protein [Aspergillus terreus NIH2624]
4354729	2701	10417.46	16656.74	1.308277707	1.50E-19	2.99E-18	Up	ATEG_10286	hypothetical protein	-	GO:0016021//integral component of membrane	GO:0015171//amino acid transmembrane transporter activity	GO:0003333//amino acid transmembrane transport	gi|115449543|ref|XP_001218634.1|/0/conserved hypothetical protein [Aspergillus terreus NIH2624]
4319203	555	5160.78	8277.17	1.307157781	2.33E-22	5.49E-21	Up	ATEG_06847	60S ribosomal protein L18	ko03010//Ribosome	GO:0022625//cytosolic large ribosomal subunit	GO:0003723//RNA binding;GO:0003735//structural constituent of ribosome	GO:0006412//translation	gi|115385984|ref|XP_001209532.1|/8.15885e-128/60S ribosomal protein L18 [Aspergillus terreus NIH2624]
4316623	696	608.52	970.38	1.305594351	1.17E-09	1.12E-08	Up	ATEG_02324	similar to uracil phosphoribosyltransferase	ko01100//Metabolic pathways;ko00240//Pyrimidine metabolism	GO:0005829//cytosol	GO:0004845//uracil phosphoribosyltransferase activity;GO:0004849//uridine kinase activity	GO:0006206//pyrimidine nucleobase metabolic process;GO:0006222//UMP biosynthetic process;GO:0043097//pyrimidine nucleoside salvage	gi|115387993|ref|XP_001211502.1|/2.76639e-165/hypothetical protein ATEG_02324 [Aspergillus terreus NIH2624]
4318040	1062	124.94	202.87	1.305485554	4.12E-05	0.00022341	Up	ATEG_03212	cyanide hydratase	ko00460//Cyanoamino acid metabolism	-	GO:0000257//nitrilase activity;GO:0030196	GO:0019500//cyanide catabolic process	gi|115389770|ref|XP_001212390.1|/0/cyanide hydratase [Aspergillus terreus NIH2624]

4353081	915	1881.65	3016.72	1.304442289	2.97E-21	6.56E-20	Up	ATEG_08317	pyridoxine biosynthesis protein PDX1	ko00750//Vitamin B6 metabolism	GO:0005576//extracellular region	GO:0036381//pyridoxal 5'-phosphate synthase (glutamine hydrolysing) activity	GO:0008615//pyridoxine biosynthetic process;GO:0042823//pyridoxal phosphate biosynthetic process	gi|115433602|ref|XP_001216938.1|/0/pyridoxine biosynthesis protein PDX1 [Aspergillus terreus NIH2624]
4355187	2945	1905.2	3031.92	1.300813377	1.10E-21	2.48E-20	Up	ATEG_00433	hypothetical protein	-	GO:0005576//extracellular region	-	-	gi|115491783|ref|XP_001210519.1|/0/conserved hypothetical protein [Aspergillus terreus NIH2624]
4315791	3186	155.22	249.51	1.295383519	3.36E-06	2.18E-05	Up	ATEG_01168	hypothetical protein	-	-	-	-	gi|115383972|ref|XP_001208533.1|/0/conserved hypothetical protein [Aspergillus terreus NIH2624]
4322566	810	59.42	98.1	1.295336513	0.00218989	0.00826872	Up	ATEG_07470	hypothetical protein	-	-	GO:0016491//oxidoreductase activity	GO:0055114//oxidation-reduction process	gi|115401006|ref|XP_001216091.1|/0/conserved hypothetical protein [Aspergillus terreus NIH2624]
4316372	1054	57.18	94.52	1.294954594	0.00245972	0.00915856	Up	ATEG_01741	hypothetical protein	-	-	-	-	gi|115385118|ref|XP_001209106.1|/1.25602e-86/conserved hypothetical protein [Aspergillus terreus NIH2624]
4323459	966	737.36	1173.76	1.293579495	1.82E-14	2.53E-13	Up	ATEG_08963	nicotinate-nucleotide pyrophosphorylase (Quinolinate phosphoribosyltransferase [decarboxylating])	ko01100//Metabolic pathways;ko00760//Nicotinate and nicotinamide metabolism	-	GO:0004514//nicotinate-nucleotide diphosphorylase (carboxylating) activity	GO:0009435//NAD biosynthetic process	gi|115402945|ref|XP_001217549.1|/0/nicotinate-nucleotide pyrophosphorylase (Quinolinate phosphoribosyltransferase [decarboxylating]) [Aspergillus terreus NIH2624]

4353192	1185	61.08	100.31	1.292883806	0.00150156	0.00589996	Up	ATEG_08182	hypothetical protein	-	GO:0016021//integral component of membrane	GO:0015171//amino acid transmembrane transporter activity	GO:0003333//amino acid transmembrane transport	gi|115433332|ref|XP_001216803.1|/0/conserved hypothetical protein [Aspergillus terreus NIH2624]
4355618	330	223.69	365.08	1.292134686	2.41E-05	0.00013577	Up	ATEG_00857	hypothetical protein	-	-	-	-	gi|115492631|ref|XP_001210943.1|/3.08954e-80/predicted protein [Aspergillus terreus NIH2624]
4323031	2248	75.05	123.38	1.291556068	0.00134532	0.0053465	Up	ATEG_07527	hypothetical protein	-	GO:0005634//nucleus	GO:0003677//DNA binding;GO:0008270//zinc ion binding	GO:0006351//transcription, DNA-templated	gi|115401120|ref|XP_001216148.1|/0/predicted protein [Aspergillus terreus NIH2624]
4353511	2436	729.08	1157.05	1.289559579	1.20E-15	1.80E-14	Up	ATEG_08291	similar to cysteinyl-tRNA-synthetase	ko00970//Aminoacyl-tRNA biosynthesis	-	GO:0004817//cysteine-tRNA ligase activity;GO:0005524//ATP binding	GO:0006423//cysteinyl-tRNA aminoacylation	gi|115433550|ref|XP_001216912.1|/0/hypothetical protein ATEG_08291 [Aspergillus terreus NIH2624]
4318666	2627	64.4	109.4	1.288635307	0.00739668	0.02392383	Up	ATEG_04112	hypothetical protein	-	-	-	-	gi|115391571|ref|XP_001213290.1|/0/predicted protein [Aspergillus terreus NIH2624]
4353267	1056	117.14	187.88	1.285980016	4.51E-05	0.00024263	Up	ATEG_08284	hypothetical protein	-	-	GO:0008270//zinc ion binding;GO:0016491//oxidoreductase activity	GO:0055114//oxidation-reduction process	gi|115433536|ref|XP_001216905.1|/0/conserved hypothetical protein [Aspergillus terreus NIH2624]
4317339	399	1495.99	2362.74	1.283900303	5.94E-18	1.04E-16	Up	ATEG_02777	60S ribosomal protein L26	ko03010//Ribosome	GO:0015934//large ribosomal subunit	GO:0003735//structural constituent of ribosome	GO:0006412//translation	gi|115388900|ref|XP_001211955.1|/3.31247e-86/60S ribosomal protein L26 [Aspergillus terreus NIH2624]

4320277	1822	6857.34	10784.18	1.28280075	1.60E-29	5.28E-28	Up	ATEG_04616	hypothetical protein	-	GO:0005622//intracellular	-	-	gi|115396310|ref|XP_001213794.1|/0/predicted protein [Aspergillus terreus NIH2624]
4317711	336	77.36	125.73	1.281582752	0.00080984	0.0033712	Up	ATEG_03324	hypothetical protein	-	-	GO:0003676//nucleic acid binding;GO:0008270//zinc ion binding	-	gi|115389994|ref|XP_001212502.1|/9.90394e-78/conserved hypothetical protein [Aspergillus terreus NIH2624]
4321365	3825	99.23	158.75	1.279808809	0.00088525	0.00365273	Up	ATEG_05997	hypothetical protein	-	GO:0016021//integral component of membrane	-	-	gi|115399172|ref|XP_001215175.1|/0/predicted protein [Aspergillus terreus NIH2624]
4318479	1318	471.73	747.14	1.279587284	2.75E-10	2.80E-09	Up	ATEG_03945	hypothetical protein	-	GO:0005829//cytosol	GO:0001671//ATPase activator activity;GO:0051087//chaperone binding	GO:0006457//protein folding;GO:0006950//response to stress;GO:0032781//positive regulation of ATPase activity	gi|115391237|ref|XP_001213123.1|/0/conserved hypothetical protein [Aspergillus terreus NIH2624]
4319273	1116	49.84	82.08	1.27926002	0.0074381	0.02402469	Up	ATEG_06963	hypothetical protein	-	-	GO:0016491//oxidoreductase activity	GO:0055114//oxidation-reduction process	gi|115386216|ref|XP_001209649.1|/0/conserved hypothetical protein [Aspergillus terreus NIH2624]
4355612	546	47.64	78.26	1.27842589	0.00508544	0.01731375	Up	ATEG_00851	similar to ARL2 G-protein	-	GO:0005622//intracellular	GO:0005525//GTP binding	GO:0007264//small GTPase mediated signal transduction	gi|115492619|ref|XP_001210937.1|/4.76634e-130/hypothetical protein ATEG_00851 [Aspergillus terreus NIH2624]

4353702	1293	113.77	180.88	1.278109091	0.00018011	0.00086489	Up	ATEG_09079	similar to extracellular phospholipase C	ko01100//Metabolic pathways;ko01110//Biosynthesis of secondary metabolites;ko00564//Glycerophospholipid metabolism;ko00562//Inositol phosphate metabolism;ko00565//Ether lipid metabolism	GO:0016021//integral component of membrane	GO:0016788//hydrolase activity, acting on ester bonds	GO:0008152//metabolic process	gi|115437004|ref|XP_001217701.1|/0/hypothetical protein ATEG_09079 [Aspergillus terreus NIH2624]
4320373	2366	220.78	347.88	1.277810644	1.11E-06	7.60E-06	Up	ATEG_04727	hypothetical protein	ko04113//Meiosis - yeast	GO:0016021//integral component of membrane	GO:0022891//substrate-specific transmembrane transporter activity	GO:0055085//transmembrane transport	gi|115396532|ref|XP_001213905.1|/0/conserved hypothetical protein [Aspergillus terreus NIH2624]
4319900	1179	3418.49	5327.93	1.276448541	3.54E-17	5.91E-16	Up	ATEG_04501	hypothetical protein	ko01100//Metabolic pathways;ko01110//Biosynthesis of secondary metabolites;ko01130//Biosynthesis of antibiotics;ko00010//Glycolysis / Gluconeogenesis	-	GO:0046872//metal ion binding	-	gi|115396080|ref|XP_001213679.1|/0/conserved hypothetical protein [Aspergillus terreus NIH2624]
4320447	420	1204.2	1888.38	1.276052044	6.96E-19	1.29E-17	Up	ATEG_04465	40S ribosomal protein S17	ko03010//Ribosome	GO:0005840//ribosome	GO:0003735//structural constituent of ribosome	GO:0006412//translation	gi|115396008|ref|XP_001213643.1|/1.60809e-94/40S ribosomal protein S17 [Aspergillus terreus NIH2624]
4317432	1852	103.67	166.28	1.275742557	0.00010469	0.0005222	Up	ATEG_03238	similar to alcohol dehydrogenase	ko00640//Propanoate metabolism	-	GO:0008270//zinc ion binding;GO:0016491//oxidoreductase activity	GO:0055114//oxidation-reduction process	gi|115389822|ref|XP_001212416.1|/0/hypothetical protein ATEG_03238 [Aspergillus terreus NIH2624]

4321585	909	725.99	1135.03	1.27570326	2.07E-07	1.58E-06	Up	ATEG_05673	similar to polysaccharide deacetylase family protein	ko01100//Metabolic pathways;ko00520//Amino sugar and nucleotide sugar metabolism	-	GO:0016810//hydrolase activity, acting on carbon-nitrogen (but not peptide) bonds	GO:0005975//carbohydrate metabolic process	gi|115398524|ref|XP_001214851.1|/0/hypothetical protein ATEG_05673 [Aspergillus terreus NIH2624]
4320778	1557	113.07	184.1	1.272372815	0.00394634	0.01391003	Up	ATEG_05398	similar to folylpolyglutamate synthetase	ko01100//Metabolic pathways;ko00790//Folate biosynthesis	-	GO:0004326//tetrahydrofolylpolyglutamate synthase activity;GO:0005524//ATP binding	GO:0006730//one-carbon metabolic process;GO:0046901//tetrahydrofolylpolyglutamate biosynthetic process	gi|115397969|ref|XP_001214576.1|/0/hypothetical protein ATEG_05398 [Aspergillus terreus NIH2624]
4316487	1041	67.2	109.38	1.270187663	0.00316289	0.01141439	Up	ATEG_02461	hypothetical protein	ko03013//RNA transport	GO:0016021//integral component of membrane	GO:0004497//monooxygenase activity;GO:0005506//iron ion binding	GO:0006633//fatty acid biosynthetic process;GO:0055114//oxidation-reduction process	gi|115388267|ref|XP_001211639.1|/0/conserved hypothetical protein [Aspergillus terreus NIH2624]
4323260	477	2866.5	4474.71	1.268574368	3.81E-24	9.70E-23	Up	ATEG_08726	60S ribosomal protein L21	ko03010//Ribosome	GO:0005840//ribosome	GO:0003735//structural constituent of ribosome	GO:0006412//translation	gi|115402471|ref|XP_001217312.1|/4.75289e-112/60S ribosomal protein L21 [Aspergillus terreus NIH2624]
4323482	1803	527.79	824.26	1.268490086	3.34E-11	3.71E-10	Up	ATEG_08987	cystathionine gamma-synthase	ko01100//Metabolic pathways;ko01110//Biosynthesis of secondary metabolites;ko01130//Biosynthesis of antibiotics;ko01230//Biosynthesis of amino acids;ko00270//Cysteine and methionine metabolism;ko00920//Sulfur metabolism;ko00450//Selenocompound metabolism	GO:0005829//cytosol	GO:0003962//cystathionine gamma-synthase activity;GO:0030170//pyridoxal phosphate binding	-	gi|115402993|ref|XP_001217573.1|/0/cystathionine gamma-synthase [Aspergillus terreus NIH2624]

4318534	1991	103.71	165.05	1.267288741	0.00024373	0.00114288	Up	ATEG_04221	hypothetical protein	-	GO:0005634//nucleus	GO:0003677//DNA binding;GO:0008270//zinc ion binding	GO:0006351//transcription, DNA-templated	gi|115391789|ref|XP_001213399.1|/0/predicted protein [Aspergillus terreus NIH2624]
4319237	2364	243.75	383.45	1.26600724	7.56E-08	6.06E-07	Up	ATEG_07102	hypothetical protein	ko01100//Metabolic pathways;ko00780//Biotin metabolism	-	GO:0000287//magnesium ion binding;GO:0004141//dethiobiotin synthase activity;GO:0005524//ATP binding;GO:0008483//transaminase activity;GO:0030170//pyridoxal phosphate binding	GO:0009102//biotin biosynthetic process	gi|115386494|ref|XP_001209788.1|/0/conserved hypothetical protein [Aspergillus terreus NIH2624]
4353253	1125	59.41	96.7	1.263041981	0.00312536	0.01131377	Up	ATEG_08003	hypothetical protein	-	GO:0016021//integral component of membrane	-	-	gi|115432974|ref|XP_001216624.1|/0/conserved hypothetical protein [Aspergillus terreus NIH2624]
4319342	1561	48.23	78.51	1.262791409	0.00962242	0.02987663	Up	ATEG_06941	hypothetical protein	ko01100//Metabolic pathways;ko00620//Pyruvate metabolism	-	GO:0010181//FMN binding;GO:0016491//oxidoreductase activity;GO:0020037//heme binding;GO:0046872//metal ion binding	GO:0055114//oxidation-reduction process	gi|115386172|ref|XP_001209627.1|/0/conserved hypothetical protein [Aspergillus terreus NIH2624]
4322188	10806	8614.41	13308.55	1.261585714	4.28E-24	1.08E-22	Up	ATEG_06545	hypothetical protein	-	-	-	-	gi|115400269|ref|XP_001215723.1|/0/conserved hypothetical protein [Aspergillus terreus NIH2624]

4353087	1821	99.7	161.09	1.260868449	0.00092393	0.00379729	Up	ATEG_08400	hypothetical protein	ko01100//Metabolic pathways	-	GO:0016787//hydrolase activity	GO:0008152//metabolic process	gi|115433769|ref|XP_001217021.1|/0/conserved hypothetical protein [Aspergillus terreus NIH2624]
4353516	864	313.9	491.2	1.260663482	1.94E-08	1.64E-07	Up	ATEG_08296	similar to pirin	-	-	-	-	gi|115433560|ref|XP_001216917.1|/0/hypothetical protein ATEG_08296 [Aspergillus terreus NIH2624]
4321802	1692	123.87	194.12	1.258553459	0.00010161	0.00050846	Up	ATEG_05819	hypothetical protein	-	-	-	-	gi|115398816|ref|XP_001214997.1|/0/conserved hypothetical protein [Aspergillus terreus NIH2624]
4353723	2274	1632.46	2520.07	1.256684947	1.86E-20	3.89E-19	Up	ATEG_09249	hypothetical protein	-	GO:0005634//nucleus	GO:0000981//RNA polymerase II transcription factor activity, sequence-specific DNA binding;GO:0008270//zinc ion binding	GO:0006357//regulation of transcription from RNA polymerase II promoter	gi|115437676|ref|XP_001217871.1|/0/conserved hypothetical protein [Aspergillus terreus NIH2624]
4316088	2512	211.34	328.46	1.256610603	1.03E-05	6.10E-05	Up	ATEG_01009	hypothetical protein	-	GO:0005634//nucleus	GO:0003677//DNA binding;GO:0008270//zinc ion binding	GO:0006351//transcription, DNA-templated	gi|115383654|ref|XP_001208374.1|/0/predicted protein [Aspergillus terreus NIH2624]

4322682	1881	1597.85	2465.66	1.256008755	2.12E-19	4.18E-18	Up	ATEG_07743	similar to fumarate reductase	ko01100//Metabolic pathways;ko01110//Biosynthesis of secondary metabolites;ko00350//Tyrosine metabolism;ko00380//Tryptophan metabolism;ko00360//Phenylalanine metabolism;ko00340//Histidine metabolism	-	GO:0000104//succinate dehydrogenase activity	GO:0055114//oxidation-reduction process	gi|115401552|ref|XP_001216364.1|/0/hypothetical protein ATEG_07743 [Aspergillus terreus NIH2624]
4321479	1683	109.82	177.37	1.255383631	0.00086752	0.0035906	Up	ATEG_06210	hypothetical protein	ko01100//Metabolic pathways;ko01110//Biosynthesis of secondary metabolites;ko01130//Biosynthesis of antibiotics;ko00230//Purine metabolism	GO:0016021//integral component of membrane	-	GO:0055085//transmembrane transport	gi|115399622|ref|XP_001215388.1|/0/predicted protein [Aspergillus terreus NIH2624]
4320639	6249	846.27	1306.55	1.255381194	2.71E-15	3.99E-14	Up	ATEG_05255	hypothetical protein	ko04144//Endocytosis	GO:0005634//nucleus	GO:0000981//RNA polymerase II transcription factor activity, sequence-specific DNA binding;GO:0003677//DNA binding;GO:0008270//zinc ion binding	GO:0006357//regulation of transcription from RNA polymerase II promoter	gi|115397683|ref|XP_001214433.1|/0/conserved hypothetical protein [Aspergillus terreus NIH2624]
4320812	7241	884.97	1365.92	1.255002964	3.19E-15	4.66E-14	Up	ATEG_05177	similar to alpha-glucosidase	ko01100//Metabolic pathways;ko00500//Starch and sucrose metabolism;ko00052//Galactose metabolism	GO:0005576//extracellular region	GO:0004558//alpha-1,4-glucosidase activity;GO:0008422//beta-glucosidase activity;GO:0030246//carbohydrate binding;GO:0032450//maltose alpha-glucosidase activity	GO:0000023//maltose metabolic process;GO:0000272//polysaccharide catabolic process;GO:0071555//cell wall organization	gi|115397527|ref|XP_001214355.1|/0/hypothetical protein ATEG_05177 [Aspergillus terreus NIH2624]

4316757	1302	112.68	176.59	1.254968493	0.00027342	0.00127383	Up	ATEG_02448	hypothetical protein	ko01100//Metabolic pathways;ko00051//Fructose and mannose metabolism;ko00650//Butanoate metabolism;ko00591//Linoleic acid metabolism	-	GO:0016491//oxidoreductase activity	GO:0055114//oxidation-reduction process	gi|115388241|ref|XP_001211626.1|/0/conserved hypothetical protein [Aspergillus terreus NIH2624]
4353210	1095	325.73	504.59	1.25333983	5.53E-07	3.97E-06	Up	ATEG_08411	hypothetical protein	ko04146//Peroxisome;ko00071//Fatty acid degradation	-	GO:0003824//catalytic activity	GO:0008152//metabolic process	gi|115433791|ref|XP_001217032.1|/0/conserved hypothetical protein [Aspergillus terreus NIH2624]
4323214	1770	6422.08	9856.94	1.250234412	1.32E-25	3.68E-24	Up	ATEG_08696	similar to di/tri peptide transporter 2	-	GO:0000324//fungal-type vacuole;GO:0016021//integral component of membrane;GO:0031520//plasma membrane of cell tip;GO:0032153//cell division site	GO:0042937//tripeptide transporter activity;GO:0071916//dipeptide transmembrane transporter activity	GO:0035442//dipeptide transmembrane transport;GO:0042939//tripeptide transport	gi|115402411|ref|XP_001217282.1|/0/hypothetical protein ATEG_08696 [Aspergillus terreus NIH2624]

4354387	1695	4950.55	7602.13	1.250152428	2.09E-26	6.12E-25	Up	ATEG_09625	serine hydroxymethyltransferase, mitochondrial precursor	ko01100//Metabolic pathways;ko01110//Biosynthesis of secondary metabolites;ko01130//Biosynthesis of antibiotics;ko01230//Biosynthesis of amino acids;ko01200//Carbon metabolism;ko00260//Glycine, serine and threonine metabolism;ko00460//Cyanoamino acid metabolism;ko00630//Glyoxylate and dicarboxylate metabolism;ko00680//Methane metabolism;ko00670//One carbon pool by folate	-	GO:0004372//glycine hydroxymethyltransferase activity;GO:0008168//methyltransferase activity;GO:0030170//pyridoxal phosphate binding	GO:0006544//glycine metabolic process;GO:0006563//L-serine metabolic process;GO:0032259//methylation;GO:0035999//tetrahydrofolate interconversion	gi|115442880|ref|XP_001218247.1|/0/serine hydroxymethyltransferase, mitochondrial precursor [Aspergillus terreus NIH2624]
4315701	588	62.2	98.45	1.24861962	0.00369979	0.01311488	Up	ATEG_01329	hypothetical protein	ko04011//MAPK signaling pathway - yeast;ko00620//Pyruvate metabolism	-	GO:0016829//lyase activity	-	gi|115384294|ref|XP_001208694.1|/1.02828e-149/conserved hypothetical protein [Aspergillus terreus NIH2624]
4323576	438	1744.29	2678.85	1.24661447	3.72E-19	7.13E-18	Up	ATEG_08567	40S ribosomal protein S23	ko03010//Ribosome	GO:0015935//small ribosomal subunit	GO:0003735//structural constituent of ribosome	GO:0006412//translation	gi|115402153|ref|XP_001217153.1|/1.20282e-101/40S ribosomal protein S23 [Aspergillus terreus NIH2624]

4355066	1249	8782.15	13479.64	1.245685206	1.21E-25	3.40E-24	Up	ATEG_00314	60S ribosomal protein L6	ko03010//Ribosome	GO:0005730//nucleolus;GO:0005829//cytosol;GO:0005840//ribosome	GO:0003735//structural constituent of ribosome	GO:0006412//translation	gi|115491545|ref|XP_001210400.1|/5.23603e-114/60S ribosomal protein L6 [Aspergillus terreus NIH2624]
4316753	1866	533	815.5	1.240193062	1.29E-11	1.49E-10	Up	ATEG_02444	hypothetical protein	-	GO:0005623//cell	GO:0004396//hexokinase activity;GO:0005524//ATP binding;GO:0005536//glucose binding	GO:0001678//cellular glucose homeostasis;GO:0006096//glycolytic process;GO:0046835//carbohydrate phosphorylation	gi|115388233|ref|XP_001211622.1|/0/conserved hypothetical protein [Aspergillus terreus NIH2624]
4318815	546	209.68	322.47	1.238558249	2.03E-05	0.00011578	Up	ATEG_04141	hypothetical protein	-	-	-	-	gi|115391629|ref|XP_001213319.1|/2.17572e-126/conserved hypothetical protein [Aspergillus terreus NIH2624]
4321117	813	249.99	381.99	1.237603634	1.24E-05	7.26E-05	Up	ATEG_05256	hypothetical protein	-	-	GO:0010181//FMN binding;GO:0016491//oxidoreductase activity	GO:0055114//oxidation-reduction process	gi|115397685|ref|XP_001214434.1|/0/conserved hypothetical protein [Aspergillus terreus NIH2624]
4354542	486	1674.25	2553.89	1.236631248	1.24E-18	2.28E-17	Up	ATEG_09624	hypothetical protein	ko04144//Endocytosis	-	-	-	gi|115442878|ref|XP_001218246.1|/1.40828e-110/predicted protein [Aspergillus terreus NIH2624]
4317914	348	88.56	138.02	1.235866709	0.00052233	0.00228806	Up	ATEG_03368	hypothetical protein	-	GO:0005622//intracellular	-	-	gi|115390082|ref|XP_001212546.1|/5.85876e-81/conserved hypothetical protein [Aspergillus terreus NIH2624]

4319993	1110	580.59	884.56	1.234052604	1.30E-12	1.64E-11	Up	ATEG_04700	homoserine dehydrogenase	ko01100//Metabolic pathways;ko01110//Biosynthesis of secondary metabolites;ko01130//Biosynthesis of antibiotics;ko01230//Biosynthesis of amino acids;ko00260//Glycine, serine and threonine metabolism;ko00270//Cysteine and methionine metabolism;ko00300//Lysine biosynthesis	GO:0005622//intracellular	GO:0004412//homoserine dehydrogenase activity;GO:0050661//NADP binding	GO:0009086//methionine biosynthetic process;GO:0009088//threonine biosynthetic process;GO:0009090//homoserine biosynthetic process;GO:0009097//isoleucine biosynthetic process;GO:0055114//oxidation-reduction process;GO:0071470//cellular response to osmotic stress	gi|115396478|ref|XP_001213878.1|/0/homoserine dehydrogenase [Aspergillus terreus NIH2624]
4320607	2422	660.98	1010.76	1.233383801	1.96E-09	1.84E-08	Up	ATEG_05165	hypothetical protein	-	GO:0016021//integral component of membrane	GO:0008324//cation transmembrane transporter activity	GO:0098655//cation transmembrane transport	gi|115397503|ref|XP_001214343.1|/0/predicted protein [Aspergillus terreus NIH2624]
4316915	1075	8418.92	12824.85	1.232844079	1.95E-23	4.80E-22	Up	ATEG_02206	60S ribosomal protein L7	ko03010//Ribosome	GO:0005840//ribosome	-	-	gi|115387757|ref|XP_001211384.1|/0/60S ribosomal protein L7 [Aspergillus terreus NIH2624]
4353962	2520	119.96	185.47	1.229463769	0.0001266	0.00062289	Up	ATEG_09329	hypothetical protein	ko01100//Metabolic pathways;ko01110//Biosynthesis of secondary metabolites;ko00500//Starch and sucrose metabolism;ko00460//Cyanoamino acid metabolism	GO:0005576//extracellular region	GO:0008422//beta-glucosidase activity	GO:0030245//cellulose catabolic process	gi|115437990|ref|XP_001217951.1|/0/conserved hypothetical protein [Aspergillus terreus NIH2624]

4353402	462	2461.47	3728.34	1.227721322	7.86E-20	1.59E-18	Up	ATEG_08066	nucleoside diphosphate kinase	ko01100//Metabolic pathways;ko01110//Biosynthesis of secondary metabolites;ko01130//Biosynthesis of antibiotics;ko00230//Purine metabolism;ko00240//Pyrimidine metabolism	GO:0005634//nucleus;GO:0005758//mitochondrial intermembrane space;GO:0005829//cytosol	GO:0004550//nucleoside diphosphate kinase activity;GO:0005524//ATP binding;GO:0042802//identical protein binding	GO:0006183//GTP biosynthetic process;GO:0006228//UTP biosynthetic process;GO:0006241//CTP biosynthetic process;GO:0006974//cellular response to DNA damage stimulus;GO:0061508//CDP phosphorylation	gi|115433100|ref|XP_001216687.1|/5.20448e-109/nucleoside diphosphate kinase [Aspergillus terreus NIH2624]
4317627	444	2387.23	3619.66	1.22472411	8.21E-16	1.26E-14	Up	ATEG_03096	40S ribosomal protein S19	ko03010//Ribosome	GO:0005840//ribosome	GO:0003735//structural constituent of ribosome	GO:0006412//translation	gi|115389538|ref|XP_001212274.1|/5.07517e-104/40S ribosomal protein S19 [Aspergillus terreus NIH2624]
4353559	3702	227.53	346.49	1.223040587	3.51E-07	2.60E-06	Up	ATEG_08075	hypothetical protein	ko00310//Lysine degradation	GO:0000142//cellular bud neck contractile ring;GO:0044697//HICS complex;GO:0051286//cell tip	GO:0030234//enzyme regulator activity	GO:0000917//barrier septum assembly;GO:0050790//regulation of catalytic activity	gi|115433118|ref|XP_001216696.1|/0/conserved hypothetical protein [Aspergillus terreus NIH2624]
4353883	1855	375.45	566.42	1.22046369	9.87E-09	8.60E-08	Up	ATEG_09154	hypothetical protein	-	GO:0016021//integral component of membrane	-	GO:0055085//transmembrane transport	gi|115437302|ref|XP_001217776.1|/1.88925e-147/predicted protein [Aspergillus terreus NIH2624]

4354109	9400	14746.56	22165.97	1.218395339	1.11E-28	3.53E-27	Up	ATEG_09637	translational activator GCN1	ko03010//Ribosome	GO:0005840//ribosome	GO:0003735//structural constituent of ribosome;GO:0019887//protein kinase regulator activity;GO:0019901//protein kinase binding;GO:0043022//ribosome binding	GO:0006417//regulation of translation;GO:0033554//cellular response to stress;GO:0033674//positive regulation of kinase activity;GO:0045859//regulation of protein kinase activity	gi|115442904|ref|XP_001218259.1|/0/translational activator GCN1 [Aspergillus terreus NIH2624]
4316026	1989	327.93	495.82	1.218283244	8.23E-08	6.57E-07	Up	ATEG_01359	similar to vacuolar sorting protein	-	GO:0016021//integral component of membrane	-	GO:0006904//vesicle docking involved in exocytosis	gi|115384354|ref|XP_001208724.1|/0/hypothetical protein ATEG_01359 [Aspergillus terreus NIH2624]
4320546	981	357.5	542.85	1.217350176	7.75E-09	6.84E-08	Up	ATEG_04768	methylenetetrahydrofolate dehydrogenase	ko01100//Metabolic pathways;ko00670//One carbon pool by folate	GO:0005634//nucleus;GO:0005829//cytosol	GO:0004487//methylenetetrahydrofolate dehydrogenase (NAD+) activity;GO:0004488//methylenetetrahydrofolate dehydrogenase (NADP+) activity	GO:0006730//one-carbon metabolic process;GO:0009113//purine nucleobase biosynthetic process;GO:0009396//folic acid-containing compound biosynthetic process;GO:0055114//oxidation-reduction process	gi|115396614|ref|XP_001213946.1|/0/methylenetetrahydrofolate dehydrogenase [Aspergillus terreus NIH2624]
4319925	939	249.3	377.19	1.216486157	1.76E-06	1.18E-05	Up	ATEG_04455	similar to aldose reductase	ko01100//Metabolic pathways;ko00561//Glycerolipid metabolism	-	GO:0016491//oxidoreductase activity	GO:0055114//oxidation-reduction process	gi|115395988|ref|XP_001213633.1|/0/hypothetical protein ATEG_04455 [Aspergillus terreus NIH2624]

4354416	1596	347.85	528.57	1.216394188	6.95E-07	4.92E-06	Up	ATEG_09594	hypothetical protein	ko01100//Metabolic pathways;ko01110//Biosynthesis of secondary metabolites;ko01130//Biosynthesis of antibiotics;ko01220//Degradation of aromatic compounds;ko00350//Tyrosine metabolism;ko00010//Glycolysis / Gluconeogenesis;ko00071//Fatty acid degradation	GO:0005886//plasma membrane;GO:0016021//integral component of membrane	GO:0031924//vitamin B6 transporter activity	GO:0031919//vitamin B6 transport;GO:0055085//transmembrane transport	gi|115442818|ref|XP_001218216.1|/0/conserved hypothetical protein [Aspergillus terreus NIH2624]
4323018	1359	531.21	799.2	1.214709247	1.73E-11	1.98E-10	Up	ATEG_07846	tubulin alpha chain	ko04145//Phagosome	GO:0005737//cytoplasm;GO:0005874//microtubule	GO:0003924//GTPase activity;GO:0005200//structural constituent of cytoskeleton;GO:0005525//GTP binding	GO:0007017//microtubule-based process	gi|115401758|ref|XP_001216467.1|/0/tubulin alpha chain [Aspergillus terreus NIH2624]
4320651	4368	1397.33	2104.48	1.213477902	4.19E-15	6.04E-14	Up	ATEG_05322	nucleolar GTP-binding protein 2	ko03008//Ribosome biogenesis in eukaryotes	GO:0005654//nucleoplasm;GO:0005730//nucleolus;GO:0030687//preribosome, large subunit precursor	GO:0005525//GTP binding	GO:0000055//ribosomal large subunit export from nucleus	gi|115397817|ref|XP_001214500.1|/0/nucleolar GTP-binding protein 2 [Aspergillus terreus NIH2624]
4317967	6459	43.15	67.95	1.211809058	0.01113214	0.03401265	Up	ATEG_03528	similar to peptide synthetase	-	-	GO:0003824//catalytic activity;GO:0031177//phosphopantetheine binding	GO:0008152//metabolic process	gi|115390402|ref|XP_001212706.1|/0/hypothetical protein ATEG_03528 [Aspergillus terreus NIH2624]
4316448	3879	84.56	133.64	1.209878981	0.00584643	0.01951513	Up	ATEG_01893	hypothetical protein	-	-	GO:0003676//nucleic acid binding	GO:0015074//DNA integration	gi|115387131|ref|XP_001211071.1|/0/predicted protein [Aspergillus terreus NIH2624]

4323210	2763	1355.02	2020.14	1.206972054	3.77E-17	6.29E-16	Up	ATEG_08613	hypothetical protein	-	-	GO:0005524//ATP binding	-	gi|115402245|ref|XP_001217199.1|/0/predicted protein [Aspergillus terreus NIH2624]
4354138	2305	149.62	227.09	1.206961437	2.48E-05	0.00013957	Up	ATEG_09633	hypothetical protein	-	GO:0016021//integral component of membrane	GO:0022891//substrate-specific transmembrane transporter activity	GO:0055085//transmembrane transport	gi|115442896|ref|XP_001218255.1|/0/predicted protein [Aspergillus terreus NIH2624]
4316669	500	4561.69	6834.02	1.20647575	4.25E-20	8.73E-19	Up	ATEG_02305	60S ribosomal protein L32	ko03010//Ribosome	GO:0022625//cytosolic large ribosomal subunit	GO:0003735//structural constituent of ribosome	GO:0006412//translation	gi|115387955|ref|XP_001211483.1|/5.96783e-93/60S ribosomal protein L32 [Aspergillus terreus NIH2624]
4323187	822	92.52	141.83	1.204395444	0.00146241	0.00575818	Up	ATEG_08672	similar to glutamic protease eqolisin	ko04144//Endocytosis	-	GO:0004190//aspartic-type endopeptidase activity	GO:0006508//proteolysis	gi|115402363|ref|XP_001217258.1|/0/hypothetical protein ATEG_08672 [Aspergillus terreus NIH2624]
4315839	1413	396.77	598.02	1.20436006	9.49E-09	8.31E-08	Up	ATEG_01186	acetylornithine aminotransferase, mitochondrial precursor	ko01100//Metabolic pathways;ko01110//Biosynthesis of secondary metabolites;ko01130//Biosynthesis of antibiotics;ko01230//Biosynthesis of amino acids;ko01210//2-Oxocarboxylic acid metabolism;ko00220//Arginine biosynthesis	-	GO:0008483//transaminase activity;GO:0030170//pyridoxal phosphate binding	GO:0006525//arginine metabolic process	gi|115384008|ref|XP_001208551.1|/0/acetylornithine aminotransferase, mitochondrial precursor [Aspergillus terreus NIH2624]

4317758	1545	436.65	661.53	1.203807878	2.44E-07	1.85E-06	Up	ATEG_03262	anthranilate synthase component I	ko01100//Metabolic pathways;ko01110//Biosynthesis of secondary metabolites;ko01230//Biosynthesis of amino acids;ko00400//Phenylalanine, tyrosine and tryptophan biosynthesis	-	GO:0004049//anthranilate synthase activity	GO:0000162//tryptophan biosynthetic process;GO:0070791//cleistothecium development	gi|115389870|ref|XP_001212440.1|/0/anthranilate synthase component I [Aspergillus terreus NIH2624]
4320933	3807	1374.14	2044.72	1.202416724	6.19E-18	1.08E-16	Up	ATEG_05182	hypothetical protein	-	GO:0005634//nucleus	GO:0000981//RNA polymerase II transcription factor activity, sequence-specific DNA binding;GO:0003677//DNA binding;GO:0008270//zinc ion binding	GO:0006357//regulation of transcription from RNA polymerase II promoter	gi|115397537|ref|XP_001214360.1|/0/conserved hypothetical protein [Aspergillus terreus NIH2624]
4355333	4373	1691.98	2517.57	1.202288708	7.16E-19	1.33E-17	Up	ATEG_00579	isoleucyl-tRNA synthetase	ko00970//Aminoacyl-tRNA biosynthesis	GO:0005829//cytosol	GO:0002161//aminoacyl-tRNA editing activity;GO:0004822//isoleucine-tRNA ligase activity;GO:0005524//ATP binding	GO:0006428//isoleucyl-tRNA aminoacylation;GO:0006450//regulation of translational fidelity	gi|115492075|ref|XP_001210665.1|/0/isoleucyl-tRNA synthetase [Aspergillus terreus NIH2624]
4318267	2595	251.63	374.49	1.196192067	6.39E-07	4.54E-06	Up	ATEG_03671	hypothetical protein	-	GO:0005634//nucleus;GO:0016021//integral component of membrane	GO:0000981//RNA polymerase II transcription factor activity, sequence-specific DNA binding;GO:0003677//DNA binding;GO:0008270//zinc ion binding	GO:0006357//regulation of transcription from RNA polymerase II promoter	gi|115390689|ref|XP_001212849.1|/0/conserved hypothetical protein [Aspergillus terreus NIH2624]

4354022	1410	234.23	348.71	1.193898908	8.90E-07	6.15E-06	Up	ATEG_09237	hypothetical protein	-	-	-	-	gi|115437630|ref|XP_001217859.1|/0/predicted protein [Aspergillus terreus NIH2624]
4317042	1650	1957.68	2922.2	1.193200288	4.89E-13	6.31E-12	Up	ATEG_02175	hypothetical protein	-	-	-	-	gi|115387695|ref|XP_001211353.1|/0/conserved hypothetical protein [Aspergillus terreus NIH2624]
4317767	2512	300.94	446.9	1.1925904	5.06E-08	4.14E-07	Up	ATEG_02976	hypothetical protein	ko01100//Metabolic pathways;ko00600//Sphingolipid metabolism	GO:0016021//integral component of membrane	-	-	gi|115389298|ref|XP_001212154.1|/0/conserved hypothetical protein [Aspergillus terreus NIH2624]
4354198	909	414.23	612.04	1.192470543	1.36E-07	1.06E-06	Up	ATEG_09779	hypothetical protein	ko01100//Metabolic pathways;ko00520//Amino sugar and nucleotide sugar metabolism	-	-	-	gi|115443188|ref|XP_001218401.1|/0/conserved hypothetical protein [Aspergillus terreus NIH2624]
4317284	1076	5234.63	7737.29	1.192180285	2.20E-22	5.18E-21	Up	ATEG_02542	60S ribosomal protein L12	ko03010//Ribosome	GO:0005840//ribosome	GO:0003735//structural constituent of ribosome	GO:0006412//translation	gi|115388429|ref|XP_001211720.1|/3.87397e-133/60S ribosomal protein L12 [Aspergillus terreus NIH2624]
4322589	852	281.25	423.46	1.191738717	3.41E-06	2.21E-05	Up	ATEG_07930	hypothetical protein	-	-	-	-	gi|115401926|ref|XP_001216551.1|/0/conserved hypothetical protein [Aspergillus terreus NIH2624]
4318457	903	2936.92	4339.53	1.191648283	4.45E-22	1.02E-20	Up	ATEG_04078	40S ribosomal protein S0	ko03010//Ribosome	GO:0022627//cytosolic small ribosomal subunit	GO:0003735//structural constituent of ribosome	GO:0000028//ribosomal small subunit assembly;GO:0006412//translation	gi|115391503|ref|XP_001213256.1|/0/40S ribosomal protein S0 [Aspergillus terreus NIH2624]

4353079	1374	490.25	730.3	1.18949682	4.04E-09	3.70E-08	Up	ATEG_08315	arginine biosynthesis bifunctional protein ARG7, mitochondrial precursor	ko01100//Metabolic pathways;ko01110//Biosynthesis of secondary metabolites;ko01130//Biosynthesis of antibiotics;ko01230//Biosynthesis of amino acids;ko01210//2-Oxocarboxylic acid metabolism;ko00220//Arginine biosynthesis	GO:0005759//mitochondrial matrix	GO:0004042//acetyl-CoA:L-glutamate N-acetyltransferase activity;GO:0004358//glutamate N-acetyltransferase activity	GO:0006526//arginine biosynthetic process;GO:0006592//ornithine biosynthetic process	gi|115433598|ref|XP_001216936.1|/0/arginine biosynthesis bifunctional protein ARG7, mitochondrial precursor [Aspergillus terreus NIH2624]
4316265	1578	326.62	484.78	1.189253816	1.70E-07	1.31E-06	Up	ATEG_01776	hypothetical protein	ko00562//Inositol phosphate metabolism;ko04145//Phagosome;ko04070//Phosphatidylinositol signaling system	GO:0043227//membrane-bounded organelle;GO:0043229//intracellular organelle;GO:0044444//cytoplasmic part	GO:0008270//zinc ion binding;GO:0016740//transferase activity	GO:0044237//cellular metabolic process	gi|115385188|ref|XP_001209141.1|/0/predicted protein [Aspergillus terreus NIH2624]
4322490	1149	161.35	240.8	1.185397871	3.44E-05	0.00018912	Up	ATEG_07571	hypothetical protein	ko01100//Metabolic pathways;ko01110//Biosynthesis of secondary metabolites;ko00130//Ubiquinone and other terpenoid-quinone biosynthesis	-	GO:0003677//DNA binding	-	gi|115401208|ref|XP_001216192.1|/0/predicted protein [Aspergillus terreus NIH2624]
4322780	756	374.9	554.48	1.183857644	4.33E-09	3.95E-08	Up	ATEG_07688	hypothetical protein	ko01100//Metabolic pathways;ko00230//Purine metabolism	-	GO:0016740//transferase activity	GO:0006541//glutamine metabolic process	gi|115401442|ref|XP_001216309.1|/0/conserved hypothetical protein [Aspergillus terreus NIH2624]
4355184	1892	3190.07	4696.54	1.18378999	1.94E-20	4.04E-19	Up	ATEG_00430	similar to GabA permease	-	GO:0016021//integral component of membrane	GO:0015171//amino acid transmembrane transporter activity	GO:0003333//amino acid transmembrane transport	gi|115491777|ref|XP_001210516.1|/0/hypothetical protein ATEG_00430 [Aspergillus terreus NIH2624]

4318025	4377	173.73	257.63	1.182723174	1.23E-05	7.22E-05	Up	ATEG_03047	beta-glucosidase 1 precursor	ko01100//Metabolic pathways;ko01110//Biosynthesis of secondary metabolites;ko00500//Starch and sucrose metabolism;ko00460//Cyanoamino acid metabolism	GO:0005576//extracellular region	GO:0008422//beta-glucosidase activity	GO:0030245//cellulose catabolic process	gi|115389440|ref|XP_001212225.1|/0/beta-glucosidase 1 precursor [Aspergillus terreus NIH2624]
4320422	606	2896.44	4255.46	1.18239011	1.45E-20	3.06E-19	Up	ATEG_04602	40S ribosomal protein S7	ko03010//Ribosome	GO:0005730//nucleolus;GO:0005829//cytosol;GO:0005840//ribosome	GO:0003735//structural constituent of ribosome	GO:0006412//translation	gi|115396282|ref|XP_001213780.1|/9.36582e-140/40S ribosomal protein S7 [Aspergillus terreus NIH2624]
4322187	963	4782.82	7003.92	1.181984495	5.44E-19	1.02E-17	Up	ATEG_06544	transaldolase	ko01100//Metabolic pathways;ko01110//Biosynthesis of secondary metabolites;ko01130//Biosynthesis of antibiotics;ko01230//Biosynthesis of amino acids;ko01200//Carbon metabolism;ko00030//Pentose phosphate pathway	GO:0005634//nucleus;GO:0005829//cytosol	GO:0004801//sedoheptulose-7-phosphate:D-glyceraldehyde-3-phosphate glyceronetransferase activity	GO:0005975//carbohydrate metabolic process;GO:0006098//pentose-phosphate shunt	gi|115400267|ref|XP_001215722.1|/0/transaldolase [Aspergillus terreus NIH2624]
4322531	1098	88.06	132.42	1.178458237	0.00754717	0.02432499	Up	ATEG_07814	similar to HAPB	-	-	GO:0003700//transcription factor activity, sequence-specific DNA binding	GO:0006355//regulation of transcription, DNA-templated	gi|115401694|ref|XP_001216435.1|/0/hypothetical protein ATEG_07814 [Aspergillus terreus NIH2624]

4321730	2023	736.02	1079.57	1.17725243	2.30E-10	2.36E-09	Up	ATEG_05868	hypothetical protein	ko01100//Metabolic pathways;ko00500//Starch and sucrose metabolism	GO:0051285//cell cortex of cell tip	-	-	gi|115398914|ref|XP_001215046.1|/0/conserved hypothetical protein [Aspergillus terreus NIH2624]
4323287	777	4312.27	6322.05	1.177048772	4.69E-16	7.30E-15	Up	ATEG_08693	40S ribosomal protein S2	ko03010//Ribosome	GO:0022627//cytosolic small ribosomal subunit;GO:0032040//small-subunit processome	GO:0003735//structural constituent of ribosome;GO:0070181//small ribosomal subunit rRNA binding	GO:0006407//rRNA export from nucleus;GO:0045903//positive regulation of translational fidelity	gi|115402405|ref|XP_001217279.1|/0/40S ribosomal protein S2 [Aspergillus terreus NIH2624]
4353370	2151	1564.83	2288.12	1.17685802	8.05E-17	1.32E-15	Up	ATEG_08056	glutamyl-tRNA synthetase	ko01100//Metabolic pathways;ko01110//Biosynthesis of secondary metabolites;ko00970//Aminoacyl-tRNA biosynthesis;ko00860//Porphyrin and chlorophyll metabolism	GO:0005634//nucleus;GO:0005739//mitochondrion;GO:0005829//cytosol;GO:0017102//methionyl glutamyl tRNA synthetase complex	GO:0004818//glutamate-tRNA ligase activity;GO:0004819//glutamine-tRNA ligase activity;GO:0005524//ATP binding	GO:0006424//glutamyl-tRNA aminoacylation	gi|115433080|ref|XP_001216677.1|/0/glutamyl-tRNA synthetase [Aspergillus terreus NIH2624]
4320477	5388	212.35	314.51	1.176792546	4.59E-06	2.88E-05	Up	ATEG_04667	similar to ATP-binding cassette	ko02010//ABC transporters	GO:0016021//integral component of membrane	GO:0005524//ATP binding;GO:0042626//ATPase activity, coupled to transmembrane movement of substances	GO:0055085//transmembrane transport	gi|115396412|ref|XP_001213845.1|/0/hypothetical protein ATEG_04667 [Aspergillus terreus NIH2624]
4318582	1719	112.64	167.96	1.176679299	0.00026403	0.00123188	Up	ATEG_04171	hypothetical protein	ko04111//Cell cycle - yeast	GO:0005634//nucleus	GO:0043565//sequence-specific DNA binding	GO:0006355//regulation of transcription, DNA-templated	gi|115391689|ref|XP_001213349.1|/0/conserved hypothetical protein [Aspergillus terreus NIH2624]
4321851	1451	186.08	275.1	1.175735569	9.49E-06	5.67E-05	Up	ATEG_05645	hypothetical protein	-	-	-	-	gi|115398468|ref|XP_001214823.1|/0/predicted protein [Aspergillus terreus NIH2624]

4318954	1301	1054.15	1533.28	1.175278863	2.33E-11	2.62E-10	Up	ATEG_07111	hypothetical protein	ko01100//Metabolic pathways;ko01110//Biosynthesis of secondary metabolites;ko00564//Glycerophospholipid metabolism	GO:0016021//integral component of membrane	-	-	gi|115386512|ref|XP_001209797.1|/0/predicted protein [Aspergillus terreus NIH2624]
4317502	2118	178.77	263.18	1.175088373	1.57E-05	9.07E-05	Up	ATEG_03039	hypothetical protein	ko04144//Endocytosis	GO:0016021//integral component of membrane	-	-	gi|115389424|ref|XP_001212217.1|/0/predicted protein [Aspergillus terreus NIH2624]
4317801	990	170.87	252.06	1.169256689	3.78E-05	0.00020637	Up	ATEG_03118	hypothetical protein	-	GO:0005743//mitochondrial inner membrane;GO:0016021//integral component of membrane	GO:0005215//transporter activity	GO:0006783//heme biosynthetic process;GO:0006810//transport	gi|115389582|ref|XP_001212296.1|/0/conserved hypothetical protein [Aspergillus terreus NIH2624]
4318170	2616	505.56	739.27	1.169122674	5.93E-10	5.81E-09	Up	ATEG_04052	hypothetical protein	-	GO:0016021//integral component of membrane	GO:0008271//secondary active sulfate transmembrane transporter activity	GO:1902358//sulfate transmembrane transport	gi|115391451|ref|XP_001213230.1|/0/conserved hypothetical protein [Aspergillus terreus NIH2624]

4317900	864	2767.68	4025.15	1.168564992	6.97E-21	1.50E-19	Up	ATEG_03200	similar to activated protein kinase C receptor	-	GO:0022627//cytosolic small ribosomal subunit	GO:0001965//G-protein alpha-subunit binding;GO:0004871//signal transducer activity;GO:0005080//protein kinase C binding;GO:0005092//GDP-dissociation inhibitor activity;GO:0043022//ribosome binding	GO:0001403//invasive growth in response to glucose limitation;GO:0007186//G-protein coupled receptor signaling pathway;GO:0010255//glucose mediated signaling pathway;GO:0017148//negative regulation of translation;GO:0031139//positive regulation of conjugation with cellular fusion;GO:0031954//positive regulation of protein autophosphorylation;GO:0032995//regulation of fungal-type cell wall biogenesis;GO:0034613//cellular protein localization;GO:0035556//intracellular signal transduction;GO:0035690//cellular response to [illegible]	gi|115389746|ref|XP_001212378.1|/0/hypothetical protein ATEG_03200 [Aspergillus terreus NIH2624]
4322275	7221	410.82	597.87	1.168353907	1.14E-08	9.81E-08	Up	ATEG_06597	hypothetical protein	-	GO:0016459//myosin complex	GO:0003774//motor activity;GO:0003779//actin binding;GO:0005524//ATP binding	-	gi|115400373|ref|XP_001215775.1|/0/hypothetical protein ATEG_06597 [Aspergillus terreus NIH2624]

4355337	1752	82.4	122.7	1.16785662	0.00206023	0.0078232	Up	ATEG_00583	hypothetical protein	-	GO:0016021//integral component of membrane	-	-	gi|115492083|ref|XP_001210669.1|/0/conserved hypothetical protein [Aspergillus terreus NIH2624]
4315995	1580	289.15	422.13	1.167612694	1.93E-07	1.48E-06	Up	ATEG_01691	hypothetical protein	ko04144//Endocytosis	-	-	-	gi|115385018|ref|XP_001209056.1|/1.38072e-171/predicted protein [Aspergillus terreus NIH2624]
4322186	492	1408.83	2042.59	1.167531118	6.12E-16	9.45E-15	Up	ATEG_06543	superoxide dismutase	ko04146//Peroxisome	GO:0005576//extracellular region;GO:0005737//cytoplasm	GO:0004784//superoxide dismutase activity;GO:0005507//copper ion binding;GO:0008270//zinc ion binding	GO:0010106//cellular response to iron ion starvation;GO:0019430//removal of superoxide radicals;GO:0055114//oxidation-reduction process	gi|115400265|ref|XP_001215721.1|/6.70375e-119/superoxide dismutase [Aspergillus terreus NIH2624]
4355716	1920	79	117.17	1.166442745	0.00437106	0.01522407	Up	ATEG_00953	hypothetical protein	ko01100//Metabolic pathways;ko01110//Biosynthesis of secondary metabolites;ko01130//Biosynthesis of antibiotics;ko01230//Biosynthesis of amino acids;ko00400//Phenylalanine, tyrosine and tryptophan biosynthesis	GO:0005634//nucleus	GO:0003677//DNA binding;GO:0008270//zinc ion binding	GO:0006351//transcription, DNA-templated	gi|115492823|ref|XP_001211039.1|/0/predicted protein [Aspergillus terreus NIH2624]

4355413	1527	414.72	604.48	1.161416939	5.51E-09	4.95E-08	Up	ATEG_00658	mannosyl-oligosaccharide alpha-1,2-mannosidase precursor	ko01100//Metabolic pathways;ko04141//Protein processing in endoplasmic reticulum;ko00510//N-Glycan biosynthesis;ko00513//Various types of N-glycan biosynthesis	GO:0016020//membrane;GO:0060205//cytoplasmic vesicle lumen	GO:0004571//mannosyl-oligosaccharide 1,2-alpha-mannosidase activity;GO:0005509//calcium ion binding	GO:0005975//carbohydrate metabolic process;GO:0006486//protein glycosylation	gi|115492233|ref|XP_001210744.1|/0/mannosyl-oligosaccharide alpha-1,2-mannosidase precursor [Aspergillus terreus NIH2624]
4318180	3576	1784.99	2573.14	1.161104992	1.57E-15	2.33E-14	Up	ATEG_04041	glutamate--cysteine ligase	ko01100//Metabolic pathways;ko00270//Cysteine and methionine metabolism;ko00480//Glutathione metabolism	-	GO:0004357//glutamate-cysteine ligase activity	GO:0006750//glutathione biosynthetic process	gi|115391429|ref|XP_001213219.1|/0/glutamate--cysteine ligase [Aspergillus terreus NIH2624]
4353578	981	215.74	314.45	1.160038865	3.08E-06	2.00E-05	Up	ATEG_08432	hypothetical protein	-	-	-	-	gi|115433833|ref|XP_001217053.1|/0/conserved hypothetical protein [Aspergillus terreus NIH2624]
4319998	1764	4189.47	6017.63	1.158574877	3.35E-15	4.88E-14	Up	ATEG_04310	general amino-acid permease GAP1	-	GO:0016021//integral component of membrane	GO:0015171//amino acid transmembrane transporter activity	GO:0003333//amino acid transmembrane transport	gi|115395202|ref|XP_001213488.1|/0/general amino-acid permease GAP1 [Aspergillus terreus NIH2624]

4317358	1110	143.03	214.33	1.158438614	0.00324066	0.01165917	Up	ATEG_03148	hypothetical protein	ko01100//Metabolic pathways;ko01110//Biosynthesis of secondary metabolites;ko01130//Biosynthesis of antibiotics;ko01230//Biosynthesis of amino acids;ko01200//Carbon metabolism;ko00260//Glycine, serine and threonine metabolism;ko00460//Cyanoamino acid metabolism;ko00630//Glyoxylate and dicarboxylate metabolism;ko00680//Methane metabolism;ko00670//One carbon pool by folate	GO:0016021//integral component of membrane	-	-	gi|115389642|ref|XP_001212326.1|/0/conserved hypothetical protein [Aspergillus terreus NIH2624]
4322325	1332	145.75	211.65	1.152951589	0.00025896	0.00121006	Up	ATEG_06635	2-oxoisovalerate dehydrogenase alpha subunit, mitochondrial precursor	ko01100//Metabolic pathways;ko01110//Biosynthesis of secondary metabolites;ko01130//Biosynthesis of antibiotics;ko00280//Valine, leucine and isoleucine degradation;ko00640//Propanoate metabolism	-	GO:0016624//oxidoreductase activity, acting on the aldehyde or oxo group of donors, disulfide as acceptor	GO:0055114//oxidation-reduction process	gi|115400449|ref|XP_001215813.1|/0/2-oxoisovalerate dehydrogenase alpha subunit, mitochondrial precursor [Aspergillus terreus NIH2624]

4318265	483	1807.5	2601.58	1.152605562	3.30E-15	4.80E-14	Up	ATEG_03669	40S ribosomal protein S11	ko03010//Ribosome	GO:0022627//cytosolic small ribosomal subunit	GO:0003735//structural constituent of ribosome	GO:0006412//translation	gi|115390685|ref|XP_001212847.1|/1.2096e-115/40S ribosomal protein S11 [Aspergillus terreus NIH2624]
4318653	2499	481.57	692.86	1.151355852	2.66E-07	2.00E-06	Up	ATEG_03794	succinyl-CoA:3-ketoacid-coenzyme A transferase 1, mitochondrial precursor	ko00650//Butanoate metabolism;ko00280//Valine, leucine and isoleucine degradation;ko00072//Synthesis and degradation of ketone bodies	GO:0005739//mitochondrion	GO:0008260//3-oxoacid CoA-transferase activity	GO:0046952//ketone body catabolic process	gi|115390935|ref|XP_001212972.1|/0/succinyl-CoA:3-ketoacid-coenzyme A transferase 1, mitochondrial precursor [Aspergillus terreus NIH2624]
4320750	1200	62.22	92.91	1.151195698	0.00639307	0.02111445	Up	ATEG_05617	deoxycytidylate deaminase	ko01100//Metabolic pathways;ko00240//Pyrimidine metabolism	-	GO:0008270//zinc ion binding;GO:0016787//hydrolase activity	-	gi|115398407|ref|XP_001214795.1|/0/deoxycytidylate deaminase [Aspergillus terreus NIH2624]
4319614	4080	239.86	346.06	1.149435812	1.91E-06	1.27E-05	Up	ATEG_09979	hypothetical protein	ko00480//Glutathione metabolism	-	GO:0016787//hydrolase activity	-	gi|115385469|ref|XP_001209281.1|/0/conserved hypothetical protein [Aspergillus terreus NIH2624]
4321839	754	3959.22	5681.07	1.14791372	4.89E-19	9.26E-18	Up	ATEG_05701	40S ribosomal protein S20	ko03010//Ribosome	GO:0022627//cytosolic small ribosomal subunit	GO:0003723//RNA binding;GO:0003735//structural constituent of ribosome	GO:0000462//maturation of SSU-rRNA from tricistronic rRNA transcript (SSU-rRNA, 5.8S rRNA, LSU-rRNA);GO:0006412//translation	gi|115398580|ref|XP_001214879.1|/3.8905e-89/40S ribosomal protein S20 [Aspergillus terreus NIH2624]
4353661	771	4561.25	6522.67	1.145130098	2.51E-21	5.58E-20	Up	ATEG_09484	40S ribosomal protein S1	ko03010//Ribosome	GO:0005576//extracellular region;GO:0022627//cytosolic small ribosomal subunit	GO:0003735//structural constituent of ribosome	GO:0006412//translation	gi|115438592|ref|XP_001218106.1|/2.64112e-180/40S ribosomal protein S1 [Aspergillus terreus NIH2624]

4321768	1671	902.54	1298.47	1.144151039	2.80E-10	2.85E-09	Up	ATEG_05895	phosphoenolpyruvate carboxykinase	ko01100//Metabolic pathways;ko01110//Biosynthesis of secondary metabolites;ko01130//Biosynthesis of antibiotics;ko01200//Carbon metabolism;ko00010//Glycolysis / Gluconeogenesis;ko00620//Pyruvate metabolism;ko00020//Citrate cycle (TCA cycle) [illegible]	GO:0005829//cytosol	GO:0004612//phosphoenolpyruvate carboxykinase (ATP) activity;GO:0004613//phosphoenolpyruvate carboxykinase (GTP) activity;GO:0005524//ATP binding;GO:0016301//kinase activity	GO:0006083//acetate metabolic process;GO:0006094//gluconeogenesis;GO:0006096//glycolytic process;GO:0015976//carbon utilization	gi|115398968|ref|XP_001215073.1|/0/phosphoenolpyruvate carboxykinase [Aspergillus terreus NIH2624]
4353828	807	280.73	404.72	1.142010045	6.51E-07	4.62E-06	Up	ATEG_09132	hypothetical protein	ko01100//Metabolic pathways;ko00770//Pantothenate and CoA biosynthesis	GO:0016021//integral component of membrane	-	-	gi|115437212|ref|XP_001217754.1|/0/conserved hypothetical protein [Aspergillus terreus NIH2624]
4321181	1882	697.02	994.09	1.14132899	2.91E-10	2.95E-09	Up	ATEG_05387	succinate-semialdehyde dehydrogenase	ko01100//Metabolic pathways;ko00350//Tyrosine metabolism;ko00650//Butanoate metabolism;ko00310//Lysine degradation;ko00250//Alanine, aspartate and glutamate metabolism	-	GO:0009013//succinate-semialdehyde dehydrogenase [NAD(P)+] activity	GO:0009450//gamma-aminobutyric acid catabolic process;GO:0055114//oxidation-reduction process	gi|115397947|ref|XP_001214565.1|/0/succinate-semialdehyde dehydrogenase [Aspergillus terreus NIH2624]
4317573	1233	6447.65	9180.33	1.138214299	2.22E-20	4.59E-19	Up	ATEG_03064	40S ribosomal protein S3	ko03010//Ribosome	GO:0005576//extracellular region;GO:0005829//cytosol;GO:0015935//small ribosomal subunit	GO:0003723//RNA binding;GO:0003735//structural constituent of ribosome	GO:0006412//translation	gi|115389474|ref|XP_001212242.1|/0/40S ribosomal protein S3 [Aspergillus terreus NIH2624]

4318618	1161	1631.66	2330.43	1.137593372	8.30E-15	1.17E-13	Up	ATEG_03746	hypothetical protein	ko00520//Amino sugar and nucleotide sugar metabolism	GO:0005741//mitochondrial outer membrane;GO:0016021//integral component of membrane	GO:0004128//cytochrome-b5 reductase activity, acting on NAD(P)H	GO:0055114//oxidation-reduction process	gi|115390839|ref|XP_001212924.1|/0/conserved hypothetical protein [Aspergillus terreus NIH2624]
4321001	2688	1077.04	1526.57	1.136011353	2.33E-12	2.85E-11	Up	ATEG_05047	hypothetical protein	-	-	-	-	gi|115397267|ref|XP_001214225.1|/0/conserved hypothetical protein [Aspergillus terreus NIH2624]
4321946	1901	7469.16	10565.53	1.132203098	1.35E-19	2.71E-18	Up	ATEG_06581	woronin body major protein	ko01100//Metabolic pathways;ko00500//Starch and sucrose metabolism	-	GO:0003746//translation elongation factor activity;GO:0043022//ribosome binding	GO:0006452//translational frameshifting;GO:0045901//positive regulation of translational elongation;GO:0045905//positive regulation of translational termination	gi|115400341|ref|XP_001215759.1|/0/woronin body major protein [Aspergillus terreus NIH2624]
4321075	1441	1665.25	2361.53	1.131337802	3.18E-15	4.65E-14	Up	ATEG_05417	60S ribosomal protein L38	ko03010//Ribosome	GO:0022625//cytosolic large ribosomal subunit	GO:0003735//structural constituent of ribosome	GO:0006412//translation	gi|115398007|ref|XP_001214595.1|/2.71061e-49/60S ribosomal protein L38 [Aspergillus terreus NIH2624]
4319661	1311	1210.83	1723.27	1.129115541	2.77E-12	3.38E-11	Up	ATEG_10179	coproporphyrinogen III oxidase, mitochondrial precursor	ko01100//Metabolic pathways;ko01110//Biosynthesis of secondary metabolites;ko00860//Porphyrin and chlorophyll metabolism	-	GO:0004109//coproporphyrinogen oxidase activity	GO:0006783//heme biosynthetic process;GO:0055114//oxidation-reduction process	gi|115385869|ref|XP_001209481.1|/0/coproporphyrinogen III oxidase, mitochondrial precursor [Aspergillus terreus NIH2624]
4316212	1095	2375.67	3345.91	1.128011024	6.62E-16	1.02E-14	Up	ATEG_01553	hypothetical protein	-	GO:0016021//integral component of membrane	GO:0004553//hydrolase activity, hydrolyzing O-glycosyl compounds	GO:0005975//carbohydrate metabolic process	gi|115384742|ref|XP_001208918.1|/0/conserved hypothetical protein [Aspergillus terreus NIH2624]

4321235	2278	6770.41	9562.31	1.127447681	9.58E-23	2.28E-21	Up	ATEG_05153	hypothetical protein	-	GO:0005634//nucleus;GO:0005829//cytosol;GO:0044732//mitotic spindle pole body;GO:0072686//mitotic spindle;GO:1990811//Msd1-Wdr8-Pkl1 complex	-	GO:0000070//mitotic sister chromatid segregation;GO:0034631;GO:1902440//protein localization to mitotic spindle pole body	gi|115397479|ref|XP_001214331.1|/0/conserved hypothetical protein [Aspergillus terreus NIH2624]
4315529	1227	1681.42	2375.41	1.125902676	1.29E-15	1.93E-14	Up	ATEG_01508	similar to mannitol-1-phosphate dehydrogenase	ko00051//Fructose and mannose metabolism	-	GO:0008926//mannitol-1-phosphate 5-dehydrogenase activity;GO:0050662//coenzyme binding	GO:0019594//mannitol metabolic process;GO:0055114//oxidation-reduction process	gi|115384652|ref|XP_001208873.1|/0/hypothetical protein ATEG_01508 [Aspergillus terreus NIH2624]
4316658	2573	498.78	705.16	1.125739363	1.21E-09	1.16E-08	Up	ATEG_02307	hypothetical protein	ko01100//Metabolic pathways;ko00190//Oxidative phosphorylation	GO:0005779//integral component of peroxisomal membrane	-	GO:0016559//peroxisome fission	gi|115387959|ref|XP_001211485.1|/3.4925e-172/conserved hypothetical protein [Aspergillus terreus NIH2624]
4318840	1590	854.14	1202.56	1.122291149	3.97E-12	4.79E-11	Up	ATEG_07129	hypothetical protein	ko01100//Metabolic pathways;ko00330//Arginine and proline metabolism;ko00410//beta-Alanine metabolism	-	GO:0016491//oxidoreductase activity	GO:0055114//oxidation-reduction process	gi|115386548|ref|XP_001209815.1|/0/conserved hypothetical protein [Aspergillus terreus NIH2624]
4355704	3738	733.4	1036.63	1.121952003	7.06E-07	4.99E-06	Up	ATEG_00941	5-oxoprolinase	ko00480//Glutathione metabolism	-	GO:0016787//hydrolase activity	-	gi|115492799|ref|XP_001211027.1|/0/5-oxoprolinase [Aspergillus terreus NIH2624]
4317147	1760	295.93	421.44	1.121325681	1.76E-06	1.18E-05	Up	ATEG_02619	hypothetical protein	ko00970//Aminoacyl-tRNA biosynthesis	GO:0005737//cytoplasm	GO:0004824//lysine-tRNA ligase activity;GO:0005524//ATP binding	GO:0006430//lysyl-tRNA aminoacylation	gi|115388583|ref|XP_001211797.1|/0/conserved hypothetical protein [Aspergillus terreus NIH2624]

4315825	1497	367.56	517.38	1.120528574	5.46E-07	3.93E-06	Up	ATEG_01639	aspartyl aminopeptidase	-	-	GO:0004177//aminopeptidase activity;GO:0008237//metallopeptidase activity;GO:0008270//zinc ion binding	GO:0006508//proteolysis	gi|115384914|ref|XP_001209004.1|/0/aspartyl aminopeptidase [Aspergillus terreus NIH2624]
4316619	900	513.85	719.42	1.119216033	8.58E-07	5.96E-06	Up	ATEG_02020	hypothetical protein	ko01110//Biosynthesis of secondary metabolites;ko00564//Glycerophospholipid metabolism;ko00561//Glycerolipid metabolism	-	-	-	gi|115387385|ref|XP_001211198.1|/0/conserved hypothetical protein [Aspergillus terreus NIH2624]
4354816	4004	331.73	467.43	1.118751726	2.26E-07	1.71E-06	Up	ATEG_00060	similar to kinesin	-	-	GO:0003777//microtubule motor activity;GO:0005524//ATP binding;GO:0008017//microtubule binding	GO:0007018//microtubule-based movement	gi|115491037|ref|XP_001210146.1|/0/hypothetical protein ATEG_00060 [Aspergillus terreus NIH2624]
4316728	1781	147.37	209.34	1.114233276	0.0001455	0.00070957	Up	ATEG_02585	hypothetical protein	-	GO:0016021//integral component of membrane	-	GO:0055085//transmembrane transport	gi|115388515|ref|XP_001211763.1|/0/conserved hypothetical protein [Aspergillus terreus NIH2624]
4319333	1488	546.97	764.21	1.113748876	1.20E-08	1.03E-07	Up	ATEG_07182	hypothetical protein	ko01100//Metabolic pathways;ko00230//Purine metabolism;ko00250//Alanine, aspartate and glutamate metabolism	GO:0016021//integral component of membrane	-	GO:0055085//transmembrane transport	gi|115386654|ref|XP_001209868.1|/0/conserved hypothetical protein [Aspergillus terreus NIH2624]
4316256	1836	480.91	681.91	1.112899381	4.31E-07	3.15E-06	Up	ATEG_01618	hypothetical protein	ko03440//Homologous recombination;ko03450//Non-homologous end-joining	GO:0016021//integral component of membrane	-	GO:0055085//transmembrane transport	gi|115384872|ref|XP_001208983.1|/0/conserved hypothetical protein [Aspergillus terreus NIH2624]

4315747	1334	458.94	641.14	1.112476121	7.86E-08	6.29E-07	Up	ATEG_01357	similar to NEDD8 activating enzyme	ko04120//Ubiquitin mediated proteolysis	-	GO:0005524//ATP binding;GO:0016881//acid-amino acid ligase activity;GO:0019781//NEDD8 activating enzyme activity	GO:0045116//protein neddylation	gi|115384350|ref|XP_001208722.1|/0/hypothetical protein ATEG_01357 [Aspergillus terreus NIH2624]
4319209	726	360.91	504.19	1.107982037	1.41E-07	1.09E-06	Up	ATEG_07196	similar to mutase	ko01100//Metabolic pathways;ko01110//Biosynthesis of secondary metabolites;ko01130//Biosynthesis of antibiotics;ko01230//Biosynthesis of amino acids;ko01200//Carbon metabolism;ko00260//Glycine, serine and threonine metabolism;ko00010//Glycolysis / Gluconeogenesis;ko00680//Methane metabolism	GO:0005829//cytosol	GO:0004619//phosphoglycerate mutase activity;GO:0050278//sedoheptulose-bisphosphatase activity	GO:0006094//gluconeogenesis;GO:0006096//glycolytic process;GO:0016311//dephosphorylation;GO:0046390//ribose phosphate biosynthetic process	gi|115386682|ref|XP_001209882.1|/9.69658e-180/hypothetical protein ATEG_07196 [Aspergillus terreus NIH2624]
4320754	1482	324.77	463.07	1.107940956	0.00086938	0.00359515	Up	ATEG_05621	similar to ATP-dependent RNA helicase	-	GO:0005730//nucleolus;GO:0030687//preribosome, large subunit precursor	GO:0003723//RNA binding;GO:0004004//ATP-dependent RNA helicase activity;GO:0005524//ATP binding	GO:0000027//ribosomal large subunit assembly;GO:0000464//endonucleolytic cleavage in ITS1 upstream of 5.8S rRNA from tricistronic rRNA transcript (SSU-rRNA, 5.8S rRNA, LSU-rRNA)	gi|115398415|ref|XP_001214799.1|/0/hypothetical protein ATEG_05621 [Aspergillus terreus NIH2624]

4353584	816	205.69	288.01	1.107809205	4.00E-05	0.00021775	Up	ATEG_08208	hypothetical protein	-	-	-	-	gi|115433384|ref|XP_001216829.1|/0/conserved hypothetical protein [Aspergillus terreus NIH2624]
4315778	675	536.31	746.77	1.104993697	1.33E-09	1.26E-08	Up	ATEG_01049	hypothetical protein	ko04144//Endocytosis	-	-	-	gi|115383734|ref|XP_001208414.1|/1.67536e-167/conserved hypothetical protein [Aspergillus terreus NIH2624]
4321203	513	75.66	110.23	1.099167131	0.01087841	0.03333318	Up	ATEG_05032	mitochondrial import receptor subunit tom-20	-	GO:0005742//mitochondrial outer membrane translocase complex;GO:0031307//integral component of mitochondrial outer membrane	GO:0030943//mitochondrion targeting sequence binding	GO:0016031//tRNA import into mitochondrion;GO:0030150//protein import into mitochondrial matrix;GO:0070096//mitochondrial outer membrane translocase complex assembly	gi|115397237|ref|XP_001214210.1|/1.3319e-117/mitochondrial import receptor subunit tom-20 [Aspergillus terreus NIH2624]
4354436	1305	377.73	527.44	1.098945908	1.20E-07	9.39E-07	Up	ATEG_09862	protein SUA5	-	GO:0005634//nucleus;GO:0005829//cytosol	GO:0003725//double-stranded RNA binding;GO:0005524//ATP binding;GO:0061710//L-threonylcarbamoyladenylate synthase	GO:0008033//tRNA processing	gi|115443354|ref|XP_001218484.1|/0/protein SUA5 [Aspergillus terreus NIH2624]
4321068	2436	59.39	84.78	1.098642564	0.01175599	0.03564022	Up	ATEG_05089	hypothetical protein	ko03040//Spliceosome	-	GO:0003824//catalytic activity	-	gi|115397351|ref|XP_001214267.1|/0/conserved hypothetical protein [Aspergillus terreus NIH2624]

4317770	1746	296.46	412.52	1.097910181	7.78E-07	5.44E-06	Up	ATEG_02979	hypothetical protein	-	GO:0016021//integral component of membrane	GO:0005215//transporter activity	GO:0055085//transmembrane transport	gi|115389304|ref|XP_001212157.1|/0/conserved hypothetical protein [Aspergillus terreus NIH2624]
4316736	939	5506.66	7605.57	1.097281813	5.30E-21	1.15E-19	Up	ATEG_02576	60S acidic ribosomal protein P0	ko03010//Ribosome	GO:0022625//cytosolic large ribosomal subunit	GO:0003735//structural constituent of ribosome;GO:0070180//large ribosomal subunit rRNA binding	GO:0000027//ribosomal large subunit assembly;GO:0002181//cytoplasmic translation	gi|115388497|ref|XP_001211754.1|/0/60S acidic ribosomal protein P0 [Aspergillus terreus NIH2624]
4353403	2091	961.56	1324.98	1.095485808	1.75E-10	1.81E-09	Up	ATEG_08067	hypothetical protein	ko01100//Metabolic pathways;ko01200//Carbon metabolism;ko00670//One carbon pool by folate	-	GO:0004489//methylenetetrahydrofolate reductase (NAD(P)H) activity	GO:0009086//methionine biosynthetic process;GO:0035999//tetrahydrofolate interconversion;GO:0055114//oxidation-reduction process	gi|115433102|ref|XP_001216688.1|/0/conserved hypothetical protein [Aspergillus terreus NIH2624]
4354772	3711	283.56	392.82	1.095365163	3.18E-06	2.06E-05	Up	ATEG_00016	hypothetical protein	ko03015//mRNA surveillance pathway	GO:0005739//mitochondrion;GO:0005829//cytosol;GO:0005847//mRNA cleavage and polyadenylation specificity factor complex	GO:0003723//RNA binding	GO:0006369//termination of RNA polymerase II transcription;GO:0006378//mRNA polyadenylation;GO:0006379//mRNA cleavage	gi|115490949|ref|XP_001210102.1|/0/conserved hypothetical protein [Aspergillus terreus NIH2624]
4320805	2639	705.07	973.83	1.090997454	1.44E-10	1.51E-09	Up	ATEG_05193	hypothetical protein	ko01100//Metabolic pathways;ko00564//Glycerophospholipid metabolism	GO:0005634//nucleus	GO:0016301//kinase activity;GO:0016773//phosphotransferase activity, alcohol group as acceptor	GO:0016310//phosphorylation	gi|115397559|ref|XP_001214371.1|/0/conserved hypothetical protein [Aspergillus terreus NIH2624]
4321136	1869	450.68	627.41	1.090195703	7.27E-07	5.12E-06	Up	ATEG_05054	hypothetical protein	-	GO:0016021//integral component of membrane	GO:0015238//drug transmembrane transporter activity;GO:0015297//antiporter activity	GO:0006855//drug transmembrane transport	gi|115397281|ref|XP_001214232.1|/0/conserved hypothetical protein [Aspergillus terreus NIH2624]

4316404	1203	515.71	720.19	1.089029717	1.98E-06	1.32E-05	Up	ATEG_01708	hypothetical protein	-	-	-	-	gi|115385052|ref|XP_001209073.1|/2.17365e-139/predicted protein [Aspergillus terreus NIH2624]
4322556	5929	3481.21	4768.39	1.08471277	3.20E-19	6.21E-18	Up	ATEG_07618	similar to pyridoxamine 5'-phosphate oxidase	ko01100//Metabolic pathways;ko00750//Vitamin B6 metabolism	-	GO:0004733//pyridoxamine-phosphate oxidase activity;GO:0010181//FMN binding	GO:0008615//pyridoxine biosynthetic process;GO:0042823//pyridoxal phosphate biosynthetic process;GO:0055114//oxidation-reduction process	gi|115401302|ref|XP_001216239.1|/0/hypothetical protein ATEG_07618 [Aspergillus terreus NIH2624]
4320426	1806	410.32	565.05	1.084594305	4.01E-07	2.95E-06	Up	ATEG_04263	hypothetical protein	-	GO:0000324//fungal-type vacuole;GO:0005829//cytosol	GO:0016787//hydrolase activity	GO:0009166//nucleotide catabolic process	gi|115394860|ref|XP_001213441.1|/0/conserved hypothetical protein [Aspergillus terreus NIH2624]
4321057	1827	139.54	194.15	1.082466881	0.00031823	0.0014637	Up	ATEG_05364	hypothetical protein	ko00970//Aminoacyl-tRNA biosynthesis	GO:0005737//cytoplasm	GO:0003723//RNA binding;GO:0004831//tyrosine-tRNA ligase activity;GO:0005524//ATP binding	GO:0006437//tyrosyl-tRNA aminoacylation	gi|115397901|ref|XP_001214542.1|/0/conserved hypothetical protein [Aspergillus terreus NIH2624]
4320610	1645	982.93	1343.62	1.082188601	6.62E-12	7.86E-11	Up	ATEG_05168	hypothetical protein	ko01100//Metabolic pathways;ko00040//Pentose and glucuronate interconversions	GO:0005634//nucleus;GO:0005829//cytosol	GO:0016279//protein-lysine N-methyltransferase activity	GO:0018027//peptidyl-lysine dimethylation;GO:0045905//positive regulation of translational termination	gi|115397509|ref|XP_001214346.1|/0/conserved hypothetical protein [Aspergillus terreus NIH2624]
4354132	1509	296.5	405.24	1.082029816	0.00017961	0.00086334	Up	ATEG_09536	hypothetical protein	-	GO:0016021//integral component of membrane	-	GO:0055085//transmembrane transport	gi|115442702|ref|XP_001218158.1|/0/conserved hypothetical protein [Aspergillus terreus NIH2624]

4318978	450	924.77	1269.03	1.082029467	1.04E-11	1.21E-10	Up	ATEG_07201	eukaryotic translation initiation factor 1A	ko03013//RNA transport	GO:0005634//nucleus;GO:0005829//cytosol	GO:0003743//translation initiation factor activity;GO:0033592//RNA strand annealing activity	GO:0006413//translational initiation	gi|115386692|ref|XP_001209887.1|/7.46032e-107/eukaryotic translation initiation factor 1A [Aspergillus terreus NIH2624]
4353639	2646	333.97	458.48	1.081305385	5.85E-07	4.19E-06	Up	ATEG_09416	hypothetical protein	ko04111//Cell cycle - yeast	GO:0016021//integral component of membrane	GO:0000978//RNA polymerase II core promoter proximal region sequence-specific DNA binding;GO:0001077//transcriptional activator activity, RNA polymerase II core promoter proximal region sequence-specific binding	GO:0000122//negative regulation of transcription from RNA polymerase II promoter;GO:0045944//positive regulation of transcription from RNA polymerase II promoter;GO:0055085//transmembrane transport	gi|115438328|ref|XP_001218038.1|/0/conserved hypothetical protein [Aspergillus terreus NIH2624]
4319575	2337	80.7	112.69	1.079954975	0.00480585	0.01652373	Up	ATEG_10141	hypothetical protein	ko01100//Metabolic pathways;ko01110//Biosynthesis of secondary metabolites;ko01130//Biosynthesis of antibiotics;ko00230//Purine metabolism	GO:0016021//integral component of membrane	-	GO:0055085//transmembrane transport	gi|115385793|ref|XP_001209443.1|/0/conserved hypothetical protein [Aspergillus terreus NIH2624]
4353981	5655	136.13	191.26	1.078240238	0.00141379	0.00558051	Up	ATEG_09311	hypothetical protein	-	GO:0016021//integral component of membrane	GO:0005488;GO:0008324//cation transmembrane transporter activity	GO:0098655//cation transmembrane transport	gi|115437918|ref|XP_001217933.1|/0/predicted protein [Aspergillus terreus NIH2624]
4353610	1185	539.04	743.46	1.078010159	7.03E-08	5.65E-07	Up	ATEG_09211	hypothetical protein	ko01212//Fatty acid metabolism;ko01040//Biosynthesis of unsaturated fatty acids	GO:0016021//integral component of membrane	-	GO:0006629//lipid metabolic process	gi|115437526|ref|XP_001217833.1|/0/conserved hypothetical protein [Aspergillus terreus NIH2624]

4318860	3550	468.46	636.74	1.073002255	2.01E-07	1.54E-06	Up	ATEG_06856	hypothetical protein	ko03022//Basal transcription factors	-	-	-	gi|115386002|ref|XP_001209541.1|/0/predicted protein [Aspergillus terreus NIH2624]
4322940	3983	692.77	938.54	1.072505431	1.57E-07	1.21E-06	Up	ATEG_07790	similar to endoglucanase B	ko01100//Metabolic pathways;ko00500//Starch and sucrose metabolism	GO:0005576//extracellular region	GO:0008810//cellulase activity;GO:0030248//cellulose binding	GO:0030245//cellulose catabolic process	gi|115401646|ref|XP_001216411.1|/0/hypothetical protein ATEG_07790 [Aspergillus terreus NIH2624]
4320831	3332	398.46	547.36	1.072408782	3.98E-07	2.93E-06	Up	ATEG_05021	hypothetical protein	ko01100//Metabolic pathways;ko00500//Starch and sucrose metabolism	-	GO:0004733//pyridoxamine-phosphate oxidase activity;GO:0010181//FMN binding	GO:0042823//pyridoxal phosphate biosynthetic process;GO:0055114//oxidation-reduction process	gi|115397215|ref|XP_001214199.1|/0/conserved hypothetical protein [Aspergillus terreus NIH2624]
4320712	1902	419.28	571.35	1.070124378	2.94E-07	2.20E-06	Up	ATEG_05327	lysophospholipase precursor	ko00564//Glycerophospholipid metabolism	-	GO:0004622//lysophospholipase activity	GO:0009395//phospholipid catabolic process	gi|115397827|ref|XP_001214505.1|/0/lysophospholipase precursor [Aspergillus terreus NIH2624]
4355193	5020	2542.97	3438.33	1.069467689	8.02E-13	1.02E-11	Up	ATEG_00439	similar to veA	ko04144//Endocytosis	GO:0005634//nucleus;GO:0005737//cytoplasm	-	GO:0006355//regulation of transcription, DNA-templated;GO:0030435//sporulation resulting in formation of a cellular spore;GO:0033246	gi|115491795|ref|XP_001210525.1|/0/hypothetical protein ATEG_00439 [Aspergillus terreus NIH2624]
4316807	1520	360.48	489.82	1.069328513	2.02E-05	0.00011522	Up	ATEG_02396	hypothetical protein	-	GO:0016021//integral component of membrane	GO:0005215//transporter activity	GO:0055085//transmembrane transport	gi|115388137|ref|XP_001211574.1|/0/predicted protein [Aspergillus terreus NIH2624]

4322269	3033	725.78	984.92	1.068829227	2.08E-10	2.15E-09	Up	ATEG_06522	hypothetical protein	-	GO:0016021//integral component of membrane	-	-	gi|115400223|ref|XP_001215700.1|/0/conserved hypothetical protein [Aspergillus terreus NIH2624]
4354880	1803	451.58	613.42	1.067555539	2.49E-07	1.88E-06	Up	ATEG_00123	beta-hexosaminidase precursor	ko01100//Metabolic pathways;ko00520//Amino sugar and nucleotide sugar metabolism;ko00511//Other glycan degradation;ko00603//Glycosphingolipid biosynthesis - globo series;ko00531//Glycosaminoglycan degradation	GO:0005576//extracellular region	GO:0016231//beta-N-acetylglucosaminidase activity	GO:0005975//carbohydrate metabolic process;GO:0006046//N-acetylglucosamine catabolic process	gi|115491163|ref|XP_001210209.1|/0/beta-hexosaminidase precursor [Aspergillus terreus NIH2624]
4321026	4674	302.65	413.61	1.064215981	3.81E-06	2.44E-05	Up	ATEG_05285	hypothetical protein	ko01100//Metabolic pathways;ko00500//Starch and sucrose metabolism	-	GO:0003677//DNA binding	-	gi|115397743|ref|XP_001214463.1|/0/predicted protein [Aspergillus terreus NIH2624]
4322280	1131	1438.96	1951.73	1.063719939	4.93E-13	6.34E-12	Up	ATEG_06331	hypothetical protein	-	-	GO:0051213//dioxygenase activity	GO:0055114//oxidation-reduction process	gi|115399840|ref|XP_001215509.1|/0/conserved hypothetical protein [Aspergillus terreus NIH2624]
4322669	1650	100.33	137.59	1.063553746	0.00368495	0.01307321	Up	ATEG_07808	hypothetical protein	-	GO:0009277//fungal-type cell wall	GO:0005199//structural constituent of cell wall	-	gi|115401682|ref|XP_001216429.1|/1.38354e-79/conserved hypothetical protein [Aspergillus terreus NIH2624]

4321478	1071	121.6	168.53	1.062607562	0.0014661	0.00577032	Up	ATEG_06209	hypothetical protein	ko01100//Metabolic pathways;ko00790//Folate biosynthesis	-	GO:0008270//zinc ion binding;GO:0016491//oxidoreductase activity	GO:0055114//oxidation-reduction process	gi|115399618|ref|XP_001215387.1|/0/predicted protein [Aspergillus terreus NIH2624]
4317255	1713	757.13	1026.99	1.061168033	3.95E-10	3.95E-09	Up	ATEG_02667	hypothetical protein	-	GO:0016021//integral component of membrane	GO:0005215//transporter activity	GO:0055085//transmembrane transport	gi|115388679|ref|XP_001211845.1|/0/predicted protein [Aspergillus terreus NIH2624]
4316628	1815	312.11	422.07	1.061107977	3.77E-06	2.41E-05	Up	ATEG_02329	G2/mitotic-specific cyclin cdc13	-	GO:0005634//nucleus	-	-	gi|[illegible]|ref|XP_001211507.1|/0/G2/mitotic-specific cyclin cdc13 [Aspergillus terreus NIH2624]
4355429	1738	73.38	102.28	1.060751692	0.0094803	0.02951349	Up	ATEG_00674	similar to tRNA guanylyltransferase	-	GO:0005829//cytosol	GO:0000287//magnesium ion binding;GO:0005525//GTP binding;GO:0008193//tRNA guanylyltransferase activity	GO:0006400//tRNA modification	gi|115492265|ref|XP_001210760.1|/0/hypothetical protein ATEG_00674 [Aspergillus terreus NIH2624]
4353260	699	73.39	101.39	1.059466527	0.00999933	0.03087323	Up	ATEG_08022	similar to chitinase 3	ko01100//Metabolic pathways;ko00520//Amino sugar and nucleotide sugar metabolism	-	GO:0004568//chitinase activity	GO:0005975//carbohydrate metabolic process	gi|115433012|ref|XP_001216643.1|/1.60161e-169/hypothetical protein ATEG_08022 [Aspergillus terreus NIH2624]
4320068	1341	651.64	876.43	1.058015365	1.99E-08	1.68E-07	Up	ATEG_04369	hypothetical protein	ko01100//Metabolic pathways;ko00500//Starch and sucrose metabolism	GO:0000324//fungal-type vacuole;GO:0016021//integral component of membrane	GO:0004888//transmembrane signaling receptor activity	GO:0007166//cell surface receptor signaling pathway;GO:0009405//pathogenesis	gi|115395816|ref|XP_001213547.1|/0/conserved hypothetical protein [Aspergillus terreus NIH2624]
4353247	1215	109.85	150.27	1.056114735	0.00154422	0.0060549	Up	ATEG_08213	hypothetical protein	ko01100//Metabolic pathways;ko00520//Amino sugar and nucleotide sugar metabolism	GO:0016021//integral component of membrane	GO:0008454//alpha-1,3-mannosylglycoprotein 4-beta-N-acetylglucosaminyltransferase activity	GO:0006491//N-glycan processing	gi|115433394|ref|XP_001216834.1|/0/conserved hypothetical protein [Aspergillus terreus NIH2624]

4320195	1962	147.88	205.69	1.054265579	0.00323394	0.01164392	Up	ATEG_04383	poly polymerase (Poly[ADP-ribose] synthetase)	ko03410//Base excision repair	-	GO:0003950//NAD+ ADP-ribosyltransferase activity	GO:0006471//protein ADP-ribosylation	gi|115395844|ref|XP_001213561.1|/0/poly polymerase (Poly[ADP-ribose] synthetase) [Aspergillus terreus NIH2624]
4353673	1401	1575.93	2124.88	1.053666239	9.89E-13	1.25E-11	Up	ATEG_09478	D-3-phosphoglycerate dehydrogenase 2	ko01100//Metabolic pathways;ko01130//Biosynthesis of antibiotics;ko01230//Biosynthesis of amino acids;ko01200//Carbon metabolism;ko00260//Glycine, serine and threonine metabolism;ko00680//Methane metabolism	-	GO:0004617//phosphoglycerate dehydrogenase activity;GO:0016597//amino acid binding;GO:0051287//NAD binding	GO:0006564//L-serine biosynthetic process;GO:0055114//oxidation-reduction process	gi|115438570|ref|XP_001218100.1|/0/D-3-phosphoglycerate dehydrogenase 2 [Aspergillus terreus NIH2624]
4354329	1173	7621.6	10223.09	1.053140432	1.08E-15	1.63E-14	Up	ATEG_09555	60S ribosomal protein L23	ko03010//Ribosome	GO:0005840//ribosome	GO:0003735//structural constituent of ribosome;GO:0008233//peptidase activity	GO:0006412//translation;GO:0006508//proteolysis	gi|115442740|ref|XP_001218177.1|/1.08551e-97/60S ribosomal protein L23 [Aspergillus terreus NIH2624]
4353460	1938	164.28	224.55	1.052547701	0.00102998	0.00420497	Up	ATEG_08260	hypothetical protein	-	GO:0016021//integral component of membrane	-	-	gi|115433488|ref|XP_001216881.1|/0/predicted protein [Aspergillus terreus NIH2624]

4315748	2639	325.01	439.37	1.052205669	1.52E-06	1.03E-05	Up	ATEG_01428	hypothetical protein	ko01100//Metabolic pathways;ko01110//Biosynthesis of secondary metabolites;ko01130//Biosynthesis of antibiotics;ko01230//Biosynthesis of amino acids;ko01210//2-Oxocarboxylic acid metabolism;ko00220//Arginine biosynthesis	GO:0005759//mitochondrial matrix	GO:0004042//acetyl-CoA:L-glutamate N-acetyltransferase activity	GO:0006526//arginine biosynthetic process;GO:0006592//ornithine biosynthetic process	gi|115384492|ref|XP_001208793.1|/0/conserved hypothetical protein [Aspergillus terreus NIH2624]
4355335	4746	1334.41	1803.1	1.051225253	4.28E-10	4.26E-09	Up	ATEG_00581	similar to arom polypeptide	ko01100//Metabolic pathways;ko01110//Biosynthesis of secondary metabolites;ko01130//Biosynthesis of antibiotics;ko01230//Biosynthesis of amino acids;ko00400//Phenylalanine, tyrosine and tryptophan biosynthesis	GO:0005829//cytosol	GO:0003855//3-dehydroquinate dehydratase activity;GO:0003856//3-dehydroquinate synthase activity;GO:0003866//3-phosphoshikimate 1-carboxyvinyltransferase activity;GO:0004764//shikimate 3-dehydrogenase (NADP+) activity;GO:0004765//shikimate kinase activity;GO:0005524//ATP binding;GO:0046872//metal ion binding	GO:0009073//aromatic amino acid family biosynthetic process;GO:0009423//chorismate biosynthetic process;GO:0016310//phosphorylation;GO:0055114//oxidation-reduction process	gi|115492079|ref|XP_001210667.1|/0/hypothetical protein ATEG_00581 [Aspergillus terreus NIH2624]

4354278	1443	984.36	1314.73	1.050702636	8.17E-08	6.53E-07	Up	ATEG_09808	hypothetical protein	ko04111//Cell cycle - yeast	GO:0016021//integral component of membrane	-	-	gi|115443246|ref|XP_001218430.1|/0/conserved hypothetical protein [Aspergillus terreus NIH2624]
4319867	2112	910.4	1221.02	1.049919646	2.04E-08	1.73E-07	Up	ATEG_04575	hypothetical protein	ko04141//Protein processing in endoplasmic reticulum	GO:0016021//integral component of membrane	-	-	gi|115396228|ref|XP_001213753.1|/0/predicted protein [Aspergillus terreus NIH2624]
4319556	2352	212.34	286.06	1.049401289	0.00010169	0.00050859	Up	ATEG_10073	hypothetical protein	-	GO:0016021//integral component of membrane	-	-	gi|115385657|ref|XP_001209375.1|/0/predicted protein [Aspergillus terreus NIH2624]
4320080	810	2546.64	3394.5	1.048976071	3.16E-12	3.83E-11	Up	ATEG_04695	nonhistone chromosomal protein 6A	-	-	-	-	gi|115396468|ref|XP_001213873.1|/5.79308e-70/nonhistone chromosomal protein 6A [Aspergillus terreus NIH2624]
4319889	1648	813.72	1092.63	1.04745906	2.37E-10	2.42E-09	Up	ATEG_04333	type II proteins geranylgeranyltransferase beta subunit	-	-	GO:0004663//Rab geranylgeranyltransferase activity	GO:0018344//protein geranylgeranylation	gi|115395496|ref|XP_001213511.1|/0/type II proteins geranylgeranyltransferase beta subunit [Aspergillus terreus NIH2624]
4315685	468	191.08	256.79	1.04661084	0.00013323	0.00065266	Up	ATEG_01103	hypothetical protein	-	GO:0005634//nucleus;GO:0005829//cytosol	-	-	gi|115383842|ref|XP_001208468.1|/1.73359e-108/conserved hypothetical protein [Aspergillus terreus NIH2624]

4318438	4197	334.58	448.41	1.045941568	1.14E-06	7.75E-06	Up	ATEG_03812	similar to thermotolerance protein	-	GO:0005634//nucleus	GO:0003676//nucleic acid binding	-	gi|115390971|ref|XP_001212990.1|/0/hypothetical protein ATEG_03812 [Aspergillus terreus NIH2624]
4318909	1371	346.95	466.45	1.044559451	2.11E-06	1.40E-05	Up	ATEG_06947	hypothetical protein	-	-	-	-	gi|115386184|ref|XP_001209633.1|/0/conserved hypothetical protein [Aspergillus terreus NIH2624]
4355414	2589	1569.96	2094.08	1.037974428	3.44E-11	3.81E-10	Up	ATEG_00659	histidinol dehydrogenase	ko01100//Metabolic pathways;ko01110//Biosynthesis of secondary metabolites;ko01230//Biosynthesis of amino acids;ko00340//Histidine metabolism	GO:0005634//nucleus;GO:0005829//cytosol	GO:0004399//histidinol dehydrogenase activity;GO:0004635//phosphoribosyl-AMP cyclohydrolase activity;GO:0004636//phosphoribosyl-ATP diphosphatase activity;GO:0008270//zinc ion binding;GO:0051287//NAD binding	GO:0000105//histidine biosynthetic process;GO:0055114//oxidation-reduction process	gi|115492235|ref|XP_001210745.1|/0/histidinol dehydrogenase [Aspergillus terreus NIH2624]
4320413	1140	1952.94	2585.93	1.037331904	1.06E-10	1.14E-09	Up	ATEG_04414	similar to purine cytosine permease Fcy2	ko01100//Metabolic pathways;ko00500//Starch and sucrose metabolism	GO:0016021//integral component of membrane	GO:0015205//nucleobase transmembrane transporter activity	GO:0006863//purine nucleobase transport;GO:0055085//transmembrane transport	gi|115395906|ref|XP_001213592.1|/0/hypothetical protein ATEG_04414 [Aspergillus terreus NIH2624]
4353741	1320	7927.79	10519.59	1.036590877	1.52E-18	2.76E-17	Up	ATEG_09467	60S ribosomal protein L25	ko03010//Ribosome	GO:0005840//ribosome	GO:0000166//nucleotide binding;GO:0003735//structural constituent of ribosome	GO:0006412//translation	gi|115438528|ref|XP_001218089.1|/3.06326e-110/60S ribosomal protein L25 [Aspergillus terreus NIH2624]

4315591	1740	2086.23	2747.81	1.036445602	1.37E-08	1.17E-07	Up	ATEG_01781	hypothetical protein	ko00380//Tryptophan metabolism;ko00360//Phenylalanine metabolism;ko00330//Arginine and proline metabolism	-	GO:0016884//carbon-nitrogen ligase activity, with glutamine as amido-N-donor	-	gi|115385198|ref|XP_001209146.1|/0/conserved hypothetical protein [Aspergillus terreus NIH2624]
4320605	2247	205.72	278.02	1.033905493	0.00038927	0.00175183	Up	ATEG_05126	hypothetical protein	-	GO:0005634//nucleus;GO:0005737//cytoplasm	GO:0003723//RNA binding;GO:0009982//pseudouridine synthase activity	GO:0000455//enzyme-directed rRNA pseudouridine synthesis;GO:0031119//tRNA pseudouridine synthesis;GO:0031120//snRNA pseudouridine synthesis;GO:1990481//mRNA pseudouridine synthesis	gi|115397425|ref|XP_001214304.1|/0/conserved hypothetical protein [Aspergillus terreus NIH2624]
4323023	1014	1162.32	1536.57	1.033834647	6.34E-12	7.54E-11	Up	ATEG_07706	hypothetical protein	ko04144//Endocytosis	-	-	GO:0044699;GO:0048518	gi|115401478|ref|XP_001216327.1|/0/conserved hypothetical protein [Aspergillus terreus NIH2624]
4355668	1719	673.14	891.22	1.0332696	6.14E-09	5.48E-08	Up	ATEG_00907	similar to dihydrosphingosine-1-phosphate lyase	ko01100//Metabolic pathways;ko00600//Sphingolipid metabolism	-	GO:0008117//sphinganine-1-phosphate aldolase activity;GO:0016831//carboxy-lyase activity;GO:0030170//pyridoxal phosphate binding	GO:0019752//carboxylic acid metabolic process	gi|115492731|ref|XP_001210993.1|/0/hypothetical protein ATEG_00907 [Aspergillus terreus NIH2624]
4321771	1062	240.34	319.26	1.032703096	0.00017451	0.00084058	Up	ATEG_05842	hypothetical protein	-	-	GO:0003824//catalytic activity;GO:0030151//molybdenum ion binding;GO:0030170//pyridoxal phosphate binding	-	gi|115398862|ref|XP_001215020.1|/0/conserved hypothetical protein [Aspergillus terreus NIH2624]

4320025	4335	810.12	1086.14	1.032214693	5.37E-06	3.34E-05	Up	ATEG_04609	hypothetical protein	-	GO:0005737//cytoplasm	GO:0003743//translation initiation factor activity	GO:0006413//translational initiation;GO:0048312//intracellular distribution of mitochondria	gi|115396296|ref|XP_001213787.1|/0/conserved hypothetical protein [Aspergillus terreus NIH2624]
4315919	2142	1361.22	1794.21	1.031533615	7.25E-12	8.57E-11	Up	ATEG_01662	similar to nitrogen regulatory protein OTam	-	GO:0005634//nucleus	GO:0000981//RNA polymerase II transcription factor activity, sequence-specific DNA binding;GO:0001128//RNA polymerase II transcription coactivator activity involved in preinitiation complex assembly;GO:0001135//transcription factor activity, RNA polymerase II transcription factor recruiting;GO:0003677//DNA binding;GO:0008270//zinc ion binding	GO:0001080//nitrogen catabolite activation of transcription from RNA polymerase II promoter;GO:0045944//positive regulation of transcription from RNA polymerase II promoter;GO:0051123//RNA polymerase II transcriptional preinitiation complex assembly;GO:1901714//positive regulation of urea catabolic process;GO:1901717//positive regulation of gamma-aminobutyric acid catabolic process	gi|115384960|ref|XP_001209027.1|/0/hypothetical protein ATEG_01662 [Aspergillus terreus NIH2624]
4321629	2955	405.68	542.8	1.031250402	3.11E-06	2.02E-05	Up	ATEG_05841	hypothetical protein	-	GO:0005634//nucleus;GO:0005829//cytosol	GO:0000182//rDNA binding;GO:0003887//DNA-directed DNA polymerase activity	GO:0042790//transcription of nuclear large rRNA transcript from RNA polymerase I promoter;GO:0071897//DNA biosynthetic process	gi|115398860|ref|XP_001215019.1|/0/conserved hypothetical protein [Aspergillus terreus NIH2624]

4318065	648	2935.27	3882.17	1.030116793	8.54E-13	1.08E-11	Up	ATEG_03007	60S ribosomal protein L27	ko03010//Ribosome	GO:0005840//ribosome;GO:0016021//integral component of membrane	GO:0003735//structural constituent of ribosome	GO:0006412//translation	gi|115389360|ref|XP_001212185.1|/5.46656e-104/60S ribosomal protein L27 [Aspergillus terreus NIH2624]
4316175	1395	124.44	166.95	1.030006135	0.00163137	0.00635678	Up	ATEG_01163	hypothetical protein	-	GO:0016021//integral component of membrane	-	-	gi|115383962|ref|XP_001208528.1|/0/conserved hypothetical protein [Aspergillus terreus NIH2624]
4323337	2358	372.61	491.69	1.028878916	5.59E-06	3.46E-05	Up	ATEG_08747	similar to xylhp	ko04113//Meiosis - yeast	GO:0016021//integral component of membrane	GO:0022891//substrate-specific transmembrane transporter activity	GO:0008643//carbohydrate transport;GO:0055085//transmembrane transport	gi|115402513|ref|XP_001217333.1|/0/hypothetical protein ATEG_08747 [Aspergillus terreus NIH2624]
4323207	2979	1179.46	1556.29	1.028182181	5.89E-09	5.26E-08	Up	ATEG_08610	hypothetical protein	-	-	GO:0003824//catalytic activity;GO:0030246//carbohydrate binding	GO:0005975//carbohydrate metabolic process	gi|115402239|ref|XP_001217196.1|/0/predicted protein [Aspergillus terreus NIH2624]
4320919	1275	811.81	1083.6	1.027359251	5.00E-07	3.62E-06	Up	ATEG_05239	adenylosuccinate synthetase	ko01100//Metabolic pathways;ko00230//Purine metabolism;ko00250//Alanine, aspartate and glutamate metabolism	GO:0005634//nucleus;GO:0005829//cytosol	GO:0000287//magnesium ion binding;GO:0004019//adenylosuccinate synthase activity;GO:0005525//GTP binding;GO:0061483//sulfinylpropanyl adenylate synthase	GO:0006106//fumarate metabolic process;GO:0044208//'de novo' AMP biosynthetic process;GO:0071276//cellular response to cadmium ion	gi|115397651|ref|XP_001214417.1|/0/adenylosuccinate synthetase [Aspergillus terreus NIH2624]
4316560	1842	112.59	152.57	1.027240737	0.00450706	0.01562814	Up	ATEG_01979	hypothetical protein	ko04113//Meiosis - yeast	GO:0016021//integral component of membrane	GO:0022891//substrate-specific transmembrane transporter activity	GO:0055085//transmembrane transport	gi|115387303|ref|XP_001211157.1|/0/conserved hypothetical protein [Aspergillus terreus NIH2624]

4355674	5047	540.92	711.83	1.02680729	9.59E-07	6.60E-06	Up	ATEG_00911	hypothetical protein	ko01100//Metabolic pathways;ko00561//Glycerolipid metabolism	-	GO:0004806//triglyceride lipase activity	GO:0016042//lipid catabolic process	gi|115492739|ref|XP_001210997.1|/0/predicted protein [Aspergillus terreus NIH2624]
4321722	855	642.05	842.38	1.026138079	1.01E-05	6.01E-05	Up	ATEG_06079	hypothetical protein	ko01100//Metabolic pathways;ko01110//Biosynthesis of secondary metabolites;ko01130//Biosynthesis of antibiotics;ko01230//Biosynthesis of amino acids;ko00330//Arginine and proline metabolism	-	GO:0004735//pyrroline-5-carboxylate reductase activity	GO:0055114//oxidation-reduction process;GO:0055129//L-proline biosynthetic process	gi|115399336|ref|XP_001215257.1|/0/conserved hypothetical protein [Aspergillus terreus NIH2624]
4316186	2851	373.79	494.99	1.025727885	6.94E-07	4.91E-06	Up	ATEG_01061	similar to KLPA	-	GO:0000235//astral microtubule;GO:0000777//condensed chromosome kinetochore;GO:0005634//nucleus;GO:0035371//microtubule plus-end;GO:0036449//microtubule minus-end;GO:0055028//cortical microtubule	GO:0005524//ATP binding;GO:0008017//microtubule binding;GO:0008569//ATP-dependent microtubule motor activity, minus-end-directed	GO:0000022//mitotic spindle elongation;GO:0000742//karyogamy involved in conjugation with cellular fusion;GO:0030951//establishment or maintenance of microtubule cytoskeleton polarity;GO:0031122//cytoplasmic microtubule organization;GO:0031534//minus-end directed microtubule sliding;GO:0090561//nuclear migration during mitotic telophase	gi|115383758|ref|XP_001208426.1|/0/hypothetical protein ATEG_01061 [Aspergillus terreus NIH2624]

4317986	855	103.13	137.43	1.025677914	0.00565431	0.01896833	Up	ATEG_03512	hypothetical protein	ko04146//Peroxisome	-	GO:0016853//isomerase activity	GO:0008152//metabolic process	gi|115390370|ref|XP_001212690.1|/0/conserved hypothetical protein [Aspergillus terreus NIH2624]
4321002	4964	457.81	602.8	1.025439718	3.09E-07	2.31E-06	Up	ATEG_05048	similar to possible kinase with calcium binding domain	ko04140//Regulation of autophagy	-	GO:0004672//protein kinase activity;GO:0005524//ATP binding	GO:0006468//protein phosphorylation	gi|115397269|ref|XP_001214226.1|/0/hypothetical protein ATEG_05048 [Aspergillus terreus NIH2624]
4319291	2669	755.27	995.65	1.024743838	5.78E-09	5.18E-08	Up	ATEG_07327	hypothetical protein	ko00620//Pyruvate metabolism	-	GO:0016884//carbon-nitrogen ligase activity, with glutamine as amido-N-donor	-	gi|115386944|ref|XP_001210013.1|/0/conserved hypothetical protein [Aspergillus terreus NIH2624]
4353755	1722	3295.97	4326.58	1.023517843	2.83E-16	4.47E-15	Up	ATEG_09464	eukaryotic peptide chain release factor subunit 1	ko03015//mRNA surveillance pathway	GO:0005634//nucleus;GO:0005829//cytosol;GO:0010494//cytoplasmic stress granule;GO:0018444//translation release factor complex	GO:0016149//translation release factor activity, codon specific	GO:0002184//cytoplasmic translational termination;GO:0006353//DNA-templated transcription, termination	gi|115438516|ref|XP_001218086.1|/0/eukaryotic peptide chain release factor subunit 1 [Aspergillus terreus NIH2624]
4355090	731	1359	1777.08	1.02170542	1.37E-10	1.44E-09	Up	ATEG_00338	hypothetical protein	-	GO:0016021//integral component of membrane	-	-	gi|115491593|ref|XP_001210424.1|/7.28633e-131/predicted protein [Aspergillus terreus NIH2624]
4315722	1401	510.52	668.66	1.017984013	8.30E-08	6.62E-07	Up	ATEG_01391	hypothetical protein	-	GO:0016021//integral component of membrane	-	-	gi|115384418|ref|XP_001208756.1|/0/conserved hypothetical protein [Aspergillus terreus NIH2624]

4318434	4892	13868.58	18131.24	1.017230725	1.91E-20	4.00E-19	Up	ATEG_03808	hypothetical protein	-	GO:0000120//RNA polymerase I transcription factor complex;GO:0005829//cytosol	GO:0003743//translation initiation factor activity	GO:0006360//transcription from RNA polymerase I promoter;GO:0006413//translational initiation	gi|115390963|ref|XP_001212986.1|/0/predicted protein [Aspergillus terreus NIH2624]
4354007	1119	7276.82	9503.25	1.015052332	3.66E-16	5.74E-15	Up	ATEG_09279	60S ribosomal protein L4	ko03010//Ribosome	GO:0005840//ribosome	GO:0003735//structural constituent of ribosome	GO:0006412//translation	gi|115437794|ref|XP_001217901.1|/0/60S ribosomal protein L4 [Aspergillus terreus NIH2624]
4317491	3862	1860.26	2427.56	1.014290605	1.23E-12	1.55E-11	Up	ATEG_02908	hypothetical protein	ko04144//Endocytosis	-	-	-	gi|115389162|ref|XP_001212086.1|/0/predicted protein [Aspergillus terreus NIH2624]
4316317	684	180.54	240.88	1.011300117	0.0019893	0.00760931	Up	ATEG_01877	hypothetical protein	-	-	-	GO:0043461//proton-transporting ATP synthase complex assembly	gi|115385390|ref|XP_001209242.1|/8.89114e-160/conserved hypothetical protein [Aspergillus terreus NIH2624]
4315782	1344	878.48	1153.32	1.010987463	4.83E-07	3.51E-06	Up	ATEG_01053	hypothetical protein	ko01100//Metabolic pathways;ko00760//Nicotinate and nicotinamide metabolism	GO:0016020//membrane	GO:0052861//glucan endo-1,3-beta-glucanase activity, C-3 substituted reducing group	GO:0005975//carbohydrate metabolic process	gi|115383742|ref|XP_001208418.1|/0/predicted protein [Aspergillus terreus NIH2624]
4320929	3404	2761.34	3600.07	1.010260368	1.12E-14	1.57E-13	Up	ATEG_05464	hypothetical protein	-	-	-	-	gi|115398101|ref|XP_001214642.1|/0/conserved hypothetical protein [Aspergillus terreus NIH2624]
4321091	1371	254.41	332.57	1.008978401	3.03E-05	0.00016795	Up	ATEG_05211	hypothetical protein	ko01100//Metabolic pathways;ko00240//Pyrimidine metabolism	-	GO:0004590//orotidine-5'-phosphate decarboxylase activity	GO:0006207//'de novo' pyrimidine nucleobase biosynthetic process	gi|115397595|ref|XP_001214389.1|/0/conserved hypothetical protein [Aspergillus terreus NIH2624]

4316063	1452	651.71	848.96	1.005488612	6.31E-09	5.62E-08	Up	ATEG_01133	adenylosuccinate lyase	ko01100//Metabolic pathways;ko01110//Biosynthesis of secondary metabolites;ko01130//Biosynthesis of antibiotics;ko00230//Purine metabolism;ko00250//Alanine, aspartate and glutamate metabolism	-	GO:0004018//N6-(1,2-dicarboxyethyl)AMP AMP-lyase (fumarate-forming) activity;GO:0070626//(S)-2-(5-amino-1-(5-phospho-D-ribosyl)imidazole-4-carboxamido)succinate AMP-lyase (fumarate-forming) activity	GO:0006189//'de novo' IMP biosynthetic process;GO:0044208//'de novo' AMP biosynthetic process	gi|115383902|ref|XP_001208498.1|/0/adenylosuccinate lyase [Aspergillus terreus NIH2624]
4322824	3614	294.24	385.44	1.00514726	1.51E-05	8.75E-05	Up	ATEG_07641	hypothetical protein	-	GO:0005730//nucleolus;GO:0005829//cytosol	-	GO:0000463//maturation of LSU-rRNA from tricistronic rRNA transcript (SSU-rRNA, 5.8S rRNA, LSU-rRNA);GO:0000466//maturation of 5.8S rRNA from tricistronic rRNA transcript (SSU-rRNA, 5.8S rRNA, LSU-rRNA)	gi|115401348|ref|XP_001216262.1|/0/conserved hypothetical protein [Aspergillus terreus NIH2624]
4318193	1503	461.26	602.8	1.004979925	5.36E-07	3.87E-06	Up	ATEG_03699	hypothetical protein	ko01100//Metabolic pathways;ko00380//Tryptophan metabolism	GO:0005741//mitochondrial outer membrane	GO:0004502//kynurenine 3-monooxygenase activity;GO:0071949//FAD binding	GO:0006569//tryptophan catabolic process;GO:0019805//quinolinate biosynthetic process;GO:0034354//'de novo' NAD biosynthetic process from tryptophan;GO:0043420//anthranilate metabolic process;GO:0055114//oxidation-reduction process	gi|115390745|ref|XP_001212877.1|/0/conserved hypothetical protein [Aspergillus terreus NIH2624]

4315732	5670	177.16	231.96	1.00271412	0.00101297	0.00413788	Up	ATEG_01734	hypothetical protein	-	-	-	-	gi|115385104|ref|XP_001209099.1|/0/predicted protein [Aspergillus terreus NIH2624]
4317135	1125	462.35	596.51	1.002677442	2.96E-05	0.00016448	Up	ATEG_02553	hypothetical protein	-	-	GO:0003824//catalytic activity;GO:0050662//coenzyme binding	-	gi|115388451|ref|XP_001211731.1|/0/conserved hypothetical protein [Aspergillus terreus NIH2624]
4322910	681	558.11	721.18	1.002489289	8.64E-07	6.00E-06	Up	ATEG_07406	hypothetical protein	ko04011//MAPK signaling pathway - yeast	-	-	-	gi|115400878|ref|XP_001216027.1|/1.00903e-169/predicted protein [Aspergillus terreus NIH2624]
4317092	2187	394.58	510.6	1.001906079	6.74E-06	4.11E-05	Up	ATEG_02138	hypothetical protein	ko01100//Metabolic pathways;ko00500//Starch and sucrose metabolism	GO:0016021//integral component of membrane	-	-	gi|115387621|ref|XP_001211316.1|/0/conserved hypothetical protein [Aspergillus terreus NIH2624]
4316400	1176	102.57	134.6	1.000761425	0.00523417	0.01776853	Up	ATEG_01704	hypothetical protein	ko01100//Metabolic pathways;ko00500//Starch and sucrose metabolism;ko00040//Pentose and glucuronate interconversions	GO:0005576//extracellular region;GO:0005618//cell wall	GO:0030599//pectinesterase activity;GO:0045330//aspartyl esterase activity	GO:0042545//cell wall modification;GO:0045490//pectin catabolic process	gi|115385044|ref|XP_001209069.1|/0/conserved hypothetical protein [Aspergillus terreus NIH2624]
4353736	1263	3086.17	0	-10.18175406	3.57E-52	2.54E-50	Down	ATEG_09398	hypothetical protein	-	GO:0005737//cytoplasm	-	-	gi|115438256|ref|XP_001218020.1|/0/conserved hypothetical protein [Aspergillus terreus NIH2624]

4317957	2175	561.54	0	-7.990904022	2.48E-27	7.57E-26	Down	ATEG_03541	hypothetical protein	ko01100//Metabolic pathways;ko00500//Starch and sucrose metabolism	-	GO:0008236//serine-type peptidase activity (protein degradation)	GO:0006508//proteolysis	gi|115390428|ref|XP_001212719.1|/0/predicted protein [Aspergillus terreus NIH2624]
4322056	1008	420.43	0	-7.592257362	8.86E-24	2.21E-22	Down	ATEG_06421	similar to CipA protein	ko01100//Metabolic pathways;ko01110//Biosynthesis of secondary metabolites;ko01230//Biosynthesis of amino acids;ko00260//Glycine, serine and threonine metabolism;ko00400//Phenylalanine, tyrosine and tryptophan biosynthesis	-	-	-	gi|115400021|ref|XP_001215599.1|/0/hypothetical protein ATEG_06421 [Aspergillus terreus NIH2624]
4353549	921	399.95	0	-7.510726943	5.00E-23	1.21E-21	Down	ATEG_08127	hypothetical protein	ko01100//Metabolic pathways;ko00600//Sphingolipid metabolism	-	GO:0016491//oxidoreductase activity	GO:0008152//metabolic process	gi|115433222|ref|XP_001216748.1|/0/conserved hypothetical protein [Aspergillus terreus NIH2624]
4323427	1098	369.68	0	-7.398303352	4.26E-22	9.84E-21	Down	ATEG_08553	hypothetical protein	ko04144//Endocytosis	GO:0016021//integral component of membrane	-	GO:0055085//transmembrane transport	gi|115402125|ref|XP_001217139.1|/0/predicted protein [Aspergillus terreus NIH2624]
4353224	1152	337.41	0	-7.315520593	1.23E-21	2.77E-20	Down	ATEG_08189	protein MNN9	ko01100//Metabolic pathways;ko00513//Various types of N-glycan biosynthesis	GO:0000136//alpha-1,6-mannosyltransferase complex;GO:0005783//endoplasmic reticulum;GO:0005801//cis-Golgi network	GO:0000009//alpha-1,6-mannosyltransferase activity (formation of mannose glycoside of a protein/lipid)	GO:0006487//protein N-linked glycosylation;GO:0070317//negative regulation of G0 to G1 transition;GO:0097502//mannosylation	gi|115433346|ref|XP_001216810.1|/0/protein MNN9 [Aspergillus terreus NIH2624]

4319294	1422	342.58	0	-7.291674084	2.91E-21	6.44E-20	Down	ATEG_07330	hypothetical protein	-	-	GO:0016491//oxidoreductase activity	GO:0055114//oxidation-reduction process	gi|115386950|ref|XP_001210016.1|/0/predicted protein [Aspergillus terreus NIH2624]
4323396	1815	328.8	0	-7.24801609	5.73E-21	1.24E-19	Down	ATEG_08622	hypothetical protein	-	GO:0005576//extracellular region	GO:0004519//endonuclease activity;GO:0004527//exonuclease activity (cleaving of nucleotide chain)	GO:0090305//nucleic acid phosphodiester bond hydrolysis	gi|115402263|ref|XP_001217208.1|/0/conserved hypothetical protein [Aspergillus terreus NIH2624]
4353120	1026	295.83	0	-7.135303014	3.02E-20	6.22E-19	Down	ATEG_08323	hypothetical protein	ko01100//Metabolic pathways;ko00500//Starch and sucrose metabolism	-	-	-	gi|115433614|ref|XP_001216944.1|/0/conserved hypothetical protein [Aspergillus terreus NIH2624]
4315881	2247	264.47	0	-6.988417588	3.40E-19	6.56E-18	Down	ATEG_01569	similar to exonuclease	ko03430//Mismatch repair	GO:0005634//nucleus	GO:0003677//DNA binding;GO:0035312//5'-3' exodeoxyribonuclease activity (degradation of nucleic acid?)	GO:0006281//DNA repair;GO:0090305//nucleic acid phosphodiester bond hydrolysis	gi|115384774|ref|XP_001208934.1|/0/hypothetical protein ATEG_01569 [Aspergillus terreus NIH2624]
4318986	1086	1323.74	5.35	-6.937753768	4.76E-61	4.42E-59	Down	ATEG_07376	hypothetical protein	-	-	GO:0003677//DNA binding	-	gi|115387042|ref|XP_001210062.1|/0/predicted protein [Aspergillus terreus NIH2624]
4320281	1353	974.03	4.13	-6.843390389	2.25E-51	1.58E-49	Down	ATEG_04792	hypothetical protein	ko03013//RNA transport;ko03015//mRNA surveillance pathway	-	-	-	gi|115396662|ref|XP_001213970.1|/0/conserved hypothetical protein [Aspergillus terreus NIH2624]

4321680	708	229.15	0	-6.79136062	8.18E-18	1.41E-16	Down	ATEG_05891	similar to peroxin-11	18.93-	GO:0005739//mitochondrion;GO:0005779//integral component of peroxisomal membrane;GO:0005783//endoplasmic reticulum;GO:1990429//peroxisomal importomer complex	-	GO:0016559//peroxisome fission;GO:0019395//fatty acid oxidation;GO:0044375//regulation of peroxisome size	gi|115398960|ref|XP_001215069.1|/2.27016e-166/hypothetical protein ATEG_05891 [Aspergillus terreus NIH2624]
4353840	1518	1986.97	9.87	-6.787014718	2.30E-84	3.73E-82	Down	ATEG_09137	similar to LAP1	-	GO:0005576//extracellular region	GO:0004177//aminopeptidase activity;GO:0008237//metallopeptidase activity;GO:0046872//metal ion binding (protein degradation, activated by Zn2+)	GO:0006508//proteolysis	gi|115437232|ref|XP_001217759.1|/0/hypothetical protein ATEG_09137 [Aspergillus terreus NIH2624]
4322610	1146	775.85	3.14	-6.757999377	1.68E-41	8.78E-40	Down	ATEG_07665	hypothetical protein	-	-	GO:0008270//zinc ion binding;GO:0016491//oxidoreductase activity	GO:0055114//oxidation-reduction process	gi|115401396|ref|XP_001216286.1|/0/conserved hypothetical protein [Aspergillus terreus NIH2624]
4318736	1299	227.06	0	-6.753183789	1.77E-17	2.99E-16	Down	ATEG_03838	similar to novel protein	ko03022//Basal transcription factors	-	GO:0003677//DNA binding;GO:0008270//zinc ion binding	-	gi|115391023|ref|XP_001213016.1|/0/hypothetical protein ATEG_03838 [Aspergillus terreus NIH2624]
4321503	819	227.75	0	-6.63635622	2.24E-16	3.58E-15	Down	ATEG_05787	hypothetical protein	-	-	-	-	gi|115398752|ref|XP_001214965.1|/0/conserved hypothetical protein [Aspergillus terreus NIH2624]

4321129	699	192.76	0	-6.561242079	2.53E-16	4.03E-15	Down	ATEG_04978	hypothetical protein	ko01100//Metabolic pathways;ko00564//Glycerophospholipid metabolism;ko00565//Ether lipid metabolism	-	GO:0016491//oxidoreductase activity	GO:0055114//oxidation-reduction process	gi|115397129|ref|XP_001214156.1|/7.03278e-164/conserved hypothetical protein [Aspergillus terreus NIH2624]
4320031	1806	188.37	0	-6.504435749	6.72E-16	1.03E-14	Down	ATEG_04680	similar to glutamyl-tRNA(Gln) amidotransferase subunit B	ko01100//Metabolic pathways;ko00970//Aminoacyl-tRNA biosynthesis	GO:0005739//mitochondrion;GO:0030956//glutamyl-tRNA(Gln) amidotransferase complex	GO:0005524//ATP binding;GO:0016740//transferase activity;GO:0050567//glutaminyl-tRNA synthase (glutamine-hydrolyzing) activity	GO:0032543//mitochondrial translation;GO:0070681//glutaminyl-tRNAGln biosynthesis via transamidation	gi|115396438|ref|XP_001213858.1|/0/hypothetical protein ATEG_04680 [Aspergillus terreus NIH2624]
4321521	960	181.59	0	-6.477984835	8.48E-16	1.30E-14	Down	ATEG_06059	similar to arabinan endo-1,5-alpha-L-arabinosidase A precursor	ko01100//Metabolic pathways;ko00520//Amino sugar and nucleotide sugar metabolism	-	GO:0004553//hydrolase activity, hydrolyzing O-glycosyl compounds (degradation of disaccharides)	GO:0045493//xylan catabolic process	gi|115399296|ref|XP_001215237.1|/0/hypothetical protein ATEG_06059 [Aspergillus terreus NIH2624]
4323015	1308	187.06	0	-6.471264036	1.27E-15	1.90E-14	Down	ATEG_07662	hypothetical protein	ko01100//Metabolic pathways;ko01220//Degradation of aromatic compounds	GO:0016021//integral component of membrane	GO:0004497//monooxygenase activity;GO:0071949//FAD binding	GO:0035690//cellular response to drug;GO:0055114//oxidation-reduction process;GO:1900554	gi|115401390|ref|XP_001216283.1|/0/conserved hypothetical protein [Aspergillus terreus NIH2624]
4355236	3636	193.52	0	-6.435850207	3.25E-15	4.74E-14	Down	ATEG_00482	hypothetical protein	-	-	-	-	gi|115491881|ref|XP_001210568.1|/0/conserved hypothetical protein [Aspergillus terreus NIH2624]
4316114	525	177.16	0	-6.415151599	2.44E-15	3.60E-14	Down	ATEG_01864	hypothetical protein	ko01100//Metabolic pathways;ko00520//Amino sugar and nucleotide sugar metabolism	-	-	-	gi|115385364|ref|XP_001209229.1|/1.00787e-123/predicted protein [Aspergillus terreus NIH2624]

4322739	1269	797.81	4.43	-6.401357717	1.11E-42	5.93E-41	Down	ATEG_07490	hypothetical protein	-	-	GO:0016491//oxidoreductase activity	GO:0055114//oxidation-reduction process	gi|115401046|ref|XP_001216111.1|/0/conserved hypothetical protein [Aspergillus terreus NIH2624]
4315966	1280	732.6	4.55	-6.317048051	8.14E-44	4.55E-42	Down	ATEG_01669	hypothetical protein	ko01100//Metabolic pathways;ko00520//Amino sugar and nucleotide sugar metabolism	GO:0005576//extracellular region	GO:0016977//chitosanase activity (fungal cell wall degradation and remodeling)	GO:0000272//polysaccharide catabolic process	gi|115384974|ref|XP_001209034.1|/0/predicted protein [Aspergillus terreus NIH2624]
4322740	693	164.27	0	-6.313091158	9.77E-15	1.37E-13	Down	ATEG_07491	hypothetical protein	ko01100//Metabolic pathways;ko00360//Phenylalanine metabolism	-	GO:0008080//N-acetyltransferase activity (detoxification)	-	gi|115401048|ref|XP_001216112.1|/1.05115e-173/predicted protein [Aspergillus terreus NIH2624]
4354529	939	536.83	3.21	-6.256001747	1.15E-34	4.60E-33	Down	ATEG_09735	hypothetical protein	ko03013//RNA transport;ko03015//mRNA surveillance pathway	GO:0016021//integral component of membrane	-	-	gi|115443100|ref|XP_001218357.1|/0/predicted protein [Aspergillus terreus NIH2624]
4355406	1110	1127.54	8.05	-6.248848228	8.74E-65	9.12E-63	Down	ATEG_00651	hypothetical protein	ko01100//Metabolic pathways;ko00564//Glycerophospholipid metabolism;ko00565//Ether lipid metabolism	GO:0016021//integral component of membrane	-	-	gi|115492219|ref|XP_001210737.1|/0/predicted protein [Aspergillus terreus NIH2624]
4354488	981	261.13	0.9	-6.240378593	2.78E-20	5.75E-19	Down	ATEG_09841	hypothetical protein	-	-	-	-	gi|115443312|ref|XP_001218463.1|/0/predicted protein [Aspergillus terreus NIH2624]
4320829	1080	365.2	1.8	-6.192053014	8.67E-25	2.31E-23	Down	ATEG_05019	hypothetical protein	ko00310//Lysine degradation	GO:0016021//integral component of membrane	-	-	gi|115397211|ref|XP_001214197.1|/0/predicted protein [Aspergillus terreus NIH2624]

4318444	840	148.03	0	-6.146467549	9.78E-14	1.30E-12	Down	ATEG_04016	hypothetical protein	ko04144//Endocytosis	-	-	-	gi|115391379|ref|XP_001213194.1|/0/predicted protein [Aspergillus terreus NIH2624]
4354101	2115	262.51	0.88	-6.113002143	4.96E-18	8.72E-17	Down	ATEG_09767	hypothetical protein	ko01100//Metabolic pathways;ko01110//Biosynthesis of secondary metabolites;ko01130//Biosynthesis of antibiotics;ko00100//Steroid biosynthesis	-	GO:0016866//intramolecular transferase activity	-	gi|115443164|ref|XP_001218389.1|/0/conserved hypothetical protein [Aspergillus terreus NIH2624]
4319068	696	266.19	1.38	-5.995185116	1.01E-20	2.16E-19	Down	ATEG_07172	similar to expression library immunization antigen 1	-	GO:0009277//fungal-type cell wall	-	-	gi|115386634|ref|XP_001209858.1|/1.79163e-161/hypothetical protein ATEG_07172 [Aspergillus terreus NIH2624]
4322609	1692	236.83	0.9	-5.982063254	4.41E-17	7.34E-16	Down	ATEG_07664	hypothetical protein	ko01100//Metabolic pathways;ko01110//Biosynthesis of secondary metabolites;ko01130//Biosynthesis of antibiotics;ko00230//Purine metabolism	GO:0016021//integral component of membrane	-	GO:0055085//transmembrane transport;GO:1900554	gi|115401394|ref|XP_001216285.1|/0/conserved hypothetical protein [Aspergillus terreus NIH2624]
4318118	2424	528.68	4.06	-5.945923362	1.18E-33	4.57E-32	Down	ATEG_02801	hypothetical protein	-	-	-	-	gi|115388948|ref|XP_001211979.1|/0/predicted protein [Aspergillus terreus NIH2624]

4315609	555	124.36	0	-5.930767786	1.26E-12	1.59E-11	Down	ATEG_01066	hypothetical protein	-	-	-	-	gi|115383768|ref|XP_001208431.1|/1.38645e-98/conserved hypothetical protein [Aspergillus terreus NIH2624]
4317204	1879	1297.16	12.18	-5.922910664	1.61E-69	1.98E-67	Down	ATEG_02711	siderophore iron transporter mirB	-	GO:0016021//integral component of membrane	-	GO:0055085//transmembrane transport	gi|115388767|ref|XP_001211889.1|/0/siderophore iron transporter mirB [Aspergillus terreus NIH2624]
4317789	1176	122.76	0	-5.922133926	1.34E-12	1.67E-11	Down	ATEG_03136	hypothetical protein	-	-	-	-	gi|115389618|ref|XP_001212314.1|/0/predicted protein [Aspergillus terreus NIH2624]
4315774	1140	1171.8	11.4	-5.884937752	4.31E-66	4.70E-64	Down	ATEG_01730	hypothetical protein	-	-	GO:0050364	GO:0009820//alkaloid metabolic process;GO:0044249//cellular biosynthetic process;GO:0044550//secondary metabolite biosynthetic process;GO:0044763;GO:1901576	gi|115385096|ref|XP_001209095.1|/0/predicted protein [Aspergillus terreus NIH2624]
4355100	1116	112.08	0	-5.801299186	5.41E-12	6.47E-11	Down	ATEG_00348	3-dehydroshikimate dehydratase	ko01100//Metabolic pathways;ko00350//Tyrosine metabolism;ko00360//Phenylalanine metabolism;ko00130//Ubiquinone and other terpenoid-quinone biosynthesis	-	GO:0016853//isomerase activity;GO:0046565;GO:0051213//dioxygenase activity	GO:0019631;GO:0055114//oxidation-reduction process	gi|115491613|ref|XP_001210434.1|/0/3-dehydroshikimate dehydratase [Aspergillus terreus NIH2624]

4323013	1446	908.7	9.02	-5.79695646	1.33E-53	9.99E-52	Down	ATEG_07660	hypothetical protein	-	GO:0005576//extracellular region	GO:0016614//oxidoreductase activity, acting on CH-OH group of donors;GO:0050660//flavin adenine dinucleotide binding	GO:0055114//oxidation-reduction process;GO:1900554	gi|115401386|ref|XP_001216281.1|/0/conserved hypothetical protein [Aspergillus terreus NIH2624]
4353830	1212	150.79	0.46	-5.774599703	4.13E-14	5.65E-13	Down	ATEG_09134	hypothetical protein	-	GO:0016021//integral component of membrane	GO:0008496//mannan endo-1,6-alpha-mannosidase activity (mannose hydrolysation)	GO:0016052//carbohydrate catabolic process	gi|115437220|ref|XP_001217756.1|/0/conserved hypothetical protein [Aspergillus terreus NIH2624]
4321206	1059	380.03	3.09	-5.738480087	7.02E-27	2.09E-25	Down	ATEG_04941	24 kDa metalloproteinase precursor	-	GO:0005576//extracellular region	GO:0004222//metalloendopeptidase activity;GO:0046872//metal ion binding (cleavage of protein, need metals)	GO:0006508//proteolysis	gi|115397055|ref|XP_001214119.1|/0/24 kDa metalloproteinase precursor [Aspergillus terreus NIH2624]
4317528	1056	146.28	0.44	-5.735863904	6.31E-14	8.49E-13	Down	ATEG_03450	hypothetical protein	ko00910//Nitrogen metabolism	-	GO:0018580//nitronate monooxygenase activity (protect the fungi from the environmental occurrence of the metabolic toxin)	GO:0055114//oxidation-reduction process	gi|115390246|ref|XP_001212628.1|/0/predicted protein [Aspergillus terreus NIH2624]
4354528	1971	2093.14	26.4	-5.591233246	5.39E-95	1.18E-92	Down	ATEG_09734	hypothetical protein	-	GO:0016021//integral component of membrane	GO:0015171//amino acid transmembrane transporter activity	GO:0003333//amino acid transmembrane transport	gi|115443098|ref|XP_001218356.1|/0/predicted protein [Aspergillus terreus NIH2624]

4317257	1434	228.62	1.8	-5.549747715	5.26E-19	9.90E-18	Down	ATEG_02658	hypothetical protein	ko01220//Degradation of aromatic compounds	-	GO:0004499//N,N-dimethylaniline monooxygenase activity;GO:0050660//flavin adenine dinucleotide binding;GO:0050661//NADP binding (oxygenation of amines)	GO:0055114//oxidation-reduction process	gi|115388661|ref|XP_001211836.1|/0/conserved hypothetical protein [Aspergillus terreus NIH2624]
4354675	1866	11723.9	158.34	-5.54582755	0	0	Down	ATEG_10350	hypothetical protein	-	GO:0016021//integral component of membrane	GO:0005215//transporter activity	GO:0006857//oligopeptide transport	gi|115449797|ref|XP_001218698.1|/0/conserved hypothetical protein [Aspergillus terreus NIH2624]
4322611	1638	527.76	5.95	-5.529099476	8.19E-34	3.17E-32	Down	ATEG_07666	hypothetical protein	-	GO:0005634//nucleus	GO:0003677//DNA binding;GO:0008270//zinc ion binding	GO:0006355//regulation of transcription, DNA-templated	gi|115401398|ref|XP_001216287.1|/0/conserved hypothetical protein [Aspergillus terreus NIH2624]
4353592	1056	91.94	0	-5.499577983	1.59E-10	1.66E-09	Down	ATEG_08393	hypothetical protein	-	-	-	-	gi|115433755|ref|XP_001217014.1|/0/predicted protein [Aspergillus terreus NIH2624]
4315965	1435	489.76	5.84	-5.44116241	8.86E-35	3.57E-33	Down	ATEG_01668	hypothetical protein	ko01100//Metabolic pathways;ko00520//Amino sugar and nucleotide sugar metabolism	-	-	-	gi|115384972|ref|XP_001209033.1|/0/predicted protein [Aspergillus terreus NIH2624]
4320190	1346	2493.58	35.39	-5.420421826	8.54E-91	1.63E-88	Down	ATEG_04723	hypothetical protein	-	-	GO:0003723//RNA binding;GO:0004540//ribonuclease activity	GO:0090501//RNA phosphodiester bond hydrolysis	gi|115396524|ref|XP_001213901.1|/1.03667e-74/predicted protein [Aspergillus terreus NIH2624]

4322540	1788	814.85	10.89	-5.349929113	2.75E-38	1.29E-36	Down	ATEG_07487	hypothetical protein	-	GO:0016021//integral component of membrane	-	GO:0055085//transmembrane transport	gi|115401040|ref|XP_001216108.1|/0/conserved hypothetical protein [Aspergillus terreus NIH2624]
4322542	4002	215.4	1.8	-5.345878515	1.08E-15	1.63E-14	Down	ATEG_07489	similar to ABC transporter	ko02010//ABC transporters	GO:0016021//integral component of membrane	GO:0005524//ATP binding;GO:0042626//ATPase activity, coupled to transmembrane movement of substances	GO:0055085//transmembrane transport	gi|115401044|ref|XP_001216110.1|/0/hypothetical protein ATEG_07489 [Aspergillus terreus NIH2624]

4323290	1152	95.7	0	-5.344826968	1.30E-09	1.24E-08	Down	ATEG_08954	hypothetical protein	ko03018//RNA degradation	GO:0000176//nuclear exosome (RNase complex);GO:0000177//cytoplasmic exosome (RNase complex)	GO:0003723//RNA binding	[illegible]///exonucleolytic trimming to generate mature 3'-end of 5.8S rRNA from tricistronic rRNA transcript (SSU-rRNA, 5.8S rRNA, LSU-rRNA);GO:0034427//nuclear-transcribed mRNA catabolic process, exonucleolytic, 3'-5';GO:0034475//U4 snRNA 3'-end processing;GO:0071035//nuclear polyadenylation-dependent rRNA catabolic process;GO:0071038//nuclear polyadenylation-dependent tRNA catabolic process;GO:0071049//nuclear retention of pre-mRNA with aberrant 3'-ends at the site of transcription;GO:0071051//polyadenylation-[illegible]	gi|115402927|ref|XP_001217540.1|/0/conserved hypothetical protein [Aspergillus terreus NIH2624]
4316734	558	83.45	0	-5.334912512	9.06E-10	8.77E-09	Down	ATEG_02274	hypothetical protein	ko04144//Endocytosis	GO:0016021//integral component of membrane	-	-	gi|115387893|ref|XP_001211452.1|/6.71997e-132/conserved hypothetical protein [Aspergillus terreus NIH2624]

4321752	1974	84.56	0	-5.327946208	1.04E-09	9.98E-09	Down	ATEG_05948	hypothetical protein	ko01100//Metabolic pathways;ko01130//Biosynthesis of antibiotics;ko01230//Biosynthesis of amino acids;ko01200//Carbon metabolism;ko00260//Glycine, serine and threonine metabolism;ko00680//Methane metabolism	-	-	-	gi|115399074|ref|XP_001215126.1|/0/conserved hypothetical protein [Aspergillus terreus NIH2624]
4317277	1173	79.57	0	-5.303459987	1.11E-09	1.07E-08	Down	ATEG_02649	hypothetical protein	ko03030//DNA replication	GO:0000790//nuclear chromatin;GO:0005730//nucleolus	GO:0003676//nucleic acid binding;GO:0004523//RNA-DNA hybrid ribonuclease activity	GO:0090502//RNA phosphodiester bond hydrolysis, endonucleolytic	gi|115388643|ref|XP_001211827.1|/0/conserved hypothetical protein [Aspergillus terreus NIH2624]
4317445	1417	194.53	1.78	-5.285040051	2.50E-16	3.99E-15	Down	ATEG_02812	hypothetical protein	-	-	-	-	gi|115388970|ref|XP_001211990.1|/0/predicted protein [Aspergillus terreus NIH2624]
4321554	861	290.28	3.57	-5.225237598	9.08E-23	2.16E-21	Down	ATEG_06120	hypothetical protein	-	-	GO:0016491//oxidoreductase activity	GO:0055114//oxidation-reduction process	gi|115399418|ref|XP_001215298.1|/0/conserved hypothetical protein [Aspergillus terreus NIH2624]
4323014	8214	1638.57	26.5	-5.22515488	1.52E-93	3.10E-91	Down	ATEG_07661	similar to polyketide synthase	-	-	GO:0016746//transferase activity, transferring acyl groups;GO:0031177//phosphopantetheine binding (fatty acid and polyketide biosynthesis)	GO:0008152//metabolic process	gi|115401388|ref|XP_001216282.1|/0/hypothetical protein ATEG_07661 [Aspergillus terreus NIH2624]

4323562	1386	178.81	1.8	-5.18171684	1.09E-15	1.65E-14	Down	ATEG_08713	hypothetical protein	ko01100//Metabolic pathways;ko00500//Starch and sucrose metabolism	-	-	-	gi|115402445|ref|XP_001217299.1|/0/predicted protein [Aspergillus terreus NIH2624]
4321999	1998	96.39	0.44	-5.123612923	1.66E-10	1.73E-09	Down	ATEG_06323	similar to monooxygenase	ko01100//Metabolic pathways;ko01220//Degradation of aromatic compounds	GO:0016021//integral component of membrane	GO:0016829//lyase activity;GO:0018658;GO:0071949//FAD binding (hydrolysis and oxidation)	GO:0055114//oxidation-reduction process	gi|115399824|ref|XP_001215501.1|/0/hypothetical protein ATEG_06323 [Aspergillus terreus NIH2624]
4323295	831	66.15	0	-5.018880263	1.60E-08	1.36E-07	Down	ATEG_08706	hypothetical protein	-	GO:0016021//integral component of membrane	-	-	gi|115402431|ref|XP_001217292.1|/0/conserved hypothetical protein [Aspergillus terreus NIH2624]
4317487	2525	278.13	4.06	-4.992355367	6.94E-20	1.41E-18	Down	ATEG_03443	protein TOXD	ko01100//Metabolic pathways;ko01220//Degradation of aromatic compounds	GO:0016020//membrane	GO:0016491//oxidoreductase activity;GO:0071949//FAD binding	GO:0055114//oxidation-reduction process	gi|115390232|ref|XP_001212621.1|/0/protein TOXD [Aspergillus terreus NIH2624]
4321723	1026	64.41	0	-4.951769699	3.02E-08	2.51E-07	Down	ATEG_06080	hypothetical protein	ko00500//Starch and sucrose metabolism	GO:0016021//integral component of membrane	-	-	gi|115399338|ref|XP_001215258.1|/0/predicted protein [Aspergillus terreus NIH2624]
4322936	1305	3204	65.09	-4.934210508	4.33E-139	1.62E-136	Down	ATEG_07786	hypothetical protein	-	-	GO:0005509//calcium ion binding;GO:0005544//calcium-dependent phospholipid binding	-	gi|115401638|ref|XP_001216407.1|/0/conserved hypothetical protein [Aspergillus terreus NIH2624]
4355407	1608	926.2	17.92	-4.923087271	2.85E-56	2.32E-54	Down	ATEG_00652	hypothetical protein	-	GO:0016021//integral component of membrane	GO:0015171//amino acid transmembrane transporter activity	GO:0003333//amino acid transmembrane transport	gi|115492221|ref|XP_001210738.1|/0/conserved hypothetical protein [Aspergillus terreus NIH2624]

4321241	1335	127.82	1.31	-4.878001749	8.27E-12	9.69E-11	Down	ATEG_05075	hypothetical protein	ko01100//Metabolic pathways;ko00500//Starch and sucrose metabolism	-	-	-	gi|115397323|ref|XP_001214253.1|/0/conserved hypothetical protein [Aspergillus terreus NIH2624]
4322937	1093	538.61	10.33	-4.870071273	3.82E-37	1.76E-35	Down	ATEG_07787	hypothetical protein	-	-	GO:0016798//hydrolase activity, acting on glycosyl bonds	GO:0008152//metabolic process	gi|115401640|ref|XP_001216408.1|/5.89657e-74/predicted protein [Aspergillus terreus NIH2624]
4315981	1287	160.87	2.24	-4.822261841	3.26E-14	4.49E-13	Down	ATEG_01678	similar to UDP-glucose 4-epimerase	ko01100//Metabolic pathways;ko01110//Biosynthesis of secondary metabolites;ko01130//Biosynthesis of antibiotics;ko00520//Amino sugar and nucleotide sugar metabolism;ko00010//Glycolysis / Gluconeogenesis;ko00052//Galactose metabolism	-	GO:0003974//UDP-N-acetylglucosamine 4-epimerase activity;GO:0003978//UDP-glucose 4-epimerase activity;GO:0050662//coenzyme binding (aminosugars metabolism)	GO:0006012//galactose metabolic process	gi|115384992|ref|XP_001209043.1|/0/hypothetical protein ATEG_01678 [Aspergillus terreus NIH2624]
4321050	825	341.92	6.2	-4.793555488	1.75E-23	4.32E-22	Down	ATEG_05357	hypothetical protein	ko04011//MAPK signaling pathway - yeast	GO:0016021//integral component of membrane	-	-	gi|115397887|ref|XP_001214535.1|/0/predicted protein [Aspergillus terreus NIH2624]
4323566	1151	453.25	9.04	-4.793062404	4.57E-31	1.60E-29	Down	ATEG_08625	hypothetical protein	ko00970//Aminoacyl-tRNA biosynthesis;ko00450//Selenocompound metabolism	-	-	-	gi|115402269|ref|XP_001217211.1|/0/conserved hypothetical protein [Aspergillus terreus NIH2624]

4355318	735	143.56	1.85	-4.7783485	7.37E-12	8.69E-11	Down	ATEG_00564	hypothetical protein	ko04144//Endocytosis	-	-	-	gi|115492045|ref|XP_001210650.1|/0/conserved hypothetical protein [Aspergillus terreus NIH2624]
4317063	714	270.74	4.91	-4.755525887	1.45E-20	3.07E-19	Down	ATEG_02154	hypothetical protein	ko04011//MAPK signaling pathway - yeast	-	-	-	gi|115387653|ref|XP_001211332.1|/9.99741e-171/predicted protein [Aspergillus terreus NIH2624]
4319612	1439	54.36	0	-4.731828933	1.72E-07	1.32E-06	Down	ATEG_09966	hypothetical protein	ko03410//Base excision repair	GO:0005634//nucleus	GO:0000981//RNA polymerase II transcription factor activity, sequence-specific DNA binding;GO:0003677//DNA binding;GO:0008270//zinc ion binding	GO:0006357//regulation of transcription from RNA polymerase II promoter	gi|115385443|ref|XP_001209268.1|/0/predicted protein [Aspergillus terreus NIH2624]
4318031	1261	54.38	0	-4.721080084	1.90E-07	1.46E-06	Down	ATEG_03436	hypothetical protein	-	GO:0016021//integral component of membrane	GO:0005215//transporter activity	GO:0055085//transmembrane transport	gi|115390218|ref|XP_001212614.1|/0/predicted protein [Aspergillus terreus NIH2624]
4354286	3939	7958.74	193.78	-4.686647787	8.77E-182	6.33E-179	Down	ATEG_09875	dipeptidyl-peptidase 5 precursor	ko03040//Spliceosome	GO:0005576//extracellular region	GO:0004177//aminopeptidase activity;GO:0008236//serine-type peptidase activity	GO:0006508//proteolysis	gi|115443380|ref|XP_001218497.1|/0/dipeptidyl-peptidase 5 precursor [Aspergillus terreus NIH2624]

4322664	2117	305.97	6.37	-4.660908134	1.49E-22	3.52E-21	Down	ATEG_07777	glutamate decarboxylase	ko01100//Metabolic pathways;ko01110//Biosynthesis of secondary metabolites;ko00650//Butanoate metabolism;ko00250//Alanine, aspartate and glutamate metabolism;ko00410//beta-Alanine metabolism;ko00430//Taurine and hypotaurine metabolism	GO:0016021//integral component of membrane	GO:0004351//glutamate decarboxylase activity;GO:0030170//pyridoxal phosphate binding	GO:0006536//glutamate metabolic process	gi|115401620|ref|XP_001216398.1|/0/glutamate decarboxylase [Aspergillus terreus NIH2624]
4354898	1611	1333.73	32.43	-4.654723898	5.22E-77	7.10E-75	Down	ATEG_00141	hypothetical protein	-	-	GO:0005507//copper ion binding;GO:0016491//oxidoreductase activity	GO:0055114//oxidation-reduction process	gi|115491199|ref|XP_001210227.1|/0/predicted protein [Aspergillus terreus NIH2624]
4354495	1719	3255.59	81.08	-4.648419695	5.01E-117	1.47E-114	Down	ATEG_09721	dicarboxylic amino acid permease	-	GO:0000328//fungal-type vacuole lumen;GO:0005886//plasma membrane;GO:0016021//integral component of membrane	GO:0005313//L-glutamate transmembrane transporter activity;GO:0015183//L-aspartate transmembrane transporter activity	GO:0003333//amino acid transmembrane transport;GO:0015813//L-glutamate transport;GO:0070778//L-aspartate transport	gi|115443072|ref|XP_001218343.1|/0/dicarboxylic amino acid permease [Aspergillus terreus NIH2624]
4315626	1779	142.38	2.31	-4.608436316	4.45E-12	5.36E-11	Down	ATEG_01017	hypothetical protein	-	GO:0016021//integral component of membrane	GO:0005315//inorganic phosphate transmembrane transporter activity	GO:0006817//phosphate ion transport;GO:0055085//transmembrane transport	gi|115383670|ref|XP_001208382.1|/0/conserved hypothetical protein [Aspergillus terreus NIH2624]

4315670	1768	381.22	7.95	-4.607140286	3.40E-22	7.89E-21	Down	ATEG_01083	cytochrome P450 61	ko01100//Metabolic pathways;ko01130//Biosynthesis of antibiotics;ko00100//Steroid biosynthesis	GO:0005783//endoplasmic reticulum;GO:0016021//integral component of membrane	GO:0000249//C-22 sterol desaturase activity;GO:0004497//monooxygenase activity;GO:0005506//iron ion binding;GO:0009055//electron carrier activity;GO:0020037//heme binding	GO:0006623//protein targeting to vacuole;GO:0006696//ergosterol biosynthetic process;GO:0006897//endocytosis;GO:0055114//oxidation-reduction process;GO:0071210	gi|115383802|ref|XP_001208448.1|/0/cytochrome P450 61 [Aspergillus terreus NIH2624]
4320666	525	189.98	3.6	-4.604500409	5.94E-16	9.21E-15	Down	ATEG_05128	hypothetical protein	-	-	-	-	gi|115397429|ref|XP_001214306.1|/1.22754e-133/predicted protein [Aspergillus terreus NIH2624]
4321049	2917	787.55	19.54	-4.580830725	2.84E-42	1.51E-40	Down	ATEG_05356	hypothetical protein	ko01100//Metabolic pathways;ko01110//Biosynthesis of secondary metabolites;ko00520//Amino sugar and nucleotide sugar metabolism;ko00051//Fructose and mannose metabolism	-	GO:0004476//mannose-6-phosphate isomerase activity;GO:0008270//zinc ion binding	GO:0005975//carbohydrate metabolic process;GO:0009298//GDP-mannose biosynthetic process	gi|115397885|ref|XP_001214534.1|/0/conserved hypothetical protein [Aspergillus terreus NIH2624]
4354896	5279	6229.32	164.19	-4.579748441	4.20E-103	1.07E-100	Down	ATEG_00139	hypothetical protein	-	GO:0005634//nucleus	GO:0000981//RNA polymerase II transcription factor activity, sequence-specific DNA binding;GO:0008270//zinc ion binding	GO:0006357//regulation of transcription from RNA polymerase II promoter	gi|115491195|ref|XP_001210225.1|/0/predicted protein [Aspergillus terreus NIH2624]

4317486	1482	253.37	5.44	-4.560940765	4.51E-19	8.57E-18	Down	ATEG_03442	hypothetical protein	-	-	GO:0016614//oxidoreductase activity, acting on CH-OH group of donors;GO:0050660//flavin adenine dinucleotide binding	GO:0055114//oxidation-reduction process	gi|115390230|ref|XP_001212620.1|/0/conserved hypothetical protein [Aspergillus terreus NIH2624]
4318828	3060	1324.35	34.81	-4.520351143	3.53E-52	2.53E-50	Down	ATEG_07068	hypothetical protein	ko04144//Endocytosis	-	-	-	gi|115386426|ref|XP_001209754.1|/0/predicted protein [Aspergillus terreus NIH2624]
4317205	1119	277.5	6.34	-4.504217733	6.97E-20	1.42E-18	Down	ATEG_02712	hypothetical protein	-	-	-	-	gi|115388769|ref|XP_001211890.1|/0/conserved hypothetical protein [Aspergillus terreus NIH2624]
4323130	1502	114.92	1.85	-4.487416302	2.26E-10	2.32E-09	Down	ATEG_08606	hypothetical protein	-	GO:0016021//integral component of membrane	-	-	gi|115402231|ref|XP_001217192.1|/0/predicted protein [Aspergillus terreus NIH2624]
4321509	669	554.11	14.46	-4.477320949	2.81E-35	1.17E-33	Down	ATEG_06139	N,O-diacetylmuramidase	ko01100//Metabolic pathways;ko00520//Amino sugar and nucleotide sugar metabolism	-	GO:0003796//lysozyme activity	GO:0005975//carbohydrate metabolic process;GO:0009253//peptidoglycan catabolic process;GO:0016998//cell wall macromolecule catabolic process	gi|115399456|ref|XP_001215317.1|/2.47891e-168/N,O-diacetylmuramidase [Aspergillus terreus NIH2624]
4323322	1761	207.43	4.5	-4.469359537	3.84E-16	6.01E-15	Down	ATEG_08640	hypothetical protein	ko01100//Metabolic pathways	-	GO:0016787//hydrolase activity	-	gi|115402299|ref|XP_001217226.1|/0/conserved hypothetical protein [Aspergillus terreus NIH2624]

4321512	870	95.79	1.38	-4.455445138	2.49E-09	2.32E-08	Down	ATEG_06142	hypothetical protein	-	-	-	-	gi|115399462|ref|XP_001215320.1|/0/predicted protein [Aspergillus terreus NIH2624]
4354704	1776	45.37	0	-4.431875478	1.63E-06	1.10E-05	Down	ATEG_10248	hypothetical protein	-	GO:0016021//integral component of membrane	-	GO:0055085//transmembrane transport	gi|115449397|ref|XP_001218596.1|/0/conserved hypothetical protein [Aspergillus terreus NIH2624]
4353707	1062	45.42	0	-4.425526678	1.71E-06	1.15E-05	Down	ATEG_09258	hypothetical protein	ko03010//Ribosome	GO:0016021//integral component of membrane	-	-	gi|115437712|ref|XP_001217880.1|/0/conserved hypothetical protein [Aspergillus terreus NIH2624]
4354928	972	218.06	4.89	-4.420222084	2.83E-16	4.48E-15	Down	ATEG_00171	hypothetical protein	ko01100//Metabolic pathways;ko00561//Glycerolipid metabolism	-	GO:0016298//lipase activity;GO:0052689//carboxylic ester hydrolase activity	GO:0008152//metabolic process	gi|115491259|ref|XP_001210257.1|/0/conserved hypothetical protein [Aspergillus terreus NIH2624]
4319543	5342	4931.46	143.71	-4.413859156	3.23E-78	4.52E-76	Down	ATEG_09958	hypothetical protein	-	-	-	-	gi|115385427|ref|XP_001209260.1|/0/conserved hypothetical protein [Aspergillus terreus NIH2624]

4355555	2199	186.11	4.13	-4.401463427	8.11E-14	1.08E-12	Down	ATEG_00794	catalase A	ko01110//Biosynthesis of secondary metabolites;ko01130//Biosynthesis of antibiotics;ko01200//Carbon metabolism;ko04011//MAPK signaling pathway - yeast;ko04146//Peroxisome;ko00380//Tryptophan metabolism;ko00630//Glyoxylate and dicarboxylate metabolism	GO:0005622//intracellular	GO:0004096//catalase activity;GO:0020037//heme binding;GO:0046872//metal ion binding	GO:0034605//cellular response to heat;GO:0042744//hydrogen peroxide catabolic process;GO:0055114//oxidation-reduction process;GO:0070301//cellular response to hydrogen peroxide;GO:0098869//cellular oxidant detoxification	gi|115492505|ref|XP_001210880.1|/0/catalase A [Aspergillus terreus NIH2624]
4353303	840	91.31	1.31	-4.364823449	6.69E-09	5.94E-08	Down	ATEG_08231	hypothetical protein	ko01100//Metabolic pathways;ko00190//Oxidative phosphorylation	-	-	-	gi|115433430|ref|XP_001216852.1|/0/predicted protein [Aspergillus terreus NIH2624]
4355529	357	248.27	6.3	-4.358514162	4.74E-19	8.99E-18	Down	ATEG_00771	hypothetical protein	-	-	-	-	gi|115492459|ref|XP_001210857.1|/4.24392e-80/predicted protein [Aspergillus terreus NIH2624]
4316489	840	87.41	1.34	-4.346378222	5.49E-09	4.93E-08	Down	ATEG_02037	hypothetical protein	ko04145//Phagosome	GO:0016021//integral component of membrane	-	-	gi|115387419|ref|XP_001211215.1|/0/predicted protein [Aspergillus terreus NIH2624]
4319107	1687	87.43	1.38	-4.32677486	9.93E-09	8.64E-08	Down	ATEG_07342	hypothetical protein	-	GO:0016021//integral component of membrane	GO:0015171//amino acid transmembrane transporter activity	GO:0003333//amino acid transmembrane transport	gi|115386974|ref|XP_001210028.1|/0/conserved hypothetical protein [Aspergillus terreus NIH2624]

4316367	997	70.62	0.9	-4.310921736	6.36E-08	5.14E-07	Down	ATEG_00995	UDP-glucuronic acid decarboxylase 1	ko01100//Metabolic pathways;ko00500//Starch and sucrose metabolism;ko00520//Amino sugar and nucleotide sugar metabolism	-	-	-	gi|115383626|ref|XP_001208360.1|/0/UDP-glucuronic acid decarboxylase 1 [Aspergillus terreus NIH2624]
4315816	2436	1332.79	41.94	-4.295826751	1.38E-70	1.77E-68	Down	ATEG_01675	hypothetical protein	ko01100//Metabolic pathways;ko00500//Starch and sucrose metabolism	-	-	-	gi|115384986|ref|XP_001209040.1|/0/conserved hypothetical protein [Aspergillus terreus NIH2624]
4319120	759	55.47	0.44	-4.276952342	6.77E-07	4.80E-06	Down	ATEG_07316	hypothetical protein	-	-	-	-	gi|115386922|ref|XP_001210002.1|/0/predicted protein [Aspergillus terreus NIH2624]
4317820	990	56.07	0.44	-4.268484143	7.76E-07	5.43E-06	Down	ATEG_03428	hypothetical protein	-	-	-	-	gi|115390202|ref|XP_001212606.1|/0/conserved hypothetical protein [Aspergillus terreus NIH2624]
4323323	1517	150.79	3.55	-4.22952431	3.23E-12	3.92E-11	Down	ATEG_08641	hypothetical protein	-	GO:0016021//integral component of membrane	-	-	gi|115402301|ref|XP_001217227.1|/0/conserved hypothetical protein [Aspergillus terreus NIH2624]
4322964	747	41.43	0	-4.221249816	6.98E-06	4.23E-05	Down	ATEG_07952	hypothetical protein	-	-	-	-	gi|115401970|ref|XP_001216573.1|/0/predicted protein [Aspergillus terreus NIH2624]

4322229	1677	1195.22	27.99	-4.206796512	3.21E-11	3.59E-10	Down	ATEG_06291	hypothetical protein	ko03440//Homologous recombination;ko03450//Non-homologous end-joining	GO:0016021//integral component of membrane	-	GO:0055085//transmembrane transport	gi|115399760|ref|XP_001215469.1|/0/conserved hypothetical protein [Aspergillus terreus NIH2624]
4353835	1314	38.66	0	-4.206706232	7.10E-06	4.30E-05	Down	ATEG_09047	hypothetical protein	ko01100//Metabolic pathways;ko01110//Biosynthesis of secondary metabolites;ko00564//Glycerophospholipid metabolism	-	GO:0004609//phosphatidylserine decarboxylase activity	GO:0008654//phospholipid biosynthetic process	gi|115436884|ref|XP_001217669.1|/0/predicted protein [Aspergillus terreus NIH2624]
4316418	1020	159.69	4.11	-4.202882363	9.81E-13	1.24E-11	Down	ATEG_01903	hypothetical protein	-	-	-	-	gi|115387151|ref|XP_001211081.1|/0/predicted protein [Aspergillus terreus NIH2624]
4317958	1019	67.22	0.88	-4.180243026	2.85E-07	2.14E-06	Down	ATEG_03530	hypothetical protein	-	GO:0016021//integral component of membrane	-	-	gi|115390406|ref|XP_001212708.1|/2.22198e-135/predicted protein [Aspergillus terreus NIH2624]
4318830	2364	39.26	0	-4.178871169	8.78E-06	5.27E-05	Down	ATEG_07070	hypothetical protein	-	-	GO:0003824//catalytic activity	GO:0009116//nucleoside metabolic process	gi|115386430|ref|XP_001209756.1|/0/conserved hypothetical protein [Aspergillus terreus NIH2624]
4322651	1602	610.85	20.18	-4.163323434	7.61E-38	3.54E-36	Down	ATEG_07891	hypothetical protein	-	-	GO:0016491//oxidoreductase activity;GO:0050058;GO:0050151//oleate hydratase activity;GO:0071949//FAD binding	GO:0006631//fatty acid metabolic process;GO:0055114//oxidation-reduction process	gi|115401848|ref|XP_001216512.1|/0/predicted protein [Aspergillus terreus NIH2624]

4318985	1371	79.03	1.31	-4.147660072	6.86E-08	5.52E-07	Down	ATEG_07375	hypothetical protein	ko01100//Metabolic pathways;ko01110//Biosynthesis of secondary metabolites;ko01130//Biosynthesis of antibiotics;ko00230//Purine metabolism	GO:0016021//integral component of membrane	-	GO:0055085//transmembrane transport	gi|115387040|ref|XP_001210061.1|/0/predicted protein [Aspergillus terreus NIH2624]
4319006	1446	508.39	16.51	-4.141905196	1.50E-29	4.96E-28	Down	ATEG_07270	hypothetical protein	ko00920//Sulfur metabolism	-	GO:0004497//monooxygenase activity;GO:0016705//oxidoreductase activity, acting on paired donors, with incorporation or reduction of molecular oxygen	GO:0055114//oxidation-reduction process	gi|115386830|ref|XP_001209956.1|/0/conserved hypothetical protein [Aspergillus terreus NIH2624]
4323177	3021	160.86	4.52	-4.108697323	4.81E-13	6.21E-12	Down	ATEG_08915	hypothetical protein	ko00562//Inositol phosphate metabolism;ko00053//Ascorbate and aldarate metabolism	-	GO:0005524//ATP binding	-	gi|115402849|ref|XP_001217501.1|/0/conserved hypothetical protein [Aspergillus terreus NIH2624]
4317678	1899	553.24	18.84	-4.107245482	2.71E-33	1.03E-31	Down	ATEG_03445	hypothetical protein	ko01100//Metabolic pathways;ko00920//Sulfur metabolism	GO:0005634//nucleus	GO:0000981//RNA polymerase II transcription factor activity, sequence-specific DNA binding;GO:0003677//DNA binding;GO:0008270//zinc ion binding	GO:0006357//regulation of transcription from RNA polymerase II promoter	gi|115390236|ref|XP_001212623.1|/0/predicted protein [Aspergillus terreus NIH2624]
4320704	1728	731.92	25.65	-4.107063529	5.53E-44	3.10E-42	Down	ATEG_05199	hypothetical protein	ko01100//Metabolic pathways;ko00500//Starch and sucrose metabolism	GO:0005634//nucleus	GO:0000981//RNA polymerase II transcription factor activity, sequence-specific DNA binding;GO:0008270//zinc ion binding	GO:0006357//regulation of transcription from RNA polymerase II promoter	gi|115397571|ref|XP_001214377.1|/0/conserved hypothetical protein [Aspergillus terreus NIH2624]

4320975	1140	35.87	0	-4.089833095	1.46E-05	8.48E-05	Down	ATEG_05564	hypothetical protein	ko01100//Metabolic pathways;ko04141//Protein processing in endoplasmic reticulum;ko00510//N-Glycan biosynthesis	-	GO:0004672//protein kinase activity;GO:0005524//ATP binding	GO:0006468//protein phosphorylation	gi|115398301|ref|XP_001214742.1|/0/predicted protein [Aspergillus terreus NIH2624]
4319545	1587	49.91	0.46	-4.075094836	3.72E-06	2.38E-05	Down	ATEG_09960	similar to cytochrome P450	ko01100//Metabolic pathways;ko01110//Biosynthesis of secondary metabolites	GO:0005789//endoplasmic reticulum membrane;GO:0016021//integral component of membrane	GO:0004497//monooxygenase activity;GO:0005506//iron ion binding;GO:0016705//oxidoreductase activity, acting on paired donors, with incorporation or reduction of molecular oxygen;GO:0020037//heme binding	GO:0030639//polyketide biosynthetic process;GO:0055114//oxidation-reduction process	gi|115385431|ref|XP_001209262.1|/0/hypothetical protein ATEG_09960 [Aspergillus terreus NIH2624]
4318492	819	48.77	0.44	-4.069333461	3.45E-06	2.23E-05	Down	ATEG_04114	hypothetical protein	-	-	GO:0016787//hydrolase activity	GO:0009166//nucleotide catabolic process	gi|115391575|ref|XP_001213292.1|/0/predicted protein [Aspergillus terreus NIH2624]
4320381	837	284.73	9.5	-4.052772144	1.22E-19	2.46E-18	Down	ATEG_04339	hypothetical protein	ko01100//Metabolic pathways;ko00520//Amino sugar and nucleotide sugar metabolism	GO:0016021//integral component of membrane	-	-	gi|115395508|ref|XP_001213517.1|/0/predicted protein [Aspergillus terreus NIH2624]
4354298	675	262.24	8.7	-4.022414446	9.13E-16	1.39E-14	Down	ATEG_09813	hypothetical protein	ko00500//Starch and sucrose metabolism	-	-	-	gi|115443256|ref|XP_001218435.1|/2.78468e-166/predicted protein [Aspergillus terreus NIH2624]

4323307	828	116.6	3.16	-4.009278303	4.18E-10	4.17E-09	Down	ATEG_08852	hypothetical protein	-	-	-	-	gi|115402723|ref|XP_001217438.1|/0/predicted protein [Aspergillus terreus NIH2624]
4320976	678	544	19.65	-4.005421588	2.36E-29	7.63E-28	Down	ATEG_05565	hypothetical protein	-	-	-	-	gi|115398303|ref|XP_001214743.1|/5.63109e-171/conserved hypothetical protein [Aspergillus terreus NIH2624]
4353194	850	614.71	23.09	-4.003444072	3.28E-35	1.35E-33	Down	ATEG_08184	hypothetical protein	-	-	GO:0016491//oxidoreductase activity	GO:0055114//oxidation-reduction process	gi|115433336|ref|XP_001216805.1|/0/predicted protein [Aspergillus terreus NIH2624]
4317191	483	45.96	0.46	-3.99558035	5.93E-06	3.66E-05	Down	ATEG_02723	hypothetical protein	-	GO:0016021//integral component of membrane	-	-	gi|115388791|ref|XP_001211901.1|/1.35909e-112/predicted protein [Aspergillus terreus NIH2624]
4323434	2433	34.2	0	-3.991175501	2.64E-05	0.00014781	Down	ATEG_08584	hypothetical protein	-	-	GO:0003677//DNA binding;GO:0046983//protein dimerization activity	-	gi|115402187|ref|XP_001217170.1|/0/predicted protein [Aspergillus terreus NIH2624]
4353705	1632	523.24	19.28	-3.988276748	1.63E-29	5.34E-28	Down	ATEG_09256	hypothetical protein	ko01100//Metabolic pathways;ko00230//Purine metabolism	GO:0016021//integral component of membrane	GO:0022891//substrate-specific transmembrane transporter activity	GO:0055085//transmembrane transport	gi|115437704|ref|XP_001217878.1|/0/conserved hypothetical protein [Aspergillus terreus NIH2624]
4322970	1998	974.92	37.99	-3.982350294	4.71E-48	3.05E-46	Down	ATEG_07496	hypothetical protein	ko01100//Metabolic pathways;ko01110//Biosynthesis of secondary metabolites;ko00100//Steroid biosynthesis	-	-	GO:0006355//regulation of transcription, DNA-templated	gi|115401058|ref|XP_001216117.1|/0/predicted protein [Aspergillus terreus NIH2624]

4322511	839	94.74	2.31	-3.969221154	4.34E-08	3.58E-07	Down	ATEG_07795	hypothetical protein	-	-	-	-	gi|115401656|ref|XP_001216416.1|/4.8387e-149/predicted protein [Aspergillus terreus NIH2624]
4319029	1729	33.64	0	-3.961138485	3.15E-05	0.00017388	Down	ATEG_07354	similar to (R,R)-butanediol dehydrogenase	ko00640//Propanoate metabolism	-	GO:0008270//zinc ion binding;GO:0016491//oxidoreductase activity	GO:0055114//oxidation-reduction process	gi|115386998|ref|XP_001210040.1|/0/hypothetical protein ATEG_07354 [Aspergillus terreus NIH2624]
4316163	1653	33.07	0	-3.96109406	3.10E-05	0.00017146	Down	ATEG_01646	hypothetical protein	-	GO:0005783//endoplasmic reticulum;GO:0005794//Golgi apparatus;GO:0016021//integral component of membrane	-	-	gi|115384928|ref|XP_001209011.1|/0/conserved hypothetical protein [Aspergillus terreus NIH2624]
4319005	1617	145.13	4.52	-3.957236664	8.75E-12	1.02E-10	Down	ATEG_07269	hypothetical protein	-	GO:0016021//integral component of membrane	GO:0022857//transmembrane transporter activity	GO:0006820//anion transport;GO:0055085//transmembrane transport	gi|115386828|ref|XP_001209955.1|/0/conserved hypothetical protein [Aspergillus terreus NIH2624]
4353473	1767	651.53	25.31	-3.934247885	9.24E-30	3.08E-28	Down	ATEG_08421	hypothetical protein	-	GO:0016021//integral component of membrane	-	GO:0055085//transmembrane transport	gi|115433811|ref|XP_001217042.1|/0/predicted protein [Aspergillus terreus NIH2624]
4353706	2533	276.7	9.97	-3.930143122	4.87E-17	8.07E-16	Down	ATEG_09257	hypothetical protein	ko03008//Ribosome biogenesis in eukaryotes;ko03018//RNA degradation	-	GO:0004061//arylformamidase activity	GO:0019441//tryptophan catabolic process to kynurenine	gi|115437708|ref|XP_001217879.1|/0/conserved hypothetical protein [Aspergillus terreus NIH2624]

4317611	4810	1789.35	73.75	-3.920307671	4.18E-59	3.66E-57	Down	ATEG_03247	hypothetical protein	-	-	GO:0046872//metal ion binding	-	gi|115389840|ref|XP_001212425.1|/2.02439e-112/predicted protein [Aspergillus terreus NIH2624]
4322864	1574	44.28	0.44	-3.92012389	9.98E-06	5.94E-05	Down	ATEG_07541	hypothetical protein	-	GO:0016021//integral component of membrane	-	GO:0006950//response to stress	gi|115401148|ref|XP_001216162.1|/1.73921e-157/predicted protein [Aspergillus terreus NIH2624]
4354788	846	56.05	0.88	-3.91419384	2.61E-06	1.71E-05	Down	ATEG_00032	hypothetical protein	-	-	GO:0004725//protein tyrosine phosphatase activity	GO:0035335//peptidyl-tyrosine dephosphorylation	gi|115490981|ref|XP_001210118.1|/0/predicted protein [Aspergillus terreus NIH2624]
4353593	1284	32.52	0	-3.913636889	4.09E-05	0.00022231	Down	ATEG_08394	hypothetical protein	-	-	-	-	gi|115433757|ref|XP_001217015.1|/0/predicted protein [Aspergillus terreus NIH2624]
4322539	1572	279.7	10.43	-3.905560474	2.65E-18	4.72E-17	Down	ATEG_07486	hypothetical protein	-	-	GO:0016491//oxidoreductase activity	GO:0055114//oxidation-reduction process	gi|115401038|ref|XP_001216107.1|/0/predicted protein [Aspergillus terreus NIH2624]
4322895	780	79.62	1.85	-3.9008572	3.20E-07	2.39E-06	Down	ATEG_07950	hypothetical protein	-	GO:0016021//integral component of membrane	-	-	gi|115401966|ref|XP_001216571.1|/1.65189e-180/conserved hypothetical protein [Aspergillus terreus NIH2624]
4355536	720	1080.85	45.05	-3.891429785	5.75E-55	4.39E-53	Down	ATEG_00777	hypothetical protein	ko04144//Endocytosis	-	-	-	gi|115492471|ref|XP_001210863.1|/3.11704e-175/conserved hypothetical protein [Aspergillus terreus NIH2624]

4316272	1953	190.54	6.71	-3.877639943	4.23E-14	5.77E-13	Down	ATEG_01760	hypothetical protein	-	-	GO:0008762//UDP-N-acetylmuramate dehydrogenase activity;GO:0050660//flavin adenine dinucleotide binding	GO:0055114//oxidation-reduction process	gi|115385156|ref|XP_001209125.1|/0/conserved hypothetical protein [Aspergillus terreus NIH2624]
4355552	1455	209.56	7.68	-3.870472929	3.87E-15	5.59E-14	Down	ATEG_00791	similar to acyl transferase	ko01100//Metabolic pathways;ko00280//Valine, leucine and isoleucine degradation	-	GO:0016747//transferase activity, transferring acyl groups other than amino-acyl groups	-	gi|115492499|ref|XP_001210877.1|/0/hypothetical protein ATEG_00791 [Aspergillus terreus NIH2624]
4354297	1549	34.79	0	-3.851622929	6.21E-05	0.00032451	Down	ATEG_09812	hypothetical protein	ko01100//Metabolic pathways;ko00561//Glycerolipid metabolism	-	-	-	gi|115443254|ref|XP_001218434.1|/0/conserved hypothetical protein [Aspergillus terreus NIH2624]
4322985	552	53.22	0.92	-3.846824679	4.77E-06	2.98E-05	Down	ATEG_07431	hypothetical protein	-	GO:0016021//integral component of membrane	-	-	gi|115400928|ref|XP_001216052.1|/1.93261e-130/conserved hypothetical protein [Aspergillus terreus NIH2624]
4317812	1527	53.26	0.92	-3.838103288	5.25E-06	3.27E-05	Down	ATEG_03358	hypothetical protein	ko01100//Metabolic pathways;ko00520//Amino sugar and nucleotide sugar metabolism	-	GO:0016491//oxidoreductase activity	GO:0055114//oxidation-reduction process	gi|115390062|ref|XP_001212536.1|/0/predicted protein [Aspergillus terreus NIH2624]
4354671	1357	312.1	12.69	-3.816235309	8.27E-20	1.67E-18	Down	ATEG_10400	hypothetical protein	ko00254//Aflatoxin biosynthesis	-	GO:0008171//O-methyltransferase activity	GO:0032259//methylation	gi|115449989|ref|XP_001218748.1|/0/conserved hypothetical protein [Aspergillus terreus NIH2624]

4318028	1470	93.06	2.67	-3.811610249	3.01E-08	2.51E-07	Down	ATEG_03433	hypothetical protein	-	-	GO:0016614//oxidoreductase activity, acting on CH-OH group of donors;GO:0050660//flavin adenine dinucleotide binding	GO:0055114//oxidation-reduction process	gi|115390212|ref|XP_001212611.1|/0/predicted protein [Aspergillus terreus NIH2624]
4322400	2207	2749.96	123.7	-3.80430048	3.25E-89	6.10E-87	Down	ATEG_06329	similar to siderochrome-iron uptake transporter	-	GO:0016021//integral component of membrane	-	GO:0055085//transmembrane transport	gi|115399836|ref|XP_001215507.1|/0/hypothetical protein ATEG_06329 [Aspergillus terreus NIH2624]
4315610	615	29.71	0	-3.792135669	7.82E-05	0.00040061	Down	ATEG_01067	hypothetical protein	-	-	-	-	gi|115383770|ref|XP_001208432.1|/6.36871e-153/predicted protein [Aspergillus terreus NIH2624]
4323296	459	42.63	0.44	-3.791262078	2.65E-05	0.00014827	Down	ATEG_08707	hypothetical protein	ko03015//mRNA surveillance pathway	-	-	-	gi|115402433|ref|XP_001217293.1|/9.72262e-118/predicted protein [Aspergillus terreus NIH2624]
4320359	2541	296.52	12.13	-3.786969027	2.57E-19	5.03E-18	Down	ATEG_04327	hypothetical protein	ko04145//Phagosome	GO:0016020//membrane	-	-	gi|115395360|ref|XP_001213505.1|/1.77762e-139/predicted protein [Aspergillus terreus NIH2624]
4317199	2964	632.55	27.1	-3.784462529	1.99E-25	5.51E-24	Down	ATEG_02719	hypothetical protein	ko00480//Glutathione metabolism	-	GO:0016787//hydrolase activity	-	gi|115388783|ref|XP_001211897.1|/0/conserved hypothetical protein [Aspergillus terreus NIH2624]

4321684	1686	29.69	0	-3.779764897	8.40E-05	0.00042799	Down	ATEG_05976	hypothetical protein	-	-	-	-	gi|115399130|ref|XP_001215154.1|/0/predicted protein [Aspergillus terreus NIH2624]
4353951	642	36.92	0	-3.742389295	0.00011736	0.00058086	Down	ATEG_09297	hypothetical protein	-	GO:0005634//nucleus;GO:0005829//cytosol	-	-	gi|115437864|ref|XP_001217919.1|/8.96851e-153/conserved hypothetical protein [Aspergillus terreus NIH2624]
4322333	936	287.51	12.06	-3.733629934	3.51E-18	6.22E-17	Down	ATEG_06795	hypothetical protein	ko01100//Metabolic pathways;ko00051//Fructose and mannose metabolism;ko00650//Butanoate metabolism;ko00591//Linoleic acid metabolism	GO:0005576//extracellular region	GO:0016491//oxidoreductase activity	GO:0008152//metabolic process	gi|115400769|ref|XP_001215973.1|/0/conserved hypothetical protein [Aspergillus terreus NIH2624]
4319248	855	47.64	0.9	-3.705445789	1.18E-05	6.96E-05	Down	ATEG_07133	hypothetical protein	-	GO:0016021//integral component of membrane	-	GO:0006950//response to stress	gi|115386556|ref|XP_001209819.1|/0/conserved hypothetical protein [Aspergillus terreus NIH2624]
4353414	1572	28.02	0	-3.703913433	0.00012321	0.00060814	Down	ATEG_08404	hypothetical protein	ko01100//Metabolic pathways;ko01110//Biosynthesis of secondary metabolites;ko01130//Biosynthesis of antibiotics;ko00230//Purine metabolism	GO:0016021//integral component of membrane	-	GO:0055085//transmembrane transport	gi|115433777|ref|XP_001217025.1|/0/predicted protein [Aspergillus terreus NIH2624]

4319526	1209	38.12	0.46	-3.697631652	4.36E-05	0.00023535	Down	ATEG_10088	hypothetical protein	ko01100//Metabolic pathways;ko01110//Biosynthesis of secondary metabolites;ko00350//Tyrosine metabolism;ko00740//Riboflavin metabolism	-	GO:0004097//catechol oxidase activity;GO:0004503//monophenol monooxygenase activity;GO:0046872//metal ion binding	GO:0055114//oxidation-reduction process	gi|115385687|ref|XP_001209390.1|/0/conserved hypothetical protein [Aspergillus terreus NIH2624]
4353879	1506	75.64	2.26	-3.696087478	3.36E-07	2.50E-06	Down	ATEG_09161	hypothetical protein	-	GO:0016021//integral component of membrane	-	GO:0055085//transmembrane transport	gi|115437330|ref|XP_001217783.1|/0/conserved hypothetical protein [Aspergillus terreus NIH2624]
4321696	2560	646.82	30.51	-3.677784873	3.69E-34	1.44E-32	Down	ATEG_06110	hypothetical protein	-	-	-	-	gi|115399398|ref|XP_001215288.1|/6.01038e-52/conserved hypothetical protein [Aspergillus terreus NIH2624]
4320192	3153	28	0	-3.675374897	0.00014385	0.00070228	Down	ATEG_04380	hypothetical protein	ko04120//Ubiquitin mediated proteolysis	-	GO:0003950//NAD+ ADP-ribosyltransferase activity	-	gi|115395838|ref|XP_001213558.1|/0/predicted protein [Aspergillus terreus NIH2624]
4355561	2847	65.01	1.8	-3.668834411	1.39E-06	9.42E-06	Down	ATEG_00800	hypothetical protein	ko01100//Metabolic pathways;ko00500//Starch and sucrose metabolism	GO:0016020//membrane	-	-	gi|115492517|ref|XP_001210886.1|/0/predicted protein [Aspergillus terreus NIH2624]
4317485	1212	107.12	3.55	-3.663003022	6.00E-08	4.87E-07	Down	ATEG_03441	hypothetical protein	ko01100//Metabolic pathways;ko00350//Tyrosine metabolism	-	GO:0004497//monooxygenase activity;GO:0071949//FAD binding	GO:0044550//secondary metabolite biosynthetic process;GO:0055114//oxidation-reduction process	gi|115390228|ref|XP_001212619.1|/0/conserved hypothetical protein [Aspergillus terreus NIH2624]

4318940	1431	167.53	6.66	-3.659214619	2.16E-11	2.45E-10	Down	ATEG_07324	hypothetical protein	-	-	GO:0004497//monooxygenase activity;GO:0005506//iron ion binding;GO:0016705//oxidoreductase activity, acting on paired donors, with incorporation or reduction of molecular oxygen;GO:0020037//heme binding	GO:0055114//oxidation-reduction process	gi|115386938|ref|XP_001210010.1|/0/predicted protein [Aspergillus terreus NIH2624]
4323435	1182	171.51	7.2	-3.647142421	2.18E-12	2.67E-11	Down	ATEG_08630	hypothetical protein	ko01100//Metabolic pathways;ko00564//Glycerophospholipid metabolism;ko00565//Ether lipid metabolism	GO:0016021//integral component of membrane	-	-	gi|115402279|ref|XP_001217216.1|/0/conserved hypothetical protein [Aspergillus terreus NIH2624]
4322332	654	36.99	0.44	-3.644772931	5.85E-05	0.00030689	Down	ATEG_06794	hypothetical protein	-	GO:0005634//nucleus	GO:0003677//DNA binding	GO:0006355//regulation of transcription, DNA-templated;GO:0045122//aflatoxin biosynthetic process	gi|115400767|ref|XP_001215972.1|/5.185e-154/conserved hypothetical protein [Aspergillus terreus NIH2624]
4354496	1011	950.13	46.94	-3.644241725	2.98E-42	1.57E-40	Down	ATEG_09722	hypothetical protein	ko01100//Metabolic pathways;ko00500//Starch and sucrose metabolism	-	-	-	gi|115443074|ref|XP_001218344.1|/0/conserved hypothetical protein [Aspergillus terreus NIH2624]
4322342	1988	421.8	19.86	-3.64298068	4.60E-22	1.05E-20	Down	ATEG_06557	mitogen-activated protein kinase sty1	ko04011//MAPK signaling pathway - yeast	GO:0005622//intracellular	GO:0004707//MAP kinase activity;GO:0005524//ATP binding	GO:0000165//MAPK cascade	gi|115400293|ref|XP_001215735.1|/0/mitogen-activated protein kinase sty1 [Aspergillus terreus NIH2624]

4317444	4593	136.77	5.47	-3.637024203	6.88E-10	6.72E-09	Down	ATEG_02811	hypothetical protein	ko01100//Metabolic pathways;ko00520//Amino sugar and nucleotide sugar metabolism	-	GO:0004568//chitinase activity;GO:0008061//chitin binding	GO:0005975//carbohydrate metabolic process;GO:0006032//chitin catabolic process	gi|115388968|ref|XP_001211989.1|/0/conserved hypothetical protein [Aspergillus terreus NIH2624]
4323475	1725	67.18	1.82	-3.632501242	4.01E-06	2.54E-05	Down	ATEG_08838	hypothetical protein	ko01100//Metabolic pathways;ko00460//Cyanoamino acid metabolism;ko00480//Glutathione metabolism;ko00430//Taurine and hypotaurine metabolism	GO:0005634//nucleus	GO:0000981//RNA polymerase II transcription factor activity, sequence-specific DNA binding;GO:0008270//zinc ion binding	GO:0006357//regulation of transcription from RNA polymerase II promoter	gi|115402695|ref|XP_001217424.1|/0/predicted protein [Aspergillus terreus NIH2624]
4319597	888	98.58	3.07	-3.620374256	4.80E-07	3.49E-06	Down	ATEG_10047	hypothetical protein	ko01100//Metabolic pathways;ko00561//Glycerolipid metabolism	-	GO:0016787//hydrolase activity	GO:0008152//metabolic process	gi|115385605|ref|XP_001209349.1|/0/conserved hypothetical protein [Aspergillus terreus NIH2624]
4317376	2412	127.85	5.01	-3.608504815	6.49E-09	5.77E-08	Down	ATEG_03342	hypothetical protein	ko04140//Regulation of autophagy	-	-	-	gi|115390030|ref|XP_001212520.1|/0/predicted protein [Aspergillus terreus NIH2624]
4316995	1512	36.96	0.44	-3.60304486	7.99E-05	0.00040882	Down	ATEG_02079	hypothetical protein	-	-	-	-	gi|115387503|ref|XP_001211257.1|/0/predicted protein [Aspergillus terreus NIH2624]
4322035	738	125.57	4.89	-3.595710631	3.08E-09	2.84E-08	Down	ATEG_06551	hypothetical protein	-	-	-	-	gi|115400281|ref|XP_001215729.1|/1.2649e-177/predicted protein [Aspergillus terreus NIH2624]

4322804	1113	56.55	1.38	-3.588914222	1.58E-05	9.10E-05	Down	ATEG_07838	hypothetical protein	ko04011//MAPK signaling pathway - yeast	GO:0016021//integral component of membrane	-	-	gi|115401742|ref|XP_001216459.1|/0/predicted protein [Aspergillus terreus NIH2624]
4322541	5754	300.63	13.37	-3.587159871	2.02E-13	2.63E-12	Down	ATEG_07488	hypothetical protein	-	-	GO:0003824//catalytic activity;GO:0031177//phosphopantetheine binding	GO:0008152//metabolic process	gi|115401042|ref|XP_001216109.1|/0/predicted protein [Aspergillus terreus NIH2624]
4354899	3203	3467.22	129.15	-3.580637	2.10E-07	1.60E-06	Down	ATEG_00142	hypothetical protein	-	-	GO:0016491//oxidoreductase activity;GO:0071949//FAD binding	GO:0055114//oxidation-reduction process	gi|115491201|ref|XP_001210228.1|/0/conserved hypothetical protein [Aspergillus terreus NIH2624]
4315768	1581	167.05	7.13	-3.578909995	3.57E-11	3.95E-10	Down	ATEG_01806	hypothetical protein	ko04146//Peroxisome	GO:0016021//integral component of membrane	-	GO:0055085//transmembrane transport	gi|115385248|ref|XP_001209171.1|/0/conserved hypothetical protein [Aspergillus terreus NIH2624]
4354391	441	25.78	0	-3.571840023	0.00023583	0.00110752	Down	ATEG_09629	hypothetical protein	-	-	GO:0003824//catalytic activity;GO:0030151//molybdenum ion binding;GO:0030170//pyridoxal phosphate binding	-	gi|115442888|ref|XP_001218251.1|/1.06638e-100/predicted protein [Aspergillus terreus NIH2624]
4320450	1254	25.79	0	-3.561745998	0.00024821	0.0011633	Down	ATEG_04468	hypothetical protein	ko04120//Ubiquitin mediated proteolysis	GO:0005634//nucleus	GO:0000981//RNA polymerase II transcription factor activity, sequence-specific DNA binding;GO:0008270//zinc ion binding	GO:0006357//regulation of transcription from RNA polymerase II promoter	gi|115396014|ref|XP_001213646.1|/0/predicted protein [Aspergillus terreus NIH2624]

4317932	2520	28.62	0	-3.559463256	0.00026358	0.00123044	Down	ATEG_03563	hypothetical protein	-	-	GO:0016788//hydrolase activity, acting on ester bonds	GO:0009058//biosynthetic process	gi|115390472|ref|XP_001212741.1|/0/predicted protein [Aspergillus terreus NIH2624]
4316508	4518	183.89	8	-3.556992704	2.78E-11	3.13E-10	Down	ATEG_02010	hypothetical protein	ko03013//RNA transport	-	-	-	gi|115387365|ref|XP_001211188.1|/0/predicted protein [Aspergillus terreus NIH2624]
4353647	1510	96.91	3.55	-3.55380324	1.15E-07	9.01E-07	Down	ATEG_09026	hypothetical protein	-	-	-	-	gi|115436804|ref|XP_001217648.1|/1.08317e-89/predicted protein [Aspergillus terreus NIH2624]
4317683	858	277.5	13.42	-3.535229462	4.95E-16	7.68E-15	Down	ATEG_03490	hypothetical protein	-	-	-	-	gi|115390326|ref|XP_001212668.1|/0/predicted protein [Aspergillus terreus NIH2624]
4353377	972	566.02	28.45	-3.527511647	2.65E-22	6.19E-21	Down	ATEG_08063	hypothetical protein	ko01100//Metabolic pathways;ko04141//Protein processing in endoplasmic reticulum;ko00510//N-Glycan biosynthesis	-	-	-	gi|115433094|ref|XP_001216684.1|/0/conserved hypothetical protein [Aspergillus terreus NIH2624]
4319927	5187	393.37	20.37	-3.524767056	4.73E-22	1.08E-20	Down	ATEG_04457	hypothetical protein	ko01100//Metabolic pathways;ko00520//Amino sugar and nucleotide sugar metabolism	-	GO:0016787//hydrolase activity	-	gi|115395992|ref|XP_001213635.1|/0/conserved hypothetical protein [Aspergillus terreus NIH2624]

4355360	504	424.78	22.15	-3.523477438	4.75E-24	1.20E-22	Down	ATEG_00606	hypothetical protein	-	GO:0016021//integral component of membrane	-	-	gi|115492129|ref|XP_001210692.1|/4.86208e-123/conserved hypothetical protein [Aspergillus terreus NIH2624]
4323176	1629	43.74	0.92	-3.515914548	5.47E-05	0.00028763	Down	ATEG_08914	hypothetical protein	-	-	-	-	gi|115402847|ref|XP_001217500.1|/0/conserved hypothetical protein [Aspergillus terreus NIH2624]
4318119	1373	52.14	1.38	-3.499151893	2.60E-05	0.00014558	Down	ATEG_02802	hypothetical protein	ko03008//Ribosome biogenesis in eukaryotes	-	GO:0003676//nucleic acid binding;GO:0046872//metal ion binding	-	gi|115388950|ref|XP_001211980.1|/0/predicted protein [Aspergillus terreus NIH2624]
4322965	747	72.24	2.19	-3.481088908	7.95E-06	4.79E-05	Down	ATEG_07953	hypothetical protein	-	-	-	-	gi|115401972|ref|XP_001216574.1|/0/predicted protein [Aspergillus terreus NIH2624]
4318233	2211	7911.57	451.72	-3.467010378	9.73E-92	1.90E-89	Down	ATEG_03900	oryzin precursor	-	GO:0005576//extracellular region	GO:0004252//serine-type endopeptidase activity;GO:0042802//identical protein binding;GO:0052689//carboxylic ester hydrolase activity;GO:0070051//fibrinogen binding	GO:0006508//proteolysis;GO:0006956//complement activation;GO:0030163//protein catabolic process;GO:0043086//negative regulation of catalytic activity;GO:0060309//elastin catabolic process	gi|115391147|ref|XP_001213078.1|/0/oryzin precursor [Aspergillus terreus NIH2624]
4322741	1464	24.1	0	-3.464596002	0.0003884	0.00175043	Down	ATEG_07492	hypothetical protein	ko01100//Metabolic pathways;ko00500//Starch and sucrose metabolism	-	-	-	gi|115401050|ref|XP_001216113.1|/0/predicted protein [Aspergillus terreus NIH2624]

4319122	999	23.54	0	-3.429300417	0.00045504	0.00201874	Down	ATEG_07318	hypothetical protein	ko01100//Metabolic pathways;ko00051//Fructose and mannose metabolism;ko00650//Butanoate metabolism;ko00591//Linoleic acid metabolism	-	GO:0008270//zinc ion binding;GO:0016491//oxidoreductase activity	GO:0055114//oxidation-reduction process	gi|115386926|ref|XP_001210004.1|/0/conserved hypothetical protein [Aspergillus terreus NIH2624]
4321898	776	64.48	2.24	-3.424936371	5.85E-06	3.61E-05	Down	ATEG_06413	hypothetical protein	-	-	-	-	gi|115400005|ref|XP_001215591.1|/6.6276e-122/predicted protein [Aspergillus terreus NIH2624]
4354321	588	27.41	0	-3.421742277	0.00049912	0.00219769	Down	ATEG_09821	similar to cleavage and polyadenylation specific factor 5	ko03015//mRNA surveillance pathway	GO:0005849//mRNA cleavage factor complex	GO:0003729//mRNA binding	GO:0006378//mRNA polyadenylation	gi|115443272|ref|XP_001218443.1|/1.77292e-150/hypothetical protein ATEG_09821 [Aspergillus terreus NIH2624]
4316539	618	49.84	1.38	-3.410659656	5.31E-05	0.00027997	Down	ATEG_01934	hypothetical protein	ko03040//Spliceosome	-	-	-	gi|115387213|ref|XP_001211112.1|/4.65136e-151/conserved hypothetical protein [Aspergillus terreus NIH2624]
4320317	708	95.22	4.11	-3.408496777	3.52E-07	2.61E-06	Down	ATEG_04324	hypothetical protein	-	GO:0016021//integral component of membrane	-	-	gi|115395354|ref|XP_001213502.1|/4.27545e-180/predicted protein [Aspergillus terreus NIH2624]
4353171	705	168.72	8.53	-3.407733614	2.21E-11	2.49E-10	Down	ATEG_08147	hypothetical protein	-	-	-	-	gi|115433262|ref|XP_001216768.1|/1.22449e-174/predicted protein [Aspergillus terreus NIH2624]

4321978	4159	467.82	26.55	-3.406325659	1.39E-24	3.63E-23	Down	ATEG_06284	hypothetical protein	ko04146//Peroxisome	GO:0016021//integral component of membrane	-	GO:0055085//transmembrane transport	gi|115399746|ref|XP_001215462.1|/0/conserved hypothetical protein [Aspergillus terreus NIH2624]
4316852	1421	67.33	2.26	-3.404150704	1.40E-05	8.14E-05	Down	ATEG_02225	hypothetical protein	-	GO:0016021//integral component of membrane	GO:0016491//oxidoreductase activity	GO:0055114//oxidation-reduction process	gi|115387795|ref|XP_001211403.1|/0/conserved hypothetical protein [Aspergillus terreus NIH2624]
4320159	543	77.33	3.14	-3.39672491	9.66E-07	6.64E-06	Down	ATEG_04916	hypothetical protein	ko01100//Metabolic pathways;ko00051//Fructose and mannose metabolism;ko00650//Butanoate metabolism;ko00591//Linoleic acid metabolism	-	-	-	gi|115396910|ref|XP_001214094.1|/5.11467e-120/predicted protein [Aspergillus terreus NIH2624]
4322573	2511	12815.21	781.94	-3.390244191	9.98E-114	2.84E-111	Down	ATEG_07477	catalase B precursor	ko01110//Biosynthesis of secondary metabolites;ko01130//Biosynthesis of antibiotics;ko01200//Carbon metabolism;ko04011//MAPK signaling pathway - yeast;ko04146//Peroxisome;ko00380//Tryptophan metabolism;ko00630//Glyoxylate and dicarboxylate metabolism	GO:0005576//extracellular region;GO:0005622//intracellular;GO:0009986//cell surface	GO:0004096//catalase activity;GO:0020037//heme binding;GO:0046872//metal ion binding	GO:0042744//hydrogen peroxide catabolic process;GO:0055114//oxidation-reduction process;GO:0070301//cellular response to hydrogen peroxide;GO:0098869//cellular oxidant detoxification	gi|115401020|ref|XP_001216098.1|/0/catalase B precursor [Aspergillus terreus NIH2624]

4318416	588	285.77	15.9	-3.387121822	1.42E-15	2.12E-14	Down	ATEG_04073	hypothetical protein	ko03013//RNA transport	-	-	-	gi|115391493|ref|XP_001213251.1|/5.45523e-136/predicted protein [Aspergillus terreus NIH2624]
4321290	744	22.98	0	-3.386335081	0.00055075	0.00239577	Down	ATEG_05994	hypothetical protein	-	GO:0016021//integral component of membrane	-	-	gi|115399166|ref|XP_001215172.1|/0/predicted protein [Aspergillus terreus NIH2624]
4318599	660	48.67	0.88	-3.382692927	0.0002072	0.00098438	Down	ATEG_03814	hypothetical protein	-	-	-	-	gi|115390975|ref|XP_001212992.1|/1.32203e-157/conserved hypothetical protein [Aspergillus terreus NIH2624]
4322564	675	450.85	25.62	-3.375583437	1.97E-19	3.89E-18	Down	ATEG_07468	hypothetical protein	ko04011//MAPK signaling pathway - yeast	GO:0016021//integral component of membrane	-	-	gi|115401002|ref|XP_001216089.1|/1.52967e-154/predicted protein [Aspergillus terreus NIH2624]
4320318	1500	172.61	8.95	-3.373994722	2.06E-11	2.34E-10	Down	ATEG_04325	hypothetical protein	-	GO:0016021//integral component of membrane	-	GO:0055085//transmembrane transport	gi|115395356|ref|XP_001213503.1|/0/predicted protein [Aspergillus terreus NIH2624]
4318172	2678	583.59	34.44	-3.364891733	9.92E-24	2.46E-22	Down	ATEG_04054	hypothetical protein	-	GO:0016021//integral component of membrane	GO:0046873//metal ion transmembrane transporter activity	GO:0030001//metal ion transport;GO:0055085//transmembrane transport	gi|115391455|ref|XP_001213232.1|/0/predicted protein [Aspergillus terreus NIH2624]
4321906	804	253.2	13.86	-3.357066777	5.39E-14	7.30E-13	Down	ATEG_06759	hypothetical protein	ko01100//Metabolic pathways;ko00520//Amino sugar and nucleotide sugar metabolism	-	-	-	gi|115400697|ref|XP_001215937.1|/0/predicted protein [Aspergillus terreus NIH2624]

4354894	1023	4442.76	193.21	-3.34752986	2.61E-06	1.71E-05	Down	ATEG_00137	hypothetical protein	ko00620//Pyruvate metabolism;ko00640//Propanoate metabolism	GO:0016021//integral component of membrane	GO:0003824//catalytic activity;GO:0050662//coenzyme binding	-	gi|115491191|ref|XP_001210223.1|/0/conserved hypothetical protein [Aspergillus terreus NIH2624]
4354260	1395	22.99	0	-3.345563282	0.00066289	0.00283368	Down	ATEG_09803	hypothetical protein	ko01100//Metabolic pathways;ko04122//Sulfur relay system;ko00730//Thiamine metabolism	-	GO:0016614//oxidoreductase activity, acting on CH-OH group of donors;GO:0050660//flavin adenine dinucleotide binding	GO:0055114//oxidation-reduction process	gi|115443236|ref|XP_001218425.1|/0/predicted protein [Aspergillus terreus NIH2624]
4321979	762	22.41	0	-3.340110715	0.00067383	0.00286869	Down	ATEG_06285	similar to pectate lyase C	ko00040//Pentose and glucuronate interconversions	GO:0005576//extracellular region	GO:0030570//pectate lyase activity	GO:0000272//polysaccharide catabolic process;GO:0071555//cell wall organization	gi|115399748|ref|XP_001215463.1|/0/hypothetical protein ATEG_06285 [Aspergillus terreus NIH2624]
4318664	990	77.36	3.09	-3.325444791	4.24E-06	2.68E-05	Down	ATEG_04110	hypothetical protein	-	GO:0016021//integral component of membrane	-	GO:0006950//response to stress	gi|115391567|ref|XP_001213288.1|/0/conserved hypothetical protein [Aspergillus terreus NIH2624]
4354900	1165	2032.24	92.18	-3.325049739	1.75E-06	1.17E-05	Down	ATEG_00143	hypothetical protein	-	-	GO:0016491//oxidoreductase activity;GO:0071949//FAD binding	GO:0055114//oxidation-reduction process	gi|115491203|ref|XP_001210229.1|/0/predicted protein [Aspergillus terreus NIH2624]
4321224	3146	243.67	13.35	-3.320566837	2.31E-12	2.83E-11	Down	ATEG_04988	hypothetical protein	-	GO:0016021//integral component of membrane	GO:0022891//substrate-specific transmembrane transporter activity	GO:0055085//transmembrane transport	gi|115397149|ref|XP_001214166.1|/0/conserved hypothetical protein [Aspergillus terreus NIH2624]

4316130	861	341.79	20.16	-3.313371896	1.48E-18	2.69E-17	Down	ATEG_01719	hypothetical protein	ko04011//MAPK signaling pathway - yeast	GO:0016021//integral component of membrane	-	-	gi|115385074|ref|XP_001209084.1|/0/predicted protein [Aspergillus terreus NIH2624]
4322243	789	21.85	0	-3.311066337	0.00076045	0.00319109	Down	ATEG_06706	hypothetical protein	ko04011//MAPK signaling pathway - yeast	GO:0016021//integral component of membrane	-	-	gi|115400591|ref|XP_001215884.1|/0/predicted protein [Aspergillus terreus NIH2624]
4323570	2041	1012.9	63.5	-3.310734022	1.18E-40	5.94E-39	Down	ATEG_08629	hypothetical protein	-	GO:0016021//integral component of membrane	GO:0015171//amino acid transmembrane transporter activity	GO:0003333//amino acid transmembrane transport	gi|115402277|ref|XP_001217215.1|/0/conserved hypothetical protein [Aspergillus terreus NIH2624]
4321274	1101	71.7	2.63	-3.302421272	1.71E-05	9.84E-05	Down	ATEG_06124	hypothetical protein	ko04111//Cell cycle - yeast	GO:0016021//integral component of membrane	-	-	gi|115399426|ref|XP_001215302.1|/0/conserved hypothetical protein [Aspergillus terreus NIH2624]
4354068	1131	22.95	0	-3.300480637	0.00080959	0.0033712	Down	ATEG_09117	Delta(14)-sterol reductase	ko01100//Metabolic pathways;ko01110//Biosynthesis of secondary metabolites;ko01130//Biosynthesis of antibiotics;ko00100//Steroid biosynthesis	GO:0016021//integral component of membrane	GO:0016628//oxidoreductase activity, acting on the CH-CH group of donors, NAD or NADP as acceptor	GO:0055114//oxidation-reduction process	gi|115437154|ref|XP_001217739.1|/0/Delta(14)-sterol reductase [Aspergillus terreus NIH2624]
4319400	960	191.56	10.21	-3.294158697	4.76E-10	4.71E-09	Down	ATEG_10019	hypothetical protein	ko01100//Metabolic pathways;ko04122//Sulfur relay system;ko00730//Thiamine metabolism	GO:0016021//integral component of membrane	-	-	gi|115385549|ref|XP_001209321.1|/0/predicted protein [Aspergillus terreus NIH2624]

4355525	2903	5901.29	383.49	-3.293162279	2.26E-87	3.85E-85	Down	ATEG_00770	hypothetical protein	ko01100//Metabolic pathways;ko00500//Starch and sucrose metabolism	GO:0005634//nucleus	GO:0000981//RNA polymerase II transcription factor activity, sequence-specific DNA binding;GO:0008270//zinc ion binding	GO:0006357//regulation of transcription from RNA polymerase II promoter	gi|115492457|ref|XP_001210856.1|/0/conserved hypothetical protein [Aspergillus terreus NIH2624]
4319437	2637	890.93	56.85	-3.259441891	3.70E-21	8.13E-20	Down	ATEG_10003	hypothetical protein	ko01100//Metabolic pathways;ko00500//Starch and sucrose metabolism	GO:0016021//integral component of membrane	GO:0008484//sulfuric ester hydrolase activity	GO:0008152//metabolic process	gi|115385517|ref|XP_001209305.1|/0/conserved hypothetical protein [Aspergillus terreus NIH2624]
4321832	1539	471.58	29.22	-3.257822429	5.95E-19	1.11E-17	Down	ATEG_05898	similar to monooxygenase	ko01100//Metabolic pathways;ko01110//Biosynthesis of secondary metabolites;ko00403//Indole diterpene alkaloid biosynthesis	-	GO:0016491//oxidoreductase activity;GO:0071949//FAD binding	GO:0055114//oxidation-reduction process	gi|115398974|ref|XP_001215076.1|/0/hypothetical protein ATEG_05898 [Aspergillus terreus NIH2624]
4322892	894	28.58	0.46	-3.253068567	0.0005102	0.00223909	Down	ATEG_07947	hypothetical protein	-	GO:0016021//integral component of membrane	-	-	gi|115401960|ref|XP_001216568.1|/0/conserved hypothetical protein [Aspergillus terreus NIH2624]
4354893	594	1587.53	78.96	-3.248718238	1.49E-06	1.01E-05	Down	ATEG_00136	hypothetical protein	-	-	-	-	gi|115491189|ref|XP_001210222.1|/5.06166e-142/predicted protein [Aspergillus terreus NIH2624]
4320707	1452	20.73	0	-3.229998453	0.00106337	0.00431931	Down	ATEG_05202	hypothetical protein	-	-	-	-	gi|115397577|ref|XP_001214380.1|/0/predicted protein [Aspergillus terreus NIH2624]

4354842	1182	1683.53	97.8	-3.227399696	2.26E-09	2.12E-08	Down	ATEG_00085	hypothetical protein	ko01100//Metabolic pathways;ko00500//Starch and sucrose metabolism	GO:0016021//integral component of membrane	-	GO:0055085//transmembrane transport	gi|115491087|ref|XP_001210171.1|/0/conserved hypothetical protein [Aspergillus terreus NIH2624]
4316446	1224	36.41	0.88	-3.226249109	0.00029842	0.00138002	Down	ATEG_01891	hypothetical protein	-	-	GO:0003677//DNA binding;GO:0046983//protein dimerization activity	-	gi|115387127|ref|XP_001211069.1|/0/predicted protein [Aspergillus terreus NIH2624]
4354127	462	368.25	23.63	-3.219464489	1.06E-16	1.72E-15	Down	ATEG_09725	hypothetical protein	ko04144//Endocytosis	-	-	-	gi|115443080|ref|XP_001218347.1|/3.25886e-112/predicted protein [Aspergillus terreus NIH2624]
4320008	1911	194.4	11.69	-3.218351261	8.26E-12	9.69E-11	Down	ATEG_04459	hypothetical protein	ko01100//Metabolic pathways;ko00500//Starch and sucrose metabolism	-	-	-	gi|115395996|ref|XP_001213637.1|/0/predicted protein [Aspergillus terreus NIH2624]
4318827	6654	68.95	3.14	-3.217372019	6.47E-06	3.96E-05	Down	ATEG_07067	similar to polyketide synthase	-	-	GO:0016491//oxidoreductase activity;GO:0016740//transferase activity;GO:0031177//phosphopantetheine binding	GO:0055114//oxidation-reduction process	gi|115386424|ref|XP_001209753.1|/0/hypothetical protein ATEG_07067 [Aspergillus terreus NIH2624]
4322865	1062	545.75	36.54	-3.196686142	3.98E-26	1.14E-24	Down	ATEG_07542	hypothetical protein	-	-	-	-	gi|115401150|ref|XP_001216163.1|/0/predicted protein [Aspergillus terreus NIH2624]
4317837	948	35.3	0.88	-3.186991728	0.00036459	0.00165585	Down	ATEG_03103	hypothetical protein	-	-	-	-	gi|115389552|ref|XP_001212281.1|/4.35796e-60/conserved hypothetical protein [Aspergillus terreus NIH2624]

4320987	522	400.72	26.52	-3.184327575	5.50E-21	1.19E-19	Down	ATEG_04982	hypothetical protein	-	-	-	-	gi|115397137|ref|XP_001214160.1|/1.00745e-118/predicted protein [Aspergillus terreus NIH2624]
4353097	877	20.19	0	-3.163248401	0.00139359	0.00551035	Down	ATEG_08447	hypothetical protein	-	-	GO:0008171//O-methyltransferase activity;GO:0046872//metal ion binding;GO:0051539//4 iron, 4 sulfur cluster binding	GO:0032259//methylation	gi|115433863|ref|XP_001217068.1|/1.98161e-85/conserved hypothetical protein [Aspergillus terreus NIH2624]
4353882	1767	60.5	2.75	-3.14758259	3.67E-05	0.0002005	Down	ATEG_09153	hypothetical protein	-	-	-	-	gi|115437298|ref|XP_001217775.1|/0/conserved hypothetical protein [Aspergillus terreus NIH2624]
4316279	882	147.99	8.95	-3.137993012	3.45E-09	3.18E-08	Down	ATEG_01767	hypothetical protein	ko04011//MAPK signaling pathway - yeast	GO:0016021//integral component of membrane	-	-	gi|115385170|ref|XP_001209132.1|/0/predicted protein [Aspergillus terreus NIH2624]
4354125	1059	19.61	0	-3.137510365	0.00153698	0.00603157	Down	ATEG_09764	hypothetical protein	ko01100//Metabolic pathways;ko00500//Starch and sucrose metabolism	GO:0016021//integral component of membrane	GO:0016757//transferase activity, transferring glycosyl groups	-	gi|115443158|ref|XP_001218386.1|/0/predicted protein [Aspergillus terreus NIH2624]
4353893	1542	26.34	0.46	-3.126085301	0.00093651	0.00384563	Down	ATEG_09351	hypothetical protein	ko01100//Metabolic pathways;ko01110//Biosynthesis of secondary metabolites;ko00100//Steroid biosynthesis	-	-	-	gi|115438076|ref|XP_001217973.1|/0/predicted protein [Aspergillus terreus NIH2624]
4318632	1617	77.87	4.01	-3.120369219	4.90E-06	3.06E-05	Down	ATEG_03823	hypothetical protein	-	-	GO:0050151//oleate hydratase activity;GO:0071949//FAD binding	GO:0006631//fatty acid metabolic process	gi|115390993|ref|XP_001213001.1|/0/predicted protein [Aspergillus terreus NIH2624]

4318414	1824	93.09	4.89	-3.119249826	2.85E-06	1.86E-05	Down	ATEG_04071	siderophore iron transporter mirA	-	GO:0016021//integral component of membrane	-	GO:0055085//transmembrane transport	gi|115391489|ref|XP_001213249.1|/0/siderophore iron transporter mirA [Aspergillus terreus NIH2624]
4317793	606	21.26	0	-3.101628058	0.00180271	0.00695412	Down	ATEG_03140	hypothetical protein	-	-	-	-	gi|115389626|ref|XP_001212318.1|/7.45086e-146/conserved hypothetical protein [Aspergillus terreus NIH2624]
4353841	510	195.02	13.08	-3.088460513	1.35E-11	1.56E-10	Down	ATEG_09138	hypothetical protein	-	-	-	-	gi|115437238|ref|XP_001217760.1|/1.69155e-124/conserved hypothetical protein [Aspergillus terreus NIH2624]
4317788	864	219.08	14.76	-3.074802814	4.87E-12	5.82E-11	Down	ATEG_03135	hypothetical protein	-	GO:0016021//integral component of membrane	-	GO:0006950//response to stress	gi|115389616|ref|XP_001212313.1|/0/conserved hypothetical protein [Aspergillus terreus NIH2624]
4355663	4233	510.57	37.41	-3.067254066	3.49E-24	8.90E-23	Down	ATEG_00902	5-oxoprolinase	ko00480//Glutathione metabolism	-	GO:0016787//hydrolase activity	-	gi|115492721|ref|XP_001210988.1|/0/5-oxoprolinase [Aspergillus terreus NIH2624]
4318851	1167	344.16	24.1	-3.058413073	2.84E-15	4.17E-14	Down	ATEG_07115	hypothetical protein	ko01100//Metabolic pathways;ko00350//Tyrosine metabolism	-	GO:0016491//oxidoreductase activity;GO:0071949//FAD binding	GO:0055114//oxidation-reduction process	gi|115386520|ref|XP_001209801.1|/0/predicted protein [Aspergillus terreus NIH2624]
4319512	2143	423.03	29.86	-3.056597943	1.30E-16	2.11E-15	Down	ATEG_09992	hypothetical protein	-	GO:0016021//integral component of membrane	-	GO:0055085//transmembrane transport	gi|115385495|ref|XP_001209294.1|/0/conserved hypothetical protein [Aspergillus terreus NIH2624]

4321852	2034	44.8	1.78	-3.054062183	0.00022432	0.00105607	Down	ATEG_05646	hypothetical protein	ko01100//Metabolic pathways;ko00330//Arginine and proline metabolism	GO:0016021//integral component of membrane	-	-	gi|115398470|ref|XP_001214824.1|/0/predicted protein [Aspergillus terreus NIH2624]
4322890	1554	19.04	0	-3.047404887	0.00217689	0.00822954	Down	ATEG_07945	hypothetical protein	ko04111//Cell cycle - yeast	-	GO:0004497//monooxygenase activity;GO:0005506//iron ion binding;GO:0009055//electron carrier activity;GO:0016705//oxidoreductase activity, acting on paired donors, with incorporation or reduction of molecular oxygen;GO:0020037//heme binding;GO:0042469	GO:0010914;GO:0055114//oxidation-reduction process	gi|115401956|ref|XP_001216566.1|/0/conserved hypothetical protein [Aspergillus terreus NIH2624]
4317616	3540	30.83	0.9	-3.040710511	0.00073475	0.00309987	Down	ATEG_03496	hypothetical protein	-	-	GO:0003824//catalytic activity	GO:0009116//nucleoside metabolic process	gi|115390338|ref|XP_001212674.1|/0/conserved hypothetical protein [Aspergillus terreus NIH2624]
4353333	2361	19.07	0	-3.028604514	0.00233749	0.00875557	Down	ATEG_07977	hypothetical protein	-	-	GO:0003723//RNA binding	-	gi|115432922|ref|XP_001216598.1|/0/conserved hypothetical protein [Aspergillus terreus NIH2624]

4317677	1169	73.99	3.5	-3.02541208	7.25E-05	0.00037464	Down	ATEG_03444	hypothetical protein	ko01100//Metabolic pathways;ko01220//Degradation of aromatic compounds	-	GO:0005506//iron ion binding;GO:0016705//oxidoreductase activity, acting on paired donors, with incorporation or reduction of molecular oxygen;GO:0020037//heme binding	GO:0055114//oxidation-reduction process	gi|115390234|ref|XP_001212622.1|/0/predicted protein [Aspergillus terreus NIH2624]
4320847	2966	82.92	4.94	-3.010545054	3.51E-06	2.26E-05	Down	ATEG_05169	hypothetical protein	-	-	GO:0004553//hydrolase activity, hydrolyzing O-glycosyl compounds;GO:0030246//carbohydrate binding	GO:0005975//carbohydrate metabolic process	gi|115397511|ref|XP_001214347.1|/0/predicted protein [Aspergillus terreus NIH2624]
4354821	3153	48.21	2.24	-3.000243117	0.00015443	0.00074961	Down	ATEG_00065	hypothetical protein	ko00380//Tryptophan metabolism;ko00071//Fatty acid degradation	-	GO:0003958//NADPH-hemoprotein reductase activity;GO:0005506//iron ion binding;GO:0010181//FMN binding;GO:0020037//heme binding;GO:0070330//aromatase activity	GO:0055114//oxidation-reduction process	gi|115491047|ref|XP_001210151.1|/0/predicted protein [Aspergillus terreus NIH2624]
4321240	1755	245.66	2.77	-2.993460391	0.00258914	0.00957584	Down	ATEG_05074	similar to acyl-CoA synthase	ko01100//Metabolic pathways;ko04146//Peroxisome;ko00071//Fatty acid degradation;ko01212//Fatty acid metabolism;ko00061//Fatty acid biosynthesis	-	GO:0003824//catalytic activity	GO:0008152//metabolic process	gi|115397321|ref|XP_001214252.1|/0/hypothetical protein ATEG_05074 [Aspergillus terreus NIH2624]

4320089	6286	808.97	62.32	-2.992369361	3.90E-26	1.12E-24	Down	ATEG_04555	hypothetical protein	ko03018//RNA degradation	GO:0005634//nucleus;GO:0005829//cytosol;GO:0030015//CCR4-NOT core complex	-	GO:0000289//nuclear-transcribed mRNA poly(A) tail shortening;GO:0000749//response to pheromone involved in conjugation with cellular fusion;GO:0007124//pseudohyphal growth;GO:0032968//positive regulation of transcription elongation from RNA polymerase II promoter;GO:0051726//regulation of cell cycle	gi|115396188|ref|XP_001213733.1|/0/conserved hypothetical protein [Aspergillus terreus NIH2624]
4322586	1926	1173.34	90.5	-2.988258139	5.19E-25	1.40E-23	Down	ATEG_07927	hypothetical protein	-	-	GO:0004499//N,N-dimethylaniline monooxygenase activity;GO:0050660//flavin adenine dinucleotide binding;GO:0050661//NADP binding	GO:0055114//oxidation-reduction process	gi|115401920|ref|XP_001216548.1|/0/predicted protein [Aspergillus terreus NIH2624]
4315946	3159	2460.6	199.73	-2.968902774	9.84E-61	9.05E-59	Down	ATEG_01202	hypothetical protein	-	GO:0016021//integral component of membrane	-	GO:0055085//transmembrane transport	gi|115384040|ref|XP_001208567.1|/0/conserved hypothetical protein [Aspergillus terreus NIH2624]

4316169	1011	42.01	1.78	-2.965308062	0.00037793	0.00170981	Down	ATEG_01140	hypothetical protein	ko01100//Metabolic pathways;ko01110//Biosynthesis of secondary metabolites;ko01130//Biosynthesis of antibiotics;ko00650//Butanoate metabolism;ko00380//Tryptophan metabolism;ko00071//Fatty acid degradation;ko00310//Lysine degradation;ko00280//Valine, leucine and isoleucine degradation;ko01212//Fatty acid metabolism;ko00062//Fatty acid elongation	-	GO:0003857//3-hydroxyacyl-CoA dehydrogenase activity;GO:0070403//NAD+ binding	GO:0006631//fatty acid metabolic process;GO:0055114//oxidation-reduction process	gi|115383916|ref|XP_001208505.1|/0/conserved hypothetical protein [Aspergillus terreus NIH2624]	
4353424	1734	17.37	0	-2.954223283	0.00303707	0.01101965	Down	ATEG_08087	hypothetical protein	ko01100//Metabolic pathways;ko00500//Starch and sucrose metabolism	-	-	-	gi|115433142|ref|XP_001216708.1|/0/predicted protein [Aspergillus terreus NIH2624]	

4322467	1560	17.37	0	-2.954085879	0.00303854	0.01102065	Down	ATEG_06678	similar to n-alkane-inducible cytochrome P-450	ko00380//Tryptophan metabolism;ko00071//Fatty acid degradation	-	GO:0005506//iron ion binding;GO:0016712//oxidoreductase activity, acting on paired donors, with incorporation or reduction of molecular oxygen, reduced flavin or flavoprotein as one donor, and incorporation of one atom of oxygen;GO:0020037//heme binding	GO:0055114//oxidation-reduction process	gi|115400535|ref|XP_001215856.1|/0/hypothetical protein ATEG_06678 [Aspergillus terreus NIH2624]
4318927	2174	96.93	6.37	-2.954001303	2.00E-06	1.33E-05	Down	ATEG_07097	hypothetical protein	-	GO:0016021//integral component of membrane	-	GO:0055085//transmembrane transport	gi|115386484|ref|XP_001209783.1|/0/predicted protein [Aspergillus terreus NIH2624]
4316830	1386	89.1	5.3	-2.949489505	1.06E-05	6.27E-05	Down	ATEG_02388	hypothetical protein	-	GO:0016021//integral component of membrane	-	-	gi|115388121|ref|XP_001211566.1|/0/predicted protein [Aspergillus terreus NIH2624]
4319632	1020	24.08	0.44	-2.948888905	0.00205836	0.00782245	Down	ATEG_10206	hypothetical protein	ko01100//Metabolic pathways;ko00520//Amino sugar and nucleotide sugar metabolism	GO:0016021//integral component of membrane	-	GO:0006950//response to stress	gi|115385923|ref|XP_001209508.1|/0/predicted protein [Aspergillus terreus NIH2624]
4322832	714	568.32	45.44	-2.947354967	7.44E-25	1.99E-23	Down	ATEG_07481	hypothetical protein	ko04144//Endocytosis	-	-	-	gi|115401028|ref|XP_001216102.1|/1.37449e-177/predicted protein [Aspergillus terreus NIH2624]

4321047	624	17.95	0	-2.946495055	0.00313789	0.01134599	Down	ATEG_05354	hypothetical protein	-	-	-	-	gi|115397881|ref|XP_001214532.1|/8.71524e-157/predicted protein [Aspergillus terreus NIH2624]
4321149	1188	280.16	21.64	-2.946299123	2.55E-14	3.54E-13	Down	ATEG_05007	hypothetical protein	-	GO:0016021//integral component of membrane	-	-	gi|115397187|ref|XP_001214185.1|/0/conserved hypothetical protein [Aspergillus terreus NIH2624]
4317197	1278	42.6	1.85	-2.941596597	0.00056032	0.00243179	Down	ATEG_02717	hypothetical protein	ko01100//Metabolic pathways;ko01220//Degradation of aromatic compounds	-	GO:0016491//oxidoreductase activity;GO:0071949//FAD binding	GO:0055114//oxidation-reduction process	gi|115388779|ref|XP_001211895.1|/0/conserved hypothetical protein [Aspergillus terreus NIH2624]
4316405	1434	74.58	4.55	-2.932091792	2.38E-05	0.00013423	Down	ATEG_01698	hypothetical protein	ko01100//Metabolic pathways;ko01110//Biosynthesis of secondary metabolites;ko00860//Porphyrin and chlorophyll metabolism	GO:0016021//integral component of membrane	GO:0046873//metal ion transmembrane transporter activity	GO:0030001//metal ion transport;GO:0055085//transmembrane transport	gi|115385032|ref|XP_001209063.1|/0/predicted protein [Aspergillus terreus NIH2624]
4317681	640	24.68	0.44	-2.930086022	0.00229414	0.00860986	Down	ATEG_03448	hypothetical protein	-	-	-	-	gi|115390242|ref|XP_001212626.1|/1.25344e-94/conserved hypothetical protein [Aspergillus terreus NIH2624]
4317230	1422	466.72	36.62	-2.92998303	9.91E-18	1.71E-16	Down	ATEG_02688	hypothetical protein	-	-	GO:0016491//oxidoreductase activity	GO:0055114//oxidation-reduction process	gi|115388721|ref|XP_001211866.1|/0/predicted protein [Aspergillus terreus NIH2624]

4317813	714	357.49	28.51	-2.927649129	3.58E-16	5.64E-15	Down	ATEG_03359	similar to theta class glutathione S-transferase	ko00480//Glutathione metabolism	-	-	-	gi|115390064|ref|XP_001212537.1|/4.60156e-176/hypothetical protein ATEG_03359 [Aspergillus terreus NIH2624]
4317762	765	217.46	16.68	-2.926467607	8.22E-12	9.66E-11	Down	ATEG_02816	hypothetical protein	-	-	-	-	gi|115388978|ref|XP_001211994.1|/0/predicted protein [Aspergillus terreus NIH2624]
4355627	1557	2468.05	206.76	-2.925429947	8.60E-69	1.03E-66	Down	ATEG_00866	hypothetical protein	ko04144//Endocytosis	-	-	-	gi|115492649|ref|XP_001210952.1|/0/conserved hypothetical protein [Aspergillus terreus NIH2624]
4321463	1740	73.43	4.15	-2.925089255	0.00011993	0.00059256	Down	ATEG_05825	sphingolipid long chain base-responsive protein PIL1	ko01100//Metabolic pathways;ko01110//Biosynthesis of secondary metabolites;ko00130//Ubiquinone and other terpenoid-quinone biosynthesis	GO:0016021//integral component of membrane;GO:0035974//meiotic spindle pole body;GO:0070056//prospore membrane leading edge;GO:0070057//prospore membrane spindle pole body attachment site	-	GO:0031322//ascospore-type prospore-specific spindle pole body remodeling	gi|115398828|ref|XP_001215003.1|/0/sphingolipid long chain base-responsive protein PIL1 [Aspergillus terreus NIH2624]
4321150	1584	95.21	5.74	-2.921056268	1.62E-05	9.33E-05	Down	ATEG_05008	hypothetical protein	-	GO:0016021//integral component of membrane	GO:0022857//transmembrane transporter activity	GO:0008643//carbohydrate transport;GO:0055085//transmembrane transport	gi|115397189|ref|XP_001214186.1|/0/conserved hypothetical protein [Aspergillus terreus NIH2624]
4317207	1254	29.72	0.92	-2.911611607	0.00158171	0.00618382	Down	ATEG_02714	hypothetical protein	ko03008//Ribosome biogenesis in eukaryotes	-	GO:0003676//nucleic acid binding	-	gi|115388773|ref|XP_001211892.1|/0/predicted protein [Aspergillus terreus NIH2624]

4323012	8052	1241.75	104.51	-2.907093268	1.24E-43	6.85E-42	Down	ATEG_07659	similar to polyketide synthase	-	-	GO:0016491//oxidoreductase activity;GO:0016740//transferase activity;GO:0031177//phosphopantetheine binding	GO:0055114//oxidation-reduction process;GO:1900554	gi|115401384|ref|XP_001216280.1|/0/hypothetical protein ATEG_07659 [Aspergillus terreus NIH2624]
4355635	1299	22.98	0.46	-2.906477802	0.00245388	0.00914044	Down	ATEG_00874	hypothetical protein	ko01100//Metabolic pathways;ko01220//Degradation of aromatic compounds	-	GO:0016491//oxidoreductase activity;GO:0071949//FAD binding	GO:0055114//oxidation-reduction process	gi|115492665|ref|XP_001210960.1|/0/predicted protein [Aspergillus terreus NIH2624]
4355516	888	70	3.5	-2.903925127	0.00022854	0.00107489	Down	ATEG_00761	hypothetical protein	ko03040//Spliceosome	GO:0000974//Prp19 complex	-	-	gi|115492439|ref|XP_001210847.1|/0/conserved hypothetical protein [Aspergillus terreus NIH2624]
4322352	1545	39.81	1.8	-2.898914014	0.00054827	0.00238721	Down	ATEG_06643	hypothetical protein	ko01100//Metabolic pathways;ko00500//Starch and sucrose metabolism;ko00520//Amino sugar and nucleotide sugar metabolism	-	GO:0004553//hydrolase activity, hydrolyzing O-glycosyl compounds	GO:0005975//carbohydrate metabolic process	gi|115400465|ref|XP_001215821.1|/0/conserved hypothetical protein [Aspergillus terreus NIH2624]
4317532	1986	330.59	27.01	-2.883242504	6.48E-16	9.98E-15	Down	ATEG_03454	hypothetical protein	-	GO:0016021//integral component of membrane	GO:0016491//oxidoreductase activity	GO:0055114//oxidation-reduction process	gi|115390254|ref|XP_001212632.1|/0/conserved hypothetical protein [Aspergillus terreus NIH2624]
4317313	1458	88	5.79	-2.881788415	6.86E-06	4.18E-05	Down	ATEG_02654	hypothetical protein	-	-	GO:0046872//metal ion binding	GO:0006355//regulation of transcription, DNA-templated	gi|115388653|ref|XP_001211832.1|/0/conserved hypothetical protein [Aspergillus terreus NIH2624]

4354892	1557	1291.31	33.14	-2.879338396	0.0028816	0.01052883	Down	ATEG_00135	hypothetical protein	-	GO:0005783//endoplasmic reticulum;GO:0016021//integral component of membrane	-	GO:0055085//transmembrane transport	gi|115491187|ref|XP_001210221.1|/0/conserved hypothetical protein [Aspergillus terreus NIH2624]
4321013	1200	24.13	0.46	-2.877642617	0.00291055	0.01062635	Down	ATEG_05024	hypothetical protein	ko04011//MAPK signaling pathway - yeast	-	-	-	gi|115397221|ref|XP_001214202.1|/0/predicted protein [Aspergillus terreus NIH2624]
4318626	1350	340.86	27.91	-2.871344777	7.98E-15	1.13E-13	Down	ATEG_03754	similar to cyclin	ko04111//Cell cycle - yeast	-	-	-	gi|115390855|ref|XP_001212932.1|/0/hypothetical protein ATEG_03754 [Aspergillus terreus NIH2624]
4322729	1782	122.16	8.72	-2.857170161	6.18E-06	3.79E-05	Down	ATEG_07586	hypothetical protein	-	GO:0005634//nucleus	GO:0000981//RNA polymerase II transcription factor activity, sequence-specific DNA binding;GO:0008270//zinc ion binding	GO:0006357//regulation of transcription from RNA polymerase II promoter	gi|115401238|ref|XP_001216207.1|/0/conserved hypothetical protein [Aspergillus terreus NIH2624]
4317845	1374	47.07	2.19	-2.84872225	0.00063814	0.00274035	Down	ATEG_02799	hypothetical protein	ko01100//Metabolic pathways;ko00500//Starch and sucrose metabolism	-	-	-	gi|115388944|ref|XP_001211977.1|/0/predicted protein [Aspergillus terreus NIH2624]
4321186	522	16.26	0	-2.848615381	0.00438192	0.01525625	Down	ATEG_04962	hypothetical protein	-	-	GO:0003796//lysozyme activity	GO:0009253//peptidoglycan catabolic process;GO:0016998//cell wall macromolecule catabolic process	gi|115397097|ref|XP_001214140.1|/8.33323e-127/predicted protein [Aspergillus terreus NIH2624]

4315815	9158	1714.4	150.28	-2.845967736	4.30E-35	1.74E-33	Down	ATEG_01674	hypothetical protein	ko01100//Metabolic pathways;ko00500//Starch and sucrose metabolism	GO:0016021//integral component of membrane	GO:0016740//transferase activity	-	gi|115384984|ref|XP_001209039.1|/0/conserved hypothetical protein [Aspergillus terreus NIH2624]
4354491	1215	290.81	24.31	-2.84107887	3.38E-14	4.64E-13	Down	ATEG_09844	hypothetical protein	ko00500//Starch and sucrose metabolism	GO:0005576//extracellular region	GO:0046557//glucan endo-1,6-beta-glucosidase activity	GO:0009251//glucan catabolic process;GO:0071555//cell wall organization	gi|115443318|ref|XP_001218466.1|/0/conserved hypothetical protein [Aspergillus terreus NIH2624]
4317247	2244	63.32	4.06	-2.839922598	5.36E-05	0.00028228	Down	ATEG_02671	hypothetical protein	-	-	GO:0016846//carbon-sulfur lyase activity	GO:0008152//metabolic process	gi|115388687|ref|XP_001211849.1|/0/conserved hypothetical protein [Aspergillus terreus NIH2624]
4318877	1431	1134.53	100	-2.837767403	1.06E-29	3.54E-28	Down	ATEG_06879	similar to ornithine monooxygenase	-	GO:0005634//nucleus;GO:0005829//cytosol	GO:0016491//oxidoreductase activity	GO:0055114//oxidation-reduction process	gi|115386048|ref|XP_001209565.1|/0/hypothetical protein ATEG_06879 [Aspergillus terreus NIH2624]
4319241	5557	48.76	2.75	-2.831209719	0.00035166	0.00160177	Down	ATEG_07378	hypothetical protein	-	-	GO:0016491//oxidoreductase activity;GO:0071949//FAD binding	GO:0055114//oxidation-reduction process	gi|115387046|ref|XP_001210064.1|/0/conserved hypothetical protein [Aspergillus terreus NIH2624]
4321082	831	28.55	0.92	-2.825503003	0.00241572	0.0090162	Down	ATEG_04970	hypothetical protein	-	-	-	-	gi|115397113|ref|XP_001214148.1|/0/conserved hypothetical protein [Aspergillus terreus NIH2624]

4322898	975	42.6	2.24	-2.825367585	0.00046818	0.00207214	Down	ATEG_07440	alternative oxidase, mitochondrial precursor	-	GO:0016021//integral component of membrane;GO:0070469//respiratory chain	GO:0009916//alternative oxidase activity;GO:0046872//metal ion binding	GO:0055114//oxidation-reduction process	gi|115400946|ref|XP_001216061.1|/0/alternative oxidase, mitochondrial precursor [Aspergillus terreus NIH2624]
4321077	723	224.13	18.64	-2.817919771	4.87E-10	4.81E-09	Down	ATEG_05147	hypothetical protein	-	GO:0016021//integral component of membrane	-	-	gi|115397467|ref|XP_001214325.1|/4.39539e-171/conserved hypothetical protein [Aspergillus terreus NIH2624]
4317952	387	16.24	0	-2.813396565	0.00494281	0.0169079	Down	ATEG_03536	similar to factor C protein precursor	-	-	-	-	gi|115390418|ref|XP_001212714.1|/2.65917e-90/hypothetical protein ATEG_03536 [Aspergillus terreus NIH2624]
4322976	3756	758.45	67.03	-2.813161666	5.59E-26	1.59E-24	Down	ATEG_07455	hypothetical protein	-	GO:0016021//integral component of membrane	-	-	gi|115400976|ref|XP_001216076.1|/0/predicted protein [Aspergillus terreus NIH2624]
4316582	1943	117.68	8.92	-2.801818844	5.94E-07	4.24E-06	Down	ATEG_02473	hypothetical protein	-	-	GO:0010181//FMN binding;GO:0016491//oxidoreductase activity	GO:0055114//oxidation-reduction process	gi|115388291|ref|XP_001211651.1|/1.18108e-166/conserved hypothetical protein [Aspergillus terreus NIH2624]
4354902	6699	8613.84	578.1	-2.796042967	0.00010858	0.00054019	Down	ATEG_00145	similar to polyketide synthase	-	-	GO:0016740//transferase activity;GO:0016788//hydrolase activity, acting on ester bonds;GO:0031177//phosphopantetheine binding	GO:0009058//biosynthetic process	gi|115491207|ref|XP_001210231.1|/0/hypothetical protein ATEG_00145 [Aspergillus terreus NIH2624]

4316766	1824	67.8	4.57	-2.794371024	8.30E-05	0.00042346	Down	ATEG_02252	hypothetical protein	ko01100//Metabolic pathways;ko00230//Purine metabolism	GO:0016021//integral component of membrane	GO:0022891//substrate-specific transmembrane transporter activity	GO:0055085//transmembrane transport	gi|115387849|ref|XP_001211430.1|/0/conserved hypothetical protein [Aspergillus terreus NIH2624]
4315672	996	15.7	0	-2.792348664	0.00528725	0.01791632	Down	ATEG_01085	hypothetical protein	-	-	-	-	gi|115383806|ref|XP_001208450.1|/0/predicted protein [Aspergillus terreus NIH2624]
4319658	1005	8117.04	753.98	-2.785490896	4.64E-47	2.90E-45	Down	ATEG_10199	glyceraldehyde-3-phosphate dehydrogenase	ko01100//Metabolic pathways;ko01110//Biosynthesis of secondary metabolites;ko01130//Biosynthesis of antibiotics;ko01230//Biosynthesis of amino acids;ko01200//Carbon metabolism;ko00010//Glycolysis / Gluconeogenesis	-	GO:0004365//glyceraldehyde-3-phosphate dehydrogenase (NAD+) (phosphorylating) activity;GO:0050661//NADP binding;GO:0051287//NAD binding	GO:0006006//glucose metabolic process;GO:0006096//glycolytic process;GO:0055114//oxidation-reduction process	gi|115385909|ref|XP_001209501.1|/0/glyceraldehyde-3-phosphate dehydrogenase [Aspergillus terreus NIH2624]

4315954	1545	366.57	31.18	-2.782682097	8.25E-13	1.05E-11	Down	ATEG_01478	hypothetical protein	-	GO:0005829//cytosol;GO:0016593//Cdc73/Paf1 complex;GO:0035327//transcriptionally active chromatin	GO:0000983//transcription factor activity, RNA polymerase II core promoter sequence-specific;GO:0001089//transcription factor activity, TFIIF-class transcription factor binding;GO:0003682//chromatin binding;GO:1990269//RNA polymerase II C-terminal domain phosphoserine binding	GO:0000083//regulation of transcription involved in G1/S transition of mitotic cell cycle;GO:0000122//negative regulation of transcription from RNA polymerase II promoter;GO:0000183//chromatin silencing at rDNA;GO:0001015//snoRNA transcription from an RNA polymerase II promoter;GO:0006353//DNA-templated transcription, termination;GO:0006364//rRNA processing;GO:0031124//mRNA 3'-end processing;GO:0031126//snoRNA 3'-end processing;GO:0031938//regulation of chromatin silencing at telomere;GO:0032968//positive	gi|115384592|ref|XP_001208843.1|/0/conserved hypothetical protein [Aspergillus terreus NIH2624]
4317321	583	848.15	77.17	-2.778533976	2.87E-19	5.60E-18	Down	ATEG_02628	hypothetical protein	-	-	-	-	gi|115388601|ref|XP_001211806.1|/2.33472e-95/conserved hypothetical protein [Aspergillus terreus NIH2624]

4319912	1161	89.68	6.76	-2.772459594	5.59E-06	3.46E-05	Down	ATEG_04490	hypothetical protein	ko01100//Metabolic pathways;ko01110//Biosynthesis of secondary metabolites;ko01130//Biosynthesis of antibiotics;ko00565//Ether lipid metabolism	-	GO:0003847//1-alkyl-2-acetylglycerophosphocholine esterase activity	GO:0016042//lipid catabolic process	gi|115396058|ref|XP_001213668.1|/0/predicted protein [Aspergillus terreus NIH2624]
4318984	1051	303.83	27.01	-2.755089992	1.60E-13	2.09E-12	Down	ATEG_07374	hypothetical protein	ko01100//Metabolic pathways;ko00260//Glycine, serine and threonine metabolism;ko00240//Pyrimidine metabolism	-	GO:0016491//oxidoreductase activity	GO:0055114//oxidation-reduction process	gi|115387038|ref|XP_001210060.1|/0/conserved hypothetical protein [Aspergillus terreus NIH2624]
4316580	846	317.79	28.25	-2.753129902	2.96E-14	4.08E-13	Down	ATEG_02034	hypothetical protein	-	GO:0016021//integral component of membrane	-	-	gi|115387413|ref|XP_001211212.1|/0/predicted protein [Aspergillus terreus NIH2624]
4316581	1416	67.27	4.43	-2.745919733	0.00013327	0.00065266	Down	ATEG_02035	hypothetical protein	-	GO:0016021//integral component of membrane	-	-	gi|115387415|ref|XP_001211213.1|/0/conserved hypothetical protein [Aspergillus terreus NIH2624]
4355524	1524	27.49	0.92	-2.733762675	0.00366891	0.0130251	Down	ATEG_00769	similar to UDP-galactopyranose mutase	ko01100//Metabolic pathways;ko00500//Starch and sucrose metabolism	-	GO:0016491//oxidoreductase activity	GO:0055114//oxidation-reduction process	gi|115492455|ref|XP_001210855.1|/0/hypothetical protein ATEG_00769 [Aspergillus terreus NIH2624]
4354897	1380	3749.16	320.08	-2.73363955	1.44E-07	1.11E-06	Down	ATEG_00140	hypothetical protein	-	-	-	-	gi|115491197|ref|XP_001210226.1|/0/predicted protein [Aspergillus terreus NIH2624]

4353620	654	59.43	3.69	-2.72711867	0.00065002	0.00278497	Down	ATEG_09340	hypothetical protein	ko01100//Metabolic pathways;ko00230//Purine metabolism;ko00240//Pyrimidine metabolism;ko03020//RNA polymerase	-	-	GO:0006359//regulation of transcription from RNA polymerase III promoter	gi|115438034|ref|XP_001217962.1|/2.21278e-164/conserved hypothetical protein [Aspergillus terreus NIH2624]
4320884	1308	15.12	0	-2.726159679	0.00655435	0.0216015	Down	ATEG_05477	hypothetical protein	-	GO:0016021//integral component of membrane	-	-	gi|115398127|ref|XP_001214655.1|/0/conserved hypothetical protein [Aspergillus terreus NIH2624]
4315547	789	4235.03	413.26	-2.713550813	1.23E-44	7.06E-43	Down	ATEG_01821	similar to ESDC	ko01100//Metabolic pathways;ko00520//Amino sugar and nucleotide sugar metabolism	-	-	GO:0043941;GO:0075308	gi|115385278|ref|XP_001209186.1|/0/hypothetical protein ATEG_01821 [Aspergillus terreus NIH2624]
4323497	1053	114.83	9.46	-2.711200065	1.52E-06	1.03E-05	Down	ATEG_08873	hypothetical protein	ko00254//Aflatoxin biosynthesis	-	GO:0016787//hydrolase activity	GO:0008152//metabolic process	gi|115402765|ref|XP_001217459.1|/8.32083e-153/predicted protein [Aspergillus terreus NIH2624]
4322284	553	49.93	2.77	-2.710064112	0.00142785	0.00562919	Down	ATEG_06661	hypothetical protein	-	-	-	-	gi|115400501|ref|XP_001215839.1|/2.46206e-103/predicted protein [Aspergillus terreus NIH2624]
4354901	3751	3789.06	266.75	-2.707847708	0.00025811	0.00120671	Down	ATEG_00144	hypothetical protein	ko00350//Tyrosine metabolism	-	-	-	gi|115491205|ref|XP_001210230.1|/0/predicted protein [Aspergillus terreus NIH2624]

4316802	864	20.16	0.46	-2.704461283	0.00539465	0.0182276	Down	ATEG_02391	hypothetical protein	-	-	-	-	gi|115388127|ref|XP_001211569.1|/0/predicted protein [Aspergillus terreus NIH2624]
4321189	1167	738.24	70.79	-2.704137939	1.50E-24	3.91E-23	Down	ATEG_04953	hypothetical protein	-	-	-	-	gi|115397079|ref|XP_001214131.1|/0/predicted protein [Aspergillus terreus NIH2624]
4319424	4065	1667.66	158.79	-2.69934659	3.47E-24	8.88E-23	Down	ATEG_10147	hypothetical protein	-	GO:0016021//integral component of membrane	GO:0003924//GTPase activity;GO:0005525//GTP binding;GO:0016491//oxidoreductase activity	GO:0055114//oxidation-reduction process	gi|115385805|ref|XP_001209449.1|/0/conserved hypothetical protein [Aspergillus terreus NIH2624]
4354085	1101	789.2	76.3	-2.693403952	2.25E-18	4.06E-17	Down	ATEG_09494	hypothetical protein	ko04011//MAPK signaling pathway - yeast	-	-	-	gi|115442618|ref|XP_001218116.1|/7.99335e-125/predicted protein [Aspergillus terreus NIH2624]
4315998	3050	5284.67	517.73	-2.692850602	2.04E-66	2.25E-64	Down	ATEG_01884	similar to heterokaryon incompatibility protein HET-C	ko01100//Metabolic pathways;ko01110//Biosynthesis of secondary metabolites;ko04144//Endocytosis;ko00564//Glycerophospholipid metabolism;ko00565//Ether lipid metabolism	-	-	-	gi|115385404|ref|XP_001209249.1|/0/hypothetical protein ATEG_01884 [Aspergillus terreus NIH2624]
4323342	1000	15.11	0	-2.681842712	0.00754922	0.02432499	Down	ATEG_08941	hypothetical protein	-	-	-	-	gi|115402901|ref|XP_001217527.1|/3.16492e-146/predicted protein [Aspergillus terreus NIH2624]

4315604	3429	1115.35	109.02	-2.672238198	4.77E-27	1.44E-25	Down	ATEG_01078	hypothetical protein	ko01100//Metabolic pathways;ko01110//Biosynthesis of secondary metabolites	GO:0016021//integral component of membrane	GO:0004497//monooxygenase activity;GO:0005506//iron ion binding;GO:0008168//methyltransferase activity;GO:0009055//electron carrier activity;GO:0016705//oxidoreductase activity, acting on paired donors, with incorporation or reduction of molecular oxygen;GO:0016787//hydrolase activity;GO:0020037//heme binding	GO:0032259//methylation;GO:0055114//oxidation-reduction process	gi|115383792|ref|XP_001208443.1|/0/conserved hypothetical protein [Aspergillus terreus NIH2624]
4354006	3177	91.39	7.31	-2.668619499	4.76E-05	0.00025372	Down	ATEG_09278	hypothetical protein	-	-	-	-	gi|115437790|ref|XP_001217900.1|/6.52971e-67/predicted protein [Aspergillus terreus NIH2624]
4322453	633	5914.7	596.56	-2.66309408	2.11E-87	3.66E-85	Down	ATEG_06372	hypothetical protein	-	-	-	-	gi|115399922|ref|XP_001215550.1|/2.69923e-166/predicted protein [Aspergillus terreus NIH2624]

4355310	1371	33.62	1.82	-2.660591062	0.00201749	0.00769808	Down	ATEG_00556	hypothetical protein	ko01100//Metabolic pathways;ko00561//Glycerolipid metabolism	GO:0016020//membrane	GO:0004497//monooxygenase activity;GO:0005506//iron ion binding;GO:0016705//oxidoreductase activity, acting on paired donors, with incorporation or reduction of molecular oxygen;GO:0016787//hydrolase activity;GO:0020037//heme binding	GO:0055114//oxidation-reduction process	gi|115492029|ref|XP_001210642.1|/0/conserved hypothetical protein [Aspergillus terreus NIH2624]
4322688	819	36.44	1.75	-2.658865198	0.00250432	0.00930986	Down	ATEG_07533	general stress protein 39	ko01100//Metabolic pathways;ko01212//Fatty acid metabolism;ko01040//Biosynthesis of unsaturated fatty acids;ko00061//Fatty acid biosynthesis;ko00780//Biotin metabolism	-	GO:0016491//oxidoreductase activity	GO:0055114//oxidation-reduction process	gi|115401132|ref|XP_001216154.1|/0/general stress protein 39 [Aspergillus terreus NIH2624]
4353718	1527	186.04	17.58	-2.631167767	2.69E-09	2.51E-08	Down	ATEG_09245	hypothetical protein	ko04144//Endocytosis	-	-	-	gi|115437662|ref|XP_001217867.1|/0/predicted protein [Aspergillus terreus NIH2624]

4322230	1083	388.48	38.24	-2.630819341	5.60E-15	8.01E-14	Down	ATEG_06292	hypothetical protein	ko01100//Metabolic pathways;ko01110//Biosynthesis of secondary metabolites;ko01130//Biosynthesis of antibiotics;ko01230//Biosynthesis of amino acids;ko00260//Glycine, serine and threonine metabolism	-	GO:0016829//lyase activity;GO:0030170//pyridoxal phosphate binding	GO:0006520//cellular amino acid metabolic process	gi|115399762|ref|XP_001215470.1|/0/conserved hypothetical protein [Aspergillus terreus NIH2624]
4320315	4386	14.01	0	-2.625334525	0.00895962	0.02820132	Down	ATEG_04322	hypothetical protein	-	-	GO:0003824//catalytic activity	GO:0008152//metabolic process	gi|115395350|ref|XP_001213500.1|/0/predicted protein [Aspergillus terreus NIH2624]
4315521	2345	625.53	63.37	-2.625206939	2.69E-19	5.25E-18	Down	ATEG_01032	similar to glucose transporter	ko04113//Meiosis - yeast	GO:0016021//integral component of membrane	GO:0022891//substrate-specific transmembrane transporter activity	GO:0055085//transmembrane transport	gi|115383700|ref|XP_001208397.1|/0/hypothetical protein ATEG_01032 [Aspergillus terreus NIH2624]
4319123	1680	254.96	24.89	-2.624380918	4.00E-11	4.40E-10	Down	ATEG_07319	hypothetical protein	-	GO:0016021//integral component of membrane	GO:0046873//metal ion transmembrane transporter activity	GO:0030001//metal ion transport;GO:0055085//transmembrane transport	gi|115386928|ref|XP_001210005.1|/0/conserved hypothetical protein [Aspergillus terreus NIH2624]
4322370	1809	14.02	0	-2.616062531	0.00921703	0.02878941	Down	ATEG_06246	glucose oxidase precursor	ko01100//Metabolic pathways;ko00260//Glycine, serine and threonine metabolism	-	GO:0016614//oxidoreductase activity, acting on CH-OH group of donors;GO:0050660//flavin adenine dinucleotide binding	GO:0055114//oxidation-reduction process	gi|115399596|ref|XP_001215424.1|/0/glucose oxidase precursor [Aspergillus terreus NIH2624]

4315947	1155	1285.12	133.12	-2.614858322	7.87E-36	3.36E-34	Down	ATEG_01203	hypothetical protein	ko03013//RNA transport;ko03015//mRNA surveillance pathway	GO:0016021//integral component of membrane	-	-	gi|115384042|ref|XP_001208568.1|/0/predicted protein [Aspergillus terreus NIH2624]
4322212	2642	2300.92	236.65	-2.613277479	5.44E-43	2.95E-41	Down	ATEG_06406	hypothetical protein	-	-	GO:0004185//serine-type carboxypeptidase activity	GO:0006508//proteolysis	gi|115399991|ref|XP_001215584.1|/0/conserved hypothetical protein [Aspergillus terreus NIH2624]
4354263	1008	281.83	27.26	-2.611199713	2.98E-11	3.34E-10	Down	ATEG_09795	hypothetical protein	-	-	GO:0016491//oxidoreductase activity	GO:0055114//oxidation-reduction process	gi|115443220|ref|XP_001218417.1|/0/predicted protein [Aspergillus terreus NIH2624]
4322498	1185	14	0	-2.607973906	0.00944692	0.02943888	Down	ATEG_07567	hypothetical protein	ko03013//RNA transport;ko03015//mRNA surveillance pathway	GO:0016021//integral component of membrane	-	-	gi|115401200|ref|XP_001216188.1|/0/predicted protein [Aspergillus terreus NIH2624]
4316084	1065	14.02	0	-2.593627305	0.00986472	0.03053798	Down	ATEG_01005	hypothetical protein	ko01100//Metabolic pathways;ko00350//Tyrosine metabolism	-	GO:0005524//ATP binding;GO:0046872//metal ion binding	-	gi|115383646|ref|XP_001208370.1|/0/predicted protein [Aspergillus terreus NIH2624]
4316682	1284	40.92	2.67	-2.587281321	0.00131057	0.00521506	Down	ATEG_02555	hypothetical protein	-	-	-	-	gi|115388455|ref|XP_001211733.1|/0/predicted protein [Aspergillus terreus NIH2624]
4317394	363	71.72	5.93	-2.58623212	0.00015218	0.00073987	Down	ATEG_03177	hypothetical protein	ko04144//Endocytosis	-	-	-	gi|115389700|ref|XP_001212355.1|/3.60073e-82/predicted protein [Aspergillus terreus NIH2624]

4317222	951	134.99	12.57	-2.577416688	1.01E-06	6.91E-06	Down	ATEG_02692	hypothetical protein	-	-	-	-	gi|115388729|ref|XP_001211870.1|/0/predicted protein [Aspergillus terreus NIH2624]
4354120	1698	31.96	1.82	-2.573032309	0.00318933	0.01149654	Down	ATEG_09759	hypothetical protein	-	-	GO:0004672//protein kinase activity;GO:0005524//ATP binding	GO:0006468//protein phosphorylation	gi|115443148|ref|XP_001218381.1|/0/predicted protein [Aspergillus terreus NIH2624]
4317039	1134	12019.86	1295.48	-2.570360296	1.60E-101	3.86E-99	Down	ATEG_02172	sterol 24-C-methyltransferase	ko01100//Metabolic pathways;ko01110//Biosynthesis of secondary metabolites;ko01130//Biosynthesis of antibiotics;ko00100//Steroid biosynthesis	-	GO:0003838//sterol 24-C-methyltransferase activity	GO:0016126//sterol biosynthetic process;GO:0032259//methylation	gi|115387689|ref|XP_001211350.1|/0/sterol 24-C-methyltransferase [Aspergillus terreus NIH2624]
4317675	1914	1747.79	187.25	-2.568419472	3.31E-46	1.98E-44	Down	ATEG_03482	hypothetical protein	ko01100//Metabolic pathways;ko00380//Tryptophan metabolism	-	GO:0020037//heme binding;GO:0051213//dioxygenase activity	GO:0055114//oxidation-reduction process	gi|115390310|ref|XP_001212660.1|/0/conserved hypothetical protein [Aspergillus terreus NIH2624]
4353938	1416	58.26	4.45	-2.567036365	0.00040192	0.00180252	Down	ATEG_09037	hypothetical protein	-	GO:0016021//integral component of membrane	GO:0005216//ion channel activity	GO:0034220//ion transmembrane transport	gi|115436848|ref|XP_001217659.1|/0/conserved hypothetical protein [Aspergillus terreus NIH2624]
4353880	1767	678.27	71.33	-2.557695014	9.76E-21	2.09E-19	Down	ATEG_09162	hypothetical protein	-	GO:0016021//integral component of membrane	-	-	gi|115437334|ref|XP_001217784.1|/0/predicted protein [Aspergillus terreus NIH2624]

4315980	903	100.33	8.39	-2.555665011	7.50E-05	0.00038572	Down	ATEG_01677	hypothetical protein	-	GO:0016021//integral component of membrane	-	-	gi|115384990|ref|XP_001209042.1|/0/conserved hypothetical protein [Aspergillus terreus NIH2624]
4354738	2310	22.4	0.9	-2.554679976	0.00659709	0.02168901	Down	ATEG_10359	hypothetical protein	ko01100//Metabolic pathways;ko00790//Folate biosynthesis	GO:0016021//integral component of membrane	-	-	gi|115449831|ref|XP_001218707.1|/0/predicted protein [Aspergillus terreus NIH2624]
4322642	498	13.44	0	-2.55098927	0.01118308	0.03415716	Down	ATEG_07702	hypothetical protein	-	-	-	-	gi|115401470|ref|XP_001216323.1|/5.94135e-123/predicted protein [Aspergillus terreus NIH2624]
4322391	615	168.72	16.61	-2.550184122	3.89E-08	3.22E-07	Down	ATEG_06528	hypothetical protein	ko00480//Glutathione metabolism	-	-	-	gi|115400235|ref|XP_001215706.1|/2.91493e-149/conserved hypothetical protein [Aspergillus terreus NIH2624]
4320632	1344	31.37	1.82	-2.54967477	0.00355033	0.01264243	Down	ATEG_04967	hypothetical protein	ko01100//Metabolic pathways;ko01110//Biosynthesis of secondary metabolites	GO:0016020//membrane	GO:0004497//monooxygenase activity;GO:0005506//iron ion binding;GO:0016705//oxidoreductase activity, acting on paired donors, with incorporation or reduction of molecular oxygen;GO:0020037//heme binding	GO:0055114//oxidation-reduction process	gi|115397107|ref|XP_001214145.1|/0/predicted protein [Aspergillus terreus NIH2624]

4320560	345	334.49	34.93	-2.549609536	1.18E-11	1.36E-10	Down	ATEG_04939	hypothetical protein	-	-	-	-	gi|115397051|ref|XP_001214117.1|/2.59478e-79/predicted protein [Aspergillus terreus NIH2624]
4320827	4731	102.02	9.41	-2.541345885	9.09E-06	5.43E-05	Down	ATEG_05017	hypothetical protein	-	-	GO:0003824//catalytic activity	GO:0009116//nucleoside metabolic process	gi|115397207|ref|XP_001214195.1|/0/conserved hypothetical protein [Aspergillus terreus NIH2624]
4355646	1113	47.65	3.6	-2.539839719	0.00080621	0.00336206	Down	ATEG_00885	hypothetical protein	-	GO:0016020//membrane	GO:0008271//secondary active sulfate transmembrane transporter activity	GO:1902358//sulfate transmembrane transport	gi|115492687|ref|XP_001210971.1|/0/predicted protein [Aspergillus terreus NIH2624]
4322870	975	31.4	1.82	-2.539127442	0.00380675	0.01345844	Down	ATEG_07547	similar to secreted protein	-	-	GO:0004065//arylsulfatase activity;GO:0018741//alkyl sulfatase activity;GO:0046983//protein dimerization activity	GO:0018909//dodecyl sulfate metabolic process	gi|115401160|ref|XP_001216168.1|/0/hypothetical protein ATEG_07547 [Aspergillus terreus NIH2624]
4323000	4473	636.12	65.92	-2.53641027	2.41E-14	3.35E-13	Down	ATEG_07864	ABC transporter CDR4	ko02010//ABC transporters	GO:0005886//plasma membrane;GO:0016021//integral component of membrane	GO:0005524//ATP binding;GO:0042626//ATPase activity, coupled to transmembrane movement of substances	GO:0046618//drug export;GO:0055085//transmembrane transport	gi|115401794|ref|XP_001216485.1|/0/ABC transporter CDR4 [Aspergillus terreus NIH2624]
4318373	819	35522.12	3908.59	-2.536187949	2.81E-46	1.70E-44	Down	ATEG_04096	hypothetical protein	-	GO:0016021//integral component of membrane	-	-	gi|115391539|ref|XP_001213274.1|/0/conserved hypothetical protein [Aspergillus terreus NIH2624]

4353123	2950	148.03	14.44	-2.535270427	8.10E-07	5.65E-06	Down	ATEG_08302	hypothetical protein	-	GO:0016021//integral component of membrane	-	-	gi|115433572|ref|XP_001216923.1|/3.94196e-152/predicted protein [Aspergillus terreus NIH2624]
4317455	1495	163.01	16.24	-2.534405061	1.38E-07	1.07E-06	Down	ATEG_03220	similar to aldo-keto reductase	ko01100//Metabolic pathways;ko00051//Fructose and mannose metabolism;ko00650//Butanoate metabolism;ko00591//Linoleic acid metabolism	-	GO:0016491//oxidoreductase activity	GO:0055114//oxidation-reduction process	gi|115389786|ref|XP_001212398.1|/0/hypothetical protein ATEG_03220 [Aspergillus terreus NIH2624]
4316579	3193	81.27	7.13	-2.523383297	7.48E-05	0.00038506	Down	ATEG_02033	hypothetical protein	-	-	-	-	gi|115387411|ref|XP_001211211.1|/0/predicted protein [Aspergillus terreus NIH2624]
4317231	3284	282.92	29.66	-2.522563517	1.78E-11	2.04E-10	Down	ATEG_02689	hypothetical protein	-	GO:0016021//integral component of membrane	-	-	gi|115388723|ref|XP_001211867.1|/0/predicted protein [Aspergillus terreus NIH2624]
4323438	891	31.4	1.78	-2.516805175	0.00421594	0.01475619	Down	ATEG_08633	hypothetical protein	ko01110//Biosynthesis of secondary metabolites;ko00564//Glycerophospholipid metabolism;ko00561//Glycerolipid metabolism	-	GO:0016491//oxidoreductase activity	GO:0055114//oxidation-reduction process	gi|115402285|ref|XP_001217219.1|/0/conserved hypothetical protein [Aspergillus terreus NIH2624]
4316893	2499	1118.82	123.72	-2.515886794	1.40E-21	3.14E-20	Down	ATEG_02219	similar to phosphoenolpyruvate synthase	ko01100//Metabolic pathways;ko01200//Carbon metabolism;ko00620//Pyruvate metabolism;ko00680//Methane metabolism	-	GO:0005524//ATP binding;GO:0008986//pyruvate, water dikinase activity	GO:0006090//pyruvate metabolic process;GO:0016310//phosphorylation	gi|115387783|ref|XP_001211397.1|/0/hypothetical protein ATEG_02219 [Aspergillus terreus NIH2624]

4323429	645	144.59	14.53	-2.513914859	8.37E-07	5.83E-06	Down	ATEG_08579	hypothetical protein	-	-	-	-	gi|115402177|ref|XP_001217165.1|/2.38912e-162/predicted protein [Aspergillus terreus NIH2624]
4353655	852	50.44	4.06	-2.50663613	0.00068696	0.00291269	Down	ATEG_09261	similar to embryo-specific protein 1	-	GO:0016021//integral component of membrane	GO:0005509//calcium ion binding	-	gi|115437724|ref|XP_001217883.1|/0/hypothetical protein ATEG_09261 [Aspergillus terreus NIH2624]
4315805	1464	29.74	1.31	-2.503085669	0.00725841	0.02354967	Down	ATEG_01697	hypothetical protein	-	GO:0016021//integral component of membrane	GO:0015171//amino acid transmembrane transporter activity	GO:0003333//amino acid transmembrane transport	gi|115385030|ref|XP_001209062.1|/0/predicted protein [Aspergillus terreus NIH2624]
4320087	1263	884.5	98.17	-2.50274564	5.86E-26	1.66E-24	Down	ATEG_04377	hypothetical protein	ko01100//Metabolic pathways;ko00500//Starch and sucrose metabolism	-	GO:0003824//catalytic activity;GO:0050662//coenzyme binding	-	gi|115395832|ref|XP_001213555.1|/0/conserved hypothetical protein [Aspergillus terreus NIH2624]
4317198	2190	179.39	18.59	-2.497879389	1.20E-07	9.36E-07	Down	ATEG_02718	hypothetical protein	-	GO:0005634//nucleus	GO:0000981//RNA polymerase II transcription factor activity, sequence-specific DNA binding;GO:0003677//DNA binding;GO:0008270//zinc ion binding	GO:0006357//regulation of transcription from RNA polymerase II promoter	gi|115388781|ref|XP_001211896.1|/0/predicted protein [Aspergillus terreus NIH2624]
4323062	363	23	0.92	-2.497701017	0.00892603	0.02811043	Down	ATEG_08598	hypothetical protein	-	-	-	-	gi|115402215|ref|XP_001217184.1|/6.96811e-89/predicted protein [Aspergillus terreus NIH2624]

4319354	1047	1942.76	218.96	-2.497451863	5.86E-25	1.57E-23	Down	ATEG_07062	similar to alcohol dehydrogenase	ko00640//Propanoate metabolism	-	GO:0008270//zinc ion binding;GO:0016491//oxidoreductase activity	GO:0055114//oxidation-reduction process	gi|115386414|ref|XP_001209748.1|/0/hypothetical protein ATEG_07062 [Aspergillus terreus NIH2624]
4316453	1323	114.83	11.26	-2.496164342	5.41E-06	3.36E-05	Down	ATEG_01915	hypothetical protein	-	-	-	-	gi|115387175|ref|XP_001211093.1|/0/predicted protein [Aspergillus terreus NIH2624]
4353869	1512	1946.71	220.8	-2.489407371	1.40E-35	5.92E-34	Down	ATEG_09163	hypothetical protein	-	GO:0016021//integral component of membrane	GO:0016757//transferase activity, transferring glycosyl groups	-	gi|115437338|ref|XP_001217785.1|/0/predicted protein [Aspergillus terreus NIH2624]
4317810	432	468.87	50.46	-2.483198294	1.33E-12	1.66E-11	Down	ATEG_03356	hypothetical protein	-	-	-	-	gi|115390058|ref|XP_001212534.1|/4.21961e-101/predicted protein [Aspergillus terreus NIH2624]
4317761	2814	533.74	59.44	-2.471531972	1.40E-15	2.09E-14	Down	ATEG_02815	hypothetical protein	ko01100//Metabolic pathways;ko04146//Peroxisome;ko00071//Fatty acid degradation;ko01212//Fatty acid metabolism;ko00061//Fatty acid biosynthesis	-	GO:0016788//hydrolase activity, acting on ester bonds;GO:0031177//phosphopantetheine binding	GO:0009058//biosynthetic process	gi|115388976|ref|XP_001211993.1|/0/predicted protein [Aspergillus terreus NIH2624]
4320242	4509	6371.31	735.86	-2.469707372	2.37E-62	2.22E-60	Down	ATEG_04403	hypothetical protein	-	-	-	-	gi|115395884|ref|XP_001213581.1|/0/conserved hypothetical protein [Aspergillus terreus NIH2624]

4322424	1785	267.84	29.22	-2.469078154	3.29E-11	3.66E-10	Down	ATEG_06304	hypothetical protein	ko04011//MAPK signaling pathway - yeast	GO:0016021//integral component of membrane	-	GO:0055085//transmembrane transport	gi|115399786|ref|XP_001215482.1|/8.9053e-177/predicted protein [Aspergillus terreus NIH2624]
4320366	1482	163.05	17.02	-2.464026014	1.39E-07	1.08E-06	Down	ATEG_04903	similar to Aft3-2	-	-	GO:0003859//3-hydroxybutyryl-CoA dehydratase activity;GO:0016853//isomerase activity	GO:0008152//metabolic process	gi|115396884|ref|XP_001214081.1|/0/hypothetical protein ATEG_04903 [Aspergillus terreus NIH2624]
4323474	2550	33.62	2.28	-2.460742875	0.00396343	0.01396501	Down	ATEG_08837	hypothetical protein	-	-	-	-	gi|115402693|ref|XP_001217423.1|/0/predicted protein [Aspergillus terreus NIH2624]
4316806	501	940.6	107.77	-2.460058699	9.96E-25	2.64E-23	Down	ATEG_02395	hypothetical protein	-	-	GO:0016798//hydrolase activity, acting on glycosyl bonds	-	gi|115388135|ref|XP_001211573.1|/1.45177e-119/predicted protein [Aspergillus terreus NIH2624]
4354676	708	99.2	9.89	-2.453982513	1.43E-05	8.33E-05	Down	ATEG_10351	hypothetical protein	-	-	GO:0010181//FMN binding;GO:0016491//oxidoreductase activity	GO:0055114//oxidation-reduction process	gi|115449801|ref|XP_001218699.1|/8.94634e-173/conserved hypothetical protein [Aspergillus terreus NIH2624]
4317074	945	123.92	12.55	-2.444648208	7.00E-06	4.24E-05	Down	ATEG_02153	hypothetical protein	ko01100//Metabolic pathways;ko01110//Biosynthesis of secondary metabolites;ko00100//Steroid biosynthesis	-	-	-	gi|115387651|ref|XP_001211331.1|/0/conserved hypothetical protein [Aspergillus terreus NIH2624]

4321321	1273	834.9	96.9	-2.442884703	2.77E-25	7.62E-24	Down	ATEG_06094	hypothetical protein	ko03010//Ribosome	GO:0016021//integral component of membrane	-	-	gi|115399366|ref|XP_001215272.1|/0/conserved hypothetical protein [Aspergillus terreus NIH2624]
4316794	717	68.94	6.23	-2.431812192	0.00033215	0.00152312	Down	ATEG_02399	hypothetical protein	ko04144//Endocytosis	-	-	-	gi|115388143|ref|XP_001211577.1|/2.6715e-180/predicted protein [Aspergillus terreus NIH2624]
4320458	1833	1402	164.89	-2.430833129	2.51E-26	7.32E-25	Down	ATEG_04360	hypothetical protein	-	GO:0016021//integral component of membrane	-	GO:0055085//transmembrane transport	gi|115395798|ref|XP_001213538.1|/0/conserved hypothetical protein [Aspergillus terreus NIH2624]
4353172	639	197.87	21.49	-2.429566551	2.36E-08	1.98E-07	Down	ATEG_08148	hypothetical protein	ko01100//Metabolic pathways;ko01130//Biosynthesis of antibiotics;ko00100//Steroid biosynthesis	GO:0016021//integral component of membrane	GO:0005506//iron ion binding;GO:0016491//oxidoreductase activity	GO:0006633//fatty acid biosynthetic process;GO:0055114//oxidation-reduction process	gi|115433264|ref|XP_001216769.1|/7.97916e-151/predicted protein [Aspergillus terreus NIH2624]
4321773	1575	690.11	79.5	-2.429337114	1.65E-17	2.81E-16	Down	ATEG_05844	hypothetical protein	ko01100//Metabolic pathways;ko01110//Biosynthesis of secondary metabolites	GO:0016021//integral component of membrane	GO:0004497//monooxygenase activity;GO:0005506//iron ion binding;GO:0009055//electron carrier activity;GO:0016705//oxidoreductase activity, acting on paired donors, with incorporation or reduction of molecular oxygen;GO:0020037//heme binding	GO:0008202//steroid metabolic process;GO:0055114//oxidation-reduction process	gi|115398866|ref|XP_001215022.1|/0/conserved hypothetical protein [Aspergillus terreus NIH2624]

4354672	1500	24.67	1.34	-2.423111283	0.00806462	0.02578204	Down	ATEG_10347	similar to cytoplasm protein	ko01100//Metabolic pathways;ko01110//Biosynthesis of secondary metabolites;ko00130//Ubiquinone and other terpenoid-quinone biosynthesis	GO:0005737//cytoplasm	GO:0010181//FMN binding;GO:0016491//oxidoreductase activity;GO:0016831//carboxy-lyase activity;GO:0046872//metal ion binding	GO:0033494//ferulate metabolic process;GO:0046281//cinnamic acid catabolic process;GO:0055114//oxidation-reduction process	gi|115449785|ref|XP_001218695.1|/0/hypothetical protein ATEG_10347 [Aspergillus terreus NIH2624]
4320540	870	1225.71	144.8	-2.420542227	5.38E-33	2.01E-31	Down	ATEG_04607	hypothetical protein	ko01100//Metabolic pathways;ko00500//Starch and sucrose metabolism	GO:0016021//integral component of membrane	-	GO:0006950//response to stress	gi|115396292|ref|XP_001213785.1|/0/conserved hypothetical protein [Aspergillus terreus NIH2624]
4354823	1758	12.32	0	-2.420533156	0.01617675	0.04713405	Down	ATEG_00067	hypothetical protein	-	GO:0016021//integral component of membrane	GO:0004222//metalloendopeptidase activity	GO:0006508//proteolysis	gi|115491051|ref|XP_001210153.1|/0/predicted protein [Aspergillus terreus NIH2624]
4320979	630	16.8	0.46	-2.408326993	0.01481776	0.04361199	Down	ATEG_05568	hypothetical protein	-	-	GO:0016491//oxidoreductase activity	GO:0055114//oxidation-reduction process	gi|115398309|ref|XP_001214746.1|/3.01491e-149/predicted protein [Aspergillus terreus NIH2624]
4354764	558	41.99	3.11	-2.408080497	0.00342687	0.01225394	Down	ATEG_00183	hypothetical protein	-	-	GO:0003796//lysozyme activity	GO:0009253//peptidoglycan catabolic process;GO:0016998//cell wall macromolecule catabolic process	gi|115491283|ref|XP_001210269.1|/2.08723e-135/conserved hypothetical protein [Aspergillus terreus NIH2624]
4317529	705	12.34	0	-2.405903136	0.01683657	0.04874346	Down	ATEG_03451	hypothetical protein	ko01100//Metabolic pathways;ko00564//Glycerophospholipid metabolism;ko00565//Ether lipid metabolism	GO:0016021//integral component of membrane	-	-	gi|115390248|ref|XP_001212629.1|/1.35786e-162/conserved hypothetical protein [Aspergillus terreus NIH2624]

4318129	786	577.63	68.86	-2.393559038	6.62E-15	9.42E-14	Down	ATEG_03052	hypothetical protein	-	GO:0016021//integral component of membrane	-	-	gi|115389450|ref|XP_001212230.1|/0/predicted protein [Aspergillus terreus NIH2624]
4355571	1206	373.63	28.87	-2.393496818	0.00451904	0.01565809	Down	ATEG_00810	GPI-anchor transamidase precursor	ko04111//Cell cycle - yeast;ko04113//Meiosis - yeast	GO:0042765//GPI-anchor transamidase complex	GO:0003923//GPI-anchor transamidase activity;GO:0008233//peptidase activity	GO:0006508//proteolysis;GO:0016255//attachment of GPI anchor to protein	gi|115492537|ref|XP_001210896.1|/0/GPI-anchor transamidase precursor [Aspergillus terreus NIH2624]
4317221	4128	169.74	18.91	-2.39275691	2.09E-07	1.59E-06	Down	ATEG_02691	hypothetical protein	-	-	-	-	gi|115388727|ref|XP_001211869.1|/0/predicted protein [Aspergillus terreus NIH2624]
4320527	2298	39.23	3.14	-2.391455018	0.00268132	0.0098856	Down	ATEG_04898	hypothetical protein	-	GO:0016021//integral component of membrane	-	-	gi|115396874|ref|XP_001214076.1|/0/predicted protein [Aspergillus terreus NIH2624]
4318716	798	98.65	10.4	-2.387475256	2.96E-05	0.00016448	Down	ATEG_03801	hypothetical protein	-	-	-	-	gi|115390949|ref|XP_001212979.1|/0/conserved hypothetical protein [Aspergillus terreus NIH2624]
4320665	1446	43.74	3.57	-2.385868428	0.00235366	0.00881262	Down	ATEG_05127	hypothetical protein	-	-	-	-	gi|115397427|ref|XP_001214305.1|/0/predicted protein [Aspergillus terreus NIH2624]
4322829	1232	70.65	6.83	-2.384878469	0.0005046	0.00221766	Down	ATEG_07478	quercetin 2,3-dioxygenase	-	-	GO:0051213//dioxygenase activity	GO:0055114//oxidation-reduction process	gi|115401022|ref|XP_001216099.1|/0/quercetin 2,3-dioxygenase [Aspergillus terreus NIH2624]

4322159	1905	221.81	25.14	-2.383147912	2.63E-08	2.19E-07	Down	ATEG_06310	hypothetical protein	-	-	GO:0003995//acyl-CoA dehydrogenase activity;GO:0050660//flavin adenine dinucleotide binding	GO:0055114//oxidation-reduction process	gi|115399798|ref|XP_001215488.1|/0/conserved hypothetical protein [Aspergillus terreus NIH2624]
4355440	2127	16.25	0.44	-2.380989497	0.01589175	0.04646691	Down	ATEG_00685	hypothetical protein	-	-	-	-	gi|115492287|ref|XP_001210771.1|/0/predicted protein [Aspergillus terreus NIH2624]
4316157	2085	519.63	62.67	-2.369877134	3.71E-16	5.82E-15	Down	ATEG_01801	protein TOXD	-	-	GO:0008270//zinc ion binding;GO:0016491//oxidoreductase activity	GO:0055114//oxidation-reduction process	gi|115385238|ref|XP_001209166.1|/0/protein TOXD [Aspergillus terreus NIH2624]
4320166	2821	4514.67	562.08	-2.365852793	5.91E-51	4.08E-49	Down	ATEG_04740	pH-response transcription factor pacC/RIM101	ko04011//MAPK signaling pathway - yeast	GO:0005634//nucleus;GO:0005737//cytoplasm	GO:0001078//transcriptional repressor activity, RNA polymerase II core promoter proximal region sequence-specific binding;GO:0003677//DNA binding;GO:0046872//metal ion binding	GO:0000122//negative regulation of transcription from RNA polymerase II promoter;GO:0000917//barrier septum assembly;GO:0007126;GO:0009272//fungal-type cell wall biogenesis;GO:0030437//ascospore formation;GO:0045944//positive regulation of transcription from RNA polymerase II promoter;GO:0071454//cellular response to anoxia;GO:0071469//cellular response to alkaline pH	gi|115396558|ref|XP_001213918.1|/0/pH-response transcription factor pacC/RIM101 [Aspergillus terreus NIH2624]

4321539	2067	38.67	3.18	-2.364583713	0.00338568	0.01212513	Down	ATEG_06179	hypothetical protein	ko01100//Metabolic pathways;ko04141//Protein processing in endoplasmic reticulum;ko00510//N-Glycan biosynthesis;ko00513//Various types of N-glycan biosynthesis	-	-	-	gi|115399536|ref|XP_001215357.1|/0/predicted protein [Aspergillus terreus NIH2624]
4322969	2496	37.55	2.77	-2.359064435	0.00659488	0.02168901	Down	ATEG_07495	hypothetical protein	-	-	-	-	gi|115401056|ref|XP_001216116.1|/0/predicted protein [Aspergillus terreus NIH2624]
4323191	654	71.71	7.15	-2.351890331	0.0003045	0.00140469	Down	ATEG_08823	hypothetical protein	ko01100//Metabolic pathways;ko00350//Tyrosine metabolism	-	GO:0008080//N-acetyltransferase activity	-	gi|115402665|ref|XP_001217409.1|/2.47693e-161/conserved hypothetical protein [Aspergillus terreus NIH2624]
4353908	423	59.37	5.08	-2.349974154	0.00381259	0.01346897	Down	ATEG_09177	hypothetical protein	-	GO:0016021//integral component of membrane;GO:0042721//mitochondrial inner membrane protein insertion complex	GO:0015266//protein channel activity;GO:0030943//mitochondrion targeting sequence binding	GO:0045039//protein import into mitochondrial inner membrane	gi|115437396|ref|XP_001217799.1|/3.23747e-99/conserved hypothetical protein [Aspergillus terreus NIH2624]
4353362	1449	7489.43	938.51	-2.346722196	5.67E-63	5.60E-61	Down	ATEG_08035	hypothetical protein	ko04144//Endocytosis	-	-	-	gi|115433038|ref|XP_001216656.1|/0/conserved hypothetical protein [Aspergillus terreus NIH2624]
4315979	978	8134.77	1026.5	-2.346633539	7.74E-69	9.43E-67	Down	ATEG_01676	hypothetical protein	ko01100//Metabolic pathways;ko00520//Amino sugar and nucleotide sugar metabolism	GO:0016021//integral component of membrane	GO:0016787//hydrolase activity	-	gi|115384988|ref|XP_001209041.1|/0/conserved hypothetical protein [Aspergillus terreus NIH2624]

4315757	1080	363.65	44.09	-2.346161219	5.79E-10	5.68E-09	Down	ATEG_01122	hypothetical protein	-	GO:0016021//integral component of membrane	-	-	gi|115383880|ref|XP_001208487.1|/0/predicted protein [Aspergillus terreus NIH2624]
4316375	789	28.59	1.85	-2.335247385	0.01044007	0.0321494	Down	ATEG_01744	hypothetical protein	ko04011//MAPK signaling pathway - yeast	GO:0016021//integral component of membrane	-	-	gi|115385124|ref|XP_001209109.1|/0/predicted protein [Aspergillus terreus NIH2624]
4317882	1191	41.46	3.57	-2.328768991	0.00294174	0.01072352	Down	ATEG_03188	hypothetical protein	-	-	-	-	gi|115389722|ref|XP_001212366.1|/0/predicted protein [Aspergillus terreus NIH2624]
4322353	825	60.56	5.93	-2.326352776	0.00121465	0.0048747	Down	ATEG_06644	similar to ferulic acid esterase A	-	-	-	-	gi|115400467|ref|XP_001215822.1|/0/hypothetical protein ATEG_06644 [Aspergillus terreus NIH2624]
4319373	1448	96.46	10.45	-2.318048266	0.00017134	0.00082701	Down	ATEG_06887	hypothetical protein	ko01100//Metabolic pathways;ko00510//N-Glycan biosynthesis;ko00513//Various types of N-glycan biosynthesis	-	-	-	gi|115386064|ref|XP_001209573.1|/6.15063e-110/predicted protein [Aspergillus terreus NIH2624]
4321113	1722	491.31	61.03	-2.313405952	2.89E-14	3.99E-13	Down	ATEG_05296	amino-acid permease inda1	-	GO:0016021//integral component of membrane	GO:0015171//amino acid transmembrane transporter activity	GO:0003333//amino acid transmembrane transport	gi|115397765|ref|XP_001214474.1|/0/amino-acid permease inda1 [Aspergillus terreus NIH2624]
4321873	3195	724.92	90.81	-2.313110637	8.66E-17	1.41E-15	Down	ATEG_06359	hypothetical protein	ko00562//Inositol phosphate metabolism;ko00053//Ascorbate and aldarate metabolism	-	GO:0005524//ATP binding	-	gi|115399896|ref|XP_001215537.1|/0/conserved hypothetical protein [Aspergillus terreus NIH2624]

4322493	1223	23.51	1.34	-2.310053467	0.01312182	0.03916095	Down	ATEG_07574	hypothetical protein	-	GO:0005634//nucleus	GO:0000981//RNA polymerase II transcription factor activity, sequence-specific DNA binding;GO:0008270//zinc ion binding	GO:0006357//regulation of transcription from RNA polymerase II promoter	gi|115401214|ref|XP_001216195.1|/0/predicted protein [Aspergillus terreus NIH2624]
4355094	801	61.64	6.3	-2.308505756	0.00059615	0.00257297	Down	ATEG_00342	non-heme chloroperoxidase	ko01100//Metabolic pathways;ko04146//Peroxisome;ko00590//Arachidonic acid metabolism	-	GO:0004601//peroxidase activity;GO:0016787//hydrolase activity	GO:0098869//cellular oxidant detoxification	gi|115491601|ref|XP_001210428.1|/0/non-heme chloroperoxidase [Aspergillus terreus NIH2624]
4318439	1083	26.34	1.78	-2.290434926	0.01051694	0.03236486	Down	ATEG_04011	hypothetical protein	-	-	-	-	gi|115391369|ref|XP_001213189.1|/0/conserved hypothetical protein [Aspergillus terreus NIH2624]
4354107	1704	54.33	5.4	-2.287531548	0.00138008	0.00546384	Down	ATEG_09757	similar to 4-coumarate:CoA ligase	ko01100//Metabolic pathways;ko01110//Biosynthesis of secondary metabolites;ko00360//Phenylalanine metabolism;ko00130//Ubiquinone and other terpenoid-quinone biosynthesis	-	GO:0003824//catalytic activity	GO:0008152//metabolic process	gi|115443144|ref|XP_001218379.1|/0/hypothetical protein ATEG_09757 [Aspergillus terreus NIH2624]
4322012	831	2635.96	344.08	-2.276303056	1.13E-36	5.00E-35	Down	ATEG_06807	hypothetical protein	ko04144//Endocytosis	-	GO:0003700//transcription factor activity, sequence-specific DNA binding;GO:0043565//sequence-specific DNA binding	GO:0006355//regulation of transcription, DNA-templated	gi|115400793|ref|XP_001215985.1|/0/predicted protein [Aspergillus terreus NIH2624]

4318236	1557	369.88	47.21	-2.274626998	6.14E-13	7.86E-12	Down	ATEG_03903	similar to n-alkane-inducible cytochrome P-450	ko00380//Tryptophan metabolism;ko00071//Fatty acid degradation	-	GO:0005506//iron ion binding;GO:0016712//oxidoreductase activity, acting on paired donors, with incorporation or reduction of molecular oxygen, reduced flavin or flavoprotein as one donor, and incorporation of one atom of oxygen;GO:0020037//heme binding	GO:0055114//oxidation-reduction process	gi|115391153|ref|XP_001213081.1|/0/hypothetical protein ATEG_03903 [Aspergillus terreus NIH2624]
4322390	1932	100.9	11.11	-2.273606212	0.00012447	0.00061341	Down	ATEG_06527	hypothetical protein	-	GO:0016021//integral component of membrane	GO:0016491//oxidoreductase activity	GO:0055114//oxidation-reduction process	gi|115400233|ref|XP_001215705.1|/0/conserved hypothetical protein [Aspergillus terreus NIH2624]
4353445	360	53.78	5.44	-2.272891501	0.00163122	0.00635678	Down	ATEG_08008	hypothetical protein	-	-	-	-	gi|115432984|ref|XP_001216629.1|/4.37784e-87/conserved hypothetical protein [Aspergillus terreus NIH2624]
4353086	2787	90.78	10.38	-2.272850972	8.09E-05	0.00041372	Down	ATEG_08399	hypothetical protein	ko01100//Metabolic pathways;ko00520//Amino sugar and nucleotide sugar metabolism	-	-	-	gi|115433767|ref|XP_001217020.1|/0/predicted protein [Aspergillus terreus NIH2624]
4355071	2379	25.79	1.82	-2.272299045	0.01148777	0.03496261	Down	ATEG_00319	hypothetical protein	-	-	GO:0005524//ATP binding;GO:0030983//mismatched DNA binding	GO:0006298//mismatch repair	gi|115491555|ref|XP_001210405.1|/0/predicted protein [Aspergillus terreus NIH2624]

4353433	525	72.35	7.59	-2.258526982	0.00078219	0.00327574	Down	ATEG_08200	hypothetical protein	ko01100//Metabolic pathways;ko01110//Biosynthesis of secondary metabolites;ko00500//Starch and sucrose metabolism;ko00460//Cyanoamino acid metabolism	GO:0005794//Golgi apparatus;GO:0005886//plasma membrane;GO:0016021//integral component of membrane;GO:0032126//eisosome;GO:0035838//growing cell tip;GO:0045121//membrane raft	-	GO:0070941//eisosome assembly;GO:0090002//establishment of protein localization to plasma membrane;GO:0090155//negative regulation of sphingolipid biosynthetic process	gi|115433368|ref|XP_001216821.1|/4.79786e-122/conserved hypothetical protein [Aspergillus terreus NIH2624]
4317931	3936	56.04	5.86	-2.256901814	0.00113904	0.00459484	Down	ATEG_03562	hypothetical protein	ko00500//Starch and sucrose metabolism	-	-	-	gi|115390470|ref|XP_001212740.1|/0/conserved hypothetical protein [Aspergillus terreus NIH2624]
4354505	972	1483.86	197.7	-2.256571523	4.00E-34	1.56E-32	Down	ATEG_09707	hypothetical protein	ko01100//Metabolic pathways;ko00051//Fructose and mannose metabolism;ko00650//Butanoate metabolism;ko00591//Linoleic acid metabolism	-	GO:0016491//oxidoreductase activity	GO:0055114//oxidation-reduction process	gi|115443044|ref|XP_001218329.1|/0/conserved hypothetical protein [Aspergillus terreus NIH2624]
4315867	399	22.44	1.36	-2.25610435	0.01585071	0.04636136	Down	ATEG_01789	hypothetical protein	ko04144//Endocytosis	-	-	-	gi|115385214|ref|XP_001209154.1|/6.17093e-100/predicted protein [Aspergillus terreus NIH2624]

4321187	1071	25.21	1.8	-2.251060001	0.01205961	0.0364312	Down	ATEG_04963	hypothetical protein	ko01100//Metabolic pathways;ko00520//Amino sugar and nucleotide sugar metabolism;ko00531//Glycosaminoglycan degradation;ko01501//beta-Lactam resistance	-	GO:0004553//hydrolase activity, hydrolyzing O-glycosyl compounds	GO:0005975//carbohydrate metabolic process	gi|115397099|ref|XP_001214141.1|/0/conserved hypothetical protein [Aspergillus terreus NIH2624]
4320609	792	59.99	6.37	-2.245872061	0.00137999	0.00546384	Down	ATEG_05167	hypothetical protein	ko04144//Endocytosis	GO:0000813//ESCRT I complex	GO:0032403//protein complex binding	GO:0006612//protein targeting to membrane;GO:0043328//protein targeting to vacuole involved in ubiquitin-dependent protein catabolic process via the multivesicular body sorting pathway	gi|115397507|ref|XP_001214345.1|/0/conserved hypothetical protein [Aspergillus terreus NIH2624]
4354870	810	3319.74	448.8	-2.237192779	5.01E-44	2.83E-42	Down	ATEG_00113	hypothetical protein	ko01100//Metabolic pathways;ko01110//Biosynthesis of secondary metabolites;ko00564//Glycerophospholipid metabolism;ko00565//Ether lipid metabolism	-	-	-	gi|115491143|ref|XP_001210199.1|/1.55707e-58/conserved hypothetical protein [Aspergillus terreus NIH2624]
4354895	1596	3393.59	458.68	-2.236913602	2.67E-39	1.29E-37	Down	ATEG_00138	hypothetical protein	-	GO:0016021//integral component of membrane	-	GO:0055085//transmembrane transport	gi|115491193|ref|XP_001210224.1|/0/conserved hypothetical protein [Aspergillus terreus NIH2624]

4320187	2282	56.07	5.84	-2.232475091	0.00165148	0.00642715	Down	ATEG_04720	hypothetical protein	ko00563//Glycosylphosphatidylinositol(GPI)-anchor biosynthesis	GO:0016021//integral component of membrane	GO:0003824//catalytic activity	GO:0008152//metabolic process	gi|115396518|ref|XP_001213898.1|/0/predicted protein [Aspergillus terreus NIH2624]
4322255	1380	31.35	2.31	-2.228820642	0.0142636	0.04207335	Down	ATEG_06462	hypothetical protein	-	-	-	-	gi|115400103|ref|XP_001215640.1|/0/predicted protein [Aspergillus terreus NIH2624]
4319633	1773	38.67	3.65	-2.22554352	0.00559123	0.0187903	Down	ATEG_10207	hypothetical protein	ko01100//Metabolic pathways;ko00500//Starch and sucrose metabolism	-	GO:0036459//thiol-dependent ubiquitinyl hydrolase activity	GO:0006511//ubiquitin-dependent protein catabolic process;GO:0016579//protein deubiquitination	gi|115385925|ref|XP_001209509.1|/0/predicted protein [Aspergillus terreus NIH2624]
4353654	1221	38.11	3.6	-2.22210624	0.00495574	0.01693978	Down	ATEG_09260	hypothetical protein	ko01100//Metabolic pathways;ko01110//Biosynthesis of secondary metabolites;ko00564//Glycerophospholipid metabolism	-	GO:0004609//phosphatidylserine decarboxylase activity	GO:0008654//phospholipid biosynthetic process	gi|115437720|ref|XP_001217882.1|/0/conserved hypothetical protein [Aspergillus terreus NIH2624]

4323229	1236	349.13	46.48	-2.22044811	9.04E-12	1.06E-10	Down	ATEG_08928	hypothetical protein	-	GO:0000790//nuclear chromatin;GO:0043596//nuclear replication fork	GO:0003682//chromatin binding;GO:0016407//acetyltransferase activity	GO:0006275//regulation of DNA replication;GO:0006302//double-strand break repair;GO:0007076//mitotic chromosome condensation;GO:0007088//regulation of mitotic nuclear division;GO:0018393//internal peptidyl-lysine acetylation;GO:0032200//telomere organization;GO:0034087//establishment of mitotic sister chromatid cohesion;GO:0070058//tRNA gene clustering	gi|115402875|ref|XP_001217514.1|/0/conserved hypothetical protein [Aspergillus terreus NIH2624]
4316246	1155	86.37	9.89	-2.220206476	0.00034426	0.0015711	Down	ATEG_01345	hypothetical protein	ko01100//Metabolic pathways;ko01110//Biosynthesis of secondary metabolites;ko00100//Steroid biosynthesis	-	-	-	gi|115384326|ref|XP_001208710.1|/0/predicted protein [Aspergillus terreus NIH2624]
4320415	1230	123.87	14.16	-2.213521673	0.00019164	0.00091555	Down	ATEG_04416	similar to trichodiene synthase	ko00909//Sesquiterpenoid and triterpenoid biosynthesis	-	GO:0045482//trichodiene synthase activity	GO:0016106//sesquiterpenoid biosynthetic process	gi|115395910|ref|XP_001213594.1|/0/hypothetical protein ATEG_04416 [Aspergillus terreus NIH2624]
4323230	1527	373.41	49.66	-2.211528115	3.03E-10	3.06E-09	Down	ATEG_08929	hypothetical protein	-	-	-	-	gi|115402877|ref|XP_001217515.1|/0/conserved hypothetical protein [Aspergillus terreus NIH2624]

4319079	2817	63.93	6.66	-2.209570976	0.00184517	0.00708977	Down	ATEG_07344	hypothetical protein	ko04111//Cell cycle - yeast;ko04113//Meiosis - yeast	-	GO:0004672//protein kinase activity;GO:0005524//ATP binding	GO:0006468//protein phosphorylation	gi|115386978|ref|XP_001210030.1|/0/predicted protein [Aspergillus terreus NIH2624]
4321096	1275	1959.55	269.95	-2.209416745	2.51E-38	1.19E-36	Down	ATEG_04999	hypothetical protein	-	-	GO:0050364	GO:0009820//alkaloid metabolic process;GO:0044249//cellular biosynthetic process;GO:0044550//secondary metabolite biosynthetic process;GO:0044763;GO:1901576	gi|115397171|ref|XP_001214177.1|/0/predicted protein [Aspergillus terreus NIH2624]
4318852	1614	211.21	27.33	-2.20279065	1.15E-07	9.01E-07	Down	ATEG_07116	hypothetical protein	ko01100//Metabolic pathways;ko00350//Tyrosine metabolism	-	GO:0016787//hydrolase activity	GO:0008152//metabolic process	gi|115386522|ref|XP_001209802.1|/0/predicted protein [Aspergillus terreus NIH2624]
4318829	3222	500.62	66.72	-2.202762436	3.03E-12	3.69E-11	Down	ATEG_07069	hypothetical protein	-	-	-	-	gi|115386428|ref|XP_001209755.1|/0/conserved hypothetical protein [Aspergillus terreus NIH2624]
4323440	1857	1269.96	173.96	-2.199268268	3.65E-25	1.00E-23	Down	ATEG_08635	hypothetical protein	ko01100//Metabolic pathways;ko00260//Glycine, serine and threonine metabolism	-	GO:0016614//oxidoreductase activity, acting on CH-OH group of donors;GO:0050660//flavin adenine dinucleotide binding	GO:0055114//oxidation-reduction process	gi|115402289|ref|XP_001217221.1|/0/predicted protein [Aspergillus terreus NIH2624]
4319087	990	94.69	11.14	-2.197661325	0.00018853	0.00090207	Down	ATEG_07364	hypothetical protein	-	-	-	-	gi|115387018|ref|XP_001210050.1|/0/predicted protein [Aspergillus terreus NIH2624]

4319250	1464	882.24	122.52	-2.19124637	9.79E-19	1.80E-17	Down	ATEG_07135	hypothetical protein	-	GO:0016021//integral component of membrane	-	-	gi|115386560|ref|XP_001209821.1|/0/predicted protein [Aspergillus terreus NIH2624]
4323005	825	118.82	15	-2.188908023	4.99E-05	0.00026509	Down	ATEG_07765	hypothetical protein	ko01100//Metabolic pathways;ko00520//Amino sugar and nucleotide sugar metabolism	GO:0016021//integral component of membrane	-	-	gi|115401596|ref|XP_001216386.1|/0/conserved hypothetical protein [Aspergillus terreus NIH2624]
4315760	4649	1023.23	141.24	-2.18586184	2.39E-22	5.61E-21	Down	ATEG_01071	hypothetical protein	-	GO:0016021//integral component of membrane	-	GO:0055085//transmembrane transport	gi|115383778|ref|XP_001208436.1|/0/conserved hypothetical protein [Aspergillus terreus NIH2624]
4355578	1587	53.25	5.52	-2.185666634	0.0049453	0.01691025	Down	ATEG_00817	hypothetical protein	ko01130//Biosynthesis of antibiotics;ko00900//Terpenoid backbone biosynthesis	-	GO:0016491//oxidoreductase activity	GO:0055114//oxidation-reduction process	gi|115492551|ref|XP_001210903.1|/0/conserved hypothetical protein [Aspergillus terreus NIH2624]
4353541	1541	474.67	65.49	-2.185093381	5.16E-14	7.03E-13	Down	ATEG_08218	hypothetical protein	-	-	-	-	gi|115433404|ref|XP_001216839.1|/3.31499e-95/predicted protein [Aspergillus terreus NIH2624]
4322381	1713	583.23	79.63	-2.181037984	4.20E-10	4.19E-09	Down	ATEG_06588	hypothetical protein	ko00500//Starch and sucrose metabolism	GO:0005886//plasma membrane;GO:0016021//integral component of membrane	GO:0042973//glucan endo-1,3-beta-D-glucosidase activity	GO:0000272//polysaccharide catabolic process;GO:0071555//cell wall organization	gi|115400355|ref|XP_001215766.1|/0/conserved hypothetical protein [Aspergillus terreus NIH2624]

4318850	1308	196.11	25.99	-2.167298029	2.59E-07	1.95E-06	Down	ATEG_07114	hypothetical protein	ko04113//Meiosis - yeast	GO:0005887//integral component of plasma membrane	GO:0005351//sugar:proton symporter activity;GO:0005355//glucose transmembrane transporter activity	GO:0035428//hexose transmembrane transport;GO:0046323//glucose import	gi|115386518|ref|XP_001209800.1|/0/predicted protein [Aspergillus terreus NIH2624]
4354613	1425	47.59	4.94	-2.158267447	0.00477031	0.01641961	Down	ATEG_10316	hypothetical protein	-	GO:0016021//integral component of membrane	-	GO:0055085//transmembrane transport	gi|115449663|ref|XP_001218664.1|/0/conserved hypothetical protein [Aspergillus terreus NIH2624]
4321558	1581	134.49	17.48	-2.155393122	9.75E-06	5.81E-05	Down	ATEG_06095	similar to high-affinity hexose transporter	ko04113//Meiosis - yeast	GO:0005737//cytoplasm;GO:0016021//integral component of membrane	GO:0022891//substrate-specific transmembrane transporter activity	GO:0008643//carbohydrate transport;GO:0055085//transmembrane transport;GO:0071333//cellular response to glucose stimulus	gi|115399368|ref|XP_001215273.1|/0/hypothetical protein ATEG_06095 [Aspergillus terreus NIH2624]
4323370	3106	2987.33	427.49	-2.151541234	4.69E-41	2.41E-39	Down	ATEG_08652	hypothetical protein	-	GO:0016021//integral component of membrane	GO:0008496//mannan endo-1,6-alpha-mannosidase activity	GO:0016052//carbohydrate catabolic process	gi|115402323|ref|XP_001217238.1|/0/conserved hypothetical protein [Aspergillus terreus NIH2624]
4320570	732	676.89	97.8	-2.128191357	6.22E-18	1.08E-16	Down	ATEG_05533	hypothetical protein	-	-	-	-	gi|115398239|ref|XP_001214711.1|/2.5666e-110/conserved hypothetical protein [Aspergillus terreus NIH2624]
4320439	1846	57.15	6.76	-2.121319665	0.00181368	0.0069888	Down	ATEG_04790	hypothetical protein	-	GO:0005634//nucleus	GO:0003677//DNA binding;GO:0008270//zinc ion binding	GO:0006351//transcription, DNA-templated	gi|115396658|ref|XP_001213968.1|/0/predicted protein [Aspergillus terreus NIH2624]

4317435	2929	454.61	65.49	-2.118167475	4.71E-12	5.66E-11	Down	ATEG_02941	hypothetical protein	-	-	-	-	gi|115389228|ref|XP_001212119.1|/0/conserved hypothetical protein [Aspergillus terreus NIH2624]
4355284	762	103.62	13.47	-2.112986821	0.00013779	0.00067304	Down	ATEG_00530	hypothetical protein	-	GO:0000124//SAGA complex;GO:0005671//Ada2/Gcn5/Ada3 transcription activator complex;GO:0005829//cytosol	GO:0001135//transcription factor activity, RNA polymerase II transcription factor recruiting;GO:0035064//methylated histone binding	GO:0034629//cellular protein complex localization;GO:0043970//histone H3-K9 acetylation;GO:0043971//histone H3-K18 acetylation;GO:0044154//histone H3-K14 acetylation;GO:0045944//positive regulation of transcription from RNA polymerase II promoter;GO:0070868//heterochromatin organization involved in chromatin silencing	gi|115491977|ref|XP_001210616.1|/2.44508e-179/conserved hypothetical protein [Aspergillus terreus NIH2624]
4321645	3096	50.43	5.52	-2.109937058	0.00724905	0.02352745	Down	ATEG_05735	hypothetical protein	-	-	-	-	gi|115398648|ref|XP_001214913.1|/0/predicted protein [Aspergillus terreus NIH2624]
4318480	3887	2919.86	429.36	-2.109133458	1.29E-25	3.60E-24	Down	ATEG_03946	hypothetical protein	ko04144//Endocytosis	-	-	-	gi|115391239|ref|XP_001213124.1|/0/conserved hypothetical protein [Aspergillus terreus NIH2624]

4318673	1368	60.55	6.46	-2.101935636	0.00964956	0.02995099	Down	ATEG_04028	hypothetical protein	ko01100//Metabolic pathways;ko01110//Biosynthesis of secondary metabolites	-	GO:0004497//monooxygenase activity;GO:0005506//iron ion binding;GO:0016705//oxidoreductase activity, acting on paired donors, with incorporation or reduction of molecular oxygen;GO:0020037//heme binding	GO:0055114//oxidation-reduction process	gi|115391403|ref|XP_001213206.1|/0/predicted protein [Aspergillus terreus NIH2624]
4354681	789	65.58	8.14	-2.100470377	0.00124219	0.00496865	Down	ATEG_10254	hypothetical protein	-	-	GO:0005524//ATP binding;GO:0008716//D-alanine-D-alanine ligase activity;GO:0046872//metal ion binding	-	gi|115449421|ref|XP_001218602.1|/0/predicted protein [Aspergillus terreus NIH2624]
4320539	2400	113.77	15.29	-2.099807387	4.45E-05	0.00023966	Down	ATEG_04606	hypothetical protein	ko01100//Metabolic pathways;ko00030//Pentose phosphate pathway	-	GO:0016832//aldehyde-lyase activity	GO:0005975//carbohydrate metabolic process	gi|115396290|ref|XP_001213784.1|/0/conserved hypothetical protein [Aspergillus terreus NIH2624]
4318688	648	2245.21	336.11	-2.091058271	4.61E-37	2.10E-35	Down	ATEG_03768	hypothetical protein	ko04144//Endocytosis	-	-	-	gi|115390883|ref|XP_001212946.1|/1.79201e-144/predicted protein [Aspergillus terreus NIH2624]
4316447	1107	56.63	6.81	-2.09097727	0.00282466	0.01034495	Down	ATEG_01892	hypothetical protein	-	-	GO:0003677//DNA binding;GO:0046983//protein dimerization activity	-	gi|115387129|ref|XP_001211070.1|/0/predicted protein [Aspergillus terreus NIH2624]

4355358	627	34.74	3.62	-2.089935586	0.0097437	0.0302032	Down	ATEG_00604	hypothetical protein	-	-	GO:0004733//pyridoxamine-phosphate oxidase activity;GO:0010181//FMN binding	GO:0055114//oxidation-reduction process	gi|115492125|ref|XP_001210690.1|/4.63831e-148/conserved hypothetical protein [Aspergillus terreus NIH2624]
4320375	1617	204.63	28.69	-2.087084648	8.89E-07	6.15E-06	Down	ATEG_04418	hypothetical protein	ko01100//Metabolic pathways;ko01110//Biosynthesis of secondary metabolites	GO:0016021//integral component of membrane	GO:0004497//monooxygenase activity;GO:0005506//iron ion binding;GO:0009055//electron carrier activity;GO:0016705//oxidoreductase activity, acting on paired donors, with incorporation or reduction of molecular oxygen;GO:0020037//heme binding	GO:0055114//oxidation-reduction process	gi|115395914|ref|XP_001213596.1|/0/conserved hypothetical protein [Aspergillus terreus NIH2624]
4315762	3408	95.32	12.35	-2.08359794	0.0011049	0.00447443	Down	ATEG_01073	hypothetical protein	ko01100//Metabolic pathways;ko04146//Peroxisome;ko00071//Fatty acid degradation;ko01212//Fatty acid metabolism;ko00061//Fatty acid biosynthesis	-	GO:0003824//catalytic activity	GO:0008152//metabolic process	gi|115383782|ref|XP_001208438.1|/0/predicted protein [Aspergillus terreus NIH2624]
4319240	2932	117.6	15.64	-2.071377661	0.00019233	0.0009184	Down	ATEG_07105	similar to MFS transporter	-	GO:0016021//integral component of membrane	-	GO:0055085//transmembrane transport	gi|115386500|ref|XP_001209791.1|/0/hypothetical protein ATEG_07105 [Aspergillus terreus NIH2624]

4317195	1647	1500.16	227.72	-2.069156115	2.08E-30	7.14E-29	Down	ATEG_02727	hypothetical protein	ko04111//Cell cycle - yeast	GO:0005634//nucleus	GO:0003677//DNA binding	-	gi|115388799|ref|XP_001211905.1|/0/predicted protein [Aspergillus terreus NIH2624]
4318372	2626	9329.91	1346.13	-2.067828962	5.67E-07	4.06E-06	Down	ATEG_04095	hypothetical protein	-	GO:0016021//integral component of membrane	-	-	gi|115391537|ref|XP_001213273.1|/0/conserved hypothetical protein [Aspergillus terreus NIH2624]
4315808	1974	577.67	86.91	-2.064476891	3.75E-15	5.44E-14	Down	ATEG_01219	hypothetical protein	ko01100//Metabolic pathways;ko04146//Peroxisome;ko00260//Glycine, serine and threonine metabolism;ko00310//Lysine degradation	GO:0016020//membrane	GO:0016491//oxidoreductase activity	GO:0055114//oxidation-reduction process	gi|115384074|ref|XP_001208584.1|/0/predicted protein [Aspergillus terreus NIH2624]
4320585	1158	28.02	2.7	-2.062023998	0.01608291	0.04692348	Down	ATEG_05548	hypothetical protein	ko04144//Endocytosis	-	-	-	gi|115398269|ref|XP_001214726.1|/0/predicted protein [Aspergillus terreus NIH2624]
4317651	3321	396.35	55.98	-2.059996646	6.00E-07	4.28E-06	Down	ATEG_02867	similar to DNA binding	ko04144//Endocytosis	-	GO:0005096//GTPase activator activity	GO:0043547//positive regulation of GTPase activity	gi|115389080|ref|XP_001212045.1|/0/hypothetical protein ATEG_02867 [Aspergillus terreus NIH2624]
4355535	1065	1317.74	202.76	-2.051738859	2.96E-22	6.92E-21	Down	ATEG_00776	hypothetical protein	ko04144//Endocytosis	-	-	-	gi|115492469|ref|XP_001210862.1|/0/conserved hypothetical protein [Aspergillus terreus NIH2624]

4317853	675	70.07	9.11	-2.046352452	0.00199258	0.00761875	Down	ATEG_03089	hypothetical protein	ko04144//Endocytosis	-	-	-	gi|115389524|ref|XP_001212267.1|/8.31258e-168/predicted protein [Aspergillus terreus NIH2624]
4353544	1047	303.68	45.58	-2.046020889	2.71E-09	2.52E-08	Down	ATEG_08122	hypothetical protein	ko00620//Pyruvate metabolism	-	GO:0016491//oxidoreductase activity;GO:0046872//metal ion binding	GO:0055114//oxidation-reduction process	gi|115433212|ref|XP_001216743.1|/0/conserved hypothetical protein [Aspergillus terreus NIH2624]
4355624	4697	390.51	58.18	-2.04198619	5.14E-10	5.07E-09	Down	ATEG_00863	hypothetical protein	ko03013//RNA transport	-	-	-	gi|115492643|ref|XP_001210949.1|/6.98627e-129/predicted protein [Aspergillus terreus NIH2624]
4319253	1985	81.79	10.7	-2.040561846	0.00086018	0.00356179	Down	ATEG_06868	hypothetical protein	-	-	-	-	gi|115386026|ref|XP_001209554.1|/0/predicted protein [Aspergillus terreus NIH2624]
4318481	2356	210.71	30.93	-2.036878934	3.46E-07	2.57E-06	Down	ATEG_03947	hypothetical protein	ko01100//Metabolic pathways;ko01220//Degradation of aromatic compounds	-	GO:0004497//monooxygenase activity;GO:0005506//iron ion binding;GO:0016705//oxidoreductase activity, acting on paired donors, with incorporation or reduction of molecular oxygen;GO:0020037//heme binding	GO:0055114//oxidation-reduction process	gi|115391241|ref|XP_001213125.1|/0/predicted protein [Aspergillus terreus NIH2624]
4320895	2283	429.82	65.74	-2.032738273	1.87E-12	2.31E-11	Down	ATEG_05091	D-lactate dehydrogenase	ko00620//Pyruvate metabolism	GO:0016021//integral component of membrane	GO:0016740//transferase activity	-	gi|115397355|ref|XP_001214269.1|/0/D-lactate dehydrogenase [Aspergillus terreus NIH2624]

4323498	966	1164.26	182.54	-2.021978034	1.39E-18	2.53E-17	Down	ATEG_08874	hypothetical protein	-	-	-	-	gi|115402767|ref|XP_001217460.1|/0/conserved hypothetical protein [Aspergillus terreus NIH2624]
4354169	1206	48.8	5.86	-2.019750641	0.00670157	0.02196328	Down	ATEG_09870	hypothetical protein	-	-	-	-	gi|115443370|ref|XP_001218492.1|/0/predicted protein [Aspergillus terreus NIH2624]
4319288	1896	30.28	3.16	-2.016914003	0.01652195	0.04795003	Down	ATEG_07141	similar to saponin hydrolase precursor	-	-	GO:0016787//hydrolase activity	-	gi|115386572|ref|XP_001209827.1|/0/hypothetical protein ATEG_07141 [Aspergillus terreus NIH2624]
4320991	2448	59.42	7.73	-2.016167194	0.00324572	0.01167289	Down	ATEG_04986	hypothetical protein	-	-	GO:0004672//protein kinase activity;GO:0005524//ATP binding	GO:0006468//protein phosphorylation	gi|115397145|ref|XP_001214164.1|/0/conserved hypothetical protein [Aspergillus terreus NIH2624]
4354697	1032	307.67	46.59	-2.013163194	1.01E-05	6.02E-05	Down	ATEG_10377	similar to oxidoreductase	ko00254//Aflatoxin biosynthesis	-	GO:0016491//oxidoreductase activity	GO:0055114//oxidation-reduction process	gi|115449899|ref|XP_001218725.1|/0/hypothetical protein ATEG_10377 [Aspergillus terreus NIH2624]
4322820	963	983.39	126.05	-2.012144039	0.00219983	0.00829624	Down	ATEG_07521	hypothetical protein	ko03040//Spliceosome	-	-	-	gi|115401108|ref|XP_001216142.1|/0/conserved hypothetical protein [Aspergillus terreus NIH2624]

4317440	579	36.44	4.11	-2.008798523	0.01355446	0.04027279	Down	ATEG_02807	hypothetical protein	-	-	-	-	gi|115388960|ref|XP_001211985.1|/1.35083e-139/conserved hypothetical protein [Aspergillus terreus NIH2624]
4322652	3908	4609.1	731.51	-2.00879226	2.08E-40	1.04E-38	Down	ATEG_07892	similar to alcohol dehydrogenase	ko01100//Metabolic pathways;ko00350//Tyrosine metabolism;ko00650//Butanoate metabolism;ko00310//Lysine degradation;ko00250//Alanine, aspartate and glutamate metabolism	-	GO:0016620//oxidoreductase activity, acting on the aldehyde or oxo group of donors, NAD or NADP as acceptor	GO:0055114//oxidation-reduction process	gi|115401850|ref|XP_001216513.1|/0/hypothetical protein ATEG_07892 [Aspergillus terreus NIH2624]
4318925	1428	233.1	34.82	-1.999194269	9.55E-07	6.58E-06	Down	ATEG_07095	hypothetical protein	ko00970//Aminoacyl-tRNA biosynthesis	-	-	-	gi|115386480|ref|XP_001209781.1|/0/predicted protein [Aspergillus terreus NIH2624]
4318091	717	56.04	7.29	-1.99871008	0.00457595	0.01583191	Down	ATEG_03314	hypothetical protein	-	-	GO:0008168//methyltransferase activity;GO:0050662//coenzyme binding	GO:0032259//methylation	gi|115389974|ref|XP_001212492.1|/1.36899e-173/conserved hypothetical protein [Aspergillus terreus NIH2624]
4319503	1230	560.98	87.99	-1.996962347	3.50E-14	4.80E-13	Down	ATEG_10113	hypothetical protein	ko01100//Metabolic pathways;ko00520//Amino sugar and nucleotide sugar metabolism	-	-	-	gi|115385737|ref|XP_001209415.1|/0/conserved hypothetical protein [Aspergillus terreus NIH2624]

4353358	1137	1651.29	264.63	-1.995668388	6.64E-23	1.59E-21	Down	ATEG_08031	plasma membrane iron permease	-	GO:0005783//endoplasmic reticulum;GO:0005794//Golgi apparatus;GO:0016021//integral component of membrane;GO:0033573//high-affinity iron permease complex	GO:0005381//iron ion transmembrane transporter activity	GO:0006827//high-affinity iron ion transmembrane transport;GO:0010106//cellular response to iron ion starvation	gi|115433030|ref|XP_001216652.1|/0/plasma membrane iron permease [Aspergillus terreus NIH2624]
4355353	1387	1995.21	318.64	-1.993603862	8.56E-29	2.73E-27	Down	ATEG_00599	hypothetical protein	-	GO:0016021//integral component of membrane	-	-	gi|115492115|ref|XP_001210685.1|/0/conserved hypothetical protein [Aspergillus terreus NIH2624]
4321100	1233	49.84	6.3	-1.982431083	0.0063553	0.02101192	Down	ATEG_05003	exoglucanase 1 precursor	ko01100//Metabolic pathways;ko00500//Starch and sucrose metabolism	GO:0005576//extracellular region	GO:0008810//cellulase activity;GO:0030248//cellulose binding	GO:0005975//carbohydrate metabolic process	gi|115397179|ref|XP_001214181.1|/0/exoglucanase 1 precursor [Aspergillus terreus NIH2624]
4318689	1545	288.22	44.58	-1.981351191	6.00E-06	3.70E-05	Down	ATEG_03769	hypothetical protein	-	-	-	-	gi|115390885|ref|XP_001212947.1|/4.20929e-136/predicted protein [Aspergillus terreus NIH2624]
4354721	1575	4329.99	705.68	-1.975741717	1.75E-35	7.38E-34	Down	ATEG_10385	hypothetical protein	ko04144//Endocytosis	GO:0000329//fungal-type vacuole membrane;GO:0005829//cytosol	-	GO:0006109//regulation of carbohydrate metabolic process;GO:0007039//protein catabolic process in the vacuole;GO:0042149//cellular response to glucose starvation;GO:0061587//transfer RNA gene-mediated silencing	gi|115449931|ref|XP_001218733.1|/0/conserved hypothetical protein [Aspergillus terreus NIH2624]

4354511	1404	132.29	19.93	-1.973412002	7.29E-05	0.00037638	Down	ATEG_09742	hypothetical protein	-	GO:0016021//integral component of membrane	-	-	gi|115443114|ref|XP_001218364.1|/0/predicted protein [Aspergillus terreus NIH2624]
4354176	3231	296.6	46.82	-1.958516579	1.06E-07	8.33E-07	Down	ATEG_09645	hypothetical protein	ko03013//RNA transport;ko03040//Spliceosome	GO:0000934//porous cell septum	-	GO:0009611//response to wounding	gi|115442920|ref|XP_001218267.1|/0/predicted protein [Aspergillus terreus NIH2624]
4323173	580	67.17	9.04	-1.956286647	0.00481566	0.01655142	Down	ATEG_08911	hypothetical protein	-	-	-	-	gi|115402841|ref|XP_001217497.1|/1.55197e-110/predicted protein [Aspergillus terreus NIH2624]
4321483	1755	53.81	7.24	-1.955638404	0.00477592	0.01643289	Down	ATEG_05802	glycerol kinase 2	ko01100//Metabolic pathways;ko00561//Glycerolipid metabolism	-	GO:0004370//glycerol kinase activity	GO:0005975//carbohydrate metabolic process;GO:0006072//glycerol-3-phosphate metabolic process;GO:0016310//phosphorylation	gi|115398782|ref|XP_001214980.1|/0/glycerol kinase 2 [Aspergillus terreus NIH2624]
4316832	2916	76.21	9.22	-1.95207833	0.00927675	0.0289374	Down	ATEG_02390	hypothetical protein	-	-	GO:0003824//catalytic activity	GO:0009116//nucleoside metabolic process	gi|115388125|ref|XP_001211568.1|/0/predicted protein [Aspergillus terreus NIH2624]
4323559	897	156.34	24.45	-1.945358946	2.13E-05	0.00012095	Down	ATEG_08501	hypothetical protein	ko01100//Metabolic pathways;ko00280//Valine, leucine and isoleucine degradation	-	GO:0004616//phosphogluconate dehydrogenase (decarboxylating) activity	GO:0055114//oxidation-reduction process	gi|115402021|ref|XP_001217087.1|/0/conserved hypothetical protein [Aspergillus terreus NIH2624]
4318921	1317	42.58	5.44	-1.944897914	0.01013858	0.03127229	Down	ATEG_06906	hypothetical protein	ko03013//RNA transport;ko03015//mRNA surveillance pathway	GO:0016021//integral component of membrane	-	-	gi|115386102|ref|XP_001209592.1|/0/predicted protein [Aspergillus terreus NIH2624]

4319076	1014	447.73	72.72	-1.940994959	1.34E-11	1.54E-10	Down	ATEG_06921	hypothetical protein	ko01100//Metabolic pathways;ko00051//Fructose and mannose metabolism;ko00650//Butanoate metabolism;ko00591//Linoleic acid metabolism	-	-	-	gi|115386132|ref|XP_001209607.1|/0/predicted protein [Aspergillus terreus NIH2624]
4323043	399	179.32	28.44	-1.937655206	3.91E-06	2.49E-05	Down	ATEG_07715	hypothetical protein	-	GO:0016021//integral component of membrane	-	-	gi|115401496|ref|XP_001216336.1|/2.15732e-91/conserved hypothetical protein [Aspergillus terreus NIH2624]
4320660	1008	53.25	7.27	-1.935888928	0.0058202	0.01944142	Down	ATEG_05135	similar to GliT	-	GO:0005576//extracellular region	GO:0016971//flavin-linked sulfhydryl oxidase activity;GO:0050660//flavin adenine dinucleotide binding	GO:0036146//cellular response to mycotoxin;GO:0055114//oxidation-reduction process;GO:2001310//gliotoxin biosynthetic process	gi|115397443|ref|XP_001214313.1|/0/hypothetical protein ATEG_05135 [Aspergillus terreus NIH2624]
4354532	5069	251.15	39.92	-1.935400546	3.70E-07	2.74E-06	Down	ATEG_09738	hypothetical protein	ko00970//Aminoacyl-tRNA biosynthesis	-	-	-	gi|115443106|ref|XP_001218360.1|/0/predicted protein [Aspergillus terreus NIH2624]
4317598	1995	59.97	8.24	-1.930731473	0.00692165	0.02259771	Down	ATEG_03393	hypothetical protein	-	GO:0016021//integral component of membrane	GO:0015171//amino acid transmembrane transporter activity	GO:0003333//amino acid transmembrane transport	gi|115390132|ref|XP_001212571.1|/0/predicted protein [Aspergillus terreus NIH2624]
4318223	975	298.73	48.79	-1.929727531	1.93E-08	1.64E-07	Down	ATEG_03967	hypothetical protein	-	-	-	-	gi|115391281|ref|XP_001213145.1|/0/predicted protein [Aspergillus terreus NIH2624]

4353820	1962	50.46	6.81	-1.927530769	0.0073593	0.02382756	Down	ATEG_09124	hypothetical protein	ko01100//Metabolic pathways;ko01110//Biosynthesis of secondary metabolites;ko01130//Biosynthesis of antibiotics;ko00010//Glycolysis / Gluconeogenesis	GO:0016021//integral component of membrane	GO:0000287//magnesium ion binding;GO:0016831//carboxy-lyase activity;GO:0030976//thiamine pyrophosphate binding	-	gi|115437180|ref|XP_001217746.1|/0/predicted protein [Aspergillus terreus NIH2624]
4317482	2242	69.53	9.97	-1.926994003	0.00280071	0.01026125	Down	ATEG_03438	hypothetical protein	ko01100//Metabolic pathways;ko00051//Fructose and mannose metabolism;ko00650//Butanoate metabolism;ko00591//Linoleic acid metabolism	-	-	-	gi|115390222|ref|XP_001212616.1|/0/conserved hypothetical protein [Aspergillus terreus NIH2624]
4323154	4582	183.24	29.34	-1.926319442	3.56E-06	2.29E-05	Down	ATEG_08878	ABC transporter CDR4	ko02010//ABC transporters	GO:0016021//integral component of membrane	GO:0005524//ATP binding;GO:0042626//ATPase activity, coupled to transmembrane movement of substances	GO:0055085//transmembrane transport	gi|115402775|ref|XP_001217464.1|/0/ABC transporter CDR4 [Aspergillus terreus NIH2624]
4354594	1068	95.26	14.42	-1.925999026	0.0003958	0.00177779	Down	ATEG_10353	hypothetical protein	-	-	GO:0016787//hydrolase activity	GO:0008152//metabolic process	gi|115449809|ref|XP_001218701.1|/0/conserved hypothetical protein [Aspergillus terreus NIH2624]

4318595	567	3802.74	645.84	-1.915631292	1.04E-39	5.09E-38	Down	ATEG_04161	glutathione peroxidase	ko00480//Glutathione metabolism;ko00590//Arachidonic acid metabolism	GO:0005634//nucleus;GO:0005739//mitochondrion;GO:0005829//cytosol	GO:0004602//glutathione peroxidase activity;GO:0008379//thioredoxin peroxidase activity	GO:0042744//hydrogen peroxide catabolic process;GO:0045454//cell redox homeostasis;GO:0055114//oxidation-reduction process;GO:0070301//cellular response to hydrogen peroxide;GO:0098869//cellular oxidant detoxification	gi|115391669|ref|XP_001213339.1|/1.58354e-136/glutathione peroxidase [Aspergillus terreus NIH2624]
4323199	1050	364.71	60.53	-1.913886495	5.10E-09	4.60E-08	Down	ATEG_08661	hypothetical protein	-	-	GO:0008270//zinc ion binding;GO:0016491//oxidoreductase activity	GO:0055114//oxidation-reduction process	gi|115402341|ref|XP_001217247.1|/0/predicted protein [Aspergillus terreus NIH2624]
4317098	798	64.99	9.16	-1.909929066	0.00702183	0.02290087	Down	ATEG_02132	hypothetical protein	ko01100//Metabolic pathways;ko01110//Biosynthesis of secondary metabolites;ko01130//Biosynthesis of antibiotics;ko00100//Steroid biosynthesis	GO:0005622//intracellular	GO:0008168//methyltransferase activity	GO:0032259//methylation	gi|115387609|ref|XP_001211310.1|/0/conserved hypothetical protein [Aspergillus terreus NIH2624]
4322141	2291	41.46	5.37	-1.908711822	0.01110937	0.03395414	Down	ATEG_06461	hypothetical protein	ko01100//Metabolic pathways;ko00600//Sphingolipid metabolism	-	-	-	gi|115400101|ref|XP_001215639.1|/0/predicted protein [Aspergillus terreus NIH2624]
4321275	1760	500.45	83.56	-1.90833065	1.68E-12	2.08E-11	Down	ATEG_06125	hypothetical protein	ko00380//Tryptophan metabolism;ko00360//Phenylalanine metabolism;ko00330//Arginine and proline metabolism	-	GO:0016884//carbon-nitrogen ligase activity, with glutamine as amido-N-donor	-	gi|115399428|ref|XP_001215303.1|/0/conserved hypothetical protein [Aspergillus terreus NIH2624]

4321205	1239	305.88	50.78	-1.905362512	3.86E-07	2.85E-06	Down	ATEG_04940	similar to glycerophosphoryl diester phosphodiesterase family protein	ko00564//Glycerophospholipid metabolism	-	GO:0008889//glycerophosphodiester phosphodiesterase activity	GO:0006629//lipid metabolic process	gi|115397053|ref|XP_001214118.1|/0/hypothetical protein ATEG_04940 [Aspergillus terreus NIH2624]
4322972	900	44.87	5.84	-1.904566371	0.01152611	0.03503313	Down	ATEG_07498	hypothetical protein	ko01220//Degradation of aromatic compounds	-	GO:0016491//oxidoreductase activity	GO:0055114//oxidation-reduction process	gi|115401062|ref|XP_001216119.1|/0/conserved hypothetical protein [Aspergillus terreus NIH2624]
4317052	1254	1613.02	277.97	-1.893685176	4.67E-19	8.87E-18	Down	ATEG_02166	hypothetical protein	ko04144//Endocytosis	GO:0016021//integral component of membrane	-	-	gi|115387677|ref|XP_001211344.1|/0/predicted protein [Aspergillus terreus NIH2624]
4319221	4641	2351.42	405.97	-1.893175575	1.11E-18	2.03E-17	Down	ATEG_07017	similar to ABC transporter	ko02010//ABC transporters	GO:0016021//integral component of membrane	GO:0005524//ATP binding;GO:0042626//ATPase activity, coupled to transmembrane movement of substances	GO:0055085//transmembrane transport	gi|115386324|ref|XP_001209703.1|/0/hypothetical protein ATEG_07017 [Aspergillus terreus NIH2624]
4315603	645	189.9	31.12	-1.889575137	8.21E-06	4.93E-05	Down	ATEG_01077	hypothetical protein	ko00480//Glutathione metabolism	-	GO:0016740//transferase activity	-	gi|115383790|ref|XP_001208442.1|/7.97757e-152/predicted protein [Aspergillus terreus NIH2624]
4353682	1926	1026.84	176.4	-1.885558331	4.29E-19	8.18E-18	Down	ATEG_09085	hypothetical protein	-	GO:0016021//integral component of membrane	GO:0047686	-	gi|115437028|ref|XP_001217707.1|/0/conserved hypothetical protein [Aspergillus terreus NIH2624]

4321926	5091	5350.97	932.42	-1.878819697	3.11E-46	1.87E-44	Down	ATEG_06589	hypothetical protein	ko04144//Endocytosis	GO:0005840//ribosome	-	-	gi|115400357|ref|XP_001215767.1|/0/conserved hypothetical protein [Aspergillus terreus NIH2624]
4355489	2243	432.08	74.09	-1.8776591	1.93E-10	1.99E-09	Down	ATEG_00734	similar to alpha-mannosidase IC	ko01100//Metabolic pathways;ko04141//Protein processing in endoplasmic reticulum;ko00510//N-Glycan biosynthesis;ko00513//Various types of N-glycan biosynthesis	GO:0016021//integral component of membrane	GO:0004571//mannosyl-oligosaccharide 1,2-alpha-mannosidase activity;GO:0005509//calcium ion binding	GO:0008152//metabolic process	gi|115492385|ref|XP_001210820.1|/0/hypothetical protein ATEG_00734 [Aspergillus terreus NIH2624]
4354865	911	51.53	7.29	-1.872583055	0.00996605	0.03081101	Down	ATEG_00108	similar to glyoxalase family protein	ko04011//MAPK signaling pathway - yeast;ko00620//Pyruvate metabolism	-	-	-	gi|115491133|ref|XP_001210194.1|/1.4123e-95/hypothetical protein ATEG_00108 [Aspergillus terreus NIH2624]
4316004	1881	460.63	78.46	-1.87037314	8.32E-11	8.94E-10	Down	ATEG_01253	hypothetical protein	ko01100//Metabolic pathways;ko00650//Butanoate metabolism;ko00360//Phenylalanine metabolism	-	GO:0003857//3-hydroxyacyl-CoA dehydrogenase activity	GO:0006631//fatty acid metabolic process;GO:0055114//oxidation-reduction process	gi|115384142|ref|XP_001208618.1|/3.05296e-136/predicted protein [Aspergillus terreus NIH2624]
4318853	2358	182.19	30.29	-1.87006602	1.45E-05	8.43E-05	Down	ATEG_07117	hypothetical protein	ko04111//Cell cycle - yeast;ko04113//Meiosis - yeast	GO:0005634//nucleus	GO:0000981//RNA polymerase II transcription factor activity, sequence-specific DNA binding;GO:0003677//DNA binding;GO:0008270//zinc ion binding	GO:0006357//regulation of transcription from RNA polymerase II promoter	gi|115386524|ref|XP_001209803.1|/0/predicted protein [Aspergillus terreus NIH2624]

4321668	1481	2079.53	365.32	-1.869164994	4.73E-25	1.28E-23	Down	ATEG_05909	hypothetical protein	ko04144//Endocytosis	GO:0016021//integral component of membrane	-	-	gi|115398996|ref|XP_001215087.1|/0/predicted protein [Aspergillus terreus NIH2624]
4319361	630	6391.39	1127.18	-1.865532753	1.69E-45	9.84E-44	Down	ATEG_07125	similar to IgE-binding protein	ko04144//Endocytosis	-	-	-	gi|115386540|ref|XP_001209811.1|/5.87072e-156/hypothetical protein ATEG_07125 [Aspergillus terreus NIH2624]
4322078	3756	47.62	6.78	-1.859047542	0.00945472	0.02945341	Down	ATEG_06843	hypothetical protein	ko02010//ABC transporters	GO:0016021//integral component of membrane	GO:0005524//ATP binding;GO:0042626//ATPase activity, coupled to transmembrane movement of substances	GO:0055085//transmembrane transport	gi|115400865|ref|XP_001216021.1|/0/predicted protein [Aspergillus terreus NIH2624]
4318204	3434	865.3	152.01	-1.858004262	4.24E-17	7.06E-16	Down	ATEG_03710	hypothetical protein	-	GO:0005634//nucleus	GO:0000981//RNA polymerase II transcription factor activity, sequence-specific DNA binding;GO:0003677//DNA binding;GO:0008270//zinc ion binding	GO:0006357//regulation of transcription from RNA polymerase II promoter	gi|115390767|ref|XP_001212888.1|/0/conserved hypothetical protein [Aspergillus terreus NIH2624]
4353870	1320	234.35	39.6	-1.85423332	2.03E-06	1.35E-05	Down	ATEG_09164	hypothetical protein	-	GO:0016021//integral component of membrane	GO:0016747//transferase activity, transferring acyl groups other than amino-acyl groups	-	gi|115437342|ref|XP_001217786.1|/0/predicted protein [Aspergillus terreus NIH2624]
4321210	1812	226.95	38.82	-1.851352427	8.99E-07	6.21E-06	Down	ATEG_04945	hypothetical protein	ko00051//Fructose and mannose metabolism	-	GO:0016757//transferase activity, transferring glycosyl groups	GO:0006486//protein glycosylation	gi|115397063|ref|XP_001214123.1|/0/predicted protein [Aspergillus terreus NIH2624]

4322993	879	493.12	86.45	-1.845601367	6.04E-12	7.20E-11	Down	ATEG_07513	hypothetical protein	-	GO:0016021//integral component of membrane	-	-	gi|115401092|ref|XP_001216134.1|/0/predicted protein [Aspergillus terreus NIH2624]
4353351	1785	107.02	17.6	-1.837076457	0.00036813	0.00167028	Down	ATEG_08030	hypothetical protein	ko04144//Endocytosis	GO:0016021//integral component of membrane	-	-	gi|115433028|ref|XP_001216651.1|/0/predicted protein [Aspergillus terreus NIH2624]
4315598	1485	4270.87	766.38	-1.836077746	6.66E-37	3.02E-35	Down	ATEG_01851	UDP-N-acetylglucosamine pyrophosphorylase	ko01100//Metabolic pathways;ko01130//Biosynthesis of antibiotics;ko00520//Amino sugar and nucleotide sugar metabolism	-	GO:0003977//UDP-N-acetylglucosamine diphosphorylase activity	GO:0008152//metabolic process	gi|115385338|ref|XP_001209216.1|/0/UDP-N-acetylglucosamine pyrophosphorylase [Aspergillus terreus NIH2624]
4317483	1308	81.86	12.45	-1.830077393	0.00327684	0.01177129	Down	ATEG_03439	hypothetical protein	-	-	GO:0008171//O-methyltransferase activity;GO:0008757//S-adenosylmethionine-dependent methyltransferase activity	GO:0019438//aromatic compound biosynthetic process;GO:0032259//methylation	gi|115390224|ref|XP_001212617.1|/0/conserved hypothetical protein [Aspergillus terreus NIH2624]
4322369	1137	60.5	9.14	-1.827783735	0.00910247	0.02853609	Down	ATEG_06245	hypothetical protein	-	-	-	-	gi|115399592|ref|XP_001215423.1|/0/predicted protein [Aspergillus terreus NIH2624]
4323197	4251	317.77	55.94	-1.827540817	1.94E-08	1.64E-07	Down	ATEG_08659	hypothetical protein	ko01100//Metabolic pathways;ko00520//Amino sugar and nucleotide sugar metabolism	-	-	-	gi|115402337|ref|XP_001217245.1|/0/predicted protein [Aspergillus terreus NIH2624]
4321035	1329	218.16	36.13	-1.823910436	9.66E-05	0.00048655	Down	ATEG_05288	hypothetical protein	ko04144//Endocytosis	GO:0016021//integral component of membrane	-	-	gi|115397749|ref|XP_001214466.1|/0/conserved hypothetical protein [Aspergillus terreus NIH2624]

4323499	1500	87.48	13.33	-1.819372331	0.00384243	0.01355906	Down	ATEG_08933	hypothetical protein	-	-	-	-	gi|115402885|ref|XP_001217519.1|/0/predicted protein [Aspergillus terreus NIH2624]
4353547	510	137.82	22.76	-1.818109208	0.00023583	0.00110752	Down	ATEG_08125	hypothetical protein	-	-	-	-	gi|115433218|ref|XP_001216746.1|/3.41459e-124/conserved hypothetical protein [Aspergillus terreus NIH2624]
4353137	549	242.49	41.09	-1.815356367	3.03E-05	0.00016797	Down	ATEG_08095	hypothetical protein	ko01100//Metabolic pathways;ko01130//Biosynthesis of antibiotics;ko01230//Biosynthesis of amino acids;ko01210//2-Oxocarboxylic acid metabolism;ko00300//Lysine biosynthesis	-	-	-	gi|115433158|ref|XP_001216716.1|/1.3641e-134/predicted protein [Aspergillus terreus NIH2624]
4353356	885	370.42	64.29	-1.815286033	1.44E-07	1.12E-06	Down	ATEG_08040	hypothetical protein	-	GO:0005634//nucleus;GO:0005737//cytoplasm	GO:0004864//protein phosphatase inhibitor activity;GO:0008047//enzyme activator activity	GO:0007059//chromosome segregation;GO:0009966//regulation of signal transduction;GO:0043085//positive regulation of catalytic activity;GO:0043086//negative regulation of catalytic activity;GO:0043666//regulation of phosphoprotein phosphatase activity	gi|115433048|ref|XP_001216661.1|/0/conserved hypothetical protein [Aspergillus terreus NIH2624]

4319069	2432	878.63	159.1	-1.806824505	8.33E-17	1.36E-15	Down	ATEG_07173	hypothetical protein	-	-	-	-	gi|115386636|ref|XP_001209859.1|/0/predicted protein [Aspergillus terreus NIH2624]
4355537	1284	43.15	6.32	-1.804066256	0.01360901	0.0403965	Down	ATEG_00778	hypothetical protein	-	-	GO:0004812//aminoacyl-tRNA ligase activity;GO:0005524//ATP binding;GO:0016787//hydrolase activity	GO:0006418//tRNA aminoacylation for protein translation	gi|115492473|ref|XP_001210864.1|/0/predicted protein [Aspergillus terreus NIH2624]
4321968	612	298.88	52.45	-1.80297393	0.00010346	0.00051721	Down	ATEG_06692	hypothetical protein	ko01100//Metabolic pathways;ko01110//Biosynthesis of secondary metabolites;ko00500//Starch and sucrose metabolism;ko00460//Cyanoamino acid metabolism	-	-	-	gi|115400563|ref|XP_001215870.1|/1.82853e-147/conserved hypothetical protein [Aspergillus terreus NIH2624]
4354190	577	147.49	24.61	-1.802750225	0.00033857	0.00154967	Down	ATEG_09517	similar to high affinity copper transporter	-	GO:0016021//integral component of membrane	GO:0005375//copper ion transmembrane transporter activity	GO:0035434//copper ion transmembrane transport	gi|115442664|ref|XP_001218139.1|/1.98548e-144/hypothetical protein ATEG_09517 [Aspergillus terreus NIH2624]
4320189	991	785.64	142.3	-1.800698771	4.54E-15	6.54E-14	Down	ATEG_04722	hypothetical protein	-	-	-	-	gi|115396522|ref|XP_001213900.1|/3.27947e-150/predicted protein [Aspergillus terreus NIH2624]
4355651	1377	81.23	12.4	-1.798682145	0.00535358	0.01811491	Down	ATEG_00890	hypothetical protein	ko00500//Starch and sucrose metabolism;ko04011//MAPK signaling pathway - yeast	-	-	GO:0019748//secondary metabolic process	gi|115492697|ref|XP_001210976.1|/0/predicted protein [Aspergillus terreus NIH2624]

4316479	1059	1189.16	218.04	-1.78654923	2.38E-18	4.29E-17	Down	ATEG_02494	hypothetical protein	ko01100//Metabolic pathways;ko01130//Biosynthesis of antibiotics;ko04146//Peroxisome;ko00260//Glycine, serine and threonine metabolism;ko00330//Arginine and proline metabolism;ko00311//Penicillin and cephalosporin biosynthesis;ko00472//D-Arginine and D-ornithine metabolism	-	GO:0003884//D-amino-acid oxidase activity;GO:0071949//FAD binding	GO:0046416//D-amino acid metabolic process;GO:0055114//oxidation-reduction process	gi|115388333|ref|XP_001211672.1|/0/conserved hypothetical protein [Aspergillus terreus NIH2624]
4354019	531	50.42	7.59	-1.778770249	0.01143778	0.03482177	Down	ATEG_09234	hypothetical protein	-	GO:0016021//integral component of membrane	-	-	gi|115437618|ref|XP_001217856.1|/1.00574e-124/predicted protein [Aspergillus terreus NIH2624]
4354504	1536	112.14	18.77	-1.777651109	0.00089996	0.00370529	Down	ATEG_09706	hypothetical protein	ko03040//Spliceosome	-	-	-	gi|115443042|ref|XP_001218328.1|/0/predicted protein [Aspergillus terreus NIH2624]
4319544	901	1302.71	242.02	-1.774227895	2.57E-18	4.61E-17	Down	ATEG_09959	hypothetical protein	-	GO:0016021//integral component of membrane	-	-	gi|115385429|ref|XP_001209261.1|/0/predicted protein [Aspergillus terreus NIH2624]
4354119	4248	2053.99	387.29	-1.767440091	6.96E-19	1.29E-17	Down	ATEG_09776	hypothetical protein	-	-	GO:0016491//oxidoreductase activity	GO:0055114//oxidation-reduction process	gi|115443182|ref|XP_001218398.1|/0/predicted protein [Aspergillus terreus NIH2624]

4317679	7863	77.39	12.55	-1.766902048	0.00433459	0.01510828	Down	ATEG_03446	similar to polyketide synthase	-	-	GO:0008270//zinc ion binding;GO:0016491//oxidoreductase activity;GO:0016747//transferase activity, transferring acyl groups other than amino-acyl groups	GO:0045461;GO:0055114//oxidation-reduction process;GO:1900554;GO:1900557;GO:1900584;GO:1900815;GO:1902086//fumagillin biosynthetic process	gi|115390238|ref|XP_001212624.1|/0/hypothetical protein ATEG_03446 [Aspergillus terreus NIH2624]
4317328	951	219.68	39.44	-1.764153522	4.19E-06	2.65E-05	Down	ATEG_02968	hypothetical protein	-	-	GO:0003824//catalytic activity	GO:0008152//metabolic process	gi|115389282|ref|XP_001212146.1|/0/predicted protein [Aspergillus terreus NIH2624]
4317469	1050	516.02	96.05	-1.764061412	8.51E-11	9.14E-10	Down	ATEG_02927	arabinogalactan endo-1,4-beta-galactosidase precursor	-	GO:0005576//extracellular region	GO:0015926//glucosidase activity;GO:0031218	GO:0045490//pectin catabolic process;GO:0071555//cell wall organization	gi|115389200|ref|XP_001212105.1|/0/arabinogalactan endo-1,4-beta-galactosidase precursor [Aspergillus terreus NIH2624]
4316108	2947	412.56	75.93	-1.762563532	3.43E-09	3.16E-08	Down	ATEG_01714	hypothetical protein	ko01100//Metabolic pathways;ko01110//Biosynthesis of secondary metabolites;ko00500//Starch and sucrose metabolism;ko00460//Cyanoamino acid metabolism	-	-	-	gi|115385064|ref|XP_001209079.1|/3.16492e-147/predicted protein [Aspergillus terreus NIH2624]
4323543	1543	64.48	10.26	-1.75318576	0.00733975	0.02377981	Down	ATEG_08589	hypothetical protein	ko01100//Metabolic pathways;ko00520//Amino sugar and nucleotide sugar metabolism	-	-	-	gi|115402197|ref|XP_001217175.1|/0/predicted protein [Aspergillus terreus NIH2624]

4316189	777	200.06	35.16	-1.74886726	9.87E-05	0.00049571	Down	ATEG_01064	similar to potential transcriptional repressor subunit	ko00310//Lysine degradation	GO:0017054//negative cofactor 2 complex	GO:0001047//core promoter binding;GO:0001129//RNA polymerase II transcription factor activity, TBP-class protein binding, involved in preinitiation complex assembly;GO:0003682//chromatin binding;GO:0003713//transcription coactivator activity;GO:0003714//transcription corepressor activity;GO:0046982//protein heterodimerization activity	GO:0000122//negative regulation of transcription from RNA polymerase II promoter;GO:0051123//RNA polymerase II transcriptional preinitiation complex assembly;GO:0061408//positive regulation of transcription from RNA polymerase II promoter in response to heat stress	gi|115383764|ref|XP_001208429.1|/0/hypothetical protein ATEG_01064 [Aspergillus terreus NIH2624]
4318312	1689	74.54	12.64	-1.747353773	0.00315724	0.01140276	Down	ATEG_04036	hypothetical protein	-	GO:0000444//MIS12/MIND type complex;GO:0000941//condensed nuclear chromosome inner kinetochore;GO:0031617//NMS complex	-	GO:0000070//mitotic sister chromatid segregation;GO:0051301//cell division	gi|115391419|ref|XP_001213214.1|/0/predicted protein [Aspergillus terreus NIH2624]
4318834	1952	482.65	91.38	-1.738329349	1.04E-08	9.02E-08	Down	ATEG_07249	hypothetical protein	ko01100//Metabolic pathways;ko01110//Biosynthesis of secondary metabolites;ko01230//Biosynthesis of amino acids;ko00270//Cysteine and methionine metabolism;ko00450//Selenocompound metabolism	-	GO:0003871//5-methyltetrahydropteroyltriglutamate-homocysteine S-methyltransferase activity;GO:0008270//zinc ion binding	GO:0009086//methionine biosynthetic process;GO:0032259//methylation	gi|115386788|ref|XP_001209935.1|/0/conserved hypothetical protein [Aspergillus terreus NIH2624]

4319401	1635	2663.63	507.65	-1.737702402	3.22E-23	7.86E-22	Down	ATEG_10020	hypothetical protein	-	GO:0005887//integral component of plasma membrane	GO:0015179//L-amino acid transmembrane transporter activity;GO:0015297//antiporter activity	GO:1902475//L-alpha-amino acid transmembrane transport	gi|115385551|ref|XP_001209322.1|/0/conserved hypothetical protein [Aspergillus terreus NIH2624]
4322461	2355	398.95	75.24	-1.737623919	2.94E-09	2.73E-08	Down	ATEG_06741	pre-mRNA splicing factor cef-1	ko03040//Spliceosome	-	GO:0003677//DNA binding	-	gi|115400661|ref|XP_001215919.1|/0/pre-mRNA splicing factor cef-1 [Aspergillus terreus NIH2624]
4318572	492	794.51	151.86	-1.735987652	5.50E-08	4.47E-07	Down	ATEG_04186	hypothetical protein	-	-	GO:0016846//carbon-sulfur lyase activity	GO:0008152//metabolic process	gi|115391719|ref|XP_001213364.1|/1.57859e-126/conserved hypothetical protein [Aspergillus terreus NIH2624]
4354194	1071	106.47	18.91	-1.733032882	0.00064559	0.00276856	Down	ATEG_09521	hypothetical protein	-	-	-	-	gi|115442672|ref|XP_001218143.1|/0/conserved hypothetical protein [Aspergillus terreus NIH2624]

4319534	1794	90.24	15.87	-1.727494667	0.00226111	0.00850001	Down	ATEG_10170	similar to dihydroxy-acid dehydratase	ko01100//Metabolic pathways;ko01110//Biosynthesis of secondary metabolites;ko01130//Biosynthesis of antibiotics;ko01230//Biosynthesis of amino acids;ko01210//2-Oxocarboxylic acid metabolism;ko00770//Pantothenate and CoA biosynthesis;ko00290//Valine, leucine and isoleucine biosynthesis	GO:0005622//intracellular	GO:0004160//dihydroxy-acid dehydratase activity;GO:0008869//galactonate dehydratase activity	GO:0009082//branched-chain amino acid biosynthetic process;GO:0019583//galactonate metabolic process	gi|115385851|ref|XP_001209472.1|/0/hypothetical protein ATEG_10170 [Aspergillus terreus NIH2624]
4353391	1769	90.27	15.53	-1.727029893	0.00612902	0.02035704	Down	ATEG_08049	hypothetical protein	-	-	-	-	gi|115433066|ref|XP_001216670.1|/0/predicted protein [Aspergillus terreus NIH2624]
4318948	1269	311.01	59.19	-1.720701119	2.00E-07	1.53E-06	Down	ATEG_07094	hypothetical protein	ko01100//Metabolic pathways	GO:0016020//membrane	-	-	gi|115386478|ref|XP_001209780.1|/0/predicted protein [Aspergillus terreus NIH2624]
4317755	615	174.77	32.45	-1.714628489	6.19E-05	0.00032366	Down	ATEG_03259	hypothetical protein	-	GO:0005840//ribosome	-	-	gi|115389864|ref|XP_001212437.1|/1.27821e-143/predicted protein [Aspergillus terreus NIH2624]
4321101	3283	307.68	58.96	-1.703792661	1.18E-07	9.28E-07	Down	ATEG_05371	hypothetical protein	-	GO:0016021//integral component of membrane	-	GO:0055085//transmembrane transport	gi|115397915|ref|XP_001214549.1|/0/conserved hypothetical protein [Aspergillus terreus NIH2624]

4321264	1023	63.31	10.89	-1.702128673	0.00815307	0.02604708	Down	ATEG_05936	hypothetical protein	ko01100//Metabolic pathways;ko00051//Fructose and mannose metabolism;ko00650//Butanoate metabolism;ko00591//Linoleic acid metabolism	-	GO:0016491//oxidoreductase activity	GO:0055114//oxidation-reduction process	gi|115399050|ref|XP_001215114.1|/0/conserved hypothetical protein [Aspergillus terreus NIH2624]
4316488	3912	210.8	39.41	-1.69402909	3.92E-05	0.00021371	Down	ATEG_02036	hypothetical protein	ko01100//Metabolic pathways;ko00590//Arachidonic acid metabolism	-	GO:0004601//peroxidase activity;GO:0005506//iron ion binding;GO:0016705//oxidoreductase activity, acting on paired donors, with incorporation or reduction of molecular oxygen;GO:0020037//heme binding	GO:0006979//response to oxidative stress;GO:0055114//oxidation-reduction process;GO:0098869//cellular oxidant detoxification	gi|115387417|ref|XP_001211214.1|/0/conserved hypothetical protein [Aspergillus terreus NIH2624]
4354890	2735	1614.56	319.28	-1.6935097	6.38E-23	1.53E-21	Down	ATEG_00133	hypothetical protein	-	GO:0005634//nucleus	GO:0000981//RNA polymerase II transcription factor activity, sequence-specific DNA binding;GO:0003677//DNA binding;GO:0008270//zinc ion binding	GO:0006357//regulation of transcription from RNA polymerase II promoter	gi|115491183|ref|XP_001210219.1|/0/conserved hypothetical protein [Aspergillus terreus NIH2624]
4321738	663	319.51	61.94	-1.692888736	6.36E-07	4.52E-06	Down	ATEG_06060	hypothetical protein	ko04144//Endocytosis	-	-	-	gi|115399298|ref|XP_001215238.1|/8.66733e-168/conserved hypothetical protein [Aspergillus terreus NIH2624]

4353937	2324	67.88	11.18	-1.689644919	0.01317488	0.03929431	Down	ATEG_09036	hypothetical protein	-	GO:0005634//nucleus	GO:0000981//RNA polymerase II transcription factor activity, sequence-specific DNA binding;GO:0008270//zinc ion binding	GO:0006357//regulation of transcription from RNA polymerase II promoter	gi|115436844|ref|XP_001217658.1|/5.07097e-95/predicted protein [Aspergillus terreus NIH2624]
4318754	1899	580.08	113.68	-1.686022964	1.97E-11	2.25E-10	Down	ATEG_04103	hypothetical protein	-	GO:0016020//membrane	-	-	gi|115391553|ref|XP_001213281.1|/0/predicted protein [Aspergillus terreus NIH2624]
4316168	855	130.53	24.26	-1.685502448	0.0003593	0.00163337	Down	ATEG_01139	hypothetical protein	ko00564//Glycerophospholipid metabolism	-	GO:0016787//hydrolase activity	-	gi|115383914|ref|XP_001208504.1|/0/predicted protein [Aspergillus terreus NIH2624]
4354790	2916	112.69	20.04	-1.681190115	0.00215838	0.0081692	Down	ATEG_00034	hypothetical protein	-	GO:0016021//integral component of membrane	-	-	gi|115490985|ref|XP_001210120.1|/0/predicted protein [Aspergillus terreus NIH2624]

4318013	1608	452.8	89.27	-1.680909513	9.30E-10	8.99E-09	Down	ATEG_03350	similar to ATP binding / protein kinase/ protein serine/threonine kinase	ko04111//Cell cycle - yeast;ko04113//Meiosis - yeast	GO:0000790//nuclear chromatin;GO:0005829//cytosol;GO:0035861//site of double-strand break	GO:0004674//protein serine/threonine kinase activity;GO:0005524//ATP binding	GO:0007095//mitotic G2 DNA damage checkpoint;GO:0018105//peptidyl-serine phosphorylation;GO:0048478//replication fork protection;GO:1902402//signal transduction involved in mitotic DNA damage checkpoint;GO:1990260//negative regulation of transcription from RNA polymerase II promoter by transcription factor localization involved in response to DNA damage checkpoint signaling	gi|115390046|ref|XP_001212528.1|/0/hypothetical protein ATEG_03350 [Aspergillus terreus NIH2624]
4354995	846	621.77	122.6	-1.680677177	1.40E-09	1.33E-08	Down	ATEG_00246	hypothetical protein	ko01100//Metabolic pathways;ko00230//Purine metabolism;ko00240//Pyrimidine metabolism;ko03420//Nucleotide excision repair;ko03030//DNA replication;ko03410//Base excision repair	GO:0005634//nucleus	GO:0046982//protein heterodimerization activity	-	gi|115491409|ref|XP_001210332.1|/0/conserved hypothetical protein [Aspergillus terreus NIH2624]

4319213	1317	152.48	28.79	-1.678175672	0.0001549	0.00075074	Down	ATEG_07027	hypothetical protein	-	GO:0016021//integral component of membrane	GO:0008483//transaminase activity;GO:0030170//pyridoxal phosphate binding	GO:0055085//transmembrane transport	gi|115386344|ref|XP_001209713.1|/0/predicted protein [Aspergillus terreus NIH2624]
4323131	1247	234.87	45.7	-1.674927812	1.29E-05	7.52E-05	Down	ATEG_08607	hypothetical protein	ko00620//Pyruvate metabolism;ko00640//Propanoate metabolism	-	GO:0003854//3-beta-hydroxy-delta5-steroid dehydrogenase activity;GO:0050662//coenzyme binding	GO:0006694//steroid biosynthetic process;GO:0055114//oxidation-reduction process	gi|115402233|ref|XP_001217193.1|/0/predicted protein [Aspergillus terreus NIH2624]
4355737	636	93.55	17.14	-1.673615607	0.00226004	0.00849937	Down	ATEG_00974	hypothetical protein	-	-	-	-	gi|115492865|ref|XP_001211060.1|/3.39334e-156/predicted protein [Aspergillus terreus NIH2624]
4320418	4261	2448.34	484.71	-1.671337505	1.72E-14	2.40E-13	Down	ATEG_04598	similar to na,K-ATPase alpha 2 subunit	ko00230//Purine metabolism	GO:0016021//integral component of membrane	GO:0005388//calcium-transporting ATPase activity;GO:0005524//ATP binding;GO:0046872//metal ion binding	GO:0070588//calcium ion transmembrane transport	gi|115396274|ref|XP_001213776.1|/0/hypothetical protein ATEG_04598 [Aspergillus terreus NIH2624]
4321656	1389	70.61	12.59	-1.669849539	0.00518504	0.01762088	Down	ATEG_06088	Delta(24(24(1)))-sterol reductase	ko01100//Metabolic pathways;ko01130//Biosynthesis of antibiotics;ko00100//Steroid biosynthesis	GO:0016021//integral component of membrane	GO:0016628//oxidoreductase activity, acting on the CH-CH group of donors, NAD or NADP as acceptor	GO:0055114//oxidation-reduction process	gi|115399354|ref|XP_001215266.1|/0/Delta(24(24(1)))-sterol reductase [Aspergillus terreus NIH2624]
4322031	6533	199.61	37.57	-1.668232707	0.00291407	0.01063202	Down	ATEG_06689	hypothetical protein	-	-	-	-	gi|115400557|ref|XP_001215867.1|/0/predicted protein [Aspergillus terreus NIH2624]

4355072	2421	3691.6	687.05	-1.664289248	0.00204073	0.00777748	Down	ATEG_00320	heat shock protein 78, mitochondrial precursor	-	-	GO:0005524//ATP binding;GO:0016787//hydrolase activity	-	gi|115491557|ref|XP_001210406.1|/0/heat shock protein 78, mitochondrial precursor [Aspergillus terreus NIH2624]
4316002	2519	715.8	142.11	-1.660512702	6.79E-11	7.33E-10	Down	ATEG_01888	hypothetical protein	ko03008//Ribosome biogenesis in eukaryotes	-	-	-	gi|115385412|ref|XP_001209253.1|/0/predicted protein [Aspergillus terreus NIH2624]
4316410	852	628.31	127.18	-1.65351493	1.56E-09	1.47E-08	Down	ATEG_01564	hypothetical protein	ko01100//Metabolic pathways;ko01110//Biosynthesis of secondary metabolites;ko00564//Glycerophospholipid metabolism;ko00561//Glycerolipid metabolism	-	-	-	gi|115384764|ref|XP_001208929.1|/0/predicted protein [Aspergillus terreus NIH2624]
4354518	1521	120.48	22.86	-1.645219629	0.00068457	0.0029061	Down	ATEG_09749	hypothetical protein	-	GO:0005634//nucleus;GO:0005829//cytosol	GO:0016788//hydrolase activity, acting on ester bonds	GO:0008152//metabolic process	gi|115443128|ref|XP_001218371.1|/0/conserved hypothetical protein [Aspergillus terreus NIH2624]
4317946	1172	237.64	47.24	-1.640360385	4.49E-06	2.82E-05	Down	ATEG_03542	hypothetical protein	-	-	GO:0016491//oxidoreductase activity	GO:0055114//oxidation-reduction process	gi|115390430|ref|XP_001212720.1|/0/predicted protein [Aspergillus terreus NIH2624]
4315612	1002	263.38	52.78	-1.640358611	1.84E-06	1.23E-05	Down	ATEG_01069	hypothetical protein	ko01100//Metabolic pathways;ko00500//Starch and sucrose metabolism	GO:0016021//integral component of membrane	-	-	gi|115383774|ref|XP_001208434.1|/0/predicted protein [Aspergillus terreus NIH2624]

4355721	1518	142.3	27.33	-1.639452298	0.00037368	0.00169138	Down	ATEG_00958	hypothetical protein	-	GO:0016021//integral component of membrane	GO:0022857//transmembrane transporter activity	GO:0055085//transmembrane transport	gi|115492833|ref|XP_001211044.1|/0/conserved hypothetical protein [Aspergillus terreus NIH2624]
4319475	1191	73.37	12.91	-1.636346465	0.01032541	0.03181718	Down	ATEG_10135	hypothetical protein	ko00254//Aflatoxin biosynthesis	-	GO:0008171//O-methyltransferase activity	GO:0032259//methylation	gi|115385781|ref|XP_001209437.1|/0/conserved hypothetical protein [Aspergillus terreus NIH2624]
4318833	867	586.86	120.22	-1.627777933	1.14E-10	1.21E-09	Down	ATEG_07248	hypothetical protein	ko01100//Metabolic pathways;ko01110//Biosynthesis of secondary metabolites;ko00403//Indole diterpene alkaloid biosynthesis	-	-	-	gi|115386786|ref|XP_001209934.1|/0/predicted protein [Aspergillus terreus NIH2624]
4354057	2112	646.82	132.93	-1.627238771	2.14E-11	2.44E-10	Down	ATEG_09265	hypothetical protein	-	-	GO:0003824//catalytic activity;GO:0050662//coenzyme binding	-	gi|115437740|ref|XP_001217887.1|/2.89736e-154/predicted protein [Aspergillus terreus NIH2624]
4355707	492	284.66	57.76	-1.62674664	1.14E-06	7.75E-06	Down	ATEG_00944	similar to : Mannose-6-phosphate isomerase	-	-	-	-	gi|115492805|ref|XP_001211030.1|/3.87514e-118/hypothetical protein ATEG_00944 [Aspergillus terreus NIH2624]
4353340	1644	344	69.78	-1.624860158	0.00013028	0.00063866	Down	ATEG_08375	hypothetical protein	-	GO:0016021//integral component of membrane	-	GO:0055085//transmembrane transport	gi|115433719|ref|XP_001216996.1|/0/conserved hypothetical protein [Aspergillus terreus NIH2624]

4315721	1080	388.25	79.42	-1.623853448	1.14E-07	8.95E-07	Down	ATEG_01390	hypothetical protein	ko04144//Endocytosis	GO:0016021//integral component of membrane	-	-	gi|115384416|ref|XP_001208755.1|/0/conserved hypothetical protein [Aspergillus terreus NIH2624]
4355710	1611	447.19	92.1	-1.622539309	6.03E-07	4.30E-06	Down	ATEG_00947	hypothetical protein	-	-	GO:0004672//protein kinase activity;GO:0005524//ATP binding;GO:0008080//N-acetyltransferase activity	GO:0006468//protein phosphorylation	gi|115492811|ref|XP_001211033.1|/0/conserved hypothetical protein [Aspergillus terreus NIH2624]
4318365	1506	101.48	19.3	-1.619287709	0.00238058	0.0088992	Down	ATEG_04068	hypothetical protein	ko01100//Metabolic pathways;ko01110//Biosynthesis of secondary metabolites	GO:0016021//integral component of membrane	GO:0004497//monooxygenase activity;GO:0005506//iron ion binding;GO:0016705//oxidoreductase activity, acting on paired donors, with incorporation or reduction of molecular oxygen;GO:0020037//heme binding	GO:0055114//oxidation-reduction process	gi|115391483|ref|XP_001213246.1|/0/predicted protein [Aspergillus terreus NIH2624]
4316043	1605	298.11	60.83	-1.618280727	5.63E-07	4.04E-06	Down	ATEG_01531	hypothetical protein	-	-	GO:0016747//transferase activity, transferring acyl groups other than amino-acyl groups	-	gi|115384698|ref|XP_001208896.1|/0/predicted protein [Aspergillus terreus NIH2624]
4322981	3273	256.85	51.25	-1.614294443	7.48E-05	0.00038506	Down	ATEG_07869	hypothetical protein	-	-	GO:0003824//catalytic activity;GO:0043531//ADP binding	GO:0009116//nucleoside metabolic process	gi|115401804|ref|XP_001216490.1|/0/conserved hypothetical protein [Aspergillus terreus NIH2624]

4353871	1449	330.52	66.72	-1.612198223	3.02E-06	1.97E-05	Down	ATEG_09165	hypothetical protein	-	-	-	-	gi|115437346|ref|XP_001217787.1|/0/conserved hypothetical protein [Aspergillus terreus NIH2624]
4319019	807	1267.25	264.84	-1.610224919	9.17E-15	1.29E-13	Down	ATEG_07272	hypothetical protein	ko04144//Endocytosis	-	GO:0005488	-	gi|115386834|ref|XP_001209958.1|/2.56813e-104/predicted protein [Aspergillus terreus NIH2624]
4319260	687	95.27	18.45	-1.606797047	0.00218949	0.00826872	Down	ATEG_07261	hypothetical protein	ko01100//Metabolic pathways;ko00520//Amino sugar and nucleotide sugar metabolism	GO:0016021//integral component of membrane	-	-	gi|115386812|ref|XP_001209947.1|/9.33565e-176/predicted protein [Aspergillus terreus NIH2624]
4318336	1344	96.45	18.62	-1.60102552	0.00550249	0.01852523	Down	ATEG_03938	hypothetical protein	-	-	-	-	gi|115391223|ref|XP_001213116.1|/0/predicted protein [Aspergillus terreus NIH2624]
4317095	2481	69.5	13.15	-1.599992877	0.00915528	0.02863465	Down	ATEG_02141	hypothetical protein	ko01100//Metabolic pathways;ko00500//Starch and sucrose metabolism	GO:0016021//integral component of membrane	-	-	gi|115387627|ref|XP_001211319.1|/0/conserved hypothetical protein [Aspergillus terreus NIH2624]
4316319	954	221.89	45.53	-1.59800103	1.51E-05	8.76E-05	Down	ATEG_01879	hypothetical protein	ko00620//Pyruvate metabolism	-	-	-	gi|115385394|ref|XP_001209244.1|/0/conserved hypothetical protein [Aspergillus terreus NIH2624]
4319559	1548	85.17	16.07	-1.597317364	0.00507575	0.01729331	Down	ATEG_10014	hypothetical protein	ko01100//Metabolic pathways;ko01220//Degradation of aromatic compounds	-	GO:0016491//oxidoreductase activity;GO:0071949//FAD binding	GO:0055114//oxidation-reduction process	gi|115385539|ref|XP_001209316.1|/0/predicted protein [Aspergillus terreus NIH2624]

4321409	1491	277.41	56.94	-1.592327451	3.11E-06	2.02E-05	Down	ATEG_06203	hypothetical protein	-	-	GO:0008171//O-methyltransferase activity	GO:0032259//methylation	gi|115399594|ref|XP_001215381.1|/0/predicted protein [Aspergillus terreus NIH2624]
4319022	3633	116.57	22.81	-1.592260337	0.00135754	0.00539052	Down	ATEG_07275	hypothetical protein	ko01100//Metabolic pathways;ko00230//Purine metabolism	GO:0016021//integral component of membrane	GO:0022891//substrate-specific transmembrane transporter activity	GO:0055085//transmembrane transport	gi|115386840|ref|XP_001209961.1|/0/conserved hypothetical protein [Aspergillus terreus NIH2624]
4321488	1338	236.4	48.84	-1.591129783	3.89E-05	0.00021222	Down	ATEG_06103	similar to luciferase	ko01100//Metabolic pathways;ko01110//Biosynthesis of secondary metabolites;ko00360//Phenylalanine metabolism;ko00130//Ubiquinone and other terpenoid-quinone biosynthesis	-	GO:0003824//catalytic activity	GO:0008152//metabolic process	gi|115399384|ref|XP_001215281.1|/0/hypothetical protein ATEG_06103 [Aspergillus terreus NIH2624]
4354818	1659	692.64	147.24	-1.586040564	5.18E-11	5.64E-10	Down	ATEG_00062	hypothetical protein	ko01100//Metabolic pathways;ko00564//Glycerophospholipid metabolism;ko00565//Ether lipid metabolism	GO:0016021//integral component of membrane	-	-	gi|115491041|ref|XP_001210148.1|/0/predicted protein [Aspergillus terreus NIH2624]
4319351	2365	127.22	25.53	-1.572954629	0.00087186	0.00360363	Down	ATEG_07234	hypothetical protein	-	-	GO:0004842//ubiquitin-protein transferase activity;GO:0008270//zinc ion binding	GO:0016567//protein ubiquitination	gi|115386758|ref|XP_001209920.1|/0/predicted protein [Aspergillus terreus NIH2624]
4315831	1687	84.6	16.63	-1.569579461	0.00446523	0.01550603	Down	ATEG_01465	hypothetical protein	-	-	-	-	gi|115384566|ref|XP_001208830.1|/0/predicted protein [Aspergillus terreus NIH2624]

4322581	2178	148.58	30.45	-1.567320935	0.00163985	0.0063872	Down	ATEG_07725	hypothetical protein	ko01100//Metabolic pathways;ko00520//Amino sugar and nucleotide sugar metabolism	GO:0016021//integral component of membrane	-	-	gi|115401516|ref|XP_001216346.1|/0/conserved hypothetical protein [Aspergillus terreus NIH2624]
4321190	867	524.51	110.58	-1.567035602	2.18E-08	1.84E-07	Down	ATEG_04954	hypothetical protein	-	-	GO:0016491//oxidoreductase activity	GO:0055114//oxidation-reduction process	gi|115397081|ref|XP_001214132.1|/0/predicted protein [Aspergillus terreus NIH2624]
4316695	1554	821.19	176.93	-1.56545196	3.71E-11	4.09E-10	Down	ATEG_02291	hypothetical protein	-	GO:0016021//integral component of membrane	GO:0004252//serine-type endopeptidase activity	GO:0006508//proteolysis	gi|115387927|ref|XP_001211469.1|/0/conserved hypothetical protein [Aspergillus terreus NIH2624]
4317534	2138	2903.68	627.26	-1.564331947	4.93E-20	1.01E-18	Down	ATEG_03371	similar to NADPH oxidase	ko04145//Phagosome	GO:0016021//integral component of membrane	GO:0016491//oxidoreductase activity	GO:0006801//superoxide metabolic process;GO:0055114//oxidation-reduction process;GO:0070798	gi|115390088|ref|XP_001212549.1|/0/hypothetical protein ATEG_03371 [Aspergillus terreus NIH2624]
4353611	1395	1381.69	300.56	-1.562686558	4.06E-10	4.06E-09	Down	ATEG_09212	acyl-CoA desaturase	ko01212//Fatty acid metabolism;ko01040//Biosynthesis of unsaturated fatty acids	GO:0016021//integral component of membrane	GO:0004768//stearoyl-CoA 9-desaturase activity;GO:0020037//heme binding;GO:0046872//metal ion binding	GO:0006636//unsaturated fatty acid biosynthetic process;GO:0055114//oxidation-reduction process	gi|115437530|ref|XP_001217834.1|/0/acyl-CoA desaturase [Aspergillus terreus NIH2624]
4316395	2152	237.07	49.98	-1.561481203	1.05E-05	6.26E-05	Down	ATEG_01683	similar to transporter	ko01100//Metabolic pathways;ko00230//Purine metabolism;ko00250//Alanine, aspartate and glutamate metabolism	GO:0016021//integral component of membrane	-	GO:0055085//transmembrane transport	gi|115385002|ref|XP_001209048.1|/0/hypothetical protein ATEG_01683 [Aspergillus terreus NIH2624]

4317670	894	205.63	43.32	-1.559611571	4.51E-05	0.00024248	Down	ATEG_02858	hypothetical protein	ko01100//Metabolic pathways;ko00510//N-Glycan biosynthesis;ko00513//Various types of N-glycan biosynthesis	-	-	-	gi|115389062|ref|XP_001212036.1|/0/conserved hypothetical protein [Aspergillus terreus NIH2624]
4354282	2284	562.82	121.38	-1.555985231	9.85E-09	8.59E-08	Down	ATEG_09819	hypothetical protein	ko00330//Arginine and proline metabolism	-	-	-	gi|115443268|ref|XP_001218441.1|/0/conserved hypothetical protein [Aspergillus terreus NIH2624]
4322632	1836	576.11	124.11	-1.555138942	2.01E-10	2.07E-09	Down	ATEG_07548	hypothetical protein	-	GO:0016021//integral component of membrane	-	-	gi|115401162|ref|XP_001216169.1|/0/predicted protein [Aspergillus terreus NIH2624]
4355548	504	481.3	103.47	-1.55491695	6.31E-09	5.62E-08	Down	ATEG_00787	hypothetical protein	-	GO:0016021//integral component of membrane	-	-	gi|115492491|ref|XP_001210873.1|/4.49388e-119/predicted protein [Aspergillus terreus NIH2624]
4320308	1281	281.3	59.83	-1.554683429	2.23E-06	1.48E-05	Down	ATEG_04316	similar to racemase	ko00051//Fructose and mannose metabolism	-	GO:0003824//catalytic activity	GO:0008152//metabolic process	gi|115395214|ref|XP_001213494.1|/0/hypothetical protein ATEG_04316 [Aspergillus terreus NIH2624]
4321967	768	63.93	12.08	-1.546784153	0.0168705	0.04879932	Down	ATEG_06691	hypothetical protein	ko00310//Lysine degradation	-	-	-	gi|115400561|ref|XP_001215869.1|/0/predicted protein [Aspergillus terreus NIH2624]
4320518	2202	2011.9	442.39	-1.541573879	3.10E-22	7.22E-21	Down	ATEG_04400	hypothetical protein	-	-	-	-	gi|115395878|ref|XP_001213578.1|/0/predicted protein [Aspergillus terreus NIH2624]

4320668	1629	1538.12	339.83	-1.54136808	1.27E-12	1.60E-11	Down	ATEG_05130	hypothetical protein	ko01100//Metabolic pathways;ko00500//Starch and sucrose metabolism	GO:0016021//integral component of membrane	GO:0016740//transferase activity	GO:0006633//fatty acid biosynthetic process	gi|115397433|ref|XP_001214308.1|/0/conserved hypothetical protein [Aspergillus terreus NIH2624]
4316974	4035	72.88	14.49	-1.539071655	0.01076001	0.03302622	Down	ATEG_02092	hypothetical protein	-	GO:0005719//nuclear euchromatin;GO:0005829//cytosol;GO:0031618//nuclear pericentric heterochromatin;GO:0034399//nuclear periphery;GO:0034507//chromosome, centromeric outer repeat region	GO:0003690//double-stranded DNA binding;GO:0003725//double-stranded RNA binding;GO:0004386//helicase activity;GO:0004525//ribonuclease III activity;GO:0005524//ATP binding;GO:0008270//zinc ion binding;GO:1990188//euchromatin binding	GO:0030422//production of siRNA involved in RNA interference;GO:0033562//co-transcriptional gene silencing by RNA interference machinery;GO:0034613//cellular protein localization;GO:0035389//establishment of chromatin silencing at silent mating-type cassette;GO:0050688//regulation of defense response to virus;GO:0051570//regulation of histone H3-K9 methylation;GO:0070868//heterochromatin organization involved in chromatin silencing;GO:0090052//regulation of chromatin silencing at centromere;GO:0090502//RNA phosphodiester bond hydrolysis, endonucleolytic;G	gi|115387529|ref|XP_001211270.1|/0/conserved hypothetical protein [Aspergillus terreus NIH2624]

4316285	582	133.91	27.81	-1.536477285	0.00081168	0.0033774	Down	ATEG_01773	hypothetical protein	ko01100//Metabolic pathways;ko00520//Amino sugar and nucleotide sugar metabolism	GO:0016021//integral component of membrane	-	-	gi|115385182|ref|XP_001209138.1|/1.9873e-137/conserved hypothetical protein [Aspergillus terreus NIH2624]
4353523	942	5023.11	1116.26	-1.533062159	5.61E-25	1.51E-23	Down	ATEG_07984	hypothetical protein	ko04144//Endocytosis	-	-	-	gi|115432936|ref|XP_001216605.1|/0/predicted protein [Aspergillus terreus NIH2624]
4321724	1188	124.38	25.98	-1.531083721	0.00435023	0.01515714	Down	ATEG_06081	hypothetical protein	-	-	GO:0008199//ferric iron binding;GO:0016702//oxidoreductase activity, acting on single donors with incorporation of molecular oxygen, incorporation of two atoms of oxygen	GO:0006725//cellular aromatic compound metabolic process;GO:0055114//oxidation-reduction process	gi|115399340|ref|XP_001215259.1|/0/conserved hypothetical protein [Aspergillus terreus NIH2624]
4317682	1647	106.5	22.07	-1.528971426	0.00231698	0.00868568	Down	ATEG_03449	hypothetical protein	-	GO:0005634//nucleus	GO:0000981//RNA polymerase II transcription factor activity, sequence-specific DNA binding;GO:0003677//DNA binding;GO:0008270//zinc ion binding	GO:0006357//regulation of transcription from RNA polymerase II promoter	gi|115390244|ref|XP_001212627.1|/0/conserved hypothetical protein [Aspergillus terreus NIH2624]

4320789	1560	5185.33	1144.47	-1.528520668	3.29E-18	5.84E-17	Down	ATEG_05609	protein disulfide-isomerase precursor	ko04141//Protein processing in endoplasmic reticulum	GO:0005783//endoplasmic reticulum	GO:0003756//protein disulfide isomerase activity;GO:0009055//electron carrier activity;GO:0015035//protein disulfide oxidoreductase activity	GO:0006457//protein folding;GO:0006662//glycerol ether metabolic process;GO:0045454//cell redox homeostasis	gi|115398391|ref|XP_001214787.1|/0/protein disulfide-isomerase precursor [Aspergillus terreus NIH2624]
4322598	2948	975.42	216.07	-1.525367788	3.96E-11	4.36E-10	Down	ATEG_07654	hypothetical protein	ko04111//Cell cycle - yeast	-	GO:0004497//monooxygenase activity;GO:0005506//iron ion binding;GO:0016705//oxidoreductase activity, acting on paired donors, with incorporation or reduction of molecular oxygen;GO:0020037//heme binding	GO:0055114//oxidation-reduction process	gi|115401374|ref|XP_001216275.1|/0/predicted protein [Aspergillus terreus NIH2624]
4318219	1434	773.28	171.87	-1.522712428	1.45E-11	1.66E-10	Down	ATEG_03863	hypothetical protein	ko00562//Inositol phosphate metabolism	-	GO:0008081//phosphoric diester hydrolase activity	GO:0006629//lipid metabolic process	gi|115391073|ref|XP_001213041.1|/0/conserved hypothetical protein [Aspergillus terreus NIH2624]

4321381	1380	285.76	61.9	-1.520199738	5.84E-06	3.61E-05	Down	ATEG_06162	hypothetical protein	-	GO:0001401//mitochondrial sorting and assembly machinery complex;GO:0031307//integral component of mitochondrial outer membrane;GO:0032865//ERMES complex	-	GO:0000002//mitochondrial genome maintenance;GO:0015914//phospholipid transport;GO:0045040//protein import into mitochondrial outer membrane;GO:0048311//mitochondrion distribution;GO:0051654//establishment of mitochondrion localization;GO:0070096//mitochondrial outer membrane translocase complex assembly;GO:0070584//mitochondrion morphogenesis;GO:1990456//mitochondrion-ER tethering	gi|115399502|ref|XP_001215340.1|/0/conserved hypothetical protein [Aspergillus terreus NIH2624]
4321932	4698	230.38	50.08	-1.519432138	3.44E-05	0.00018912	Down	ATEG_06502	hypothetical protein	ko03018//RNA degradation	GO:0016021//integral component of membrane	GO:0005215//transporter activity;GO:0005524//ATP binding;GO:0016887//ATPase activity	GO:0006810//transport	gi|115400183|ref|XP_001215680.1|/0/conserved hypothetical protein [Aspergillus terreus NIH2624]
4316041	1509	425.43	93.62	-1.519127053	6.71E-08	5.41E-07	Down	ATEG_01529	hypothetical protein	ko01100//Metabolic pathways;ko00500//Starch and sucrose metabolism	GO:0005794//Golgi apparatus;GO:0016021//integral component of membrane	-	-	gi|115384694|ref|XP_001208894.1|/0/predicted protein [Aspergillus terreus NIH2624]

4320405	2106	470.99	103.59	-1.513996746	9.88E-07	6.78E-06	Down	ATEG_04867	hypothetical protein	-	GO:0005634//nucleus;GO:0044732//mitotic spindle pole body	GO:0003688//DNA replication origin binding	-	gi|115396812|ref|XP_001214045.1|/0/conserved hypothetical protein [Aspergillus terreus NIH2624]
4316528	429	483.54	105.58	-1.511465994	4.22E-07	3.10E-06	Down	ATEG_01971	hypothetical protein	-	-	-	-	gi|115387287|ref|XP_001211149.1|/2.64799e-99/predicted protein [Aspergillus terreus NIH2624]
4323536	2967	838.46	184.7	-1.505777102	5.88E-09	5.25E-08	Down	ATEG_08513	hypothetical protein	-	-	-	-	gi|115402045|ref|XP_001217099.1|/0/conserved hypothetical protein [Aspergillus terreus NIH2624]
4321997	1242	1223.02	273.23	-1.503137404	3.90E-12	4.71E-11	Down	ATEG_06321	hypothetical protein	-	GO:0005737//cytoplasm	GO:0004190//aspartic-type endopeptidase activity;GO:0043130//ubiquitin binding	GO:0006508//proteolysis;GO:0015031//protein transport	gi|115399820|ref|XP_001215499.1|/0/conserved hypothetical protein [Aspergillus terreus NIH2624]
4354617	1239	802.38	181.01	-1.502826138	4.94E-11	5.39E-10	Down	ATEG_10370	hypothetical protein	-	GO:0016021//integral component of membrane	-	-	gi|115449871|ref|XP_001218718.1|/0/predicted protein [Aspergillus terreus NIH2624]
4322580	1880	102.56	21.57	-1.502337692	0.00291763	0.01063975	Down	ATEG_07724	hypothetical protein	ko01100//Metabolic pathways;ko00230//Purine metabolism;ko00250//Alanine, aspartate and glutamate metabolism	GO:0016021//integral component of membrane	-	GO:0055085//transmembrane transport	gi|115401514|ref|XP_001216345.1|/0/predicted protein [Aspergillus terreus NIH2624]

4319095	744	130.01	28.03	-1.501734808	0.00118908	0.00477619	Down	ATEG_06953	hypothetical protein	-	GO:0005886//plasma membrane;GO:0016021//integral component of membrane	-	-	gi|115386196|ref|XP_001209639.1|/0/conserved hypothetical protein [Aspergillus terreus NIH2624]
4323063	1457	1229	273.94	-1.500642296	4.35E-10	4.32E-09	Down	ATEG_08864	DNA-repair protein rad2	ko03030//DNA replication;ko03410//Base excision repair;ko03450//Non-homologous end-joining	GO:0005654//nucleoplasm;GO:0005730//nucleolus;GO:0005739//mitochondrion;GO:0035861//site of double-strand break	GO:0000287//magnesium ion binding;GO:0003677//DNA binding;GO:0017108//5'-flap endonuclease activity;GO:0045145//single-stranded DNA 5'-3' exodeoxyribonuclease activity;GO:0051908//double-stranded DNA 5'-3' exodeoxyribonuclease activity;GO:1904162//5'-3' exodeoxyribonuclease activity involved in UV-damage excision repair	GO:0006284//base-excision repair;GO:0070914//UV-damage excision repair;GO:0090305//nucleic acid phosphodiester bond hydrolysis;GO:1903469//removal of RNA primer involved in mitotic DNA replication	gi|115402747|ref|XP_001217450.1|/0/DNA-repair protein rad2 [Aspergillus terreus NIH2624]
4316647	375	320.96	70.41	-1.499448607	1.57E-05	9.05E-05	Down	ATEG_01996	hypothetical protein	ko03440//Homologous recombination;ko03420//Nucleotide excision repair;ko03030//DNA replication;ko03430//Mismatch repair	GO:0005634//nucleus	GO:0003677//DNA binding	GO:0006260//DNA replication;GO:0006281//DNA repair;GO:0006310//DNA recombination	gi|115387337|ref|XP_001211174.1|/2.21874e-87/conserved hypothetical protein [Aspergillus terreus NIH2624]

4320714	612	122.17	26.04	-1.498089453	0.00141612	0.00558531	Down	ATEG_05103	hypothetical protein	-	-	-	-	gi|115397379|ref|XP_001214281.1|/3.99797e-151/conserved hypothetical protein [Aspergillus terreus NIH2624]
4322716	1841	2688.84	608.53	-1.494909023	5.48E-19	1.03E-17	Down	ATEG_07886	hypothetical protein	-	GO:0016021//integral component of membrane	GO:0005215//transporter activity	GO:0055085//transmembrane transport	gi|115401838|ref|XP_001216507.1|/0/conserved hypothetical protein [Aspergillus terreus NIH2624]
4320835	5372	671.1	150.57	-1.494027917	9.91E-09	8.63E-08	Down	ATEG_05265	calcium-transporting ATPase 3	-	GO:0016021//integral component of membrane	GO:0005524//ATP binding;GO:0019829//cation-transporting ATPase activity;GO:0046872//metal ion binding	GO:0098655//cation transmembrane transport	gi|115397703|ref|XP_001214443.1|/0/calcium-transporting ATPase 3 [Aspergillus terreus NIH2624]
4354560	1500	307.75	68.86	-1.492720046	2.85E-05	0.00015858	Down	ATEG_09845	hypothetical protein	ko01100//Metabolic pathways;ko01110//Biosynthesis of secondary metabolites;ko01040//Biosynthesis of unsaturated fatty acids;ko00062//Fatty acid elongation	-	GO:0003676//nucleic acid binding;GO:0008270//zinc ion binding;GO:0047617//acyl-CoA hydrolase activity	GO:0006637//acyl-CoA metabolic process	gi|115443320|ref|XP_001218467.1|/0/predicted protein [Aspergillus terreus NIH2624]
4318791	4459	141.73	29.89	-1.491621457	0.00206009	0.0078232	Down	ATEG_04129	hypothetical protein	-	-	-	-	gi|115391605|ref|XP_001213307.1|/0/predicted protein [Aspergillus terreus NIH2624]

4354074	1231	84.04	17.7	-1.49059193	0.00898893	0.02826566	Down	ATEG_09288	hypothetical protein	-	-	-	-	gi|115437830|ref|XP_001217910.1|/3.01721e-154/predicted protein [Aspergillus terreus NIH2624]
4318622	894	525.62	119.05	-1.489165239	5.84E-09	5.23E-08	Down	ATEG_03750	hypothetical protein	-	-	-	-	gi|115390847|ref|XP_001212928.1|/0/conserved hypothetical protein [Aspergillus terreus NIH2624]
4318924	2658	935.39	213.44	-1.488462168	4.02E-06	2.55E-05	Down	ATEG_06909	similar to ATP-dependent metalloprotease	-	GO:0005745//m-AAA complex;GO:0097002//mitochondrial inner boundary membrane	GO:0004222//metalloendopeptidase activity;GO:0005524//ATP binding;GO:0008270//zinc ion binding;GO:0016887//ATPase activity	GO:0001302//replicative cell aging;GO:0002181//cytoplasmic translation;GO:0006461//protein complex assembly;GO:0006465//signal peptide processing	gi|115386108|ref|XP_001209595.1|/0/hypothetical protein ATEG_06909 [Aspergillus terreus NIH2624]
4317823	1686	270.66	59.52	-1.488048944	2.52E-05	0.00014135	Down	ATEG_03431	hypothetical protein	ko01220//Degradation of aromatic compounds	-	GO:0004499//N,N-dimethylaniline monooxygenase activity;GO:0050660//flavin adenine dinucleotide binding;GO:0050661//NADP binding	GO:0045461;GO:0055114//oxidation-reduction process	gi|115390208|ref|XP_001212609.1|/0/conserved hypothetical protein [Aspergillus terreus NIH2624]
4355246	979	88.02	18.43	-1.486147418	0.00680987	0.02227154	Down	ATEG_00492	hypothetical protein	-	-	-	-	gi|115491901|ref|XP_001210578.1|/5.20001e-177/conserved hypothetical protein [Aspergillus terreus NIH2624]

4317087	2172	7275.95	1668.76	-1.485074676	2.16E-33	8.31E-32	Down	ATEG_02144	hypothetical protein	-	GO:0016021//integral component of membrane	GO:0015171//amino acid transmembrane transporter activity	GO:0003333//amino acid transmembrane transport	gi|115387633|ref|XP_001211322.1|/0/conserved hypothetical protein [Aspergillus terreus NIH2624]
4321336	914	165.32	36.44	-1.48015841	0.00029453	0.00136337	Down	ATEG_06223	hypothetical protein	-	-	-	-	gi|115399674|ref|XP_001215401.1|/1.00164e-180/predicted protein [Aspergillus terreus NIH2624]
4354819	6376	316.63	71.86	-1.476645211	4.59E-06	2.88E-05	Down	ATEG_00063	similar to ABC1 transport protein	ko02010//ABC transporters	GO:0016021//integral component of membrane	GO:0005524//ATP binding;GO:0042626//ATPase activity, coupled to transmembrane movement of substances	GO:0055085//transmembrane transport	gi|115491043|ref|XP_001210149.1|/0/hypothetical protein ATEG_00063 [Aspergillus terreus NIH2624]
4317259	999	428.69	98.15	-1.47253908	2.18E-05	0.00012382	Down	ATEG_02660	hypothetical protein	ko01100//Metabolic pathways;ko01200//Carbon metabolism;ko00280//Valine, leucine and isoleucine degradation;ko00640//Propanoate metabolism;ko00562//Inositol phosphate metabolism;ko00410//beta-Alanine metabolism	-	-	-	gi|115388665|ref|XP_001211838.1|/0/conserved hypothetical protein [Aspergillus terreus NIH2624]
4316872	3289	87.39	18.67	-1.469380338	0.01276341	0.03823726	Down	ATEG_02376	hypothetical protein	-	-	-	-	gi|115388097|ref|XP_001211554.1|/9.01541e-143/predicted protein [Aspergillus terreus NIH2624]

4322923	1404	712.32	163.86	-1.468212359	5.10E-11	5.57E-10	Down	ATEG_07831	similar to subunit of a Golgi mannosyltransferase complex also containing Anp1p	ko01100//Metabolic pathways;ko00513//Various types of N-glycan biosynthesis	GO:0000136//alpha-1,6-mannosyltransferase complex	GO:0000009//alpha-1,6-mannosyltransferase activity	GO:0000032//cell wall mannoprotein biosynthetic process;GO:0000917//barrier septum assembly;GO:0006487//protein N-linked glycosylation;GO:0007114//cell budding;GO:0097502//mannosylation	gi|115401728|ref|XP_001216452.1|/0/hypothetical protein ATEG_07831 [Aspergillus terreus NIH2624]
4319911	1866	192.83	43.27	-1.465403638	0.00017717	0.00085293	Down	ATEG_04489	hypothetical protein	-	-	-	-	gi|115396056|ref|XP_001213667.1|/0/predicted protein [Aspergillus terreus NIH2624]
4353124	1602	700.85	160.09	-1.465249587	6.94E-08	5.58E-07	Down	ATEG_08303	hypothetical protein	ko00380//Tryptophan metabolism;ko00460//Cyanoamino acid metabolism;ko00910//Nitrogen metabolism	GO:0005576//extracellular region	GO:0008762//UDP-N-acetylmuramate dehydrogenase activity;GO:0050660//flavin adenine dinucleotide binding	GO:0055114//oxidation-reduction process	gi|115433574|ref|XP_001216924.1|/0/conserved hypothetical protein [Aspergillus terreus NIH2624]
4318343	1790	2584.25	597.35	-1.461569201	6.61E-18	1.14E-16	Down	ATEG_03933	zinc finger protein 160	-	-	GO:0003676//nucleic acid binding;GO:0046872//metal ion binding	-	gi|115391213|ref|XP_001213111.1|/0/zinc finger protein 160 [Aspergillus terreus NIH2624]
4319306	750	218.55	49.38	-1.454347533	6.92E-05	0.00035921	Down	ATEG_06981	hypothetical protein	ko04141//Protein processing in endoplasmic reticulum	GO:0005789//endoplasmic reticulum membrane;GO:0016021//integral component of membrane	-	GO:0044257//cellular protein catabolic process	gi|115386252|ref|XP_001209667.1|/0/conserved hypothetical protein [Aspergillus terreus NIH2624]

4318590	1967	138.95	31.16	-1.451866296	0.00122121	0.00489686	Down	ATEG_04167	hypothetical protein	-	GO:0005634//nucleus	GO:0000981//RNA polymerase II transcription factor activity, sequence-specific DNA binding;GO:0003677//DNA binding;GO:0008270//zinc ion binding	GO:0006357//regulation of transcription from RNA polymerase II promoter	gi|115391681|ref|XP_001213345.1|/0/predicted protein [Aspergillus terreus NIH2624]
4316812	456	326.54	74.69	-1.450154815	2.54E-05	0.00014217	Down	ATEG_02643	hypothetical protein	-	-	-	-	gi|115388631|ref|XP_001211821.1|/6.60835e-111/conserved hypothetical protein [Aspergillus terreus NIH2624]
4353959	2366	159.66	36.05	-1.44627043	0.00067102	0.00285801	Down	ATEG_09326	hypothetical protein	ko01100//Metabolic pathways;ko00051//Fructose and mannose metabolism;ko00650//Butanoate metabolism;ko00591//Linoleic acid metabolism	-	-	-	gi|115437978|ref|XP_001217948.1|/0/conserved hypothetical protein [Aspergillus terreus NIH2624]
4315785	2016	293.65	68.13	-1.443708848	0.00012931	0.00063482	Down	ATEG_01723	hypothetical protein	-	GO:0005634//nucleus	GO:0000981//RNA polymerase II transcription factor activity, sequence-specific DNA binding;GO:0008270//zinc ion binding	GO:0006357//regulation of transcription from RNA polymerase II promoter	gi|115385082|ref|XP_001209088.1|/0/conserved hypothetical protein [Aspergillus terreus NIH2624]
4319013	1785	332.38	77.25	-1.442762866	5.89E-06	3.63E-05	Down	ATEG_07277	hypothetical protein	-	GO:0005634//nucleus	GO:0003677//DNA binding;GO:0008270//zinc ion binding	GO:0006351//transcription, DNA-templated	gi|115386844|ref|XP_001209963.1|/0/predicted protein [Aspergillus terreus NIH2624]

4322596	1362	613.63	143.7	-1.442687022	9.36E-10	9.03E-09	Down	ATEG_07652	hypothetical protein	ko01100//Metabolic pathways;ko01220//Degradation of aromatic compounds	-	GO:0004497//monooxygenase activity;GO:0071949//FAD binding	GO:0055114//oxidation-reduction process	gi|115401370|ref|XP_001216273.1|/0/predicted protein [Aspergillus terreus NIH2624]
4319642	1629	170.91	38.61	-1.442592881	0.00037018	0.00167718	Down	ATEG_09967	similar to efflux pump	-	GO:0016021//integral component of membrane	-	GO:0055085//transmembrane transport	gi|115385445|ref|XP_001209269.1|/0/hypothetical protein ATEG_09967 [Aspergillus terreus NIH2624]
4319945	630	421.83	97.14	-1.434302376	5.82E-06	3.60E-05	Down	ATEG_04769	hypothetical protein	ko03060//Protein export	GO:0005787//signal peptidase complex;GO:0016021//integral component of membrane	GO:0008233//peptidase activity	GO:0006465//signal peptide processing;GO:0045047//protein targeting to ER	gi|115396616|ref|XP_001213947.1|/1.2806e-150/conserved hypothetical protein [Aspergillus terreus NIH2624]
4317821	1071	186.01	42.09	-1.433219596	0.00044537	0.00198146	Down	ATEG_03429	hypothetical protein	-	-	-	-	gi|115390204|ref|XP_001212607.1|/0/predicted protein [Aspergillus terreus NIH2624]
4321326	1222	270.6	62.39	-1.432480904	3.04E-05	0.00016824	Down	ATEG_06138	hypothetical protein	-	-	-	-	gi|115399454|ref|XP_001215316.1|/1.11166e-106/predicted protein [Aspergillus terreus NIH2624]
4320071	936	85.2	18.29	-1.425409532	0.01221811	0.03684237	Down	ATEG_04372	hypothetical protein	ko01100//Metabolic pathways;ko00520//Amino sugar and nucleotide sugar metabolism	-	-	-	gi|115395822|ref|XP_001213550.1|/0/conserved hypothetical protein [Aspergillus terreus NIH2624]
4355352	5043	3546.83	847.43	-1.423285297	9.53E-25	2.53E-23	Down	ATEG_00598	hypothetical protein	ko01100//Metabolic pathways;ko00520//Amino sugar and nucleotide sugar metabolism	GO:0016021//integral component of membrane	-	GO:0055085//transmembrane transport	gi|115492113|ref|XP_001210684.1|/0/conserved hypothetical protein [Aspergillus terreus NIH2624]

4316792	4023	419.22	99.74	-1.415898405	4.94E-07	3.58E-06	Down	ATEG_02408	hypothetical protein	ko01100//Metabolic pathways;ko00500//Starch and sucrose metabolism	-	GO:0003824//catalytic activity;GO:0030246//carbohydrate binding	GO:0005975//carbohydrate metabolic process	gi|115388161|ref|XP_001211586.1|/0/predicted protein [Aspergillus terreus NIH2624]
4354881	954	853.62	202.55	-1.412687053	6.52E-08	5.26E-07	Down	ATEG_00124	hypothetical protein	ko01100//Metabolic pathways;ko00040//Pentose and glucuronate interconversions	-	GO:0016829//lyase activity	GO:0008152//metabolic process	gi|115491165|ref|XP_001210210.1|/0/conserved hypothetical protein [Aspergillus terreus NIH2624]
4317752	977	174.27	39.18	-1.409337507	0.00210938	0.00799366	Down	ATEG_03489	hypothetical protein	ko01100//Metabolic pathways;ko00051//Fructose and mannose metabolism;ko00650//Butanoate metabolism;ko00591//Linoleic acid metabolism	-	GO:0016491//oxidoreductase activity	GO:0055114//oxidation-reduction process	gi|115390324|ref|XP_001212667.1|/0/conserved hypothetical protein [Aspergillus terreus NIH2624]
4322875	1185	105.38	24.02	-1.40877963	0.0063871	0.02110217	Down	ATEG_07739	hypothetical protein	ko03022//Basal transcription factors	GO:0005674//transcription factor TFIIF complex;GO:0044732//mitotic spindle pole body	GO:0000991//transcription factor activity, core RNA polymerase II binding;GO:0003743//translation initiation factor activity;GO:0003824//catalytic activity	GO:0001174//transcriptional start site selection at RNA polymerase II promoter;GO:0006357//regulation of transcription from RNA polymerase II promoter;GO:0006368//transcription elongation from RNA polymerase II promoter;GO:0006413//translational initiation	gi|115401544|ref|XP_001216360.1|/0/conserved hypothetical protein [Aspergillus terreus NIH2624]

4319314	2112	1770.46	427.39	-1.408589673	2.51E-17	4.21E-16	Down	ATEG_06903	hypothetical protein	ko04011//MAPK signaling pathway - yeast	GO:0005634//nucleus;GO:0005829//cytosol	GO:0017017//MAP kinase tyrosine/serine/threonine phosphatase activity;GO:0033550//MAP kinase tyrosine phosphatase activity	GO:0000188//inactivation of MAPK activity;GO:0022604//regulation of cell morphogenesis;GO:0030644//cellular chloride ion homeostasis;GO:0071472//cellular response to salt stress;GO:0090028//positive regulation of pheromone-dependent signal transduction involved in conjugation with cellular fusion;GO:1902412//regulation of mitotic cytokinesis;GO:1990264//peptidyl-tyrosine dephosphorylation involved in inactivation of protein kinase activity	gi|115386096|ref|XP_001209589.1|/0/conserved hypothetical protein [Aspergillus terreus NIH2624]
4354867	5842	4674.59	1126.44	-1.408155618	6.18E-24	1.55E-22	Down	ATEG_00110	hypothetical protein	ko04141//Protein processing in endoplasmic reticulum;ko04120//Ubiquitin mediated proteolysis	GO:0000836//Hrd1p ubiquitin ligase complex;GO:0016021//integral component of membrane	GO:0008270//zinc ion binding;GO:0061630//ubiquitin protein ligase activity	GO:0016567//protein ubiquitination;GO:0030433//ubiquitin-dependent ERAD pathway	gi|115491137|ref|XP_001210196.1|/0/conserved hypothetical protein [Aspergillus terreus NIH2624]
4354017	4070	328.96	78.21	-1.40224074	3.66E-06	2.35E-05	Down	ATEG_09232	hypothetical protein	-	-	-	-	gi|115437610|ref|XP_001217854.1|/1.27039e-175/predicted protein [Aspergillus terreus NIH2624]

4320404	2133	423.78	101.19	-1.400489575	9.35E-07	6.45E-06	Down	ATEG_04866	hypothetical protein	-	-	-	-	gi|115396810|ref|XP_001214044.1|/0/conserved hypothetical protein [Aspergillus terreus NIH2624]
4318713	3847	283.63	67.39	-1.394662784	2.52E-05	0.00014158	Down	ATEG_03798	hypothetical protein	-	-	GO:0003824//catalytic activity	GO:0009116//nucleoside metabolic process	gi|115390943|ref|XP_001212976.1|/0/predicted protein [Aspergillus terreus NIH2624]
4320186	1638	126.08	29.25	-1.39403786	0.00219595	0.00828491	Down	ATEG_04719	hypothetical protein	ko00563//Glycosylphosphatidylinositol(GPI)-anchor biosynthesis	-	-	-	gi|115396516|ref|XP_001213897.1|/0/predicted protein [Aspergillus terreus NIH2624]
4355706	990	239.89	57.13	-1.390521112	6.39E-05	0.00033344	Down	ATEG_00943	hypothetical protein	ko00254//Aflatoxin biosynthesis	-	-	-	gi|115492803|ref|XP_001211029.1|/0/conserved hypothetical protein [Aspergillus terreus NIH2624]
4320505	4425	537.52	129.25	-1.390339274	1.01E-07	7.99E-07	Down	ATEG_04818	hypothetical protein	-	GO:0030288//outer membrane-bounded periplasmic space	-	GO:0006810//transport	gi|115396714|ref|XP_001213996.1|/0/predicted protein [Aspergillus terreus NIH2624]
4353634	669	366.95	88.08	-1.388770983	4.96E-06	3.10E-05	Down	ATEG_09089	hypothetical protein	ko01100//Metabolic pathways;ko01110//Biosynthesis of secondary metabolites;ko00500//Starch and sucrose metabolism;ko00460//Cyanoamino acid metabolism	-	-	-	gi|115437042|ref|XP_001217711.1|/1.49634e-169/predicted protein [Aspergillus terreus NIH2624]

4321797	1542	3079.35	759.66	-1.388437548	3.56E-11	3.94E-10	Down	ATEG_05917	cytochrome P450 51	ko01100//Metabolic pathways;ko01110//Biosynthesis of secondary metabolites;ko01130//Biosynthesis of antibiotics;ko00100//Steroid biosynthesis	GO:0016021//integral component of membrane	GO:0004497//monooxygenase activity;GO:0005506//iron ion binding;GO:0016705//oxidoreductase activity, acting on paired donors, with incorporation or reduction of molecular oxygen;GO:0020037//heme binding	GO:0055114//oxidation-reduction process	gi|115399012|ref|XP_001215095.1|/0/cytochrome P450 51 [Aspergillus terreus NIH2624]
4355147	1854	887.14	215.51	-1.385622046	6.17E-11	6.69E-10	Down	ATEG_00393	hypothetical protein	ko04141//Protein processing in endoplasmic reticulum	-	GO:0019901//protein kinase binding	GO:0000079//regulation of cyclin-dependent protein serine/threonine kinase activity	gi|115491703|ref|XP_001210479.1|/0/conserved hypothetical protein [Aspergillus terreus NIH2624]
4322502	1947	378.72	91.24	-1.385090835	3.62E-06	2.32E-05	Down	ATEG_07932	hypothetical protein	ko01100//Metabolic pathways;ko00562//Inositol phosphate metabolism;ko04070//Phosphatidylinositol signaling system	GO:0016021//integral component of membrane	GO:0015171//amino acid transmembrane transporter activity	GO:0003333//amino acid transmembrane transport	gi|115401930|ref|XP_001216553.1|/0/predicted protein [Aspergillus terreus NIH2624]
4355725	1383	391.73	95.08	-1.383947965	8.53E-07	5.93E-06	Down	ATEG_00962	hypothetical protein	-	GO:0005634//nucleus	GO:0000981//RNA polymerase II transcription factor activity, sequence-specific DNA binding;GO:0008270//zinc ion binding	GO:0006357//regulation of transcription from RNA polymerase II promoter	gi|115492841|ref|XP_001211048.1|/0/predicted protein [Aspergillus terreus NIH2624]
4355054	1137	224.85	52.88	-1.381265458	0.00061403	0.00264409	Down	ATEG_00303	hypothetical protein	-	GO:0016021//integral component of membrane	-	GO:0055085//transmembrane transport	gi|115491523|ref|XP_001210389.1|/0/predicted protein [Aspergillus terreus NIH2624]

4317375	3968	339.73	81.86	-1.380225858	1.52E-05	8.80E-05	Down	ATEG_03341	hypothetical protein	-	GO:0016021//integral component of membrane	-	-	gi|115390028|ref|XP_001212519.1|/0/predicted protein [Aspergillus terreus NIH2624]
4319430	891	768.91	187.77	-1.378241875	3.54E-10	3.56E-09	Down	ATEG_10217	hypothetical protein	-	-	GO:0016491//oxidoreductase activity	GO:0055114//oxidation-reduction process	gi|115385945|ref|XP_001209519.1|/0/conserved hypothetical protein [Aspergillus terreus NIH2624]
4316567	760	1700.99	422.78	-1.374596219	7.73E-09	6.83E-08	Down	ATEG_02487	hypothetical protein	-	-	-	-	gi|115388319|ref|XP_001211665.1|/7.39436e-126/predicted protein [Aspergillus terreus NIH2624]
4354599	4032	585.73	144.37	-1.374330536	6.04E-07	4.30E-06	Down	ATEG_10358	hypothetical protein	-	GO:0005634//nucleus;GO:0016020//membrane	GO:0000166//nucleotide binding;GO:0000981//RNA polymerase II transcription factor activity, sequence-specific DNA binding;GO:0003677//DNA binding;GO:0008270//zinc ion binding;GO:0016887//ATPase activity	GO:0006357//regulation of transcription from RNA polymerase II promoter;GO:0006810//transport	gi|115449827|ref|XP_001218706.1|/0/conserved hypothetical protein [Aspergillus terreus NIH2624]
4319408	2655	1956.02	485.95	-1.371513748	3.78E-15	5.47E-14	Down	ATEG_10105	hypothetical protein	-	-	-	-	gi|115385721|ref|XP_001209407.1|/0/conserved hypothetical protein [Aspergillus terreus NIH2624]

4323160	1419	435.39	107.41	-1.369613949	1.19E-05	6.98E-05	Down	ATEG_08896	hypothetical protein	-	-	GO:0004252//serine-type endopeptidase activity	GO:0006508//proteolysis	gi|115402811|ref|XP_001217482.1|/0/conserved hypothetical protein [Aspergillus terreus NIH2624]
4316201	1960	124.41	28.5	-1.365731303	0.00686904	0.02244156	Down	ATEG_01376	hypothetical protein	-	GO:0016021//integral component of membrane	-	-	gi|115384388|ref|XP_001208741.1|/0/predicted protein [Aspergillus terreus NIH2624]
4315548	1026	278.52	68	-1.365056875	2.29E-05	0.00012964	Down	ATEG_01822	hypothetical protein	-	-	GO:0008236//serine-type peptidase activity	GO:0006508//proteolysis	gi|115385280|ref|XP_001209187.1|/0/conserved hypothetical protein [Aspergillus terreus NIH2624]
4354799	3690	14645.11	3636.23	-1.3626481	7.03E-24	1.75E-22	Down	ATEG_00043	hypothetical protein	ko03013//RNA transport	-	-	-	gi|115491003|ref|XP_001210129.1|/0/conserved hypothetical protein [Aspergillus terreus NIH2624]
4319020	1770	1513.17	374.32	-1.362395239	1.48E-13	1.94E-12	Down	ATEG_07273	hypothetical protein	ko01100//Metabolic pathways;ko00500//Starch and sucrose metabolism	-	GO:0004499//N,N-dimethylaniline monooxygenase activity;GO:0050660//flavin adenine dinucleotide binding;GO:0050661//NADP binding	GO:0055114//oxidation-reduction process	gi|115386836|ref|XP_001209959.1|/0/predicted protein [Aspergillus terreus NIH2624]
4318739	1332	412.46	101.86	-1.361266017	7.43E-07	5.23E-06	Down	ATEG_03841	similar to : Uncharacterized conserved protein	ko01100//Metabolic pathways;ko00280//Valine, leucine and isoleucine degradation	-	GO:0051920//peroxiredoxin activity	GO:0055114//oxidation-reduction process;GO:0098869//cellular oxidant detoxification	gi|115391029|ref|XP_001213019.1|/1.90367e-142/hypothetical protein ATEG_03841 [Aspergillus terreus NIH2624]

4322926	711	384.92	95.08	-1.35821002	2.57E-06	1.69E-05	Down	ATEG_07410	hypothetical protein	ko04144//Endocytosis	GO:0030134//ER to Golgi transport vesicle;GO:0030173//integral component of Golgi membrane;GO:0030176//integral component of endoplasmic reticulum membrane;GO:0031965//nuclear membrane	GO:0097020//COPII adaptor activity	GO:0006486//protein glycosylation;GO:0006888//ER to Golgi vesicle-mediated transport;GO:0031505//fungal-type cell wall organization;GO:0045053//protein retention in Golgi apparatus	gi|115400886|ref|XP_001216031.1|/2.66229e-170/conserved hypothetical protein [Aspergillus terreus NIH2624]
4321640	2112	212.4	51.81	-1.357121115	0.00017989	0.00086425	Down	ATEG_05730	hypothetical protein	-	-	-	-	gi|115398638|ref|XP_001214908.1|/0/predicted protein [Aspergillus terreus NIH2624]
4319956	1020	554.6	137.17	-1.35616263	3.75E-07	2.77E-06	Down	ATEG_04445	hypothetical protein	-	-	-	-	gi|115395968|ref|XP_001213623.1|/0/predicted protein [Aspergillus terreus NIH2624]
4321283	726	122.14	28.6	-1.353018271	0.00537652	0.0181794	Down	ATEG_06044	hypothetical protein	-	-	-	-	gi|115399266|ref|XP_001215222.1|/4.42203e-176/conserved hypothetical protein [Aspergillus terreus NIH2624]
4318938	4181	94.17	22.24	-1.352730811	0.01286871	0.03849125	Down	ATEG_07322	hypothetical protein	-	GO:0005622//intracellular	GO:0000155//phosphorelay sensor kinase activity	GO:0000160//phosphorelay signal transduction system;GO:0023014//signal transduction by protein phosphorylation	gi|115386934|ref|XP_001210008.1|/0/conserved hypothetical protein [Aspergillus terreus NIH2624]
4315977	849	1717.92	434.76	-1.344013966	5.29E-14	7.18E-13	Down	ATEG_01367	hypothetical protein	-	-	-	-	gi|115384370|ref|XP_001208732.1|/0/predicted protein [Aspergillus terreus NIH2624]

4317547	969	726.97	183.06	-1.342346808	4.73E-09	4.29E-08	Down	ATEG_03275	hypothetical protein	ko01100//Metabolic pathways;ko04141//Protein processing in endoplasmic reticulum;ko00510//N-Glycan biosynthesis;ko00513//Various types of N-glycan biosynthesis	-	-	-	gi|115389896|ref|XP_001212453.1|/0/conserved hypothetical protein [Aspergillus terreus NIH2624]
4320222	2756	1348.11	342.97	-1.333625216	8.71E-13	1.10E-11	Down	ATEG_04660	endochitinase 1 precursor	ko01100//Metabolic pathways;ko00520//Amino sugar and nucleotide sugar metabolism	GO:0016021//integral component of membrane	GO:0004568//chitinase activity	GO:0005975//carbohydrate metabolic process;GO:0006032//chitin catabolic process	gi|115396398|ref|XP_001213838.1|/0/endochitinase 1 precursor [Aspergillus terreus NIH2624]
4355457	1602	511.61	128.47	-1.333074237	1.65E-07	1.27E-06	Down	ATEG_00702	hypothetical protein	-	-	GO:0004659//prenyltransferase activity;GO:0050364	GO:0045461;GO:1900796	gi|115492321|ref|XP_001210788.1|/0/predicted protein [Aspergillus terreus NIH2624]
4316314	1953	95.82	22.97	-1.330986217	0.00887619	0.02797659	Down	ATEG_01128	beta-glucan synthesis-associated protein KRE6	ko03013//RNA transport;ko03015//mRNA surveillance pathway	GO:0016021//integral component of membrane	GO:0004553//hydrolase activity, hydrolyzing O-glycosyl compounds	GO:0005975//carbohydrate metabolic process	gi|115383892|ref|XP_001208493.1|/0/beta-glucan synthesis-associated protein KRE6 [Aspergillus terreus NIH2624]
4318709	1827	164.7	40.67	-1.330742676	0.00166457	0.00646734	Down	ATEG_03758	hypothetical protein	ko01100//Metabolic pathways;ko01110//Biosynthesis of secondary metabolites	GO:0016021//integral component of membrane	GO:0004497//monooxygenase activity;GO:0005506//iron ion binding;GO:0016705//oxidoreductase activity, acting on paired donors, with incorporation or reduction of molecular oxygen;GO:0020037//heme binding	GO:0055114//oxidation-reduction process	gi|115390863|ref|XP_001212936.1|/0/conserved hypothetical protein [Aspergillus terreus NIH2624]

4320077	2121	136.17	33.38	-1.330201958	0.00245211	0.00914044	Down	ATEG_04692	hypothetical protein	ko01100//Metabolic pathways;ko01110//Biosynthesis of secondary metabolites;ko00100//Steroid biosynthesis	GO:0005634//nucleus	GO:0000981//RNA polymerase II transcription factor activity, sequence-specific DNA binding;GO:0008270//zinc ion binding	GO:0006357//regulation of transcription from RNA polymerase II promoter	gi|115396462|ref|XP_001213870.1|/0/predicted protein [Aspergillus terreus NIH2624]
4316437	762	271.76	67.18	-1.328971188	0.00011152	0.00055363	Down	ATEG_02028	hypothetical protein	ko01100//Metabolic pathways;ko01212//Fatty acid metabolism;ko00061//Fatty acid biosynthesis	GO:0016021//integral component of membrane	-	-	gi|115387401|ref|XP_001211206.1|/0/conserved hypothetical protein [Aspergillus terreus NIH2624]
4317143	1460	241.58	60.32	-1.325912425	0.00013545	0.00066266	Down	ATEG_02615	hypothetical protein	ko01100//Metabolic pathways;ko01110//Biosynthesis of secondary metabolites;ko01130//Biosynthesis of antibiotics;ko01220//Degradation of aromatic compounds;ko00350//Tyrosine metabolism;ko00010//Glycolysis / Gluconeogenesis;ko00071//Fatty acid degradation	-	GO:0008270//zinc ion binding;GO:0016491//oxidoreductase activity	GO:0055114//oxidation-reduction process	gi|115388575|ref|XP_001211793.1|/0/conserved hypothetical protein [Aspergillus terreus NIH2624]
4322575	486	389.44	98.87	-1.324044384	4.44E-06	2.80E-05	Down	ATEG_07613	hypothetical protein	-	GO:0016021//integral component of membrane	-	-	gi|115401292|ref|XP_001216234.1|/3.59962e-122/conserved hypothetical protein [Aspergillus terreus NIH2624]
4318011	3346	120.49	29.1	-1.320795413	0.00511357	0.01739691	Down	ATEG_03348	hypothetical protein	-	-	-	-	gi|115390042|ref|XP_001212526.1|/0/predicted protein [Aspergillus terreus NIH2624]

4354530	1701	139.57	34.54	-1.319668905	0.00624986	0.02069976	Down	ATEG_09736	hypothetical protein	ko04011//MAPK signaling pathway - yeast	GO:0005634//nucleus	GO:0003677//DNA binding;GO:0008270//zinc ion binding	GO:0006351//transcription, DNA-templated	gi|115443102|ref|XP_001218358.1|/0/predicted protein [Aspergillus terreus NIH2624]
4354715	2301	173.21	42.6	-1.319625252	0.00130669	0.00520182	Down	ATEG_10322	hypothetical protein	-	GO:0016021//integral component of membrane	GO:0016491//oxidoreductase activity	GO:0055114//oxidation-reduction process	gi|115449687|ref|XP_001218670.1|/0/conserved hypothetical protein [Aspergillus terreus NIH2624]
4320678	3679	828.42	213.41	-1.315660505	4.27E-08	3.53E-07	Down	ATEG_05141	hypothetical protein	ko04144//Endocytosis	-	-	-	gi|115397455|ref|XP_001214319.1|/0/conserved hypothetical protein [Aspergillus terreus NIH2624]
4319266	1228	427.48	108.07	-1.312755364	6.42E-06	3.93E-05	Down	ATEG_07010	hypothetical protein	-	GO:0030176//integral component of endoplasmic reticulum membrane	-	GO:2001256//regulation of store-operated calcium entry	gi|115386310|ref|XP_001209696.1|/0/conserved hypothetical protein [Aspergillus terreus NIH2624]
4317234	1913	563.6	142.36	-1.311083014	3.62E-06	2.32E-05	Down	ATEG_02683	hypothetical protein	ko01100//Metabolic pathways;ko00500//Starch and sucrose metabolism	-	-	-	gi|115388711|ref|XP_001211861.1|/0/conserved hypothetical protein [Aspergillus terreus NIH2624]

4321444	2019	5059.42	1306.9	-1.308471612	1.49E-18	2.72E-17	Down	ATEG_06040	78 kDa glucose-regulated protein precursor	ko04141//Protein processing in endoplasmic reticulum;ko03060//Protein export	GO:0005794//Golgi apparatus;GO:0031965//nuclear membrane;GO:0034099//luminal surveillance complex	GO:0005524//ATP binding;GO:0016887//ATPase activity;GO:0051082//unfolded protein binding	GO:0000742//karyogamy involved in conjugation with cellular fusion;GO:0006616//SRP-dependent cotranslational protein targeting to membrane, translocation;GO:0006986//response to unfolded protein;GO:0030433//ubiquitin-dependent ERAD pathway;GO:0031204//posttranslational protein targeting to membrane, translocation;GO:0051084//'de novo' posttranslational protein folding	gi|115399258|ref|XP_001215218.1|/0/78 kDa glucose-regulated protein precursor [Aspergillus terreus NIH2624]
4354363	1233	722.38	186.51	-1.306497362	2.47E-09	2.30E-08	Down	ATEG_09559	hypothetical protein	-	GO:0005634//nucleus;GO:0005829//cytosol	-	-	gi|115442748|ref|XP_001218181.1|/0/conserved hypothetical protein [Aspergillus terreus NIH2624]
4316572	567	6247.97	1619.09	-1.305589466	4.89E-23	1.18E-21	Down	ATEG_01954	hypothetical protein	ko04144//Endocytosis	-	-	-	gi|115387253|ref|XP_001211132.1|/4.7049e-129/predicted protein [Aspergillus terreus NIH2624]
4321736	1337	141.76	34.94	-1.305141929	0.00316548	0.01141934	Down	ATEG_05659	hypothetical protein	-	-	-	-	gi|115398496|ref|XP_001214837.1|/1.16221e-132/conserved hypothetical protein [Aspergillus terreus NIH2624]

4323544	3314	103.13	25.14	-1.304985616	0.0085071	0.02700342	Down	ATEG_08590	hypothetical protein	ko01100//Metabolic pathways;ko00520//Amino sugar and nucleotide sugar metabolism	-	GO:0004568//chitinase activity;GO:0008061//chitin binding	GO:0005975//carbohydrate metabolic process;GO:0006032//chitin catabolic process	gi|115402199|ref|XP_001217176.1|/0/predicted protein [Aspergillus terreus NIH2624]
4320860	2199	996.59	256.52	-1.304518294	4.30E-10	4.27E-09	Down	ATEG_05520	hypothetical protein	-	GO:0005634//nucleus	GO:0000981//RNA polymerase II transcription factor activity, sequence-specific DNA binding;GO:0003677//DNA binding;GO:0008270//zinc ion binding	GO:0006357//regulation of transcription from RNA polymerase II promoter	gi|115398213|ref|XP_001214698.1|/0/predicted protein [Aspergillus terreus NIH2624]
4322039	2819	115.39	28.3	-1.304472136	0.00702683	0.02290921	Down	ATEG_06533	hypothetical protein	ko01100//Metabolic pathways;ko00500//Starch and sucrose metabolism	GO:0016021//integral component of membrane	-	-	gi|115400245|ref|XP_001215711.1|/0/predicted protein [Aspergillus terreus NIH2624]
4315751	918	3076.29	801.4	-1.299861335	3.18E-19	6.19E-18	Down	ATEG_01431	hypothetical protein	ko04144//Endocytosis	GO:0005737//cytoplasm	-	-	gi|115384498|ref|XP_001208796.1|/0/conserved hypothetical protein [Aspergillus terreus NIH2624]
4317102	690	2146.2	553.93	-1.299214712	2.23E-08	1.87E-07	Down	ATEG_02136	hypothetical protein	ko03030//DNA replication	-	-	-	gi|115387617|ref|XP_001211314.1|/0/predicted protein [Aspergillus terreus NIH2624]
4353935	1241	9691.29	2546.65	-1.297096351	2.93E-15	4.29E-14	Down	ATEG_09034	hypothetical protein	ko01100//Metabolic pathways;ko00520//Amino sugar and nucleotide sugar metabolism	GO:0016020//membrane	-	-	gi|115436836|ref|XP_001217656.1|/3.90692e-150/predicted protein [Aspergillus terreus NIH2624]

4319611	3147	541.4	141.34	-1.293403037	9.03E-05	0.00045706	Down	ATEG_09965	3-hydroxy-3-methylglutaryl-coenzyme A reductase	ko01100//Metabolic pathways;ko01110//Biosynthesis of secondary metabolites;ko01130//Biosynthesis of antibiotics;ko00900//Terpenoid backbone biosynthesis	GO:0005789//endoplasmic reticulum membrane;GO:0016021//integral component of membrane	GO:0004420//hydroxymethylglutaryl-CoA reductase (NADPH) activity;GO:0050661//NADP binding	GO:0008299//isoprenoid biosynthetic process;GO:0015936//coenzyme A metabolic process;GO:0016126//sterol biosynthetic process;GO:0055114//oxidation-reduction process	gi|115385441|ref|XP_001209267.1|/0/3-hydroxy-3-methylglutaryl-coenzyme A reductase [Aspergillus terreus NIH2624]
4316739	1317	1085.89	280.27	-1.289272013	2.14E-08	1.80E-07	Down	ATEG_02579	hypothetical protein	ko01100//Metabolic pathways;ko00520//Amino sugar and nucleotide sugar metabolism	GO:0030134//ER to Golgi transport vesicle;GO:0030173//integral component of Golgi membrane;GO:0030176//integral component of endoplasmic reticulum membrane	-	GO:0006888//ER to Golgi vesicle-mediated transport	gi|115388503|ref|XP_001211757.1|/0/conserved hypothetical protein [Aspergillus terreus NIH2624]
4353173	1353	4861.48	1281.16	-1.287914347	8.93E-16	1.36E-14	Down	ATEG_08149	glycolipid-anchored surface protein 5 precursor	ko01100//Metabolic pathways;ko00500//Starch and sucrose metabolism	GO:0005886//plasma membrane;GO:0009277//fungal-type cell wall;GO:0016021//integral component of membrane;GO:0031225//anchored component of membrane	GO:0042124//1,3-beta-glucanosyltransferase activity	GO:0005975//carbohydrate metabolic process	gi|115433266|ref|XP_001216770.1|/0/glycolipid-anchored surface protein 5 precursor [Aspergillus terreus NIH2624]
4355616	1038	130.6	32.8	-1.283808453	0.00418049	0.01465826	Down	ATEG_00855	hypothetical protein	-	GO:0005634//nucleus	GO:0000981//RNA polymerase II transcription factor activity, sequence-specific DNA binding;GO:0008270//zinc ion binding	GO:0006357//regulation of transcription from RNA polymerase II promoter	gi|115492627|ref|XP_001210941.1|/0/predicted protein [Aspergillus terreus NIH2624]

4317402	1851	92.47	23.12	-1.279836744	0.01606062	0.04687303	Down	ATEG_02786	hypothetical protein	ko01100//Metabolic pathways;ko00520//Amino sugar and nucleotide sugar metabolism	-	-	-	gi|115388918|ref|XP_001211964.1|/0/predicted protein [Aspergillus terreus NIH2624]
4318907	906	1515.79	397.72	-1.279068222	1.11E-10	1.18E-09	Down	ATEG_06945	hypothetical protein	ko01100//Metabolic pathways;ko01110//Biosynthesis of secondary metabolites;ko01130//Biosynthesis of antibiotics;ko00100//Steroid biosynthesis	GO:0016021//integral component of membrane	GO:0008168//methyltransferase activity	GO:0032259//methylation	gi|115386180|ref|XP_001209631.1|/0/conserved hypothetical protein [Aspergillus terreus NIH2624]
4316021	2440	1460.36	381.19	-1.278669626	1.86E-08	1.58E-07	Down	ATEG_01588	similar to epoxide hydrolase	-	-	GO:0033961//cis-stilbene-oxide hydrolase activity	-	gi|115384812|ref|XP_001208953.1|/0/hypothetical protein ATEG_01588 [Aspergillus terreus NIH2624]
4323180	930	3892.04	1034.14	-1.274677744	5.21E-21	1.13E-19	Down	ATEG_08521	hypothetical protein	-	-	-	-	gi|115402061|ref|XP_001217107.1|/6.36172e-120/conserved hypothetical protein [Aspergillus terreus NIH2624]
4321468	3679	546.28	143.42	-1.273103602	3.91E-07	2.88E-06	Down	ATEG_05859	hypothetical protein	ko01100//Metabolic pathways;ko00561//Glycerolipid metabolism	-	GO:0016787//hydrolase activity	-	gi|115398896|ref|XP_001215037.1|/0/predicted protein [Aspergillus terreus NIH2624]
4355686	1065	6819.58	1810.32	-1.273047532	1.20E-24	3.17E-23	Down	ATEG_00923	hypothetical protein	-	GO:0016021//integral component of membrane	-	-	gi|115492763|ref|XP_001211009.1|/0/predicted protein [Aspergillus terreus NIH2624]

4316710	2973	3265.14	865.22	-1.271751657	2.61E-18	4.66E-17	Down	ATEG_02570	similar to sulfate permease SutB	-	GO:0016021//integral component of membrane	GO:0008271//secondary active sulfate transmembrane transporter activity	GO:1902358//sulfate transmembrane transport	gi|115388485|ref|XP_001211748.1|/0/hypothetical protein ATEG_02570 [Aspergillus terreus NIH2624]
4320191	6073	120.47	30.61	-1.269739775	0.00563913	0.01893092	Down	ATEG_04379	hypothetical protein	ko01100//Metabolic pathways;ko00562//Inositol phosphate metabolism;ko04070//Phosphatidylinositol signaling system	GO:0016021//integral component of membrane	GO:0015171//amino acid transmembrane transporter activity	GO:0003333//amino acid transmembrane transport	gi|115395836|ref|XP_001213557.1|/0/conserved hypothetical protein [Aspergillus terreus NIH2624]
4319660	1917	16605.29	4317.29	-1.269601048	0.0008504	0.00352599	Down	ATEG_10178	heat shock 70 kDa protein	ko04144//Endocytosis;ko04141//Protein processing in endoplasmic reticulum;ko03040//Spliceosome	GO:0005576//extracellular region;GO:0005622//intracellular	GO:0005524//ATP binding	-	gi|115385867|ref|XP_001209480.1|/0/heat shock 70 kDa protein [Aspergillus terreus NIH2624]
4320674	480	263.91	68.73	-1.266477372	0.00013609	0.0006654	Down	ATEG_05206	hypothetical protein	ko01100//Metabolic pathways;ko00520//Amino sugar and nucleotide sugar metabolism	-	-	-	gi|115397585|ref|XP_001214384.1|/7.58219e-114/predicted protein [Aspergillus terreus NIH2624]
4353873	1395	2879.56	769.7	-1.266148835	1.66E-17	2.82E-16	Down	ATEG_09167	hypothetical protein	ko01100//Metabolic pathways;ko00564//Glycerophospholipid metabolism;ko00565//Ether lipid metabolism	GO:0016021//integral component of membrane	-	-	gi|115437354|ref|XP_001217789.1|/0/conserved hypothetical protein [Aspergillus terreus NIH2624]
4320647	3515	1342.11	357.45	-1.266109519	5.29E-13	6.80E-12	Down	ATEG_05318	hypothetical protein	ko01110//Biosynthesis of secondary metabolites;ko00564//Glycerophospholipid metabolism;ko00561//Glycerolipid metabolism	GO:0016021//integral component of membrane	-	-	gi|115397809|ref|XP_001214496.1|/0/conserved hypothetical protein [Aspergillus terreus NIH2624]

4320932	621	1897.55	507.14	-1.265900145	8.99E-15	1.27E-13	Down	ATEG_05181	hypothetical protein	ko00310//Lysine degradation	-	-	-	gi|115397535|ref|XP_001214359.1|/8.63206e-143/predicted protein [Aspergillus terreus NIH2624]
4322071	745	1397.51	373.45	-1.26558219	4.74E-12	5.68E-11	Down	ATEG_06837	hypothetical protein	-	-	-	-	gi|115400853|ref|XP_001216015.1|/2.99852e-126/conserved hypothetical protein [Aspergillus terreus NIH2624]
4319310	2431	1286.61	342.91	-1.263822741	1.33E-12	1.66E-11	Down	ATEG_06899	similar to glutathione S-transferase domain protein	-	-	GO:0019120//hydrolase activity, acting on acid halide bonds, in C-halide compounds	GO:0008152//metabolic process	gi|115386088|ref|XP_001209585.1|/0/hypothetical protein ATEG_06899 [Aspergillus terreus NIH2624]
4321158	903	162.07	41.54	-1.26335135	0.00593006	0.01973813	Down	ATEG_05215	CDP-diacylglycerol--inositol 3-phosphatidyltransferase	ko01100//Metabolic pathways;ko00564//Glycerophospholipid metabolism;ko00562//Inositol phosphate metabolism;ko04070//Phosphatidylinositol signaling system	GO:0016021//integral component of membrane	GO:0016780//phosphotransferase activity, for other substituted phosphate groups	GO:0008654//phospholipid biosynthetic process	gi|115397603|ref|XP_001214393.1|/0/CDP-diacylglycerol--inositol 3-phosphatidyltransferase [Aspergillus terreus NIH2624]
4318002	1602	327.87	85.68	-1.262572604	4.16E-05	0.00022554	Down	ATEG_03075	hypothetical protein	ko01100//Metabolic pathways;ko01110//Biosynthesis of secondary metabolites;ko00500//Starch and sucrose metabolism;ko00460//Cyanoamino acid metabolism	-	GO:0003677//DNA binding	-	gi|115389496|ref|XP_001212253.1|/0/conserved hypothetical protein [Aspergillus terreus NIH2624]

4318402	3042	817.15	217.82	-1.253099521	6.90E-09	6.13E-08	Down	ATEG_03912	hypothetical protein	ko04011//MAPK signaling pathway - yeast	-	-	-	gi|115391171|ref|XP_001213090.1|/0/predicted protein [Aspergillus terreus NIH2624]
4316429	747	250.97	65.4	-1.251234947	0.00059865	0.00258261	Down	ATEG_02014	hypothetical protein	ko01100//Metabolic pathways;ko00563//Glycosylphosphatidylinositol(GPI)-anchor biosynthesis	-	-	-	gi|115387373|ref|XP_001211192.1|/3.91435e-178/conserved hypothetical protein [Aspergillus terreus NIH2624]
4316288	750	138.93	36.22	-1.250091488	0.00620752	0.02058131	Down	ATEG_01313	hypothetical protein	ko01100//Metabolic pathways;ko00520//Amino sugar and nucleotide sugar metabolism	-	-	-	gi|115384262|ref|XP_001208678.1|/9.3151e-157/predicted protein [Aspergillus terreus NIH2624]
4353435	996	280.32	74.59	-1.24619213	0.00256413	0.00949831	Down	ATEG_08202	hypothetical protein	-	GO:0005730//nucleolus;GO:0005737//cytoplasm	GO:0000994//RNA polymerase III core binding;GO:0016301//kinase activity	GO:0015031//protein transport;GO:0016310//phosphorylation;GO:0016480//negative regulation of transcription from RNA polymerase III promoter;GO:0061587//transfer RNA gene-mediated silencing;GO:0070217//transcription factor TFIIIB complex assembly	gi|115433372|ref|XP_001216823.1|/0/conserved hypothetical protein [Aspergillus terreus NIH2624]
4321500	1789	1467.3	397.13	-1.245782564	3.05E-12	3.71E-11	Down	ATEG_05784	hypothetical protein	ko00230//Purine metabolism	GO:0000324//fungal-type vacuole;GO:0016021//integral component of membrane	GO:0016787//hydrolase activity	-	gi|115398746|ref|XP_001214962.1|/0/conserved hypothetical protein [Aspergillus terreus NIH2624]

4320334	1307	1733.25	468.97	-1.244193308	1.73E-14	2.42E-13	Down	ATEG_04778	hypothetical protein	-	-	-	-	gi|115396634|ref|XP_001213956.1|/2.02663e-130/predicted protein [Aspergillus terreus NIH2624]
4354512	513	426.65	114.73	-1.242843778	0.00025362	0.00118689	Down	ATEG_09743	hypothetical protein	-	-	-	-	gi|115443116|ref|XP_001218365.1|/4.44358e-96/predicted protein [Aspergillus terreus NIH2624]
4316371	792	285.15	75.37	-1.241782489	0.00022425	0.00105607	Down	ATEG_01740	hypothetical protein	ko04144//Endocytosis	-	-	-	gi|115385116|ref|XP_001209105.1|/0/predicted protein [Aspergillus terreus NIH2624]
4318478	1194	664.25	179.13	-1.238584217	1.92E-07	1.47E-06	Down	ATEG_03944	similar to dehydrodolichyl diphosphate synthase	ko01110//Biosynthesis of secondary metabolites;ko00900//Terpenoid backbone biosynthesis	-	GO:0045547//dehydrodolichyl diphosphate synthase activity	GO:0006486//protein glycosylation	gi|115391235|ref|XP_001213122.1|/0/hypothetical protein ATEG_03944 [Aspergillus terreus NIH2624]
4354020	1239	172.04	45.46	-1.23628512	0.00154535	0.0060568	Down	ATEG_09235	hypothetical protein	ko01100//Metabolic pathways;ko00350//Tyrosine metabolism	-	GO:0004334//fumarylacetoacetase activity	GO:0009072//aromatic amino acid family metabolic process	gi|115437622|ref|XP_001217857.1|/0/conserved hypothetical protein [Aspergillus terreus NIH2624]
4355673	3027	225.19	59.66	-1.229331036	0.0010352	0.00422132	Down	ATEG_00910	hypothetical protein	ko01100//Metabolic pathways;ko00790//Folate biosynthesis	-	-	-	gi|115492737|ref|XP_001210996.1|/0/conserved hypothetical protein [Aspergillus terreus NIH2624]

4320517	1206	212.34	56.74	-1.229307027	0.00073676	0.00310696	Down	ATEG_04399	hypothetical protein	ko01100//Metabolic pathways;ko00230//Purine metabolism;ko00240//Pyrimidine metabolism;ko03020//RNA polymerase	-	-	-	gi|115395876|ref|XP_001213577.1|/0/conserved hypothetical protein [Aspergillus terreus NIH2624]
4317333	576	133.88	35.39	-1.228088964	0.01092569	0.03344724	Down	ATEG_02973	hypothetical protein	ko01100//Metabolic pathways	-	-	-	gi|115389292|ref|XP_001212151.1|/1.99517e-136/predicted protein [Aspergillus terreus NIH2624]
4355586	2790	18469.65	4885.93	-1.223872926	0.00806345	0.02578204	Down	ATEG_00825	heat shock protein HSP98	-	GO:0005635//nuclear envelope;GO:0005829//cytosol	GO:0005524//ATP binding;GO:0016887//ATPase activity;GO:0051787//misfolded protein binding	GO:0019538//protein metabolic process;GO:0042026//protein refolding;GO:0043335//protein unfolding;GO:0071218//cellular response to misfolded protein	gi|115492567|ref|XP_001210911.1|/0/heat shock protein HSP98 [Aspergillus terreus NIH2624]
4320122	1794	99.2	25.53	-1.223372784	0.01527201	0.04483645	Down	ATEG_04645	hypothetical protein	ko01100//Metabolic pathways;ko01110//Biosynthesis of secondary metabolites;ko00650//Butanoate metabolism;ko00250//Alanine, aspartate and glutamate metabolism;ko00410//beta-Alanine metabolism;ko00430//Taurine and hypotaurine metabolism	-	GO:0016831//carboxy-lyase activity;GO:0030170//pyridoxal phosphate binding	GO:0019752//carboxylic acid metabolic process	gi|115396368|ref|XP_001213823.1|/0/conserved hypothetical protein [Aspergillus terreus NIH2624]

4322198	1356	10518.78	2916.92	-1.223125553	1.11E-10	1.18E-09	Down	ATEG_06342	acyl-CoA desaturase	ko01212//Fatty acid metabolism;ko01040//Biosynthesis of unsaturated fatty acids	GO:0005783//endoplasmic reticulum;GO:0016021//integral component of membrane	GO:0004768//stearoyl-CoA 9-desaturase activity;GO:0005506//iron ion binding;GO:0020037//heme binding	GO:0006636//unsaturated fatty acid biosynthetic process;GO:0043936//asexual sporulation resulting in formation of a cellular spore;GO:0055114//oxidation-reduction process	gi|115399862|ref|XP_001215520.1|/0/acyl-CoA desaturase [Aspergillus terreus NIH2624]
4322569	837	528.28	143.32	-1.222152013	5.41E-06	3.36E-05	Down	ATEG_07473	hypothetical protein	-	GO:0005730//nucleolus	GO:0008270//zinc ion binding	-	gi|115401012|ref|XP_001216094.1|/0/conserved hypothetical protein [Aspergillus terreus NIH2624]
4320656	777	147.88	39.21	-1.221644312	0.00548999	0.01850309	Down	ATEG_05271	hypothetical protein	-	GO:0016021//integral component of membrane	GO:0008080//N-acetyltransferase activity	-	gi|115397715|ref|XP_001214449.1|/0/predicted protein [Aspergillus terreus NIH2624]
4320621	3330	812.88	220.86	-1.219882164	5.39E-07	3.89E-06	Down	ATEG_05580	hypothetical protein	-	GO:0005622//intracellular	-	GO:0035556//intracellular signal transduction	gi|115398333|ref|XP_001214758.1|/0/conserved hypothetical protein [Aspergillus terreus NIH2624]
4355664	2063	394.53	107.51	-1.217144499	8.95E-06	5.37E-05	Down	ATEG_00903	hypothetical protein	ko04111//Cell cycle - yeast;ko04113//Meiosis - yeast;ko04120//Ubiquitin mediated proteolysis	GO:0016021//integral component of membrane	-	GO:0055085//transmembrane transport	gi|115492723|ref|XP_001210989.1|/0/conserved hypothetical protein [Aspergillus terreus NIH2624]
4320032	1871	1061.56	292.17	-1.216316568	4.46E-10	4.41E-09	Down	ATEG_04681	hypothetical protein	-	GO:0016021//integral component of membrane	-	-	gi|115396440|ref|XP_001213859.1|/2.5495e-158/predicted protein [Aspergillus terreus NIH2624]

4318483	1182	397.94	109.45	-1.214754275	0.00042976	0.00191566	Down	ATEG_03658	similar to Png1p	ko04141//Protein processing in endoplasmic reticulum	-	-	-	gi|115390663|ref|XP_001212836.1|/0/hypothetical protein ATEG_03658 [Aspergillus terreus NIH2624]
4316602	1116	880.45	242.65	-1.213768613	1.85E-09	1.74E-08	Down	ATEG_01944	hypothetical protein	ko01100//Metabolic pathways;ko00520//Amino sugar and nucleotide sugar metabolism	-	GO:0004568//chitinase activity	GO:0005975//carbohydrate metabolic process;GO:0006032//chitin catabolic process	gi|115387233|ref|XP_001211122.1|/0/conserved hypothetical protein [Aspergillus terreus NIH2624]
4322431	1722	355.28	97.55	-1.212658113	4.38E-05	0.00023624	Down	ATEG_06282	hypothetical protein	-	GO:0016021//integral component of membrane	-	GO:0055085//transmembrane transport	gi|115399740|ref|XP_001215460.1|/0/conserved hypothetical protein [Aspergillus terreus NIH2624]
4316376	759	2234.83	601.16	-1.208269222	0.01150821	0.03501347	Down	ATEG_01439	hypothetical protein	-	GO:0016020//membrane	-	-	gi|115384514|ref|XP_001208804.1|/0/predicted protein [Aspergillus terreus NIH2624]
4320897	893	749.49	207.41	-1.206362407	4.40E-07	3.21E-06	Down	ATEG_05093	similar to : Glutathione S-transferase	ko00480//Glutathione metabolism	GO:0005741//mitochondrial outer membrane;GO:0005783//endoplasmic reticulum	GO:0004364//glutathione transferase activity;GO:0004602//glutathione peroxidase activity	GO:0006749//glutathione metabolic process;GO:0055114//oxidation-reduction process;GO:0098869//cellular oxidant detoxification	gi|115397359|ref|XP_001214271.1|/0/hypothetical protein ATEG_05093 [Aspergillus terreus NIH2624]
4319018	507	398.45	109.96	-1.203991565	1.09E-05	6.45E-05	Down	ATEG_07271	hypothetical protein	ko04144//Endocytosis	-	GO:0005488	-	gi|115386832|ref|XP_001209957.1|/8.37563e-120/predicted protein [Aspergillus terreus NIH2624]

4317427	1302	2621.29	732.09	-1.202726304	1.69E-15	2.50E-14	Down	ATEG_02951	similar to metacaspase	-	-	GO:0008234//cysteine-type peptidase activity	GO:0006508//proteolysis;GO:0006915//apoptotic process	gi|115389248|ref|XP_001212129.1|/0/hypothetical protein ATEG_02951 [Aspergillus terreus NIH2624]
4318077	1503	108.14	29.02	-1.19846283	0.01688036	0.04880999	Down	ATEG_03408	hypothetical protein	-	-	-	-	gi|115390162|ref|XP_001212586.1|/0/predicted protein [Aspergillus terreus NIH2624]
4322633	1980	477.99	132.93	-1.196202556	2.66E-06	1.74E-05	Down	ATEG_07549	hypothetical protein	-	-	-	-	gi|115401164|ref|XP_001216170.1|/0/predicted protein [Aspergillus terreus NIH2624]
4323190	1449	2020.07	567.67	-1.194827444	3.16E-13	4.09E-12	Down	ATEG_08675	hypothetical protein	ko04141//Protein processing in endoplasmic reticulum	GO:0005829//cytosol	-	-	gi|115402369|ref|XP_001217261.1|/0/conserved hypothetical protein [Aspergillus terreus NIH2624]
4318774	3765	511.75	141.31	-1.193059408	0.00094333	0.0038719	Down	ATEG_04123	similar to : Cation transport ATPase	-	GO:0016021//integral component of membrane	GO:0000166//nucleotide binding;GO:0005507//copper ion binding;GO:0019829//cation-transporting ATPase activity	GO:0030001//metal ion transport;GO:0098655//cation transmembrane transport	gi|115391593|ref|XP_001213301.1|/0/hypothetical protein ATEG_04123 [Aspergillus terreus NIH2624]
4319635	711	237.54	64.48	-1.19281691	0.00111552	0.00450772	Down	ATEG_10209	hypothetical protein	-	GO:0005634//nucleus;GO:0005829//cytosol	GO:0016300//tRNA (uracil) methyltransferase activity	GO:0001300//chronological cell aging;GO:0002098//tRNA wobble uridine modification;GO:0030488//tRNA methylation;GO:0035690//cellular response to drug	gi|115385929|ref|XP_001209511.1|/3.00659e-176/conserved hypothetical protein [Aspergillus terreus NIH2624]

4317484	912	767.17	215.33	-1.192484782	8.65E-08	6.88E-07	Down	ATEG_03440	hypothetical protein	ko01100//Metabolic pathways;ko00280//Valine, leucine and isoleucine degradation	-	GO:0008270//zinc ion binding;GO:0016491//oxidoreductase activity	GO:0055114//oxidation-reduction process	gi|115390226|ref|XP_001212618.1|/0/conserved hypothetical protein [Aspergillus terreus NIH2624]
4321124	1647	350.9	97.39	-1.191883751	7.01E-05	0.00036317	Down	ATEG_05280	hypothetical protein	-	GO:0016021//integral component of membrane	GO:0022857//transmembrane transporter activity	GO:0055085//transmembrane transport	gi|115397733|ref|XP_001214458.1|/0/conserved hypothetical protein [Aspergillus terreus NIH2624]
4318519	901	440.99	123.23	-1.191287385	1.10E-05	6.50E-05	Down	ATEG_04230	hypothetical protein	ko01100//Metabolic pathways;ko00230//Purine metabolism	-	-	-	gi|115391807|ref|XP_001213408.1|/2.21595e-149/predicted protein [Aspergillus terreus NIH2624]
4317588	1695	2725.18	758.51	-1.190664216	1.91E-10	1.98E-09	Down	ATEG_03166	calnexin precursor	ko04141//Protein processing in endoplasmic reticulum;ko04145//Phagosome	GO:0005783//endoplasmic reticulum;GO:0016021//integral component of membrane	GO:0005509//calcium ion binding;GO:0030246//carbohydrate binding;GO:0051082//unfolded protein binding	GO:0006457//protein folding	gi|115389678|ref|XP_001212344.1|/0/calnexin precursor [Aspergillus terreus NIH2624]
4319293	825	233.68	64.3	-1.18953057	0.00048682	0.00214652	Down	ATEG_07329	hypothetical protein	ko00254//Aflatoxin biosynthesis	-	-	-	gi|115386948|ref|XP_001210015.1|/0/conserved hypothetical protein [Aspergillus terreus NIH2624]
4355238	2580	728.09	204.37	-1.189374449	2.10E-07	1.60E-06	Down	ATEG_00484	glycogen phosphorylase	ko01100//Metabolic pathways;ko01110//Biosynthesis of secondary metabolites;ko00500//Starch and sucrose metabolism;ko04931//Insulin resistance	-	GO:0008184//glycogen phosphorylase activity;GO:0030170//pyridoxal phosphate binding	GO:0005975//carbohydrate metabolic process	gi|115491885|ref|XP_001210570.1|/0/glycogen phosphorylase [Aspergillus terreus NIH2624]

4321888	729	348.45	96.97	-1.188725541	0.00012339	0.00060872	Down	ATEG_06357	hypothetical protein	ko04130//SNARE interactions in vesicular transport	GO:0005789//endoplasmic reticulum membrane;GO:0005794//Golgi apparatus;GO:0012507//ER to Golgi transport vesicle membrane;GO:0016021//integral component of membrane;GO:0031201//SNARE complex	GO:0005484//SNAP receptor activity	GO:0006888//ER to Golgi vesicle-mediated transport;GO:0048280//vesicle fusion with Golgi apparatus	gi|115399892|ref|XP_001215535.1|/2.97452e-170/conserved hypothetical protein [Aspergillus terreus NIH2624]
4317261	1357	361.33	100.48	-1.188142793	9.74E-05	0.00048974	Down	ATEG_02662	hypothetical protein	ko01100//Metabolic pathways;ko01110//Biosynthesis of secondary metabolites;ko01230//Biosynthesis of amino acids;ko00260//Glycine, serine and threonine metabolism;ko00400//Phenylalanine, tyrosine and tryptophan biosynthesis	-	-	-	gi|115388669|ref|XP_001211840.1|/0/conserved hypothetical protein [Aspergillus terreus NIH2624]
4319021	1311	1344.02	379.97	-1.1868491	4.74E-09	4.30E-08	Down	ATEG_07274	hypothetical protein	-	GO:0016021//integral component of membrane	-	-	gi|115386838|ref|XP_001209960.1|/0/conserved hypothetical protein [Aspergillus terreus NIH2624]
4355728	851	325.09	91.03	-1.184493962	0.00037958	0.00171548	Down	ATEG_00965	hypothetical protein	ko04144//Endocytosis	-	-	-	gi|115492847|ref|XP_001211051.1|/1.84512e-92/conserved hypothetical protein [Aspergillus terreus NIH2624]

4354515	834	1047.28	296.99	-1.184050277	6.17E-07	4.40E-06	Down	ATEG_09746	hypothetical protein	ko01130//Biosynthesis of antibiotics	-	GO:0008080//N-acetyltransferase activity	-	gi|115443122|ref|XP_001218368.1|/6.32849e-164/predicted protein [Aspergillus terreus NIH2624]
4353431	1211	1152.48	322.75	-1.183455603	1.02E-08	8.85E-08	Down	ATEG_08198	hypothetical protein	ko01100//Metabolic pathways;ko00500//Starch and sucrose metabolism	GO:0030134//ER to Golgi transport vesicle;GO:0030173//integral component of Golgi membrane;GO:0030176//integral component of endoplasmic reticulum membrane	-	GO:0006888//ER to Golgi vesicle-mediated transport	gi|115433364|ref|XP_001216819.1|/0/conserved hypothetical protein [Aspergillus terreus NIH2624]
4323234	2250	929.3	262.36	-1.181961757	1.19E-08	1.02E-07	Down	ATEG_08801	hypothetical protein	-	GO:0005829//cytosol;GO:0032153//cell division site	GO:0005096//GTPase activator activity	GO:0035024//negative regulation of Rho protein signal transduction;GO:0043547//positive regulation of GTPase activity	gi|115402621|ref|XP_001217387.1|/0/conserved hypothetical protein [Aspergillus terreus NIH2624]
4355239	6704	1040.2	294.5	-1.177219442	7.75E-10	7.53E-09	Down	ATEG_00485	hypothetical protein	-	-	GO:0004601//peroxidase activity	GO:0098869//cellular oxidant detoxification	gi|115491887|ref|XP_001210571.1|/0/predicted protein [Aspergillus terreus NIH2624]
4315775	4211	892.2	251.54	-1.1750609	1.08E-08	9.27E-08	Down	ATEG_01731	hypothetical protein	-	GO:0016021//integral component of membrane	-	-	gi|115385098|ref|XP_001209096.1|/0/conserved hypothetical protein [Aspergillus terreus NIH2624]
4316247	1407	523.44	147.57	-1.174235264	1.43E-06	9.68E-06	Down	ATEG_01871	similar to malolactic enzyme	ko01200//Carbon metabolism;ko00620//Pyruvate metabolism	-	GO:0004471//malate dehydrogenase (decarboxylating) (NAD+) activity;GO:0046872//metal ion binding;GO:0051287//NAD binding	GO:0006108//malate metabolic process;GO:0055114//oxidation-reduction process	gi|115385378|ref|XP_001209236.1|/0/hypothetical protein ATEG_01871 [Aspergillus terreus NIH2624]

4316042	1377	2034.59	578.34	-1.172807161	8.01E-13	1.02E-11	Down	ATEG_01530	cAMP-independent regulatory protein pac2	ko04144//Endocytosis	-	-	-	gi|115384696|ref|XP_001208895.1|/0/cAMP-independent regulatory protein pac2 [Aspergillus terreus NIH2624]
4316642	1533	166.99	46.45	-1.171864882	0.01265603	0.03798833	Down	ATEG_02003	hypothetical protein	-	-	-	-	gi|115387351|ref|XP_001211181.1|/0/conserved hypothetical protein [Aspergillus terreus NIH2624]
4354889	774	145.65	40.33	-1.171388586	0.00972851	0.03018604	Down	ATEG_00132	hypothetical protein	ko01100//Metabolic pathways;ko00500//Starch and sucrose metabolism	-	-	-	gi|115491181|ref|XP_001210218.1|/0/conserved hypothetical protein [Aspergillus terreus NIH2624]
4323285	1382	648.5	185.21	-1.165623237	2.30E-06	1.52E-05	Down	ATEG_08691	hypothetical protein	-	GO:0016021//integral component of membrane	GO:0005216//ion channel activity	GO:0034220//ion transmembrane transport	gi|115402401|ref|XP_001217277.1|/9.61589e-180/conserved hypothetical protein [Aspergillus terreus NIH2624]
4320376	1068	915.01	260.28	-1.165166277	1.05E-08	9.12E-08	Down	ATEG_04419	similar to 2-nitropropane dioxygenase	-	-	GO:0018580//nitronate monooxygenase activity;GO:0051213//dioxygenase activity	GO:0055114//oxidation-reduction process	gi|115395916|ref|XP_001213597.1|/0/hypothetical protein ATEG_04419 [Aspergillus terreus NIH2624]
4319995	3880	2744.65	788.15	-1.165037705	6.44E-13	8.22E-12	Down	ATEG_04702	hypothetical protein	ko01100//Metabolic pathways;ko00500//Starch and sucrose metabolism	GO:0016021//integral component of membrane;GO:0032153//cell division site;GO:0051286//cell tip	-	-	gi|115396482|ref|XP_001213880.1|/0/conserved hypothetical protein [Aspergillus terreus NIH2624]

4317323	2436	2989.44	855.88	-1.16403708	6.01E-16	9.31E-15	Down	ATEG_03268	similar to stress response regulator SrrA	-	GO:0005634//nucleus	GO:0000156//phosphorelay response regulator activity;GO:0003700//transcription factor activity, sequence-specific DNA binding;GO:0043565//sequence-specific DNA binding	GO:0000160//phosphorelay signal transduction system;GO:0006355//regulation of transcription, DNA-templated;GO:0006950//response to stress	gi|115389882|ref|XP_001212446.1|/0/hypothetical protein ATEG_03268 [Aspergillus terreus NIH2624]
4354275	1197	657.32	187.01	-1.162082374	2.28E-07	1.72E-06	Down	ATEG_09807	activator 1 subunit 3	ko03420//Nucleotide excision repair;ko03030//DNA replication;ko03430//Mismatch repair	GO:0005634//nucleus;GO:0005663//DNA replication factor C complex;GO:0005829//cytosol;GO:0031389//Rad17 RFC-like complex;GO:0031390//Ctf18 RFC-like complex;GO:0031391//Elg1 RFC-like complex	GO:0003677//DNA binding;GO:0005524//ATP binding;GO:0016887//ATPase activity	GO:0006272//leading strand elongation;GO:0007062//sister chromatid cohesion	gi|115443244|ref|XP_001218429.1|/0/activator 1 subunit 3 [Aspergillus terreus NIH2624]
4316359	5475	3820.94	1095.79	-1.161768996	8.05E-09	7.09E-08	Down	ATEG_01319	chitin synthase 6	ko00520//Amino sugar and nucleotide sugar metabolism	GO:0016021//integral component of membrane;GO:0016459//myosin complex	GO:0003677//DNA binding;GO:0003774//motor activity;GO:0005524//ATP binding;GO:0016758//transferase activity, transferring hexosyl groups	-	gi|115384274|ref|XP_001208684.1|/0/chitin synthase 6 [Aspergillus terreus NIH2624]
4321708	579	175.41	49.27	-1.160760863	0.00336561	0.01206245	Down	ATEG_05710	D-tyrosyl-tRNA(Tyr) deacylase	-	GO:0005737//cytoplasm	GO:0016788//hydrolase activity, acting on ester bonds	GO:0019478//D-amino acid catabolic process	gi|115398598|ref|XP_001214888.1|/6.17557e-136/D-tyrosyl-tRNA(Tyr) deacylase [Aspergillus terreus NIH2624]

4315579	2002	1616.02	464.97	-1.158414604	9.95E-12	1.16E-10	Down	ATEG_01625	hypothetical protein	ko00500//Starch and sucrose metabolism;ko04011//MAPK signaling pathway - yeast	-	-	-	gi|115384886|ref|XP_001208990.1|/0/conserved hypothetical protein [Aspergillus terreus NIH2624]
4317980	3282	2713.56	780.16	-1.157379218	7.12E-14	9.53E-13	Down	ATEG_03518	copper amine oxidase 1	ko01100//Metabolic pathways;ko01110//Biosynthesis of secondary metabolites;ko00350//Tyrosine metabolism;ko00260//Glycine, serine and threonine metabolism;ko00360//Phenylalanine metabolism;ko00410//beta-Alanine metabolism	-	GO:0005507//copper ion binding;GO:0008131//primary amine oxidase activity;GO:0048038//quinone binding	GO:0009308//amine metabolic process;GO:0055114//oxidation-reduction process	gi|115390382|ref|XP_001212696.1|/0/copper amine oxidase 1 [Aspergillus terreus NIH2624]
4320203	1316	138.99	38.8	-1.155724751	0.00686454	0.02243786	Down	ATEG_04522	hypothetical protein	-	-	-	-	gi|115396122|ref|XP_001213700.1|/7.83865e-86/predicted protein [Aspergillus terreus NIH2624]
4317454	1595	1336.38	385.65	-1.155499459	6.19E-10	6.06E-09	Down	ATEG_03219	hypothetical protein	ko03040//Spliceosome	GO:0000243//commitment complex;GO:0005685//U1 snRNP;GO:0071004//U2-type prespliceosome	GO:0003729//mRNA binding;GO:0008270//zinc ion binding;GO:0030619//U1 snRNA binding	GO:0000387//spliceosomal snRNP assembly;GO:0000395//mRNA 5'-splice site recognition	gi|115389784|ref|XP_001212397.1|/3.06275e-156/predicted protein [Aspergillus terreus NIH2624]

4323568	1449	384.91	110.1	-1.154428896	6.94E-05	0.00035996	Down	ATEG_08627	hypothetical protein	ko01100//Metabolic pathways;ko01130//Biosynthesis of antibiotics;ko01230//Biosynthesis of amino acids;ko00260//Glycine, serine and threonine metabolism;ko00270//Cysteine and methionine metabolism;ko00450//Selenocompound metabolism	-	-	-	gi|115402273|ref|XP_001217213.1|/0/conserved hypothetical protein [Aspergillus terreus NIH2624]
4353410	1306	2373.36	685.83	-1.153980527	2.57E-14	3.55E-13	Down	ATEG_08264	similar to 2-nitropropane dioxygenase	-	-	GO:0018580//nitronate monooxygenase activity;GO:0051213//dioxygenase activity	GO:0055114//oxidation-reduction process	gi|115433496|ref|XP_001216885.1|/0/hypothetical protein ATEG_08264 [Aspergillus terreus NIH2624]
4354446	2112	876.53	252.34	-1.153781532	1.39E-08	1.19E-07	Down	ATEG_09588	hypothetical protein	ko04144//Endocytosis	GO:0005724//nuclear telomeric heterochromatin;GO:0005739//mitochondrion	-	-	gi|115442806|ref|XP_001218210.1|/0/conserved hypothetical protein [Aspergillus terreus NIH2624]
4315545	1341	819.31	234.47	-1.153240002	4.21E-08	3.48E-07	Down	ATEG_01263	hypothetical protein	-	GO:0031011//Ino80 complex	-	GO:0006338//chromatin remodeling;GO:0006355//regulation of transcription, DNA-templated	gi|115384162|ref|XP_001208628.1|/0/conserved hypothetical protein [Aspergillus terreus NIH2624]
4317846	675	336.24	96.43	-1.153074295	0.00031977	0.00146937	Down	ATEG_02800	hypothetical protein	-	-	-	-	gi|115388946|ref|XP_001211978.1|/2.76292e-157/conserved hypothetical protein [Aspergillus terreus NIH2624]

4355546	384	141.77	39.79	-1.152824484	0.00770388	0.02477229	Down	ATEG_00785	hypothetical protein	-	-	-	-	gi|115492487|ref|XP_001210871.1|/3.64272e-92/conserved hypothetical protein [Aspergillus terreus NIH2624]
4353170	558	624.3	178.79	-1.149817153	6.38E-07	4.53E-06	Down	ATEG_08146	hypothetical protein	ko04144//Endocytosis	-	-	-	gi|115433260|ref|XP_001216767.1|/5.96143e-126/predicted protein [Aspergillus terreus NIH2624]
4315724	2487	348.53	100.16	-1.146613927	9.10E-05	0.00046002	Down	ATEG_01393	similar to breast cancer resistance protein	ko02010//ABC transporters	GO:0016021//integral component of membrane	GO:0005524//ATP binding;GO:0008080//N-acetyltransferase activity;GO:0016887//ATPase activity	-	gi|115384422|ref|XP_001208758.1|/0/hypothetical protein ATEG_01393 [Aspergillus terreus NIH2624]
4320131	1920	136.18	38.36	-1.143224265	0.00800795	0.02562707	Down	ATEG_04596	hypothetical protein	-	-	-	-	gi|115396270|ref|XP_001213774.1|/0/conserved hypothetical protein [Aspergillus terreus NIH2624]
4322779	657	122.16	34.37	-1.141857732	0.01292005	0.03859563	Down	ATEG_07687	hypothetical protein	ko04141//Protein processing in endoplasmic reticulum	GO:0005635//nuclear envelope;GO:0005829//cytosol	-	GO:0006020//inositol metabolic process	gi|115401440|ref|XP_001216308.1|/9.67628e-155/conserved hypothetical protein [Aspergillus terreus NIH2624]
4319235	747	124.96	35.25	-1.138672363	0.01184607	0.03585536	Down	ATEG_06866	hypothetical protein	-	-	-	GO:0006078//(1->6)-beta-D-glucan biosynthetic process;GO:0042546//cell wall biogenesis	gi|115386022|ref|XP_001209552.1|/2.87802e-180/predicted protein [Aspergillus terreus NIH2624]

4320494	2960	1103.05	317.61	-1.135248246	3.11E-07	2.32E-06	Down	ATEG_04282	hypothetical protein	ko01100//Metabolic pathways;ko00500//Starch and sucrose metabolism	GO:0016021//integral component of membrane	-	-	gi|115394898|ref|XP_001213460.1|/0/conserved hypothetical protein [Aspergillus terreus NIH2624]
4322136	2254	740.24	215.9	-1.134727349	1.46E-07	1.13E-06	Down	ATEG_06456	similar to alcohol dehydrogenase	ko01100//Metabolic pathways;ko00051//Fructose and mannose metabolism;ko00040//Pentose and glucuronate interconversions	-	GO:0008270//zinc ion binding;GO:0016491//oxidoreductase activity	GO:0055114//oxidation-reduction process	gi|115400091|ref|XP_001215634.1|/0/hypothetical protein ATEG_06456 [Aspergillus terreus NIH2624]
4322449	1464	275.81	79.72	-1.133256557	0.00780611	0.02505807	Down	ATEG_06266	hypothetical protein	-	-	-	-	gi|115399676|ref|XP_001215444.1|/0/conserved hypothetical protein [Aspergillus terreus NIH2624]
4322565	1308	326.11	94.93	-1.129990097	0.00030143	0.00139129	Down	ATEG_07469	hypothetical protein	ko04144//Endocytosis	-	GO:0016491//oxidoreductase activity	GO:0055114//oxidation-reduction process	gi|115401004|ref|XP_001216090.1|/0/conserved hypothetical protein [Aspergillus terreus NIH2624]
4353343	558	262.18	76.08	-1.1292591	0.00188976	0.00724628	Down	ATEG_08378	hypothetical protein	-	GO:0016021//integral component of membrane	-	-	gi|115433725|ref|XP_001216999.1|/1.16221e-132/conserved hypothetical protein [Aspergillus terreus NIH2624]
4320246	860	273.97	79.35	-1.127851297	0.00055977	0.00243051	Down	ATEG_04620	hypothetical protein	ko03013//RNA transport;ko03015//mRNA surveillance pathway	-	-	-	gi|115396318|ref|XP_001213798.1|/2.31754e-143/predicted protein [Aspergillus terreus NIH2624]

4323509	912	145.65	41.54	-1.127279188	0.00974116	0.0302032	Down	ATEG_08703	similar to UDP-glucose 4-epimerase	ko01100//Metabolic pathways;ko01110//Biosynthesis of secondary metabolites;ko01130//Biosynthesis of antibiotics;ko00520//Amino sugar and nucleotide sugar metabolism;ko00010//Glycolysis / Gluconeogenesis;ko00052//Galactose metabolism	-	GO:0003824//catalytic activity;GO:0050662//coenzyme binding	-	gi|115402425|ref|XP_001217289.1|/0/hypothetical protein ATEG_08703 [Aspergillus terreus NIH2624]
4316849	1011	277.97	79.8	-1.125210703	0.00057045	0.00247228	Down	ATEG_02547	hypothetical protein	ko04146//Peroxisome	GO:0005779//integral component of peroxisomal membrane	GO:0000295//adenine nucleotide transmembrane transporter activity	GO:0006635//fatty acid beta-oxidation;GO:0007031//peroxisome organization;GO:0015867//ATP transport;GO:0055085//transmembrane transport	gi|115388439|ref|XP_001211725.1|/0/conserved hypothetical protein [Aspergillus terreus NIH2624]
4320367	1296	1978.03	579.99	-1.124937843	2.82E-11	3.17E-10	Down	ATEG_04904	hypothetical protein	-	GO:0016021//integral component of membrane	-	-	gi|115396886|ref|XP_001214082.1|/0/conserved hypothetical protein [Aspergillus terreus NIH2624]
4316144	2931	8611.61	2542.86	-1.123392222	2.22E-20	4.59E-19	Down	ATEG_01371	hypothetical protein	ko04144//Endocytosis	-	-	-	gi|115384378|ref|XP_001208736.1|/0/conserved hypothetical protein [Aspergillus terreus NIH2624]
4323196	1662	8593.69	2528.08	-1.119250009	1.69E-15	2.50E-14	Down	ATEG_08658	hypothetical protein	-	GO:0016021//integral component of membrane	GO:0015171//amino acid transmembrane transporter activity	GO:0003333//amino acid transmembrane transport	gi|115402335|ref|XP_001217244.1|/0/conserved hypothetical protein [Aspergillus terreus NIH2624]

4318302	1563	589.46	173.31	-1.11769655	1.95E-06	1.30E-05	Down	ATEG_04032	hypothetical protein	-	GO:0005634//nucleus;GO:0005829//cytosol	GO:0008270//zinc ion binding	GO:0000070//mitotic sister chromatid segregation	gi|115391411|ref|XP_001213210.1|/0/conserved hypothetical protein [Aspergillus terreus NIH2624]
4319309	3807	1215.62	360.73	-1.116402621	1.39E-08	1.19E-07	Down	ATEG_06898	hypothetical protein	-	GO:0005938//cell cortex	GO:0005543//phospholipid binding	GO:0032065//cortical protein anchoring	gi|115386086|ref|XP_001209584.1|/0/conserved hypothetical protein [Aspergillus terreus NIH2624]
4354523	1509	470.67	138.04	-1.115170805	1.41E-05	8.22E-05	Down	ATEG_09729	hypothetical protein	-	GO:0016592//mediator complex	GO:0001104//RNA polymerase II transcription cofactor activity	GO:0006357//regulation of transcription from RNA polymerase II promoter	gi|115443088|ref|XP_001218351.1|/3.1388e-59/conserved hypothetical protein [Aspergillus terreus NIH2624]
4317062	2639	397.31	117.88	-1.106030396	8.68E-05	0.00044048	Down	ATEG_02164	inorganic phosphate transporter PHO84	-	GO:0016021//integral component of membrane	GO:0008080//N-acetyltransferase activity;GO:0022857//transmembrane transporter activity	GO:0055085//transmembrane transport	gi|115387673|ref|XP_001211342.1|/0/inorganic phosphate transporter PHO84 [Aspergillus terreus NIH2624]
4321053	952	391.21	115.77	-1.103604813	5.96E-05	0.00031197	Down	ATEG_05360	hypothetical protein	-	GO:0016592//mediator complex	GO:0001104//RNA polymerase II transcription cofactor activity	GO:0006357//regulation of transcription from RNA polymerase II promoter	gi|115397893|ref|XP_001214538.1|/0/conserved hypothetical protein [Aspergillus terreus NIH2624]
4317022	1155	466.13	137.42	-1.102759341	4.56E-05	0.00024497	Down	ATEG_02745	hypothetical protein	ko01100//Metabolic pathways;ko00564//Glycerophospholipid metabolism;ko00565//Ether lipid metabolism	GO:0016021//integral component of membrane	GO:0016491//oxidoreductase activity	GO:0055114//oxidation-reduction process	gi|115388835|ref|XP_001211923.1|/0/predicted protein [Aspergillus terreus NIH2624]

4322998	723	226.25	66.48	-1.098892067	0.00705839	0.02298017	Down	ATEG_07862	hypothetical protein	ko01100//Metabolic pathways;ko00350//Tyrosine metabolism	-	GO:0008171//O-methyltransferase activity	GO:0032259//methylation	gi|115401790|ref|XP_001216483.1|/1.5385e-171/predicted protein [Aspergillus terreus NIH2624]
4317613	1888	1853.2	556.85	-1.09866753	2.15E-11	2.44E-10	Down	ATEG_03249	similar to phosphatidylserine synthase	ko01100//Metabolic pathways;ko01110//Biosynthesis of secondary metabolites;ko00260//Glycine, serine and threonine metabolism;ko00564//Glycerophospholipid metabolism	GO:0005789//endoplasmic reticulum membrane;GO:0005829//cytosol;GO:0016021//integral component of membrane	GO:0003882//CDP-diacylglycerol-serine O-phosphatidyltransferase activity	GO:0006646//phosphatidylethanolamine biosynthetic process;GO:0006659//phosphatidylserine biosynthetic process;GO:0010512//negative regulation of phosphatidylinositol biosynthetic process;GO:0070317//negative regulation of G0 to G1 transition;GO:0071852//fungal-type cell wall organization or biogenesis	gi|115389844|ref|XP_001212427.1|/0/hypothetical protein ATEG_03249 [Aspergillus terreus NIH2624]
4317424	2759	576.74	172.33	-1.098271498	6.87E-06	4.18E-05	Down	ATEG_02948	hypothetical protein	ko01100//Metabolic pathways;ko00052//Galactose metabolism	GO:0005634//nucleus;GO:0016021//integral component of membrane	GO:0000981//RNA polymerase II transcription factor activity, sequence-specific DNA binding;GO:0003677//DNA binding;GO:0008270//zinc ion binding	GO:0006357//regulation of transcription from RNA polymerase II promoter	gi|115389242|ref|XP_001212126.1|/0/predicted protein [Aspergillus terreus NIH2624]

4321412	1605	1466.65	441.75	-1.097163477	9.52E-09	8.33E-08	Down	ATEG_05929	hypothetical protein	ko01100//Metabolic pathways;ko01110//Biosynthesis of secondary metabolites	GO:0016021//integral component of membrane	GO:0004497//monooxygenase activity;GO:0005506//iron ion binding;GO:0016705//oxidoreductase activity, acting on paired donors, with incorporation or reduction of molecular oxygen;GO:0020037//heme binding	GO:0055114//oxidation-reduction process	gi|115399036|ref|XP_001215107.1|/0/conserved hypothetical protein [Aspergillus terreus NIH2624]
4319865	2333	1670.48	501.27	-1.09482754	4.97E-08	4.08E-07	Down	ATEG_04573	dolichol-phosphate mannosyltransferase	ko01100//Metabolic pathways;ko00510//N-Glycan biosynthesis	-	GO:0016757//transferase activity, transferring glycosyl groups	-	gi|115396224|ref|XP_001213751.1|/0/dolichol-phosphate mannosyltransferase [Aspergillus terreus NIH2624]
4316264	6567	3779.56	1135.3	-1.093858271	3.49E-10	3.51E-09	Down	ATEG_01775	hypothetical protein	ko01100//Metabolic pathways;ko00562//Inositol phosphate metabolism;ko04070//Phosphatidylinositol signaling system	GO:0005794//Golgi apparatus;GO:0005829//cytosol;GO:0016021//integral component of membrane	-	GO:0097553//calcium ion transmembrane import into cytosol	gi|115385186|ref|XP_001209140.1|/0/conserved hypothetical protein [Aspergillus terreus NIH2624]
4323106	2934	1149.39	345.41	-1.093404131	1.74E-09	1.65E-08	Down	ATEG_08887	hypothetical protein	-	-	-	-	gi|115402793|ref|XP_001217473.1|/0/conserved hypothetical protein [Aspergillus terreus NIH2624]

4321496	1850	288.54	85.36	-1.090736211	0.00061332	0.00264224	Down	ATEG_05669	hypothetical protein	-	GO:0005634//nucleus	GO:0000166//nucleotide binding;GO:0003723//RNA binding;GO:0004518//nuclease activity;GO:0046872//metal ion binding	GO:0006353//DNA-templated transcription, termination;GO:0006355//regulation of transcription, DNA-templated;GO:0006364//rRNA processing;GO:0006397//mRNA processing;GO:0090305//nucleic acid phosphodiester bond hydrolysis	gi|115398516|ref|XP_001214847.1|/0/conserved hypothetical protein [Aspergillus terreus NIH2624]
4318257	468	208.96	61.65	-1.089397232	0.00290579	0.0106131	Down	ATEG_04017	hypothetical protein	ko00310//Lysine degradation	-	-	-	gi|115391381|ref|XP_001213195.1|/2.5666e-110/predicted protein [Aspergillus terreus NIH2624]
4323550	2500	3895.51	1172.82	-1.088347173	9.36E-15	1.32E-13	Down	ATEG_08764	similar to phosphoadenosine phosphosulfate reductase	ko01100//Metabolic pathways;ko00920//Sulfur metabolism	GO:0005634//nucleus;GO:0005829//cytosol	GO:0004604//phosphoadenylyl-sulfate reductase (thioredoxin) activity	GO:0009086//methionine biosynthetic process;GO:0019379//sulfate assimilation, phosphoadenylyl sulfate reduction by phosphoadenylyl-sulfate reductase (thioredoxin)	gi|115402547|ref|XP_001217350.1|/0/hypothetical protein ATEG_08764 [Aspergillus terreus NIH2624]
4320597	2824	8210.09	2501.16	-1.085586023	8.27E-12	9.69E-11	Down	ATEG_05560	ribonucleoside-diphosphate reductase large chain	ko01100//Metabolic pathways;ko00230//Purine metabolism;ko00240//Pyrimidine metabolism;ko00480//Glutathione metabolism	-	GO:0004748//ribonucleoside-diphosphate reductase activity, thioredoxin disulfide as acceptor;GO:0005524//ATP binding	GO:0006260//DNA replication;GO:0055114//oxidation-reduction process	gi|115398293|ref|XP_001214738.1|/0/ribonucleoside-diphosphate reductase large chain [Aspergillus terreus NIH2624]

4320356	933	427.01	128.55	-1.084482126	4.64E-05	0.00024822	Down	ATEG_04354	hypothetical protein	-	GO:0005783//endoplasmic reticulum;GO:0016021//integral component of membrane	-	-	gi|115395786|ref|XP_001213532.1|/0/conserved hypothetical protein [Aspergillus terreus NIH2624]
4316714	1242	3092.8	940.38	-1.084277943	6.83E-11	7.37E-10	Down	ATEG_02574	mitochondrial protein import protein MAS5	ko04141//Protein processing in endoplasmic reticulum	GO:0005634//nucleus	GO:0005524//ATP binding;GO:0031072//heat shock protein binding;GO:0046872//metal ion binding;GO:0051082//unfolded protein binding	GO:0000226//microtubule cytoskeleton organization;GO:0006457//protein folding;GO:1900035//negative regulation of cellular response to heat	gi|115388493|ref|XP_001211752.1|/0/mitochondrial protein import protein MAS5 [Aspergillus terreus NIH2624]
4322111	2652	1925.31	581.13	-1.083768702	4.31E-11	4.74E-10	Down	ATEG_06542	hypothetical protein	-	GO:0005829//cytosol;GO:0016442//RISC complex	GO:0003712//transcription cofactor activity	GO:0035194//posttranscriptional gene silencing by RNA;GO:1903506//regulation of nucleic acid-templated transcription	gi|115400263|ref|XP_001215720.1|/0/conserved hypothetical protein [Aspergillus terreus NIH2624]
4318281	714	307	91.1	-1.083062193	0.00078418	0.00328188	Down	ATEG_04097	hypothetical protein	ko04120//Ubiquitin mediated proteolysis	-	GO:0003677//DNA binding;GO:0008270//zinc ion binding	-	gi|115391541|ref|XP_001213275.1|/3.11184e-174/predicted protein [Aspergillus terreus NIH2624]
4353371	3069	716.95	216.08	-1.081757127	3.02E-06	1.97E-05	Down	ATEG_08057	plasma membrane ATPase	ko00190//Oxidative phosphorylation	GO:0016021//integral component of membrane	GO:0005524//ATP binding;GO:0008553//hydrogen-exporting ATPase activity, phosphorylative mechanism;GO:0046872//metal ion binding	GO:0006754//ATP biosynthetic process;GO:1902600//hydrogen ion transmembrane transport	gi|115433082|ref|XP_001216678.1|/0/plasma membrane ATPase [Aspergillus terreus NIH2624]

4318677	1332	1310.07	397.06	-1.08018806	1.16E-07	9.10E-07	Down	ATEG_04156	hypothetical protein	ko00970//Aminoacyl-tRNA biosynthesis	-	-	-	gi|115391659|ref|XP_001213334.1|/0/conserved hypothetical protein [Aspergillus terreus NIH2624]
4354353	1200	527.72	158.76	-1.079342875	3.82E-05	0.00020878	Down	ATEG_09695	hypothetical protein	ko00770//Pantothenate and CoA biosynthesis	-	GO:0000287//magnesium ion binding;GO:0008897//holo-[acyl-carrier-protein] synthase activity	GO:0018130;GO:0018958//phenol-containing compound metabolic process;GO:0019438//aromatic compound biosynthetic process;GO:0042181;GO:0043436;GO:0044550//secondary metabolite biosynthetic process;GO:1901362;GO:1901566	gi|115443020|ref|XP_001218317.1|/0/conserved hypothetical protein [Aspergillus terreus NIH2624]
4317809	1200	329.07	97.76	-1.077944573	0.00079761	0.00333214	Down	ATEG_03355	hypothetical protein	ko01100//Metabolic pathways;ko00500//Starch and sucrose metabolism	-	-	-	gi|115390056|ref|XP_001212533.1|/0/predicted protein [Aspergillus terreus NIH2624]
4320619	2313	300.93	89.76	-1.077833901	0.00047312	0.00209203	Down	ATEG_05578	hypothetical protein	-	-	GO:0008270//zinc ion binding	-	gi|115398329|ref|XP_001214756.1|/0/predicted protein [Aspergillus terreus NIH2624]
4317416	540	588.9	178.37	-1.077008837	4.29E-06	2.71E-05	Down	ATEG_03209	protein ORM1	ko01100//Metabolic pathways;ko00520//Amino sugar and nucleotide sugar metabolism	GO:0016021//integral component of membrane;GO:0035339//SPOTS complex	-	GO:0006986//response to unfolded protein;GO:0090155//negative regulation of sphingolipid biosynthetic process;GO:0090156//cellular sphingolipid homeostasis	gi|115389764|ref|XP_001212387.1|/1.80179e-133/protein ORM1 [Aspergillus terreus NIH2624]

4316205	543	156.38	46.58	-1.075427318	0.00957442	0.02977687	Down	ATEG_01380	hypothetical protein	-	-	-	-	gi|115384396|ref|XP_001208745.1|/2.19208e-139/predicted protein [Aspergillus terreus NIH2624]
4322153	1002	319.9	96.56	-1.07505518	0.00053452	0.0023349	Down	ATEG_06699	hypothetical protein	ko01100//Metabolic pathways;ko00790//Folate biosynthesis	-	GO:0008270//zinc ion binding;GO:0016491//oxidoreductase activity	GO:0055114//oxidation-reduction process	gi|115400577|ref|XP_001215877.1|/0/predicted protein [Aspergillus terreus NIH2624]
4315875	1587	578.34	176.83	-1.072908962	0.00021206	0.00100492	Down	ATEG_01506	similar to pi-transporter A-1	-	GO:0016021//integral component of membrane	GO:0022857//transmembrane transporter activity	GO:0055085//transmembrane transport	gi|115384648|ref|XP_001208871.1|/0/hypothetical protein ATEG_01506 [Aspergillus terreus NIH2624]

4322013	2107	7508.22	2290.73	-1.071413181	6.77E-17	1.11E-15	Down	ATEG_06808	branched-chain-amino-acid aminotransferase	ko01100//Metabolic pathways;ko01110//Biosynthesis of secondary metabolites;ko01130//Biosynthesis of antibiotics;ko01230//Biosynthesis of amino acids;ko00280//Valine, leucine and isoleucine degradation;ko00270//Cysteine and methionine metabolism;ko01210//2-Oxocarboxylic acid metabolism;ko00770//Pantothenate and CoA biosynthesis;ko00290//Valine, leucine and isoleucine biosynthesis	-	GO:0052654//L-leucine transaminase activity;GO:0052655//L-valine transaminase activity;GO:0052656//L-isoleucine transaminase activity	GO:0009082//branched-chain amino acid biosynthetic process	gi|115400795|ref|XP_001215986.1|/0/branched-chain-amino-acid aminotransferase [Aspergillus terreus NIH2624]
4354106	3845	1579.72	479.91	-1.070093101	1.33E-09	1.26E-08	Down	ATEG_09756	hypothetical protein	ko01100//Metabolic pathways;ko00564//Glycerophospholipid metabolism;ko00565//Ether lipid metabolism	-	-	-	gi|115443142|ref|XP_001218378.1|/2.66101e-171/conserved hypothetical protein [Aspergillus terreus NIH2624]
4323565	1086	263.93	79.72	-1.067242715	0.0008252	0.00343059	Down	ATEG_08716	hypothetical protein	ko01100//Metabolic pathways;ko00500//Starch and sucrose metabolism	-	GO:0003677//DNA binding	-	gi|115402451|ref|XP_001217302.1|/0/predicted protein [Aspergillus terreus NIH2624]

4316601	1128	5309.46	1616.77	-1.063205652	1.22E-10	1.29E-09	Down	ATEG_01943	L-asparaginase precursor	ko01100//Metabolic pathways;ko01110//Biosynthesis of secondary metabolites;ko00250//Alanine, aspartate and glutamate metabolism;ko00460//Cyanoamino acid metabolism	-	GO:0004067//asparaginase activity	GO:0006528//asparagine metabolic process	gi|115387231|ref|XP_001211121.1|/0/L-asparaginase precursor [Aspergillus terreus NIH2624]
4323261	1188	681.01	209.43	-1.059967376	6.03E-06	3.71E-05	Down	ATEG_08727	hypothetical protein	-	GO:0016021//integral component of membrane	-	GO:0055085//transmembrane transport	gi|115402473|ref|XP_001217313.1|/0/conserved hypothetical protein [Aspergillus terreus NIH2624]
4319513	3195	145.71	43.64	-1.056448282	0.01013682	0.03127229	Down	ATEG_09993	similar to cellobiose dehydrogenase	-	GO:0016020//membrane	GO:0016614//oxidoreductase activity, acting on CH-OH group of donors;GO:0050660//flavin adenine dinucleotide binding	GO:0055114//oxidation-reduction process	gi|115385497|ref|XP_001209295.1|/0/hypothetical protein ATEG_09993 [Aspergillus terreus NIH2624]
4320851	2023	556.33	170.64	-1.05636161	2.29E-05	0.00012955	Down	ATEG_05173	hypothetical protein	ko01100//Metabolic pathways;ko00230//Purine metabolism;ko00250//Alanine, aspartate and glutamate metabolism	GO:0016021//integral component of membrane	-	GO:0055085//transmembrane transport	gi|115397519|ref|XP_001214351.1|/0/conserved hypothetical protein [Aspergillus terreus NIH2624]
4317040	1179	2707.85	840.21	-1.05630699	1.21E-10	1.28E-09	Down	ATEG_02173	pre-mRNA splicing factor slt-11	ko03040//Spliceosome	GO:0000974//Prp19 complex;GO:0005681//spliceosomal complex	GO:0000166//nucleotide binding;GO:0003676//nucleic acid binding	GO:0000398//mRNA splicing, via spliceosome	gi|115387691|ref|XP_001211351.1|/0/pre-mRNA splicing factor slt-11 [Aspergillus terreus NIH2624]

4354272	1243	229.18	69.87	-1.052333584	0.00204218	0.00777985	Down	ATEG_09670	hypothetical protein	-	-	GO:0010181//FMN binding;GO:0016491//oxidoreductase activity	GO:0055114//oxidation-reduction process	gi|115442970|ref|XP_001218292.1|/0/conserved hypothetical protein [Aspergillus terreus NIH2624]
4355472	1092	558.36	172.45	-1.04907057	9.91E-05	0.00049755	Down	ATEG_00717	hypothetical protein	ko00920//Sulfur metabolism	-	GO:0016705//oxidoreductase activity, acting on paired donors, with incorporation or reduction of molecular oxygen	GO:0055114//oxidation-reduction process	gi|115492351|ref|XP_001210803.1|/0/conserved hypothetical protein [Aspergillus terreus NIH2624]
4323317	5031	4297.09	1328.61	-1.048972484	1.29E-13	1.70E-12	Down	ATEG_08800	hypothetical protein	-	GO:0005634//nucleus	GO:0000981//RNA polymerase II transcription factor activity, sequence-specific DNA binding;GO:0008270//zinc ion binding	GO:0006357//regulation of transcription from RNA polymerase II promoter	gi|115402619|ref|XP_001217386.1|/0/conserved hypothetical protein [Aspergillus terreus NIH2624]
4318680	975	3074.02	954.24	-1.048962259	9.71E-14	1.29E-12	Down	ATEG_03678	hypothetical protein	ko01100//Metabolic pathways;ko00500//Starch and sucrose metabolism	GO:0005886//plasma membrane;GO:0016021//integral component of membrane	-	-	gi|115390703|ref|XP_001212856.1|/0/conserved hypothetical protein [Aspergillus terreus NIH2624]
4316336	1464	1690.37	521.31	-1.048777164	1.25E-08	1.07E-07	Down	ATEG_01155	hypothetical protein	ko04141//Protein processing in endoplasmic reticulum	GO:0000329//fungal-type vacuole membrane;GO:0005783//endoplasmic reticulum;GO:0005794//Golgi apparatus;GO:0016021//integral component of membrane	-	-	gi|115383946|ref|XP_001208520.1|/0/conserved hypothetical protein [Aspergillus terreus NIH2624]

4320715	1455	187.7	57.25	-1.045542096	0.00503078	0.01715253	Down	ATEG_05104	phosphatidylinositol N-acetylglucosaminyltransferase GPI3 subunit	ko01100//Metabolic pathways;ko00563//Glycosylphosphatidylinositol(GPI)-anchor biosynthesis	GO:0005783//endoplasmic reticulum;GO:0016021//integral component of membrane	GO:0017176//phosphatidylinositol N-acetylglucosaminyltransferase activity	GO:0006506//GPI anchor biosynthetic process	gi|115397381|ref|XP_001214282.1|/0/phosphatidylinositol N-acetylglucosaminyltransferase GPI3 subunit [Aspergillus terreus NIH2624]
4322050	1344	333.99	102.5	-1.045227459	0.0002974	0.00137598	Down	ATEG_06753	hypothetical protein	-	-	-	-	gi|115400685|ref|XP_001215931.1|/3.09006e-70/predicted protein [Aspergillus terreus NIH2624]
4353494	1037	2078.15	646.66	-1.043779843	1.61E-10	1.68E-09	Down	ATEG_08274	similar to aldo-keto reductase	ko01100//Metabolic pathways;ko00051//Fructose and mannose metabolism;ko00650//Butanoate metabolism;ko00591//Linoleic acid metabolism	GO:0005634//nucleus;GO:0005829//cytosol	GO:0016491//oxidoreductase activity	GO:0055114//oxidation-reduction process	gi|115433516|ref|XP_001216895.1|/8.08114e-117/hypothetical protein ATEG_08274 [Aspergillus terreus NIH2624]
4353666	1407	330.03	101.91	-1.043561132	0.000409	0.00183181	Down	ATEG_09449	hypothetical protein	-	-	-	-	gi|115438458|ref|XP_001218071.1|/0/conserved hypothetical protein [Aspergillus terreus NIH2624]
4353136	729	707.47	216.32	-1.043479987	0.00015096	0.00073467	Down	ATEG_08094	hypothetical protein	ko03060//Protein export	GO:0005787//signal peptidase complex;GO:0016021//integral component of membrane	GO:0008233//peptidase activity	GO:0006465//signal peptide processing;GO:0045047//protein targeting to ER	gi|115433156|ref|XP_001216715.1|/2.3721e-176/conserved hypothetical protein [Aspergillus terreus NIH2624]

4320142	678	292.06	89.56	-1.043115022	0.0010886	0.00441415	Down	ATEG_04884	hypothetical protein	ko00910//Nitrogen metabolism	-	-	-	gi|115396846|ref|XP_001214062.1|/1.12651e-161/conserved hypothetical protein [Aspergillus terreus NIH2624]
4319916	1848	868.95	266.05	-1.042165452	4.72E-05	0.00025218	Down	ATEG_04506	hypothetical protein	-	-	GO:0003676//nucleic acid binding;GO:0046872//metal ion binding	-	gi|115396090|ref|XP_001213684.1|/0/conserved hypothetical protein [Aspergillus terreus NIH2624]
4353854	1737	3190.42	999.96	-1.041140999	1.08E-10	1.15E-09	Down	ATEG_09149	similar to purine transporter	-	GO:0016021//integral component of membrane	GO:0005345//purine nucleobase transmembrane transporter activity	GO:1904823//purine nucleobase transmembrane transport	gi|115437282|ref|XP_001217771.1|/0/hypothetical protein ATEG_09149 [Aspergillus terreus NIH2624]
4317619	2106	749.15	230.76	-1.038683963	1.36E-05	7.94E-05	Down	ATEG_03499	similar to stxbp-unc-18-sec1 family protein	-	GO:0005829//cytosol	-	GO:0006904//vesicle docking involved in exocytosis	gi|115390344|ref|XP_001212677.1|/0/hypothetical protein ATEG_03499 [Aspergillus terreus NIH2624]
4317407	2377	800.21	249.5	-1.038582412	4.12E-07	3.03E-06	Down	ATEG_02959	vacuolar protein sorting-associated protein 74	-	GO:0005634//nucleus;GO:0005829//cytosol	GO:0070273//phosphatidylinositol-4-phosphate binding	GO:0048193//Golgi vesicle transport	gi|115389264|ref|XP_001212137.1|/0/vacuolar protein sorting-associated protein 74 [Aspergillus terreus NIH2624]
4322254	5995	2864.1	896.25	-1.038317758	1.13E-10	1.20E-09	Down	ATEG_06420	hypothetical protein	-	GO:0005634//nucleus;GO:0016021//integral component of membrane	GO:0000981//RNA polymerase II transcription factor activity, sequence-specific DNA binding;GO:0003677//DNA binding;GO:0008270//zinc ion binding	GO:0006357//regulation of transcription from RNA polymerase II promoter;GO:0009410//response to xenobiotic stimulus	gi|115400019|ref|XP_001215598.1|/0/predicted protein [Aspergillus terreus NIH2624]

4315858	1074	317.65	97.35	-1.037589916	0.00111161	0.00449579	Down	ATEG_01843	hypothetical protein	-	GO:0016021//integral component of membrane	-	-	gi|115385322|ref|XP_001209208.1|/0/conserved hypothetical protein [Aspergillus terreus NIH2624]
4354284	4788	2953.23	923.57	-1.037102772	1.63E-13	2.13E-12	Down	ATEG_09873	DNA replication licensing factor mcm4	ko04111//Cell cycle - yeast;ko04113//Meiosis - yeast;ko03030//DNA replication	GO:0030875//rDNA protrusion;GO:0042555//MCM complex;GO:0097373//MCM core complex	GO:0003682//chromatin binding;GO:0003697//single-stranded DNA binding;GO:0003724//RNA helicase activity;GO:0003727//single-stranded RNA binding;GO:0005524//ATP binding;GO:0033679//3'-5' DNA/RNA helicase activity;GO:1990518//single-stranded DNA-dependent ATP-dependent 3'-5' DNA helicase activitv	GO:1902450//negative regulation of ATP-dependent DNA helicase activity;GO:1902975//mitotic DNA replication initiation	gi|115443376|ref|XP_001218495.1|/0/DNA replication licensing factor mcm4 [Aspergillus terreus NIH2624]
4322689	717	4228.77	1320.29	-1.036912481	2.46E-13	3.19E-12	Down	ATEG_07534	hypothetical protein	ko00480//Glutathione metabolism	GO:0005737//cytoplasm	GO:0004364//glutathione transferase activity	GO:0006749//glutathione metabolic process;GO:0009407//toxin catabolic process	gi|115401134|ref|XP_001216155.1|/3.22644e-172/conserved hypothetical protein [Aspergillus terreus NIH2624]

4316302	7237	1099.7	342.58	-1.035761519	9.27E-08	7.35E-07	Down	ATEG_01538	similar to DNA polymerase II subunit epsilon	ko01100//Metabolic pathways;ko00230//Purine metabolism;ko00240//Pyrimidine metabolism;ko03420//Nucleotide excision repair;ko03030//DNA replication;ko03410//Base excision repair	GO:0008622//epsilon DNA polymerase complex	GO:0000166//nucleotide binding;GO:0003677//DNA binding;GO:0003887//DNA-directed DNA polymerase activity;GO:0008270//zinc ion binding;GO:0008408//3'-5' exonuclease activity	GO:0006260//DNA replication;GO:0006281//DNA repair;GO:0071897//DNA biosynthetic process;GO:0090305//nucleic acid phosphodiester bond hydrolysis	gi|115384712|ref|XP_001208903.1|/0/hypothetical protein ATEG_01538 [Aspergillus terreus NIH2624]
4316495	1167	1018.86	320.25	-1.028773902	5.24E-08	4.29E-07	Down	ATEG_01991	hypothetical protein	ko04144//Endocytosis	-	-	-	gi|115387327|ref|XP_001211169.1|/0/predicted protein [Aspergillus terreus NIH2624]
4317956	3330	146.8	45.05	-1.026862947	0.01228764	0.03702471	Down	ATEG_03540	alpha-N-arabinofuranosidase A precursor	ko00520//Amino sugar and nucleotide sugar metabolism	-	GO:0046556//alpha-L-arabinofuranosidase activity	GO:0046373//L-arabinose metabolic process	gi|115390426|ref|XP_001212718.1|/0/alpha-N-arabinofuranosidase A precursor [Aspergillus terreus NIH2624]
4316908	2511	313.74	98.38	-1.021411927	0.00110699	0.00448097	Down	ATEG_02436	hypothetical protein	-	-	GO:0008168//methyltransferase activity	GO:0032259//methylation	gi|115388217|ref|XP_001211614.1|/0/conserved hypothetical protein [Aspergillus terreus NIH2624]

4354802	1221	350.95	109.21	-1.020639856	0.0009042	0.0037211	Down	ATEG_00046	cystathionine gamma-lyase	ko01100//Metabolic pathways;ko01130//Biosynthesis of antibiotics;ko01230//Biosynthesis of amino acids;ko00260//Glycine, serine and threonine metabolism;ko00270//Cysteine and methionine metabolism;ko00450//Selenocompound metabolism	-	GO:0004121//cystathionine beta-lyase activity;GO:0004123//cystathionine gamma-lyase activity;GO:0030170//pyridoxal phosphate binding;GO:0042802//identical protein binding	GO:0009086//methionine biosynthetic process;GO:0019343//cysteine biosynthetic process via cystathionine;GO:0019346//transsulfuration	gi|115491009|ref|XP_001210132.1|/0/cystathionine gamma-lyase [Aspergillus terreus NIH2624]	
4317106	1428	601.9	190.54	-1.019311904	2.12E-05	0.00012059	Down	ATEG_02181	hypothetical protein	ko01100//Metabolic pathways;ko01110//Biosynthesis of secondary metabolites;ko01130//Biosynthesis of antibiotics	-	GO:0016787//hydrolase activity	-	gi|115387707|ref|XP_001211359.1|/0/conserved hypothetical protein [Aspergillus terreus NIH2624]	
4321038	444	614.17	192.46	-1.019021669	0.00458974	0.01586912	Down	ATEG_05291	hypothetical protein	-	GO:0005634//nucleus;GO:0005829//cytosol	-	-	gi|115397755|ref|XP_001214469.1|/1.57199e-107/conserved hypothetical protein [Aspergillus terreus NIH2624]	

4353270	2834	897.23	286.09	-1.017737244	0.00010575	0.00052694	Down	ATEG_08346	acetolactate synthase, mitochondrial precursor	ko01100//Metabolic pathways;ko01110//Biosynthesis of secondary metabolites;ko01130//Biosynthesis of antibiotics;ko01230//Biosynthesis of amino acids;ko00650//Butanoate metabolism;ko01210//2-Oxocarboxylic acid metabolism;ko00770//Pantothenate and CoA biosynthesis;ko00290//Valine, leucine and isoleucine biosynthesis;ko00660//C5-Branched dibasic acid metabolism	GO:0005948//acetolactate synthase complex	GO:0000287//magnesium ion binding;GO:0003984//acetolactate synthase activity;GO:0030976//thiamine pyrophosphate binding;GO:0050660//flavin adenine dinucleotide binding	GO:0009097//isoleucine biosynthetic process;GO:0009099//valine biosynthetic process	gi|115433660|ref|XP_001216967.1|/0/acetolactate synthase, mitochondrial precursor [Aspergillus terreus NIH2624]
4319178	1012	347.07	107.75	-1.01682867	0.00210354	0.00797474	Down	ATEG_07033	hypothetical protein	ko04146//Peroxisome;ko00071//Fatty acid degradation	-	GO:0016853//isomerase activity	GO:0008152//metabolic process	gi|115386356|ref|XP_001209719.1|/0/conserved hypothetical protein [Aspergillus terreus NIH2624]
4321548	1486	381.52	119.64	-1.01680438	0.00041563	0.0018606	Down	ATEG_06231	tubulin beta-1 chain	ko04145//Phagosome	GO:0005737//cytoplasm;GO:0005874//microtubule	GO:0003924//GTPase activity;GO:0005200//structural constituent of cytoskeleton;GO:0005525//GTP binding	GO:0007017//microtubule-based process	gi|115399706|ref|XP_001215409.1|/0/tubulin beta-1 chain [Aspergillus terreus NIH2624]

4353631	981	355.62	108.38	-1.016637243	0.00892775	0.02811043	Down	ATEG_09086	hypothetical protein	ko01100//Metabolic pathways;ko01220//Degradation of aromatic compounds	-	GO:0016491//oxidoreductase activity;GO:0071949//FAD binding	GO:0055114//oxidation-reduction process	gi|115437032|ref|XP_001217708.1|/0/conserved hypothetical protein [Aspergillus terreus NIH2624]
4318403	2658	1213.54	384.74	-1.016249084	1.29E-07	1.01E-06	Down	ATEG_03913	hypothetical protein	ko03013//RNA transport	-	-	-	gi|115391173|ref|XP_001213091.1|/0/predicted protein [Aspergillus terreus NIH2624]
4316907	780	298.04	92.99	-1.015836553	0.00145869	0.00574595	Down	ATEG_02435	hypothetical protein	-	-	-	-	gi|115388215|ref|XP_001211613.1|/0/predicted protein [Aspergillus terreus NIH2624]
4355688	1448	868.13	270.67	-1.015789273	3.32E-05	0.00018252	Down	ATEG_00925	hypothetical protein	-	GO:0016021//integral component of membrane	-	GO:0006950//response to stress	gi|115492767|ref|XP_001211011.1|/0/conserved hypothetical protein [Aspergillus terreus NIH2624]

4322059	2202	1527.24	485.92	-1.014335635	8.53E-08	6.79E-07	Down	ATEG_06424	hypothetical protein	-	GO:0000790//nuclear chromatin;GO:0005829//cytosol;GO:0031298//replication fork protection complex;GO:0034507//chromosome, centromeric outer repeat region;GO:0035101//FACT complex	GO:0003677//DNA binding	GO:0000070//mitotic sister chromatid segregation;GO:0006261//DNA-dependent DNA replication;GO:0006281//DNA repair;GO:0030466//chromatin silencing at silent mating-type cassette;GO:0034613//cellular protein localization;GO:0034724//DNA replication-independent nucleosome organization;GO:0045899//positive regulation of RNA polymerase II transcriptional preinitiation complex assembly;GO:1990141//chromatin silencing at centromere outer repeat region	gi|115400027|ref|XP_001215602.1|/0/conserved hypothetical protein [Aspergillus terreus NIH2624]
4316702	717	433.23	136.13	-1.013695883	0.00011452	0.00056794	Down	ATEG_02286	hypothetical protein	-	-	-	-	gi|115387917|ref|XP_001211464.1|/0/conserved hypothetical protein [Aspergillus terreus NIH2624]
4353464	2106	9746.47	3097.55	-1.011595789	5.24E-07	3.78E-06	Down	ATEG_07996	heat shock protein 82	ko04141//Protein processing in endoplasmic reticulum	-	GO:0005524//ATP binding;GO:0051082//unfolded protein binding	GO:0006457//protein folding;GO:0006950//response to stress	gi|115432960|ref|XP_001216617.1|/0/heat shock protein 82 [Aspergillus terreus NIH2624]

4316129	834	550.34	174.05	-1.010929438	1.74E-05	1.00E-04	Down	ATEG_01718	hypothetical protein	-	GO:0016021//integral component of membrane	-	-	gi|115385072|ref|XP_001209083.1|/0/conserved hypothetical protein [Aspergillus terreus NIH2624]
4321916	456	192.24	59.71	-1.010217332	0.00606166	0.02014043	Down	ATEG_06830	hypothetical protein	-	GO:0005737//cytoplasm;GO:0016021//integral component of membrane	-	-	gi|115400839|ref|XP_001216008.1|/6.85861e-104/conserved hypothetical protein [Aspergillus terreus NIH2624]
4319359	533	331.2	105.02	-1.005568939	0.00050715	0.0022278	Down	ATEG_07123	hypothetical protein	-	-	-	-	gi|115386536|ref|XP_001209809.1|/8.26361e-180/predicted protein [Aspergillus terreus NIH2624]
4316179	1062	807.48	258.73	-1.005113724	4.37E-06	2.76E-05	Down	ATEG_01423	hypothetical protein	ko01100//Metabolic pathways;ko01110//Biosynthesis of secondary metabolites;ko01212//Fatty acid metabolism;ko01040//Biosynthesis of unsaturated fatty acids;ko00062//Fatty acid elongation	GO:0005789//endoplasmic reticulum membrane;GO:0016021//integral component of membrane	GO:0045703//ketoreductase activity	GO:0030497//fatty acid elongation;GO:0055114//oxidation-reduction process	gi|115384482|ref|XP_001208788.1|/0/conserved hypothetical protein [Aspergillus terreus NIH2624]
4322855	5478	4617.51	1473.38	-1.004217624	6.40E-13	8.18E-12	Down	ATEG_07428	hypothetical protein	ko04144//Endocytosis	-	-	-	gi|115400922|ref|XP_001216049.1|/0/predicted protein [Aspergillus terreus NIH2624]

4323538	1878	2017.16	645.02	-1.00153066	7.95E-10	7.72E-09	Down	ATEG_08515	similar to TPA: TPA_exp: carboxypeptidase	-	GO:0005794//Golgi apparatus;GO:0016021//integral component of membrane	GO:0004185//serine-type carboxypeptidase activity	GO:0006508//proteolysis;GO:0006915//apoptotic process	gi|115402049|ref|XP_001217101.1|/0/hypothetical protein ATEG_08515 [Aspergillus terreus NIH2624]
